# KDM2B and its peptides promote the stem cells from apical papilla mediated nerve injury repair in rats by intervening EZH2 function

**DOI:** 10.1111/cpr.13756

**Published:** 2024-10-02

**Authors:** Yangyang Cao, Yantong Wang, Dengsheng Xia, Zhipeng Fan

**Affiliations:** ^1^ Laboratory of Molecular Signaling and Stem Cells Therapy, Beijing Key Laboratory of Tooth Regeneration and Function Reconstruction Capital Medical University School of Stomatology Beijing China; ^2^ Department of General Dentistry and Integrated Emergency Dental Care Capital Medical University School of Stomatology Beijing China; ^3^ Beijing Laboratory of Oral Health Capital Medical University School Beijing China; ^4^ Research Unit of Tooth Development and Regeneration Chinese Academy of Medical Sciences Beijing China

## Abstract

How to improve the neurogenic potential of mesenchymal stem cells (MSCs) and develop biological agent based on the underlying epigenetic mechanism remains a challenge. Here, we investigated the effect of histone demethylase Lysine (K)‐specific demethylase 2B (KDM2B) on neurogenic differentiation and nerve injury repair by using MSCs from dental apical papilla (SCAP). We found that KDM2B promoted the neurogenic indicators expression and neural spheres formation in SCAP, and modified the Histone H3K4 trimethylation (H3K4me3) methylation on neurogenesis‐related genes. KDM2B improved the SCAP mediated recovery of motor ability at the early healing stage of spinal cord injury rats. Meanwhile, KDM2B acted as a negative regulator to its partner EZH2 during neurogenic differentiation, enhancer of zeste homologue 2 (EZH2) suppressed the neurogenic ability of SCAP. Further, the protein interaction between KDM2B and EZH2 was identified which decreased during neurogenic differentiation. On this basis, we revealed seven key protein binding sequences of KDM2B to EZH2, and synthesized KDM2B‐peptides based on these sequences. By the usage of KDM2B‐peptides, EZH2 function was effectively intervened and the neurogenic ability of SCAP was promoted. More, KDM2B‐peptides significantly improved the SCAP mediated functional recovery at SCI early phase. Our study revealed that KDM2B acted as a promotor to neurogenic differentiation ability of dental MSCs through binding and negatively regulating EZH2, and provided the KDM2B‐peptides as candidate agents for improving the neurogenic ability of MSCs and nerve injury repair.

## Graphical abstract

KDM2B promote the neurogenesis of stem cells from apical papilla (SCAP), through up‐regulating mitogen‐activated protein kinase‐1 (MAP2K1), PBX3, Neuregulin‐1 (NRG1), neurofilament heavy (NEFH) and Cadherin‐2 (CDH2) via H3K4me3 methylation. Enhancer of zeste homologue 2 (EZH2) inhibited the neurogenic differentiation in SCAP, and was negatively regulated by KDM2B during neurogenic induction. KDM2B interacted with EZH2, and the binding between KDM2B and EZH2 was decreased during neurogenic induction. KDM2B‐peptides were synthesized basing on KDM2B binding sequences with EZH2. By using KDM2B‐peptides, the binding of EZH2 with KDM2B was effectively decreased. And KDM2B‐peptides promoted the neurogenic differentiation ability of SCAP by intervening EZH2 function. The usage of KDM2B‐peptides treated SCAP improved the motor ability of SCI rats.

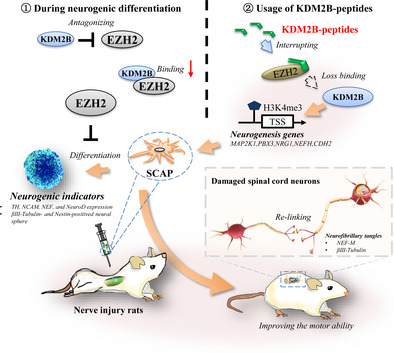

## 1 INTRODUCTION

Nerve injury such as spinal cord injury (SCI) accounts for a large proportion of global injury burden, which includes the physical, emotional and financial burden on patients and society.^1^, ^2^, ^3^ The therapy for nerve injury such as surgery and neuroprotective treatments mainly stimulate the re‐assembly and re‐link of microtubule in injured nerve fibres.^3^, ^4^ However, the analysis of SCI revealed that only one third cases existed functional improvement after treatment.^5^, ^6^ These suggest that the self‐healing ability of injured nerve fibre is not effective. Nowadays, clinical trials have proceeded in SCI patients by transplanting mesenchymal stem cells (MSCs) or neural tissue‐derived cells such as neural stem cells (NSCs).^7^, ^8^, ^9^, ^10^, ^11^ NSCs exhibit the repair potential to nerve injury based on its ability to secret neurotrophic factors and to differentiate neurogenic cells.^12^, ^13^, ^14^ But some evidence shows that autologous NSCs existed in injury location do not have enough effect to support the recovery of nerve injury.^1^ The obtain sources of NSCs are limited, with inevitably damage to donor nerve tissue during obtain process. Recently study shows that the transplantation of human dental apical papilla could support the recovery of SCI in rat.^15^ Human dental apical papilla origin from neural crest, stem cells from dental apical papilla (SCAP) could secret neurotrophic factors such as neural cell adhesion molecule (NCAM) and tyrosine hydroxylase (TH), and differentiate into neuron‐like cells.^16^, ^17^, ^18^, ^19^, ^20^ The transplantation of SCAP could promote the recovery of SCI in rats.^19^, ^21^ But its neurogenic differentiation ability still needs to be promoted for further application.

Epigenetic modification has considered to correlate with the differentiation and lineage maintenance of neurogenic cells, especially histone methylation Histone H3K4 trimethylation (H3K4me3) and Histone H4 acethylated at lysine 16 (H4K16ac).^22^, ^23^, ^24^ Lysine‐specific demethylase 2B (KDM2B) is a histone demethylase, and removes H3K4me3.^25^ Study has report that KDM2B could upregulate the expression of neurogenic genes neuron specific gene 2 (Nsg2) in haematopoietic stem cells by removing H3K4me3 modification, and promotes the formation and migration of neurons.^26^ KDM2B overexpression promotes neural differentiation and neurite elongation in NSCs.^27^ Knock‐out of KDM2B causes the neural tube closure disorders and brain malformations in mice.^28^ These evidences suggest that KDM2B might correlate with the differentiation and lineage maintenance of neurogenic cells. However, it is still insufficient about KDM2B to the neurogenic differentiation of dental MSCs. How to develop biological agent based on the underlying mechanism regulated by KDM2B also remains a challenge.

Previous studies reported that KDM2B acts as a component of the polycomb repressive complex 1 (PRC1) and regulates the expression of key gene which participating in metabolism, cell differentiation, and morphogenesis. The non‐classical PRC1‐KDM2B complex recruited the PRC2 complex to bind with the CpG region.^29^, ^30^ Enhancer of zeste homologue 2 (EZH2) is the core subunit of PRC2 complex, which binding with embryo development of the ectoderm protein (EED) and suppressor of zeste 12 (SUZ12). During the senescence of mouse embryonic fibroblasts, KDM2B regulates let‐7, which following affects EZH2 expression.^31^ As one core epigenetic factor of H3K4me3, KDM2B contains four mainly protein domains including the Jumonji C (JmjC), Cys‐Xaa‐Xaa‐Cys (CXXC), Prolyl Hydroxylase Domain (PHD) and Leucine‐rich repeat.^25^ Study reports that the CXXC domain of KDM2B is essential for the DNA binding capacity and regulates the senescence process of primary cell.^32^ The immunoprecipitation analysis reveals that KDM2B and EZH2 exist protein binding in Human Embryonic Kidney 293T (HEK293T) cells.^33^ These results suggest that epigenetic factors KDM2B and EZH2 exist functional interaction. But the key protein interaction sites between KDM2B and EZH2 is unclear. How to improve the neurogenic ability of MSCs and nerve injury repair based on the protein interaction between KDM2B and EZH2 is still need study.

In this study, we aimed to reveal the role and interaction mechanism of KDM2B and EZH2 on neurogenic differentiation by using SCAP, and provide candidate agent for nerve injury repair. We found that KDM2B acted as a promotor to neurogenesis ability of dental MSCs in vivo/vitro. Notably, KDM2B bound and negatively regulated its partner EZH2. Based on this, KDM2B‐peptides were synthesized, which might develop as candidate agent for improving the neurogenic ability of MSCs and the recovery of nerve injury repair at the early healing stage.

## 2 MATERIALS AND METHODS

Ethics statement: The human stem cells research in this study conform to the ISSCR ‘Guidelines for the Conduct of Human Embryonic Stem Cell Research’ (Ethics committee approval number: CMUSH‐IRB‐KJ‐PJ‐2022‐24). Our research was approved by the animal care committee and was performed in accordance with the animal experimentation ordinance of the Beijing Stomatological Hospital, Capital Medical University (Approval of Animal Ethical and Welfare: KQYY‐202110‐003).

### 2.1 Mesenchymal stem cells culture

SCAP were derived from dental apical papilla as described.^34^, ^35^ flow cytometry analysed the MSCs surface markers included CD34 (Catalogue No. ab81289; Abcam, Cambridge, UK), CD45 (Catalogue No. ab10558; Abcam), CD90 (Catalogue No. ab225; Abcam) and CD105 (Catalogue No. ab11414; Abcam). The culture medium was Alpha‐Modified Eagle Medium (α‐MEM) (Invitrogen) with 15% fetal bovine serum (FBS; Invitrogen), 100 U/mL penicillin and 100 μg/mL streptomycin (Invitrogen), and the passage 3–5 of SCAP were used for study.

Human embryonic kidney 293 T cells, for packaging viral constructs, were maintained in Dulbecco's modified Eagle's medium (DMEM) (Invitrogen) with 10% FBS, 100 U/mL penicillin, and 100 μg/mL streptomycin (Invitrogen).

### 2.2 Plasmid construction and viral infection

As described in our previous study,^34^ the plasmids of short hairpin RNA (shRNA) with specific target sequences of EZH2 or KDM2B (pLKO.1 lentiviral vector; Addgene, Cambridge, USA) and full‐length gene sequence with an HA‐tag of EZH2 or KDM2B (HA‐pQCXIN retroviral vector; BD Biosciences) were constructed, verified and obtained; subsequently, the virus was packaged; and the SCAP were infected with the retro‐ or lentivirus; the infected cells were selected with 60 μg/mL neomycin for the pQCXIN vector or 2 μg/mL puromycin for the pLKO.1 vector. The control of scramble shRNA (Scramsh) was purchased from Addgene (Cambridge, MA, USA). The target sequences were as follows: EZH2 shRNA (EZH2sh), 5′‐GCTGATGAAGTAAAGAGTATG‐3′ and KDM2B shRNA (KDM2Bsh), 5′‐GAGTCAAGACGTAGAATAA‐3′.

### 2.3 Reverse transcriptase‐polymerase chain reaction and real‐time RT‐PCR

As previous described,^34^ the total RNA of SCAP was extracted with TRIzol™ Reagent (Invitrogen) and cDNA was synthesized according to the manufacturer's protocol (Invitrogen). Real‐time reverse transcriptase‐polymerase chain reaction (RT‐PCR) tests were performed with the QuantiTect SYBR Green PCR kit (Qiagen, Hilden, Germany) and an iCycler iQ™ Multi‐colour Realtime PCR detection system, according to the standard protocol. The sequences of all primers are listed in Table S1.

### 2.4 Co‐Immunoprecipitation

The total protein of SCAP was extracted by Pierce IP Lysis Buffer (Invitrogen) and the co‐immunoprecipitation (Co‐IP) analysis was performed with the Pierce™ Direct IP Kit (Thermo Fisher Scientific, Waltham, USA), according to the standard protocol. Subsequently, 400 μg of total protein were incubated with beads cross‐linked with antibodies overnight at 4°C, and eluted the next day. Thereafter, the protein samples were denatured by boiling and subjected to SDS‐polyacrylamide gel electrophoresis analysis. The primary antibodies were anti‐KDM2B (Catalogue No. 17–10,264; Millipore, Darmstadt, Germany), anti‐EZH2 (Catalogue No. ab191250; Abcam), normal rabbit IgG (Catalogue No. ab172730; Abcam) and normal mouse IgG (Catalogue No. sc‐2025; Santa Cruz Biotechnology, USA).

### 2.5 Western blot

Total protein of SCAP was extracted, loaded and separated via SDS‐polyacrylamide gel electrophoresis. The primary antibodies were anti‐KDM2B (Catalogue No. 17–10,264; Millipore), anti‐HA (Catalogue No. ab9110; Abcam), anti‐EZH2 (Catalogue No. ab191250; Abcam), anti‐EED (Catalogue No. ab126542; Abcam), anti‐SUZ12 (Catalogue No. ab175187; Abcam), normal rabbit IgG (Catalogue No. ab172730; Abcam) and normal mouse IgG (Catalogue No. sc‐2025; Santa Cruz Biotechnology, USA). Anti‐beta‐actin (Catalogue No. C1313; APPLYGEN, China) and anti‐histone H3 (Catalogue No. ab1791; Abcam) were used as internal control protein.

### 2.6 Neural sphere formation analysis

As previously described,^34^ 1 × 10^6^ SCAP were cultured into a non‐adherent culture dish (Corning, NY, USA). The medium used for neurogenic induction was Neurobasal‐A medium (Gibco, USA) containing B27 supplement (Gibco), 40 ng/mL bFGF (Gibco) and 20 ng/mL EGF (Gibco). After inducted for 9 days, the neural sphere morphology of SCAP were observed under the Olympus microscope (Tokyo, Japan), and were collected and analysed via the following RT‐PCR analysis or IF staining.

### 2.7 SCI in rat models

Total 32 Sprague–Dawley rats (12 weeks‐old, female; Experimental Animals Center of Research‐Science, Shanghai, China) were randomly divided into the sham group (8 rats) and SCI group (24 rats). All rats were anaesthetised with a mixture of xylazine (5 mg/kg) and ketamine (60 mg/kg), and the spinal cord at 10th thoracic vertebra was injured by cutting with a surgical blade, whereas the spinal cord of the Sham group was merely exposed. The paravertebral muscles and skin were sutured in layers. Bladder evacuation was applied daily post‐operation. All rats were maintained under postoperative care for 1 week and the BBB score was evaluated subsequently.

### 2.8 Basso, Beattie and Bresnahan (BBB) locomotor score evaluation

Hind limb neurobehavioral testing was performed using the Basso, Beattie, and Bresnahan (BBB) locomotor rating scale. The BBB open‐field locomotion testing was performed by two examiners blinded to group identity. The 21‐point BBB scale was used to assess hind limb locomotor recovery, including joint movements, stepping ability, coordination and trunk stability. A score of 21 indicated unimpaired locomotion as observed in uninjured rats. The animals were placed in an open field and the locomotion score was evaluated after 5 min of observation. The scores were analysed by repeated‐measures ANOVA with Tukey's multiple comparison tests at each time point.

### 2.9 Treatments in SCI

In the injection system of cell transplantation, 1 × 10^6^ SCAP were drawn into 30 μL saline and 30 ng peptides were drawn into 30 μL saline. Subsequently, the injection system was deposited into the focal point of the SCI site at a depth of 2 mm with a micromanipulator syringe (injection rate: 10 μL/min). Post‐injection, the rats were micturated and administered antibiotics for 1 week post‐operatively and were continued to be maintained under postoperative care for 3 weeks, as mentioned earlier, and the BBB score was evaluated weekly. After 3–4 weeks, animals in each group (*n* = 5) were anaesthetised and they underwent transcardiac perfusion with 0.9% NaCl and 4% paraformaldehyde. Subsequently, the spinal cord tissue surrounding the SCIs sites were harvested, fixed with 4% paraformaldehyde, dehydrated with gradient alcohol, embedded in paraffin and sectioned.

### 2.10 Haematoxylin–eosin staining

The sections were deparaffinized in xylene; the slides were transferred to 100%–50% alcohol, incubated in Weigert's iron haematoxylin solution for 5 min, acidized within seconds, and incubated in 0.1% eosin staining solution for 2 min. After sealing with neutral resin, the haematoxylin–eosin (HE)‐stained images were observed under the microscope.

### 2.11 Immunofluorescence staining

Neural spheres at induction Day 9 were collected, fixed with 4% paraformaldehyde, permeabilizated with 1% Triton X‐100 and blocked with 5% BSA. Then spheres were incubated with anti‐Nestin (Catalogue No. ab6142, Abcam) or anti‐βIII‐Tubulin (Catalogue No. ab18207, Abcam) overnight at 4°C, and incubated with the secondary antibody (Goat anti‐mouse/rabbit Alexa 488, Abcam). Nuclear staining was performed with 4′,6‐diamidino‐2‐phenylindole, dihydrochloride (DAPI, Invitrogen) and the images were observed under microscope and quantified.

For nerve sections, sections were deparaffinized and hydrated, permeabilizated with 1% Triton X‐100, and blocked with 5% BSA. Sections were incubated with primary antibodies overnight at 4°C, and incubated with the secondary antibody for 2 h at 4°C. After nuclear staining with DAPI, images were observed under microscope. The primary antibodies were anti‐βIII‐Tubulin (Catalogue No. ab18207, Abcam), anti‐NEF‐M (Catalogue No. ab253639, Abcam) and anti‐Mitochondria (Catalogue No. ab92824, Abcam).

### 2.12 mRNA Microarray Hybridization

Total RNA of SCAP were purified with the RNeasy mini kit (Qiagen Corporation, Hilden, Germany) and fragmented to cDNA using a GeneChip WT Terminal Labeling kit (Affymetrix, Santa Clara, CA, USA). Fragmented cDNA samples were subsequently hybridized to Human Transcriptome Array (Affymetrix). The Affymetrix Expression Console software (Version 1.3.1; Affymetrix) was used to extract raw data and provide RMA normalization. The arrays were scanned with the GeneChip® Scanner 3000 7G (Affymetrix) and the images were processed. Thereafter, the GeneSpring software (Version 12.5; Agilent Technologies) was employed to complete the basic analysis.

### 2.13 Chromatin immunoprecipitation‐Sequencing (ChIP‐Seq) Assays

According to the manufacturer's protocol (Merck Millipore, Darmstadt, Germany), SCAP were incubated in a 1% formaldehyde solution for 15 min. Each ChIP reaction employed 2 × 10^6^ SCAP. Anti‐histone H3K4me3 (Catalogue No. ab8580; Abcam) was applied for DNA precipitation. Normal rabbit IgG (Catalogue No. ab172730; Abcam) or normal mouse IgG (Catalogue No. sc‐2025; Santa Cruz Biotechnology, USA) were applied as negative control. For the ChIP‐Seq assays, the precipitated DNA samples were treated with the Paired‐End DNA Sample Prep kit (Illumina) and sequencing of each gene's promoter. After filtering, the clean data were aligned with the genome sequence of the target species using SOAPaligner/SOAP2 (Version: 2.21 T).

### 2.14 Bioinformatic Analysis

After the normalized data were compared and filtered, the differentially expressed genes/peaks were filtered using the random variance mode *t*‐test (differences between two classes) (*p* < 0.05) for subsequent analysis. Hierarchical clusters were performed using EPCLUST. Gene ontology (GO) analysis was applied to analyse the main function of the differentially expressed genes (DEGs) according to the two‐sided Fisher's exact test and χ^2f^ test (*p* < 0.05, false discovery rate [FDR] < 0.01). Enrichment of the GO category was calculated for significance of the function. Go‐map analysis was performed to identify the interaction net of DEGs. Furthermore, KEGG Pathway analysis was used to discover the significant pathway of the DEGs using Fisher's exact test and χ^2^ test (*p* < 0.05, FDR < 0.01).

### 2.15 Peptide Microarray Analysis

The KDM2B full‐length protein sequence was obtained from Uniprot (https://www.uniprot.org). According overlapping principle, KDM2B peptide microarray was synthesized, which consisted total 266 peptides and each peptide was 15 amino acids lengths. Peptide microarray was incubated in 5% BSA for 2 h at room temperature, then was incubated with biotin‐labelled EZH2 recombinant protein overnight at 4°C. After washing, peptide microarray was incubated with Horseradish peroxidase‐labelled chemiluminescent solution for 2 h at room temperature. By adding enhanced chemiluminescence chemiluminescence reagent, peptide microarray image was scanned by Chempchemi imaging system. The optical density values of colour rendering points were read and analysed by TotalLab image software. Bioinformatics analysis was used to compare the sequences between KDM2B protein and possible binding sites.

### 2.16 The CCK8 analysis

About 5 × 10^3^ SCAP were planted into the each well of 96 culture dish (Corning, NY, USA). The 10 μg/mL peptides were added into the culture medium, and cultured for 72 hours. According to the procedure of CyQUANT® Cell Quantitation assay kit (Catalogue No. C7026, Invitrogen), the number of SCAP was tested, and the fluorescence OD value was read at 485 nm, and analysed by using the Spectrometer (Tokyo, Japan).

### 2.17 Statistical Analysis

All statistical calculations were analysed by the Statistical Package for the Social Sciences 10 statistical software. Statistical significance was analysed by the one‐way ANOVA or Student's *t*‐test; *p* < 0.05 was considered statistically significant.

## 3 RESULTS

### 3.1 KDM2B enhanced the neurogenic indicators expression and neural spheres formation in SCAP

**FIGURE 1:**
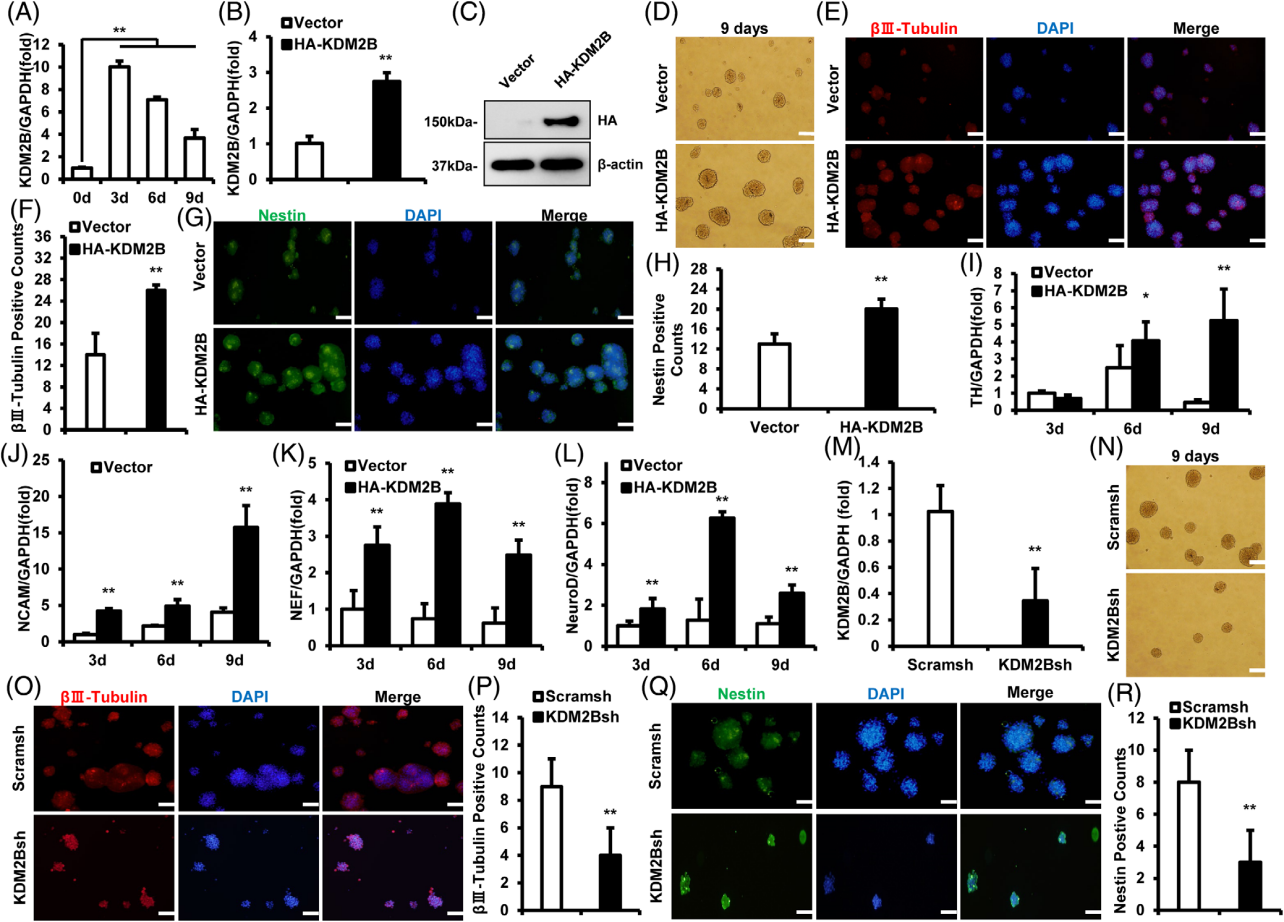
KDM2B promoted the neurogenic indicators expression of SCAP in vitro. (A) Real‐time RT‐PCR results of KDM2B level in SCAP at Days 0, 3, 6 and 9 after neurogenic induction. (B) Real‐time RT‐PCR and (C) western blot result of KDM2B overexpression level in SCAP. (D) Neural spheres formation of SCAP on Day 9 induction; Bar = 100 μm. (E) IF staining and (F) quantitative result of βIII‐Tubulin positive neural spheres; Bar = 100 μm. (G) IF staining and (H) quantitative results of Nestin positive neural spheres; Bar = 100 μm. (I–L) Real‐time RT‐PCR results of TH(I), NCAM(J), NEF(K) and NeuroD(L) expression in SCAP on Days 3, 6 and 9 after neurogenic induction. (M) Real‐time RT‐PCR result of KDM2B deletion in SCAP. (N) Neural spheres formation of SCAP on Day 9 induction; Bar = 100 μm. (O) IF staining and (P) quantitative result of βIII‐Tubulin positive neural spheres; Bar = 100 μm. (Q) IF staining and (R) quantitative results of Nestin positive neural spheres; Bar = 100 μm. glyceraldehyde‐3‐phosphate dehydrogenase (GAPDH) was taken as control; Error bar = SD, *n* = 3; Statistical significance was evaluated by Student's *t*‐test; *p < 0.05, **p < 0.01; Blank delineation explicated in this blot was cropped from different parts of different gels. IF, immunofluorescence; RT‐PCR, reverse transcriptase‐polymerase chain reaction; SCAP, stem cells from apical papilla.

Flow cytometry analysis revealed that the isolated SCAP is positive for CD90 (99.8%) and CD105 (99.9%) and negative for CD34 (1.66%) and CD45 (0.64%) (Figure S1). Real‐time RT‐PCR result showed that the level of KDM2B increased at Days 3, 6 and 9 after neurogenic induction (Figure 1A). Then, KDM2B was over‐expressed in SCAP by using its retrovirus (Figure 1B,C). After 9 days of induction, the morphological change showed that the formed neural spheres in KDM2B overexpressed group was significantly increased than the control group (Figure 1D). Immunofluorescence staining showed that the βIII‐Tubulin‐ and Nestin‐positive neural spheres in the KDM2B overexpression group were more and larger (Figure 1E–H). Next, real‐time RT‐PCR showed that KDM2B overexpression significantly enhanced the expression of neurogenic markers TH on induction Days 6 and 9, and NCAM, neurofilament (NEF) and NeuroD on Days 3, 6 and 9 (Figure 1I–L). Next, KDM2B was knocked‐down by shRNA lentivirus (Figure 1M). The morphological changes showed that KDM2B knockdown significantly decreased the number and size of formed βIII‐Tubulin‐ and Nestin‐positive neural spheres (Figure 1N–R). These suggested that KDM2B promoted the neurogenic indicators expression and neural spheres formation in SCAP.

### 3.2 KDM2B modified the H3K4me3 methylation on neurogenesis‐related genes

**FIGURE 2:**
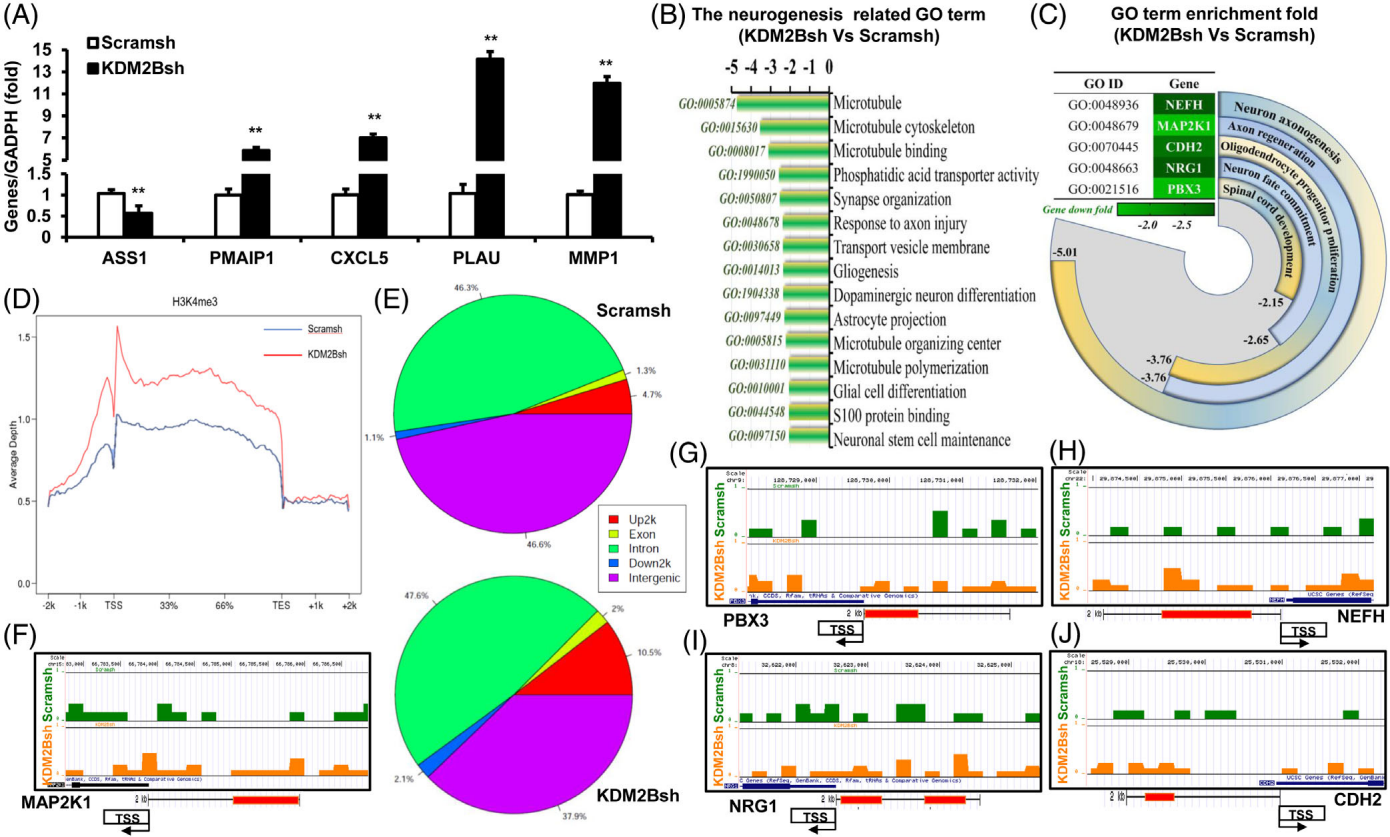
The mRNA microarray analysis and Chip‐Seq analysis in KDM2B‐deleted SCAP. (A) Real‐time RT‐PCR results of genes. (B) The associated GO terms of neurogenesis gene. (C) The GO regulatory function of key neurogenesis gene. (D) The ChIP‐Seq analysis in the KDM2B‐deleted SCAP. (E) The H3K4me3 modified rates of gene structure. (F–J) The H3K4me3 modified map in promoter region of MAP2K1(F), PBX3(G), NEFH(H), NRG1(I) and CDH2(J). GAPDH was taken as control; Error bar = SD, *n* = 3; Statistical significance was evaluated by Student's *t*‐test; **p < 0.01. CDH2, Cadherin‐2; MAP2K1, mitogen‐activated protein kinase‐1; NEFH, neurofilament heavy; NRG1, Neuregulin‐1; RT‐PCR, reverse transcriptase‐polymerase chain reaction; SCAP, stem cells from apical papilla.

The microarray analysis in the KDM2B‐deleted SCAP was performed, which reliability was verified by five randomly selected genes (Figure 2A). Total of 757 upregulated and 529 downregulated DEGs were revealed (Table S2). And the DEGs‐related GO terms showed that KDM2B knockdown mainly repressed the secretion and assembly of neurofilament protein S100 and tubulin protein, and the differentiation/repair function of neurogenic cells (Figure 2B). Among which, five neurogenesis‐related genes were focused on, including neurofilament heavy (NEFH), mitogen‐activated protein kinase‐1 (MAP2K1), Cadherin‐2 (CDH2), Neuregulin‐1 (NRG1) and PBX Homeobox‐3 (PBX3) (Figure 2C). Next, the ChIP‐Seq analysis was performed to reveal the differently H3K4me3 modification sites of neurogenesis‐related genes (Table S3; Figure 2D). There showed that the 2Kb upstream of promotor increased to 10.5% in the KDM2Bsh group compared with 4.7% in the control group, and the intergenic region decreased to 37.9% in the KDM2Bsh group compared with 46.6% in the control group (Figure 2E). The main differently H3K4me3 regions were respectively existed at the 1Kb‐2Kb upstream of MAP2K1 promotor (Figure 2F), the 0–2 kb upstream of PBX3 promotor (Figure 2G), the 0.5–1.5 kb upstream of NEFH promotor (Figure 2H), the 0–0.5 kb and 1.5–1.8 kb upstream of NRG1 promotor (Figure 2I), and the 1.5–1.8 kb upstream of CDH2 promotor (Figure 2J).

### 3.3 KDM2B improved the SCAP mediated recovery of motor ability at SCI early phase

**FIGURE 3:**
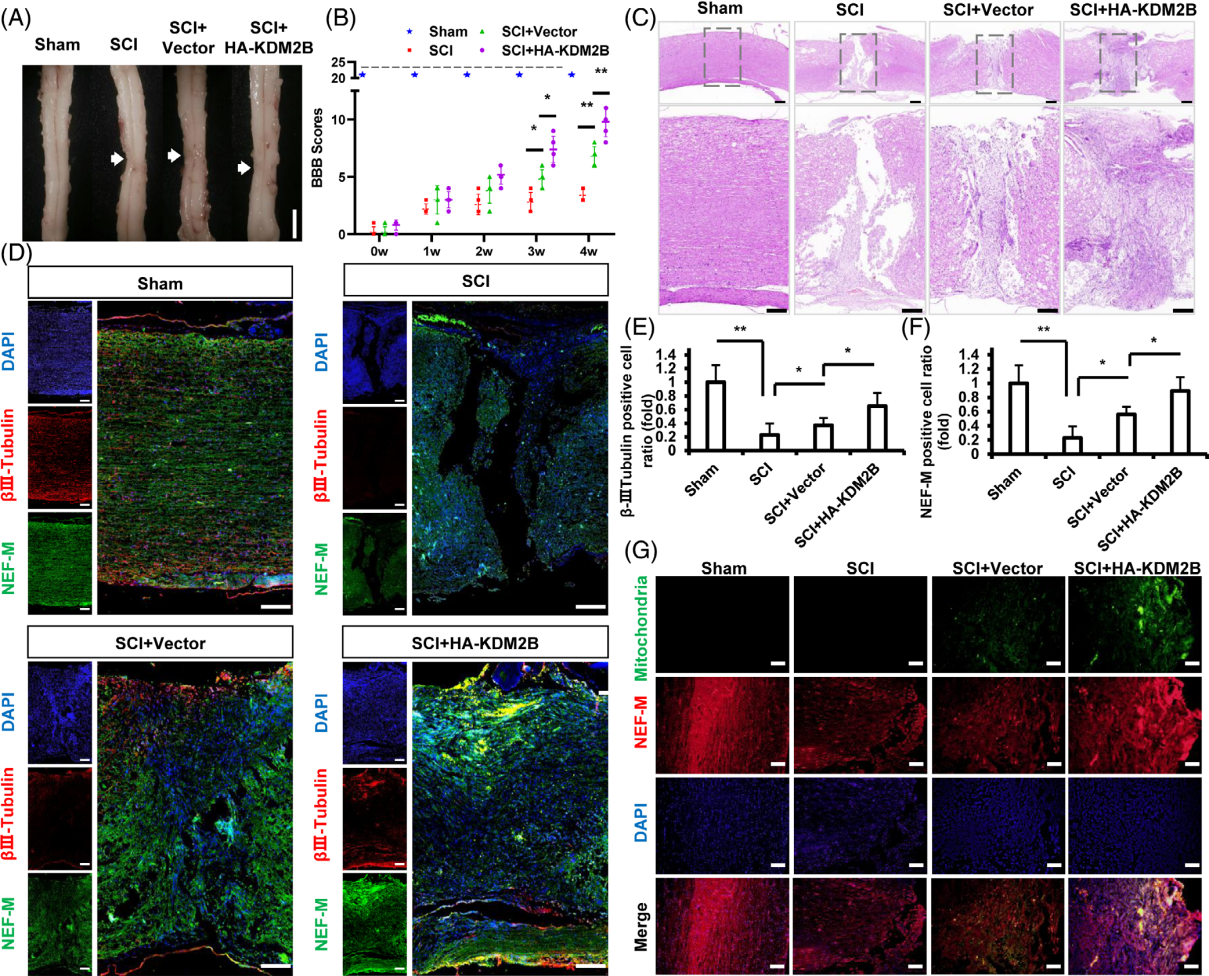
KDM2B promoted the SCAP mediated SCI repair in Sprague–Dawley rat models. (A) Gross observation of T10 spinal cord tissues; bar = 5 mm. (B) BBB score of the rat models. (C) HE staining of T10 spinal cord tissue; bar = 100 μm. (D) IF staining and (E, F) quantitative results of βIII‐Tubulin and NEF‐M; bar = 100 μm. (G) IF staining of human origin specificity marker mitochondria and NEF‐M in SCI tissue; bar = 100 μm. Error bar = SD, *n* = 3; Statistical significance was evaluated by ANOVA; *p < 0.05, **p < 0.01. BBB, Basso, Beattie, and Bresnahan; HE, haematoxylin–eosin; IF, immunofluorescence; NEF, neurofilament; SCAP, stem cells from apical papilla; SCI, spinal cord injury.

SCAP were transplanted into the injury site of SCI rats. After 4 weeks, gross observation showed that the SCI group existed the obvious tissue depression, uneven surface and colour variation in the injury site. The SCI + Vector group showed that the injury got partly improved, while the tissue depression and colour variation were still visible. Among which, the SCI + HA‐KDM2B group showed that the injury existed the better improvement in the tissue depression and colour variation, with more plump tissue appearing (Figure 3A). Further, the BBB scale results showed that at the start of week 0, the average BBB score of the three groups all fell within the range of grade 1–2, and showed no difference. In week 3, the average BBB score of SCI + HA‐KDM2B group elevated at grade 7.40 ± 1.14, which statistically higher than the SCI + Vector group (grade 4.80 ± 0.84) and the SCI group (grade 2.80 ± 0.84). In week 4, the average BBB score of SCI + HA‐KDM2B group continued to rise to grade 9.80 ± 1.30, the Vector group rose to grade 6.80 ± 0.84, while the SCI group still fluctuated at grade 3.40 ± 0.55. The average BBB scores of the three groups showed an increasing trend during the observation, among which the SCI + HA‐KDM2B group showed the best rise tendency than the other two groups (Figure 3B).

To assess the tissue structure, HE staining showed that the SCI group existed an obvious breakage trace and disintegration of nerve fibres, and the SCI + Vector group showed that the transplanted cells were aggregated and connected to the fractured nerve fibres at two flanks, of which existed the vacuoles. The SCI + HA‐KDM2B group showed that the transplanted cells were more evenly distributed, and the nerve fibres at both ends were denser and only existed a few small vacuoles (Figure 3C). Next, βIII‐Tubulin in injury location was detected to evaluate the elongation behaviour of axons during nerve repair, and the results showed that βIII‐Tubulin decreased in the SCI group compared with the Sham group. The Vector group was more positive than the SCI group, while the SCI + HA‐KDM2B group was more positive than the Vector group (Figure 3D,E). NEF‐M in SCI location was detected to evaluate the formation of nerve fibre, and the results showed that NEF‐M was decreased and structureless in the SCI group. The vector group was more organized than the SCI group, and the SCI + HA‐KDM2B group enhanced the expression of NEF‐M and showed more denser than that in the Vector and SCI group (Figure 3D,F). Further, to investigate the relationship between these neurofibrillary‐like structure and the transplanted SCAP, mitochondria, a specificity marker of human origin, was detected. And there showed that the Sham and SCI group were negative of fluorescence signal, while the mitochondria‐positived cells were observed in the SCI + Vector and SCI + HA‐KDM2B group adjacent to NEF‐M‐positived fibre‐like structure (Figure 3G). These suggested that KDM2B promoted the neurogenesis ability of SCAP and the SCAP mediated recovery at SCI early phase.

### 3.4 EZH2 was negatively regulated by KDM2B during neurogenic differentiation, and suppressed the neurogenic ability of SCAP

**FIGURE 4:**
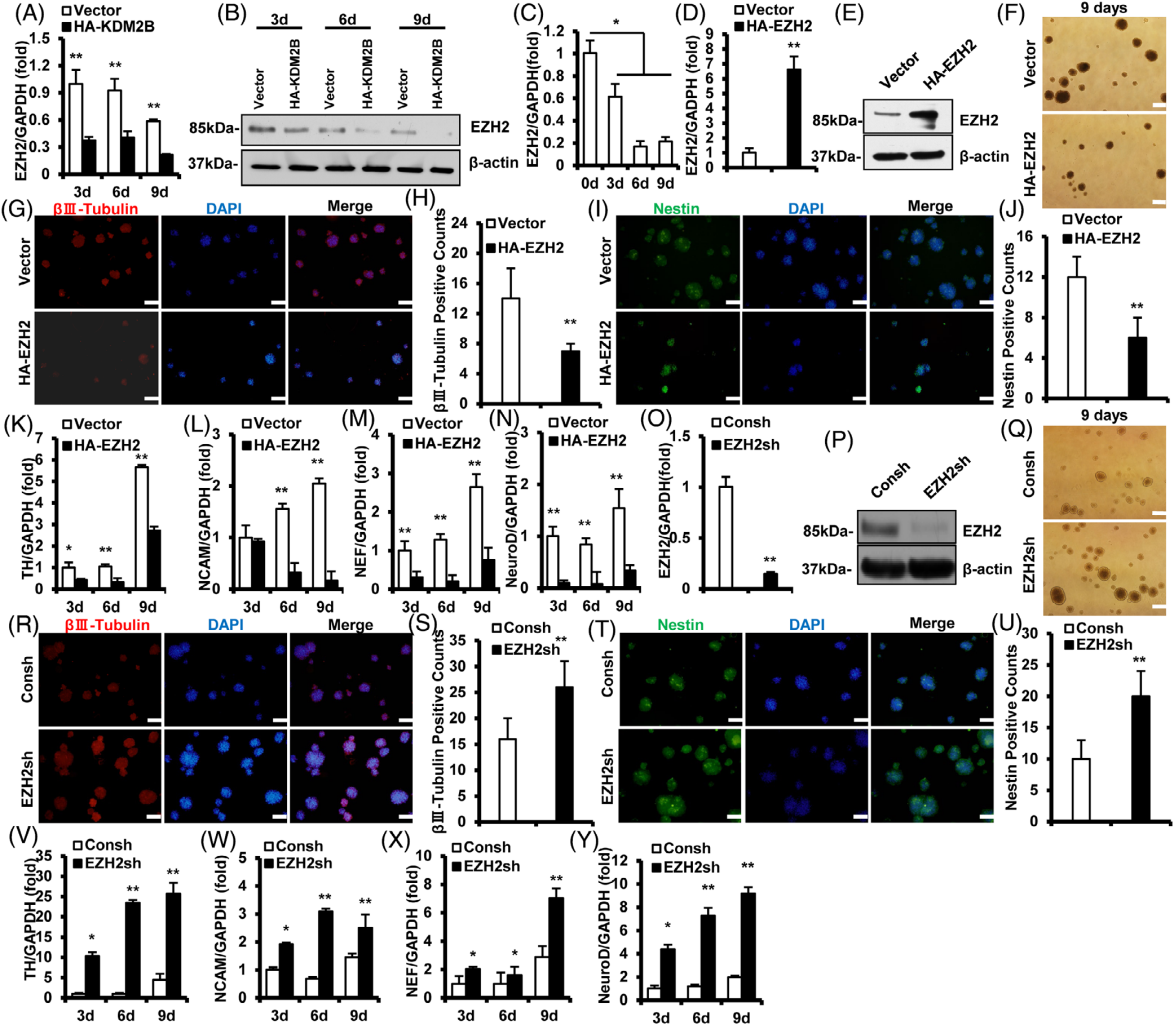
EZH2 was negatively regulated by KDM2B during neurogenic induction, and repressed the neurogenic indicators expression in SCAP. (A) Real‐time RT‐PCR and (B) western blot result of EZH2 expression in the KDM2B‐overexpressed SCAP. (C) EZH2 expression in SCAP at Days 0, 3, 6 and 9 during neurogenic induction. (D) Real‐time RT‐PCR and (E) western blot result of EZH2‐overexpression level in SCAP. (F) Neural spheres formation of EZH2‐overexpressed SCAP on the inducted 9 days. (G) IF staining and (H) quantitative result of βIII‐Tubulin positive neural spheres. (I) IF staining and (J) quantitative result of Nestin positive neural spheres. (K–N) The expression of TH(K), NCAM(L), NEF(M) and NeuroD(N) in EZH2‐overexpressed SCAP. (O) Real‐time RT‐PCR and (P) western blot result of EZH2 deletion in SCAP. (Q) Neural spheres formation of EZH2‐deleted SCAP on the inducted 9 days. (R) IF staining and (S) quantitative result of βIII‐Tubulin positive neural spheres. (T) IF staining and (U) quantitative result of Nestin positive neural spheres. (V–Y) The expression of TH(V), NCAM(W), NEF(X) and NeuroD(Y) in EZH2‐deleted SCAP. GAPDH was taken as control; Error bar = SD, *n* = 3; White bar = 100 μm; Statistical significance was evaluated by Student's t‐test; *p < 0.05, **p < 0.01; Blank delineation explicated this blot was cropped from different parts of different gels. EZH2, enhancer of zeste homologue 2; IF, immunofluorescence; NCAM, neural cell adhesion molecule; NEF, neurofilament; RT‐PCR, reverse transcriptase‐polymerase chain reaction; SCAP, stem cells from apical papilla; TH, tyrosine hydroxylase.

We supposed EZH2 might exists an interaction with KDM2B in SCAP. Thus, the expression of EZH2 was detected and results showed that EZH2 was significantly decreased in KDM2B‐overexpressed SCAP during neurogenic induction (Figure 4A,B). These suggested that KDM2B negatively regulated EZH2 during neurogenic differentiation. Real‐time RT‐PCR results showed that EZH2 was significantly decreased during neurogenic induction (Figure 4C). We overexpressed EZH2 in SCAP by its retroviruses (Figure 4D,E), and there showed that EZH2 overexpression significantly repressed the neural spheres formation ability of SCAP after 9 days induction (Figure 4F). Further immunofluorescence staining showed that EZH2 overexpression significantly repressed the formation of βIII‐Tubulin‐ and Nestin‐positived neural spheres (Figure 4G–J). Real‐time RT‐PCR results showed that EZH2 overexpression decreased the expression of NCAM on Days 6 and 9, and NEF, NeuroD and TH on Days 3, 6, and 9 after neurogenic induction (Figure 4K–N). Next, EZH2 was knocked‐down by shRNA lentivirus (Figure 4O,P). EZH2 knock‐down significantly increased the number and size of formed βIII‐Tubulin‐ and Nestin‐positived neural spheres (Figure 4Q–U). Also, EZH2 deletion enhanced the expression of NCAM, NEF, NeuroD and TH on Days 3, 6, and 9 after neurogenic induction (Figure 4V–Y). Together, these suggested that EZH2 repressed the neurogenic differentiation ability of SCAP, and negatively regulated by KDM2B.

### 3.5 The binding between EZH2 and KDM2B was decreased during neurogenic differentiation in SCAP

**FIGURE 5:**
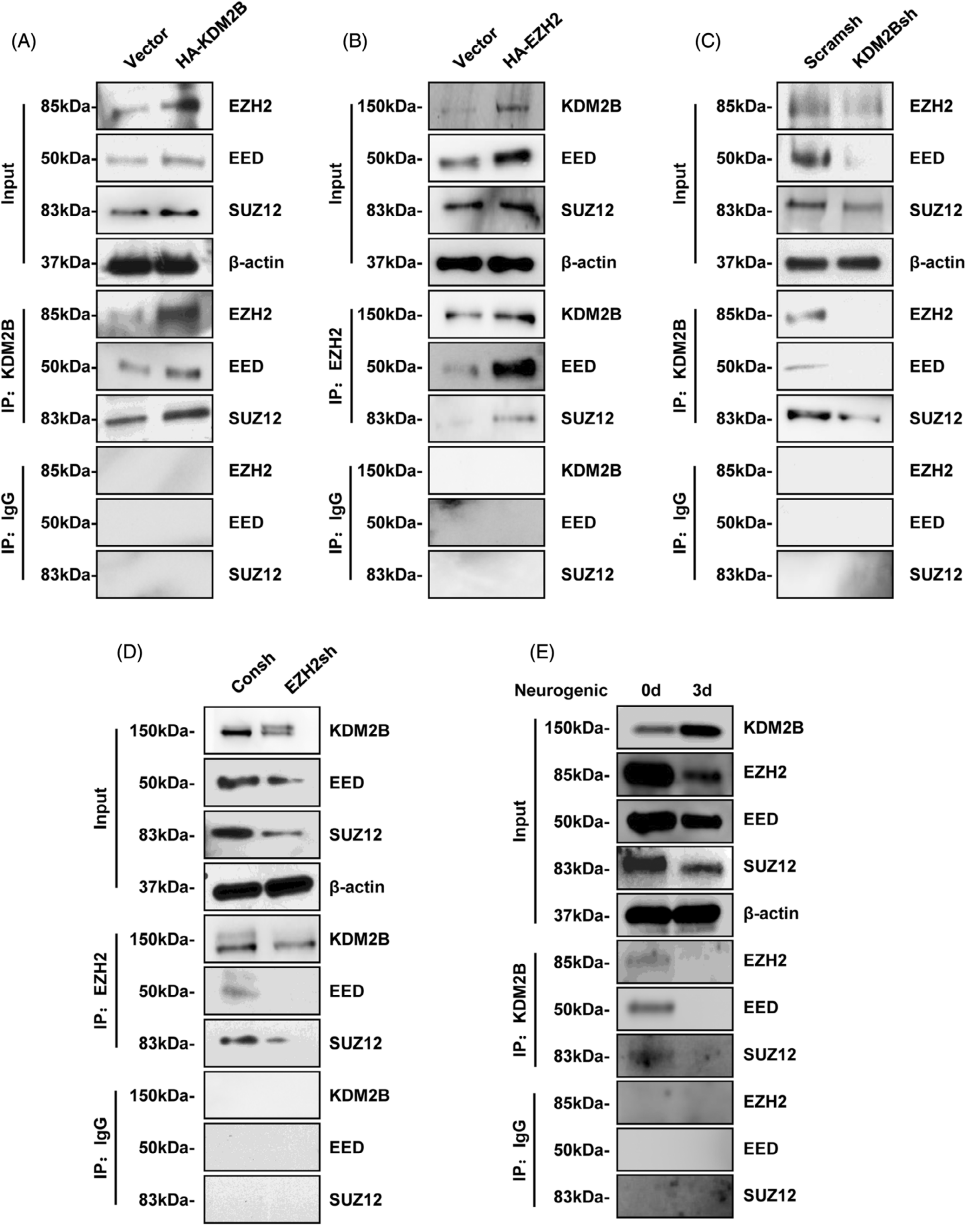
The PRC2 proteins lost binding to KDM2B during neurogenic induction in SCAP. (A) Co‐IP results of the binding between KDM2B and EZH2, EED, and SUZ12 in KDM2B‐overexpressed SCAP. (B) Co‐IP results of the binding between KDM2B and EZH2, EED and SUZ12 in EZH2‐overexpressed SCAP. (C) Co‐IP results of the binding between KDM2B and EZH2, EED and SUZ12 in KDM2B‐knockdown SCAP. (D) Co‐IP results of the binding between KDM2B and EZH2, EED and SUZ12 in EZH2‐knockdown SCAP. (E) Co‐IP results of the binding between KDM2B and EZH2, EED and SUZ12 at neurogenic induction Day 0 and Day 3 in SCAP. Blank delineation explicated in this blot is cropped from different parts of different gels. Co‐IP, co‐immunoprecipitation; EED, embryo development of the ectoderm protein; EZH2, enhancer of zeste homologue 2; IF, immunofluorescence; SUZ12, suppressor of zeste 12; SCAP, stem cells from apical papilla.

To detect the interaction of KDM2B and EZH2, Co‐IP analysis showed that the binding between KDM2B and EZH2, EED and SUZ12 were significantly increased in the HA‐KDM2B group compared with the control group (Figure 5A). And the binding between EZH2 and KDM2B, EED and SUZ12 was also significantly increased in the HA‐EZH2 group (Figure 5B). Simultaneously, the binding between KDM2B and EZH2, EED and SUZ12 significantly decrease in the KDM2Bsh group (Figure 5C), and EZH2 knocked‐down was also significantly decreased the binding between EZH2 and KDM2B, EED and SUZ12 (Figure 5D). To evaluate the binding interaction between EZH2 and KDM2B during neurogenic differentiation, Co‐IP analysis showed that the binding between KDM2B and EZH2, EED and SUZ12 was significantly decreased in neurogenic induction Day 3 compared with Day 0 (Figure 5E). Together, these suggested that KDM2B and EZH2 existed the protein interaction in SCAP, which was decreased during neurogenic process. And the decreased binding between KDM2B and EZH2 might be essential for neurogenic differentiation.

### 3.6 KDM2B‐peptides decreased the binding of EZH2 to KDM2B, and enhanced the neurogenic ability of SCAP

**FIGURE 6:**
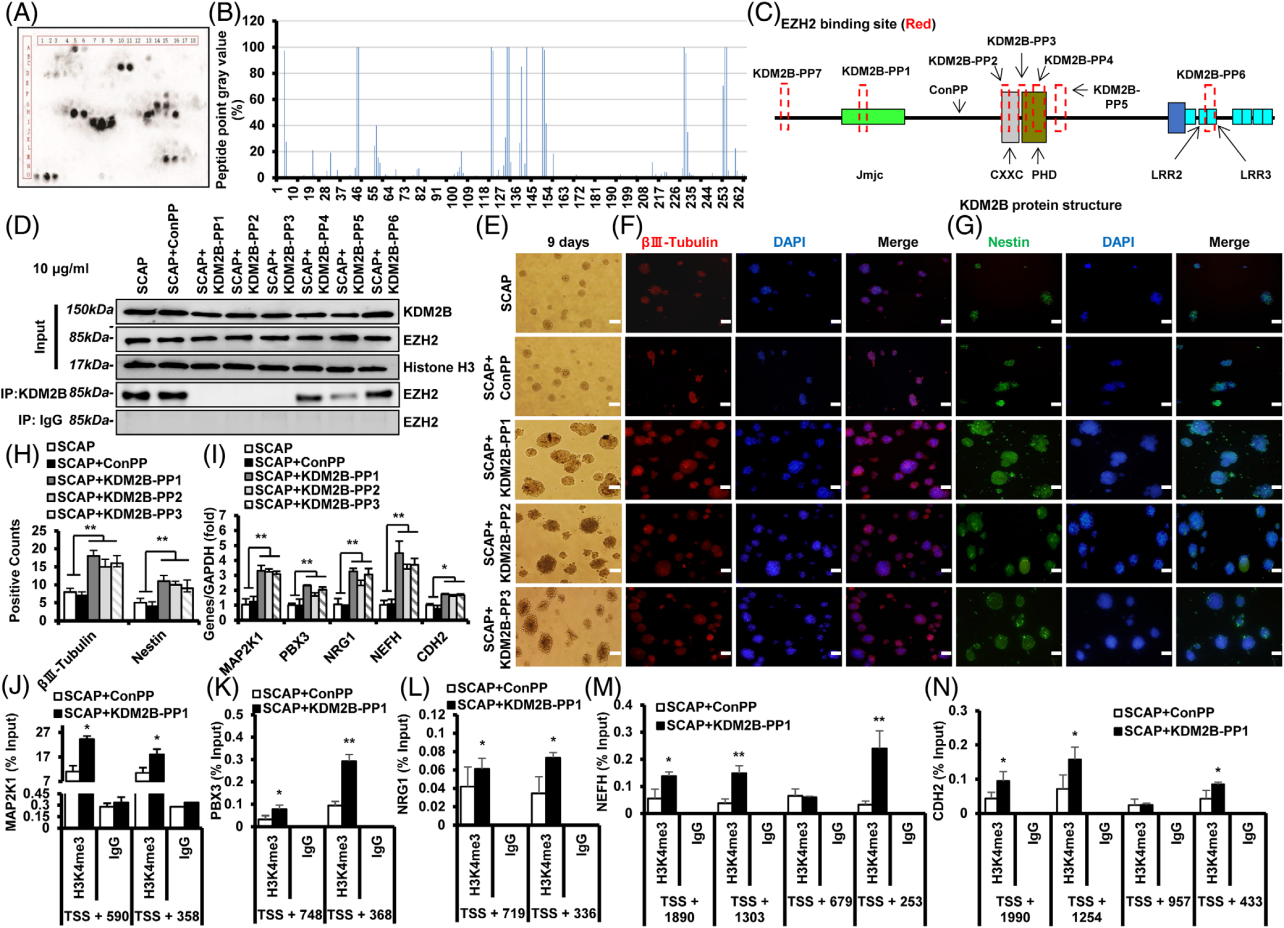
KDM2B‐peptides decreased the binding of EZH2 to KDM2B, and enhanced the neurogenic indicators expression of SCAP. (A) The immuno‐hybridization plot and (B) peptides grey value of peptide microarray assay. (C) Diagrammatic illustration of banding sites (red box) between EZH2 and KDM2B. (D) The binding level of KDM2B and EZH2 after treated with KDM2B‐peptides in SCAP. (E) Neural spheres formation. (F–H) βIII‐Tubulin positive neural spheres and Nestin positive neural spheres. (I) Expression of MAP2K1, PBX3, NRG1, NEFH and CDH2 in KDM2B‐peptides treated SCAP. (J–N) The H3K4me3 levels in promoter of MAP2K1(J), PBX3(K), NRG1(L), NEFH(M) and CDH2(N) in the KDM2B‐peptides treated SCAP. GAPDH was taken as control; Error bar = SD, *n* = 3; White bar = 100 μm; Statistical significance was evaluated by Student's *t*‐test; **p < 0.01. CDH2, Cadherin‐2; EZH2, enhancer of zeste homologue 2; MAP2K1, MAP2K1, mitogen‐activated protein kinase‐1; NEFH, neurofilament heavy; NRG1, Neuregulin‐1; SCAP, stem cells from apical papilla.

Peptide microarray analysis revealed total 13 sites as the significant protein binding sites of KDM2B to EZH2 (Figure 6A,B). These 13 sites were distributed across seven fragments of KDM2B protein (Figure 6C; Table S4). Based on the amino acids sequence of these seven binding fragments, we synthesized KDM2B‐peptides (KDM2B‐PP). Also, a negative binding region was chosen and synthesized as the control peptide (ConPP), with no specific domain nearby. The CCK8 analysis results showed that the number of SCAP have no difference in the SCAP group, the SCAP + ConPP group, and the SCAP + KDM2B‐PP1 group (Figure S2). Co‐IP results showed that the binding between KDM2B and EZH2 was completely reduced in the SCAP + KDM2B‐PP1, SCAP + KDM2B‐PP2 and SCAP + KDM2B‐PP3 group, and partly reduced in the SCAP + KDM2B‐PP5 group compared with the SCAP + ConPP group, while KDM2B‐PP4 and KDM2B‐PP6 showed not affect the binding between KDM2B and EZH2 (Figure 6D). These suggested that KDM2B‐PP1, KDM2B‐PP2 and KDM2B‐PP3 could block the binding site of EZH2 to KDM2B, and be one effective agent to intervene EZH2 function.

Further morphological changes showed that the formation of βIII‐Tubulin‐ and Nestin‐positive neural spheres were significantly increased in the SCAP + KDM2B‐PP1, SCAP + KDM2B‐PP2 and SCAP + KDM2B‐PP3 group (Figure 6E–H). Mechanistically, real‐time RT‐PCR result showed that MAP2K1, PBX3, NRG1, NEFH and CDH2 were all up‐regulated in the SCAP + KDM2B‐PP1, SCAP + KDM2B‐PP2 and SCAP + KDM2B‐PP3 group (Figure 6I). The ChIP assay results showed that the H3K4me3 level were enhanced at promotor region of MAP2K1 (TSS +590 and + 358; Figure 6J), PBX3 (TSS +748 and + 368; Figure 6K), NRG1 (TSS +719 and + 336; Figure 6L), NEFH (TSS +1890, + 1303, and+ 253; Figure 6M) and CDH2 (TSS +1990, + 1254 and + 433; Figure 6N). Western blot results showed that EZH2 decreased in the SCAP + KDM2B‐PP1 group at neurogenic Day 3 compared with the SCAP + ConPP group (Figure S3). These suggested that the usage of EZH2 function intervened agents, KDM2B‐peptides, could promoted the neurogenic differentiation ability of SCAP.

### 3.7 KDM2B‐PP1 improved the SCAP mediated recovery of motor ability at SCI early phase

**FIGURE 7:**
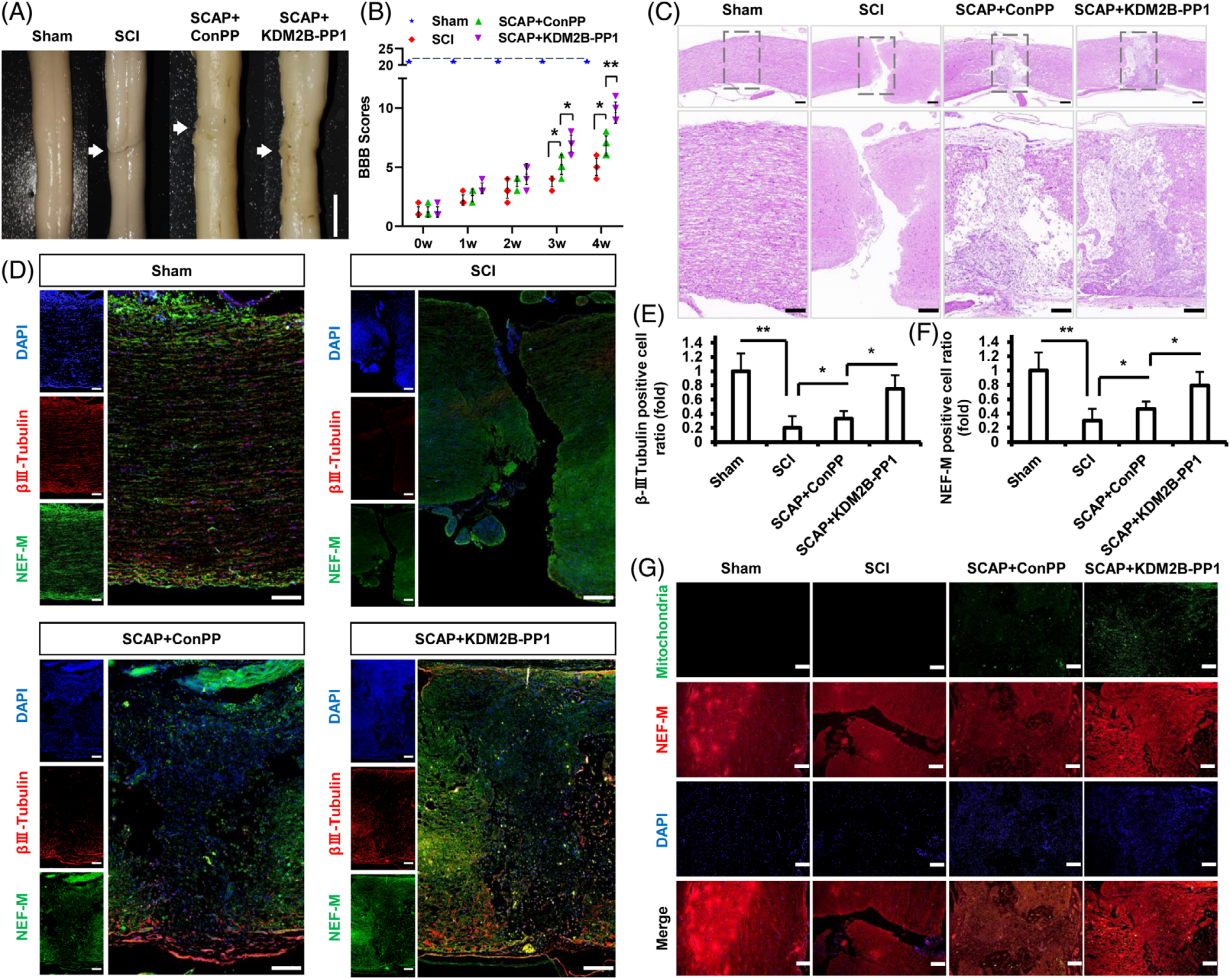
KDM2B‐peptides promoted the SCAP mediated SCI repair in SD rat models. (A) The gross observation of T10 spinal cord tissues; bar = 5 mm. (B) The BBB score of the rat models. (C) The HE staining of T10 spinal cord tissue; bar = 100 μm. (D) The IF staining and (E, F) quantitative results of βIII‐Tubulin and NEF‐M; bar = 100 μm. (G) The IF staining of Mitochondria and NEF‐M in SCI tissue; bar = 100 μm. Error bar = SD, *n* = 3; Statistical significance was evaluated by ANOVA; *p < 0.05, **p < 0.01. BBB, Basso, Beattie, and Bresnahan; HE, haematoxylin–eosin; IF, immunofluorescence; NEF, neurofilament; SCAP, stem cells from apical papilla.

Subsequently, the effect of KDM2B‐PP1 on nerve injury repair at early phase was investigated, and the gross observation showed that the SCI group existed the obvious tissue depression, uneven surface, and colour variation in the injury site. The ConPP‐pretreated‐SCAP transplantation (SCAP + ConPP) group showed that the injury got partly improved, while the tissue depression and colour variation were still visible in the injury site. Among which, The KDM2B‐PP1‐pretreated‐SCAP transplantation (SCAP + KDM2B‐PP1) group showed that the injury existed the better improvement in tissue depression and colour variation (Figure 7A). Next, the BBB scale showed that in week 3, the average BBB score of SCAP + KDM2B‐PP1 group elevated at grade 7.00 ± 0.70, which statistically higher than the SCAP + ConPP group (grade 5.20 ± 0.83) and the SCI group (grade 3.80 ± 0.44). In week 4, the average BBB score of SCAP + KDM2B‐PP1 group continued to rise to grade 9.60 ± 0.89, the Vector group rose to grade 6.80 ± 0.83, while the SCI group still fluctuated at grade 5.00 ± 0.70 (Figure 7B). The average BBB scores of three groups showed an increasing trend during observation, among which the SCAP + KDM2B‐PP1 group showed the best rise than the other two groups.

Further HE staining showed that the SCI group existed an obvious breakage trace and disintegration of nerve fibres, and the SCAP + ConPP group showed that the transplanted cells were aggregated and connected to fractured nerve fibres at two flanks, of which existed vacuoles. Further, the SCAP + KDM2B‐PP1 group showed that the transplanted cells were denser and only existed a few vacuoles (Figure 7C). IF staining showed that the expression of βIII‐Tubulin and NEF‐M were decreased in the SCI group compared with the sham group. The SCAP + ConPP group was more positive than the SCI group, and the SCAP + KDM2B‐PP1 group was more positive than the SCAP + ConPP group (Figure 7D–F). More, results at the injured nerve location of human origin marker mitochondria showed that the sham and SCI group were negative of fluorescence signal, while the Mitochondria‐positive cells were observed adjacent to the NEF‐M positive fibre‐like structure in the SCAP + ConPP and SCAP + KDM2B‐PP1 group (Figure 7G). Together, these suggested that the treatment of KDM2B‐peptides significantly improved the neurogenesis ability of SCAP and the SCAP mediated recovery at SCI early phase.

## 4 DISCUSSION

In this study, we provide evidence for KDM2B to neurogenic differentiation and nerve injury repair by using dental MSCs. Studies report that neurogenic cells have the characteristics of forming neural spheres, and significantly expressed the neurogenic marker Nestin and βIII‐Tubulin.^19^, ^34^, ^36^ We found that KDM2B was increased during neurogenic. KDM2B promoted the formation of βIII‐Tubulin‐ and Nestin‐positived neural spheres, and promoted the expression of NeuroD, NCAM, NEF and TH in SCAP. NeuroD is the key transcription factor for neurogenic cells to form neural precursor cells (NPCs).^34^ NPCs are high expressed NCAM, and could further differentiate into glial cells (high expressed NEF) and granule neurons (high expressed TH).^36^, ^37^ Study reports that KDM2B up‐regulates neuron specific gene in haematopoietic stem cells.^25^ KDM2B could promote the NSCs mediated neurite elongation.^26^ And KDM2B promotes the formation and migration of cortical projection neurons.^27^ These suggested that KDM2B acted as a promotor to neurogenic differentiation ability of dental MSCs.

Mechanistically, our found that KDM2B knockdown mainly repressed the secretion and assembly of neurofilament protein S100 and tubulin protein, and the repair of neurogenic linages. And five neurogenesis‐related genes were revealed including MAP2K1, PBX3, NRG1, NEFH and CDH2. Study reports that MAP2K1 is involved in axon formation.^38^, ^39^ PBX3 shows highly expressed during central nervous development.^40^ NRG1 promotes the repair of spinal cord and sciatic nerve axons in mice.^41^ NEFM and NEFH show significantly increased during neurogenic differentiation in hADSCs and hBMSCs.^42^ Conditional deletion of CDH2 inhibits the migration ability of granular neurons.^43^ These results were consistent with our observation. Further ChIP‐seq revealed that KDM2B mainly modified the H3K4me3 in 1Kb‐2Kb upstream region at MAP2K1 promotor, the 0–2 kb upstream region at PBX3 promotor, the 0.5–1.5 kb upstream region at NEFH promotor, the 0–0.5 kb and 1.5–1.8 kb upstream region at NRG1 promotor, and the 1.5–1.kb upstream region at CDH2 promotor. These suggested that KDM2B regulated the expression of MAP2K1, PBX3, NRG1, NEFH and CDH2 via H3K4me3 modification, and the target sites of KDM2B modified H3K4me3 mainly existed at the upstream 0–2 kb region of promoter in MAP2K1, PBX3, NRG1, NEFH and CDH2.

Further, we hypothesized the role of KDM2B to nerve injury repair, and found that nerve injury existed better improvement by transplanting the KDM2B‐overexpressed SCAP. The BBB scale is used to take as one recovery index to assess the motor ability after SCI.^44^, ^45^, ^46^ We found that the motor ability had a better improvement by transplanting the KDM2B‐overexpressed SCAP at early stage after SCI. To assess the repair of injured nerve, NEF‐M and βIII‐Tubulin were detected. We found that KDM2B significantly increased βIII‐Tubulin and NEF‐M expression in injured nerve, and promoted the transplanted SCAP to form an organized and denser fibre‐like structure. Study has reported that the microtubules along nerve fibre are the basis of intracytoplasmic transport during injury repair.^47^, ^48^ As the main components of nerve microtubule, βIII‐Tubulin mainly evaluate the elongation behaviour of axons, and NEF‐M evaluate the formation of broken nerve fibres.^49^ We further found that the NEF‐M positive fibres existed more colocalization with the transplanted SCAP (Mitochondria positive), which indicated that KDM2B improved the SCAP mediated elongation and microtubule repair of injured nerve fibres. These indicated that KDM2B promoted the SCAP mediated recovery at SCI early phase.

Study reports that KDM2B could regulate EZH2 expression in mouse embryonic fibroblasts.^31^ We hypothesized that the interaction between EZH2 and KDM2B during neurogenic differentiation, and found that KDM2B negatively regulated EZH2 during neurogenic. More, EZH2 was decreased during neurogenic differentiation, and inhibited the βIII‐Tubulin‐ and Nestin‐positived neural spheres formation and NeuroD, NCAM, NEF and TH expression in SCAP. Study reports that EZH2 reduces during neurogenic differentiation of mNSCs.^50^ EZH2 inhibits the differentiation ability of ventral midbrain‐derived NSCs into dopaminergic neurons.^51^ And EZH2 represses the repair of SCI rat by increasing the apoptosis of neuronal cells.^52^ Our results were consistent with the previous studies, suggesting that EZH2 acted as a negative regulator to neurogenic differentiation of MSCs, and this repressed function was negatively regulated by KDM2B. EZH2 is the core subunit protein of PRC2 complex, and acts as a co‐binding protein of KDM2B in HEK293T cells.^32^, ^33^ We found that KDM2B and EZH2 existed the protein binding interaction in SCAP, and decreased during neurogenic differentiation. These suggested that the decreased binding of EZH2 to KDM2B might be essential for neurogenic differentiation, and might provide the intervention target for the improvement of neurogenic differentiation.

To interfere EZH2 function based on the negative regulatory and interaction of KDM2B, we revealed 7 key protein binding sequences of KDM2B to EZH2, and synthesized as KDM2B‐peptides. And KDM2B‐PP1, KDM2B‐PP2 and KDM2B‐PP3 could all effectively decrease the binding of EZH2 to KDM2B. Previous studies have reported that the mutation in KDM2B exon 7–8 (located in the JmjC domain) caused half of the homozygous mice exhibited neural tube closure disorder.^25^, ^28^ We found that KDM2B‐PP1 located at the middle of KDM2B Jmjc domain. KDM2B‐PP2 located at the start of KDM2B CXXC domain. KDM2B‐PP3 located at the interface of CXXC and PHD domain. These indicated that the binding of KDM2B to EZH2 mainly existed in the JmjC and CXXC domain of KDM2B, and KDM2B‐peptide could be used as an effective agent to intervene EZH2 function. More, the usage of KDM2B‐PP1, KDM2B‐PP2 and KDM2B‐PP3 could promote the βIII‐Tubulin‐ and Nestin‐positive neural spheres formation in SCAP, and could up‐regulate the expression of the neurogenesis‐related genes MAP2K1, PBX3, NRG1, NEFH and CDH2. Following ChIP assay showed that KDM2B‐PP1 effectively enhance the H3K4me3 level at the promoter region of MAP2K1, PBX3, NRG1, NEFH and CDH2. Together, these suggested that the usage of EZH2 function intervened agents, KDM2B‐peptides, could promoted the neurogenic differentiation ability of SCAP.

To investigate the effect of KDM2B‐peptides to nerve injury repair, the KDM2B‐PP1 pretreated SCAP was transplanted into the SCI sites, and the nerve injury and motor ability of SCI rats both had a better improvement by transplanting the KDM2B‐PP1 pretreated SCAP. More, we found that KDM2B‐PP1 promoted the transplanted SCAP to form an organized and denser fibre‐like structure in injury site, with significantly increased expression of βIII‐Tubulin and NEF‐M. NEF‐M positive fibres and Mitochondria positive cells existed more colocalization by transplanting the KDM2B‐PP1 pretreated SCAP in SCI tissue. These indicated that KDM2B‐peptides could improve SCAP mediated microstructural repair and relink of injured nerve fibres. Although further research still needed to focus on the better usage concentration and treatment time, these findings suggested that the treatment of KDM2B‐peptides could promoted SCAP mediated repair of injured nerve at early stage, and could be taken as an agent for improving the neurogenesis ability of MSCs and the repair of nerve injury.

## AUTHOR CONTRIBUTIONS

Yangyang Cao, Yantong Wang: Collection and assembly of data, data analysis and interpretation, manuscript writing; Dengsheng Xia: Data analysis and interpretation, manuscript writing; Yangyang Cao and Zhipeng Fan: Conception and design, final approval of manuscript, financial support. All authors have read and approved the final version of the manuscript.

## CONFLICT OF INTEREST STATEMENT

The authors declare that they have no conflict of interest.

## Supporting information


Figure S1.

Figure S2.

Figure S3.

Table S1.

Table S2.

Table S3.

Table S4.

File S1.

## Data Availability

The data that supports the findings of this study are available in the supplementary material of this article.
