# Supplementary figures and images for: KDM2B and its peptides promote the stem cells from apical papilla mediated nerve injury repair in rats by intervening EZH2 function
Source: Cell Prolif. 2024 Oct 2;58(2):e13756. doi: 10.1111/cpr.13756 (PMC11839186; doi:10.1111/cpr.13756)

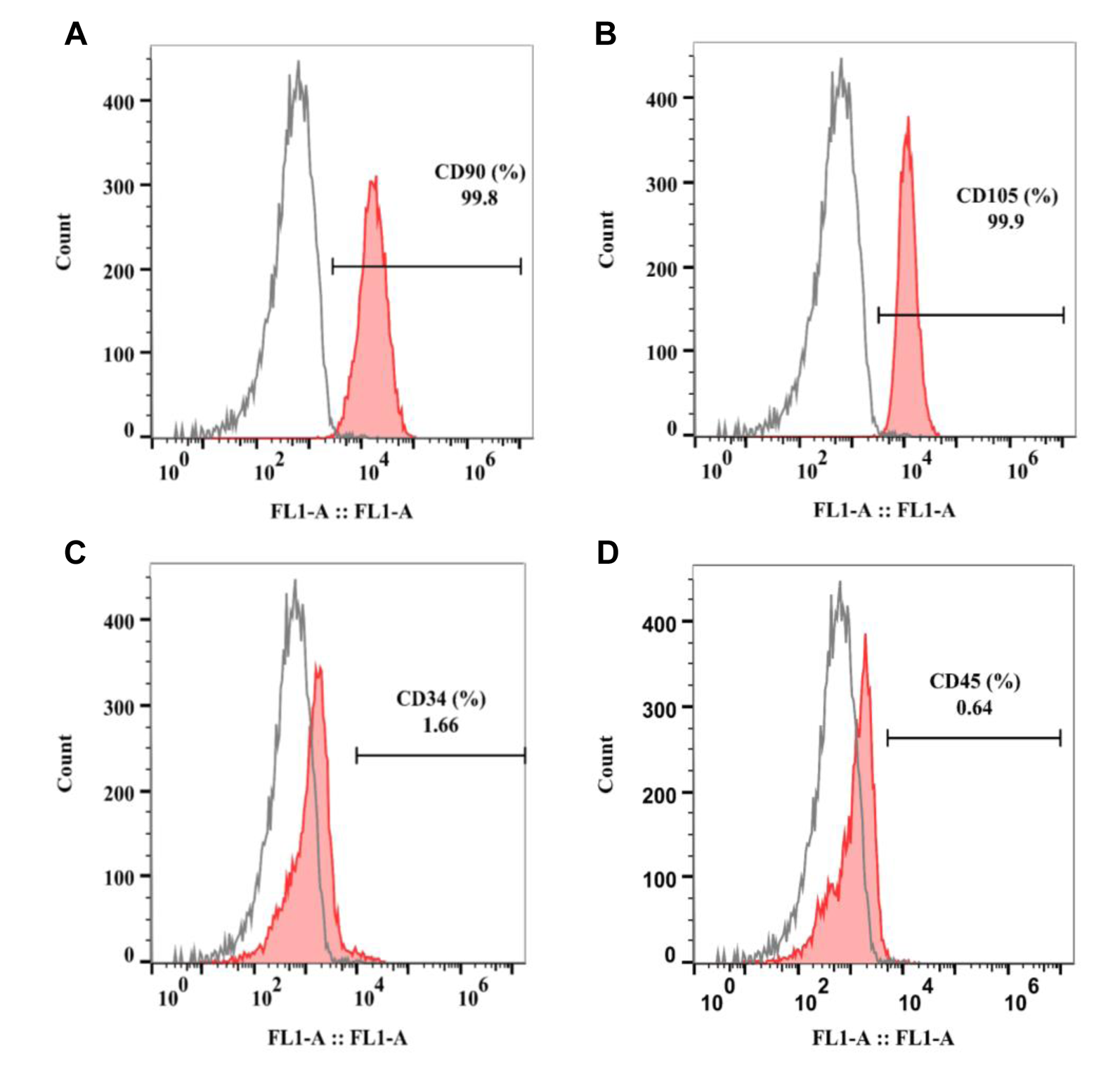

Supplement: Supplementary file 1 — Figure S1. [file CPR-58-e13756-s003.tif]

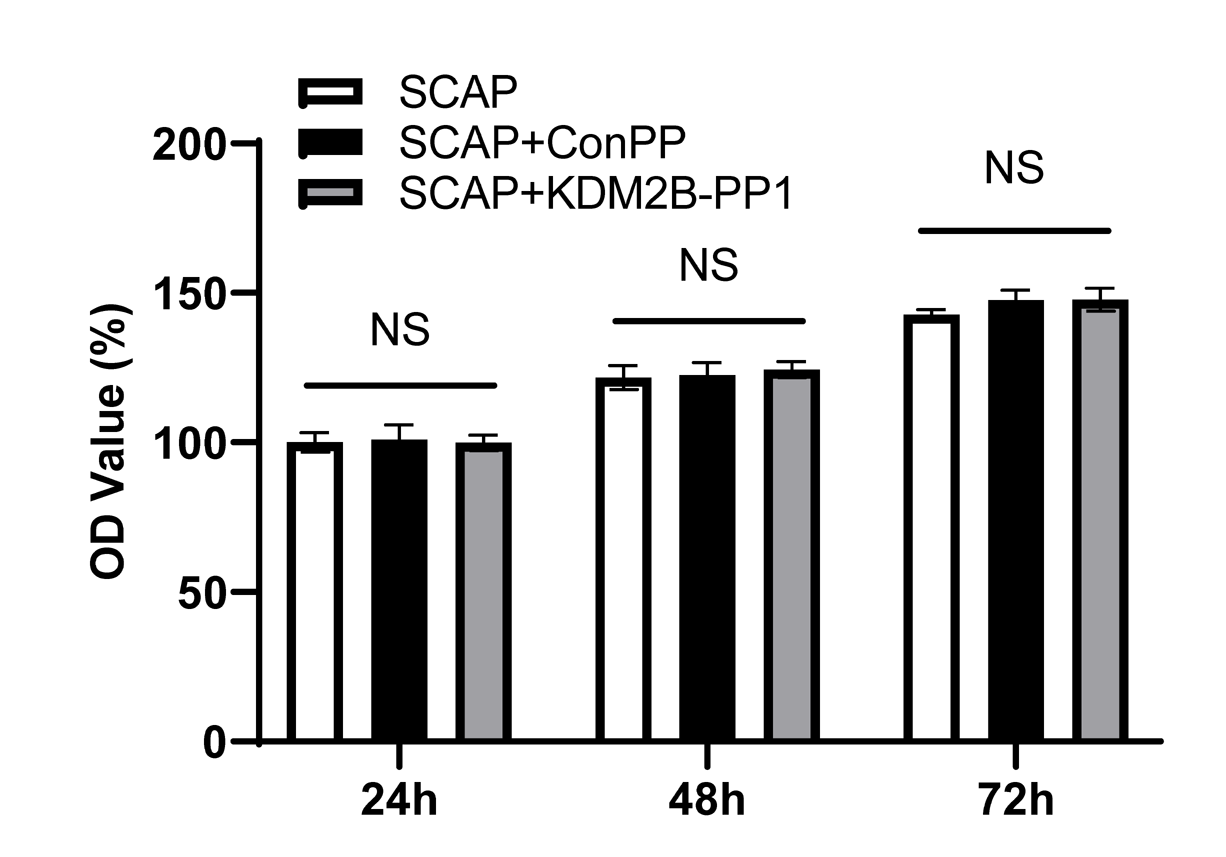

Supplement: Supplementary file 2 — Figure S2. [file CPR-58-e13756-s008.tiff]

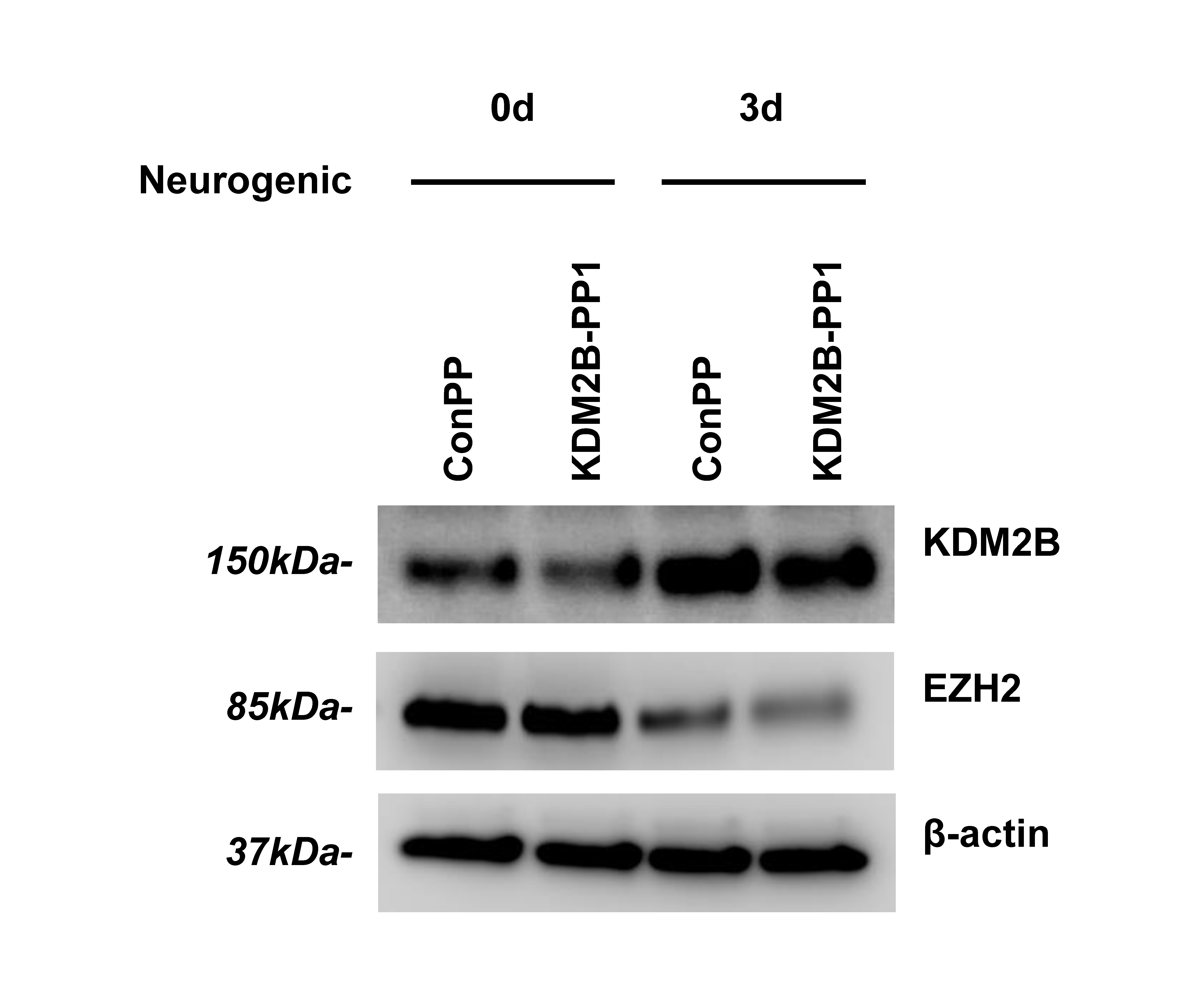

Supplement: Supplementary file 3 — Figure S3. [file CPR-58-e13756-s002.tif]
