# Supplementary material for: KDM2B and its peptides promote the stem cells from apical papilla mediated nerve injury repair in rats by intervening EZH2 function
Source: Cell Prolif. 2024 Oct 2;58(2):e13756. doi: 10.1111/cpr.13756 (PMC11839186; doi:10.1111/cpr.13756)
Supplement: Supplementary file 4 — Table S1. [file CPR-58-e13756-s001.pdf]

Table S1. The specific primers for tested genes.

Genes	Target Sequences
β Ⅲ-Tubulin-Forward	5’ GGCCAAGGGTCACTACACG-3’
β Ⅲ-Tubulin-Reverse	5’ GCAGTCGCAGTTTTCACACTC-3’
ASS1-Forward	5’ TCCGTGGTTCTGGCCTACA-3’
ASS1-Reverse	5’ GGCTTCCTCGAAGTCTTCCTT-3’
BMP4-Forward	5’ ATGATTCCTGGTAACCGAATGC-3’
BMP4-Reverse	5’ CCCCGTCTCAGGTATCAAACT-3’
CDH2-Forward	5’ TGCGGTACAGTGTAACTGGG-3’
CDH2-Reverse	5’ GAAACCGGGCTATCTGCTCG-3’
COL1A1-Forward	5’ GAGGGCCAAGACGAAGACATC-3’
COL1A1-Reverse	5’ CAGATCACGTCATCGCACAAC-3’
CXCL5-Forward	5’ AGCTGCGTTGCGTTTGTTTAC-3’
CXCL5-Reverse	5’ TGGCGAACACTTGCAGATTAC-3’
FGFR2-Forward	5’ GGAAAGTGTGGTCCCATCTGA-3’
FGFR2-Reverse	5’ TCCAGGTGGTACGTGTGATTG-3’
GAPDH-Forward	5’ GATCATCAGCAATGCCTCCT-3’
GAPDH-Reverse	5’ ACCTGGTGCTCAGTGTAGCC-3’
KDM2B-Forward	5’ GTTAGTGGTAGTGGTGTTTTGG-3’
KDM2B-Reverse	5’ AGCAGATGTGGTGTGTGGTC-3’
MAP2K1-Forward	5’ GGGCTTCTATGGTGCGTTCTA-3’
MAP2K1-Reverse	5’ CCCACGGGAGTTGACTAGGAT-3’
MMP1-Forward	5’ AAAATTACACGCCAGATTTGCC-3’
MMP1-Reverse	5’ GGTGTGACATTACTCCAGAGTTG-3’
NCAM-Forward	5’ CGGGACCTGGAGGACTTCTACCCG-3’
NCAM-Reverse	5’ ACCATGTGCCCATCCAGAGTC-3’
NEF-Forward	5’ CGAAGTCAATGGTTTCCTCCACTTCG-3’
NEF-Reverse	5’ CCGCTCCTTCCCGTCCTACTAC-3’
NEFH-Forward	5’ GCAGTCCGAGGAGTGGTTC-3’
NEFH-Reverse	5’ CGCATAGCGTCTGTGTTCA-3’
Nestin-Forward	5’ CTGCTACCCTTGAGACACCTG-3’
Nestin-Reverse	5’ GGGCTCTGATCTCTGCATCTAC-3’
NeuroD-Forward	5’ CGACTGACCCCTACTCCTACCAGTCG-3’
NeuroD-Reverse	5’ TGGAAGACATGGGAGCTGTCC-3’
NRG1-Forward	5’ GTGAAAGGTATGTGTCAGCCA-3’
NRG1-Reverse	5’ GCGTGTGGAAATCTACAGGTG-3’
PBX3-Forward	5’ GACGGAAAAGGCGTAACTTCA-3’
PBX3-Reverse	5’ GGTTGCTGAGGTGTGAGTAAAAA-3’
PLAU-Forward	5’ CTGTCACCTACGTGTGTGGAG-3’
PLAU-Reverse	5’ TGAGCGACCCAGGTAGACG-3’
PMAIP1-Forward	5’ ACCAAGCCGGATTTGCGATT-3’
PMAIP1-Reverse	5’ ACTTGCACTTGTTCCTCGTGG-3’
TH-Forward	5’ CCGAGCTGTGAAGGTGTTTGA-3’
TH-Reverse	5’ CGGGCCGGGTCTCTAGAT-3’

WNT5A-Forward	5’ GCCAGTATCAATTCCGACATCG-3’
WNT5A-Reverse	5’ TCACCGCGTATGTGAAGGC-3’
ChIP-CDH2-site1-Forward	5’ TCCAAATTACCAGGAATGGCCT-3’
ChIP-CDH2-site1-Reverse	5’ TGTAAGACAATCCAGCCAGGG-3’
ChIP-CDH2-site2-Forward	5’ CTTCTGTAGCATGGCAGCGG-3’
ChIP-CDH2-site2-Reverse	5’ GTTGATGTACAATTCACAGGCCAT-3’
ChIP-CDH2-site3-Forward	5’ GTACAACAGCATGGAGGCAC-3’
ChIP-CDH2-site3-Reverse	5’ CCAGAGCTTGCCTCTGGGAA-3’
ChIP-CDH2-site4-Forward	5’ GGTGGTGGCTCTAATGGGAA-3’
ChIP-CDH2-site4-Reverse	5’ CAACCATGTACGGTGTGCAG-3’
ChIP-MAP2K1-site1-Forward	5’ CTGCTCTCAAAAACTGTCGCA-3’
ChIP-MAP2K1-site1-Reverse	5’ GCCCAACAAACTGACGCATA-3’
ChIP-MAP2K1-site2-Forward	5’ ATTTTCGTCAACACGGCTCG-3’
ChIP-MAP2K1-site2-Reverse	5’ GCAGATGACGCGGAAATACG-3’
ChIP-NEFH-site1-Forward	5’ TGGCCTGGTATGTGGTTTCT-3’
ChIP-NEFH-site1-Reverse	5’ GTCTCGATCTCCTGACCTCG-3’
ChIP-NEFH-site2-Forward	5’ ATCGCTTAAACTCGGGAGGT-3’
ChIP-NEFH-site2-Reverse	5’ ATCCTGGGCTCAAGTGATCC-3’
ChIP-NEFH-site3-Forward	5’ GCCAAGATTGTGTCACTGCA-3’
ChIP-NEFH-site3-Reverse	5’ CCTCCCAGGTTCAAGCAATT-3’
ChIP-NEFH-site4-Forward	5’ ATCAAACATGCCCAAGGACTCT-3’
ChIP-NEFH-site4-Reverse	5’ GAGGTCCGTCTGATTTCCCT-3’
ChIP-NRG1-site1-Forward	5’ CCCGACCACAACACAAGAGT-3’
ChIP-NRG1-site1-Reverse	5’ TAAAGACCAGGGTAACGGGC-3’
ChIP-NRG1-site2-Forward	5’ TTCTGAGTCGTGCTCCGAAC-3’
ChIP-NRG1-site2-Reverse	5’ ACAGGAAGGGCTAAGCGAAC-3’
ChIP-PBX3-site1-Forward	5’ GGCAGTTACACTGTGAGGGT-3’
ChIP-PBX3-site1-Reverse	5’ TGGGAGCAATTCCTACAGGC-3’
ChIP-PBX3-site2-Forward	5’ TCCTAGGGAGGCCGAAATGA-3’
ChIP-PBX3-site2-Reverse	5’ AGACATGCCTGCACAGTCTC-3’
