# Supplementary material for: KDM2B and its peptides promote the stem cells from apical papilla mediated nerve injury repair in rats by intervening EZH2 function
Source: Cell Prolif. 2024 Oct 2;58(2):e13756. doi: 10.1111/cpr.13756 (PMC11839186; doi:10.1111/cpr.13756)
Supplement: Supplementary file 5 — Table S2. [file CPR-58-e13756-s005.pdf]

Table S2. Differentially expressed genes in SCAPs-KDM2Bsh compared with the Scramsh group.

Genes	Fold	Regulation	p-Value
EPGN	-35.37	down	0.0026
NPTX1	-29.44	down	0.0007
SHISA2	-15.77	down	0.0047
IL13RA2	-13.63	down	0.0031
TFPI2	-13.30	down	0.0029
HSD17B2	-12.27	down	0.0033
APOBEC3A	-11.90	down	0.0030
ADGRG1	-11.78	down	0.0025
APOBEC3B	-11.17	down	0.0018
CPA4	-10.90	down	0.0034
ANGPTL4	-10.58	down	0.0047
SLC16A6	-10.00	down	0.0050
BEX1	-9.91	down	0.0019
MYPN	-9.75	down	0.0048
MEST	-9.63	down	0.0048
AREG	-9.49	down	0.0106
THBD	-9.16	down	0.0034
SHCBP1	-9.11	down	0.0064
HBEGF	-8.79	down	0.0059
E2F7	-8.11	down	0.0030
SERPIND1	-7.64	down	0.0105
C15orf48	-7.46	down	0.0040
PBK	-7.32	down	0.0068
GPAT3	-7.26	down	0.0066
SPC25	-7.16	down	0.0050
CCNE2	-7.15	down	0.0124
STC1	-7.08	down	0.0038
KRT34	-7.06	down	0.0026
HMMR	-6.67	down	0.0054
GPR39	-6.64	down	0.0033
FAM111B	-6.54	down	0.0097
CEP55	-6.49	down	0.0060
PNPLA3	-6.48	down	0.0088
LINC01588	-6.28	down	0.0030
LY6K	-6.26	down	0.0030
KIAA0101	-6.14	down	0.0152
SLC14A1	-5.91	down	0.0089
RNF182	-5.86	down	0.0070
RRM2	-5.84	down	0.0061
G0S2	-5.77	down	0.0067
RGS4	-5.73	down	0.0150
DLGAP5	-5.60	down	0.0066
MKI67	-5.58	down	0.0041
BUB1	-5.35	down	0.0043
OLAH	-5.35	down	0.0100
FRMD3	-5.33	down	0.0059
NUF2	-5.27	down	0.0073
GMFG	-5.26	down	0.0039
ADRB2	-5.25	down	0.0161
SPC24	-5.25	down	0.0112
CNIH3	-5.15	down	0.0032

ANLN	-5.13	down	0.0063
ANOS1	-5.08	down	0.0067
F3	-5.01	down	0.0162
NEK2	-4.97	down	0.0061
EBF3	-4.93	down	0.0030
GPRC5A	-4.91	down	0.0025
DPP4	-4.90	down	0.0040
MET	-4.90	down	0.0026
INSC	-4.88	down	0.0094
POLE2	-4.88	down	0.0108
MPP4	-4.87	down	0.0142
FMN1	-4.84	down	0.0018
FZD8	-4.84	down	0.0068
NDP	-4.84	down	0.0072
KIF14	-4.74	down	0.0077
HJURP	-4.68	down	0.0092
CDKN3	-4.66	down	0.0030
PLK4	-4.66	down	0.0165
CASC5	-4.64	down	0.0134
HAS3	-4.64	down	0.0056
CDC20	-4.62	down	0.0130
LAMC2	-4.60	down	0.0110
GLDC	-4.58	down	0.0352
TM4SF18	-4.55	down	0.0166
ZNF185	-4.54	down	0.0079
TCF21	-4.51	down	0.0069
RAD51AP1	-4.45	down	0.0096
ADORA2B	-4.43	down	0.0032
ESCO2	-4.41	down	0.0083
KIF20A	-4.40	down	0.0044
MARCH4	-4.39	down	0.0062
RFX8	-4.39	down	0.0073
ZNF367	-4.38	down	0.0177
FJX1	-4.36	down	0.0030
NUSAP1	-4.35	down	0.0086
MYBL1	-4.34	down	0.0064
LPXN	-4.33	down	0.0048
PREX1	-4.33	down	0.0027
MMP3	-4.31	down	0.0423
AURKA	-4.28	down	0.0057
CNR1	-4.27	down	0.0178
KIF23	-4.23	down	0.0045
TRIP13	-4.23	down	0.0095
IFI30	-4.22	down	0.0030
BUB1B	-4.20	down	0.0043
FOSL1	-4.18	down	0.0026
CENPA	-4.17	down	0.0117
SKA1	-4.16	down	0.0093
PRC1	-4.14	down	0.0055
SGOL2	-4.13	down	0.0318
CD74	-4.11	down	0.0026
FAM24B	-4.10	down	0.0050
DEPDC1	-4.09	down	0.0176
ERCC6L	-4.06	down	0.0132

PAQR5	-4.05	down	0.0059
TMEM132B	-4.05	down	0.0147
BIRC5	-4.04	down	0.0055
DKK1	-4.04	down	0.0038
GINS4	-4.04	down	0.0093
SMTN	-4.04	down	0.0074
ASPM	-4.02	down	0.0048
KIF2C	-4.00	down	0.0079
LAMA1	-3.98	down	0.0036
TOP2A	-3.97	down	0.0068
CENPH	-3.96	down	0.0141
PTTG1	-3.94	down	0.0054
RACGAP1	-3.92	down	0.0110
AGTR1	-3.91	down	0.0025
DUSP4	-3.90	down	0.0057
GPR68	-3.89	down	0.0113
SPHK1	-3.88	down	0.0088
ABI3BP	-3.86	down	0.0033
MAD2L1	-3.86	down	0.0202
MND1	-3.86	down	0.0219
CDK1	-3.85	down	0.0255
SOST	-3.84	down	0.0338
TCF19	-3.83	down	0.0098
ORC6	-3.79	down	0.0073
PRR11	-3.79	down	0.0193
NAV2	-3.78	down	0.0033
PLXNA2	-3.78	down	0.0260
TTK	-3.78	down	0.0081
KIAA1524	-3.76	down	0.0219
TMCC3	-3.76	down	0.0147
SPON2	-3.75	down	0.0025
TK1	-3.75	down	0.0040
LY96	-3.74	down	0.0118
FAM212B	-3.73	down	0.0090
ARHGAP18	-3.72	down	0.0084
PORCN	-3.72	down	0.0076
TNFSF18	-3.71	down	0.0670
KRT7	-3.70	down	0.0270
UBE2C	-3.70	down	0.0099
ARHGDIB	-3.69	down	0.0109
HPCAL1	-3.64	down	0.0026
ASF1B	-3.63	down	0.0181
CDC6	-3.60	down	0.0102
BATF	-3.59	down	0.0056
RGS20	-3.59	down	0.0119
LAPTM5	-3.58	down	0.0629
BCAR3	-3.57	down	0.0142
CDCA8	-3.57	down	0.0082
SMOC1	-3.56	down	0.0102
GTSE1	-3.53	down	0.0095
SLC38A5	-3.53	down	0.0068
DCBLD2	-3.52	down	0.0158
NDC80	-3.52	down	0.0140
NRIP3	-3.52	down	0.0498

CENPI	-3.50	down	0.0048
NCAPG	-3.48	down	0.0062
RPS27A	-3.48	down	0.0597
TACC3	-3.47	down	0.0099
DIAPH3	-3.46	down	0.0022
PNP	-3.45	down	0.0048
CDCA3	-3.44	down	0.0168
CKAP2L	-3.43	down	0.0059
ITGA3	-3.43	down	0.0026
CENPF	-3.42	down	0.0048
NCEH1	-3.40	down	0.0155
SKA3	-3.39	down	0.0118
SPAG5	-3.39	down	0.0048
MTHFD2L	-3.37	down	0.0279
AK5	-3.35	down	0.0056
CCNB2	-3.35	down	0.0068
GRAMD1B	-3.35	down	0.0339
SLC20A1	-3.34	down	0.0026
KNSTRN	-3.31	down	0.0065
SVIP	-3.31	down	0.0429
HMGA1	-3.29	down	0.0051
CDCA2	-3.28	down	0.0165
CKS1B	-3.28	down	0.0094
CHAC2	-3.27	down	0.0121
SNORD17	-3.27	down	0.1027
ETV4	-3.26	down	0.0040
CORO2B	-3.24	down	0.0085
GSAP	-3.24	down	0.0064
MAPKAPK3	-3.24	down	0.0059
MCTP1	-3.23	down	0.0373
IL12A	-3.22	down	0.0153
CCNA2	-3.21	down	0.0050
MTFR2	-3.21	down	0.0236
SMURF2	-3.21	down	0.0041
SLC22A4	-3.20	down	0.0080
TYMS	-3.19	down	0.0063
BMPER	-3.18	down	0.0094
CIITA	-3.18	down	0.0188
EDN1	-3.18	down	0.0288
IKZF2	-3.18	down	0.0121
BRCA2	-3.17	down	0.0125
CCNB1	-3.17	down	0.0088
FAM64A	-3.16	down	0.0064
CENPN	-3.15	down	0.0060
POLQ	-3.13	down	0.0095
GPNMB	-3.12	down	0.0055
KIF11	-3.12	down	0.0129
TNC	-3.11	down	0.0046
DTL	-3.10	down	0.0148
ZWINT	-3.10	down	0.0089
NDC1	-3.09	down	0.0358
SYT9	-3.09	down	0.0188
PARPBP	-3.07	down	0.0317
PFKP	-3.07	down	0.0033

TM4SF1	-3.05	down	0.0078
STX1A	-3.04	down	0.0148
VRK1	-3.04	down	0.0251
UPP1	-3.02	down	0.0113
DEPDC1B	-3.01	down	0.0132
HAUS8	-3.01	down	0.0027
GNPNAT1	-2.99	down	0.0048
DDIAS	-2.98	down	0.0282
HOPX	-2.98	down	0.0087
LYN	-2.98	down	0.0235
TUBA4A	-2.98	down	0.0051
RNASEH2A	-2.97	down	0.0064
MAP3K7CL	-2.96	down	0.0105
TMEM171	-2.96	down	0.0102
OIP5	-2.94	down	0.0147
STEAP3	-2.94	down	0.0058
TIAM1	-2.94	down	0.0161
IGF2BP3	-2.93	down	0.0029
NEFH	-2.93	down	0.0523
NRG1	-2.93	down	0.0202
SEC14L2	-2.93	down	0.0060
SH2B3	-2.93	down	0.0058
TROAP	-2.93	down	0.0155
CENPE	-2.92	down	0.0103
CITED4	-2.92	down	0.0056
DNMBP	-2.92	down	0.0025
PDCD1LG2	-2.91	down	0.0056
GINS2	-2.90	down	0.0180
ERCC6	-2.89	down	0.0153
BRI3	-2.88	down	0.0447
CIT	-2.88	down	0.0064
BIRC3	-2.87	down	0.0168
NOX4	-2.87	down	0.0739
STIL	-2.87	down	0.0047
RPL27A	-2.85	down	0.0018
ADGRE5	-2.84	down	0.0238
FAM83D	-2.83	down	0.0121
VEPH1	-2.83	down	0.0440
ARHGAP22	-2.82	down	0.0030
C17orf96	-2.82	down	0.0831
HCG22	-2.82	down	0.0368
ATP8A2	-2.81	down	0.0105
BRCA1	-2.81	down	0.0166
CDC25C	-2.81	down	0.0160
KIF20B	-2.81	down	0.0082
DBF4	-2.79	down	0.0347
SPRY2	-2.79	down	0.0113
ARHGAP11A	-2.78	down	0.0294
NOTCH3	-2.78	down	0.0264
SS18	-2.77	down	0.0097
ZWILCH	-2.77	down	0.0115
FAM53A	-2.76	down	0.0033
CENPU	-2.75	down	0.0203
EFCAB11	-2.75	down	0.0048

KIFC1	-2.75	down	0.0208
C1orf112	-2.74	down	0.0184
WHSC1	-2.73	down	0.0132
LETM2	-2.72	down	0.0067
CENPM	-2.71	down	0.0181
PALMD	-2.70	down	0.0136
HSPA8	-2.69	down	0.0103
IL31RA	-2.69	down	0.0029
MMP16	-2.68	down	0.0063
NUMB	-2.68	down	0.1504
TPX2	-2.68	down	0.0157
WDR76	-2.68	down	0.0223
AGER	-2.67	down	0.1244
GPANK1	-2.67	down	0.0049
MASTL	-2.67	down	0.0198
NETO2	-2.67	down	0.0246
MCFD2	-2.66	down	0.0077
ASB5	-2.65	down	0.0176
CLEC2B	-2.65	down	0.0101
CDCA5	-2.64	down	0.0136
CEP128	-2.64	down	0.0579
PINK1	-2.64	down	0.0181
RGMB	-2.64	down	0.0034
SLC26A4	-2.64	down	0.0330
BTBD11	-2.63	down	0.0246
CLMN	-2.63	down	0.0186
FANCD2	-2.63	down	0.0127
SMC2	-2.63	down	0.0136
MCM8	-2.62	down	0.0444
SMYD3	-2.61	down	0.0345
MELK	-2.60	down	0.0136
TMEM158	-2.60	down	0.0048
CDH2	-2.59	down	0.0116
PBX3	-2.59	down	0.0294
SPRY4	-2.59	down	0.0311
ZNF726	-2.59	down	0.0280
EPS8	-2.58	down	0.0712
FCRLB	-2.58	down	0.0602
KCTD12	-2.58	down	0.0322
NEDD4	-2.58	down	0.1214
NSG1	-2.58	down	0.0291
DAW1	-2.57	down	0.0107
LRR1	-2.57	down	0.0083
MCM4	-2.57	down	0.0122
PRKCH	-2.57	down	0.0370
TFPI	-2.57	down	0.0061
AFG3L2	-2.56	down	0.0129
FEN1	-2.56	down	0.0139
NF2	-2.56	down	0.0050
INA	-2.55	down	0.0599
KCNMA1	-2.55	down	0.0068
MCM10	-2.55	down	0.0821
LRRC32	-2.54	down	0.0101
MLPH	-2.54	down	0.0076

POC1A	-2.54	down	0.0188
SLC16A3	-2.54	down	0.0041
DRD2	-2.53	down	0.1585
MAP2K1	-2.53	down	0.0260
MAPK13	-2.53	down	0.0076
HSD17B10	-2.52	down	0.0041
MPZ	-2.51	down	0.0150
NOTCH1	-2.51	down	0.0998
PPP1R3C	-2.51	down	0.0094
C18orf54	-2.50	down	0.0045
HERC4	-2.50	down	0.0068
WDHD1	-2.50	down	0.0190
CENPK	-2.49	down	0.0097
MAMLD1	-2.49	down	0.0184
SPDL1	-2.49	down	0.0190
TNFAIP8L3	-2.49	down	0.0119
NAV3	-2.48	down	0.0084
PLEK2	-2.48	down	0.0218
TLDC1	-2.48	down	0.0829
UBASH3B	-2.48	down	0.0237
ESR2	-2.47	down	0.0061
FOXM1	-2.47	down	0.0238
KIF15	-2.47	down	0.0058
TGM2	-2.47	down	0.0445
ECT2	-2.46	down	0.0081
KRT15	-2.46	down	0.0160
TIPARP	-2.45	down	0.0094
TM4SF19	-2.45	down	0.0813
UTP11L	-2.45	down	0.0190
BAIAP2	-2.44	down	0.0076
CARD10	-2.44	down	0.0408
NRROS	-2.44	down	0.0218
FAM107B	-2.43	down	0.0157
IGFBP3	-2.43	down	0.1522
NCAPG2	-2.43	down	0.0129
RRM1	-2.43	down	0.0076
SYNJ2	-2.43	down	0.0034
TEX30	-2.43	down	0.0063
ANKH	-2.42	down	0.0036
EPHB1	-2.42	down	0.0075
GSTCD	-2.42	down	0.0140
IRX3	-2.42	down	0.0343
RAD18	-2.42	down	0.0244
SLC37A2	-2.42	down	0.0130
SNHG12	-2.42	down	0.0450
TBCCD1	-2.42	down	0.0271
TMED4	-2.42	down	0.0064
ASS1	-2.41	down	0.0031
EVI2B	-2.41	down	0.0081
FANCI	-2.41	down	0.0073
GGH	-2.41	down	0.0093
AQP1	-2.40	down	0.0181
LINC01119	-2.40	down	0.0121
SEMA7A	-2.40	down	0.0095

CENPL	-2.39	down	0.0072
CKS2	-2.39	down	0.0195
KIF18A	-2.39	down	0.0316
PTPRJ	-2.39	down	0.0103
AHNAK2	-2.38	down	0.0054
ATAD2	-2.38	down	0.0152
CLSTN2	-2.38	down	0.0026
DYNLRB1	-2.38	down	0.0030
EFHD2	-2.38	down	0.0068
GINS1	-2.38	down	0.0163
SMC4	-2.38	down	0.1092
CDCA4	-2.37	down	0.0449
FAM167A	-2.37	down	0.0116
SLC43A3	-2.37	down	0.0033
CPA3	-2.36	down	0.0108
MT1A	-2.36	down	0.0102
MT1G	-2.35	down	0.0232
NAV1	-2.35	down	0.0209
AUNIP	-2.34	down	0.0248
MMS22L	-2.34	down	0.0228
MOK	-2.34	down	0.0314
ADK	-2.33	down	0.0148
CENPW	-2.33	down	0.0191
DKK2	-2.32	down	0.0228
GALNT3	-2.32	down	0.0025
TMPO	-2.32	down	0.1028
TOX2	-2.32	down	0.0087
ULBP1	-2.32	down	0.0127
AURKB	-2.31	down	0.0272
CARS2	-2.31	down	0.0133
PTGER4	-2.31	down	0.0036
STRIP2	-2.31	down	0.0036
TBC1D8	-2.31	down	0.0048
CADPS	-2.30	down	0.0121
DHFR	-2.30	down	0.1207
SFR1	-2.30	down	0.0068
SLITRK2	-2.30	down	0.0148
AMPD3	-2.29	down	0.0139
AXL	-2.29	down	0.0107
COL13A1	-2.29	down	0.0079
KNTC1	-2.29	down	0.0039
WDR66	-2.29	down	0.0245
PYGB	-2.28	down	0.0121
UAP1	-2.28	down	0.0117
SEMA3C	-2.27	down	0.1039
TDP1	-2.27	down	0.0203
FABP5	-2.26	down	0.0132
MT1E	-2.26	down	0.0041
CPNE7	-2.25	down	0.0132
GPSM2	-2.25	down	0.0284
LMNB1	-2.25	down	0.0208
RAB27B	-2.25	down	0.0136
ALDH1A3	-2.24	down	0.0132
DSN1	-2.24	down	0.0025

MATN2	-2.24	down	0.0255
IQGAP3	-2.23	down	0.0281
LGALS8	-2.23	down	0.0282
MSRA	-2.23	down	0.0048
PCGF5	-2.23	down	0.0375
STMN1	-2.23	down	0.0117
ABCD3	-2.22	down	0.0874
FGF5	-2.22	down	0.0210
HHIP	-2.22	down	0.0113
MCM7	-2.22	down	0.0329
PPFIBP1	-2.22	down	0.0131
EFTUD1	-2.21	down	0.0222
FTSJ1	-2.21	down	0.0051
GSTO1	-2.21	down	0.0080
BHLHE41	-2.20	down	0.0121
CAV2	-2.20	down	0.0087
KIAA1549L	-2.20	down	0.0208
NUDT19	-2.20	down	0.0031
RPL37A	-2.20	down	0.1625
UACA	-2.20	down	0.0121
CCDC150	-2.19	down	0.0155
KPNA2	-2.19	down	0.0032
KRT19	-2.19	down	0.0260
PIGW	-2.18	down	0.0156
SLC22A23	-2.18	down	0.0297
APITD1	-2.17	down	0.0209
DNAJC9	-2.17	down	0.0181
KDR	-2.17	down	0.0234
MT1X	-2.16	down	0.0043
NMNAT2	-2.16	down	0.0496
PAPL	-2.16	down	0.0653
CDH13	-2.15	down	0.0274
KLHL7	-2.15	down	0.0334
NCAPD3	-2.15	down	0.0175
NCAPH	-2.15	down	0.0273
POLA2	-2.15	down	0.0288
PRDM8	-2.15	down	0.0159
PSMC3IP	-2.15	down	0.0215
ASXL2	-2.14	down	0.0967
C11orf68	-2.14	down	0.0102
CHST1	-2.14	down	0.0588
CRACR2A	-2.14	down	0.0166
STRA13	-2.14	down	0.0053
CACYBP	-2.13	down	0.0078
JRK	-2.13	down	0.0698
NT5E	-2.13	down	0.0102
DEFB103A	-2.12	down	0.0297
NUP88	-2.12	down	0.0055
SFXN3	-2.12	down	0.0043
TFAP2C	-2.11	down	0.0056
GRB14	-2.10	down	0.0451
LRRC2	-2.10	down	0.0275
MLKL	-2.10	down	0.0101
PLAUR	-2.10	down	0.0202

PRIM1	-2.10	down	0.0194
H2AFX	-2.09	down	0.0315
RBM3	-2.09	down	0.0081
SPATA5	-2.09	down	0.0114
UBE2T	-2.09	down	0.0089
ATRNL1	-2.08	down	0.0140
CCDC18	-2.08	down	0.0759
EPG5	-2.08	down	0.0111
FIGNL1	-2.08	down	0.0178
GLRX3	-2.08	down	0.0144
SFRP1	-2.08	down	0.0048
ADGRL4	-2.07	down	0.0363
COL4A1	-2.07	down	0.0130
DDX39A	-2.07	down	0.0133
UCHL5	-2.07	down	0.0226
ZNF267	-2.07	down	0.0122
CHAF1A	-2.06	down	0.0125
SH3KBP1	-2.06	down	0.0418
SQRDL	-2.06	down	0.0097
C2CD2	-2.05	down	0.0033
CCDC81	-2.05	down	0.0230
COX20	-2.05	down	0.0150
SMAGP	-2.05	down	0.0038
AFAP1L1	-2.04	down	0.0591
BUB3	-2.04	down	0.0161
EZH2	-2.04	down	0.0461
FBXO5	-2.04	down	0.0224
H2AFZ	-2.04	down	0.0118
ULBP3	-2.04	down	0.0844
GSG1	-2.03	down	0.0420
IL11	-2.03	down	0.0558
PFKFB4	-2.03	down	0.0138
RPS28	-2.03	down	0.1099
TPRKB	-2.03	down	0.0034
USF2	-2.03	down	0.0191
WDFY2	-2.03	down	0.0203
CKAP2	-2.02	down	0.0119
EVI2A	-2.02	down	0.0520
MBP	-2.02	down	0.0374
PCBD2	-2.02	down	0.0168
SERPINE2	-2.02	down	0.0100
ANPEP	-2.01	down	0.0243
APOLD1	-2.01	down	0.0185
COX7A2	-2.01	down	0.0050
MROH1	-2.01	down	0.0121
PDSS1	-2.01	down	0.0109
AZI2	2.01	up	0.0380
BTG1	2.01	up	0.0648
GXYLT2	2.01	up	0.0048
IFI44L	2.01	up	0.0569
IL16	2.01	up	0.0605
ISYNA1	2.01	up	0.0183
MID1	2.01	up	0.0081
MNX1	2.01	up	0.0063

PNKD	2.01	up	0.0037
TET1	2.01	up	0.0062
TRIB3	2.01	up	0.0055
XIST	2.01	up	0.0279
ZFP36L2	2.01	up	0.1164
AMOT	2.02	up	0.0105
ASIC1	2.02	up	0.0113
CADPS2	2.02	up	0.0411
FANK1	2.02	up	0.0150
RHEB	2.02	up	0.1014
SKAP2	2.02	up	0.0232
SOX12	2.02	up	0.0653
WNT2	2.02	up	0.0162
AASS	2.03	up	0.0232
C11orf21	2.03	up	0.0374
C2orf88	2.03	up	0.0246
FOXF1	2.03	up	0.0043
LZTS1	2.03	up	0.0504
MME	2.03	up	0.0454
PI15	2.03	up	0.0445
SH3BP5	2.03	up	0.0192
TRPV4	2.03	up	0.0214
ZNF532	2.03	up	0.0100
F11R	2.04	up	0.0121
FAM65C	2.04	up	0.0317
FILIP1L	2.04	up	0.0489
MOCOS	2.04	up	0.0163
PRSS23	2.04	up	0.0041
SAT1	2.04	up	0.0621
TMEM65	2.04	up	0.0270
TSC22D1	2.04	up	0.0117
CKB	2.05	up	0.0485
IGDCC4	2.05	up	0.0066
LXN	2.05	up	0.0571
MSI1	2.05	up	0.0056
PHC1	2.05	up	0.0467
PIP	2.05	up	0.0069
ROR1	2.05	up	0.0056
ULK2	2.05	up	0.0338
CNN2	2.06	up	0.0841
IFNGR1	2.06	up	0.0762
ISG15	2.06	up	0.0104
JAK3	2.06	up	0.0243
NXN	2.06	up	0.0062
SLC7A8	2.06	up	0.0605
TOB1	2.06	up	0.0239
ALPK1	2.07	up	0.0059
CCNL1	2.07	up	0.1241
DSE	2.07	up	0.0420
GLI3	2.07	up	0.0029
GPT2	2.07	up	0.0048
LRP1	2.07	up	0.0400
PAPPA	2.07	up	0.0171
PCDHB5	2.07	up	0.0325

PRTG	2.07	up	0.0083
TAGLN	2.07	up	0.0048
TMEM56	2.07	up	0.0221
UST	2.07	up	0.0256
AUTS2	2.08	up	0.0045
BNC2	2.08	up	0.0033
CCDC181	2.08	up	0.0358
DNAJC12	2.08	up	0.0121
KCNJ15	2.08	up	0.0097
MAGI1	2.08	up	0.0121
RBMS1	2.08	up	0.0099
RBPMS2	2.08	up	0.0087
USP44	2.08	up	0.0980
ACTA2	2.09	up	0.0031
CORO2A	2.09	up	0.0437
CYP2U1	2.09	up	0.1374
HOXC6	2.09	up	0.0612
IDH2	2.09	up	0.0029
PCDHGA1	2.09	up	0.0539
PGM2L1	2.09	up	0.0067
PXYLP1	2.09	up	0.0077
STK38L	2.09	up	0.0412
CH25H	2.10	up	0.1261
CTTNBP2	2.10	up	0.0341
DNMT3A	2.10	up	0.0519
EPHX1	2.10	up	0.0178
HIVEP2	2.10	up	0.0420
MITF	2.10	up	0.0250
MLF1	2.10	up	0.0169
PIK3R1	2.10	up	0.0544
SEPT11	2.10	up	0.0111
SOBP	2.10	up	0.0237
ST5	2.10	up	0.0073
APOL1	2.11	up	0.0122
CAPS	2.11	up	0.0260
FOXG1	2.11	up	0.0208
KLF6	2.11	up	0.1178
NOL4L	2.11	up	0.0089
PHF14	2.11	up	0.1434
STX17	2.11	up	0.0572
TCTN1	2.11	up	0.0288
TMTC4	2.11	up	0.0133
FAM171B	2.12	up	0.0205
GALNT10	2.12	up	0.0048
ORMDL1	2.12	up	0.0315
LOX	2.13	up	0.0059
NLGN1	2.13	up	0.0561
RAB15	2.13	up	0.0150
SIAE	2.13	up	0.0062
SOWAHD	2.13	up	0.0163
ACSS3	2.14	up	0.0384
CCDC8	2.14	up	0.0145
FGFR2	2.14	up	0.0843
INHBA	2.14	up	0.0509

MOB3B	2.14	up	0.0969
PELI1	2.14	up	0.1345
SLC7A11	2.14	up	0.0123
TLR3	2.14	up	0.0465
TRPC3	2.14	up	0.0364
BHLHE40	2.15	up	0.0954
LRIG3	2.15	up	0.0117
PRPH2	2.15	up	0.0093
ST8SIA1	2.15	up	0.0193
TBX1	2.15	up	0.0087
TNFSF13B	2.15	up	0.0108
ALDH6A1	2.16	up	0.0146
ATP8B2	2.16	up	0.0044
GGT7	2.16	up	0.0648
IGF1R	2.16	up	0.0075
PIK3IP1	2.16	up	0.0087
RAB2A	2.16	up	0.0328
CNTNAP1	2.17	up	0.0128
CTSF	2.17	up	0.0029
GUCY1B3	2.17	up	0.0225
HHAT	2.17	up	0.0154
IL1R2	2.17	up	0.0117
JDP2	2.17	up	0.0110
ODF3B	2.17	up	0.0187
OSMR	2.17	up	0.0575
POU4F1	2.17	up	0.0229
SLC25A23	2.17	up	0.0720
STEAP4	2.17	up	0.0097
CLSTN3	2.18	up	0.0403
FAM110B	2.18	up	0.0113
GCA	2.18	up	0.0140
RASL12	2.18	up	0.0165
SESN2	2.18	up	0.0038
SH3GL3	2.18	up	0.0051
ZBTB16	2.18	up	0.0092
ASB9	2.19	up	0.0358
DTWD1	2.19	up	0.0934
RFTN1	2.19	up	0.0047
RRAGB	2.19	up	0.0250
SH3PXD2A	2.19	up	0.0092
THRA	2.19	up	0.0023
APBA2	2.20	up	0.0091
ARHGEF3	2.20	up	0.0332
CCDC170	2.20	up	0.0569
DTNA	2.20	up	0.1565
HYKK	2.20	up	0.0229
ITGA4	2.20	up	0.0697
NATD1	2.20	up	0.0280
PARP9	2.20	up	0.0960
UBE2G1	2.20	up	0.0238
ASCC1	2.21	up	0.0667
B3GALT2	2.21	up	0.0429
BCL11A	2.21	up	0.0032
CDKL2	2.21	up	0.0360

FAM117B	2.21	up	0.0917
HHIPL2	2.21	up	0.0099
MFGE8	2.21	up	0.0173
RNASE4	2.21	up	0.1200
SLIT3	2.21	up	0.0218
CFB	2.22	up	0.0057
DMD	2.22	up	0.0136
FOXO3	2.22	up	0.0055
H2AFY2	2.22	up	0.0066
KCNMB4	2.22	up	0.0147
MDK	2.22	up	0.0026
TANC1	2.22	up	0.0539
EDNRB	2.23	up	0.0317
FOXF2	2.23	up	0.0080
PSAT1	2.23	up	0.0162
RBCK1	2.23	up	0.0229
TMEM47	2.23	up	0.0196
TTC17	2.23	up	0.0488
TXNIP	2.23	up	0.0568
VASH2	2.23	up	0.0346
ASRGL1	2.24	up	0.0045
CASP1	2.24	up	0.0170
CDKN1C	2.24	up	0.0177
COL1A1	2.24	up	0.0395
ELOVL4	2.24	up	0.0018
KIF26A	2.24	up	0.0296
NIM1K	2.24	up	0.0352
PABPC4L	2.24	up	0.0034
SELM	2.25	up	0.0116
SLC16A12	2.25	up	0.0209
SYNE2	2.25	up	0.0077
ASNS	2.26	up	0.0089
CSPG5	2.26	up	0.0388
FNDC3B	2.26	up	0.0156
HGF	2.26	up	0.0221
RARB	2.26	up	0.0107
TENM3	2.26	up	0.0225
THY1	2.26	up	0.0249
TMEM108	2.26	up	0.0282
IGFBP6	2.27	up	0.0088
KLF11	2.27	up	0.0056
MARC2	2.27	up	0.0388
MYH10	2.27	up	0.0313
RARRES3	2.27	up	0.0054
CLCA2	2.28	up	0.0522
PTGFRN	2.28	up	0.0376
RAD1	2.28	up	0.0927
TMEM140	2.28	up	0.0181
VWA5A	2.28	up	0.0161
WLS	2.28	up	0.0155
CHD3	2.29	up	0.0152
CRELD1	2.29	up	0.0034
OAS1	2.29	up	0.0393
RUNX2	2.29	up	0.0099

ABCC5	2.30	up	0.0415
GPR155	2.30	up	0.1295
MAN1C1	2.30	up	0.0587
MEX3B	2.30	up	0.0166
NEO1	2.30	up	0.0100
EPSTI1	2.31	up	0.0166
HIST1H2AC	2.31	up	0.0466
LGMN	2.31	up	0.0111
MSX2	2.31	up	0.0309
ST3GAL1	2.31	up	0.0163
TMEM98	2.31	up	0.0046
CABYR	2.32	up	0.0029
CCDC144A	2.32	up	0.0947
MALAT1	2.32	up	0.0585
NFIX	2.32	up	0.0207
PTPRG	2.32	up	0.0257
TMEM133	2.32	up	0.0225
BHMT2	2.34	up	0.0521
CD55	2.34	up	0.1256
NISCH	2.34	up	0.0071
CHSY3	2.35	up	0.0376
GAA	2.35	up	0.0073
LRRC49	2.35	up	0.1444
GHR	2.36	up	0.0179
HOXD10	2.36	up	0.0032
MEX3A	2.36	up	0.0323
NYNRIN	2.36	up	0.0085
ADAM12	2.37	up	0.0034
C14orf39	2.37	up	0.0156
FABP3	2.37	up	0.1070
LRRK2	2.37	up	0.0496
STOM	2.37	up	0.0129
JPH1	2.38	up	0.0610
NMU	2.38	up	0.0163
RDH10	2.38	up	0.0319
BEX4	2.39	up	0.0078
CDH6	2.39	up	0.0062
CLTCL1	2.39	up	0.0100
LDLR	2.39	up	0.0111
KCNQ1OT1	2.40	up	0.0076
SNX18	2.40	up	0.0180
CFH	2.41	up	0.0379
KCTD15	2.41	up	0.0932
SALL1	2.41	up	0.0215
C16orf45	2.42	up	0.0034
SEPT6	2.42	up	0.0034
MCC	2.43	up	0.0408
PLXND1	2.43	up	0.0133
RBPMS	2.43	up	0.0119
SLC26A10	2.43	up	0.0130
TNS3	2.43	up	0.0022
TP53INP1	2.43	up	0.0078
FSIP1	2.44	up	0.0256
GPC3	2.44	up	0.0174

GRIA3	2.44	up	0.0317
LITAF	2.44	up	0.0032
TYMP	2.44	up	0.0244
EIF4E3	2.45	up	0.0177
NEDD9	2.45	up	0.0091
NHS	2.45	up	0.0114
PRDM6	2.45	up	0.0230
RASL11A	2.45	up	0.0118
RGCC	2.45	up	0.0177
RUNX1T1	2.45	up	0.0582
SUSD6	2.45	up	0.0028
SLC13A3	2.46	up	0.0034
TGFBR1	2.46	up	0.0056
AGPAT4	2.47	up	0.0063
FBXO2	2.47	up	0.0212
FZD5	2.47	up	0.0463
NPR3	2.47	up	0.0035
REV3L	2.47	up	0.0537
ADD3	2.48	up	0.0164
CPEB1	2.48	up	0.0050
CPQ	2.48	up	0.0266
LRRFIP1	2.48	up	0.0181
PAM	2.48	up	0.0245
TSC22D3	2.48	up	0.0338
ANKRD6	2.49	up	0.0175
CA13	2.49	up	0.0342
CPVL	2.49	up	0.0162
FSTL1	2.49	up	0.0281
SLC2A12	2.49	up	0.0156
SUSD3	2.49	up	0.0087
TDRD6	2.49	up	0.0323
KGFLP2	2.50	up	0.0087
MBNL2	2.50	up	0.0471
SEMA3B	2.50	up	0.0277
ZFPM2	2.50	up	0.0172
APOD	2.51	up	0.0033
BAALC	2.51	up	0.0220
IFIH1	2.51	up	0.1002
SPOCK1	2.51	up	0.0401
ARHGEF25	2.52	up	0.0088
GBP1	2.52	up	0.0168
NFATC4	2.52	up	0.0072
SELENBP1	2.52	up	0.0125
ADARB1	2.53	up	0.0101
C11orf63	2.53	up	0.0196
FAM84A	2.53	up	0.0208
MARC1	2.53	up	0.0356
PDGFRB	2.53	up	0.0050
TCEAL7	2.53	up	0.0182
ZNF521	2.53	up	0.0089
ENOX1	2.54	up	0.0062
ZNF204P	2.54	up	0.0379
ANGPTL2	2.55	up	0.0392
LMCD1	2.55	up	0.0048

VAMP8	2.55	up	0.0261
CALD1	2.56	up	0.0771
N4BP2	2.56	up	0.1149
FAM20C	2.57	up	0.0088
JAM2	2.57	up	0.0026
KLHL24	2.57	up	0.0210
ST6GAL1	2.57	up	0.0336
TGFB2	2.57	up	0.0162
ADCY9	2.58	up	0.0026
CNTN3	2.58	up	0.0896
H1FX	2.58	up	0.0617
PDGFRA	2.58	up	0.0068
RECK	2.58	up	0.0145
SPATA18	2.58	up	0.0121
ZFP36	2.58	up	0.1427
HES6	2.59	up	0.0269
PDE7B	2.59	up	0.0053
ABCC3	2.60	up	0.0088
AEBP1	2.60	up	0.0182
CAND2	2.60	up	0.0135
HOXB9	2.60	up	0.0101
SLC30A1	2.60	up	0.0097
STON1	2.60	up	0.0309
GALNT15	2.61	up	0.0405
ARRDC4	2.62	up	0.0320
CMTM8	2.62	up	0.0050
JADE1	2.62	up	0.0302
PLXDC2	2.62	up	0.0085
HOXD13	2.63	up	0.0149
ZCCHC24	2.63	up	0.0083
CHAC1	2.64	up	0.0025
FAM69A	2.64	up	0.0293
KREMEN1	2.64	up	0.0240
MFAP3L	2.64	up	0.0097
NRN1	2.64	up	0.0114
OLFML1	2.64	up	0.0048
FADS2	2.65	up	0.0753
TRIB1	2.65	up	0.1432
CITED1	2.66	up	0.0063
COL5A2	2.66	up	0.0037
EYA2	2.66	up	0.0512
GK	2.66	up	0.0058
MRGPRF	2.66	up	0.0039
TBC1D2B	2.66	up	0.0077
ITM2C	2.67	up	0.0154
PDE4DIP	2.67	up	0.0578
THBS2	2.67	up	0.0192
ALCAM	2.68	up	0.0333
PTGS1	2.68	up	0.0072
RAVER2	2.68	up	0.0133
ROBO1	2.68	up	0.0415
SLC7A5	2.68	up	0.0090
ANTXR1	2.69	up	0.0033
ENTPD3	2.69	up	0.0642

SUSD2	2.69	up	0.0583
APOC1	2.70	up	0.0135
DNM1	2.70	up	0.0026
TLE1	2.70	up	0.0050
IER5L	2.71	up	0.0036
PRKAR2B	2.72	up	0.0323
PTCH1	2.72	up	0.0026
BBS9	2.73	up	0.0226
METTL7A	2.73	up	0.0083
BST1	2.74	up	0.0369
DSP	2.74	up	0.0070
RAB30	2.74	up	0.0220
RORB	2.74	up	0.0190
TIMP4	2.74	up	0.0101
SERPINF1	2.75	up	0.0031
CRYAB	2.76	up	0.0019
IFI6	2.76	up	0.0138
SPAG16	2.76	up	0.0146
LUM	2.77	up	0.0257
PLTP	2.77	up	0.0055
MMP2	2.78	up	0.0039
STAT3	2.78	up	0.0041
HPSE	2.79	up	0.0301
IRF2BP2	2.79	up	0.0161
MSC	2.80	up	0.0155
RPS6KA2	2.81	up	0.0045
GDF5	2.83	up	0.0072
TNFAIP8	2.83	up	0.0755
ABCA5	2.84	up	0.0061
PMAIP1	2.84	up	0.0145
SDC2	2.84	up	0.0105
SMO	2.84	up	0.0038
THBS3	2.84	up	0.0072
ADAMTS9	2.86	up	0.0034
FAM46A	2.86	up	0.0118
REEP2	2.86	up	0.0095
IL1R1	2.87	up	0.0208
TP53I11	2.87	up	0.0182
PLBD1	2.88	up	0.0095
AKR1C3	2.89	up	0.0112
NDRG4	2.89	up	0.0052
OSBPL1A	2.90	up	0.0092
HEG1	2.91	up	0.0083
HOXA5	2.91	up	0.0144
MMD	2.92	up	0.0309
NAP1L3	2.92	up	0.0537
RASSF4	2.92	up	0.0415
CYBRD1	2.93	up	0.0218
DPYSL3	2.93	up	0.0026
MRO	2.93	up	0.0097
SCN4B	2.93	up	0.0275
ECM2	2.94	up	0.0086
CYP1B1	2.95	up	0.0119
KIT	2.95	up	0.0362

P4HA3	2.95	up	0.0231
XAF1	2.95	up	0.0705
CDIPT	2.96	up	0.0063
ITPR1	2.96	up	0.0312
PTK7	2.97	up	0.0073
BMP1	2.98	up	0.0084
EPDR1	2.98	up	0.0469
KCNT2	2.99	up	0.0155
CKMT1A	3.00	up	0.0248
CRISPLD2	3.00	up	0.0147
LOXL4	3.00	up	0.0032
FBLN5	3.01	up	0.0039
PLK2	3.01	up	0.0310
HSPB3	3.02	up	0.0156
LRRC8B	3.02	up	0.0147
RHOU	3.02	up	0.0162
TRO	3.02	up	0.0081
WISP1	3.02	up	0.0288
WNT5A	3.02	up	0.0102
GBP2	3.03	up	0.0166
TBL1XR1	3.03	up	0.0303
CXorf57	3.04	up	0.0198
H3F3B	3.05	up	0.0257
PARD6G	3.05	up	0.0040
SLC22A17	3.05	up	0.0072
TSPAN9	3.05	up	0.0034
IFIT1	3.06	up	0.0177
SSBP2	3.07	up	0.0200
BRINP1	3.08	up	0.0193
E2F5	3.08	up	0.0041
DACH1	3.09	up	0.0109
HMCN1	3.09	up	0.0026
HSPA2	3.09	up	0.0032
OLFML2A	3.09	up	0.0146
SLC12A8	3.09	up	0.0071
DXO	3.10	up	0.0599
SPRY1	3.10	up	0.0056
ASAP3	3.11	up	0.0061
CHST15	3.11	up	0.0026
PRKG1	3.11	up	0.0197
IFITM1	3.12	up	0.0048
MGARP	3.12	up	0.0162
PDE5A	3.13	up	0.0096
RSAD2	3.13	up	0.0165
FLRT2	3.14	up	0.0018
HLA-C	3.16	up	0.0138
LSAMP	3.18	up	0.0116
RGMA	3.18	up	0.0162
ARID5B	3.19	up	0.0369
OLFML3	3.19	up	0.0023
PLSCR1	3.19	up	0.0235
HS6ST1	3.21	up	0.0051
ITGB8	3.21	up	0.0277
ZCCHC12	3.21	up	0.0139

DMKN	3.23	up	0.0081
ENAH	3.23	up	0.0165
TTC28	3.23	up	0.0040
MCOLN3	3.24	up	0.0089
MBTPS1	3.27	up	0.0580
COL11A1	3.28	up	0.0222
SOCS3	3.28	up	0.0102
SYNPO2	3.28	up	0.0055
CACHD1	3.29	up	0.0048
CMPK2	3.29	up	0.0124
RNF144A	3.29	up	0.0030
LDB2	3.30	up	0.0250
NFIL3	3.31	up	0.0137
SALL2	3.31	up	0.0068
C1orf198	3.32	up	0.0031
DAB2	3.32	up	0.0036
PCDHB2	3.32	up	0.0176
HDAC5	3.34	up	0.0181
NFASC	3.35	up	0.0063
PODNL1	3.36	up	0.0030
ATL1	3.38	up	0.0123
CHN1	3.38	up	0.0129
DACT1	3.41	up	0.0135
DSC2	3.42	up	0.0044
EMB	3.42	up	0.0046
PGF	3.42	up	0.0139
TMEM119	3.43	up	0.0094
RBM47	3.44	up	0.0197
COL1A2	3.46	up	0.0163
APCDD1	3.47	up	0.0102
FAM169A	3.47	up	0.0075
SLC9A9	3.48	up	0.0880
FAM13C	3.49	up	0.0076
CDC42EP5	3.50	up	0.0037
MX2	3.50	up	0.0042
KCNE3	3.51	up	0.0068
EFHC2	3.52	up	0.0195
FAM198B	3.53	up	0.0146
CXADR	3.54	up	0.0296
LRCH2	3.54	up	0.0388
ABCC6	3.55	up	0.0205
CYP39A1	3.55	up	0.0124
GAL3ST4	3.55	up	0.0025
KIAA0895	3.55	up	0.0119
F10	3.56	up	0.0036
FMO2	3.56	up	0.0284
MAP2K6	3.56	up	0.0080
PHLDB2	3.56	up	0.0128
JUN	3.57	up	0.1577
PTGFR	3.57	up	0.0192
NAP1L2	3.59	up	0.0150
COCH	3.60	up	0.0195
CTHRC1	3.60	up	0.0087
C14orf37	3.61	up	0.0058

GCH1	3.61	up	0.0067
NUPR1	3.61	up	0.0096
PNRC1	3.61	up	0.0051
PRRT2	3.62	up	0.0197
GNG7	3.63	up	0.0153
PDZRN3	3.63	up	0.0066
PRDM1	3.66	up	0.0048
PLSCR4	3.67	up	0.0147
SERPINH1	3.67	up	0.0063
FOXC1	3.68	up	0.0214
MAFB	3.68	up	0.0407
BDKRB2	3.69	up	0.0100
FHL1	3.71	up	0.0202
LHX8	3.71	up	0.0094
HS6ST2	3.72	up	0.0076
RPL31	3.75	up	0.0082
HCFC1R1	3.76	up	0.0078
HTRA3	3.76	up	0.0139
NEFL	3.76	up	0.0039
PPIC	3.77	up	0.0102
ENPP2	3.78	up	0.0153
ADGRA2	3.79	up	0.0018
KCNK1	3.79	up	0.0125
GLDN	3.80	up	0.0043
GLUL	3.80	up	0.0034
NACAD	3.80	up	0.0081
CXCL1	3.81	up	0.0026
NNMT	3.81	up	0.0026
HOXA11	3.82	up	0.0280
STK32B	3.86	up	0.0079
CHRNA1	3.87	up	0.0149
MAF	3.90	up	0.0073
REC8	3.92	up	0.0028
BCL6	3.93	up	0.0139
QPRT	3.93	up	0.0147
ZIC5	3.93	up	0.0026
STMN3	3.95	up	0.0074
CORIN	3.96	up	0.0050
EPHB6	3.97	up	0.0163
COL21A1	3.99	up	0.0068
ENTPD1	4.00	up	0.0036
MMP13	4.00	up	0.0235
TNFRSF19	4.03	up	0.0026
CPXM2	4.04	up	0.0069
GPC6	4.06	up	0.0044
CDH11	4.08	up	0.0068
HEPH	4.10	up	0.0025
SCG2	4.10	up	0.0131
BMP2	4.11	up	0.0192
RNF175	4.11	up	0.0090
GPC4	4.12	up	0.0402
ABCA8	4.13	up	0.0221
RHOBTB1	4.14	up	0.0223
EDIL3	4.15	up	0.0281

FOSL2	4.18	up	0.0054
KLF5	4.18	up	0.0122
MAGEA3	4.18	up	0.0221
DCN	4.19	up	0.0074
TPD52	4.20	up	0.0107
LRRN4CL	4.21	up	0.0115
DHRS3	4.25	up	0.0038
BMP4	4.26	up	0.0025
BICC1	4.28	up	0.0222
SSPN	4.29	up	0.0157
KLF9	4.30	up	0.0110
MPPED2	4.32	up	0.0248
MX1	4.32	up	0.0047
MAP2	4.35	up	0.0086
ABAT	4.36	up	0.0025
SLC2A10	4.36	up	0.0055
GREM2	4.38	up	0.0087
PTGIS	4.39	up	0.0030
NFKBIZ	4.41	up	0.0591
TNFAIP6	4.42	up	0.0121
PCBP3	4.44	up	0.0030
ZNF608	4.45	up	0.0095
COL12A1	4.48	up	0.0026
SCRG1	4.48	up	0.0410
COL5A1	4.53	up	0.0081
SLAIN1	4.53	up	0.0110
CLDN23	4.54	up	0.0152
HEY1	4.57	up	0.0034
JUNB	4.60	up	0.0565
COL4A5	4.61	up	0.0018
NR4A3	4.62	up	0.0819
SVEP1	4.62	up	0.0026
MFAP4	4.66	up	0.0039
SAMD5	4.67	up	0.0099
PTPRD	4.68	up	0.0031
SAMHD1	4.70	up	0.0154
CXCL5	4.71	up	0.0145
HAS2	4.71	up	0.0083
ADAMTS3	4.74	up	0.0104
TMEM150C	4.74	up	0.0123
FBXO32	4.75	up	0.0039
S1PR1	4.78	up	0.0071
TENM2	4.79	up	0.0030
PALLD	4.81	up	0.0071
CEBPD	4.86	up	0.0118
DIRAS3	4.90	up	0.0097
CD14	4.96	up	0.0030
SEL1L3	4.97	up	0.0043
ANGPT1	4.98	up	0.0097
DSG2	5.02	up	0.0033
KLF4	5.03	up	0.1605
PDGFRL	5.05	up	0.0064
C1QTNF5	5.06	up	0.0029
PBX1	5.06	up	0.0049

CACNB2	5.09	up	0.0030
SCG5	5.09	up	0.0034
PCDH18	5.11	up	0.0068
FAM71A	5.13	up	0.0264
NRCAM	5.17	up	0.0241
SLC40A1	5.19	up	0.0261
NOVA1	5.21	up	0.0048
ARHGAP26	5.22	up	0.0058
TPD52L1	5.26	up	0.0039
SLC25A27	5.29	up	0.0248
MN1	5.31	up	0.0089
C1RL	5.33	up	0.0036
ZFAND5	5.35	up	0.1661
ADAM33	5.44	up	0.0068
EFEMP1	5.47	up	0.0036
CD200	5.51	up	0.0087
FBLN1	5.51	up	0.0032
HAPLN1	5.65	up	0.0034
VCAM1	5.76	up	0.0108
RGS2	5.80	up	0.0171
CEMIP	5.83	up	0.0019
GYG2	5.83	up	0.0121
SEPT4	5.92	up	0.0188
CLU	6.03	up	0.0030
CD302	6.07	up	0.0072
MCTP2	6.07	up	0.0083
PLAU	6.10	up	0.0120
NR4A2	6.12	up	0.1155
ADH1A	6.24	up	0.0082
OMD	6.29	up	0.0026
LRRC1	6.32	up	0.0026
RASD1	6.37	up	0.0072
KLHL23	6.40	up	0.0060
SNED1	6.46	up	0.0085
SNCA	6.66	up	0.0117
CCL2	6.75	up	0.0081
LIMCH1	6.78	up	0.0034
SCN2A	6.82	up	0.0138
OBSL1	6.90	up	0.0014
DDIT4L	7.14	up	0.0057
SERPING1	7.14	up	0.0052
DIO2	7.28	up	0.0030
SULF1	7.37	up	0.0016
PRSS35	7.87	up	0.0056
COL3A1	7.98	up	0.0032
FIBIN	8.03	up	0.0059
EYA1	8.07	up	0.0030
C10orf10	8.08	up	0.0031
ZNF711	8.14	up	0.0038
LAMA2	8.24	up	0.0030
SERPINA3	8.32	up	0.0041
CXCL6	8.33	up	0.0107
THBS1	8.43	up	0.0526
FAM20A	8.53	up	0.0031

NLGN4X	8.56	up	0.0073
MASP1	8.58	up	0.0018
ID4	8.63	up	0.1505
ZIC2	8.65	up	0.0025
FRY	8.68	up	0.0074
CRISPLD1	8.74	up	0.0103
RARRES1	8.79	up	0.0048
SNCAIP	8.85	up	0.0032
HOXA10-HOXA9	8.89	up	0.0155
JAKMIP2	8.96	up	0.0026
BEX2	9.10	up	0.0030
FNBP1L	9.20	up	0.0126
SULF2	9.29	up	0.0150
LIN28B	9.85	up	0.0093
EPB41L3	10.19	up	0.0030
KIF26B	10.19	up	0.0018
VCAN	10.44	up	0.0019
RGS18	10.57	up	0.0107
CFI	10.89	up	0.0026
PIM1	11.18	up	0.0228
FGF7	11.32	up	0.0058
GAS1	11.51	up	0.0034
SEPP1	11.78	up	0.0051
NR4A1	12.18	up	0.0149
ATF3	12.77	up	0.1119
CPE	12.78	up	0.0087
MKX	12.94	up	0.0047
AMPH	12.96	up	0.0029
MXRA5	13.05	up	0.0024
GAL	13.75	up	0.0018
C1S	13.97	up	0.0025
SLC1A3	14.00	up	0.0033
SOX4	14.46	up	0.0007
SGCD	15.03	up	0.0050
ARHGAP28	15.74	up	0.0026
STAR	16.06	up	0.0059
FOSB	16.07	up	0.1779
EREG	16.96	up	0.0025
CYTL1	17.73	up	0.0043
PDGFD	18.11	up	0.0062
RBP1	20.07	up	0.0054
FMO3	20.21	up	0.0034
FOS	20.60	up	0.1497
ADH1B	21.11	up	0.0033
C1R	25.44	up	0.0025
CA2	29.83	up	0.0020
MMP1	32.40	up	0.0016
CXCL12	43.86	up	0.0030
