# Supplementary material for: KDM2B and its peptides promote the stem cells from apical papilla mediated nerve injury repair in rats by intervening EZH2 function
Source: Cell Prolif. 2024 Oct 2;58(2):e13756. doi: 10.1111/cpr.13756 (PMC11839186; doi:10.1111/cpr.13756)
Supplement: Supplementary file 6 — Table S3. [file CPR-58-e13756-s004.pdf]

**Table S3. Differential peaks of H3K4me3 in KDM2B-deleted SCAPs compared with the Scramsh group.**

| Genes | Gene ID | Chromosome | Peak Start | Peak End | H3K4me3 Enrichment (KDM2Bsh vs Scramsh) | Fold | p-Value |
|---|---|---|---|---|---|---|---|
| AAGAB | 79719 | chr15 | 67253200 | 67255199 | low | -4.00 | 0.001185 |
| AAMDC | 28971 | chr11 | 77816200 | 77822599 | high | 35.55 | 0.000372 |
| AAR2 | 25980 | chr20 | 36236000 | 36237399 | high | 7.77 | 0.000717 |
| AARS | 16 | chr16 | 70250000 | 70255199 | high | 28.88 | 0.002278 |
| AARS | 16 | chr16 | 70285000 | 70291399 | high | 35.55 | 0.002276 |
| AARS2 | 57505 | chr6 | 44310400 | 44312399 | low | -4.00 | 0.001298 |
| AATK | 9625 | chr17 | 81159200 | 81161599 | high | 13.33 | 0.000119 |
| ABCA1 | 19 | chr9 | 104903800 | 104913599 | high | 54.44 | 0.000181 |
| ABCA10 | 10349 | chr17 | 69244600 | 69247399 | high | 15.55 | 0.000149 |
| ABCA4 | 24 | chr1 | 94112200 | 94116399 | high | 23.33 | 0.002550 |
| ABCA4 | 24 | chr1 | 94048000 | 94052399 | high | 24.44 | 0.000255 |
| ABCA4 | 24 | chr1 | 94002400 | 94008399 | high | 33.33 | 0.002553 |
| ABCA4 | 24 | chr1 | 94038400 | 94045799 | high | 41.11 | 0.000255 |
| ABCA5 | 23461 | chr17 | 69244600 | 69247399 | high | 15.55 | 0.000339 |
| ABCA7 | 10347 | chr19 | 1060000 | 1065999 | high | 33.33 | 0.009761 |
| ABCA8 | 10351 | chr17 | 68940800 | 68945599 | high | 26.66 | 0.000150 |
| ABCA8 | 10351 | chr17 | 68951200 | 68957799 | high | 36.66 | 0.000150 |
| ABCA9 | 10350 | chr17 | 69025000 | 69026999 | low | -4.00 | 0.000150 |
| ABCB10 | 23456 | chr1 | 229546800 | 229549799 | low | -6.00 | 0.000102 |
| ABCB9 | 23457 | chr12 | 122924200 | 122929399 | high | 28.88 | 0.000191 |
| ABCC10 | 89845 | chr6 | 43426000 | 43429399 | high | 18.88 | 0.002069 |
| ABCC2 | 1244 | chr10 | 99842400 | 99848999 | high | 36.66 | 0.000012 |
| ABCC3 | 8714 | chr17 | 50671000 | 50673799 | high | 15.55 | 0.000172 |
| ABCD3 | 5825 | chr1 | 94471200 | 94475199 | high | 22.22 | 0.000062 |
| ABCD3 | 5825 | chr1 | 94513200 | 94522999 | high | 54.44 | 0.000062 |
| ABCE1 | 6059 | chr4 | 145097000 | 145099399 | low | -4.80 | 0.000042 |
| ABCG2 | 9429 | chr4 | 88156400 | 88159399 | high | 16.66 | 0.000107 |
| ABCG2 | 9429 | chr4 | 88135200 | 88138599 | high | 18.88 | 0.000107 |
| ABCG2 | 9429 | chr4 | 88220600 | 88225199 | high | 25.55 | 0.000107 |
| ABHD10 | 55347 | chr3 | 111988000 | 111989999 | high | 11.11 | 0.000494 |
| ABHD12 | 26090 | chr20 | 25314000 | 25318199 | high | 23.33 | 0.001031 |
| ABHD12 | 26090 | chr20 | 25367200 | 25373399 | high | 34.44 | 0.001028 |
| ABHD13 | 84945 | chr13 | 108218000 | 108219399 | high | 7.77 | 0.000785 |
| ABHD2 | 11057 | chr15 | 89138000 | 89141199 | high | 17.77 | 0.000124 |
| ABHD2 | 11057 | chr15 | 89130400 | 89134399 | high | 22.22 | 0.000124 |
| ABHD2 | 11057 | chr15 | 89110800 | 89115199 | high | 24.44 | 0.000124 |
| ABHD2 | 11057 | chr15 | 89142000 | 89146599 | high | 25.55 | 0.000124 |
| ABHD2 | 11057 | chr15 | 89101000 | 89105599 | high | 25.55 | 0.000124 |
| ABHD3 | 171586 | chr18 | 21669400 | 21673199 | high | 21.11 | 0.007918 |
| ABHD4 | 63874 | chr14 | 22596800 | 22600999 | high | 23.33 | 0.002827 |
| ABHD5 | 51099 | chr3 | 43688200 | 43693999 | high | 32.22 | 0.001170 |
| ABHD6 | 57406 | chr3 | 58237000 | 58239199 | high | 12.22 | 0.000986 |
| ABI1 | 10006 | chr10 | 26750400 | 26753799 | low | -6.80 | 0.000374 |
| ABI3BP | 25890 | chr3 | 100751400 | 100755999 | high | 25.55 | 0.000257 |
| ABL1 | 25 | chr9 | 130712400 | 130715599 | high | 17.77 | 0.019126 |
| ABL1 | 25 | chr9 | 130763000 | 130768199 | high | 28.88 | 0.001912 |
| ABL1 | 25 | chr9 | 130853600 | 130866399 | high | 71.11 | 0.000191 |
| ABLIM3 | 22885 | chr5 | 149178800 | 149181199 | high | 13.33 | 0.000153 |
| ABLIM3 | 22885 | chr5 | 149162400 | 149165199 | high | 15.55 | 0.000153 |
| ABLIM3 | 22885 | chr5 | 149166000 | 149172199 | high | 34.44 | 0.000153 |
| ABTB1 | 80325 | chr3 | 127670400 | 127675199 | high | 26.66 | 0.000629 |
| ABTB2 | 25841 | chr11 | 34229800 | 34232599 | high | 15.55 | 0.000755 |
| ABTB2 | 25841 | chr11 | 34343800 | 34347399 | high | 19.99 | 0.000752 |
| ABTB2 | 25841 | chr11 | 34348600 | 34357399 | high | 48.88 | 0.000752 |
| ACAA1 | 30 | chr3 | 38135200 | 38136999 | low | -3.60 | 0.007867 |
| ACAD10 | 80724 | chr12 | 111726600 | 111733399 | high | 37.77 | 0.000723 |
| ACAD9 | 28976 | chr3 | 128878400 | 128880599 | high | 12.22 | 0.000225 |
| ACADVL | 37 | chr17 | 7214000 | 7221399 | high | 41.11 | 0.000005 |
| ACAP1 | 9744 | chr17 | 7351000 | 7353599 | low | -5.20 | 0.001326 |
| ACAP3 | 116983 | chr1 | 1307600 | 1310199 | low | -5.20 | 0.009940 |
| ACAT2 | 39 | chr6 | 159761200 | 159763599 | high | 13.33 | 0.002441 |
| ACBD3 | 64746 | chr1 | 226184600 | 226187999 | high | 18.88 | 0.000286 |
| ACBD4 | 79777 | chr17 | 45127200 | 45132999 | high | 32.22 | 0.001768 |
| ACBD5 | 91452 | chr10 | 27240600 | 27243199 | high | 14.44 | 0.003357 |
| ACD | 65057 | chr16 | 67653800 | 67660799 | high | 38.88 | 0.000962 |
| ACE | 1636 | chr17 | 63483800 | 63489599 | high | 32.22 | 0.000026 |
| ACER3 | 55331 | chr11 | 76990200 | 76994399 | high | 23.33 | 0.000719 |
| ACIN1 | 22985 | chr14 | 23052800 | 23058999 | high | 34.44 | 0.000997 |
| ACLY | 47 | chr17 | 41915000 | 41920399 | high | 29.99 | 0.000001 |
| ACO1 | 48 | chr9 | 32426200 | 32428599 | high | 13.33 | 0.000001 |
| ACO2 | 50 | chr22 | 41466800 | 41469799 | high | 16.66 | 0.000001 |
| ACO2 | 50 | chr22 | 41487000 | 41493799 | high | 37.77 | 0.000001 |

| | | | | | | | |
|---|---|---|---|---|---|---|---|
| ACO2 | 50 | chr22 | 41528400 | 41535399 | high | 38.88 | 0.000001 |
| ACOT4 | 122970 | chr14 | 73591400 | 73594399 | high | 16.66 | 0.001671 |
| ACOX2 | 8309 | chr3 | 58511400 | 58514199 | high | 15.55 | 0.000142 |
| ACOXL | 55289 | chr2 | 111097200 | 111100599 | high | 18.88 | 0.000498 |
| ACOXL | 55289 | chr2 | 110736400 | 110741599 | high | 28.88 | 0.000499 |
| ACSL3 | 2181 | chr2 | 222859600 | 222865399 | high | 32.22 | 0.000010 |
| ACSL4 | 2182 | chrX | 109732200 | 109733599 | high | 7.77 | 0.000020 |
| ACSS2 | 55902 | chr20 | 34872800 | 34877399 | high | 25.55 | 0.001603 |
| ACSS2 | 55902 | chr20 | 34918400 | 34923199 | high | 26.66 | 0.001601 |
| ACTB | 60 | chr7 | 5528600 | 5531399 | high | 15.55 | 0.000011 |
| ACTG1 | 71 | chr17 | 81508400 | 81513799 | low | -10.80 | 0.000871 |
| ACTG2 | 72 | chr2 | 73893400 | 73897599 | high | 23.33 | 0.000001 |
| ACTG2 | 72 | chr2 | 73910800 | 73915799 | high | 27.77 | 0.000001 |
| ACTN1 | 87 | chr14 | 68889200 | 68893399 | high | 23.33 | 0.000001 |
| ACTN1 | 87 | chr14 | 68945400 | 68949999 | high | 25.55 | 0.000001 |
| ACTN4 | 81 | chr19 | 38695600 | 38698799 | high | 17.77 | 0.000002 |
| ACTN4 | 81 | chr19 | 38704400 | 38708599 | high | 23.33 | 0.000002 |
| ACTR10 | 55860 | chr14 | 58200000 | 58201399 | low | -2.80 | 0.000960 |
| ACTR1B | 10120 | chr2 | 97661600 | 97663599 | low | -4.00 | 0.000104 |
| ACTR2 | 10097 | chr2 | 65264800 | 65269999 | high | 28.88 | 0.000155 |
| ACVR1 | 90 | chr2 | 157747600 | 157750399 | low | -5.60 | 0.000571 |
| ACVRL1 | 94 | chr12 | 51910000 | 51913599 | high | 19.99 | 0.000002 |
| ACYP1 | 97 | chr14 | 75059000 | 75064399 | high | 29.99 | 0.000001 |
| ACYP2 | 98 | chr2 | 54237400 | 54242999 | low | -11.20 | 0.000002 |
| ACYP2 | 98 | chr2 | 54300800 | 54302999 | low | -4.40 | 0.000002 |
| ADAL | 161823 | chr15 | 43329000 | 43332599 | low | -7.20 | 0.003735 |
| ADAP2 | 55803 | chr17 | 30956400 | 30959199 | high | 15.55 | 0.001803 |
| ADARB1 | 104 | chr21 | 45163800 | 45168199 | high | 24.44 | 0.000002 |
| ADAT1 | 23536 | chr16 | 75621600 | 75623199 | low | -3.20 | 0.000311 |
| ADCK5 | 203054 | chr8 | 144373000 | 144375599 | high | 14.44 | 0.001406 |
| ADCY9 | 115 | chr16 | 4077200 | 4081399 | high | 23.33 | 0.000028 |
| ADD1 | 118 | chr4 | 2881200 | 2884999 | high | 21.11 | 0.000041 |
| ADD3 | 120 | chr10 | 110068200 | 110071599 | high | 18.88 | 0.000001 |
| ADD3 | 120 | chr10 | 110104200 | 110107799 | high | 19.99 | 0.000001 |
| ADH5 | 128 | chr4 | 99088000 | 99089999 | low | -4.00 | 0.000001 |
| ADI1 | 55256 | chr2 | 3518200 | 3519799 | low | -3.20 | 0.015706 |
| ADIPOR1 | 51094 | chr1 | 202957400 | 202958399 | low | -2.00 | 0.000252 |
| ADIPOR2 | 79602 | chr12 | 1690400 | 1693199 | low | -5.60 | 0.005232 |
| ADIPOR2 | 79602 | chr12 | 1781200 | 1783399 | low | -4.40 | 0.004966 |
| ADIPOR2 | 79602 | chr12 | 1707000 | 1708399 | low | -2.80 | 0.005181 |
| ADIRF | 10974 | chr10 | 86970000 | 86973199 | high | 17.77 | 0.000126 |
| ADK | 132 | chr10 | 74371400 | 74373199 | high | 9.99 | 0.000002 |
| ADK | 132 | chr10 | 74426600 | 74429999 | high | 18.88 | 0.000002 |
| ADK | 132 | chr10 | 74388400 | 74391999 | high | 19.99 | 0.000002 |
| ADK | 132 | chr10 | 74396200 | 74400399 | high | 23.33 | 0.000002 |
| ADK | 132 | chr10 | 74381600 | 74386399 | high | 26.66 | 0.000002 |
| ADK | 132 | chr10 | 74254200 | 74261599 | high | 41.11 | 0.000002 |
| ADK | 132 | chr10 | 74201600 | 74210999 | high | 52.22 | 0.000002 |
| ADM | 133 | chr11 | 10304200 | 10306199 | low | -4.00 | 0.000013 |
| ADM5 | 199800 | chr19 | 49690000 | 49694199 | high | 23.33 | 0.004021 |
| ADNP | 23394 | chr20 | 50889000 | 50891399 | high | 13.33 | 0.000460 |
| ADNP | 23394 | chr20 | 50928400 | 50933799 | high | 29.99 | 0.000459 |
| ADNP | 23394 | chr20 | 50914400 | 50920799 | high | 35.55 | 0.000459 |
| ADNP2 | 22850 | chr18 | 80117000 | 80119399 | low | -4.80 | 0.000285 |
| ADPGK | 83440 | chr15 | 72780600 | 72784599 | high | 16.00 | 0.001146 |
| ADRA1B | 147 | chr5 | 159927400 | 159929999 | low | -5.20 | 0.000001 |
| ADRA1D | 146 | chr20 | 4230600 | 4234799 | high | 16.80 | 0.000035 |
| AEBP2 | 121536 | chr12 | 19471600 | 19476799 | high | 20.80 | 0.006242 |
| AEBP2 | 121536 | chr12 | 19436600 | 19443399 | high | 27.20 | 0.006253 |
| AES | 166 | chr19 | 3061600 | 3062999 | low | -2.80 | 0.000054 |
| AFAP1 | 60312 | chr4 | 7865200 | 7868199 | high | 12.00 | 0.007668 |
| AFAP1 | 60312 | chr4 | 7850200 | 7854199 | high | 16.00 | 0.007683 |
| AFF1 | 4299 | chr4 | 87057800 | 87058799 | high | 4.00 | 0.000049 |
| AFF1 | 4299 | chr4 | 87022000 | 87031399 | high | 37.60 | 0.000049 |
| AFTPH | 54812 | chr2 | 64584200 | 64586399 | high | 8.80 | 0.000849 |
| AGAP1 | 116987 | chr2 | 235719400 | 235721599 | low | -4.40 | 0.000496 |
| AGAP11 | 119385 | chr10 | 86970000 | 86973199 | high | 12.80 | 0.001373 |
| AGAP2 | 116986 | chr12 | 57738800 | 57741799 | high | 12.00 | 0.002026 |
| AGAP3 | 116988 | chr7 | 151085600 | 151088799 | low | -6.40 | 0.000774 |
| AGBL3 | 340351 | chr7 | 134989000 | 134989999 | low | -2.00 | 0.002521 |
| AGBL5 | 60509 | chr2 | 27070200 | 27072399 | high | 8.80 | 0.002235 |
| AGFG2 | 3268 | chr7 | 100543000 | 100546599 | high | 14.40 | 0.000033 |
| AGFG2 | 3268 | chr7 | 100550000 | 100554999 | high | 20.00 | 0.000033 |
| AGL | 178 | chr1 | 99919800 | 99924799 | high | 20.00 | 0.000002 |
| AGL | 178 | chr1 | 99869600 | 99875399 | high | 23.20 | 0.000002 |
| AGMO | 392636 | chr7 | 15302000 | 15303599 | high | 6.40 | 0.002851 |
| AGO4 | 192670 | chr1 | 35808600 | 35810799 | low | -4.40 | 0.005381 |
| AGPAT2 | 10555 | chr9 | 136683600 | 136687199 | high | 14.40 | 0.000077 |

| | | | | | | | |
|---|---|---|---|---|---|---|---|
| AGPAT3 | 56894 | chr21 | 43865600 | 43870399 | high | 19.20 | 0.001297 |
| AGPAT4 | 56895 | chr6 | 161227400 | 161234199 | high | 27.20 | 0.000353 |
| AGPAT5 | 55326 | chr8 | 6707800 | 6710599 | high | 11.20 | 0.008248 |
| AGPS | 8540 | chr2 | 177452000 | 177454399 | low | -4.80 | 0.000048 |
| AGPS | 8540 | chr2 | 177392600 | 177394599 | low | -4.00 | 0.000048 |
| AGRN | 375790 | chr1 | 1019200 | 1021399 | high | 8.80 | 0.000754 |
| AHCTF1 | 25909 | chr1 | 246928600 | 246931999 | high | 13.60 | 0.000105 |
| AHCY | 191 | chr20 | 34284600 | 34286399 | high | 7.20 | 0.000006 |
| AHCYL1 | 10768 | chr1 | 110002000 | 110003599 | high | 6.40 | 0.000098 |
| AHNAK | 79026 | chr11 | 62501600 | 62506399 | high | 19.20 | 0.001264 |
| AHNAK | 79026 | chr11 | 62443800 | 62450599 | high | 27.20 | 0.001266 |
| AHNAK2 | 113146 | chr14 | 104966000 | 104971399 | high | 21.60 | 0.001078 |
| AHR | 196 | chr7 | 17298800 | 17303599 | low | -9.60 | 0.000011 |
| AHR | 196 | chr7 | 17343600 | 17346599 | low | -6.00 | 0.000011 |
| AHRR | 57491 | chr5 | 321000 | 323399 | high | 9.60 | 0.008847 |
| AHRR | 57491 | chr5 | 413800 | 416799 | high | 12.00 | 0.000022 |
| AHRR | 57491 | chr5 | 335000 | 338599 | high | 14.40 | 0.004925 |
| AHRR | 57491 | chr5 | 419600 | 423399 | high | 15.20 | 0.001382 |
| AHRR | 57491 | chr5 | 339800 | 344399 | high | 18.40 | 0.018799 |
| AHRR | 57491 | chr5 | 313600 | 319999 | high | 25.60 | 0.001833 |
| AHRR | 57491 | chr5 | 433600 | 440399 | high | 27.20 | 0.001326 |
| AHSA1 | 10598 | chr14 | 77457000 | 77459399 | low | -4.80 | 0.000137 |
| AHSA2 | 130872 | chr2 | 61174000 | 61186599 | high | 50.40 | 0.002139 |
| AIDA | 64853 | chr1 | 222712000 | 222713999 | low | -4.00 | 0.000291 |
| AIFM3 | 150209 | chr22 | 20980000 | 20982599 | low | -5.20 | 0.007160 |
| AIG1 | 51390 | chr6 | 143252400 | 143255399 | low | -6.00 | 0.000359 |
| AIG1 | 51390 | chr6 | 143063800 | 143065399 | low | -3.20 | 0.000359 |
| AIM1 | 202 | chr6 | 106525800 | 106529199 | low | -6.80 | 0.000002 |
| AIM1 | 202 | chr6 | 106511400 | 106513999 | low | -5.20 | 0.000002 |
| AIMP1 | 9255 | chr4 | 106315000 | 106318199 | low | -6.40 | 0.000087 |
| AIMP2 | 7965 | chr7 | 6007800 | 6013399 | high | 22.40 | 0.001326 |
| AIP | 9049 | chr11 | 67482200 | 67485199 | high | 12.00 | 0.000134 |
| AK1 | 203 | chr9 | 127868800 | 127874399 | high | 22.40 | 0.000002 |
| AK2 | 204 | chr1 | 33013000 | 33016199 | low | -6.40 | 0.000006 |
| AK4 | 205 | chr1 | 65213400 | 65216999 | high | 14.40 | 0.000003 |
| AK5 | 26289 | chr1 | 77508400 | 77510199 | high | 7.20 | 0.000339 |
| AK5 | 26289 | chr1 | 77534000 | 77536799 | high | 11.20 | 0.000339 |
| AK5 | 26289 | chr1 | 77462800 | 77466199 | high | 13.60 | 0.000339 |
| AK5 | 26289 | chr1 | 77379200 | 77382799 | high | 14.40 | 0.000340 |
| AK5 | 26289 | chr1 | 77468400 | 77472399 | high | 16.00 | 0.000339 |
| AK5 | 26289 | chr1 | 77281600 | 77285799 | high | 16.80 | 0.000340 |
| AK5 | 26289 | chr1 | 77292800 | 77297199 | high | 17.60 | 0.000340 |
| AK5 | 26289 | chr1 | 77371000 | 77375599 | high | 18.40 | 0.000340 |
| AK5 | 26289 | chr1 | 77356400 | 77360999 | high | 18.40 | 0.000340 |
| AK5 | 26289 | chr1 | 77539600 | 77544399 | high | 19.20 | 0.000339 |
| AK5 | 26289 | chr1 | 77321200 | 77326399 | high | 20.80 | 0.000340 |
| AK5 | 26289 | chr1 | 77312000 | 77317199 | high | 20.80 | 0.000340 |
| AK5 | 26289 | chr1 | 77386000 | 77391799 | high | 23.20 | 0.000340 |
| AKAP10 | 11216 | chr17 | 19928000 | 19930199 | low | -4.40 | 0.000563 |
| AKAP11 | 11215 | chr13 | 42319800 | 42324399 | high | 18.40 | 0.000265 |
| AKAP11 | 11215 | chr13 | 42298800 | 42303599 | high | 19.20 | 0.000265 |
| AKAP11 | 11215 | chr13 | 42305200 | 42310399 | high | 20.80 | 0.000265 |
| AKAP11 | 11215 | chr13 | 42280000 | 42285199 | high | 20.80 | 0.000265 |
| AKAP11 | 11215 | chr13 | 42269800 | 42275199 | high | 21.60 | 0.000265 |
| AKAP11 | 11215 | chr13 | 42289800 | 42297599 | high | 31.20 | 0.000265 |
| AKAP12 | 9590 | chr6 | 151322200 | 151327799 | high | 22.40 | 0.000063 |
| AKAP12 | 9590 | chr6 | 151288200 | 151293799 | high | 22.40 | 0.000063 |
| AKAP13 | 11214 | chr15 | 85450000 | 85453399 | high | 13.60 | 0.000131 |
| AKAP13 | 11214 | chr15 | 85518600 | 85522799 | high | 16.80 | 0.000131 |
| AKAP13 | 11214 | chr15 | 85492200 | 85496599 | high | 17.60 | 0.000131 |
| AKAP13 | 11214 | chr15 | 85621000 | 85625599 | high | 18.40 | 0.000131 |
| AKAP13 | 11214 | chr15 | 85525800 | 85530599 | high | 19.20 | 0.000131 |
| AKAP13 | 11214 | chr15 | 85598800 | 85603799 | high | 20.00 | 0.000131 |
| AKAP13 | 11214 | chr15 | 85688200 | 85693999 | high | 23.20 | 0.000131 |
| AKAP13 | 11214 | chr15 | 85716800 | 85723799 | high | 28.00 | 0.000131 |
| AKAP13 | 11214 | chr15 | 85474200 | 85481199 | high | 28.00 | 0.000131 |
| AKAP13 | 11214 | chr15 | 85694800 | 85704599 | high | 39.20 | 0.000131 |
| AKAP2 | 11217 | chr9 | 110155200 | 110158399 | high | 12.80 | 0.000102 |
| AKAP2 | 11217 | chr9 | 110163800 | 110169399 | high | 22.40 | 0.000102 |
| AKAP2 | 11217 | chr9 | 110133200 | 110138799 | high | 22.40 | 0.000102 |
| AKAP5 | 9495 | chr14 | 64467400 | 64470199 | low | -5.60 | 0.000147 |
| AKAP8 | 10270 | chr19 | 15376400 | 15381599 | high | 20.80 | 0.000668 |
| AKAP8L | 26993 | chr19 | 15376400 | 15381599 | high | 20.80 | 0.001755 |
| AKAP9 | 10142 | chr7 | 91961200 | 91966599 | high | 21.60 | 0.000110 |
| AKIP1 | 56672 | chr11 | 8910000 | 8912799 | high | 11.20 | 0.006360 |
| AKIRIN2 | 55122 | chr6 | 87695400 | 87698999 | high | 14.40 | 0.000629 |
| AKR1C3 | 8644 | chr10 | 5087800 | 5090799 | high | 12.00 | 0.001699 |
| AKR1C3 | 8644 | chr10 | 5065600 | 5069199 | high | 14.40 | 0.001706 |

| | | | | | | | |
|---|---|---|---|---|---|---|---|
| AKR1C6P | 389932 | chr10 | 4909000 | 4910999 | low | -8.69 | 0.008826 |
| AKR1C6P | 389932 | chr10 | 4887000 | 4888599 | low | -6.95 | 0.008866 |
| AKT1 | 207 | chr14 | 104793200 | 104795999 | high | 11.20 | 0.000002 |
| AKT1S1 | 84335 | chr19 | 49868200 | 49870799 | high | 10.40 | 0.001691 |
| AKT1S1 | 84335 | chr19 | 49873800 | 49879599 | high | 23.20 | 0.001691 |
| AKT2 | 208 | chr19 | 40282000 | 40285199 | low | -13.91 | 0.000005 |
| ALAD | 210 | chr9 | 113398600 | 113402999 | high | 17.60 | 0.000002 |
| ALCAM | 214 | chr3 | 105429400 | 105432799 | high | 13.60 | 0.000002 |
| ALCAM | 214 | chr3 | 105409800 | 105413199 | high | 13.60 | 0.000002 |
| ALDH1L2 | 160428 | chr12 | 105081200 | 105085799 | high | 18.40 | 0.001527 |
| ALDH3B1 | 221 | chr11 | 68029200 | 68032399 | high | 12.80 | 0.000003 |
| ALDH3B1 | 221 | chr11 | 68012000 | 68015799 | high | 15.20 | 0.000003 |
| ALDH6A1 | 4329 | chr14 | 74083000 | 74087199 | high | 16.80 | 0.000058 |
| ALDOC | 230 | chr17 | 28568400 | 28574399 | high | 24.00 | 0.000008 |
| ALG14 | 199857 | chr1 | 95037000 | 95039399 | high | 9.60 | 0.002103 |
| ALG14 | 199857 | chr1 | 95071000 | 95073999 | high | 12.00 | 0.002102 |
| ALG14 | 199857 | chr1 | 94987200 | 94992199 | high | 20.00 | 0.002104 |
| ALG2 | 85365 | chr9 | 99219600 | 99222799 | high | 12.80 | 0.000860 |
| ALG5 | 29880 | chr13 | 36997600 | 36999999 | high | 9.60 | 0.000808 |
| ALG9 | 79796 | chr11 | 111843600 | 111847599 | high | 16.00 | 0.000713 |
| ALG9 | 79796 | chr11 | 111834400 | 111839199 | high | 19.20 | 0.000714 |
| ALKBH1 | 8846 | chr14 | 77706400 | 77711199 | high | 19.20 | 0.000114 |
| ALOX5AP | 241 | chr13 | 30712000 | 30717599 | high | 22.40 | 0.000008 |
| ALPK1 | 80216 | chr4 | 112297200 | 112301999 | high | 19.20 | 0.000714 |
| ALPK2 | 115701 | chr18 | 58541000 | 58545999 | high | 20.00 | 0.001976 |
| ALS2 | 57679 | chr2 | 201709400 | 201712999 | high | 14.40 | 0.000286 |
| ALS2 | 57679 | chr2 | 201730200 | 201734799 | high | 18.40 | 0.000286 |
| ALS2CR12 | 130540 | chr2 | 201287800 | 201290799 | low | -13.04 | 0.000649 |
| ALS2CR12 | 130540 | chr2 | 201336000 | 201337799 | low | -7.82 | 0.000648 |
| AMBRA1 | 55626 | chr11 | 46564600 | 46566799 | low | -9.56 | 0.001195 |
| AMD1 | 262 | chr6 | 110811400 | 110816999 | high | 22.40 | 0.000002 |
| AMIGO2 | 347902 | chr12 | 47079000 | 47083599 | high | 18.40 | 0.007390 |
| AMMECR1 | 9949 | chrX | 110315400 | 110319599 | high | 16.80 | 0.000090 |
| AMN1 | 196394 | chr12 | 31728200 | 31729799 | low | -6.95 | 0.006190 |
| AMPD2 | 271 | chr1 | 109628000 | 109631199 | high | 12.80 | 0.000002 |
| AMPD3 | 272 | chr11 | 10451400 | 10454199 | high | 11.20 | 0.000026 |
| AMPD3 | 272 | chr11 | 10479800 | 10484199 | high | 17.60 | 0.000026 |
| AMPD3 | 272 | chr11 | 10488400 | 10495999 | high | 30.40 | 0.000026 |
| ANK1 | 286 | chr8 | 41696800 | 41700199 | high | 13.60 | 0.000007 |
| ANK2 | 287 | chr4 | 113360600 | 113366199 | high | 22.40 | 0.000003 |
| ANLN | 54443 | chr7 | 36407600 | 36413599 | high | 24.00 | 0.001495 |
| ANO10 | 55129 | chr3 | 43433200 | 43438999 | high | 23.20 | 0.001269 |
| ANO3 | 63982 | chr11 | 26638600 | 26641799 | low | -13.91 | 0.002402 |
| ANO6 | 196527 | chr12 | 45268600 | 45270599 | high | 8.00 | 0.004341 |
| ANO6 | 196527 | chr12 | 45232600 | 45235599 | high | 12.00 | 0.004345 |
| ANO6 | 196527 | chr12 | 45412400 | 45416399 | high | 16.00 | 0.004328 |
| ANO6 | 196527 | chr12 | 45222800 | 45226799 | high | 16.00 | 0.004346 |
| ANO6 | 196527 | chr12 | 45258600 | 45262999 | high | 17.60 | 0.004342 |
| ANO6 | 196527 | chr12 | 45245000 | 45251399 | high | 25.60 | 0.004344 |
| ANO6 | 196527 | chr12 | 45215000 | 45221599 | high | 26.40 | 0.004347 |
| ANO7 | 50636 | chr2 | 241217800 | 241220399 | low | -11.30 | 0.000210 |
| ANP32A | 8125 | chr15 | 68817800 | 68821199 | low | -14.78 | 0.000118 |
| ANP32B | 10541 | chr9 | 97981600 | 97985599 | high | 16.00 | 0.000108 |
| ANPEP | 290 | chr15 | 89812600 | 89815999 | high | 13.60 | 0.000003 |
| ANPEP | 290 | chr15 | 89805400 | 89811199 | high | 23.20 | 0.000003 |
| AOC2 | 314 | chr17 | 42838200 | 42844799 | high | 26.40 | 0.007330 |
| AOX1 | 316 | chr2 | 200585400 | 200587999 | high | 10.40 | 0.000002 |
| AOX1 | 316 | chr2 | 200609000 | 200611999 | high | 12.00 | 0.000002 |
| AOX1 | 316 | chr2 | 200601200 | 200606199 | high | 20.00 | 0.000002 |
| AP1AR | 55435 | chr4 | 112241800 | 112245399 | low | -15.65 | 0.000494 |
| AP1M1 | 8907 | chr19 | 16196200 | 16201199 | high | 20.00 | 0.000550 |
| AP1S1 | 1174 | chr7 | 101154400 | 101157199 | low | -12.17 | 0.000012 |
| AP1S3 | 130340 | chr2 | 223835800 | 223838999 | high | 12.80 | 0.000582 |
| AP2A1 | 160 | chr19 | 49769400 | 49773599 | high | 16.80 | 0.000003 |
| AP2A1 | 160 | chr19 | 49802200 | 49806999 | high | 19.20 | 0.000003 |
| AP2S1 | 1175 | chr19 | 46849400 | 46851399 | high | 8.00 | 0.000025 |
| AP3B1 | 8546 | chr5 | 78198400 | 78201199 | high | 11.20 | 0.000109 |
| AP3B1 | 8546 | chr5 | 78029600 | 78033999 | high | 17.60 | 0.000110 |
| AP3B1 | 8546 | chr5 | 78282800 | 78287999 | high | 20.80 | 0.000109 |
| AP3B1 | 8546 | chr5 | 78263800 | 78268999 | high | 20.80 | 0.000109 |
| AP3B1 | 8546 | chr5 | 78273000 | 78279799 | high | 27.20 | 0.000109 |
| AP3B1 | 8546 | chr5 | 78061400 | 78068599 | high | 28.80 | 0.000109 |
| AP3B1 | 8546 | chr5 | 78005000 | 78012199 | high | 28.80 | 0.000110 |
| AP3B1 | 8546 | chr5 | 78127800 | 78136399 | high | 34.40 | 0.000109 |
| AP3M2 | 10947 | chr8 | 42152200 | 42158999 | high | 27.20 | 0.000260 |
| AP3S1 | 1176 | chr5 | 115899400 | 115900999 | low | -6.95 | 0.000010 |
| AP4E1 | 23431 | chr15 | 50992000 | 50995399 | high | 13.60 | 0.000460 |
| AP4M1 | 9179 | chr7 | 100099200 | 100104199 | high | 20.00 | 0.000092 |

| | | | | | | | |
|---|---|---|---|---|---|---|---|
| AP4S1 | 11154 | chr14 | 31091000 | 31094399 | high | 13.60 | 0.000359 |
| AP4S1 | 11154 | chr14 | 31059400 | 31063999 | high | 18.40 | 0.000359 |
| APAF1 | 317 | chr12 | 98643600 | 98646999 | high | 13.60 | 0.000003 |
| APAF1 | 317 | chr12 | 98675200 | 98680999 | high | 23.20 | 0.000003 |
| APBA3 | 9546 | chr19 | 3759200 | 3763199 | high | 16.00 | 0.002539 |
| APBB2 | 323 | chr4 | 41068000 | 41070199 | high | 8.80 | 0.000008 |
| APBB2 | 323 | chr4 | 41212400 | 41215599 | high | 12.80 | 0.000008 |
| APBB2 | 323 | chr4 | 41058600 | 41063599 | high | 20.00 | 0.000008 |
| APBB2 | 323 | chr4 | 41037600 | 41044599 | high | 28.00 | 0.000008 |
| APCDD1 | 147495 | chr18 | 10467400 | 10469199 | low | -7.82 | 0.014091 |
| APEH | 327 | chr3 | 49681800 | 49682999 | low | -5.21 | 0.000007 |
| APH1B | 83464 | chr15 | 63291800 | 63295999 | high | 16.80 | 0.001319 |
| APIP | 51074 | chr11 | 34897800 | 34904399 | high | 26.40 | 0.001464 |
| APOA5 | 116519 | chr11 | 116781600 | 116792399 | high | 43.20 | 0.000998 |
| APOL4 | 80832 | chr22 | 36194000 | 36200799 | high | 27.20 | 0.002233 |
| APOL6 | 80830 | chr22 | 35648800 | 35651399 | high | 10.40 | 0.002267 |
| APOL6 | 80830 | chr22 | 35658600 | 35661999 | high | 13.60 | 0.002267 |
| APOLD1 | 81575 | chr12 | 12786200 | 12790799 | high | 18.40 | 0.006380 |
| APP | 351 | chr21 | 25910400 | 25913799 | high | 13.60 | 0.000014 |
| APP | 351 | chr21 | 25966400 | 25970399 | high | 16.00 | 0.000014 |
| APP | 351 | chr21 | 25993800 | 25997999 | high | 16.80 | 0.000014 |
| APP | 351 | chr21 | 25944000 | 25949399 | high | 21.60 | 0.000014 |
| APP | 351 | chr21 | 26037200 | 26044199 | high | 28.00 | 0.000013 |
| APPBP2 | 10513 | chr17 | 60461000 | 60464199 | high | 12.80 | 0.000174 |
| APPBP2 | 10513 | chr17 | 60456800 | 60460199 | high | 13.60 | 0.000174 |
| APPBP2 | 10513 | chr17 | 60439000 | 60447599 | high | 34.40 | 0.000174 |
| APPL2 | 55198 | chr12 | 105196200 | 105202599 | high | 25.60 | 0.000525 |
| APTR | 100505854 | chr7 | 77687800 | 77690799 | low | -13.04 | 0.000131 |
| AQP10 | 89872 | chr1 | 154322600 | 154327799 | high | 20.80 | 0.000582 |
| AQP3 | 360 | chr9 | 33445000 | 33448399 | high | 13.60 | 0.000011 |
| AQR | 9716 | chr15 | 34909400 | 34913799 | high | 17.60 | 0.000278 |
| AQR | 9716 | chr15 | 34899200 | 34904799 | high | 22.40 | 0.000278 |
| ARAP1 | 116985 | chr11 | 72729000 | 72732799 | high | 15.20 | 0.001609 |
| ARAP1 | 116985 | chr11 | 72740600 | 72744799 | high | 16.80 | 0.001608 |
| ARAP1 | 116985 | chr11 | 72722000 | 72726999 | high | 20.00 | 0.001609 |
| ARAP3 | 64411 | chr5 | 141656000 | 141661599 | high | 22.40 | 0.000455 |
| ARAP3 | 64411 | chr5 | 141678000 | 141684199 | high | 24.80 | 0.000455 |
| ARCN1 | 372 | chr11 | 118589600 | 118595199 | high | 22.40 | 0.000003 |
| AREL1 | 9870 | chr14 | 74670600 | 74676399 | high | 23.20 | 0.000132 |
| ARF1 | 375 | chr1 | 228082200 | 228084799 | low | -11.30 | 0.000002 |
| ARF3 | 377 | chr12 | 48956800 | 48957999 | low | -5.21 | 0.000008 |
| ARF6 | 382 | chr14 | 49895400 | 49896799 | low | -6.08 | 0.000008 |
| ARID1A | 8289 | chr1 | 26774600 | 26776799 | high | 8.80 | 0.000310 |
| ARID1A | 8289 | chr1 | 26771200 | 26773799 | high | 10.40 | 0.000310 |
| ARID1A | 8289 | chr1 | 26720400 | 26724199 | high | 15.20 | 0.000310 |
| ARID1A | 8289 | chr1 | 26743200 | 26747399 | high | 16.80 | 0.000310 |
| ARID1A | 8289 | chr1 | 26695800 | 26700599 | high | 19.20 | 0.000310 |
| ARID1A | 8289 | chr1 | 26701400 | 26707599 | high | 24.80 | 0.000310 |
| ARID1B | 57492 | chr6 | 157170000 | 157173999 | high | 16.00 | 0.000366 |
| ARID1B | 57492 | chr6 | 157147800 | 157154199 | high | 25.60 | 0.000366 |
| ARID2 | 196528 | chr12 | 45879400 | 45882599 | low | -13.91 | 0.004284 |
| ARID3A | 1820 | chr19 | 925600 | 927599 | low | -8.69 | 0.001966 |
| ARID3B | 10620 | chr15 | 74576000 | 74580999 | high | 20.00 | 0.000142 |
| ARID4A | 5926 | chr14 | 58295400 | 58299799 | low | -19.12 | 0.000102 |
| ARID4B | 51742 | chr1 | 235222800 | 235225999 | low | -13.91 | 0.000220 |
| ARID4B | 51742 | chr1 | 235245600 | 235247599 | low | -8.69 | 0.000220 |
| ARIH1 | 25820 | chr15 | 72542000 | 72545999 | high | 16.00 | 0.000356 |
| ARIH1 | 25820 | chr15 | 72558000 | 72564399 | high | 25.60 | 0.000356 |
| ARIH2 | 10425 | chr3 | 48917400 | 48920199 | high | 11.20 | 0.000213 |
| ARL1 | 400 | chr12 | 101405800 | 101409799 | high | 16.00 | 0.000004 |
| ARL10 | 285598 | chr5 | 176365000 | 176367199 | low | -9.56 | 0.001619 |
| ARL13B | 200894 | chr3 | 93992200 | 93997199 | high | 20.00 | 0.002137 |
| ARL15 | 54622 | chr5 | 53947200 | 53949999 | high | 11.20 | 0.001013 |
| ARL15 | 54622 | chr5 | 53985800 | 53993599 | high | 31.20 | 0.001012 |
| ARL3 | 403 | chr10 | 102698600 | 102701399 | high | 11.20 | 0.000004 |
| ARL4A | 10124 | chr7 | 12686200 | 12688799 | low | -11.30 | 0.000798 |
| ARL4C | 10123 | chr2 | 234495600 | 234496999 | low | -6.08 | 0.000043 |
| ARL5B | 221079 | chr10 | 18657200 | 18661799 | high | 18.40 | 0.011850 |
| ARL6IP1 | 23204 | chr16 | 18799200 | 18802999 | high | 15.20 | 0.001234 |
| ARL6IP4 | 51329 | chr12 | 122980000 | 122983599 | high | 14.40 | 0.000417 |
| ARL8B | 55207 | chr3 | 5122000 | 5122799 | low | -3.47 | 0.010778 |
| ARL9 | 132946 | chr4 | 56514200 | 56515999 | low | -7.82 | 0.002352 |
| ARMC5 | 79798 | chr16 | 31458200 | 31461199 | low | -13.04 | 0.002537 |
| ARMC6 | 93436 | chr19 | 19048800 | 19053399 | high | 18.40 | 0.004905 |
| ARMC6 | 93436 | chr19 | 19043000 | 19047999 | high | 20.00 | 0.004907 |
| ARMC7 | 79637 | chr17 | 75109600 | 75110799 | low | -5.21 | 0.001060 |
| ARMC8 | 25852 | chr3 | 138218600 | 138221399 | high | 11.20 | 0.000187 |
| ARMC8 | 25852 | chr3 | 138274600 | 138279799 | high | 20.80 | 0.000187 |

| | | | | | | | |
|---|---|---|---|---|---|---|---|
| ARMT1 | 79624 | chr6 | 151451200 | 151454199 | low | -13.04 | 0.000526 |
| ARNT | 405 | chr1 | 150829600 | 150835199 | high | 22.40 | 0.000003 |
| ARNT | 405 | chr1 | 150872200 | 150878199 | high | 24.00 | 0.000003 |
| ARNT2 | 9915 | chr15 | 80583600 | 80588399 | low | -20.87 | 0.000123 |
| ARNT2 | 9915 | chr15 | 80484000 | 80490599 | high | 26.40 | 0.000123 |
| ARNTL2 | 56938 | chr12 | 27421800 | 27423799 | high | 8.00 | 0.002076 |
| ARNTL2 | 56938 | chr12 | 27395600 | 27399799 | high | 16.80 | 0.002078 |
| ARNTL2 | 56938 | chr12 | 27381600 | 27386799 | high | 20.80 | 0.002079 |
| ARNTL2 | 56938 | chr12 | 27354200 | 27365999 | high | 47.20 | 0.002082 |
| ARPC1A | 10552 | chr7 | 99344200 | 99349399 | low | -22.64 | 0.000106 |
| ARPC1A | 10552 | chr7 | 99324600 | 99329599 | high | 20.00 | 0.000106 |
| ARPC2 | 10109 | chr2 | 218216600 | 218218599 | low | -8.69 | 0.000046 |
| ARPC3 | 10094 | chr12 | 110441200 | 110445799 | high | 18.40 | 0.000091 |
| ARPC5 | 10092 | chr1 | 183628800 | 183631999 | high | 12.80 | 0.000055 |
| ARPP19 | 10776 | chr15 | 52567400 | 52571199 | high | 15.20 | 0.000205 |
| ARPP19 | 10776 | chr15 | 52551600 | 52557999 | high | 25.60 | 0.000205 |
| ARRDC3 | 57561 | chr5 | 91380600 | 91388799 | high | 32.80 | 0.000630 |
| ARSB | 411 | chr5 | 78875400 | 78879399 | high | 16.00 | 0.000005 |
| ARSG | 22901 | chr17 | 68316600 | 68320399 | high | 15.20 | 0.000335 |
| ARSG | 22901 | chr17 | 68296200 | 68300799 | high | 18.40 | 0.000335 |
| ARSG | 22901 | chr17 | 68289800 | 68295399 | high | 22.40 | 0.000335 |
| ARSG | 22901 | chr17 | 68303800 | 68311399 | high | 30.40 | 0.000335 |
| ARSJ | 79642 | chr4 | 113899400 | 113902599 | high | 12.80 | 0.000699 |
| ARSK | 153642 | chr5 | 95600400 | 95601799 | low | -6.08 | 0.001607 |
| ARVCF | 421 | chr22 | 19988200 | 19992199 | high | 16.00 | 0.000021 |
| ASAP1 | 50807 | chr8 | 130141600 | 130144599 | high | 12.00 | 0.000390 |
| ASAP1 | 50807 | chr8 | 130281400 | 130284599 | high | 12.80 | 0.000390 |
| ASAP1 | 50807 | chr8 | 130312600 | 130316199 | high | 14.40 | 0.000390 |
| ASAP1 | 50807 | chr8 | 130160000 | 130164199 | high | 16.80 | 0.000390 |
| ASAP1 | 50807 | chr8 | 130419000 | 130423799 | high | 19.20 | 0.000390 |
| ASAP1 | 50807 | chr8 | 130250600 | 130255599 | high | 20.00 | 0.000390 |
| ASAP1 | 50807 | chr8 | 130129000 | 130133999 | high | 20.00 | 0.000390 |
| ASAP1 | 50807 | chr8 | 130320400 | 130330399 | high | 40.00 | 0.000390 |
| ASAP2 | 8853 | chr2 | 9234000 | 9237799 | high | 15.20 | 0.000959 |
| ASAP2 | 8853 | chr2 | 9371400 | 9376999 | high | 22.40 | 0.000945 |
| ASAP3 | 55616 | chr1 | 23441000 | 23445199 | high | 16.80 | 0.002373 |
| ASAP3 | 55616 | chr1 | 23430800 | 23435199 | high | 17.60 | 0.002374 |
| ASAP3 | 55616 | chr1 | 23462200 | 23468799 | high | 26.40 | 0.002370 |
| ASB1 | 51665 | chr2 | 238427400 | 238433999 | high | 16.50 | 0.000217 |
| ASB13 | 79754 | chr10 | 5658600 | 5661999 | high | 8.50 | 0.014094 |
| ASB3 | 51130 | chr2 | 53776400 | 53778999 | high | 6.50 | 0.000951 |
| ASB5 | 140458 | chr4 | 176262600 | 176265999 | high | 8.50 | 0.000797 |
| ASB6 | 140459 | chr9 | 129641000 | 129642599 | high | 4.00 | 0.001083 |
| ASCC1 | 51008 | chr10 | 72168000 | 72171999 | high | 10.00 | 0.000707 |
| ASCC1 | 51008 | chr10 | 72131400 | 72136199 | high | 12.00 | 0.000707 |
| ASCC2 | 84164 | chr22 | 29794400 | 29798599 | high | 10.50 | 0.002825 |
| ASCC2 | 84164 | chr22 | 29799400 | 29805399 | high | 15.00 | 0.002824 |
| ASCC3 | 10973 | chr6 | 100594400 | 100597799 | low | -14.78 | 0.000109 |
| ASCC3 | 10973 | chr6 | 100553400 | 100556399 | low | -13.04 | 0.000109 |
| ASCC3 | 10973 | chr6 | 100532800 | 100534599 | low | -7.82 | 0.000109 |
| ASF1A | 25842 | chr6 | 118907600 | 118909799 | high | 5.50 | 0.000217 |
| ASF1B | 55723 | chr19 | 14114000 | 14120799 | high | 17.00 | 0.003948 |
| ASGR1 | 432 | chr17 | 7177400 | 7180399 | high | 7.50 | 0.000060 |
| ASH1L | 55870 | chr1 | 155462000 | 155465799 | high | 9.50 | 0.000359 |
| ASH1L | 55870 | chr1 | 155499000 | 155506399 | high | 18.50 | 0.000359 |
| ASH2L | 9070 | chr8 | 38104600 | 38108799 | high | 10.50 | 0.000238 |
| ASIC1 | 41 | chr12 | 50055000 | 50061199 | high | 15.50 | 0.008191 |
| ASIC1 | 41 | chr12 | 50078000 | 50086199 | high | 20.50 | 0.008187 |
| ASL | 435 | chr7 | 66075800 | 66077799 | low | -8.69 | 0.000007 |
| ASNA1 | 439 | chr19 | 12735800 | 12740199 | high | 11.00 | 0.000034 |
| ASNSD1 | 54529 | chr2 | 189660600 | 189663999 | low | -14.78 | 0.000288 |
| ASPH | 444 | chr8 | 61583800 | 61585799 | high | 5.00 | 0.000007 |
| ASPH | 444 | chr8 | 61564800 | 61567999 | high | 8.00 | 0.000007 |
| ASPH | 444 | chr8 | 61541400 | 61546599 | high | 13.00 | 0.000007 |
| ASPN | 54829 | chr9 | 92481400 | 92484799 | low | -14.78 | 0.000593 |
| ASPN | 54829 | chr9 | 92463200 | 92464999 | low | -7.82 | 0.000593 |
| ASPSCR1 | 79058 | chr17 | 81979000 | 81979999 | low | -4.34 | 0.000964 |
| ASTE1 | 28990 | chr3 | 131015200 | 131018799 | high | 9.00 | 0.000221 |
| ASTN2 | 23245 | chr9 | 116578000 | 116582999 | high | 12.50 | 0.000199 |
| ASUN | 55726 | chr12 | 26928000 | 26932599 | low | -19.91 | 0.002069 |
| ASXL1 | 171023 | chr20 | 32366600 | 32369399 | high | 7.00 | 0.005284 |
| ASXL2 | 55252 | chr2 | 25834800 | 25839199 | high | 11.00 | 0.002139 |
| ASXL2 | 55252 | chr2 | 25844200 | 25849599 | high | 13.50 | 0.002138 |
| ATAD1 | 84896 | chr10 | 87758400 | 87761399 | low | -13.04 | 0.000967 |
| ATAD2B | 54454 | chr2 | 23799600 | 23801999 | high | 6.00 | 0.002288 |
| ATAD2B | 54454 | chr2 | 23827400 | 23831399 | high | 10.00 | 0.002285 |
| ATAD3B | 83858 | chr1 | 1470000 | 1474799 | high | 12.00 | 0.004014 |
| ATAD3C | 219293 | chr1 | 1470000 | 1474799 | high | 12.00 | 0.005646 |

| | | | | | | | |
|---|---|---|---|---|---|---|---|
| ATAD5 | 79915 | chr17 | 30837200 | 30838999 | low | -7.82 | 0.002592 |
| ATE1 | 11101 | chr10 | 121927000 | 121928599 | low | -6.95 | 0.000091 |
| ATF1 | 466 | chr12 | 50815400 | 50818199 | low | -12.17 | 0.000009 |
| ATF3 | 467 | chr1 | 212606600 | 212609999 | high | 8.50 | 0.000002 |
| ATF5 | 22809 | chr19 | 49927400 | 49930799 | high | 8.50 | 0.000457 |
| ATF6 | 22926 | chr1 | 161790400 | 161793399 | high | 7.50 | 0.000142 |
| ATF6 | 22926 | chr1 | 161951400 | 161959199 | high | 19.50 | 0.000142 |
| ATF7 | 11016 | chr12 | 53508400 | 53512399 | high | 10.00 | 0.000206 |
| ATF7 | 11016 | chr12 | 53582600 | 53586999 | high | 11.00 | 0.000206 |
| ATF7 | 11016 | chr12 | 53565600 | 53570999 | high | 13.50 | 0.000206 |
| ATF7 | 11016 | chr12 | 53538000 | 53544199 | high | 15.50 | 0.000206 |
| ATG10 | 83734 | chr5 | 82249200 | 82252599 | high | 8.50 | 0.001018 |
| ATG10 | 83734 | chr5 | 82150400 | 82154399 | high | 10.00 | 0.001019 |
| ATG10 | 83734 | chr5 | 82178200 | 82182599 | high | 11.00 | 0.001019 |
| ATG13 | 9776 | chr11 | 46615800 | 46618599 | high | 7.00 | 0.000210 |
| ATG13 | 9776 | chr11 | 46673600 | 46677199 | high | 9.00 | 0.000209 |
| ATG13 | 9776 | chr11 | 46663400 | 46667199 | high | 9.50 | 0.000210 |
| ATG13 | 9776 | chr11 | 46637400 | 46641599 | high | 10.50 | 0.000210 |
| ATG14 | 22863 | chr14 | 55368800 | 55371399 | low | -11.30 | 0.000413 |
| ATG14 | 22863 | chr14 | 55401800 | 55403999 | low | -9.56 | 0.000413 |
| ATG4D | 84971 | chr19 | 10545400 | 10547399 | high | 5.00 | 0.008058 |
| ATG5 | 9474 | chr6 | 106312600 | 106317399 | high | 12.00 | 0.000089 |
| ATG5 | 9474 | chr6 | 106185600 | 106190799 | high | 13.00 | 0.000089 |
| ATG5 | 9474 | chr6 | 106320800 | 106326399 | high | 14.00 | 0.000089 |
| ATG5 | 9474 | chr6 | 106192000 | 106197999 | high | 15.00 | 0.000089 |
| ATG5 | 9474 | chr6 | 106280000 | 106286199 | high | 15.50 | 0.000089 |
| ATG7 | 10533 | chr3 | 11548000 | 11551399 | high | 8.50 | 0.000912 |
| ATG7 | 10533 | chr3 | 11457000 | 11460599 | high | 9.00 | 0.000919 |
| ATG7 | 10533 | chr3 | 11517800 | 11521599 | high | 9.50 | 0.000914 |
| ATG7 | 10533 | chr3 | 11556600 | 11562799 | high | 15.50 | 0.000911 |
| ATG7 | 10533 | chr3 | 11373600 | 11380199 | high | 16.50 | 0.000926 |
| ATL1 | 51062 | chr14 | 50601800 | 50605599 | high | 9.50 | 0.001009 |
| ATL1 | 51062 | chr14 | 50611600 | 50615799 | high | 10.50 | 0.001009 |
| ATL1 | 51062 | chr14 | 50556000 | 50561199 | high | 13.00 | 0.001010 |
| ATL1 | 51062 | chr14 | 50625200 | 50631199 | high | 15.00 | 0.001009 |
| ATL1 | 51062 | chr14 | 50540800 | 50550599 | high | 24.50 | 0.001010 |
| ATL2 | 64225 | chr2 | 38375000 | 38377799 | low | -12.17 | 0.001674 |
| ATL3 | 25923 | chr11 | 63668200 | 63673599 | high | 13.50 | 0.000407 |
| ATOH8 | 84913 | chr2 | 85776200 | 85782999 | high | 17.00 | 0.000990 |
| ATOX1 | 475 | chr5 | 151743600 | 151745799 | low | -9.56 | 0.000003 |
| ATOX1 | 475 | chr5 | 151757400 | 151759399 | low | -8.69 | 0.000003 |
| ATP10D | 57205 | chr4 | 47497000 | 47500999 | high | 10.00 | 0.001204 |
| ATP11B | 23200 | chr3 | 182866400 | 182868399 | low | -8.69 | 0.000127 |
| ATP2A2 | 488 | chr12 | 110349000 | 110351399 | high | 6.00 | 0.000004 |
| ATP2A2 | 488 | chr12 | 110303800 | 110306799 | high | 7.50 | 0.000004 |
| ATP2A2 | 488 | chr12 | 110340600 | 110346399 | high | 14.50 | 0.000004 |
| ATP2A3 | 489 | chr17 | 3941400 | 3945799 | high | 11.00 | 0.000124 |
| ATP4B | 496 | chr13 | 113653600 | 113657999 | high | 11.00 | 0.000004 |
| ATP5B | 506 | chr12 | 56631800 | 56638399 | low | -28.69 | 0.000009 |
| ATP5F1 | 515 | chr1 | 111452800 | 111455399 | low | -11.30 | 0.000005 |
| ATP5F1 | 515 | chr1 | 111448800 | 111449799 | low | -4.34 | 0.000005 |
| ATP5G2 | 517 | chr12 | 53673400 | 53677199 | low | -16.51 | 0.000010 |
| ATP5I | 521 | chr4 | 672000 | 675599 | high | 9.00 | 0.000775 |
| ATP5L | 10632 | chr11 | 118400800 | 118402599 | low | -7.82 | 0.000090 |
| ATP5S | 27109 | chr14 | 50321600 | 50324599 | low | -13.04 | 0.000539 |
| ATP8B1 | 5205 | chr18 | 57779000 | 57781999 | high | 7.50 | 0.000090 |
| ATP8B2 | 57198 | chr1 | 154322600 | 154327799 | high | 13.00 | 0.000371 |
| ATP8B3 | 148229 | chr19 | 1810600 | 1813399 | high | 7.00 | 0.009096 |
| ATP8B4 | 79895 | chr15 | 50039400 | 50042399 | high | 7.50 | 0.001597 |
| ATP8B4 | 79895 | chr15 | 50088000 | 50091599 | high | 9.00 | 0.001595 |
| ATP8B4 | 79895 | chr15 | 50034800 | 50038399 | high | 9.00 | 0.001597 |
| ATP8B4 | 79895 | chr15 | 49864000 | 49868199 | high | 10.50 | 0.001602 |
| ATP8B4 | 79895 | chr15 | 50001000 | 50005999 | high | 12.50 | 0.001598 |
| ATP8B4 | 79895 | chr15 | 49883600 | 49889799 | high | 15.50 | 0.001602 |
| ATP8B4 | 79895 | chr15 | 49990000 | 49996599 | high | 16.50 | 0.001598 |
| ATP8B4 | 79895 | chr15 | 49978800 | 49987199 | high | 21.00 | 0.001599 |
| ATP9A | 10079 | chr20 | 51631800 | 51636599 | high | 12.00 | 0.000195 |
| ATPAF1 | 64756 | chr1 | 46666600 | 46669599 | high | 7.50 | 0.001388 |
| ATPAF2 | 91647 | chr17 | 18037400 | 18042399 | high | 12.50 | 0.005081 |
| ATPIF1 | 93974 | chr1 | 28236000 | 28239999 | high | 10.00 | 0.003328 |
| ATR | 545 | chr3 | 142576800 | 142580799 | high | 10.00 | 0.000004 |
| ATR | 545 | chr3 | 142446200 | 142455999 | high | 24.50 | 0.000004 |
| ATRIP | 84126 | chr3 | 48446400 | 48448199 | low | -7.82 | 0.001736 |
| ATXN1 | 6310 | chr6 | 16731600 | 16736399 | high | 12.00 | 0.000377 |
| ATXN1 | 6310 | chr6 | 16737400 | 16742399 | high | 12.50 | 0.000377 |
| ATXN1 | 6310 | chr6 | 16757000 | 16763199 | high | 15.50 | 0.000377 |
| ATXN1 | 6310 | chr6 | 16743200 | 16749999 | high | 17.00 | 0.000377 |
| ATXN10 | 25814 | chr22 | 45811800 | 45815399 | high | 9.00 | 0.000563 |

| | | | | | | | |
|---|---|---|---|---|---|---|---|
| ATXN10 | 25814 | chr22 | 45776400 | 45779999 | high | 9.00 | 0.000564 |
| ATXN10 | 25814 | chr22 | 45827600 | 45831399 | high | 9.50 | 0.000563 |
| ATXN10 | 25814 | chr22 | 45781200 | 45787599 | high | 16.00 | 0.000564 |
| ATXN10 | 25814 | chr22 | 45670200 | 45680399 | high | 25.50 | 0.000565 |
| ATXN2 | 6311 | chr12 | 111569000 | 111572599 | high | 9.00 | 0.000057 |
| ATXN2 | 6311 | chr12 | 111557200 | 111561999 | high | 12.00 | 0.000057 |
| ATXN3 | 4287 | chr14 | 92103600 | 92106999 | high | 8.50 | 0.000047 |
| ATXN7 | 6314 | chr3 | 63862200 | 63867199 | high | 12.50 | 0.000099 |
| ATXN7 | 6314 | chr3 | 63907800 | 63913999 | high | 15.50 | 0.000099 |
| AUP1 | 550 | chr2 | 74529000 | 74532399 | high | 8.50 | 0.000007 |
| AURKA | 6790 | chr20 | 56383600 | 56388599 | high | 12.50 | 0.000120 |
| AURKB | 9212 | chr17 | 8209600 | 8210799 | low | -5.21 | 0.001122 |
| AVIL | 10677 | chr12 | 57797800 | 57800599 | low | -12.17 | 0.000185 |
| AVL9 | 23080 | chr7 | 32535200 | 32537999 | high | 7.00 | 0.000709 |
| AVPI1 | 60370 | chr10 | 97679800 | 97682999 | high | 8.00 | 0.000618 |
| AXIN1 | 8312 | chr16 | 351600 | 353399 | high | 4.50 | 0.002627 |
| AXL | 558 | chr19 | 41219200 | 41220599 | low | -6.08 | 0.000014 |
| AZI2 | 64343 | chr3 | 28348000 | 28350799 | high | 7.00 | 0.002270 |
| AZIN1 | 51582 | chr8 | 102853800 | 102855799 | high | 5.00 | 0.000502 |
| AZIN2 | 113451 | chr1 | 33080400 | 33082399 | high | 5.00 | 0.003430 |
| B9D1 | 27077 | chr17 | 19360400 | 19363199 | high | 7.00 | 0.001399 |
| B9D1 | 27077 | chr17 | 19344600 | 19348199 | high | 9.00 | 0.001400 |
| B9D1 | 27077 | chr17 | 19376200 | 19380199 | high | 10.00 | 0.001397 |
| B9D2 | 80776 | chr19 | 41362600 | 41366799 | high | 10.50 | 0.001953 |
| B9D2 | 80776 | chr19 | 41351000 | 41356199 | high | 13.00 | 0.001953 |
| BAD | 572 | chr11 | 64283400 | 64285799 | high | 6.00 | 0.000009 |
| BAD | 572 | chr11 | 64266200 | 64270799 | high | 11.50 | 0.000009 |
| BAG1 | 573 | chr9 | 33264000 | 33265799 | high | 4.50 | 0.000017 |
| BAG2 | 9532 | chr6 | 57177800 | 57180399 | low | -11.30 | 0.000167 |
| BAG2 | 9532 | chr6 | 57183400 | 57185799 | low | -10.43 | 0.000167 |
| BAG4 | 9530 | chr8 | 38185400 | 38190799 | high | 13.50 | 0.000250 |
| BAGE | 574 | chr21 | 10424400 | 10430399 | low | -26.08 | 0.000055 |
| BAGE | 574 | chr21 | 10415000 | 10417999 | low | -13.04 | 0.000055 |
| BAGE2 | 85319 | chr21 | 10424400 | 10430399 | low | -26.02 | 0.008185 |
| BAGE2 | 85319 | chr21 | 10415000 | 10417999 | low | -13.04 | 0.008192 |
| BAGE3 | 85318 | chr21 | 10424400 | 10430399 | low | -26.82 | 0.008184 |
| BAGE3 | 85318 | chr21 | 10415000 | 10417999 | low | -13.04 | 0.008192 |
| BAGE4 | 85317 | chr21 | 10424400 | 10430399 | low | -26.69 | 0.008184 |
| BAGE4 | 85317 | chr21 | 10415000 | 10417999 | low | -13.04 | 0.008192 |
| BAGE5 | 85316 | chr21 | 10424400 | 10430399 | low | -24.22 | 0.008184 |
| BAGE5 | 85316 | chr21 | 10415000 | 10417999 | low | -13.04 | 0.008192 |
| BAK1 | 578 | chr6 | 33577000 | 33580999 | high | 10.00 | 0.000017 |
| BANF1 | 8815 | chr11 | 66000400 | 66002599 | low | -9.56 | 0.000134 |
| BANF2 | 140836 | chr20 | 17699400 | 17703399 | high | 10.00 | 0.007957 |
| BANK1 | 55024 | chr4 | 101858200 | 101861199 | high | 7.50 | 0.000540 |
| BANK1 | 55024 | chr4 | 101852200 | 101857399 | high | 13.00 | 0.000540 |
| BARD1 | 580 | chr2 | 214789000 | 214792999 | high | 10.00 | 0.000003 |
| BASP1 | 10409 | chr5 | 17217600 | 17219599 | low | -8.69 | 0.000605 |
| BATF | 10538 | chr14 | 75542200 | 75545199 | high | 7.50 | 0.000139 |
| BATF | 10538 | chr14 | 75529600 | 75533199 | high | 9.00 | 0.000140 |
| BATF3 | 55509 | chr1 | 212696200 | 212697799 | low | -6.95 | 0.000261 |
| BBS2 | 583 | chr16 | 56491200 | 56497799 | high | 16.50 | 0.000010 |
| BBS4 | 585 | chr15 | 72726400 | 72729599 | high | 8.00 | 0.000008 |
| BBS7 | 55212 | chr4 | 121851200 | 121853399 | low | -9.56 | 0.000453 |
| BBS7 | 55212 | chr4 | 121869200 | 121870999 | low | -7.82 | 0.000453 |
| BBS9 | 27241 | chr7 | 33137400 | 33139999 | low | -11.30 | 0.000822 |
| BBX | 56987 | chr3 | 107662800 | 107665199 | high | 6.00 | 0.000529 |
| BCAR1 | 9564 | chr16 | 75249400 | 75252799 | high | 8.50 | 0.000127 |
| BCAR1 | 9564 | chr16 | 75245000 | 75248599 | high | 9.00 | 0.000127 |
| BCAR1 | 9564 | chr16 | 75236200 | 75241399 | high | 13.00 | 0.000127 |
| BCAR1 | 9564 | chr16 | 75227600 | 75232799 | high | 13.00 | 0.000127 |
| BCAR3 | 8412 | chr1 | 93712600 | 93716599 | high | 10.00 | 0.000090 |
| BCAR3 | 8412 | chr1 | 93611600 | 93615999 | high | 11.00 | 0.000090 |
| BCAR3 | 8412 | chr1 | 93623200 | 93630599 | high | 18.50 | 0.000090 |
| BCAR3 | 8412 | chr1 | 93662400 | 93670399 | high | 20.00 | 0.000090 |
| BCAS3 | 54828 | chr17 | 61335200 | 61338599 | high | 8.50 | 0.000894 |
| BCAS3 | 54828 | chr17 | 61008800 | 61012199 | high | 8.50 | 0.000899 |
| BCAS3 | 54828 | chr17 | 61330400 | 61334399 | high | 10.00 | 0.000894 |
| BCAS3 | 54828 | chr17 | 61263000 | 61266999 | high | 10.00 | 0.000895 |
| BCAS3 | 54828 | chr17 | 60829000 | 60832999 | high | 10.00 | 0.000901 |
| BCAS3 | 54828 | chr17 | 61025200 | 61029599 | high | 11.00 | 0.000898 |
| BCAS3 | 54828 | chr17 | 61036200 | 61040999 | high | 12.00 | 0.000898 |
| BCAS3 | 54828 | chr17 | 60936800 | 60941999 | high | 13.00 | 0.000900 |
| BCAS3 | 54828 | chr17 | 61267800 | 61273799 | high | 15.00 | 0.000895 |
| BCAS3 | 54828 | chr17 | 61313400 | 61320199 | high | 17.00 | 0.000894 |
| BCAS3 | 54828 | chr17 | 61015400 | 61022399 | high | 17.50 | 0.000899 |
| BCAS4 | 55653 | chr20 | 50827200 | 50832199 | high | 12.50 | 0.001095 |
| BCAS4 | 55653 | chr20 | 50862600 | 50868999 | high | 16.00 | 0.001094 |

| | | | | | | | |
|---|---|---|---|---|---|---|---|
| BCAT1 | 586 | chr12 | 24914200 | 24919599 | high | 13.50 | 0.000024 |
| BCCIP | 56647 | chr10 | 125823000 | 125824199 | low | -5.21 | 0.000450 |
| BCL3 | 602 | chr19 | 44747600 | 44749999 | low | -10.43 | 0.000013 |
| BCL6 | 604 | chr3 | 187735800 | 187740799 | high | 12.50 | 0.000003 |
| BCL7A | 605 | chr12 | 122021400 | 122026599 | high | 13.00 | 0.000005 |
| BCL7B | 9275 | chr7 | 73536800 | 73540399 | high | 9.00 | 0.000126 |
| BCL9L | 283149 | chr11 | 118901400 | 118905199 | high | 9.50 | 0.002381 |
| BCLAF1 | 9774 | chr6 | 136288800 | 136290599 | low | -7.82 | 0.000072 |
| BCR | 613 | chr22 | 23245200 | 23249799 | high | 11.50 | 0.000026 |
| BDH2 | 56898 | chr4 | 103096800 | 103099999 | high | 8.00 | 0.000552 |
| BDNF | 627 | chr11 | 27709600 | 27713399 | high | 9.50 | 0.000023 |
| BDNF | 627 | chr11 | 27719000 | 27722999 | high | 10.00 | 0.000023 |
| BEND6 | 221336 | chr6 | 56953000 | 56955599 | high | 6.50 | 0.003886 |
| BEND6 | 221336 | chr6 | 57007000 | 57010399 | high | 8.50 | 0.003883 |
| BEND6 | 221336 | chr6 | 57002800 | 57006199 | high | 8.50 | 0.003883 |
| BEND6 | 221336 | chr6 | 57021000 | 57025399 | high | 11.00 | 0.003882 |
| BEND6 | 221336 | chr6 | 56986400 | 56994799 | high | 21.00 | 0.003884 |
| BEST1 | 7439 | chr11 | 61958800 | 61964199 | high | 13.50 | 0.000120 |
| BET1 | 10282 | chr7 | 93989400 | 93991399 | low | -8.69 | 0.000109 |
| BET1 | 10282 | chr7 | 94001000 | 94001999 | low | -4.34 | 0.000109 |
| BFSP1 | 631 | chr20 | 17504000 | 17507399 | high | 8.50 | 0.000036 |
| BFSP1 | 631 | chr20 | 17554400 | 17557999 | high | 9.00 | 0.000036 |
| BFSP1 | 631 | chr20 | 17539200 | 17544199 | high | 12.50 | 0.000036 |
| BFSP1 | 631 | chr20 | 17545200 | 17550999 | high | 14.50 | 0.000036 |
| BHMG1 | 388553 | chr19 | 45737000 | 45741799 | high | 12.00 | 0.008495 |
| BHMG1 | 388553 | chr19 | 45763600 | 45772199 | high | 21.50 | 0.008490 |
| BICC1 | 80114 | chr10 | 58547400 | 58551799 | high | 11.00 | 0.001368 |
| BICC1 | 80114 | chr10 | 58558200 | 58564599 | high | 16.00 | 0.001368 |
| BICC1 | 80114 | chr10 | 58612200 | 58619199 | high | 17.50 | 0.001367 |
| BICD1 | 636 | chr12 | 32340600 | 32341999 | low | -6.08 | 0.000020 |
| BICD2 | 23299 | chr9 | 92762400 | 92766199 | low | -16.73 | 0.000251 |
| BICDL2 | 146439 | chr16 | 3028600 | 3032399 | high | 9.50 | 0.008486 |
| BIN1 | 274 | chr2 | 127104400 | 127108399 | high | 10.00 | 0.000002 |
| BIN3 | 55909 | chr8 | 22651000 | 22653799 | high | 7.00 | 0.002468 |
| BIN3 | 55909 | chr8 | 22620200 | 22624399 | high | 10.50 | 0.002472 |
| BIN3 | 55909 | chr8 | 22634800 | 22639599 | high | 12.00 | 0.002470 |
| BIRC3 | 330 | chr11 | 102318200 | 102321599 | high | 8.50 | 0.000003 |
| BIRC6 | 57448 | chr2 | 32498400 | 32503399 | high | 12.50 | 0.001768 |
| BIRC6 | 57448 | chr2 | 32541600 | 32546799 | high | 13.00 | 0.001765 |
| BIVM | 54841 | chr13 | 102807200 | 102809199 | low | -8.69 | 0.000533 |
| BLCAP | 10904 | chr20 | 37525800 | 37529199 | high | 8.50 | 0.000291 |
| BLK | 640 | chr8 | 11497200 | 11500999 | high | 9.50 | 0.000056 |
| BLM | 641 | chr15 | 90715800 | 90718999 | high | 8.00 | 0.000007 |
| BLM | 641 | chr15 | 90750800 | 90754199 | high | 8.50 | 0.000007 |
| BLM | 641 | chr15 | 90731200 | 90734599 | high | 8.50 | 0.000007 |
| BLM | 641 | chr15 | 90761200 | 90765399 | high | 10.50 | 0.000007 |
| BLM | 641 | chr15 | 90790000 | 90794999 | high | 12.50 | 0.000007 |
| BLM | 641 | chr15 | 90735400 | 90740399 | high | 12.50 | 0.000007 |
| BLVRA | 644 | chr7 | 43763000 | 43767199 | high | 10.50 | 0.000015 |
| BLVRA | 644 | chr7 | 43781200 | 43789599 | high | 21.00 | 0.000015 |
| BMF | 90427 | chr15 | 40104600 | 40108799 | high | 10.50 | 0.002255 |
| BMI1 | 648 | chr10 | 22319600 | 22322599 | low | -13.04 | 0.000029 |
| BMP1 | 649 | chr8 | 22162600 | 22165599 | high | 7.50 | 0.000029 |
| BMP2K | 55589 | chr4 | 78880800 | 78884799 | high | 10.00 | 0.000705 |
| BMP2K | 55589 | chr4 | 78887200 | 78892199 | high | 12.50 | 0.000705 |
| BMP4 | 652 | chr14 | 53951200 | 53958799 | low | -7.53 | 0.002966 |
| BMPR1A | 657 | chr10 | 86768400 | 86769999 | low | -6.95 | 0.000008 |
| BMPR1B | 658 | chr4 | 95120400 | 95122599 | low | -9.56 | 0.000007 |
| BMS1P4 | 729096 | chr10 | 73715600 | 73721599 | high | 15.00 | 0.009891 |
| BNC1 | 646 | chr15 | 83274000 | 83276799 | low | -12.17 | 0.000008 |
| BNC1 | 646 | chr15 | 83266800 | 83268599 | low | -7.82 | 0.000008 |
| BNC2 | 54796 | chr9 | 16725600 | 16728399 | high | 7.00 | 0.003276 |
| BNC2 | 54796 | chr9 | 16695800 | 16699599 | high | 9.50 | 0.003282 |
| BNC2 | 54796 | chr9 | 16459200 | 16462999 | high | 9.50 | 0.003329 |
| BNC2 | 54796 | chr9 | 16744800 | 16748799 | high | 10.00 | 0.003272 |
| BNC2 | 54796 | chr9 | 16639600 | 16643799 | high | 10.50 | 0.003293 |
| BNC2 | 54796 | chr9 | 16631800 | 16636999 | high | 13.00 | 0.003295 |
| BNC2 | 54796 | chr9 | 16667800 | 16673199 | high | 13.50 | 0.003288 |
| BNC2 | 54796 | chr9 | 16621000 | 16626399 | high | 13.50 | 0.003297 |
| BNC2 | 54796 | chr9 | 16430400 | 16435799 | high | 13.50 | 0.003335 |
| BNC2 | 54796 | chr9 | 16818400 | 16823999 | high | 14.00 | 0.003258 |
| BNC2 | 54796 | chr9 | 16679000 | 16685999 | high | 17.50 | 0.003285 |
| BNC2 | 54796 | chr9 | 16783400 | 16790599 | high | 18.00 | 0.003265 |
| BNIP2 | 663 | chr15 | 59654800 | 59659199 | high | 11.00 | 0.000011 |
| BNIPL | 149428 | chr1 | 151045600 | 151048999 | high | 8.50 | 0.000989 |
| BOD1 | 91272 | chr5 | 173612000 | 173615999 | high | 10.00 | 0.000526 |
| BOD1L1 | 259282 | chr4 | 13625800 | 13628999 | high | 8.00 | 0.019029 |
| BOK | 666 | chr2 | 241556800 | 241559999 | high | 8.00 | 0.000003 |

| | | | | | | | |
|---|---|---|---|---|---|---|---|
| BOP1 | 23246 | chr8 | 144290400 | 144293199 | high | 7.00 | 0.000161 |
| BORCS5 | 118426 | chr12 | 12434000 | 12436599 | low | -11.30 | 0.009524 |
| BORCS5 | 118426 | chr12 | 12356400 | 12358999 | low | -11.30 | 0.009584 |
| BPHL | 670 | chr6 | 3137800 | 3140999 | high | 8.00 | 0.000214 |
| BPI | 671 | chr20 | 38302800 | 38307599 | high | 12.00 | 0.000018 |
| BPTF | 2186 | chr17 | 67862000 | 67868399 | high | 16.00 | 0.000032 |
| BRAT1 | 221927 | chr7 | 2552600 | 2557199 | high | 11.50 | 0.009660 |
| BRCA1 | 672 | chr17 | 43101600 | 43107999 | high | 16.00 | 0.000016 |
| BRD1 | 23774 | chr22 | 49825800 | 49827399 | low | -6.95 | 0.000477 |
| BRD4 | 23476 | chr19 | 15249600 | 15254999 | high | 13.50 | 0.001539 |
| BRD8 | 10902 | chr5 | 138178000 | 138178599 | low | -2.60 | 0.000079 |
| BRD9 | 65980 | chr5 | 889800 | 895199 | high | 13.50 | 0.009141 |
| BRE | 9577 | chr2 | 28101000 | 28103799 | high | 7.00 | 0.000341 |
| BRE | 9577 | chr2 | 28324800 | 28327999 | high | 8.00 | 0.000338 |
| BRE | 9577 | chr2 | 27982800 | 27985999 | high | 8.00 | 0.000342 |
| BRE | 9577 | chr2 | 27987000 | 27990399 | high | 8.50 | 0.000342 |
| BRE | 9577 | chr2 | 28231000 | 28234599 | high | 9.00 | 0.000339 |
| BRE | 9577 | chr2 | 28319600 | 28323599 | high | 10.00 | 0.000338 |
| BRE | 9577 | chr2 | 28070800 | 28075599 | high | 12.00 | 0.000341 |
| BRE | 9577 | chr2 | 28313600 | 28318599 | high | 12.50 | 0.000338 |
| BRE | 9577 | chr2 | 28121000 | 28125999 | high | 12.50 | 0.000341 |
| BRE | 9577 | chr2 | 28076600 | 28081799 | high | 13.00 | 0.000341 |
| BRE | 9577 | chr2 | 28000400 | 28005799 | high | 13.50 | 0.000342 |
| BRE | 9577 | chr2 | 28032000 | 28039799 | high | 19.50 | 0.000342 |
| BRE | 9577 | chr2 | 28272000 | 28281999 | high | 25.00 | 0.000339 |
| BRF1 | 2972 | chr14 | 105311600 | 105315799 | high | 10.50 | 0.000028 |
| BRF2 | 55290 | chr8 | 37849000 | 37850199 | low | -5.21 | 0.001461 |
| BRINP3 | 339479 | chr1 | 190295000 | 190298599 | high | 9.00 | 0.001784 |
| BRINP3 | 339479 | chr1 | 190267000 | 190270599 | high | 9.00 | 0.001784 |
| BRINP3 | 339479 | chr1 | 190275000 | 190280599 | high | 14.00 | 0.001784 |
| BRINP3 | 339479 | chr1 | 190393800 | 190399599 | high | 14.50 | 0.001783 |
| BRIP1 | 83990 | chr17 | 61857800 | 61861399 | low | -15.65 | 0.001358 |
| BRIP1 | 83990 | chr17 | 61835400 | 61838999 | low | -15.65 | 0.001358 |
| BRIP1 | 83990 | chr17 | 61725400 | 61728199 | low | -12.17 | 0.001361 |
| BRIP1 | 83990 | chr17 | 61707400 | 61709999 | low | -11.30 | 0.001361 |
| BRIP1 | 83990 | chr17 | 61814000 | 61816199 | low | -9.56 | 0.001359 |
| BRIP1 | 83990 | chr17 | 61862400 | 61863999 | low | -6.95 | 0.001358 |
| BRMS1L | 84312 | chr14 | 35826400 | 35826999 | low | -2.60 | 0.002353 |
| BRPF1 | 7862 | chr3 | 9743800 | 9746199 | low | -10.43 | 0.000807 |
| BSCL2 | 26580 | chr11 | 62690800 | 62693199 | high | 6.00 | 0.000424 |
| BSCL2 | 26580 | chr11 | 62704600 | 62707399 | high | 7.00 | 0.000424 |
| BSN | 8927 | chr3 | 49613400 | 49616599 | high | 8.00 | 0.000180 |
| BTAF1 | 9044 | chr10 | 91939800 | 91943799 | high | 10.00 | 0.000098 |
| BTBD11 | 121551 | chr12 | 107397600 | 107402399 | high | 12.00 | 0.001132 |
| BTBD3 | 22903 | chr20 | 11890800 | 11892599 | low | -7.82 | 0.001926 |
| BTBD6 | 90135 | chr14 | 105248200 | 105250399 | low | -9.56 | 0.000856 |
| BTBD9 | 114781 | chr6 | 38514600 | 38518399 | high | 9.50 | 0.002980 |
| BTBD9 | 114781 | chr6 | 38489200 | 38493799 | high | 11.50 | 0.002982 |
| BTBD9 | 114781 | chr6 | 38440400 | 38444999 | high | 11.50 | 0.002986 |
| BTD | 686 | chr3 | 15628600 | 15631399 | high | 7.00 | 0.000044 |
| BTD | 686 | chr3 | 15597800 | 15602999 | high | 13.00 | 0.000044 |
| BTF3 | 689 | chr5 | 73502800 | 73507799 | high | 12.50 | 0.000009 |
| BTF3L4 | 91408 | chr1 | 52052400 | 52056999 | high | 11.50 | 0.001756 |
| BTG4 | 54766 | chr11 | 111471600 | 111477199 | high | 14.00 | 0.000491 |
| BTN3A2 | 11118 | chr6 | 26376800 | 26379399 | high | 6.50 | 0.000422 |
| BTN3A2 | 11118 | chr6 | 26363200 | 26367399 | high | 10.50 | 0.000422 |
| BTN3A3 | 10384 | chr6 | 26438600 | 26443199 | high | 11.50 | 0.000393 |
| BTRC | 8945 | chr10 | 101363800 | 101366599 | low | -12.17 | 0.000088 |
| BUB1 | 699 | chr2 | 110651000 | 110657399 | high | 16.00 | 0.000006 |
| BUB1B | 701 | chr15 | 40169400 | 40171799 | high | 6.00 | 0.000017 |
| BUB3 | 9184 | chr10 | 123154000 | 123156399 | high | 6.00 | 0.000075 |
| BUD13 | 84811 | chr11 | 116770800 | 116773799 | high | 7.50 | 0.000726 |
| BVES | 11149 | chr6 | 105134400 | 105139799 | high | 13.50 | 0.000106 |
| BZW2 | 28969 | chr7 | 16650200 | 16655999 | low | -25.26 | 0.001740 |
| BZW2 | 28969 | chr7 | 16688000 | 16691199 | low | -13.91 | 0.001736 |
| C1D | 10438 | chr2 | 68050200 | 68054599 | high | 11.00 | 0.000153 |
| C1orf35 | 79169 | chr1 | 228101400 | 228104599 | high | 8.00 | 0.000347 |
| C1orf50 | 79078 | chr1 | 42766200 | 42768999 | high | 7.00 | 0.001849 |
| C1QL4 | 338761 | chr12 | 49333800 | 49339399 | high | 14.00 | 0.006867 |
| C1QTNF1 | 114897 | chr17 | 79035800 | 79039399 | high | 9.00 | 0.001454 |
| C1QTNF3 | 114899 | chr5 | 34043000 | 34046399 | high | 8.50 | 0.003375 |
| C1QTNF7 | 114905 | chr4 | 15393400 | 15398399 | high | 12.50 | 0.007465 |
| C1RL | 51279 | chr12 | 7106200 | 7110799 | high | 11.50 | 0.007216 |
| CA12 | 771 | chr15 | 63329200 | 63333799 | high | 11.50 | 0.000012 |
| CA12 | 771 | chr15 | 63370800 | 63375599 | high | 12.00 | 0.000012 |
| CA12 | 771 | chr15 | 63363800 | 63369799 | high | 15.00 | 0.000012 |
| CA14 | 23632 | chr1 | 150258800 | 150269199 | high | 26.00 | 0.000157 |
| CA5B | 11238 | chrX | 15770600 | 15772799 | low | -9.56 | 0.000713 |

| | | | | | | | |
|---|---|---|---|---|---|---|---|
| CA5BP1 | 340591 | chrX | 15674400 | 15681999 | high | 19.00 | 0.002414 |
| CA9 | 768 | chr9 | 35680200 | 35683799 | high | 9.00 | 0.000022 |
| CAB39 | 51719 | chr2 | 230712600 | 230714399 | low | -7.82 | 0.000224 |
| CAB39L | 81617 | chr13 | 49316600 | 49323199 | high | 16.50 | 0.001655 |
| CAB39L | 81617 | chr13 | 49309200 | 49315799 | high | 16.50 | 0.001655 |
| CAB39L | 81617 | chr13 | 49351400 | 49359399 | high | 20.00 | 0.001654 |
| CABIN1 | 23523 | chr22 | 24155200 | 24157599 | high | 6.00 | 0.000974 |
| CABIN1 | 23523 | chr22 | 24025600 | 24029199 | high | 9.00 | 0.000979 |
| CABIN1 | 23523 | chr22 | 24174800 | 24178799 | high | 10.00 | 0.000973 |
| CABIN1 | 23523 | chr22 | 24077000 | 24081399 | high | 11.00 | 0.000977 |
| CABIN1 | 23523 | chr22 | 24059400 | 24064399 | high | 12.50 | 0.000978 |
| CABLES1 | 91768 | chr18 | 23207600 | 23210999 | high | 8.50 | 0.003954 |
| CABLES1 | 91768 | chr18 | 23220200 | 23223999 | high | 9.50 | 0.003952 |
| CABLES1 | 91768 | chr18 | 23162800 | 23170799 | high | 20.00 | 0.003962 |
| CABLES1 | 91768 | chr18 | 23134800 | 23142799 | high | 20.00 | 0.003967 |
| CABP4 | 57010 | chr11 | 67452400 | 67457399 | high | 12.50 | 0.000845 |
| CADM4 | 199731 | chr19 | 43634600 | 43639399 | high | 12.00 | 0.004577 |
| CAHM | 100526820 | chr6 | 163413200 | 163416399 | low | -13.91 | 0.000062 |
| CALD1 | 800 | chr7 | 134830200 | 134833599 | high | 8.50 | 0.000006 |
| CALD1 | 800 | chr7 | 134915000 | 134918799 | high | 9.50 | 0.000006 |
| CALD1 | 800 | chr7 | 134864200 | 134869199 | high | 12.50 | 0.000006 |
| CALM1 | 801 | chr14 | 90397000 | 90399199 | high | 5.50 | 0.000009 |
| CALML4 | 91860 | chr15 | 68198200 | 68203399 | high | 13.00 | 0.001347 |
| CALML4 | 91860 | chr15 | 68189600 | 68194799 | high | 13.00 | 0.001347 |
| CALR | 811 | chr19 | 12938400 | 12941199 | low | -12.17 | 0.000063 |
| CALR3 | 125972 | chr19 | 16492800 | 16498799 | high | 15.00 | 0.007638 |
| CANT1 | 124583 | chr17 | 78993400 | 78996199 | high | 7.00 | 0.001577 |
| CANX | 821 | chr5 | 179699400 | 179700199 | low | -3.47 | 0.000005 |
| CAP1 | 10487 | chr1 | 40040200 | 40042199 | low | -8.69 | 0.000262 |
| CAPG | 822 | chr2 | 85394400 | 85397999 | high | 9.00 | 0.000010 |
| CAPG | 822 | chr2 | 85410600 | 85415799 | high | 13.00 | 0.000010 |
| CAPN3 | 825 | chr15 | 42411600 | 42414799 | high | 8.00 | 0.000019 |
| CAPN3 | 825 | chr15 | 42375800 | 42379999 | high | 10.50 | 0.000019 |
| CAPN3 | 825 | chr15 | 42359400 | 42363799 | high | 11.00 | 0.000019 |
| CAPN3 | 825 | chr15 | 42405400 | 42410199 | high | 12.00 | 0.000019 |
| CAPN3 | 825 | chr15 | 42384000 | 42389999 | high | 15.00 | 0.000019 |
| CAPN7 | 23473 | chr3 | 15210600 | 15213599 | high | 7.50 | 0.001543 |
| CAPN7 | 23473 | chr3 | 15204600 | 15209399 | high | 12.00 | 0.001544 |
| CAPS2 | 84698 | chr12 | 75323600 | 75326199 | low | -11.30 | 0.001124 |
| CAPS2 | 84698 | chr12 | 75320800 | 75322799 | low | -8.69 | 0.001124 |
| CAPZA1 | 829 | chr1 | 112624000 | 112629599 | high | 14.00 | 0.000007 |
| CAPZB | 832 | chr1 | 19371400 | 19373999 | high | 6.50 | 0.000043 |
| CAPZB | 832 | chr1 | 19451000 | 19453799 | high | 7.00 | 0.000043 |
| CAPZB | 832 | chr1 | 19442200 | 19446999 | high | 12.00 | 0.000043 |
| CAPZB | 832 | chr1 | 19456400 | 19461999 | high | 14.00 | 0.000043 |
| CARD18 | 59082 | chr11 | 105137000 | 105140799 | high | 9.50 | 0.000562 |
| CARD9 | 64170 | chr9 | 136370400 | 136375999 | high | 14.00 | 0.000471 |
| CARM1 | 10498 | chr19 | 10914000 | 10916799 | high | 7.00 | 0.000962 |
| CARM1 | 10498 | chr19 | 10899000 | 10901999 | high | 7.50 | 0.000963 |
| CARM1 | 10498 | chr19 | 10886600 | 10892599 | high | 15.00 | 0.000964 |
| CASC1 | 55259 | chr12 | 25194400 | 25197199 | high | 7.00 | 0.002193 |
| CASC4 | 113201 | chr15 | 44338400 | 44341799 | high | 8.50 | 0.002553 |
| CASC4 | 113201 | chr15 | 44287200 | 44290799 | high | 9.00 | 0.002556 |
| CASC4 | 113201 | chr15 | 44383000 | 44388399 | high | 13.50 | 0.002551 |
| CASKIN2 | 57513 | chr17 | 75503200 | 75506999 | high | 9.50 | 0.000762 |
| CASP10 | 843 | chr2 | 201218400 | 201221199 | low | -12.17 | 0.000004 |
| CASP10 | 843 | chr2 | 201209400 | 201211799 | low | -10.43 | 0.000004 |
| CASP2 | 835 | chr7 | 143285200 | 143290399 | high | 13.00 | 0.000006 |
| CASP4 | 837 | chr11 | 104958800 | 104960799 | high | 5.00 | 0.000008 |
| CASP4 | 837 | chr11 | 104964800 | 104967999 | high | 8.00 | 0.000008 |
| CASP4 | 837 | chr11 | 104945000 | 104949599 | high | 11.50 | 0.000008 |
| CASP5 | 838 | chr11 | 105012200 | 105014599 | low | -10.43 | 0.000008 |
| CASP8 | 841 | chr2 | 201235000 | 201237799 | high | 7.00 | 0.000004 |
| CASP9 | 842 | chr1 | 15522600 | 15524999 | low | -10.43 | 0.000054 |
| CASS4 | 57091 | chr20 | 56430400 | 56433199 | high | 7.00 | 0.001012 |
| CAST | 831 | chr5 | 96770000 | 96771999 | high | 5.00 | 0.000009 |
| CAST | 831 | chr5 | 96701800 | 96704599 | high | 7.00 | 0.000009 |
| CAST | 831 | chr5 | 96726000 | 96729799 | high | 9.50 | 0.000009 |
| CAST | 831 | chr5 | 96773600 | 96778599 | high | 12.50 | 0.000009 |
| CAST | 831 | chr5 | 96740200 | 96745399 | high | 13.00 | 0.000009 |
| CAST | 831 | chr5 | 96711000 | 96716999 | high | 15.00 | 0.000009 |
| CAV2 | 858 | chr7 | 116499200 | 116502999 | low | -16.43 | 0.000007 |
| CAV2 | 858 | chr7 | 116505000 | 116506599 | low | -6.95 | 0.000007 |
| CBARP | 255057 | chr19 | 1237000 | 1238399 | high | 3.50 | 0.000021 |
| CBFB | 865 | chr16 | 67059000 | 67061999 | high | 7.50 | 0.000013 |
| CBFB | 865 | chr16 | 67038800 | 67045199 | high | 16.00 | 0.000013 |
| CBFB | 865 | chr16 | 67086800 | 67093999 | high | 18.00 | 0.000013 |
| CBLB | 868 | chr3 | 105670600 | 105674399 | high | 9.50 | 0.000008 |

| | | | | | | | |
|---|---|---|---|---|---|---|---|
| CBLB | 868 | chr3 | 105685800 | 105689999 | high | 10.50 | 0.000008 |
| CBLB | 868 | chr3 | 105678200 | 105682599 | high | 11.00 | 0.000008 |
| CBLL1 | 79872 | chr7 | 107743000 | 107748199 | low | -22.67 | 0.000741 |
| CBR1 | 873 | chr21 | 36070200 | 36073199 | high | 7.50 | 0.000024 |
| CBX1 | 10951 | chr17 | 48099200 | 48101399 | low | -9.56 | 0.000228 |
| CBX7 | 23492 | chr22 | 39150800 | 39153999 | high | 8.00 | 0.000600 |
| CBY1 | 25776 | chr22 | 38655600 | 38659799 | high | 10.50 | 0.000667 |
| CC2D1B | 200014 | chr1 | 52365200 | 52366799 | low | -6.95 | 0.003820 |
| CC2D1B | 200014 | chr1 | 52357400 | 52358799 | low | -6.08 | 0.003820 |
| CCAR2 | 57805 | chr8 | 22604200 | 22605599 | high | 3.50 | 0.002557 |
| CCAR2 | 57805 | chr8 | 22620200 | 22624399 | high | 10.50 | 0.002555 |
| CCBE1 | 147372 | chr18 | 59532400 | 59535799 | high | 8.50 | 0.002475 |
| CCBE1 | 147372 | chr18 | 59513400 | 59517199 | high | 9.50 | 0.002476 |
| CCBE1 | 147372 | chr18 | 59660800 | 59665399 | high | 11.50 | 0.002470 |
| CCBE1 | 147372 | chr18 | 59554000 | 59559199 | high | 13.00 | 0.002475 |
| CCBE1 | 147372 | chr18 | 59482200 | 59487999 | high | 14.50 | 0.002478 |
| CCBE1 | 147372 | chr18 | 59668600 | 59674599 | high | 15.00 | 0.002470 |
| CCBE1 | 147372 | chr18 | 59451800 | 59457799 | high | 15.00 | 0.002479 |
| CCBE1 | 147372 | chr18 | 59497000 | 59506999 | high | 25.00 | 0.002477 |
| CDH11 | 1009 | chr16 | 65045400 | 65048399 | low | -13.04 | 0.000016 |
| CDH13 | 1012 | chr16 | 82759800 | 82765999 | high | 15.50 | 0.000012 |
| CDH17 | 1015 | chr8 | 94141000 | 94144399 | high | 8.50 | 0.000011 |
| CDH17 | 1015 | chr8 | 94188800 | 94192799 | high | 10.00 | 0.000011 |
| CDH2 | 1000 | chr18 | 28050400 | 28052999 | low | -11.30 | 0.000036 |
| CDH2 | 1000 | chr18 | 28033400 | 28035199 | low | -7.83 | 0.000036 |
| CDIP1 | 29965 | chr16 | 4535200 | 4538799 | high | 9.00 | 0.006607 |
| CDIPT | 10423 | chr16 | 29862200 | 29863399 | low | -5.21 | 0.000349 |
| CDK1 | 983 | chr10 | 60779000 | 60780599 | low | -6.95 | 0.000016 |
| CDK14 | 5218 | chr7 | 91194000 | 91196999 | high | 7.50 | 0.000057 |
| CDK14 | 5218 | chr7 | 90822000 | 90826599 | high | 11.50 | 0.000057 |
| CDK14 | 5218 | chr7 | 91111200 | 91116199 | high | 12.50 | 0.000057 |
| CDK15 | 65061 | chr2 | 201816800 | 201820999 | high | 10.50 | 0.000322 |
| CDK15 | 65061 | chr2 | 201870000 | 201874999 | high | 12.50 | 0.000322 |
| CDK17 | 5128 | chr12 | 96295000 | 96298399 | high | 8.50 | 0.000053 |
| CDK17 | 5128 | chr12 | 96281800 | 96285199 | high | 8.50 | 0.000053 |
| CDK17 | 5128 | chr12 | 96310000 | 96315599 | high | 14.00 | 0.000053 |
| CDK17 | 5128 | chr12 | 96303600 | 96309199 | high | 14.00 | 0.000053 |
| CDK17 | 5128 | chr12 | 96287200 | 96292999 | high | 14.50 | 0.000053 |
| CDK9 | 1025 | chr9 | 127785000 | 127788199 | high | 8.00 | 0.000008 |
| CDKL1 | 8814 | chr14 | 50348400 | 50353599 | high | 13.00 | 0.000175 |
| CDKL1 | 8814 | chr14 | 50392200 | 50398599 | high | 16.00 | 0.000175 |
| CDKL4 | 344387 | chr2 | 39223600 | 39228799 | high | 13.00 | 0.008780 |
| CDKN3 | 1033 | chr14 | 54410600 | 54414199 | high | 9.00 | 0.000019 |
| CDNF | 441549 | chr10 | 14837600 | 14841399 | high | 9.50 | 0.003307 |
| CDNF | 441549 | chr10 | 14820200 | 14824199 | high | 10.00 | 0.003310 |
| CDS2 | 8760 | chr20 | 5123400 | 5127999 | high | 11.50 | 0.001710 |
| CDT1 | 81620 | chr16 | 88803400 | 88807799 | high | 11.00 | 0.000919 |
| CDT1 | 81620 | chr16 | 88808800 | 88813399 | high | 11.50 | 0.000919 |
| CEBPB | 1051 | chr20 | 50189600 | 50191999 | high | 6.00 | 0.000021 |
| CEBPZ | 10153 | chr2 | 37209400 | 37214599 | high | 13.00 | 0.000273 |
| CELA1 | 1990 | chr12 | 51345200 | 51346799 | high | 4.00 | 0.000039 |
| CELA1 | 1990 | chr12 | 51329600 | 51334199 | high | 11.50 | 0.000039 |
| CELF1 | 10658 | chr11 | 47512800 | 47518999 | high | 15.50 | 0.000224 |
| CELSR2 | 1952 | chr1 | 109270600 | 109273999 | high | 8.50 | 0.000018 |
| CEMIP | 57214 | chr15 | 80788600 | 80798799 | high | 25.50 | 0.000708 |
| CENPB | 1059 | chr20 | 3784800 | 3788799 | high | 10.00 | 0.000280 |
| CENPC | 1060 | chr4 | 67543400 | 67545999 | low | -11.30 | 0.000016 |
| CENPF | 1063 | chr1 | 214602200 | 214604599 | high | 6.00 | 0.000005 |
| CENPH | 64946 | chr5 | 69188400 | 69191399 | high | 7.50 | 0.000939 |
| CENPK | 64105 | chr5 | 65548600 | 65550399 | low | -7.82 | 0.000978 |
| CENPO | 79172 | chr2 | 24791200 | 24795399 | high | 10.50 | 0.003194 |
| CENPP | 401541 | chr9 | 92599600 | 92602599 | high | 7.50 | 0.004336 |
| CENPP | 401541 | chr9 | 92323000 | 92326799 | high | 9.50 | 0.004349 |
| CENPP | 401541 | chr9 | 92387800 | 92391999 | high | 10.50 | 0.004346 |
| CENPP | 401541 | chr9 | 92583000 | 92587599 | high | 11.50 | 0.004337 |
| CENPP | 401541 | chr9 | 92368400 | 92375199 | high | 17.00 | 0.004347 |
| CENPP | 401541 | chr9 | 92327600 | 92334399 | high | 17.00 | 0.004349 |
| CENPT | 80152 | chr16 | 67831400 | 67835999 | high | 11.50 | 0.001182 |
| CENPU | 79682 | chr4 | 184732400 | 184733999 | high | 4.00 | 0.000431 |
| CENPV | 201161 | chr17 | 16350600 | 16353999 | high | 8.50 | 0.012303 |
| CEP112 | 201134 | chr17 | 66114600 | 66119399 | high | 12.00 | 0.003042 |
| CEP112 | 201134 | chr17 | 66041200 | 66046199 | high | 12.50 | 0.003046 |
| CEP120 | 153241 | chr5 | 123422200 | 123424399 | low | -9.56 | 0.001242 |
| CEP126 | 57562 | chr11 | 101912000 | 101917999 | high | 15.00 | 0.000565 |
| CEP128 | 145508 | chr14 | 80936600 | 80938999 | low | -10.43 | 0.001798 |
| CEP128 | 145508 | chr14 | 80542000 | 80544199 | low | -9.56 | 0.001807 |
| CEP128 | 145508 | chr14 | 80872400 | 80873799 | low | -6.08 | 0.001799 |
| CEP131 | 22994 | chr17 | 81220000 | 81223999 | high | 10.00 | 0.000283 |

| | | | | | | | |
|---|---|---|---|---|---|---|---|
| CEP152 | 22995 | chr15 | 48751400 | 48754999 | high | 9.00 | 0.000472 |
| CEP152 | 22995 | chr15 | 48807600 | 48811399 | high | 9.50 | 0.000471 |
| CEP152 | 22995 | chr15 | 48785800 | 48789799 | high | 10.00 | 0.000471 |
| CEP152 | 22995 | chr15 | 48778000 | 48783399 | high | 13.50 | 0.000471 |
| CEP19 | 84984 | chr3 | 196710600 | 196712799 | high | 5.50 | 0.000432 |
| CEP192 | 55125 | chr18 | 12990600 | 12993599 | high | 7.50 | 0.004243 |
| CEP192 | 55125 | chr18 | 13025600 | 13029999 | high | 11.00 | 0.004232 |
| CEP192 | 55125 | chr18 | 13018400 | 13023399 | high | 12.50 | 0.004234 |
| CEP250 | 11190 | chr20 | 35451400 | 35455799 | high | 11.00 | 0.000316 |
| CEP350 | 9857 | chr1 | 180073800 | 180077599 | high | 9.50 | 0.000055 |
| CEP350 | 9857 | chr1 | 180046200 | 180050199 | high | 10.00 | 0.000055 |
| CEP350 | 9857 | chr1 | 180022200 | 180030999 | high | 22.00 | 0.000055 |
| CEP41 | 95681 | chr7 | 130399600 | 130405199 | high | 14.00 | 0.000734 |
| CEP57 | 9702 | chr11 | 95832400 | 95837399 | high | 12.50 | 0.000101 |
| CEP63 | 80254 | chr3 | 134485000 | 134487199 | high | 5.50 | 0.000597 |
| CEP85 | 64793 | chr1 | 26273200 | 26280399 | high | 18.00 | 0.002466 |
| CEP85L | 387119 | chr6 | 118650200 | 118652199 | high | 5.00 | 0.003263 |
| CEP85L | 387119 | chr6 | 118655000 | 118659399 | high | 11.00 | 0.003263 |
| CEP85L | 387119 | chr6 | 118706200 | 118711799 | high | 14.00 | 0.003261 |
| CEP95 | 90799 | chr17 | 64528000 | 64533999 | high | 15.00 | 0.001407 |
| CERK | 64781 | chr22 | 46736800 | 46739599 | high | 7.00 | 0.001386 |
| CERKL | 375298 | chr2 | 181545200 | 181548799 | high | 9.00 | 0.002067 |
| CERS5 | 91012 | chr12 | 50165000 | 50167399 | high | 6.00 | 0.001814 |
| CERS5 | 91012 | chr12 | 50141200 | 50144999 | high | 9.50 | 0.001815 |
| CES2 | 8824 | chr16 | 66939200 | 66942999 | high | 9.50 | 0.000132 |
| CES4A | 283848 | chr16 | 66995800 | 67001799 | high | 15.00 | 0.004237 |
| CETP | 1071 | chr16 | 56968200 | 56973199 | high | 12.50 | 0.000019 |
| CFAP20 | 29105 | chr16 | 58127000 | 58129999 | low | -13.04 | 0.000501 |
| CFAP45 | 25790 | chr1 | 159886600 | 159889999 | high | 8.50 | 0.000161 |
| CFAP54 | 144535 | chr12 | 96491000 | 96499199 | high | 20.50 | 0.001498 |
| CFAP61 | 26074 | chr20 | 20051400 | 20052599 | low | -5.21 | 0.001300 |
| CFAP69 | 79846 | chr7 | 90254200 | 90256199 | high | 5.00 | 0.000885 |
| CFAP70 | 118491 | chr10 | 73351400 | 73354199 | high | 7.00 | 0.001615 |
| CFAP70 | 118491 | chr10 | 73355000 | 73359199 | high | 10.50 | 0.001615 |
| CFAP70 | 118491 | chr10 | 73290600 | 73294799 | high | 10.50 | 0.001617 |
| CFAP99 | 402160 | chr4 | 2443000 | 2445799 | low | -12.17 | 0.018291 |
| CFAP99 | 402160 | chr4 | 2430600 | 2433199 | low | -11.30 | 0.018384 |
| CFDP1 | 10428 | chr16 | 75303800 | 75307399 | high | 9.00 | 0.000138 |
| CFDP1 | 10428 | chr16 | 75388400 | 75392399 | high | 10.00 | 0.000138 |
| CFDP1 | 10428 | chr16 | 75416600 | 75420999 | high | 11.00 | 0.000138 |
| CFL1 | 1072 | chr11 | 65856200 | 65858999 | high | 7.00 | 0.000016 |
| CFL2 | 1073 | chr14 | 34713000 | 34715399 | low | -10.43 | 0.000031 |
| CFLAR | 8837 | chr2 | 201134400 | 201136799 | high | 6.00 | 0.000044 |
| CHMP7 | 91782 | chr8 | 23242600 | 23247199 | high | 11.50 | 0.003949 |
| CHMP7 | 91782 | chr8 | 23248200 | 23253999 | high | 14.50 | 0.003948 |
| CHN1 | 1123 | chr2 | 174812400 | 174816799 | high | 11.00 | 0.000006 |
| CHN1 | 1123 | chr2 | 174820800 | 174826399 | high | 14.00 | 0.000006 |
| CHP1 | 11261 | chr15 | 41229200 | 41233399 | low | -18.65 | 0.000273 |
| CHP1 | 11261 | chr15 | 41262000 | 41264599 | low | -11.30 | 0.000273 |
| CHP1 | 11261 | chr15 | 41259000 | 41261199 | low | -9.56 | 0.000273 |
| CHPF2 | 54480 | chr7 | 151232600 | 151233999 | low | -6.08 | 0.000360 |
| CHPT1 | 56994 | chr12 | 101697200 | 101698999 | high | 4.50 | 0.000560 |
| CHRAC1 | 54108 | chr8 | 140511400 | 140512799 | low | -6.08 | 0.000385 |
| CHST11 | 50515 | chr12 | 104601400 | 104605799 | high | 11.00 | 0.000483 |
| CHST11 | 50515 | chr12 | 104708200 | 104714199 | high | 15.00 | 0.000482 |
| CHST2 | 9435 | chr3 | 143119000 | 143121399 | low | -10.43 | 0.000066 |
| CHST3 | 9469 | chr10 | 72008600 | 72011999 | high | 8.50 | 0.000131 |
| CHST3 | 9469 | chr10 | 71978000 | 71984199 | high | 15.50 | 0.000132 |
| CHSY1 | 22856 | chr15 | 101237600 | 101242199 | high | 11.50 | 0.000226 |
| CHSY1 | 22856 | chr15 | 101221200 | 101228799 | high | 19.00 | 0.000226 |
| CHSY3 | 337876 | chr5 | 129969200 | 129971599 | high | 6.00 | 0.002600 |
| CHUK | 1147 | chr10 | 100214200 | 100218599 | high | 11.00 | 0.000011 |
| CHURC1 | 91612 | chr14 | 64916800 | 64922399 | high | 14.00 | 0.001411 |
| CIART | 148523 | chr1 | 150282200 | 150287399 | high | 13.00 | 0.000988 |
| CIB1 | 10519 | chr15 | 90261600 | 90266599 | high | 12.50 | 0.000117 |
| CINP | 51550 | chr14 | 102358200 | 102364999 | high | 17.00 | 0.000504 |
| CIPC | 85457 | chr14 | 77115800 | 77117999 | low | -9.56 | 0.001108 |
| CIPC | 85457 | chr14 | 77097400 | 77100199 | high | 7.00 | 0.001108 |
| CIR1 | 9541 | chr2 | 174380600 | 174382399 | low | -7.82 | 0.000055 |
| CIR1 | 9541 | chr2 | 174394800 | 174396399 | low | -6.95 | 0.000055 |
| CISD1 | 55847 | chr10 | 58265200 | 58269399 | high | 10.50 | 0.000958 |
| CIT | 11113 | chr12 | 119818800 | 119824599 | high | 14.50 | 0.000093 |
| CIZ1 | 25792 | chr9 | 128201600 | 128204999 | high | 8.50 | 0.000201 |
| CKAP2L | 150468 | chr2 | 112747000 | 112750199 | high | 8.00 | 0.001335 |
| CKAP2L | 150468 | chr2 | 112736400 | 112739799 | high | 8.50 | 0.001335 |
| CKAP4 | 10970 | chr12 | 106245200 | 106248399 | high | 8.00 | 0.000103 |
| CKB | 1152 | chr14 | 103519000 | 103524199 | high | 13.00 | 0.000011 |
| CKLF | 51192 | chr16 | 66558600 | 66560599 | low | -8.69 | 0.000769 |

| | | | | | | | |
|---|---|---|---|---|---|---|---|
| CKS1B | 1163 | chr1 | 154966800 | 154978599 | high | 29.50 | 0.000008 |
| CKS2 | 1164 | chr9 | 89308800 | 89315999 | high | 18.00 | 0.000013 |
| CLASRP | 11129 | chr19 | 45067000 | 45070999 | high | 10.00 | 0.000247 |
| CLCC1 | 23155 | chr1 | 108934600 | 108938999 | high | 11.00 | 0.000213 |
| CLCC1 | 23155 | chr1 | 108946800 | 108951799 | high | 12.50 | 0.000213 |
| CLCF1 | 23529 | chr11 | 67364400 | 67369599 | high | 13.00 | 0.000349 |
| CLCN2 | 1181 | chr3 | 184361000 | 184364799 | high | 9.50 | 0.000006 |
| CLCN6 | 1185 | chr1 | 11805000 | 11806999 | high | 5.00 | 0.000100 |
| CLCNKB | 1188 | chr1 | 16048800 | 16054999 | high | 15.50 | 0.000074 |
| CLDN1 | 9076 | chr3 | 190309200 | 190312999 | low | -16.50 | 0.000048 |
| CLDN11 | 5010 | chr3 | 170416800 | 170421599 | high | 12.00 | 0.000029 |
| CLDN14 | 23562 | chr21 | 36500400 | 36505399 | high | 12.50 | 0.000646 |
| CLDN20 | 49861 | chr6 | 155256800 | 155265599 | high | 22.00 | 0.000321 |
| CLDN7 | 1366 | chr17 | 7255200 | 7261199 | high | 15.00 | 0.000188 |
| CLIC4 | 25932 | chr1 | 24751800 | 24754399 | high | 6.50 | 0.001048 |
| CLIC4 | 25932 | chr1 | 24772600 | 24776799 | high | 10.50 | 0.001047 |
| CLIC4 | 25932 | chr1 | 24765000 | 24771199 | high | 15.50 | 0.001047 |
| CLINT1 | 9685 | chr5 | 157858000 | 157859799 | high | 4.50 | 0.000061 |
| CLIP1 | 6249 | chr12 | 122385400 | 122388999 | high | 9.00 | 0.000051 |
| CLIP1 | 6249 | chr12 | 122409000 | 122414999 | high | 15.00 | 0.000051 |
| CLIP1 | 6249 | chr12 | 122416400 | 122425399 | high | 22.50 | 0.000051 |
| CLIP3 | 25999 | chr19 | 36031800 | 36034599 | high | 7.00 | 0.000722 |
| CLIP3 | 25999 | chr19 | 36018800 | 36024399 | high | 14.00 | 0.000722 |
| CLIP4 | 79745 | chr2 | 29115600 | 29117799 | high | 5.50 | 0.002739 |
| CLMP | 79827 | chr11 | 123080600 | 123084999 | high | 11.00 | 0.000649 |
| CLMP | 79827 | chr11 | 123181800 | 123186799 | high | 12.50 | 0.000648 |
| CLMP | 79827 | chr11 | 123151200 | 123158199 | high | 17.50 | 0.000648 |
| CLN3 | 1201 | chr16 | 28490000 | 28492599 | low | -11.30 | 0.000042 |
| CLN6 | 54982 | chr15 | 68217000 | 68222399 | high | 13.50 | 0.000806 |
| CLNK | 116449 | chr4 | 10644800 | 10647599 | low | -12.17 | 0.010940 |
| CLOCK | 9575 | chr4 | 55445800 | 55448999 | high | 8.00 | 0.000173 |
| CLOCK | 9575 | chr4 | 55491400 | 55495399 | high | 10.00 | 0.000173 |
| CLPB | 81570 | chr11 | 72297800 | 72303399 | high | 14.00 | 0.001128 |
| CLPB | 81570 | chr11 | 72380400 | 72386199 | high | 14.50 | 0.001127 |
| CLPP | 8192 | chr19 | 6363400 | 6369199 | high | 14.50 | 0.001287 |
| CLPTM1 | 1209 | chr19 | 44987400 | 44990599 | high | 8.00 | 0.000027 |
| CLSTN1 | 22883 | chr1 | 9821600 | 9825999 | high | 11.00 | 0.002330 |
| CLSTN3 | 9746 | chr12 | 7139000 | 7141199 | high | 5.50 | 0.001365 |
| CLTA | 1211 | chr9 | 36206800 | 36210999 | high | 10.50 | 0.000033 |
| CLTC | 1213 | chr17 | 59625800 | 59628999 | high | 8.00 | 0.000020 |
| CLTC | 1213 | chr17 | 59692400 | 59696199 | high | 9.50 | 0.000020 |
| CLTC | 1213 | chr17 | 59618800 | 59622599 | high | 9.50 | 0.000020 |
| CLTC | 1213 | chr17 | 59676000 | 59679999 | high | 10.00 | 0.000020 |
| CLTC | 1213 | chr17 | 59644600 | 59650199 | high | 14.00 | 0.000020 |
| CLTCL1 | 8218 | chr22 | 19290200 | 19293199 | high | 7.50 | 0.000426 |
| CLTCL1 | 8218 | chr22 | 19176600 | 19183199 | high | 16.50 | 0.000429 |
| CLUAP1 | 23059 | chr16 | 3518200 | 3520999 | high | 7.00 | 0.006554 |
| CLUAP1 | 23059 | chr16 | 3500200 | 3504599 | high | 11.00 | 0.006588 |
| CLUH | 23277 | chr17 | 2706400 | 2708599 | high | 5.50 | 0.008601 |
| CLUH | 23277 | chr17 | 2709400 | 2712199 | high | 7.00 | 0.008591 |
| CLUL1 | 27098 | chr18 | 615800 | 621399 | high | 14.00 | 0.004889 |
| CMBL | 134147 | chr5 | 10304400 | 10306799 | high | 6.00 | 0.013018 |
| CMBL | 134147 | chr5 | 10284400 | 10289999 | high | 14.00 | 0.013044 |
| CMC2 | 56942 | chr16 | 80998000 | 80999599 | high | 4.00 | 0.000703 |
| CMSS1 | 84319 | chr3 | 99861200 | 99864399 | high | 8.00 | 0.000844 |
| CMSS1 | 84319 | chr3 | 99867800 | 99871799 | high | 10.00 | 0.000844 |
| CMSS1 | 84319 | chr3 | 99817000 | 99825199 | high | 20.50 | 0.000845 |
| CMTM3 | 123920 | chr16 | 66603400 | 66605399 | low | -8.69 | 0.001861 |
| CMTM4 | 146223 | chr16 | 66616400 | 66618999 | low | -11.30 | 0.002195 |
| CMTM6 | 54918 | chr3 | 32501200 | 32504199 | high | 7.50 | 0.001690 |
| CMTM7 | 112616 | chr3 | 32452000 | 32456999 | high | 12.50 | 0.003470 |
| CMTM7 | 112616 | chr3 | 32425000 | 32430799 | high | 14.50 | 0.003473 |
| CMTM7 | 112616 | chr3 | 32396400 | 32403199 | high | 17.00 | 0.003476 |
| CNDP2 | 55748 | chr18 | 74495400 | 74497799 | high | 6.00 | 0.000748 |
| CNGB1 | 1258 | chr16 | 57887600 | 57891199 | high | 9.00 | 0.000022 |
| CNKSR3 | 154043 | chr6 | 154488800 | 154492399 | high | 9.00 | 0.000997 |
| CNN3 | 1266 | chr1 | 94924600 | 94929599 | low | -21.76 | 0.000013 |
| CNNM3 | 26505 | chr2 | 96816000 | 96818999 | high | 7.50 | 0.000274 |
| CNOT1 | 23019 | chr16 | 58520400 | 58524399 | high | 10.00 | 0.000393 |
| CNOT1 | 23019 | chr16 | 58578400 | 58583199 | high | 12.00 | 0.000393 |
| CNOT1 | 23019 | chr16 | 58547800 | 58552999 | high | 13.00 | 0.000393 |
| CNOT10 | 25904 | chr3 | 32684400 | 32687199 | high | 7.00 | 0.000793 |
| CNOT2 | 4848 | chr12 | 70245600 | 70248199 | high | 6.50 | 0.000069 |
| CNOT4 | 4850 | chr7 | 135507600 | 135510599 | high | 7.50 | 0.000036 |
| CNOT4 | 4850 | chr7 | 135402600 | 135408799 | high | 15.50 | 0.000036 |
| CNOT6L | 246175 | chr4 | 77817600 | 77820199 | high | 6.50 | 0.003163 |
| CNOT9 | 9125 | chr2 | 218566600 | 218574599 | high | 20.00 | 0.000042 |
| CNPPD1 | 27013 | chr2 | 219173800 | 219177199 | high | 8.50 | 0.000123 |

| | | | | | | | |
|---|---|---|---|---|---|---|---|
| CNPY3 | 10695 | chr6 | 42930400 | 42934599 | high | 10.50 | 0.000249 |
| CNRIP1 | 25927 | chr2 | 68312400 | 68315799 | high | 8.50 | 0.000380 |
| CNST | 163882 | chr1 | 246593600 | 246597199 | high | 9.00 | 0.000665 |
| CNTD1 | 124817 | chr17 | 42792400 | 42798999 | high | 16.50 | 0.002917 |
| CNTRL | 11064 | chr9 | 121120400 | 121124999 | high | 11.50 | 0.000091 |
| COA1 | 55744 | chr7 | 43654000 | 43657399 | high | 8.50 | 0.001277 |
| COA1 | 55744 | chr7 | 43660000 | 43663599 | high | 9.00 | 0.001277 |
| COA1 | 55744 | chr7 | 43639200 | 43642799 | high | 9.00 | 0.001277 |
| COA1 | 55744 | chr7 | 43691800 | 43696599 | high | 12.00 | 0.001276 |
| COA3 | 28958 | chr17 | 42792400 | 42798999 | high | 16.50 | 0.000677 |
| COA5 | 493753 | chr2 | 98607000 | 98609199 | high | 5.50 | 0.005007 |
| COA6 | 388753 | chr1 | 234379200 | 234382599 | low | -14.78 | 0.001659 |
| COA7 | 65260 | chr1 | 52694600 | 52698599 | high | 10.00 | 0.001238 |
| COG1 | 9382 | chr17 | 73191800 | 73197399 | high | 14.00 | 0.000128 |
| COG3 | 83548 | chr13 | 45518000 | 45519799 | low | -7.82 | 0.001835 |
| COG4 | 25839 | chr16 | 70522200 | 70524599 | high | 6.00 | 0.000366 |
| COG7 | 91949 | chr16 | 23452000 | 23452799 | low | -3.47 | 0.003921 |
| COG8 | 84342 | chr16 | 69326400 | 69334399 | high | 20.00 | 0.001217 |
| COL12A1 | 1303 | chr6 | 75195600 | 75200199 | high | 11.50 | 0.000017 |
| COL13A1 | 1305 | chr10 | 69814000 | 69817399 | high | 8.50 | 0.000019 |
| COL13A1 | 1305 | chr10 | 69807400 | 69810799 | high | 8.50 | 0.000019 |
| COL13A1 | 1305 | chr10 | 69836000 | 69840999 | high | 12.50 | 0.000019 |
| COL16A1 | 1307 | chr1 | 31661600 | 31666799 | high | 13.00 | 0.000041 |
| COL17A1 | 1308 | chr10 | 104058000 | 104063799 | high | 14.50 | 0.000013 |
| COL18A1 | 80781 | chr21 | 45445200 | 45447999 | high | 7.00 | 0.001778 |
| COL18A1 | 80781 | chr21 | 45479800 | 45483399 | high | 9.00 | 0.001776 |
| COL18A1 | 80781 | chr21 | 45505600 | 45509599 | high | 10.00 | 0.001775 |
| COL1A1 | 1277 | chr17 | 50199000 | 50213399 | low | -6.83 | 0.000353 |
| COL21A1 | 81578 | chr6 | 56255400 | 56258599 | high | 8.00 | 0.001450 |
| COL21A1 | 81578 | chr6 | 56347200 | 56350599 | high | 8.50 | 0.001448 |
| COL21A1 | 81578 | chr6 | 56240400 | 56244199 | high | 9.50 | 0.001451 |
| COL21A1 | 81578 | chr6 | 56352800 | 56357799 | high | 12.50 | 0.001448 |
| COL21A1 | 81578 | chr6 | 56363400 | 56368799 | high | 13.50 | 0.001447 |
| COL21A1 | 81578 | chr6 | 56370200 | 56375999 | high | 14.50 | 0.001447 |
| COL21A1 | 81578 | chr6 | 56310200 | 56316799 | high | 16.50 | 0.001449 |
| COL27A1 | 85301 | chr9 | 114215400 | 114220799 | high | 13.50 | 0.000747 |
| COL28A1 | 340267 | chr7 | 7490600 | 7493599 | high | 7.50 | 0.005047 |
| COL5A2 | 1290 | chr2 | 189121000 | 189124599 | high | 9.00 | 0.000007 |
| COL5A2 | 1290 | chr2 | 189102600 | 189106399 | high | 9.50 | 0.000007 |
| COL5A2 | 1290 | chr2 | 189037800 | 189041799 | high | 10.00 | 0.000007 |
| COL5A2 | 1290 | chr2 | 189129200 | 189134799 | high | 14.00 | 0.000007 |
| COL5A2 | 1290 | chr2 | 189112000 | 189119599 | high | 19.00 | 0.000007 |
| COL5A2 | 1290 | chr2 | 189135800 | 189145599 | high | 24.50 | 0.000007 |
| COL6A3 | 1293 | chr2 | 237331800 | 237335799 | high | 10.00 | 0.000005 |
| COL6A3 | 1293 | chr2 | 237380200 | 237384999 | high | 12.00 | 0.000005 |
| COPS7B | 64708 | chr2 | 231778400 | 231787999 | low | -41.73 | 0.000279 |
| COPS8 | 10920 | chr2 | 237085000 | 237086199 | high | 3.00 | 0.000046 |
| COPZ1 | 22818 | chr12 | 54324400 | 54332399 | high | 20.00 | 0.000420 |
| COPZ2 | 51226 | chr17 | 48036800 | 48038599 | low | -7.82 | 0.001066 |
| COQ10A | 93058 | chr12 | 56266600 | 56269199 | high | 6.50 | 0.001654 |
| COQ10B | 80219 | chr2 | 197453200 | 197454599 | low | -6.08 | 0.000406 |
| COQ2 | 27235 | chr4 | 83274400 | 83276399 | low | -8.69 | 0.000327 |
| COQ3 | 51805 | chr6 | 99392200 | 99395399 | high | 8.00 | 0.000521 |
| COQ4 | 51117 | chr9 | 128320000 | 128325399 | high | 13.50 | 0.000398 |
| COQ6 | 51004 | chr14 | 73950000 | 73951999 | low | -8.69 | 0.000690 |
| COQ7 | 10229 | chr16 | 19065800 | 19068399 | low | -11.30 | 0.000537 |
| COQ8B | 79934 | chr19 | 40711400 | 40718199 | high | 17.00 | 0.001963 |
| COQ9 | 57017 | chr16 | 57458800 | 57461799 | low | -13.04 | 0.000992 |
| CORIN | 10699 | chr4 | 47733600 | 47734999 | low | -6.08 | 0.000224 |
| CORO1B | 57175 | chr11 | 67442200 | 67444399 | low | -9.56 | 0.000848 |
| COTL1 | 23406 | chr16 | 84614200 | 84617799 | high | 9.00 | 0.000277 |
| COTL1 | 23406 | chr16 | 84575600 | 84579599 | high | 10.00 | 0.000277 |
| COTL1 | 23406 | chr16 | 84563400 | 84567999 | high | 11.50 | 0.000277 |
| COX10 | 1352 | chr17 | 14126200 | 14129399 | low | -13.91 | 0.000096 |
| COX10 | 1352 | chr17 | 14118200 | 14121399 | low | -13.91 | 0.000096 |
| COX15 | 1355 | chr10 | 99731800 | 99734799 | high | 7.50 | 0.000014 |
| COX18 | 285521 | chr4 | 73065600 | 73071199 | high | 14.00 | 0.003908 |
| COX5A | 9377 | chr15 | 74937000 | 74937999 | low | -4.34 | 0.000125 |
| CP | 1356 | chr3 | 149168000 | 149170799 | high | 7.00 | 0.000009 |
| CP | 1356 | chr3 | 149192200 | 149196799 | high | 11.50 | 0.000009 |
| CPA2 | 1358 | chr7 | 130275600 | 130278999 | high | 8.50 | 0.000010 |
| CPA4 | 51200 | chr7 | 130309000 | 130314399 | high | 13.50 | 0.000393 |
| CPA5 | 93979 | chr7 | 130356800 | 130361199 | high | 11.00 | 0.000721 |
| CPD | 1362 | chr17 | 30392400 | 30396799 | high | 11.00 | 0.000045 |
| CPD | 1362 | chr17 | 30411000 | 30415999 | high | 12.50 | 0.000045 |
| CPEB2 | 132864 | chr4 | 15066400 | 15068199 | high | 4.50 | 0.008819 |
| CPEB2 | 132864 | chr4 | 15054600 | 15059399 | high | 12.00 | 0.008825 |
| CPEB2 | 132864 | chr4 | 15023400 | 15029199 | high | 14.50 | 0.008844 |

| | | | | | | | |
|---|---|---|---|---|---|---|---|
| CPEB3 | 22849 | chr10 | 92288800 | 92292199 | high | 8.50 | 0.000248 |
| CPEB4 | 80315 | chr5 | 173888400 | 173890199 | low | -7.82 | 0.000462 |
| CPED1 | 79974 | chr7 | 121174400 | 121176999 | high | 6.50 | 0.000660 |
| CPED1 | 79974 | chr7 | 121184400 | 121187599 | high | 8.00 | 0.000660 |
| CPED1 | 79974 | chr7 | 121015600 | 121019199 | high | 9.00 | 0.000661 |
| CPED1 | 79974 | chr7 | 121043600 | 121047599 | high | 10.00 | 0.000661 |
| CPED1 | 79974 | chr7 | 120993600 | 120997599 | high | 10.00 | 0.000661 |
| CPED1 | 79974 | chr7 | 121061600 | 121065999 | high | 11.00 | 0.000661 |
| CPED1 | 79974 | chr7 | 121088200 | 121093199 | high | 12.50 | 0.000660 |
| CPED1 | 79974 | chr7 | 121125800 | 121136799 | high | 27.50 | 0.000660 |
| CPNE1 | 8904 | chr20 | 35630800 | 35633399 | high | 6.50 | 0.000250 |
| CPNE3 | 8895 | chr8 | 86506400 | 86515399 | high | 22.50 | 0.000103 |
| CPNE4 | 131034 | chr3 | 131911600 | 131915199 | high | 9.00 | 0.000993 |
| CPNE7 | 27132 | chr16 | 89585400 | 89590599 | high | 13.00 | 0.000303 |
| CPQ | 10404 | chr8 | 97127800 | 97130199 | low | -10.43 | 0.000107 |
| CPQ | 10404 | chr8 | 96811800 | 96813799 | low | -8.69 | 0.000107 |
| CPQ | 10404 | chr8 | 96806400 | 96808199 | low | -7.82 | 0.000107 |
| CPS1 | 1373 | chr2 | 210474000 | 210477799 | high | 9.50 | 0.000007 |
| CPSF1 | 29894 | chr8 | 144405600 | 144410799 | high | 13.00 | 0.000207 |
| CPSF2 | 53981 | chr14 | 92119600 | 92123199 | high | 9.00 | 0.000586 |
| CPSF3L | 54973 | chr1 | 1313200 | 1316599 | high | 8.50 | 0.004651 |
| CPSF6 | 11052 | chr12 | 69239000 | 69240599 | high | 4.00 | 0.000160 |
| CPT1A | 1374 | chr11 | 68765200 | 68767799 | low | -11.30 | 0.000020 |
| CPT1C | 126129 | chr19 | 49690000 | 49694199 | high | 10.50 | 0.002538 |
| CUX1 | 1523 | chr7 | 102044000 | 102051599 | high | 19.00 | 0.000015 |
| CWC15 | 51503 | chr11 | 94972200 | 94974599 | low | -10.43 | 0.000542 |
| CWC22 | 57703 | chr2 | 180000000 | 180003999 | high | 10.00 | 0.000321 |
| CWC27 | 10283 | chr5 | 64847600 | 64850999 | high | 8.50 | 0.000159 |
| CWC27 | 10283 | chr5 | 64841400 | 64846199 | high | 12.00 | 0.000159 |
| CWC27 | 10283 | chr5 | 64835200 | 64839999 | high | 12.00 | 0.000159 |
| CWC27 | 10283 | chr5 | 64822800 | 64827999 | high | 13.00 | 0.000159 |
| CWC27 | 10283 | chr5 | 64868400 | 64874399 | high | 15.00 | 0.000159 |
| CXCL1 | 2919 | chr4 | 73869000 | 73870599 | low | -6.95 | 0.000040 |
| CXCL3 | 2921 | chr4 | 74036600 | 74039599 | low | -13.04 | 0.000039 |
| CXCL8 | 3576 | chr4 | 73740000 | 73743599 | low | -15.65 | 0.000048 |
| CXCL9 | 4283 | chr4 | 76002200 | 76004199 | low | -8.69 | 0.000056 |
| CYB5B | 80777 | chr16 | 69464400 | 69467799 | high | 8.50 | 0.001163 |
| CYTH3 | 9265 | chr7 | 6268600 | 6271399 | low | -12.17 | 0.001478 |
| D2HGDH | 728294 | chr2 | 241734400 | 241735999 | high | 4.00 | 0.003013 |
| DAAM1 | 23002 | chr14 | 59341000 | 59345199 | high | 10.50 | 0.000388 |
| DAAM1 | 23002 | chr14 | 59324600 | 59329399 | high | 12.00 | 0.000388 |
| DAAM1 | 23002 | chr14 | 59187000 | 59192199 | high | 13.00 | 0.000389 |
| DAAM1 | 23002 | chr14 | 59278200 | 59284599 | high | 16.00 | 0.000388 |
| DAB2 | 1601 | chr5 | 39409200 | 39413599 | high | 11.00 | 0.000041 |
| DAB2 | 1601 | chr5 | 39389600 | 39394399 | high | 12.00 | 0.000041 |
| DAD1 | 1603 | chr14 | 22588200 | 22589199 | low | -4.34 | 0.000071 |
| DAG1 | 1605 | chr3 | 49465400 | 49472199 | high | 17.00 | 0.000032 |
| DAGLB | 221955 | chr7 | 6444800 | 6448199 | high | 8.50 | 0.003827 |
| DANCR | 57291 | chr4 | 52712200 | 52713399 | low | -5.21 | 0.001087 |
| DAPK2 | 23604 | chr15 | 64008800 | 64011999 | low | -13.91 | 0.000369 |
| DAPK3 | 1613 | chr19 | 3968800 | 3971799 | high | 7.50 | 0.000406 |
| DARS | 1615 | chr2 | 135983200 | 135987199 | high | 10.00 | 0.000012 |
| DARS2 | 55157 | chr1 | 173833000 | 173834199 | low | -5.21 | 0.000317 |
| DAZAP2 | 9802 | chr12 | 51238000 | 51239599 | low | -6.95 | 0.000191 |
| DBF4B | 80174 | chr17 | 44708000 | 44710799 | high | 7.00 | 0.001793 |
| DBIL5P | 100131454 | chr17 | 751600 | 754199 | low | -11.30 | 0.013457 |
| DBT | 1629 | chr1 | 100229200 | 100233399 | high | 10.50 | 0.000016 |
| DCAF11 | 80344 | chr14 | 24113400 | 24120799 | high | 18.50 | 0.003332 |
| DCAF12 | 25853 | chr9 | 34124200 | 34126799 | low | -11.30 | 0.000758 |
| DCAF13 | 25879 | chr8 | 103418400 | 103422199 | high | 9.50 | 0.000250 |
| DCAF15 | 90379 | chr19 | 13949600 | 13954799 | high | 13.00 | 0.006479 |
| DCAF17 | 80067 | chr2 | 171468400 | 171471799 | high | 8.50 | 0.000467 |
| DCAF4 | 26094 | chr14 | 72943800 | 72948399 | high | 11.50 | 0.000358 |
| DCAF4 | 26094 | chr14 | 72925200 | 72931199 | high | 15.00 | 0.000358 |
| DCAF5 | 8816 | chr14 | 69111800 | 69115199 | high | 8.50 | 0.000128 |
| DCAF5 | 8816 | chr14 | 69090400 | 69095199 | high | 12.00 | 0.000128 |
| DCAF5 | 8816 | chr14 | 69127600 | 69133399 | high | 14.50 | 0.000128 |
| DCAF6 | 55827 | chr1 | 167934800 | 167938799 | high | 10.00 | 0.000332 |
| DCAF6 | 55827 | chr1 | 167979000 | 167983199 | high | 10.50 | 0.000332 |
| DCAF6 | 55827 | chr1 | 168010600 | 168015199 | high | 11.50 | 0.000332 |
| DCAF7 | 10238 | chr17 | 63557600 | 63560999 | low | -14.78 | 0.000161 |
| DCAF7 | 10238 | chr17 | 63554600 | 63556799 | low | -9.56 | 0.000161 |
| DCAF8 | 50717 | chr1 | 160261400 | 160262399 | low | -4.34 | 0.000316 |
| DCBLD1 | 285761 | chr6 | 117513800 | 117517399 | high | 9.00 | 0.002432 |
| DCBLD1 | 285761 | chr6 | 117527600 | 117531399 | high | 9.50 | 0.002431 |
| DCBLD1 | 285761 | chr6 | 117509200 | 117512999 | high | 9.50 | 0.002432 |
| DCBLD1 | 285761 | chr6 | 117547000 | 117552399 | high | 13.50 | 0.002431 |
| DCBLD2 | 131566 | chr3 | 98806800 | 98810399 | high | 9.00 | 0.001332 |

| | | | | | | | |
|---|---|---|---|---|---|---|---|
| DCBLD2 | 131566 | chr3 | 98842400 | 98847399 | high | 12.50 | 0.001331 |
| DCDC1 | 341019 | chr11 | 31369400 | 31372199 | high | 7.00 | 0.010871 |
| DCDC2B | 149069 | chr1 | 32207000 | 32212199 | high | 13.00 | 0.004628 |
| DCHS1 | 8642 | chr11 | 6648200 | 6651199 | high | 7.50 | 0.001300 |
| DCHS1 | 8642 | chr11 | 6615200 | 6622599 | high | 18.50 | 0.001306 |
| DCK | 1633 | chr4 | 70993000 | 70994399 | low | -6.08 | 0.000023 |
| DCLK2 | 166614 | chr4 | 150100400 | 150103999 | high | 9.00 | 0.001110 |
| DCLK2 | 166614 | chr4 | 150124000 | 150128599 | high | 11.50 | 0.001110 |
| DCN | 1634 | chr12 | 91146000 | 91147999 | low | -8.69 | 0.000018 |
| DCN | 1634 | chr12 | 91178200 | 91179399 | low | -5.21 | 0.000018 |
| DCP1A | 55802 | chr3 | 53327000 | 53331799 | high | 12.00 | 0.001046 |
| DCP1B | 196513 | chr12 | 1962600 | 1965599 | high | 7.50 | 0.011125 |
| DCP1B | 196513 | chr12 | 1978000 | 1982599 | high | 11.50 | 0.011039 |
| DCP1B | 196513 | chr12 | 1992800 | 1998799 | high | 15.00 | 0.010957 |
| DCPS | 28960 | chr11 | 126317200 | 126319999 | low | -12.17 | 0.000229 |
| DCTN1 | 1639 | chr2 | 74390400 | 74392599 | high | 5.50 | 0.000022 |
| DCTN2 | 10540 | chr12 | 57545800 | 57547799 | high | 5.00 | 0.000183 |
| DCTN2 | 10540 | chr12 | 57539200 | 57544799 | high | 14.00 | 0.000183 |
| DCTN4 | 51164 | chr5 | 150721000 | 150724799 | high | 9.50 | 0.000339 |
| DCTN6 | 10671 | chr8 | 30154000 | 30158799 | high | 12.00 | 0.000354 |
| DDA1 | 79016 | chr19 | 17308600 | 17314199 | high | 14.00 | 0.004565 |
| DDB2 | 1643 | chr11 | 47214000 | 47216999 | high | 7.50 | 0.000035 |
| DDHD1 | 80821 | chr14 | 53050400 | 53055599 | high | 13.00 | 0.001523 |
| DDI2 | 84301 | chr1 | 15616800 | 15619999 | low | -13.91 | 0.005398 |
| DDIAS | 220042 | chr11 | 82901200 | 82903999 | high | 7.00 | 0.002654 |
| DDIT4 | 54541 | chr10 | 72273600 | 72274799 | low | -5.21 | 0.000755 |
| DDN | 23109 | chr12 | 48997000 | 49000399 | high | 8.50 | 0.000472 |
| DDOST | 1650 | chr1 | 20659400 | 20663399 | high | 10.00 | 0.000080 |
| DDR2 | 4921 | chr1 | 162709200 | 162713399 | high | 10.50 | 0.000030 |
| DDR2 | 4921 | chr1 | 162686200 | 162692599 | high | 16.00 | 0.000030 |
| DDR2 | 4921 | chr1 | 162679000 | 162685399 | high | 16.00 | 0.000030 |
| DDR2 | 4921 | chr1 | 162719000 | 162726799 | high | 19.50 | 0.000030 |
| DDRGK1 | 65992 | chr20 | 3188400 | 3192599 | high | 10.50 | 0.002300 |
| DEAF1 | 10522 | chr11 | 694600 | 696999 | high | 6.00 | 0.015148 |
| DEK | 7913 | chr6 | 18262600 | 18265399 | high | 7.00 | 0.000433 |
| DEPDC4 | 120863 | chr12 | 100266800 | 100268399 | low | -6.95 | 0.001205 |
| DEPDC5 | 9681 | chr22 | 31870000 | 31874999 | high | 12.50 | 0.000304 |
| DERA | 51071 | chr12 | 15969600 | 15974799 | high | 13.00 | 0.003198 |
| DESI2 | 51029 | chr1 | 244651800 | 244654399 | high | 6.50 | 0.000209 |
| DEXI | 28955 | chr16 | 10937200 | 10944799 | high | 19.00 | 0.002647 |
| DGCR6L | 85359 | chr22 | 20319200 | 20320199 | low | -4.34 | 0.004201 |
| DGCR8 | 54487 | chr22 | 20079800 | 20081399 | high | 4.00 | 0.002714 |
| DGKB | 1607 | chr7 | 14149400 | 14151799 | low | -10.43 | 0.000114 |
| DGKD | 8527 | chr2 | 233411400 | 233415599 | high | 10.50 | 0.000037 |
| DGKE | 8526 | chr17 | 56830400 | 56835599 | high | 13.00 | 0.000150 |
| DGKI | 9162 | chr7 | 137589600 | 137592399 | high | 7.00 | 0.000067 |
| DGKI | 9162 | chr7 | 137678800 | 137682799 | high | 10.00 | 0.000067 |
| DGKI | 9162 | chr7 | 137761800 | 137766599 | high | 12.00 | 0.000067 |
| DGKZ | 8525 | chr11 | 46352000 | 46355599 | high | 9.00 | 0.000184 |
| DGKZ | 8525 | chr11 | 46367600 | 46372399 | high | 12.00 | 0.000184 |
| DGUOK | 1716 | chr2 | 73949400 | 73954199 | high | 12.00 | 0.000023 |
| DGUOK | 1716 | chr2 | 73958400 | 73964199 | high | 14.50 | 0.000023 |
| DHCR24 | 1718 | chr1 | 54863000 | 54868199 | high | 13.00 | 0.000031 |
| DHDDS | 79947 | chr1 | 26468200 | 26472599 | high | 11.00 | 0.003020 |
| DHFR | 1719 | chr5 | 80652400 | 80656799 | low | -19.60 | 0.000021 |
| DHH | 50846 | chr12 | 49091200 | 49095999 | high | 12.00 | 0.001036 |
| DHPS | 1725 | chr19 | 12675200 | 12680199 | high | 12.50 | 0.000136 |
| DHRS1 | 115817 | chr14 | 24299000 | 24302199 | high | 8.00 | 0.004766 |
| DHRS13 | 147015 | chr17 | 28901000 | 28903399 | high | 6.00 | 0.005087 |
| DHTKD1 | 55526 | chr10 | 12069000 | 12070199 | low | -5.21 | 0.004601 |
| DHX15 | 1665 | chr4 | 24582400 | 24584999 | low | -11.30 | 0.000068 |
| DHX29 | 54505 | chr5 | 55292400 | 55294799 | low | -10.43 | 0.000986 |
| DHX29 | 54505 | chr5 | 55276000 | 55278399 | low | -10.43 | 0.000986 |
| DHX29 | 54505 | chr5 | 55296400 | 55298199 | low | -7.82 | 0.000986 |
| DHX29 | 54505 | chr5 | 55269200 | 55270999 | low | -7.82 | 0.000986 |
| DHX30 | 22907 | chr3 | 47801400 | 47806599 | high | 13.00 | 0.000479 |
| DHX33 | 56919 | chr17 | 5466600 | 5471199 | high | 11.50 | 0.010412 |
| DHX34 | 9704 | chr19 | 47347400 | 47352399 | high | 12.50 | 0.000205 |
| DHX35 | 60625 | chr20 | 38962000 | 38963999 | low | -8.69 | 0.001556 |
| DHX57 | 90957 | chr2 | 38833800 | 38838999 | high | 13.00 | 0.002342 |
| DHX58 | 79132 | chr17 | 42111600 | 42118799 | high | 18.00 | 0.001879 |
| DHX8 | 1659 | chr17 | 43492200 | 43495799 | high | 9.00 | 0.000038 |
| DHX9 | 1660 | chr1 | 182838600 | 182840599 | high | 5.00 | 0.000009 |
| DIABLO | 56616 | chr12 | 122225200 | 122228399 | high | 8.00 | 0.000463 |
| DIAPH1 | 1729 | chr5 | 141524600 | 141528599 | high | 10.00 | 0.000012 |
| DIAPH1 | 1729 | chr5 | 141616000 | 141620199 | high | 10.50 | 0.000012 |
| DIAPH2 | 1730 | chrX | 96929200 | 96934199 | high | 12.50 | 0.000018 |
| DICER1 | 23405 | chr14 | 95140800 | 95144999 | low | -18.21 | 0.000246 |

| | | | | | | | |
|---|---|---|---|---|---|---|---|
| DICER1 | 23405 | chr14 | 95123000 | 95125999 | low | -13.04 | 0.000246 |
| DIDO1 | 11083 | chr20 | 62936600 | 62939999 | high | 8.50 | 0.000176 |
| DIDO1 | 11083 | chr20 | 62906400 | 62910999 | high | 11.50 | 0.000176 |
| DIMT1 | 27292 | chr5 | 62401000 | 62403999 | high | 7.50 | 0.000437 |
| DIP2A | 23181 | chr21 | 46531200 | 46535599 | high | 11.00 | 0.000498 |
| DIP2A | 23181 | chr21 | 46497800 | 46502999 | high | 13.00 | 0.000499 |
| DIP2B | 57609 | chr12 | 50558600 | 50563399 | high | 12.00 | 0.001139 |
| DIP2B | 57609 | chr12 | 50721000 | 50726399 | high | 13.50 | 0.001136 |
| DIP2B | 57609 | chr12 | 50544000 | 50549599 | high | 14.00 | 0.001140 |
| DIP2B | 57609 | chr12 | 50569400 | 50578199 | high | 22.00 | 0.001139 |
| DIP2C | 22982 | chr10 | 498000 | 502199 | high | 10.50 | 0.005128 |
| DIP2C | 22982 | chr10 | 404800 | 411199 | high | 16.00 | 0.005677 |
| DIRC1 | 116093 | chr2 | 188784000 | 188788599 | high | 11.50 | 0.000615 |
| DIRC1 | 116093 | chr2 | 188732200 | 188736799 | high | 11.50 | 0.000615 |
| DIRC1 | 116093 | chr2 | 188769400 | 188780999 | high | 29.00 | 0.000615 |
| DIRC2 | 84925 | chr3 | 122789400 | 122796199 | high | 17.00 | 0.000692 |
| DIS3L2 | 129563 | chr2 | 231999600 | 232001399 | low | -7.82 | 0.000558 |
| DISP1 | 84976 | chr1 | 222814000 | 222818199 | high | 10.50 | 0.000381 |
| DISP2 | 85455 | chr15 | 40360400 | 40362999 | high | 6.50 | 0.002117 |
| DKK2 | 27123 | chr4 | 107002000 | 107004799 | low | -12.17 | 0.000253 |
| DKK3 | 27122 | chr11 | 12002800 | 12005399 | high | 6.50 | 0.002260 |
| DKK3 | 27122 | chr11 | 11963600 | 11966199 | high | 6.50 | 0.002267 |
| DKK3 | 27122 | chr11 | 11976400 | 11984399 | high | 20.00 | 0.002265 |
| DLAT | 1737 | chr11 | 112055600 | 112059599 | high | 10.00 | 0.000016 |
| DLC1 | 10395 | chr8 | 13491600 | 13494199 | high | 6.50 | 0.000770 |
| DLC1 | 10395 | chr8 | 13111600 | 13116399 | high | 12.00 | 0.000793 |
| DLC1 | 10395 | chr8 | 13097800 | 13102599 | high | 12.00 | 0.000794 |
| DLC1 | 10395 | chr8 | 13498400 | 13503599 | high | 13.00 | 0.000770 |
| DLC1 | 10395 | chr8 | 13507800 | 13514999 | high | 18.00 | 0.000770 |
| DLD | 1738 | chr7 | 107906200 | 107911199 | high | 12.50 | 0.000016 |
| DLEU2 | 8847 | chr13 | 50052600 | 50055199 | high | 6.50 | 0.000177 |
| DLEU2 | 8847 | chr13 | 50003600 | 50006399 | high | 7.00 | 0.000177 |
| DLEU2 | 8847 | chr13 | 50062600 | 50066999 | high | 11.00 | 0.000177 |
| DLEU2 | 8847 | chr13 | 50028400 | 50033199 | high | 12.00 | 0.000177 |
| DLEU2 | 8847 | chr13 | 49994200 | 49999399 | high | 13.00 | 0.000177 |
| DLEU2 | 8847 | chr13 | 50079000 | 50085799 | high | 17.00 | 0.000177 |
| DLEU2L | 79469 | chr1 | 63549400 | 63553399 | high | 10.00 | 0.001251 |
| DLG1 | 1739 | chr3 | 197179600 | 197182599 | high | 7.50 | 0.000009 |
| DLG2 | 1740 | chr11 | 85625600 | 85632599 | high | 17.50 | 0.000020 |
| DLG3 | 1741 | chrX | 70452400 | 70454799 | low | -10.43 | 0.000025 |
| DLG4 | 1742 | chr17 | 7202600 | 7205999 | high | 8.50 | 0.000242 |
| DLG4 | 1742 | chr17 | 7214000 | 7221399 | high | 18.50 | 0.000241 |
| DLGAP1 | 9229 | chr18 | 3673200 | 3677799 | high | 11.50 | 0.002513 |
| DLGAP1 | 9229 | chr18 | 3645000 | 3650199 | high | 13.00 | 0.002532 |
| DLGAP1 | 9229 | chr18 | 3621200 | 3631799 | high | 26.50 | 0.002549 |
| DLGAP4 | 22839 | chr20 | 36517800 | 36522799 | high | 12.50 | 0.000625 |
| DLGAP5 | 9787 | chr14 | 55147000 | 55151999 | high | 12.50 | 0.000177 |
| DLST | 1743 | chr14 | 74894400 | 74898599 | high | 10.50 | 0.000023 |
| DLST | 1743 | chr14 | 74888200 | 74892599 | high | 11.00 | 0.000023 |
| DLX2 | 1746 | chr2 | 172099400 | 172104199 | high | 12.00 | 0.000010 |
| DMC1 | 11144 | chr22 | 38538400 | 38541999 | high | 9.00 | 0.000289 |
| DMPK | 1760 | chr19 | 45763600 | 45772199 | high | 21.50 | 0.000038 |
| DMWD | 1762 | chr19 | 45789400 | 45793199 | high | 9.50 | 0.000038 |
| DMXL1 | 1657 | chr5 | 119071000 | 119073199 | low | -9.56 | 0.000014 |
| DMXL2 | 23312 | chr15 | 51620000 | 51623399 | high | 8.50 | 0.000452 |
| DMXL2 | 23312 | chr15 | 51452200 | 51456199 | high | 10.00 | 0.000453 |
| DMXL2 | 23312 | chr15 | 51484200 | 51488799 | high | 11.50 | 0.000453 |
| DNA2 | 1763 | chr10 | 68469000 | 68473399 | high | 11.00 | 0.000026 |
| DNAAF2 | 55172 | chr14 | 49628000 | 49631199 | high | 8.00 | 0.001112 |
| DNAH1 | 25981 | chr3 | 52317600 | 52319799 | high | 5.50 | 0.000497 |
| DNAH1 | 25981 | chr3 | 52324200 | 52329399 | high | 13.00 | 0.000497 |
| DNAH1 | 25981 | chr3 | 52344600 | 52350599 | high | 15.00 | 0.000496 |
| DNAH2 | 146754 | chr17 | 7757800 | 7761799 | high | 10.00 | 0.018917 |
| DNAH2 | 146754 | chr17 | 7750400 | 7756999 | high | 16.50 | 0.018935 |
| DNAH5 | 1767 | chr5 | 13827600 | 13832799 | high | 13.00 | 0.000128 |
| DNAH6 | 1768 | chr2 | 84674200 | 84678799 | high | 11.50 | 0.000021 |
| DNAH7 | 56171 | chr2 | 195843600 | 195845199 | low | -6.95 | 0.000287 |
| DNAH7 | 56171 | chr2 | 195829600 | 195830399 | low | -3.47 | 0.000287 |
| DNAI1 | 27019 | chr9 | 34456600 | 34463799 | high | 18.00 | 0.000784 |
| DNAJA1 | 3301 | chr9 | 33030400 | 33035999 | high | 14.00 | 0.000100 |
| DNAJA2 | 10294 | chr16 | 46971400 | 46974399 | high | 7.50 | 0.000219 |
| DNAJB1 | 3337 | chr19 | 14515200 | 14519199 | high | 10.00 | 0.000230 |
| DNAJB11 | 51726 | chr3 | 186569400 | 186572999 | high | 9.00 | 0.000277 |
| DNAJB12 | 54788 | chr10 | 72331800 | 72336999 | high | 13.00 | 0.000757 |
| DNM1 | 1759 | chr9 | 128201600 | 128204999 | high | 8.50 | 0.000014 |
| DNM1 | 1759 | chr9 | 128253000 | 128256599 | high | 9.00 | 0.000014 |
| DNM1L | 10059 | chr12 | 32678400 | 32680599 | low | -9.56 | 0.000308 |
| DNM2 | 1785 | chr19 | 10776000 | 10784599 | high | 21.50 | 0.000166 |

| | | | | | | | |
|---|---|---|---|---|---|---|---|
| DNM3 | 26052 | chr1 | 172134200 | 172137799 | high | 9.00 | 0.000151 |
| DNM3 | 26052 | chr1 | 172107200 | 172111399 | high | 10.50 | 0.000151 |
| DOK6 | 220164 | chr18 | 69655400 | 69659599 | high | 10.50 | 0.003161 |
| DOK6 | 220164 | chr18 | 69589400 | 69593599 | high | 10.50 | 0.003164 |
| DOK6 | 220164 | chr18 | 69558200 | 69562599 | high | 11.00 | 0.003165 |
| DOK6 | 220164 | chr18 | 69787000 | 69791599 | high | 11.50 | 0.003155 |
| DOK6 | 220164 | chr18 | 69626600 | 69631399 | high | 12.00 | 0.003162 |
| DOK6 | 220164 | chr18 | 69813400 | 69820799 | high | 18.50 | 0.003154 |
| DOK6 | 220164 | chr18 | 69516600 | 69524199 | high | 19.00 | 0.003167 |
| DPF3 | 8110 | chr14 | 72750800 | 72754399 | high | 9.00 | 0.000111 |
| DPF3 | 8110 | chr14 | 72884200 | 72889199 | high | 12.50 | 0.000111 |
| DPF3 | 8110 | chr14 | 72890200 | 72896799 | high | 16.50 | 0.000111 |
| DPF3 | 8110 | chr14 | 72680000 | 72686799 | high | 17.00 | 0.000112 |
| DPH1 | 1801 | chr17 | 2030000 | 2030999 | low | -4.34 | 0.000887 |
| DPH3 | 285381 | chr3 | 16263600 | 16265999 | high | 6.00 | 0.017547 |
| DPH5 | 51611 | chr1 | 101013600 | 101017599 | low | -17.17 | 0.000511 |
| DPH5 | 51611 | chr1 | 101006800 | 101008799 | low | -8.69 | 0.000511 |
| DPH6 | 89978 | chr15 | 35431800 | 35435599 | high | 9.50 | 0.002539 |
| DPH6 | 89978 | chr15 | 35544000 | 35553199 | high | 23.00 | 0.002531 |
| DPH7 | 92715 | chr9 | 137574200 | 137578399 | high | 10.50 | 0.000674 |
| DPM2 | 8818 | chr9 | 127935600 | 127938799 | high | 8.00 | 0.000069 |
| DPM3 | 54344 | chr1 | 155140000 | 155140999 | high | 2.50 | 0.000350 |
| DPP7 | 29952 | chr9 | 137110800 | 137116399 | high | 14.00 | 0.000218 |
| DPP8 | 54878 | chr15 | 65516800 | 65518399 | high | 4.00 | 0.000838 |
| DPP8 | 54878 | chr15 | 65509200 | 65515799 | high | 16.50 | 0.000838 |
| DPP9 | 91039 | chr19 | 4717600 | 4721599 | high | 10.00 | 0.019298 |
| DRC3 | 83450 | chr17 | 17987200 | 17990799 | high | 9.00 | 0.004639 |
| DRG1 | 4733 | chr22 | 31424000 | 31426199 | high | 5.50 | 0.000151 |
| DSCR3 | 10311 | chr21 | 37258800 | 37262599 | high | 9.50 | 0.000277 |
| DSCR3 | 10311 | chr21 | 37264400 | 37268999 | high | 11.50 | 0.000277 |
| DSE | 29940 | chr6 | 116350000 | 116353199 | high | 8.00 | 0.000257 |
| DSE | 29940 | chr6 | 116392800 | 116397799 | high | 12.50 | 0.000257 |
| DSE | 29940 | chr6 | 116403400 | 116410399 | high | 17.50 | 0.000257 |
| DSN1 | 79980 | chr20 | 36761400 | 36764199 | low | -12.17 | 0.002176 |
| DZIP3 | 9666 | chr3 | 108588200 | 108590999 | high | 7.00 | 0.000089 |
| E2F1 | 1869 | chr20 | 33676200 | 33679599 | low | -14.78 | 0.000055 |
| E2F7 | 144455 | chr12 | 77061400 | 77066199 | low | -20.52 | 0.001875 |
| E2F7 | 144455 | chr12 | 77051000 | 77054599 | low | -15.65 | 0.001875 |
| EBF1 | 1879 | chr5 | 159060200 | 159063199 | low | -13.04 | 0.000012 |
| EBF1 | 1879 | chr5 | 159069600 | 159070999 | low | -6.08 | 0.000012 |
| EBLN2 | 55096 | chr3 | 73060600 | 73064599 | high | 10.00 | 0.000754 |
| EBLN3P | 100506710 | chr9 | 37079400 | 37080799 | low | -6.08 | 0.000274 |
| EBPL | 84650 | chr13 | 49680200 | 49684399 | high | 10.50 | 0.001704 |
| EBPL | 84650 | chr13 | 49670600 | 49674999 | high | 11.00 | 0.001704 |
| ECD | 11319 | chr10 | 73165600 | 73168999 | low | -14.78 | 0.000155 |
| ECE1 | 1889 | chr1 | 21344200 | 21346199 | high | 5.00 | 0.000089 |
| ECE1 | 1889 | chr1 | 21287400 | 21291399 | high | 10.00 | 0.000089 |
| ECH1 | 1891 | chr19 | 38827200 | 38832799 | high | 14.00 | 0.000049 |
| ECSCR | 641700 | chr5 | 139457400 | 139462599 | high | 13.00 | 0.004601 |
| ECSIT | 51295 | chr19 | 11503200 | 11506799 | high | 9.00 | 0.004459 |
| ECT2 | 1894 | chr3 | 172763000 | 172766599 | high | 9.00 | 0.000011 |
| ECT2 | 1894 | chr3 | 172749400 | 172754199 | high | 12.00 | 0.000011 |
| ECT2 | 1894 | chr3 | 172782400 | 172787799 | high | 13.50 | 0.000011 |
| ECT2L | 345930 | chr6 | 138895600 | 138897999 | low | -10.43 | 0.002491 |
| EDC3 | 80153 | chr15 | 74651400 | 74656599 | high | 13.00 | 0.001074 |
| EDC4 | 23644 | chr16 | 67871400 | 67877399 | high | 15.00 | 0.000348 |
| EDEM2 | 55741 | chr20 | 35146400 | 35147799 | high | 3.50 | 0.001586 |
| EDEM3 | 80267 | chr1 | 184693600 | 184698199 | high | 11.50 | 0.000435 |
| EDEM3 | 80267 | chr1 | 184714000 | 184719999 | high | 15.00 | 0.000435 |
| EDIL3 | 10085 | chr5 | 84220400 | 84224199 | high | 9.50 | 0.000120 |
| EDIL3 | 10085 | chr5 | 84234800 | 84239799 | high | 12.50 | 0.000120 |
| EDIL3 | 10085 | chr5 | 83945200 | 83950399 | high | 13.00 | 0.000120 |
| EDIL3 | 10085 | chr5 | 84056400 | 84061999 | high | 14.00 | 0.000120 |
| EDIL3 | 10085 | chr5 | 84227200 | 84233799 | high | 16.50 | 0.000120 |
| EDNRA | 1909 | chr4 | 147514800 | 147519799 | high | 12.50 | 0.000013 |
| EDNRA | 1909 | chr4 | 147506400 | 147512599 | high | 15.50 | 0.000013 |
| EED | 8726 | chr11 | 86242600 | 86247799 | high | 13.00 | 0.000101 |
| EEF1A1 | 1915 | chr6 | 73518800 | 73522999 | high | 10.50 | 0.000026 |
| EEF1E1 | 9521 | chr6 | 8100400 | 8102799 | low | -10.43 | 0.001175 |
| EEF1G | 1937 | chr11 | 62573000 | 62574199 | low | -5.21 | 0.000031 |
| EEF2K | 29904 | chr16 | 22206000 | 22208999 | high | 7.50 | 0.001347 |
| EEFSEC | 60678 | chr3 | 128277800 | 128281999 | high | 10.50 | 0.000473 |
| EEFSEC | 60678 | chr3 | 128303200 | 128308799 | high | 14.00 | 0.000473 |
| EFHC1 | 114327 | chr6 | 52433200 | 52440199 | high | 17.50 | 0.002180 |
| EFHD2 | 79180 | chr1 | 15409200 | 15411199 | high | 5.00 | 0.005138 |
| EFL1 | 79631 | chr15 | 82150800 | 82154199 | high | 8.50 | 0.000969 |
| EFNA5 | 1946 | chr5 | 107563000 | 107566799 | high | 9.50 | 0.000018 |
| EFNA5 | 1946 | chr5 | 107397800 | 107401999 | high | 10.50 | 0.000018 |

| | | | | | | | |
|---|---|---|---|---|---|---|---|
| EFNA5 | 1946 | chr5 | 107575200 | 107579999 | high | 12.00 | 0.000018 |
| EFNA5 | 1946 | chr5 | 107465800 | 107470599 | high | 12.00 | 0.000018 |
| EFNA5 | 1946 | chr5 | 107458400 | 107463199 | high | 12.00 | 0.000018 |
| EFNA5 | 1946 | chr5 | 107435400 | 107443399 | high | 20.00 | 0.000018 |
| EFNA5 | 1946 | chr5 | 107477200 | 107486799 | high | 24.00 | 0.000018 |
| EGFR | 1956 | chr7 | 55116200 | 55121599 | high | 13.50 | 0.000035 |
| EGFR | 1956 | chr7 | 55102600 | 55108399 | high | 14.50 | 0.000035 |
| EGFR | 1956 | chr7 | 55130000 | 55136799 | high | 17.00 | 0.000035 |
| EGLN1 | 54583 | chr1 | 231417800 | 231422399 | high | 11.50 | 0.000236 |
| EGR2 | 1959 | chr10 | 62816400 | 62818399 | low | -8.69 | 0.000031 |
| EHD1 | 10938 | chr11 | 64876400 | 64879799 | high | 8.50 | 0.000169 |
| EHD2 | 30846 | chr19 | 47713400 | 47714799 | low | -6.08 | 0.000646 |
| EHD4 | 30844 | chr15 | 41971400 | 41972999 | high | 4.00 | 0.000735 |
| EHD4 | 30844 | chr15 | 41922600 | 41925599 | high | 7.50 | 0.000736 |
| EHD4 | 30844 | chr15 | 41918200 | 41921399 | high | 8.00 | 0.000736 |
| EHD4 | 30844 | chr15 | 41964200 | 41967999 | high | 9.50 | 0.000735 |
| EHD4 | 30844 | chr15 | 41936400 | 41940999 | high | 11.50 | 0.000735 |
| EHD4 | 30844 | chr15 | 41942800 | 41947599 | high | 12.00 | 0.000735 |
| EHD4 | 30844 | chr15 | 41929800 | 41935599 | high | 14.50 | 0.000736 |
| EHF | 26298 | chr11 | 34659000 | 34662199 | high | 8.00 | 0.000759 |
| EHF | 26298 | chr11 | 34649800 | 34655399 | high | 14.00 | 0.000759 |
| EHF | 26298 | chr11 | 34643400 | 34648999 | high | 14.00 | 0.000759 |
| EHMT1 | 79813 | chr9 | 137617600 | 137620399 | low | -12.17 | 0.000580 |
| EID1 | 23741 | chr15 | 48880000 | 48881799 | high | 4.50 | 0.000486 |
| EID1 | 23741 | chr15 | 48876800 | 48879199 | high | 6.00 | 0.000486 |
| EIF1AD | 84285 | chr11 | 66000400 | 66002599 | low | -9.56 | 0.001277 |
| EIF1B | 10289 | chr3 | 40309200 | 40311599 | high | 6.00 | 0.000255 |
| EIF2AK2 | 5610 | chr2 | 37156000 | 37157599 | low | -6.95 | 0.000151 |
| EIF2B2 | 8892 | chr14 | 75007200 | 75009199 | low | -8.69 | 0.000119 |
| EIF2B3 | 8891 | chr1 | 44984200 | 44987599 | high | 8.50 | 0.000198 |
| EIF2B3 | 8891 | chr1 | 44936000 | 44939399 | high | 8.50 | 0.000198 |
| EIF2B5 | 8893 | chr3 | 184134800 | 184135799 | low | -4.34 | 0.000048 |
| EIF2D | 1939 | chr1 | 206599600 | 206602399 | low | -12.17 | 0.000009 |
| EIF2S1 | 1965 | chr14 | 67358800 | 67362199 | low | -14.78 | 0.000029 |
| EIF2S2 | 8894 | chr20 | 34106800 | 34109999 | high | 8.00 | 0.000261 |
| EIF2S2 | 8894 | chr20 | 34101400 | 34105199 | high | 9.50 | 0.000261 |
| EIF3A | 8661 | chr10 | 119079000 | 119082399 | low | -14.78 | 0.000073 |
| EIF3E | 3646 | chr8 | 108244600 | 108251399 | high | 17.00 | 0.000034 |
| EIF3H | 8667 | chr8 | 116705400 | 116708999 | high | 9.00 | 0.000074 |
| EIF3H | 8667 | chr8 | 116682400 | 116686199 | high | 9.50 | 0.000074 |
| EIF3H | 8667 | chr8 | 116650800 | 116654599 | high | 9.50 | 0.000074 |
| EIF3I | 8668 | chr1 | 32219600 | 32224199 | high | 11.50 | 0.000269 |
| EIF3L | 51386 | chr22 | 37851200 | 37853399 | high | 5.50 | 0.001358 |
| EIF4A2 | 1974 | chr3 | 186782800 | 186784799 | low | -8.69 | 0.000011 |
| EIF4B | 1975 | chr12 | 53035200 | 53040599 | high | 13.50 | 0.000037 |
| EIF4E | 1977 | chr4 | 98926800 | 98928799 | low | -8.69 | 0.000020 |
| EIF4E2 | 9470 | chr2 | 232563800 | 232567399 | low | -15.65 | 0.000041 |
| EIF4G1 | 1981 | chr3 | 184313400 | 184320399 | high | 17.50 | 0.000011 |
| EIF4G2 | 1982 | chr11 | 10807400 | 10810599 | low | -13.91 | 0.000183 |
| EIF5 | 1983 | chr14 | 103333200 | 103336799 | low | -15.65 | 0.000019 |
| EIF5A2 | 56648 | chr3 | 170906200 | 170908799 | low | -11.30 | 0.000331 |
| EIF5B | 9669 | chr2 | 99387000 | 99391199 | high | 10.50 | 0.000097 |
| EIF6 | 3692 | chr20 | 35281200 | 35285199 | low | -17.39 | 0.000105 |
| ELF5 | 2001 | chr11 | 34484000 | 34495799 | low | -51.30 | 0.000058 |
| ELK4 | 2005 | chr1 | 205629600 | 205633199 | low | -15.65 | 0.000010 |
| ELL | 8178 | chr19 | 18487200 | 18490799 | high | 9.00 | 0.000442 |
| ELL | 8178 | chr19 | 18458200 | 18462399 | high | 10.50 | 0.000443 |
| ELP2 | 55250 | chr18 | 36147200 | 36149999 | high | 7.00 | 0.001528 |
| ELP2 | 55250 | chr18 | 36150800 | 36156399 | high | 14.00 | 0.001528 |
| ELP5 | 23587 | chr17 | 7250000 | 7254199 | high | 10.50 | 0.003253 |
| EMC3 | 55831 | chr3 | 9985000 | 9987999 | high | 7.50 | 0.005591 |
| EML1 | 2009 | chr14 | 99799400 | 99804999 | high | 14.00 | 0.000020 |
| EML1 | 2009 | chr14 | 99792000 | 99798599 | high | 16.50 | 0.000020 |
| EMP3 | 2014 | chr19 | 48325600 | 48331199 | high | 14.00 | 0.000042 |
| ENG | 2022 | chr9 | 127854000 | 127854599 | low | -2.60 | 0.000016 |
| ENKD1 | 84080 | chr16 | 67666000 | 67668399 | high | 6.00 | 0.001243 |
| ENKUR | 219670 | chr10 | 25014800 | 25016599 | high | 4.50 | 0.008782 |
| ENKUR | 219670 | chr10 | 24983400 | 24986799 | high | 8.50 | 0.008793 |
| ENO2 | 2026 | chr12 | 6910200 | 6916399 | high | 15.50 | 0.000293 |
| ENO3 | 2027 | chr17 | 4946400 | 4951399 | high | 12.50 | 0.000410 |
| ENOSF1 | 55556 | chr18 | 709400 | 715799 | high | 16.00 | 0.008702 |
| ENOSF1 | 55556 | chr18 | 677800 | 687399 | high | 24.00 | 0.009107 |
| ENOX1 | 55068 | chr13 | 43311800 | 43314799 | high | 7.50 | 0.001271 |
| ENOX1 | 55068 | chr13 | 43298000 | 43301799 | high | 9.50 | 0.001272 |
| ENOX1 | 55068 | chr13 | 43269200 | 43272999 | high | 9.50 | 0.001273 |
| ENOX1 | 55068 | chr13 | 43293000 | 43296999 | high | 10.00 | 0.001272 |
| ENOX1 | 55068 | chr13 | 43367000 | 43371199 | high | 10.50 | 0.001270 |
| ENOX1 | 55068 | chr13 | 43243400 | 43247599 | high | 10.50 | 0.001273 |

| | | | | | | | |
|---|---|---|---|---|---|---|---|
| ENOX1 | 55068 | chr13 | 43441600 | 43447599 | high | 15.00 | 0.001268 |
| ENOX1 | 55068 | chr13 | 43432000 | 43437999 | high | 15.00 | 0.001268 |
| ENOX1 | 55068 | chr13 | 43279200 | 43285199 | high | 15.00 | 0.001272 |
| ENOX1 | 55068 | chr13 | 43235800 | 43242599 | high | 17.00 | 0.001274 |
| ENPP1 | 5167 | chr6 | 131873200 | 131877199 | low | -17.91 | 0.000039 |
| ENPP3 | 5169 | chr6 | 131729000 | 131731799 | high | 7.00 | 0.000039 |
| ENSA | 2029 | chr1 | 150628600 | 150630399 | low | -7.82 | 0.000013 |
| ENTPD6 | 955 | chr20 | 25220000 | 25226599 | high | 16.50 | 0.000038 |
| ENTPD7 | 57089 | chr10 | 99659000 | 99663199 | high | 10.50 | 0.000573 |
| EP300 | 2033 | chr22 | 41149400 | 41154799 | high | 13.50 | 0.000049 |
| EPAS1 | 2034 | chr2 | 46361800 | 46365199 | high | 8.50 | 0.000044 |
| EPAS1 | 2034 | chr2 | 46317600 | 46321599 | high | 10.00 | 0.000044 |
| EPAS1 | 2034 | chr2 | 46331800 | 46335999 | high | 10.50 | 0.000044 |
| EPAS1 | 2034 | chr2 | 46302200 | 46310199 | high | 20.00 | 0.000044 |
| EPB41 | 2035 | chr1 | 29119400 | 29126199 | high | 17.00 | 0.000070 |
| EPC1 | 80314 | chr10 | 32337200 | 32342799 | high | 14.00 | 0.002484 |
| EPG5 | 57724 | chr18 | 45891400 | 45895399 | high | 10.00 | 0.001258 |
| EPG5 | 57724 | chr18 | 45846000 | 45850599 | high | 11.50 | 0.001259 |
| EPHA2 | 1969 | chr1 | 16140400 | 16146199 | high | 14.50 | 0.000122 |
| EPHA3 | 2042 | chr3 | 89258600 | 89261199 | low | -11.30 | 0.000023 |
| EPHA5 | 2044 | chr4 | 65642000 | 65644399 | high | 6.00 | 0.000031 |
| EPHB2 | 2048 | chr1 | 22749000 | 22755599 | low | -28.63 | 0.000090 |
| EPN2 | 22905 | chr17 | 19268000 | 19272799 | high | 12.00 | 0.001189 |
| EPN2 | 22905 | chr17 | 19293600 | 19298599 | high | 12.50 | 0.001187 |
| EPN2 | 22905 | chr17 | 19310600 | 19316799 | high | 15.50 | 0.001186 |
| EPN2 | 22905 | chr17 | 19280200 | 19287999 | high | 19.50 | 0.001188 |
| EPO | 2056 | chr7 | 100720400 | 100726199 | high | 14.50 | 0.000020 |
| EPOR | 2057 | chr19 | 11380200 | 11381999 | low | -7.82 | 0.000181 |
| EPS15 | 2060 | chr1 | 51515400 | 51520199 | low | -20.34 | 0.000040 |
| EPS15 | 2060 | chr1 | 51498200 | 51501199 | low | -13.04 | 0.000040 |
| EPS15 | 2060 | chr1 | 51459800 | 51462599 | low | -12.17 | 0.000040 |
| EPS8 | 2059 | chr12 | 15656600 | 15658399 | high | 4.50 | 0.000132 |
| EPS8 | 2059 | chr12 | 15678200 | 15680799 | high | 6.50 | 0.000131 |
| EPS8 | 2059 | chr12 | 15748800 | 15751599 | high | 7.00 | 0.000131 |
| EPS8 | 2059 | chr12 | 15640800 | 15644199 | high | 8.50 | 0.000132 |
| EPS8 | 2059 | chr12 | 15687400 | 15691599 | high | 10.50 | 0.000131 |
| EPS8 | 2059 | chr12 | 15663600 | 15668199 | high | 11.50 | 0.000131 |
| EPS8 | 2059 | chr12 | 15645200 | 15650199 | high | 12.50 | 0.000132 |
| EPS8 | 2059 | chr12 | 15700400 | 15708799 | high | 21.00 | 0.000131 |
| EPT1 | 85465 | chr2 | 26345000 | 26348799 | low | -16.18 | 0.003244 |
| EPYC | 1833 | chr12 | 90998600 | 90999599 | low | -4.34 | 0.000020 |
| ERAL1 | 26284 | chr17 | 28854200 | 28855999 | low | -7.82 | 0.000911 |
| ERAP1 | 51752 | chr5 | 96770000 | 96771999 | high | 5.00 | 0.000535 |
| ERAP1 | 51752 | chr5 | 96795800 | 96798999 | high | 8.00 | 0.000535 |
| ERAP1 | 51752 | chr5 | 96807200 | 96811199 | high | 10.00 | 0.000535 |
| ERAP1 | 51752 | chr5 | 96773600 | 96778599 | high | 12.50 | 0.000535 |
| ERBB4 | 2066 | chr2 | 211835400 | 211836799 | high | 3.50 | 0.000010 |
| ERC1 | 23085 | chr12 | 1441000 | 1444199 | high | 8.00 | 0.016020 |
| ERC1 | 23085 | chr12 | 1431200 | 1436599 | high | 13.50 | 0.016130 |
| ERC1 | 23085 | chr12 | 1449200 | 1457599 | high | 21.00 | 0.015929 |
| ERC1 | 23085 | chr12 | 1470600 | 1479599 | high | 22.50 | 0.015698 |
| ERCC1 | 2067 | chr19 | 45405000 | 45408399 | high | 8.50 | 0.000046 |
| ERCC2 | 2068 | chr19 | 45369600 | 45370999 | high | 3.50 | 0.000046 |
| ERCC4 | 2072 | chr16 | 13932200 | 13934599 | high | 6.00 | 0.000149 |
| ERCC5 | 2073 | chr13 | 102846000 | 102847599 | low | -6.95 | 0.000020 |
| ERCC6 | 2074 | chr10 | 49538200 | 49540199 | low | -8.69 | 0.000042 |
| ERFE | 151176 | chr2 | 238168600 | 238173599 | high | 12.50 | 0.000635 |
| ERGIC1 | 57222 | chr5 | 172881200 | 172885799 | high | 11.50 | 0.000331 |
| ERGIC1 | 57222 | chr5 | 172833600 | 172840599 | high | 17.50 | 0.000331 |
| ERI2 | 112479 | chr16 | 20806000 | 20807999 | low | -8.69 | 0.005406 |
| ERI3 | 79033 | chr1 | 44327400 | 44331599 | low | -18.91 | 0.001783 |
| ERN1 | 2081 | chr17 | 64112600 | 64116799 | low | -18.30 | 0.000032 |
| ERN1 | 2081 | chr17 | 64128400 | 64130599 | low | -9.56 | 0.000032 |
| ERO1A | 30001 | chr14 | 52693800 | 52696999 | high | 8.00 | 0.000569 |
| ERP29 | 10961 | chr12 | 112007600 | 112014799 | low | -31.30 | 0.000098 |
| ERP44 | 23071 | chr9 | 100097200 | 100100399 | high | 8.00 | 0.000230 |
| ERP44 | 23071 | chr9 | 100055800 | 100059799 | high | 10.00 | 0.000231 |
| ESD | 2098 | chr13 | 46795000 | 46797199 | low | -9.56 | 0.000045 |
| ESR1 | 2099 | chr6 | 151935400 | 151938999 | high | 9.00 | 0.000014 |
| ESR1 | 2099 | chr6 | 151908000 | 151914399 | high | 16.00 | 0.000014 |
| ESR2 | 2100 | chr14 | 64231600 | 64235599 | high | 10.00 | 0.000033 |
| ESR2 | 2100 | chr14 | 64334400 | 64339999 | high | 14.00 | 0.000033 |
| ESR2 | 2100 | chr14 | 64322600 | 64328799 | high | 15.50 | 0.000033 |
| ESRRA | 2101 | chr11 | 64313200 | 64315799 | high | 6.50 | 0.000033 |
| ESRRA | 2101 | chr11 | 64316600 | 64320799 | high | 10.50 | 0.000033 |
| ESRRA | 2101 | chr11 | 64304000 | 64308599 | high | 11.50 | 0.000033 |
| ESYT2 | 57488 | chr7 | 158828200 | 158830599 | low | -10.43 | 0.000362 |
| ETF1 | 2107 | chr5 | 138520200 | 138525199 | high | 12.50 | 0.000015 |

| | | | | | | | |
|---|---|---|---|---|---|---|---|
| ETNK1 | 55500 | chr12 | 22625200 | 22627599 | high | 6.00 | 0.002453 |
| ETS2 | 2114 | chr21 | 38806200 | 38808999 | low | -12.17 | 0.000054 |
| ETV1 | 2115 | chr7 | 13903800 | 13906599 | high | 7.00 | 0.000152 |
| ETV1 | 2115 | chr7 | 13951400 | 13954999 | high | 9.00 | 0.000152 |
| ETV1 | 2115 | chr7 | 13988000 | 13992199 | high | 10.50 | 0.000151 |
| ETV1 | 2115 | chr7 | 13943400 | 13948999 | high | 14.00 | 0.000152 |
| ETV1 | 2115 | chr7 | 13987200 | 13993599 | high | 16.00 | 0.000151 |
| ETV1 | 2115 | chr7 | 13894000 | 13900399 | high | 16.00 | 0.000152 |
| ETV2 | 2116 | chr19 | 35642000 | 35644999 | high | 7.50 | 0.000059 |
| ETV5 | 2119 | chr3 | 186106800 | 186109799 | high | 7.50 | 0.000011 |
| ETV7 | 51513 | chr6 | 36368800 | 36370999 | low | -9.56 | 0.001416 |
| EVA1A | 84141 | chr2 | 75525800 | 75529199 | high | 8.50 | 0.001114 |
| EVA1A | 84141 | chr2 | 75553200 | 75563599 | high | 26.00 | 0.001114 |
| EVA1C | 59271 | chr21 | 32410600 | 32415199 | high | 11.50 | 0.001829 |
| EVA1C | 59271 | chr21 | 32457200 | 32465799 | high | 21.50 | 0.001826 |
| EVC | 2121 | chr4 | 5711200 | 5713399 | high | 5.50 | 0.000371 |
| EVC | 2121 | chr4 | 5811000 | 5815199 | high | 10.50 | 0.000365 |
| EVC | 2121 | chr4 | 5749200 | 5755799 | high | 16.50 | 0.000369 |
| EVC2 | 132884 | chr4 | 5707000 | 5709799 | low | -12.17 | 0.002587 |
| EVI2A | 2123 | chr17 | 31313800 | 31316599 | high | 7.00 | 0.000068 |
| EVI2B | 2124 | chr17 | 31313800 | 31316599 | high | 7.00 | 0.000068 |
| EVI2B | 2124 | chr17 | 31309200 | 31312999 | high | 9.50 | 0.000068 |
| EVL | 51466 | chr14 | 100069400 | 100073399 | high | 10.00 | 0.000514 |
| EXD2 | 55218 | chr14 | 69191000 | 69192999 | low | -8.69 | 0.000798 |
| EXOC1 | 55763 | chr4 | 55893200 | 55895799 | high | 6.50 | 0.000998 |
| EXOC2 | 55770 | chr6 | 577200 | 580399 | high | 8.00 | 0.010736 |
| EXOC3 | 11336 | chr5 | 442200 | 444799 | low | -11.30 | 0.002848 |
| EXOC6 | 54536 | chr10 | 93052800 | 93056199 | high | 8.50 | 0.000586 |
| EXOC7 | 23265 | chr17 | 76085600 | 76089199 | high | 9.00 | 0.000306 |
| EXOC8 | 149371 | chr1 | 231337800 | 231339599 | low | -7.82 | 0.000646 |
| EXOSC10 | 5394 | chr1 | 11097200 | 11100799 | high | 9.00 | 0.000486 |
| EXOSC2 | 23404 | chr9 | 130693000 | 130694599 | high | 4.00 | 0.000179 |
| EXOSC3 | 51010 | chr9 | 37782200 | 37786799 | high | 11.50 | 0.001350 |
| EXOSC4 | 54512 | chr8 | 144077600 | 144080399 | high | 7.00 | 0.000378 |
| EXOSC6 | 118460 | chr16 | 70250000 | 70255199 | high | 13.00 | 0.001686 |
| EXOSC7 | 23016 | chr3 | 44981200 | 44985199 | high | 10.00 | 0.000512 |
| EXOSC8 | 11340 | chr13 | 36999800 | 37002799 | low | -13.04 | 0.000306 |
| EXOSC8 | 11340 | chr13 | 37006400 | 37008199 | low | -7.82 | 0.000306 |
| EXT1 | 2131 | chr8 | 117978000 | 117982599 | high | 11.50 | 0.000018 |
| EXT1 | 2131 | chr8 | 117996000 | 118000999 | high | 12.50 | 0.000018 |
| EXT1 | 2131 | chr8 | 118049000 | 118054599 | high | 14.00 | 0.000018 |
| EXT1 | 2131 | chr8 | 118016600 | 118023999 | high | 18.50 | 0.000018 |
| EXTL1 | 2134 | chr1 | 26032400 | 26038799 | high | 16.00 | 0.000082 |
| EXTL3 | 2137 | chr8 | 28701800 | 28703599 | low | -7.82 | 0.000074 |
| EYA3 | 2140 | chr1 | 28014200 | 28017199 | low | -13.04 | 0.000076 |
| EYA3 | 2140 | chr1 | 28007400 | 28010399 | low | -13.04 | 0.000076 |
| EYA3 | 2140 | chr1 | 28033000 | 28035399 | low | -10.43 | 0.000076 |
| EYA3 | 2140 | chr1 | 28049600 | 28051799 | low | -9.56 | 0.000076 |
| EYA3 | 2140 | chr1 | 28042400 | 28044599 | low | -9.56 | 0.000076 |
| EYA4 | 2070 | chr6 | 133394000 | 133396199 | low | -9.56 | 0.000016 |
| EYA4 | 2070 | chr6 | 133311400 | 133313599 | low | -9.56 | 0.000016 |
| EZH1 | 2145 | chr17 | 42723600 | 42726199 | low | -11.30 | 0.000050 |
| EZH2 | 2146 | chr7 | 148879200 | 148885799 | low | -16.50 | 0.000014 |
| F12 | 2161 | chr5 | 177402200 | 177405599 | high | 8.50 | 0.000012 |
| F2R | 2149 | chr5 | 76714400 | 76719199 | high | 12.00 | 0.000028 |
| F2RL1 | 2150 | chr5 | 76818200 | 76822199 | high | 10.00 | 0.000028 |
| F3 | 2152 | chr1 | 94538200 | 94542199 | high | 10.00 | 0.000023 |
| F5 | 2153 | chr1 | 169518200 | 169523999 | high | 14.50 | 0.000013 |
| FADS3 | 3995 | chr11 | 61890000 | 61892199 | high | 5.50 | 0.000065 |
| FADS3 | 3995 | chr11 | 61878600 | 61889199 | high | 26.50 | 0.000065 |
| FAF1 | 11124 | chr1 | 50929400 | 50935999 | high | 16.50 | 0.000218 |
| FAF2 | 23197 | chr5 | 176482400 | 176486799 | high | 11.00 | 0.000131 |
| FAF2 | 23197 | chr5 | 176445600 | 176451799 | high | 15.50 | 0.000131 |
| FAH | 2184 | chr15 | 80159200 | 80163399 | high | 10.50 | 0.000027 |
| FAH | 2184 | chr15 | 80150800 | 80158399 | high | 19.00 | 0.000027 |
| FAHD1 | 81889 | chr16 | 1823400 | 1830199 | high | 17.00 | 0.004990 |
| FALEC | 100874054 | chr1 | 150515000 | 150517199 | high | 5.50 | 0.000068 |
| FAS | 355 | chr10 | 88998600 | 89002799 | high | 10.50 | 0.000004 |
| FASLG | 356 | chr1 | 172658600 | 172663799 | high | 13.00 | 0.000002 |
| FASN | 2194 | chr17 | 82094400 | 82101599 | high | 18.00 | 0.000027 |
| FASTK | 10922 | chr7 | 151075200 | 151080799 | high | 14.00 | 0.000072 |
| FAT4 | 79633 | chr4 | 125359000 | 125362199 | high | 8.00 | 0.000635 |
| FAT4 | 79633 | chr4 | 125343800 | 125347199 | high | 8.50 | 0.000635 |
| FAT4 | 79633 | chr4 | 125348000 | 125352199 | high | 10.50 | 0.000635 |
| FAU | 2197 | chr11 | 65120400 | 65122799 | low | -10.43 | 0.000034 |
| FBL | 2091 | chr19 | 39843600 | 39847599 | high | 10.00 | 0.000052 |
| FBLN1 | 2192 | chr22 | 45540800 | 45543199 | high | 6.00 | 0.000048 |
| FBLN1 | 2192 | chr22 | 45502000 | 45505399 | high | 8.50 | 0.000048 |

| | | | | | | | |
|---|---|---|---|---|---|---|---|
| FBLN1 | 2192 | chr22 | 45552400 | 45555999 | high | 9.00 | 0.000048 |
| FBLN1 | 2192 | chr22 | 45547800 | 45551599 | high | 9.50 | 0.000048 |
| FBLN1 | 2192 | chr22 | 45520600 | 45524599 | high | 10.00 | 0.000048 |
| FBLN5 | 10516 | chr14 | 91917600 | 91920799 | high | 8.00 | 0.000114 |
| FBN1 | 2200 | chr15 | 48607600 | 48610599 | high | 7.50 | 0.000045 |
| FBN1 | 2200 | chr15 | 48601600 | 48605399 | high | 9.50 | 0.000045 |
| FBN1 | 2200 | chr15 | 48633200 | 48637599 | high | 11.00 | 0.000045 |
| FBN1 | 2200 | chr15 | 48585600 | 48590399 | high | 12.00 | 0.000045 |
| FBN1 | 2200 | chr15 | 48506400 | 48511599 | high | 13.00 | 0.000045 |
| FBN1 | 2200 | chr15 | 48546800 | 48555599 | high | 22.00 | 0.000045 |
| FBN1 | 2200 | chr15 | 48570800 | 48580999 | high | 25.50 | 0.000045 |
| FBN1 | 2200 | chr15 | 48516200 | 48527399 | high | 28.00 | 0.000045 |
| FBN2 | 2201 | chr5 | 128408800 | 128413199 | high | 11.00 | 0.000017 |
| FBN2 | 2201 | chr5 | 128360000 | 128364799 | high | 12.00 | 0.000017 |
| FBN2 | 2201 | chr5 | 128395000 | 128399999 | high | 12.50 | 0.000017 |
| FBN2 | 2201 | chr5 | 128415600 | 128422199 | high | 16.50 | 0.000017 |
| FBN2 | 2201 | chr5 | 128532400 | 128540199 | high | 19.50 | 0.000017 |
| FBXL12 | 54850 | chr19 | 9818400 | 9818999 | low | -2.60 | 0.005586 |
| FBXL14 | 144699 | chr12 | 1590400 | 1596599 | high | 15.50 | 0.010109 |
| FBXL15 | 79176 | chr10 | 102420800 | 102423199 | high | 6.00 | 0.000773 |
| FBXL17 | 64839 | chr5 | 108076800 | 108079999 | high | 8.00 | 0.000600 |
| FBXL17 | 64839 | chr5 | 107958600 | 107961999 | high | 8.50 | 0.000601 |
| FBXL17 | 64839 | chr5 | 108070800 | 108074799 | high | 10.00 | 0.000600 |
| FBXL17 | 64839 | chr5 | 107874000 | 107877999 | high | 10.00 | 0.000601 |
| FBXL18 | 80028 | chr7 | 5486400 | 5489199 | high | 7.00 | 0.014587 |
| FBXL22 | 283807 | chr15 | 63596200 | 63601399 | high | 13.00 | 0.004463 |
| FBXL3 | 26224 | chr13 | 77025200 | 77027599 | high | 6.00 | 0.000340 |
| FBXL4 | 26235 | chr6 | 98944200 | 98947399 | low | -13.91 | 0.000265 |
| FBXL7 | 23194 | chr5 | 15500000 | 15503399 | low | -14.78 | 0.001496 |
| FBXL8 | 55336 | chr16 | 67159600 | 67167399 | high | 19.50 | 0.000824 |
| FGF1 | 2246 | chr5 | 142661200 | 142666799 | high | 14.00 | 0.000016 |
| FGF1 | 2246 | chr5 | 142594400 | 142601999 | high | 19.00 | 0.000016 |
| FGF5 | 2250 | chr4 | 80263600 | 80270199 | high | 16.50 | 0.000028 |
| FGFBP3 | 143282 | chr10 | 91906200 | 91908799 | high | 6.50 | 0.001559 |
| FGFR2 | 2263 | chr10 | 121595600 | 121598999 | low | -4.22 | 0.000325 |
| FGGY | 55277 | chr1 | 59431000 | 59433999 | high | 7.50 | 0.000930 |
| FGGY | 55277 | chr1 | 59632600 | 59636199 | high | 9.00 | 0.000927 |
| FGGY | 55277 | chr1 | 59540200 | 59544599 | high | 11.00 | 0.000928 |
| FGGY | 55277 | chr1 | 59574000 | 59578599 | high | 11.50 | 0.000928 |
| FGGY | 55277 | chr1 | 59400000 | 59404599 | high | 11.50 | 0.000931 |
| FH | 2271 | chr1 | 241517600 | 241519999 | low | -10.43 | 0.000009 |
| FHL3 | 2275 | chr1 | 38003600 | 38004999 | low | -6.08 | 0.000060 |
| FHOD1 | 29109 | chr16 | 67233600 | 67236999 | high | 8.50 | 0.000433 |
| FHOD1 | 29109 | chr16 | 67241200 | 67244999 | high | 9.50 | 0.000433 |
| FHOD3 | 80206 | chr18 | 36330000 | 36333799 | high | 9.50 | 0.002208 |
| FHOD3 | 80206 | chr18 | 36322600 | 36326399 | high | 9.50 | 0.002208 |
| FHOD3 | 80206 | chr18 | 36346800 | 36351799 | high | 12.50 | 0.002207 |
| FHOD3 | 80206 | chr18 | 36363200 | 36368399 | high | 13.00 | 0.002206 |
| FICD | 11153 | chr12 | 108514600 | 108519199 | low | -19.39 | 0.000103 |
| FILIP1 | 27145 | chr6 | 75437000 | 75439199 | high | 5.50 | 0.000360 |
| FILIP1 | 27145 | chr6 | 75324000 | 75327199 | high | 8.00 | 0.000360 |
| FILIP1 | 27145 | chr6 | 75299000 | 75302999 | high | 10.00 | 0.000360 |
| FILIP1 | 27145 | chr6 | 75480600 | 75484999 | high | 11.00 | 0.000360 |
| FILIP1 | 27145 | chr6 | 75303800 | 75308399 | high | 11.50 | 0.000360 |
| FILIP1 | 27145 | chr6 | 75449000 | 75454199 | high | 13.00 | 0.000360 |
| FJX1 | 24147 | chr11 | 35617400 | 35622599 | low | -22.26 | 0.000678 |
| FKBP10 | 60681 | chr17 | 41811200 | 41814399 | low | -13.91 | 0.001451 |
| FN1 | 2335 | chr2 | 215429600 | 215436599 | low | -30.43 | 0.000011 |
| FN1 | 2335 | chr2 | 215381600 | 215384399 | low | -12.17 | 0.000011 |
| FNBP1 | 23048 | chr9 | 129981400 | 129986599 | low | -22.04 | 0.000177 |
| FNBP1 | 23048 | chr9 | 129997200 | 130000999 | low | -16.00 | 0.000177 |
| FNBP1L | 54874 | chr1 | 93526800 | 93529399 | low | -11.30 | 0.000587 |
| FNBP4 | 23360 | chr11 | 47762400 | 47768399 | low | -25.56 | 0.000489 |
| FNDC7 | 163479 | chr1 | 108720600 | 108724799 | high | 10.50 | 0.001504 |
| FNIP1 | 96459 | chr5 | 131645000 | 131648599 | high | 9.00 | 0.000733 |
| FNIP1 | 96459 | chr5 | 131668000 | 131673199 | high | 13.00 | 0.000733 |
| FNIP1 | 96459 | chr5 | 131707400 | 131712799 | high | 13.50 | 0.000732 |
| FNIP2 | 57600 | chr4 | 158767800 | 158773799 | high | 15.00 | 0.000363 |
| FNTA | 2339 | chr8 | 43055600 | 43057799 | low | -9.56 | 0.000054 |
| FNTB | 2342 | chr14 | 64989000 | 64991399 | low | -10.43 | 0.000036 |
| FOCAD | 54914 | chr9 | 20907800 | 20909799 | high | 5.00 | 0.002626 |
| FOCAD | 54914 | chr9 | 20741800 | 20744399 | high | 6.50 | 0.002648 |
| FOCAD | 54914 | chr9 | 20724400 | 20726999 | high | 6.50 | 0.002650 |
| FOCAD | 54914 | chr9 | 20754200 | 20757199 | high | 7.50 | 0.002646 |
| FOCAD | 54914 | chr9 | 20699800 | 20703199 | high | 8.50 | 0.002653 |
| FOCAD | 54914 | chr9 | 20746800 | 20750799 | high | 10.00 | 0.002647 |
| FOCAD | 54914 | chr9 | 20980800 | 20985999 | high | 13.00 | 0.002617 |
| FOCAD | 54914 | chr9 | 20687200 | 20693399 | high | 15.50 | 0.002654 |

| | | | | | | | |
|---|---|---|---|---|---|---|---|
| FOLR2 | 2350 | chr11 | 72221400 | 72228599 | high | 18.00 | 0.000033 |
| FOSB | 2354 | chr19 | 45471600 | 45475199 | high | 9.00 | 0.000052 |
| FOSL1 | 8061 | chr11 | 65899000 | 65901599 | high | 6.50 | 0.000122 |
| FOXA3 | 3171 | chr19 | 45865200 | 45867799 | high | 6.50 | 0.000069 |
| FOXD1 | 2297 | chr5 | 73444800 | 73448999 | low | -18.24 | 0.000031 |
| FOXJ1 | 2302 | chr17 | 76138800 | 76142999 | high | 10.50 | 0.000030 |
| FOXJ3 | 22887 | chr1 | 42190000 | 42191999 | high | 5.00 | 0.000542 |
| FOXK2 | 3607 | chr17 | 82586200 | 82589599 | high | 8.50 | 0.000044 |
| FOXK2 | 3607 | chr17 | 82534600 | 82538799 | high | 10.50 | 0.000044 |
| FOXK2 | 3607 | chr17 | 82518000 | 82522399 | high | 11.00 | 0.000044 |
| FOXL2 | 668 | chr3 | 138943600 | 138947599 | high | 10.00 | 0.000005 |
| FOXM1 | 2305 | chr12 | 2863400 | 2868799 | high | 13.50 | 0.000805 |
| FOXN2 | 3344 | chr2 | 48360000 | 48363599 | high | 9.00 | 0.000069 |
| FOXN3 | 1112 | chr14 | 89415800 | 89420199 | high | 11.00 | 0.000012 |
| FOXO3 | 2309 | chr6 | 108573800 | 108576799 | high | 7.50 | 0.000021 |
| FOXO3 | 2309 | chr6 | 108628200 | 108631599 | high | 8.50 | 0.000021 |
| FOXO3 | 2309 | chr6 | 108595200 | 108598799 | high | 9.00 | 0.000021 |
| FOXP1 | 27086 | chr3 | 71527000 | 71531199 | high | 10.50 | 0.000379 |
| FOXP1 | 27086 | chr3 | 71153200 | 71157599 | high | 11.00 | 0.000381 |
| FOXP1 | 27086 | chr3 | 71083800 | 71088199 | high | 11.00 | 0.000381 |
| FOXP1 | 27086 | chr3 | 70968000 | 70973599 | high | 14.00 | 0.000382 |
| FPGS | 2356 | chr9 | 127803000 | 127806199 | low | -13.91 | 0.000018 |
| FRMD5 | 84978 | chr15 | 43921000 | 43924799 | high | 9.50 | 0.001935 |
| FRMD5 | 84978 | chr15 | 44027000 | 44032999 | high | 15.00 | 0.001930 |
| FRMD5 | 84978 | chr15 | 43931400 | 43937799 | high | 16.00 | 0.001934 |
| FRMD6 | 122786 | chr14 | 51488400 | 51492399 | high | 10.00 | 0.002385 |
| FRMD6 | 122786 | chr14 | 51604600 | 51608799 | high | 10.50 | 0.002379 |
| FRMD6 | 122786 | chr14 | 51575800 | 51580199 | high | 11.00 | 0.002381 |
| FRMD6 | 122786 | chr14 | 51590800 | 51595399 | high | 11.50 | 0.002380 |
| FRMD6 | 122786 | chr14 | 51581000 | 51585599 | high | 11.50 | 0.002380 |
| FRMD6 | 122786 | chr14 | 51535200 | 51540799 | high | 14.00 | 0.002383 |
| FRMD6 | 122786 | chr14 | 51528600 | 51534199 | high | 14.00 | 0.002383 |
| FRMD6 | 122786 | chr14 | 51597600 | 51603799 | high | 15.50 | 0.002380 |
| FRMD6 | 122786 | chr14 | 51542000 | 51550799 | high | 22.00 | 0.002382 |
| FRS2 | 10818 | chr12 | 69469600 | 69472399 | high | 7.00 | 0.000156 |
| FRS2 | 10818 | chr12 | 69524800 | 69532999 | high | 20.50 | 0.000156 |
| FRS3 | 10817 | chr6 | 41778000 | 41780199 | high | 5.50 | 0.000259 |
| FRYL | 285527 | chr4 | 48725200 | 48729599 | high | 11.00 | 0.005860 |
| FRYL | 285527 | chr4 | 48542600 | 48547799 | high | 13.00 | 0.005882 |
| FSBP | 100861412 | chr8 | 94430400 | 94434999 | high | 11.50 | 0.009558 |
| FSTL1 | 11167 | chr3 | 120393600 | 120395999 | high | 6.00 | 0.000093 |
| FSTL3 | 10272 | chr19 | 679000 | 680999 | high | 5.00 | 0.015128 |
| FTH1 | 2495 | chr11 | 61965200 | 61967199 | low | -8.69 | 0.000040 |
| FTO | 79068 | chr16 | 53870800 | 53872999 | high | 5.50 | 0.001468 |
| FTO | 79068 | chr16 | 53703800 | 53706599 | high | 7.00 | 0.001472 |
| FTX | 100302692 | chrX | 74290000 | 74293399 | low | -14.78 | 0.000136 |
| FUK | 197258 | chr16 | 70453000 | 70458999 | high | 15.00 | 0.002800 |
| FURIN | 5045 | chr15 | 90869600 | 90874199 | high | 11.50 | 0.000056 |
| FUT6 | 2528 | chr19 | 5824400 | 5831199 | high | 17.00 | 0.000434 |
| FUT8 | 2530 | chr14 | 65511000 | 65513199 | high | 5.50 | 0.000039 |
| FUT8 | 2530 | chr14 | 65412000 | 65415399 | high | 8.50 | 0.000039 |
| FUT8 | 2530 | chr14 | 65429800 | 65434199 | high | 11.00 | 0.000039 |
| FUZ | 80199 | chr19 | 49802200 | 49806999 | high | 12.00 | 0.001610 |
| FXR1 | 8087 | chr3 | 180981400 | 180983599 | low | -9.56 | 0.000045 |
| FYB | 2533 | chr5 | 39147600 | 39150799 | high | 8.00 | 0.000065 |
| FYB | 2533 | chr5 | 39245800 | 39249599 | high | 9.50 | 0.000065 |
| FYB | 2533 | chr5 | 39182400 | 39187399 | high | 12.50 | 0.000065 |
| FYB | 2533 | chr5 | 39173800 | 39179199 | high | 13.50 | 0.000065 |
| FYB | 2533 | chr5 | 39139600 | 39145199 | high | 14.00 | 0.000065 |
| FYB | 2533 | chr5 | 39152400 | 39158199 | high | 14.50 | 0.000065 |
| FYB | 2533 | chr5 | 39159400 | 39165799 | high | 16.00 | 0.000065 |
| FYB | 2533 | chr5 | 39250400 | 39259199 | high | 22.00 | 0.000065 |
| FYCO1 | 79443 | chr3 | 45954200 | 45956799 | high | 6.50 | 0.001729 |
| FYCO1 | 79443 | chr3 | 45969200 | 45973999 | high | 12.00 | 0.001728 |
| FYN | 2534 | chr6 | 111662000 | 111664199 | high | 5.50 | 0.000023 |
| FYN | 2534 | chr6 | 111665400 | 111668799 | high | 8.50 | 0.000023 |
| FYN | 2534 | chr6 | 111670200 | 111674799 | high | 11.50 | 0.000023 |
| FYN | 2534 | chr6 | 111736200 | 111741999 | high | 14.50 | 0.000023 |
| FYTTD1 | 84248 | chr3 | 197753000 | 197757599 | high | 11.50 | 0.000426 |
| FZD2 | 2535 | chr17 | 44556000 | 44560399 | high | 11.00 | 0.000057 |
| FZD4 | 8322 | chr11 | 86946400 | 86953399 | high | 17.50 | 0.000096 |
| FZD6 | 8323 | chr8 | 103298000 | 103302199 | high | 10.50 | 0.000081 |
| FZD6 | 8323 | chr8 | 103303800 | 103309399 | high | 14.00 | 0.000081 |
| FZD7 | 8324 | chr2 | 202033200 | 202039799 | high | 16.50 | 0.000041 |
| G2E3 | 55632 | chr14 | 30608800 | 30611999 | high | 8.00 | 0.001818 |
| G2E3 | 55632 | chr14 | 30596000 | 30600999 | high | 12.50 | 0.001818 |
| G2E3 | 55632 | chr14 | 30556600 | 30561999 | high | 13.50 | 0.001821 |
| G2E3 | 55632 | chr14 | 30618000 | 30626599 | high | 21.50 | 0.001817 |

| | | | | | | | |
|---|---|---|---|---|---|---|---|
| G3BP2 | 9908 | chr4 | 75650200 | 75654399 | high | 10.50 | 0.000131 |
| G3BP2 | 9908 | chr4 | 75643000 | 75648799 | high | 14.50 | 0.000131 |
| G6PC | 2538 | chr17 | 42899200 | 42903799 | high | 11.50 | 0.000059 |
| G6PD | 2539 | chrX | 154546600 | 154550599 | high | 10.00 | 0.000016 |
| GAA | 2548 | chr17 | 80108400 | 80112399 | high | 10.00 | 0.000032 |
| GAA | 2548 | chr17 | 80099600 | 80103999 | high | 11.00 | 0.000032 |
| GAB2 | 9846 | chr11 | 78414800 | 78419399 | high | 11.50 | 0.000126 |
| GAB3 | 139716 | chrX | 154749600 | 154752799 | low | -13.91 | 0.000903 |
| GALK2 | 2585 | chr15 | 49223400 | 49228399 | high | 12.50 | 0.000053 |
| GALK2 | 2585 | chr15 | 49159800 | 49165599 | high | 14.50 | 0.000053 |
| GALK2 | 2585 | chr15 | 49329200 | 49335999 | high | 17.00 | 0.000052 |
| GALM | 130589 | chr2 | 38700600 | 38705999 | high | 13.50 | 0.003374 |
| GALNT10 | 55568 | chr5 | 154370400 | 154373799 | high | 8.50 | 0.000360 |
| GALNT10 | 55568 | chr5 | 154362600 | 154367199 | high | 11.50 | 0.000360 |
| GALNT15 | 117248 | chr3 | 16186600 | 16189799 | high | 8.00 | 0.007244 |
| GALNT15 | 117248 | chr3 | 16191000 | 16195999 | high | 12.50 | 0.007242 |
| GALNT15 | 117248 | chr3 | 16173600 | 16180199 | high | 16.50 | 0.007249 |
| GALNT15 | 117248 | chr3 | 16201600 | 16209599 | high | 20.00 | 0.007237 |
| GALNT16 | 57452 | chr14 | 69277800 | 69282599 | high | 12.00 | 0.000829 |
| GALNT2 | 2590 | chr1 | 230066400 | 230068799 | high | 6.00 | 0.000011 |
| GALNT4 | 8693 | chr12 | 89520800 | 89527399 | low | -28.67 | 0.000097 |
| GALNT4 | 8693 | chr12 | 89524600 | 89526999 | low | -10.43 | 0.000097 |
| GALNT6 | 11226 | chr12 | 51354600 | 51359199 | high | 11.50 | 0.000219 |
| GAPVD1 | 26130 | chr9 | 125260400 | 125263199 | high | 7.00 | 0.000209 |
| GAPVD1 | 26130 | chr9 | 125287800 | 125291799 | high | 10.00 | 0.000209 |
| GAREM1 | 64762 | chr18 | 32380200 | 32384199 | high | 10.00 | 0.002000 |
| GAREM1 | 64762 | chr18 | 32353400 | 32357999 | high | 11.50 | 0.002002 |
| GARNL3 | 84253 | chr9 | 127388800 | 127391199 | high | 6.00 | 0.000661 |
| GART | 2618 | chr21 | 33514600 | 33517799 | high | 8.00 | 0.000078 |
| GAS1 | 2619 | chr9 | 86944800 | 86949199 | low | -19.52 | 0.000030 |
| GAS1RR | 100506834 | chr9 | 86944800 | 86949199 | low | -19.17 | 0.000117 |
| GAS2L3 | 283431 | chr12 | 100573400 | 100574399 | low | -4.34 | 0.002818 |
| GAS5 | 60674 | chr1 | 173865800 | 173871799 | low | -24.95 | 0.000349 |
| GAS5 | 60674 | chr1 | 173864600 | 173869199 | low | -19.13 | 0.000349 |
| GAS6 | 2621 | chr13 | 113837200 | 113839999 | high | 7.00 | 0.000023 |
| GATC | 283459 | chr12 | 120444200 | 120447999 | high | 9.50 | 0.002353 |
| GATC | 283459 | chr12 | 120452200 | 120465399 | high | 33.00 | 0.002353 |
| GBAT2 | 101927886 | chr1 | 151345600 | 151347399 | high | 4.50 | 0.000068 |
| GBE1 | 2632 | chr3 | 81727600 | 81729999 | high | 6.00 | 0.000032 |
| GBE1 | 2632 | chr3 | 81759600 | 81763199 | high | 9.00 | 0.000032 |
| GBE1 | 2632 | chr3 | 81738600 | 81742799 | high | 10.50 | 0.000032 |
| GBF1 | 8729 | chr10 | 102324200 | 102326799 | high | 6.50 | 0.000085 |
| GBF1 | 8729 | chr10 | 102347000 | 102350599 | high | 9.00 | 0.000085 |
| GBF1 | 8729 | chr10 | 102331200 | 102334799 | high | 9.00 | 0.000085 |
| GBF1 | 8729 | chr10 | 102309200 | 102313999 | high | 12.00 | 0.000085 |
| GBF1 | 8729 | chr10 | 102341000 | 102345999 | high | 12.50 | 0.000085 |
| GBP3 | 2635 | chr1 | 89020200 | 89022399 | low | -9.56 | 0.000030 |
| GBP3 | 2635 | chr1 | 89007000 | 89008399 | low | -6.08 | 0.000030 |
| GCFC2 | 6936 | chr2 | 75694600 | 75696999 | low | -10.43 | 0.000092 |
| GCH1 | 2643 | chr14 | 54900200 | 54902599 | high | 6.00 | 0.000048 |
| GCK | 2645 | chr7 | 44165200 | 44168999 | high | 9.50 | 0.000060 |
| GCKR | 2646 | chr2 | 27492400 | 27496999 | high | 11.50 | 0.000096 |
| GCLM | 2730 | chr1 | 93908000 | 93909399 | high | 3.50 | 0.000029 |
| GCLM | 2730 | chr1 | 93906200 | 93909799 | high | 9.00 | 0.000029 |
| GCOM1 | 145781 | chr15 | 57706000 | 57707599 | low | -6.95 | 0.002526 |
| GDE1 | 51573 | chr16 | 19521200 | 19522199 | low | -4.34 | 0.002642 |
| GDF5 | 8200 | chr20 | 35443000 | 35446599 | high | 9.00 | 0.000231 |
| GDF5 | 8200 | chr20 | 35451400 | 35455799 | high | 11.00 | 0.000231 |
| GDF5 | 8200 | chr20 | 35433600 | 35440799 | high | 18.00 | 0.000231 |
| GDF9 | 2661 | chr5 | 132865200 | 132868999 | high | 9.50 | 0.000020 |
| GDI2 | 2665 | chr10 | 5810200 | 5813799 | low | -15.65 | 0.000459 |
| GDPD4 | 220032 | chr11 | 77264600 | 77272199 | high | 19.00 | 0.002848 |
| GDPD4 | 220032 | chr11 | 77246000 | 77254399 | high | 21.00 | 0.002848 |
| GEN1 | 348654 | chr2 | 17763600 | 17767799 | low | -18.21 | 0.019627 |
| GFM1 | 85476 | chr3 | 158644800 | 158647399 | low | -11.30 | 0.000539 |
| GFM2 | 84340 | chr5 | 74766000 | 74769799 | low | -16.50 | 0.001128 |
| GFOD2 | 81577 | chr16 | 67683200 | 67685999 | high | 7.00 | 0.001205 |
| GFOD2 | 81577 | chr16 | 67716800 | 67719799 | high | 7.50 | 0.001205 |
| GFPT1 | 2673 | chr2 | 69333200 | 69337399 | high | 10.50 | 0.000039 |
| GFPT1 | 2673 | chr2 | 69343200 | 69348199 | high | 12.50 | 0.000039 |
| GFPT1 | 2673 | chr2 | 69320200 | 69327799 | high | 19.00 | 0.000039 |
| GGA2 | 23062 | chr16 | 23508000 | 23510399 | low | -10.43 | 0.000981 |
| GGCT | 79017 | chr7 | 30502000 | 30505399 | high | 8.50 | 0.002591 |
| GGCX | 2677 | chr2 | 85559800 | 85562199 | high | 6.00 | 0.000031 |
| GGCX | 2677 | chr2 | 85549400 | 85556399 | high | 17.50 | 0.000031 |
| GGH | 8836 | chr8 | 63037000 | 63040199 | high | 8.00 | 0.000140 |
| GGN | 199720 | chr19 | 38382800 | 38386199 | high | 8.50 | 0.005203 |
| GGPS1 | 9453 | chr1 | 235325800 | 235329599 | high | 9.50 | 0.000040 |

| | | | | | | | |
|---|---|---|---|---|---|---|---|
| GGT7 | 2686 | chr20 | 34872800 | 34877399 | high | 11.50 | 0.000077 |
| GHDC | 84514 | chr17 | 42192400 | 42194599 | high | 5.50 | 0.002003 |
| GID4 | 79018 | chr17 | 18044600 | 18046599 | high | 5.00 | 0.004379 |
| GID4 | 79018 | chr17 | 18065600 | 18070999 | high | 13.50 | 0.004374 |
| GID8 | 54994 | chr20 | 62936600 | 62939999 | high | 8.50 | 0.000874 |
| GINS1 | 9837 | chr20 | 25419200 | 25422399 | low | -13.91 | 0.000387 |
| GIPC1 | 10755 | chr19 | 14494200 | 14496999 | high | 7.00 | 0.000742 |
| GIT1 | 28964 | chr17 | 29583200 | 29586399 | high | 8.00 | 0.000979 |
| GIT1 | 28964 | chr17 | 29576200 | 29579399 | high | 8.00 | 0.000979 |
| GIT1 | 28964 | chr17 | 29570200 | 29575399 | high | 13.00 | 0.000979 |
| GIT2 | 9815 | chr12 | 109995600 | 109996799 | low | -5.21 | 0.000089 |
| GJA9 | 81025 | chr1 | 38872000 | 38876599 | high | 11.50 | 0.002084 |
| GK5 | 256356 | chr3 | 142224400 | 142226599 | low | -9.56 | 0.001802 |
| GLB1L | 79411 | chr2 | 219243400 | 219245999 | high | 6.50 | 0.000362 |
| GLCCI1 | 113263 | chr7 | 7968200 | 7973399 | high | 13.00 | 0.014214 |
| GLCE | 26035 | chr15 | 69219800 | 69223999 | high | 10.50 | 0.000376 |
| GLCE | 26035 | chr15 | 69199200 | 69203999 | high | 12.00 | 0.000376 |
| GLCE | 26035 | chr15 | 69158800 | 69165399 | high | 16.50 | 0.000376 |
| GLDC | 2731 | chr9 | 6560000 | 6562799 | high | 7.00 | 0.000416 |
| GLDC | 2731 | chr9 | 6563600 | 6568799 | high | 13.00 | 0.000416 |
| GLDN | 342035 | chr15 | 51401200 | 51404799 | high | 9.00 | 0.006654 |
| GLDN | 342035 | chr15 | 51392200 | 51395799 | high | 9.00 | 0.006655 |
| GLI1 | 2735 | chr12 | 57469600 | 57475999 | high | 16.00 | 0.000048 |
| GLI3 | 2737 | chr7 | 42234800 | 42237799 | high | 7.50 | 0.000065 |
| GLI3 | 2737 | chr7 | 42159200 | 42162999 | high | 9.50 | 0.000065 |
| GLI3 | 2737 | chr7 | 41970800 | 41974599 | high | 9.50 | 0.000065 |
| GLI3 | 2737 | chr7 | 42097600 | 42101799 | high | 10.50 | 0.000065 |
| GLI3 | 2737 | chr7 | 41996400 | 42000599 | high | 10.50 | 0.000065 |
| GLI3 | 2737 | chr7 | 42109800 | 42114199 | high | 11.00 | 0.000065 |
| GLI3 | 2737 | chr7 | 42028200 | 42032799 | high | 11.50 | 0.000065 |
| GLI3 | 2737 | chr7 | 42045800 | 42050999 | high | 13.00 | 0.000065 |
| GLI3 | 2737 | chr7 | 42152000 | 42157399 | high | 13.50 | 0.000065 |
| GLI3 | 2737 | chr7 | 41976000 | 41982399 | high | 16.00 | 0.000065 |
| GLIS3 | 169792 | chr9 | 4193800 | 4196399 | high | 6.50 | 0.004498 |
| GLIS3 | 169792 | chr9 | 4260800 | 4263599 | high | 7.00 | 0.004428 |
| GLIS3 | 169792 | chr9 | 4085400 | 4088599 | high | 8.00 | 0.004618 |
| GLIS3 | 169792 | chr9 | 4078800 | 4081999 | high | 8.00 | 0.004625 |
| GLIS3 | 169792 | chr9 | 3853200 | 3856399 | high | 8.00 | 0.004896 |
| GLIS3 | 169792 | chr9 | 4241800 | 4245399 | high | 9.00 | 0.004448 |
| GLIS3 | 169792 | chr9 | 3902600 | 3906399 | high | 9.50 | 0.003195 |
| GLIS3 | 169792 | chr9 | 4114800 | 4118999 | high | 10.50 | 0.006373 |
| GLIS3 | 169792 | chr9 | 4045000 | 4049599 | high | 11.50 | 0.004664 |
| GLIS3 | 169792 | chr9 | 3864800 | 3869599 | high | 12.00 | 0.004881 |
| GLIS3 | 169792 | chr9 | 4024400 | 4029799 | high | 13.50 | 0.004688 |
| GLIS3 | 169792 | chr9 | 4269200 | 4274999 | high | 14.50 | 0.007397 |
| GLIS3 | 169792 | chr9 | 3985600 | 3991599 | high | 15.00 | 0.004733 |
| GLIS3 | 169792 | chr9 | 3877200 | 3883199 | high | 15.00 | 0.004866 |
| GLIS3 | 169792 | chr9 | 3842600 | 3848999 | high | 16.00 | 0.004910 |
| GLMN | 11146 | chr1 | 92297400 | 92300199 | low | -12.17 | 0.000121 |
| GLOD4 | 51031 | chr17 | 780200 | 783199 | low | -13.04 | 0.007268 |
| GLRB | 2743 | chr4 | 157138800 | 157141999 | low | -13.91 | 0.000017 |
| GLRX2 | 51022 | chr1 | 193103400 | 193105799 | low | -10.43 | 0.000264 |
| GLS | 2744 | chr2 | 190938800 | 190941799 | high | 7.50 | 0.000014 |
| GLS | 2744 | chr2 | 190933600 | 190937199 | high | 9.00 | 0.000014 |
| GLS | 2744 | chr2 | 190959400 | 190964999 | high | 14.00 | 0.000014 |
| GLTP | 51228 | chr12 | 109861800 | 109869399 | high | 19.00 | 0.000466 |
| GLUD1 | 2746 | chr10 | 87066800 | 87069399 | low | -11.30 | 0.000032 |
| GLYR1 | 84656 | chr16 | 4801600 | 4804199 | high | 6.50 | 0.017631 |
| GLYR1 | 84656 | chr16 | 4846400 | 4850399 | high | 10.00 | 0.017468 |
| GMDS | 2762 | chr6 | 2168200 | 2170599 | low | -10.43 | 0.001274 |
| GMEB1 | 10691 | chr1 | 28667800 | 28671599 | high | 9.50 | 0.000373 |
| GMIP | 51291 | chr19 | 19642000 | 19643999 | low | -8.69 | 0.002611 |
| GMPPB | 29925 | chr3 | 49720800 | 49724599 | high | 9.50 | 0.000602 |
| GMPR2 | 51292 | chr14 | 24230800 | 24235399 | high | 11.50 | 0.002117 |
| GNA11 | 2767 | chr19 | 3119800 | 3123799 | high | 10.00 | 0.000887 |
| GNA12 | 2768 | chr7 | 2807400 | 2811199 | high | 9.50 | 0.000986 |
| GNA12 | 2768 | chr7 | 2759400 | 2764999 | high | 14.00 | 0.001003 |
| GNA12 | 2768 | chr7 | 2780600 | 2786999 | high | 16.00 | 0.000995 |
| GNAI3 | 2773 | chr1 | 109591400 | 109593999 | low | -11.30 | 0.000025 |
| GNAL | 2774 | chr18 | 11850400 | 11852599 | low | -9.56 | 0.000234 |
| GNAO1 | 2775 | chr16 | 56354800 | 56358399 | high | 9.00 | 0.000049 |
| GNAQ | 2776 | chr9 | 77957600 | 77961199 | low | -15.65 | 0.000036 |
| GNAQ | 2776 | chr9 | 77947400 | 77949599 | low | -9.56 | 0.000036 |
| GNAT2 | 2780 | chr1 | 109603000 | 109605999 | high | 7.50 | 0.000025 |
| GNAT3 | 346562 | chr7 | 80465400 | 80470399 | high | 12.50 | 0.004307 |
| GNB1 | 2782 | chr1 | 1832200 | 1835599 | high | 8.50 | 0.001518 |
| GNB1 | 2782 | chr1 | 1791000 | 1795599 | high | 11.50 | 0.001553 |
| GNB1 | 2782 | chr1 | 1842600 | 1850199 | high | 19.00 | 0.001510 |

| | | | | | | | |
|---|---|---|---|---|---|---|---|
| GNB1L | 54584 | chr22 | 19829200 | 19832999 | high | 9.50 | 0.002753 |
| GNB2 | 2783 | chr7 | 100673400 | 100676199 | low | -12.17 | 0.000028 |
| GNB4 | 59345 | chr3 | 179431200 | 179436799 | high | 14.00 | 0.000331 |
| GNB5 | 10681 | chr15 | 52178200 | 52182999 | high | 12.00 | 0.000205 |
| GNB5 | 10681 | chr15 | 52146400 | 52151199 | high | 12.00 | 0.000205 |
| GNB5 | 10681 | chr15 | 52125400 | 52130999 | high | 14.00 | 0.000205 |
| GNE | 10020 | chr9 | 36217200 | 36221999 | high | 12.00 | 0.000277 |
| GNG10 | 2790 | chr9 | 111658200 | 111662599 | high | 11.00 | 0.000025 |
| GNG12 | 55970 | chr1 | 67723200 | 67726999 | high | 9.50 | 0.000826 |
| GNG12 | 55970 | chr1 | 67704600 | 67708399 | high | 9.50 | 0.000827 |
| GNG12 | 55970 | chr1 | 67735600 | 67740199 | high | 11.50 | 0.000826 |
| GNG2 | 54331 | chr14 | 51966800 | 51969799 | high | 7.50 | 0.001045 |
| GNG2 | 54331 | chr14 | 51935400 | 51939199 | high | 9.50 | 0.001046 |
| GNG2 | 54331 | chr14 | 51958600 | 51965999 | high | 18.50 | 0.001046 |
| GNG5 | 2787 | chr1 | 84504800 | 84507199 | high | 6.00 | 0.000033 |
| GNG5 | 2787 | chr1 | 84499400 | 84508999 | high | 24.00 | 0.000033 |
| GNG7 | 2788 | chr19 | 2588600 | 2593799 | high | 13.00 | 0.001077 |
| GRHL1 | 29841 | chr2 | 9968400 | 9969799 | low | -6.08 | 0.002994 |
| GRIA1 | 2890 | chr5 | 153553600 | 153557999 | high | 11.00 | 0.000019 |
| GRIA4 | 2893 | chr11 | 105780800 | 105783599 | high | 7.00 | 0.000027 |
| GRIN3B | 116444 | chr19 | 1008000 | 1010199 | high | 5.50 | 0.012836 |
| GRIP1 | 23426 | chr12 | 66420200 | 66422799 | low | -11.30 | 0.000353 |
| GRK4 | 2868 | chr4 | 3020000 | 3024999 | high | 12.50 | 0.000950 |
| GRK5 | 2869 | chr10 | 119398400 | 119402199 | high | 9.50 | 0.000024 |
| GRK5 | 2869 | chr10 | 119416800 | 119420799 | high | 10.00 | 0.000024 |
| GRK5 | 2869 | chr10 | 119267800 | 119271799 | high | 10.00 | 0.000024 |
| GRK5 | 2869 | chr10 | 119442800 | 119446999 | high | 10.50 | 0.000024 |
| GRK5 | 2869 | chr10 | 119372400 | 119377999 | high | 14.00 | 0.000024 |
| GRK5 | 2869 | chr10 | 119205400 | 119210999 | high | 14.00 | 0.000024 |
| GRK5 | 2869 | chr10 | 119307600 | 119316999 | high | 23.50 | 0.000024 |
| GRM1 | 2911 | chr6 | 146329200 | 146332599 | high | 8.50 | 0.000020 |
| GRM1 | 2911 | chr6 | 146305200 | 146312599 | high | 18.50 | 0.000020 |
| GRN | 2896 | chr17 | 44344400 | 44346399 | high | 5.00 | 0.000065 |
| GRP | 2922 | chr18 | 59227800 | 59231799 | high | 10.00 | 0.000049 |
| GRP | 2922 | chr18 | 59218200 | 59223599 | high | 13.50 | 0.000049 |
| GRPEL1 | 80273 | chr4 | 7066000 | 7069199 | high | 8.00 | 0.011360 |
| GRWD1 | 83743 | chr19 | 48445400 | 48446399 | low | -4.34 | 0.001729 |
| GSAP | 54103 | chr7 | 77401400 | 77405199 | high | 9.50 | 0.000699 |
| GSDMB | 55876 | chr17 | 39915200 | 39918799 | high | 9.00 | 0.001400 |
| GSG1 | 83445 | chr12 | 13099000 | 13105799 | low | -29.56 | 0.006370 |
| GSG1 | 83445 | chr12 | 13082600 | 13088799 | low | -26.95 | 0.006378 |
| GSK3A | 2931 | chr19 | 42241000 | 42243199 | low | -9.56 | 0.000069 |
| GSK3B | 2932 | chr3 | 119911600 | 119914399 | high | 7.00 | 0.000024 |
| GSK3B | 2932 | chr3 | 119978800 | 119982199 | high | 8.50 | 0.000024 |
| GSK3B | 2932 | chr3 | 119847200 | 119851799 | high | 11.50 | 0.000024 |
| GSK3B | 2932 | chr3 | 119881000 | 119885799 | high | 12.00 | 0.000024 |
| GSK3B | 2932 | chr3 | 119863000 | 119868799 | high | 14.50 | 0.000024 |
| GSN | 2934 | chr9 | 121318600 | 121321399 | high | 7.00 | 0.000024 |
| GSN | 2934 | chr9 | 121300800 | 121305399 | high | 11.50 | 0.000024 |
| GSN | 2934 | chr9 | 121267200 | 121281399 | high | 35.50 | 0.000024 |
| GSR | 2936 | chr8 | 30718800 | 30729199 | high | 26.00 | 0.000096 |
| GSS | 2937 | chr20 | 34955200 | 34958599 | high | 8.50 | 0.000084 |
| GSTA4 | 2941 | chr6 | 52993000 | 52996799 | low | -16.70 | 0.000055 |
| GSTCD | 79807 | chr4 | 105806000 | 105808599 | low | -11.30 | 0.000754 |
| GSTP1 | 2950 | chr11 | 67581800 | 67586599 | high | 12.00 | 0.000044 |
| GSX2 | 170825 | chr4 | 54099600 | 54101199 | low | -6.95 | 0.003158 |
| GTDC1 | 79712 | chr2 | 144158000 | 144159799 | high | 4.50 | 0.000553 |
| GTDC1 | 79712 | chr2 | 143992800 | 143996399 | high | 9.00 | 0.000554 |
| GTDC1 | 79712 | chr2 | 144165000 | 144169599 | high | 11.50 | 0.000553 |
| GTDC1 | 79712 | chr2 | 144069200 | 144074399 | high | 13.00 | 0.000553 |
| GTDC1 | 79712 | chr2 | 143997400 | 144003199 | high | 14.50 | 0.000554 |
| GTDC1 | 79712 | chr2 | 144095200 | 144101199 | high | 15.00 | 0.000553 |
| GTDC1 | 79712 | chr2 | 143958800 | 143964999 | high | 15.50 | 0.000554 |
| GTDC1 | 79712 | chr2 | 144147600 | 144156999 | high | 23.50 | 0.000553 |
| GTDC1 | 79712 | chr2 | 144075200 | 144085999 | high | 27.00 | 0.000553 |
| GTF2A1 | 2957 | chr14 | 81219600 | 81221599 | high | 5.00 | 0.000036 |
| GTF2E2 | 2961 | chr8 | 30612400 | 30615799 | high | 8.50 | 0.000097 |
| GTF2E2 | 2961 | chr8 | 30603200 | 30608399 | high | 13.00 | 0.000097 |
| GTF2F1 | 2962 | chr19 | 6390800 | 6394199 | high | 8.50 | 0.000463 |
| GTF2F2 | 2963 | chr13 | 45180200 | 45183799 | high | 9.00 | 0.000066 |
| GTF2F2 | 2963 | chr13 | 45191400 | 45195199 | high | 9.50 | 0.000066 |
| GTF2F2 | 2963 | chr13 | 45171600 | 45175399 | high | 9.50 | 0.000066 |
| GTF2F2 | 2963 | chr13 | 45133200 | 45137599 | high | 11.00 | 0.000066 |
| GTF2F2 | 2963 | chr13 | 45158800 | 45163599 | high | 12.00 | 0.000066 |
| GTF2F2 | 2963 | chr13 | 45184600 | 45189799 | high | 13.00 | 0.000066 |
| GTF2H3 | 2967 | chr12 | 123655000 | 123657799 | high | 7.00 | 0.000024 |
| GTF2I | 2969 | chr7 | 74723800 | 74726799 | high | 7.50 | 0.000040 |
| GTF3C1 | 2975 | chr16 | 27538400 | 27543799 | high | 13.50 | 0.000108 |

| | | | | | | | |
|---|---|---|---|---|---|---|---|
| GTF3C1 | 2975 | chr16 | 27512200 | 27518199 | high | 15.00 | 0.000108 |
| GTF3C4 | 9329 | chr9 | 132669200 | 132671999 | high | 7.00 | 0.000070 |
| GTF3C5 | 9328 | chr9 | 133029400 | 133033399 | high | 10.00 | 0.000070 |
| GTF3C6 | 112495 | chr6 | 110957000 | 110960199 | high | 8.00 | 0.001014 |
| GTPBP1 | 9567 | chr22 | 38721600 | 38724399 | high | 7.00 | 0.000247 |
| GTPBP3 | 84705 | chr19 | 17336000 | 17340999 | high | 12.50 | 0.004886 |
| GTPBP8 | 29083 | chr3 | 112991400 | 112994199 | low | -12.17 | 0.000257 |
| GTSE1 | 51512 | chr22 | 46295600 | 46298799 | high | 8.00 | 0.001113 |
| GUCA1A | 2978 | chr6 | 42167800 | 42174399 | high | 16.50 | 0.000071 |
| GUCD1 | 83606 | chr22 | 24552600 | 24557399 | low | -20.17 | 0.003405 |
| GUCD1 | 83606 | chr22 | 24554200 | 24558399 | low | -18.64 | 0.003405 |
| GUCY2D | 3000 | chr17 | 8009000 | 8013799 | high | 12.00 | 0.000375 |
| GUCY2D | 3000 | chr17 | 8016400 | 8021599 | high | 13.00 | 0.000374 |
| GUK1 | 2987 | chr1 | 228139600 | 228141199 | low | -6.95 | 0.000013 |
| GULP1 | 51454 | chr2 | 188549400 | 188553199 | high | 9.50 | 0.000273 |
| GULP1 | 51454 | chr2 | 188468200 | 188474599 | high | 16.00 | 0.000273 |
| GULP1 | 51454 | chr2 | 188488200 | 188495599 | high | 18.50 | 0.000273 |
| GUSB | 2990 | chr7 | 65959600 | 65964399 | high | 12.00 | 0.000045 |
| GXYLT1 | 283464 | chr12 | 42142200 | 42144599 | low | -10.43 | 0.006726 |
| GXYLT2 | 727936 | chr3 | 72890000 | 72894399 | low | -19.68 | 0.009987 |
| GZF1 | 64412 | chr20 | 23361400 | 23363399 | high | 5.00 | 0.002757 |
| GZF1 | 64412 | chr20 | 23365800 | 23370599 | high | 12.00 | 0.002757 |
| H1F0 | 3005 | chr22 | 37802200 | 37808599 | low | -27.82 | 0.000079 |
| H1F0 | 3005 | chr22 | 37804200 | 37808799 | low | -19.93 | 0.000079 |
| H1FX | 8971 | chr3 | 129315200 | 129318799 | low | -15.65 | 0.000069 |
| H2AFJ | 55766 | chr12 | 14774400 | 14776599 | low | -9.56 | 0.003775 |
| H2AFY | 9555 | chr5 | 135396200 | 135401199 | high | 12.50 | 0.000071 |
| H2AFY2 | 55506 | chr10 | 70050000 | 70057599 | high | 19.00 | 0.000792 |
| H2AFZ | 3015 | chr4 | 99949200 | 99950999 | low | -7.82 | 0.000030 |
| H3F3B | 3021 | chr17 | 75778800 | 75780799 | low | -8.69 | 0.000040 |
| H6PD | 9563 | chr1 | 9234800 | 9237799 | high | 7.50 | 0.001036 |
| H6PD | 9563 | chr1 | 9239200 | 9246599 | high | 18.50 | 0.001035 |
| HABP4 | 22927 | chr9 | 96469800 | 96473999 | high | 10.50 | 0.000238 |
| HACD1 | 9200 | chr10 | 17615000 | 17618199 | low | -13.91 | 0.000522 |
| HACD3 | 51495 | chr15 | 65529200 | 65531999 | low | -12.17 | 0.000786 |
| HACD4 | 401494 | chr9 | 21029000 | 21031199 | low | -9.56 | 0.019092 |
| HACE1 | 57531 | chr6 | 104854400 | 104860999 | high | 16.50 | 0.000549 |
| HACL1 | 26061 | chr3 | 15597800 | 15602999 | high | 13.00 | 0.001671 |
| HADH | 3033 | chr4 | 108017400 | 108019999 | low | -11.30 | 0.000028 |
| HADH | 3033 | chr4 | 107994600 | 107995999 | low | -6.08 | 0.000028 |
| HADHA | 3030 | chr2 | 26235000 | 26240399 | high | 13.50 | 0.000115 |
| HAGH | 3029 | chr16 | 1823400 | 1830199 | high | 17.00 | 0.001661 |
| HAGHL | 84264 | chr16 | 727200 | 732399 | high | 13.00 | 0.008746 |
| HAPLN1 | 1404 | chr5 | 83667800 | 83670599 | high | 7.00 | 0.000017 |
| HAPLN3 | 145864 | chr15 | 88885200 | 88890599 | high | 13.50 | 0.001641 |
| HARBI1 | 283254 | chr11 | 46615800 | 46618599 | high | 7.00 | 0.006076 |
| HAS1 | 3036 | chr19 | 51713000 | 51719399 | high | 16.00 | 0.000059 |
| HAS2 | 3037 | chr8 | 121617000 | 121618599 | high | 4.00 | 0.000025 |
| HAT1 | 8520 | chr2 | 171976200 | 171982799 | high | 16.50 | 0.000050 |
| HAUS2 | 55142 | chr15 | 42545000 | 42550999 | high | 15.00 | 0.001296 |
| HAUS3 | 79441 | chr4 | 2240600 | 2241999 | low | -6.08 | 0.003939 |
| HAUS4 | 54930 | chr14 | 22955000 | 22957199 | high | 5.50 | 0.002393 |
| HBEGF | 1839 | chr5 | 140344600 | 140347799 | high | 8.00 | 0.000013 |
| HBEGF | 1839 | chr5 | 140337200 | 140342599 | high | 13.50 | 0.000013 |
| HBS1L | 10767 | chr6 | 135019800 | 135023999 | high | 10.50 | 0.000080 |
| HBS1L | 10767 | chr6 | 135036600 | 135042199 | high | 14.00 | 0.000080 |
| HDAC10 | 83933 | chr22 | 50243800 | 50247399 | high | 9.00 | 0.001671 |
| HDAC4 | 9759 | chr2 | 239327200 | 239330799 | high | 9.00 | 0.000041 |
| HDAC7 | 51564 | chr12 | 47813200 | 47817799 | low | -19.55 | 0.001078 |
| HDAC9 | 9734 | chr7 | 18368400 | 18373199 | low | -20.91 | 0.000530 |
| HDAC9 | 9734 | chr7 | 18398200 | 18400799 | low | -11.30 | 0.000529 |
| HDLBP | 3069 | chr2 | 241240400 | 241243599 | high | 8.00 | 0.000013 |
| HEG1 | 57493 | chr3 | 125004600 | 125008599 | high | 10.00 | 0.000460 |
| HEG1 | 57493 | chr3 | 124982200 | 124986199 | high | 10.00 | 0.000460 |
| HELB | 92797 | chr12 | 66337000 | 66341799 | high | 12.00 | 0.001399 |
| HELLS | 3070 | chr10 | 94544400 | 94547999 | high | 9.00 | 0.000032 |
| HELZ | 9931 | chr17 | 67160000 | 67163399 | low | -14.78 | 0.000148 |
| HELZ | 9931 | chr17 | 67089600 | 67092199 | low | -11.30 | 0.000148 |
| HELZ | 9931 | chr17 | 67153200 | 67155399 | low | -9.56 | 0.000148 |
| HFE2 | 148738 | chr1 | 146016200 | 146020799 | high | 11.50 | 0.001019 |
| HGF | 3082 | chr7 | 81748800 | 81751999 | high | 8.00 | 0.000038 |
| HGF | 3082 | chr7 | 81713600 | 81717999 | high | 11.00 | 0.000038 |
| HGF | 3082 | chr7 | 81730600 | 81735199 | high | 11.50 | 0.000038 |
| HGF | 3082 | chr7 | 81697200 | 81704999 | high | 19.50 | 0.000038 |
| HGF | 3082 | chr7 | 81756400 | 81764399 | high | 20.00 | 0.000038 |
| HGF | 3082 | chr7 | 81765200 | 81774799 | high | 24.00 | 0.000038 |
| HHEX | 3087 | chr10 | 92689200 | 92690999 | low | -7.82 | 0.000033 |
| HHIPL2 | 79802 | chr1 | 222521200 | 222523399 | low | -9.56 | 0.000359 |

| | | | | | | | |
|---|---|---|---|---|---|---|---|
| HHLA3 | 11147 | chr1 | 70353200 | 70355399 | high | 5.50 | 0.000158 |
| HIBCH | 26275 | chr2 | 190311400 | 190313999 | low | -11.30 | 0.000138 |
| HIF1A | 3091 | chr14 | 61743800 | 61749999 | low | -26.13 | 0.000050 |
| HIF1A | 3091 | chr14 | 61737400 | 61740399 | low | -13.04 | 0.000050 |
| HINT1 | 3094 | chr5 | 131161400 | 131168999 | high | 19.00 | 0.000024 |
| HINT2 | 84681 | chr9 | 35814200 | 35815199 | high | 2.50 | 0.002364 |
| HINT3 | 135114 | chr6 | 125955800 | 125957999 | low | -9.56 | 0.001073 |
| HIP1 | 3092 | chr7 | 75639400 | 75643199 | high | 9.50 | 0.000041 |
| HIP1 | 3092 | chr7 | 75712600 | 75717199 | high | 11.50 | 0.000041 |
| HIP1 | 3092 | chr7 | 75617600 | 75623599 | high | 15.00 | 0.000041 |
| HIP1R | 9026 | chr12 | 122859800 | 122863599 | high | 9.50 | 0.000073 |
| HIPK2 | 28996 | chr7 | 139698000 | 139700799 | high | 7.00 | 0.000208 |
| HIPK2 | 28996 | chr7 | 139657000 | 139661199 | high | 10.50 | 0.000208 |
| HIPK2 | 28996 | chr7 | 139639800 | 139644799 | high | 12.50 | 0.000208 |
| HIPK2 | 28996 | chr7 | 139616400 | 139621599 | high | 13.00 | 0.000208 |
| HIPK2 | 28996 | chr7 | 139592200 | 139598199 | high | 15.00 | 0.000208 |
| HIPK2 | 28996 | chr7 | 139752400 | 139758599 | high | 15.50 | 0.000207 |
| HIPK2 | 28996 | chr7 | 139682000 | 139690599 | high | 21.50 | 0.000208 |
| HIPK3 | 10114 | chr11 | 33338600 | 33344399 | high | 14.50 | 0.000303 |
| HIRA | 7290 | chr22 | 19403000 | 19407199 | high | 10.50 | 0.000376 |
| HIRIP3 | 8479 | chr16 | 29992400 | 29996999 | high | 11.50 | 0.000283 |
| HJURP | 55355 | chr2 | 233850400 | 233855399 | high | 12.50 | 0.000237 |
| HK1 | 3098 | chr10 | 69382200 | 69384599 | high | 6.00 | 0.000045 |
| HK1 | 3098 | chr10 | 69326800 | 69331199 | high | 11.00 | 0.000045 |
| HK2 | 3099 | chr2 | 74849600 | 74851799 | low | -9.56 | 0.000041 |
| HKDC1 | 80201 | chr10 | 69226800 | 69230399 | high | 9.00 | 0.001159 |
| HLCS | 3141 | chr21 | 36843200 | 36845399 | high | 5.50 | 0.000085 |
| HLCS | 3141 | chr21 | 36909200 | 36912599 | high | 8.50 | 0.000085 |
| HLTF | 6596 | chr3 | 149039400 | 149044399 | high | 12.50 | 0.000044 |
| HLTF | 6596 | chr3 | 149051400 | 149056799 | high | 13.50 | 0.000044 |
| HLX | 3142 | chr1 | 220878000 | 220879999 | low | -8.69 | 0.000014 |
| HM13 | 81502 | chr20 | 31546400 | 31550799 | high | 11.00 | 0.002584 |
| HM13 | 81502 | chr20 | 31568200 | 31574399 | high | 15.50 | 0.002582 |
| HN1 | 51155 | chr17 | 75152200 | 75154599 | high | 6.00 | 0.000681 |
| HN1 | 51155 | chr17 | 75146000 | 75149199 | high | 8.00 | 0.000681 |
| HNF4A | 3172 | chr20 | 44398200 | 44400399 | low | -9.56 | 0.000071 |
| HNRNPA0 | 10949 | chr5 | 137752000 | 137754999 | low | -13.04 | 0.000079 |
| HNRNPA1 | 3178 | chr12 | 54278200 | 54281999 | low | -16.51 | 0.000059 |
| HNRNPA3 | 220988 | chr2 | 177212400 | 177213999 | low | -6.95 | 0.001247 |
| HNRNPC | 3183 | chr14 | 21267800 | 21269599 | low | -7.82 | 0.000150 |
| HNRNPD | 3184 | chr4 | 82371000 | 82375599 | high | 11.50 | 0.000039 |
| HNRNPK | 3190 | chr9 | 83977200 | 83983799 | high | 16.50 | 0.000038 |
| HNRNPM | 4670 | chr19 | 8453800 | 8456799 | high | 7.50 | 0.000552 |
| HNRNPR | 10236 | chr1 | 23302000 | 23308999 | low | -30.47 | 0.000439 |
| HNRNPUL2 | 221092 | chr11 | 62718200 | 62723599 | high | 13.50 | 0.003525 |
| HOMER1 | 9456 | chr5 | 79511200 | 79515399 | high | 10.50 | 0.000119 |
| HOMER1 | 9456 | chr5 | 79442000 | 79449599 | high | 19.00 | 0.000119 |
| HOMEZ | 57594 | chr14 | 23283600 | 23286599 | high | 7.50 | 0.002474 |
| HOOK3 | 84376 | chr8 | 42997600 | 43000799 | high | 8.00 | 0.001962 |
| HOOK3 | 84376 | chr8 | 42911800 | 42914999 | high | 8.00 | 0.001966 |
| HOOK3 | 84376 | chr8 | 43025200 | 43029599 | high | 11.00 | 0.001961 |
| HOPX | 84525 | chr4 | 56653600 | 56657799 | high | 10.50 | 0.001492 |
| HORMAD1 | 84072 | chr1 | 150697000 | 150700799 | high | 9.50 | 0.000558 |
| HORMAD1 | 84072 | chr1 | 150711000 | 150715799 | high | 12.00 | 0.000558 |
| HP09053 | 101929357 | chr3 | 99817000 | 99825199 | high | 20.50 | 0.007004 |
| HP1BP3 | 50809 | chr1 | 20785400 | 20787199 | low | -7.82 | 0.002444 |
| HPCAL1 | 3241 | chr2 | 10405400 | 10408999 | high | 9.00 | 0.000311 |
| HPCAL1 | 3241 | chr2 | 10323200 | 10326799 | high | 9.00 | 0.000314 |
| HPD | 3242 | chr12 | 121844400 | 121847199 | high | 7.00 | 0.000027 |
| HPD | 3242 | chr12 | 121838800 | 121841799 | high | 7.50 | 0.000027 |
| HPD | 3242 | chr12 | 121869400 | 121874199 | high | 12.00 | 0.000027 |
| HPF1 | 54969 | chr4 | 169755400 | 169757799 | low | -10.43 | 0.000324 |
| HPS1 | 3257 | chr10 | 98444600 | 98448199 | high | 9.00 | 0.000033 |
| HPS3 | 84343 | chr3 | 149168000 | 149170799 | high | 7.00 | 0.000565 |
| HPS3 | 84343 | chr3 | 149129200 | 149131999 | high | 7.00 | 0.000566 |
| HPS5 | 11234 | chr11 | 18321400 | 18324199 | low | -12.17 | 0.000613 |
| HRAT17 | 101928036 | chr7 | 112965000 | 112967599 | high | 6.50 | 0.000091 |
| HRH1 | 3269 | chr3 | 11154400 | 11158999 | high | 11.50 | 0.000293 |
| HRH1 | 3269 | chr3 | 11196800 | 11201999 | high | 13.00 | 0.000292 |
| HS1BP3 | 64342 | chr2 | 20646000 | 20652199 | high | 15.50 | 0.003116 |
| HS3ST3A1 | 9955 | chr17 | 13600200 | 13603399 | high | 8.00 | 0.000732 |
| HS3ST3A1 | 9955 | chr17 | 13531000 | 13534999 | high | 10.00 | 0.000736 |
| HS3ST3A1 | 9955 | chr17 | 13536000 | 13541799 | high | 14.50 | 0.000735 |
| HS3ST3A1 | 9955 | chr17 | 13566600 | 13575599 | high | 22.50 | 0.000734 |
| HS3ST3B1 | 9953 | chr17 | 14300600 | 14304199 | high | 9.00 | 0.000696 |
| HS6ST1 | 9394 | chr2 | 128299200 | 128305599 | high | 16.00 | 0.000073 |
| HSCB | 150274 | chr22 | 28740200 | 28744399 | low | -18.21 | 0.005229 |
| HSD17B1 | 3292 | chr17 | 42551000 | 42556799 | high | 14.50 | 0.000077 |

| | | | | | | | |
|---|---|---|---|---|---|---|---|
| HSD17B11 | 51170 | chr4 | 87388800 | 87392599 | high | 9.50 | 0.000586 |
| HSD17B14 | 51171 | chr19 | 48835400 | 48837799 | high | 6.00 | 0.001048 |
| HSD17B4 | 3295 | chr5 | 119452000 | 119454799 | high | 7.00 | 0.000028 |
| HSD52 | 729467 | chr1 | 59132000 | 59135199 | high | 8.00 | 0.012336 |
| HSD52 | 729467 | chr1 | 59136000 | 59139999 | high | 10.00 | 0.012335 |
| HSDL2 | 84263 | chr9 | 112378600 | 112381999 | high | 8.50 | 0.000750 |
| HSF1 | 3297 | chr8 | 144290400 | 144293199 | high | 7.00 | 0.000023 |
| HSF2 | 3298 | chr6 | 122399200 | 122400599 | low | -6.08 | 0.000027 |
| HSF4 | 3299 | chr16 | 67159600 | 67167399 | high | 19.50 | 0.000049 |
| HSPA12B | 116835 | chr20 | 3752800 | 3756999 | high | 10.50 | 0.003459 |
| HSPA4 | 3308 | chr5 | 133051400 | 133054599 | high | 8.00 | 0.000025 |
| HSPA4L | 22824 | chr4 | 127783000 | 127785799 | low | -12.17 | 0.000179 |
| HSPA8 | 3312 | chr11 | 123059200 | 123062799 | high | 9.00 | 0.000027 |
| HSPA9 | 3313 | chr5 | 138562800 | 138565799 | high | 7.50 | 0.000024 |
| HSPB1 | 3315 | chr7 | 76302000 | 76305199 | high | 8.00 | 0.000043 |
| HSPBAP1 | 79663 | chr3 | 122789400 | 122796199 | high | 17.00 | 0.000649 |
| HSPG2 | 3339 | chr1 | 21933800 | 21937999 | high | 10.50 | 0.000152 |
| HTR2B | 3357 | chr2 | 231112600 | 231118199 | low | -24.96 | 0.000015 |
| HTR7 | 3363 | chr10 | 90779200 | 90782199 | high | 7.50 | 0.000037 |
| HTR7 | 3363 | chr10 | 90751600 | 90754799 | high | 8.00 | 0.000037 |
| HTR7 | 3363 | chr10 | 90855000 | 90858399 | high | 8.50 | 0.000037 |
| HTR7 | 3363 | chr10 | 90746600 | 90750199 | high | 9.00 | 0.000037 |
| HTR7 | 3363 | chr10 | 90783200 | 90788199 | high | 12.50 | 0.000037 |
| HTR7 | 3363 | chr10 | 90738000 | 90744399 | high | 16.00 | 0.000037 |
| HTRA2 | 27429 | chr2 | 74529000 | 74532399 | high | 8.50 | 0.000368 |
| HTT | 3064 | chr4 | 3225200 | 3228999 | high | 9.50 | 0.000950 |
| HUS1B | 135458 | chr6 | 653200 | 657199 | high | 10.00 | 0.001010 |
| HUWE1 | 10075 | chrX | 53680600 | 53683599 | low | -13.04 | 0.000188 |
| HYAL2 | 8692 | chr3 | 50321000 | 50322399 | low | -6.08 | 0.000173 |
| HYAL3 | 8372 | chr3 | 50287800 | 50294799 | high | 17.50 | 0.000166 |
| HYDIN | 54768 | chr16 | 70807600 | 70810599 | high | 7.50 | 0.000773 |
| HYI | 81888 | chr1 | 43448000 | 43458199 | high | 25.50 | 0.001885 |
| HYPK | 25764 | chr15 | 43799000 | 43802199 | low | -13.91 | 0.000588 |
| IAH1 | 285148 | chr2 | 9474200 | 9479599 | high | 13.50 | 0.003344 |
| IARS | 3376 | chr9 | 92278200 | 92281999 | high | 9.50 | 0.000037 |
| IARS | 3376 | chr9 | 92271000 | 92274999 | high | 10.00 | 0.000037 |
| IARS | 3376 | chr9 | 92243400 | 92250199 | high | 17.00 | 0.000037 |
| ICE2 | 79664 | chr15 | 60443800 | 60448799 | high | 12.50 | 0.001318 |
| ICE2 | 79664 | chr15 | 60427200 | 60432999 | high | 14.50 | 0.001318 |
| ICE2 | 79664 | chr15 | 60416000 | 60425799 | high | 24.50 | 0.001319 |
| ICK | 22858 | chr6 | 53000200 | 53004199 | high | 10.00 | 0.000431 |
| ID3 | 3399 | chr1 | 23558000 | 23559199 | low | -5.21 | 0.000144 |
| IDE | 3416 | chr10 | 92572000 | 92574999 | high | 7.50 | 0.000037 |
| IDH2 | 3418 | chr15 | 90098200 | 90104999 | high | 17.00 | 0.000038 |
| IDI1 | 3422 | chr10 | 1047800 | 1051999 | high | 10.50 | 0.003266 |
| IER5L | 389792 | chr9 | 129173800 | 129178199 | high | 11.00 | 0.003018 |
| IFI6 | 2537 | chr1 | 27668400 | 27673799 | high | 13.50 | 0.000092 |
| IFIH1 | 64135 | chr2 | 162283200 | 162286199 | low | -13.04 | 0.000395 |
| IFIT1 | 3434 | chr10 | 89402600 | 89405599 | high | 7.50 | 0.000038 |
| IFITM3 | 10410 | chr11 | 319200 | 320999 | low | -7.82 | 0.003624 |
| IFNAR1 | 3454 | chr21 | 33324000 | 33326799 | high | 7.00 | 0.000104 |
| IFNAR2 | 3455 | chr21 | 33250200 | 33253199 | low | -13.04 | 0.000104 |
| IFNGR1 | 3459 | chr6 | 137215400 | 137220599 | high | 13.00 | 0.000025 |
| IFRD2 | 7866 | chr3 | 50287800 | 50294799 | high | 17.50 | 0.000156 |
| IFT140 | 9742 | chr16 | 1607800 | 1616199 | high | 21.00 | 0.006059 |
| IFT57 | 55081 | chr3 | 108219000 | 108224799 | high | 14.50 | 0.000509 |
| IGDCC4 | 57722 | chr15 | 65408200 | 65409999 | high | 4.50 | 0.000882 |
| IGF2 | 3481 | chr11 | 2137000 | 2137999 | low | -4.34 | 0.001629 |
| IGF2BP1 | 10642 | chr17 | 49012600 | 49015599 | high | 7.50 | 0.000217 |
| IGF2BP1 | 10642 | chr17 | 49049000 | 49053599 | high | 11.50 | 0.000217 |
| IGF2BP1 | 10642 | chr17 | 48996200 | 49000799 | high | 11.50 | 0.000217 |
| IGF2BP2 | 10644 | chr3 | 185731800 | 185735399 | high | 9.00 | 0.000057 |
| IGF2BP2 | 10644 | chr3 | 185789800 | 185794399 | high | 11.50 | 0.000057 |
| IGF2BP2 | 10644 | chr3 | 185812200 | 185817399 | high | 13.00 | 0.000057 |
| IGF2BP2 | 10644 | chr3 | 185736200 | 185742199 | high | 15.00 | 0.000057 |
| IGF2BP3 | 10643 | chr7 | 23454400 | 23459399 | high | 12.50 | 0.000454 |
| IGF2BP3 | 10643 | chr7 | 23328400 | 23333999 | high | 14.00 | 0.000456 |
| IGF2BP3 | 10643 | chr7 | 23410400 | 23416399 | high | 15.00 | 0.000455 |
| IGF2BP3 | 10643 | chr7 | 23357600 | 23363999 | high | 16.00 | 0.000456 |
| IGF2BP3 | 10643 | chr7 | 23379400 | 23387999 | high | 21.50 | 0.000455 |
| IGF2BP3 | 10643 | chr7 | 23297600 | 23314799 | high | 43.00 | 0.000457 |
| IGFBP6 | 3489 | chr12 | 53096000 | 53099799 | high | 9.50 | 0.000066 |
| IGFBP7 | 3490 | chr4 | 57088800 | 57092399 | low | -15.65 | 0.000061 |
| IGFBP7 | 3490 | chr4 | 57109000 | 57111399 | low | -10.43 | 0.000061 |
| IGSF22 | 283284 | chr11 | 18720200 | 18726599 | high | 16.00 | 0.015133 |
| IGSF6 | 10261 | chr16 | 21651600 | 21656199 | high | 11.50 | 0.000474 |
| IKBIP | 121457 | chr12 | 98643600 | 98646999 | high | 8.50 | 0.001231 |
| IKBKG | 8517 | chrX | 154546600 | 154550599 | high | 10.00 | 0.000055 |

| | | | | | | | |
|---|---|---|---|---|---|---|---|
| IKZF2 | 22807 | chr2 | 213149600 | 213151599 | low | -8.69 | 0.000107 |
| IKZF4 | 64375 | chr12 | 56029000 | 56032599 | high | 9.00 | 0.001149 |
| IL10RB | 3588 | chr21 | 33264800 | 33267999 | low | -13.91 | 0.000108 |
| IL11 | 3589 | chr19 | 55368800 | 55370199 | low | -6.08 | 0.000065 |
| IL13RA2 | 3598 | chrX | 115014200 | 115018399 | high | 10.50 | 0.000031 |
| IL17RC | 84818 | chr3 | 9932400 | 9934599 | high | 5.50 | 0.008540 |
| IL18R1 | 8809 | chr2 | 102385800 | 102386999 | low | -5.21 | 0.000086 |
| IL19 | 29949 | chr1 | 206798200 | 206800199 | low | -8.69 | 0.000145 |
| IL1RAP | 3556 | chr3 | 190606200 | 190607999 | low | -7.82 | 0.000019 |
| IL1RAPL2 | 26280 | chrX | 105334600 | 105337999 | low | -14.78 | 0.000249 |
| IL1RL2 | 8808 | chr2 | 102187800 | 102190599 | low | -12.17 | 0.000086 |
| IL20RB | 53833 | chr3 | 137008200 | 137008999 | low | -3.47 | 0.000393 |
| IL31 | 386653 | chr12 | 122173800 | 122178599 | high | 12.00 | 0.003165 |
| IL31RA | 133396 | chr5 | 55908000 | 55910599 | high | 6.50 | 0.002386 |
| IL31RA | 133396 | chr5 | 55913400 | 55919599 | high | 15.50 | 0.002386 |
| IL4I1 | 259307 | chr19 | 49927400 | 49930799 | high | 8.50 | 0.005194 |
| IL4R | 3566 | chr16 | 27322600 | 27328799 | high | 15.50 | 0.000131 |
| IL4R | 3566 | chr16 | 27312000 | 27319799 | high | 19.50 | 0.000131 |
| IL6 | 3569 | chr7 | 22727200 | 22730599 | high | 8.50 | 0.000157 |
| IL6ST | 3572 | chr5 | 55993400 | 55997199 | high | 9.50 | 0.000064 |
| IL6ST | 3572 | chr5 | 55951200 | 55955999 | high | 12.00 | 0.000064 |
| IL6ST | 3572 | chr5 | 55977800 | 55983199 | high | 13.50 | 0.000064 |
| IL7R | 3575 | chr5 | 35874200 | 35879399 | high | 13.00 | 0.000100 |
| ILKAP | 80895 | chr2 | 238168600 | 238173599 | high | 12.50 | 0.000340 |
| ILKAP | 80895 | chr2 | 238197400 | 238205399 | high | 20.00 | 0.000340 |
| ILVBL | 10994 | chr19 | 15123200 | 15126799 | high | 9.00 | 0.000727 |
| IMMP2L | 83943 | chr7 | 111384400 | 111387399 | low | -13.04 | 0.000754 |
| IMMP2L | 83943 | chr7 | 111130800 | 111132599 | low | -7.82 | 0.000755 |
| IMMP2L | 83943 | chr7 | 111440000 | 111441399 | low | -6.08 | 0.000753 |
| IMMT | 10989 | chr2 | 86145200 | 86150599 | high | 13.50 | 0.000128 |
| IMP3 | 55272 | chr15 | 75639400 | 75640999 | high | 4.00 | 0.000731 |
| IMPDH1 | 3614 | chr7 | 128403200 | 128413599 | high | 26.00 | 0.000028 |
| INF2 | 64423 | chr14 | 104688600 | 104692999 | high | 11.00 | 0.000615 |
| ING5 | 84289 | chr2 | 241700400 | 241703999 | low | -15.65 | 0.000349 |
| INHBA | 3624 | chr7 | 41688000 | 41692399 | high | 11.00 | 0.000087 |
| INO80 | 54617 | chr15 | 40976800 | 40982199 | high | 13.50 | 0.001333 |
| INO80 | 54617 | chr15 | 41000400 | 41009199 | high | 22.00 | 0.001332 |
| INO80E | 283899 | chr16 | 29992400 | 29996999 | high | 11.50 | 0.009466 |
| INPP4B | 8821 | chr4 | 142714000 | 142716199 | high | 5.50 | 0.000062 |
| INPP4B | 8821 | chr4 | 142526000 | 142529199 | high | 8.00 | 0.000062 |
| INPP4B | 8821 | chr4 | 142449200 | 142452999 | high | 9.50 | 0.000062 |
| INPP4B | 8821 | chr4 | 142813800 | 142817799 | high | 10.00 | 0.000062 |
| INPP4B | 8821 | chr4 | 142613400 | 142619399 | high | 15.00 | 0.000062 |
| INPP4B | 8821 | chr4 | 142467000 | 142473999 | high | 17.50 | 0.000062 |
| INPP4B | 8821 | chr4 | 142568000 | 142576799 | high | 22.00 | 0.000062 |
| INPP5B | 3633 | chr1 | 37944600 | 37947599 | high | 7.50 | 0.000096 |
| INPP5F | 22876 | chr10 | 119783800 | 119787799 | high | 10.00 | 0.000191 |
| INPP5F | 22876 | chr10 | 119760000 | 119764599 | high | 11.50 | 0.000191 |
| INPP5K | 51763 | chr17 | 1515400 | 1518199 | high | 7.00 | 0.003795 |
| INPP5K | 51763 | chr17 | 1503800 | 1507399 | high | 9.00 | 0.003825 |
| INSC | 387755 | chr11 | 15118600 | 15120799 | high | 5.50 | 0.002850 |
| INSC | 387755 | chr11 | 15137400 | 15140199 | high | 7.00 | 0.002846 |
| INSC | 387755 | chr11 | 15186600 | 15190799 | high | 10.50 | 0.002837 |
| INTS10 | 55174 | chr8 | 19843200 | 19845599 | low | -10.43 | 0.002780 |
| INTS2 | 57508 | chr17 | 61926800 | 61928199 | high | 3.50 | 0.000929 |
| INTS2 | 57508 | chr17 | 61905000 | 61909199 | high | 10.50 | 0.000929 |
| INTS2 | 57508 | chr17 | 61869800 | 61875999 | high | 15.50 | 0.000930 |
| INTS4 | 92105 | chr11 | 77971000 | 77973999 | high | 7.50 | 0.001181 |
| INTS6 | 26512 | chr13 | 51366200 | 51369599 | low | -14.78 | 0.000516 |
| INTS6 | 26512 | chr13 | 51430400 | 51432799 | low | -10.43 | 0.000515 |
| INTS9 | 55756 | chr8 | 28775200 | 28779999 | high | 12.00 | 0.001938 |
| INVS | 27130 | chr9 | 100104000 | 100106199 | high | 5.50 | 0.000271 |
| INVS | 27130 | chr9 | 100097200 | 100100399 | high | 8.00 | 0.000271 |
| INVS | 27130 | chr9 | 100148400 | 100152799 | high | 11.00 | 0.000271 |
| INVS | 27130 | chr9 | 100130600 | 100136999 | high | 16.00 | 0.000271 |
| IPCEF1 | 26034 | chr6 | 154276400 | 154280399 | high | 10.00 | 0.000169 |
| IPMK | 253430 | chr10 | 58236600 | 58238399 | high | 4.50 | 0.004352 |
| IPMK | 253430 | chr10 | 58265200 | 58269399 | high | 10.50 | 0.004350 |
| IPO11 | 51194 | chr5 | 62562000 | 62567199 | high | 13.00 | 0.000818 |
| IPO7 | 10527 | chr11 | 9398600 | 9401999 | high | 8.50 | 0.001120 |
| IPO8 | 10526 | chr12 | 30693200 | 30697799 | high | 11.50 | 0.000343 |
| IPO9 | 55705 | chr1 | 201856800 | 201860399 | high | 9.00 | 0.000276 |
| IPO9 | 55705 | chr1 | 201848400 | 201852199 | high | 9.50 | 0.000276 |
| IPO9 | 55705 | chr1 | 201841400 | 201845199 | high | 9.50 | 0.000276 |
| IPO9 | 55705 | chr1 | 201832400 | 201836799 | high | 11.00 | 0.000276 |
| IPO9 | 55705 | chr1 | 201866400 | 201871999 | high | 14.00 | 0.000276 |
| IPO9 | 55705 | chr1 | 201824800 | 201831399 | high | 16.50 | 0.000276 |
| IPP | 3652 | chr1 | 45737600 | 45740999 | high | 8.50 | 0.000080 |

| | | | | | | | |
|---|---|---|---|---|---|---|---|
| IPPK | 64768 | chr9 | 92668800 | 92671399 | low | -11.30 | 0.000699 |
| IQCC | 55721 | chr1 | 32203400 | 32206199 | high | 7.00 | 0.001730 |
| IQCC | 55721 | chr1 | 32207000 | 32212199 | high | 13.00 | 0.001730 |
| IQCD | 115811 | chr12 | 113214800 | 113217199 | low | -10.43 | 0.001023 |
| IQCE | 23288 | chr7 | 2557800 | 2560999 | low | -13.91 | 0.009105 |
| IQCJ | 654502 | chr3 | 159129600 | 159133799 | high | 10.50 | 0.004113 |
| IQCJ | 654502 | chr3 | 159193400 | 159197799 | high | 11.00 | 0.004111 |
| IQCJ | 654502 | chr3 | 159107400 | 159113199 | high | 14.50 | 0.004114 |
| IQCJ | 654502 | chr3 | 159241800 | 159247799 | high | 15.00 | 0.004110 |
| IQCJ | 654502 | chr3 | 159165000 | 159175199 | high | 25.50 | 0.004112 |
| IQGAP1 | 8826 | chr15 | 90404000 | 90407799 | high | 9.50 | 0.000098 |
| IQGAP1 | 8826 | chr15 | 90446600 | 90451199 | high | 11.50 | 0.000098 |
| IQSEC1 | 9922 | chr3 | 12894400 | 12900399 | high | 15.00 | 0.000769 |
| IRAK3 | 11213 | chr12 | 66194400 | 66199199 | high | 12.00 | 0.000169 |
| IRAK3 | 11213 | chr12 | 66187600 | 66193199 | high | 14.00 | 0.000169 |
| IRAK3 | 11213 | chr12 | 66200000 | 66207399 | high | 18.50 | 0.000169 |
| IRF2BP2 | 359948 | chr1 | 234602600 | 234606399 | high | 9.50 | 0.001534 |
| IRF2BPL | 64207 | chr14 | 77024600 | 77029399 | high | 12.00 | 0.000834 |
| ISCU | 23479 | chr12 | 108553000 | 108568199 | high | 38.00 | 0.000216 |
| ISG20L2 | 81875 | chr1 | 156724200 | 156729999 | high | 14.50 | 0.000522 |
| ISPD | 729920 | chr7 | 16302600 | 16306399 | high | 9.50 | 0.006532 |
| ISPD | 729920 | chr7 | 16127600 | 16131399 | high | 9.50 | 0.009004 |
| ISPD | 729920 | chr7 | 16364800 | 16369799 | high | 12.50 | 0.004956 |
| ISYNA1 | 51477 | chr19 | 18431600 | 18435799 | high | 10.50 | 0.002793 |
| ITCH | 83737 | chr20 | 34503200 | 34507199 | high | 10.00 | 0.002427 |
| ITFG2 | 55846 | chr12 | 2812200 | 2813199 | low | -4.34 | 0.019858 |
| ITGA1 | 3672 | chr5 | 52947400 | 52949999 | high | 6.50 | 0.000069 |
| ITGA1 | 3672 | chr5 | 52937400 | 52941599 | high | 10.50 | 0.000069 |
| ITGA1 | 3672 | chr5 | 52922600 | 52926999 | high | 11.00 | 0.000069 |
| ITGA1 | 3672 | chr5 | 52875600 | 52881799 | high | 15.50 | 0.000069 |
| ITGA1 | 3672 | chr5 | 52841800 | 52848799 | high | 17.50 | 0.000069 |
| ITGA1 | 3672 | chr5 | 52856400 | 52863999 | high | 19.00 | 0.000069 |
| ITGA10 | 8515 | chr1 | 145895600 | 145910599 | high | 37.50 | 0.000058 |
| ITGA3 | 3675 | chr17 | 50061000 | 50065799 | high | 12.00 | 0.000073 |
| ITGA6 | 3655 | chr2 | 172441800 | 172444199 | high | 6.00 | 0.000021 |
| ITGA6 | 3655 | chr2 | 172445800 | 172448999 | high | 8.00 | 0.000021 |
| ITGA6 | 3655 | chr2 | 172464000 | 172467599 | high | 9.00 | 0.000021 |
| ITGA6 | 3655 | chr2 | 172472400 | 172476599 | high | 10.50 | 0.000021 |
| ITGA6 | 3655 | chr2 | 172425800 | 172430599 | high | 12.00 | 0.000021 |
| ITGAL | 3683 | chr16 | 30502000 | 30506999 | high | 12.50 | 0.000121 |
| ITGB1 | 3688 | chr10 | 32909200 | 32912599 | high | 8.50 | 0.000112 |
| ITGB3 | 3690 | chr17 | 47296600 | 47300599 | high | 10.00 | 0.000078 |
| ITGB3 | 3690 | chr17 | 47289000 | 47293399 | high | 11.00 | 0.000078 |
| ITGB3 | 3690 | chr17 | 47264200 | 47268799 | high | 11.50 | 0.000078 |
| ITGB3 | 3690 | chr17 | 47279600 | 47287799 | high | 20.50 | 0.000078 |
| ITGB4 | 3691 | chr17 | 75751800 | 75754599 | high | 7.00 | 0.000049 |
| ITGB4 | 3691 | chr17 | 75735000 | 75741399 | high | 16.00 | 0.000049 |
| ITGB5 | 3693 | chr3 | 124773200 | 124776599 | high | 8.50 | 0.000030 |
| ITGB5 | 3693 | chr3 | 124810400 | 124814199 | high | 9.50 | 0.000030 |
| ITGB5 | 3693 | chr3 | 124868600 | 124872799 | high | 10.50 | 0.000030 |
| ITIH3 | 3699 | chr3 | 52795200 | 52799799 | high | 11.50 | 0.000070 |
| ITM2B | 9445 | chr13 | 48234400 | 48238399 | low | -17.51 | 0.000196 |
| ITPR2 | 3709 | chr12 | 26581600 | 26585999 | low | -19.57 | 0.000140 |
| ITPR2 | 3709 | chr12 | 26574800 | 26577399 | low | -11.30 | 0.000140 |
| ITPR2 | 3709 | chr12 | 26650200 | 26652199 | low | -8.69 | 0.000139 |
| ITPR2 | 3709 | chr12 | 26487400 | 26488599 | low | -5.21 | 0.000140 |
| ITPR3 | 3710 | chr6 | 33653600 | 33656199 | high | 6.50 | 0.000110 |
| ITPR3 | 3710 | chr6 | 33673800 | 33679799 | high | 15.00 | 0.000110 |
| ITPR3 | 3710 | chr6 | 33619000 | 33626399 | high | 18.50 | 0.000110 |
| ITPRIP | 85450 | chr10 | 104319600 | 104324799 | high | 13.00 | 0.000819 |
| IVD | 3712 | chr15 | 40403800 | 40408399 | high | 11.50 | 0.000092 |
| IWS1 | 55677 | chr2 | 127525400 | 127526599 | low | -5.21 | 0.000437 |
| JADE2 | 23338 | chr5 | 134566000 | 134569999 | high | 10.00 | 0.000173 |
| JADE2 | 23338 | chr5 | 134522200 | 134527999 | high | 14.50 | 0.000173 |
| JAG1 | 182 | chr20 | 10668800 | 10674599 | high | 14.50 | 0.000017 |
| JAG1 | 182 | chr20 | 10660000 | 10667599 | high | 19.00 | 0.000017 |
| JARID2 | 3720 | chr6 | 15284800 | 15287399 | high | 6.50 | 0.000243 |
| JARID2 | 3720 | chr6 | 15278600 | 15281999 | high | 8.50 | 0.000243 |
| JARID2 | 3720 | chr6 | 15428000 | 15431999 | high | 10.00 | 0.000241 |
| JARID2 | 3720 | chr6 | 15244800 | 15249599 | high | 12.00 | 0.000244 |
| JAZF1 | 221895 | chr7 | 28017400 | 28020599 | high | 8.00 | 0.007920 |
| JAZF1 | 221895 | chr7 | 28044800 | 28048799 | high | 10.00 | 0.007912 |
| JAZF1 | 221895 | chr7 | 28054800 | 28059199 | high | 11.00 | 0.007909 |
| JAZF1 | 221895 | chr7 | 28067400 | 28072199 | high | 12.00 | 0.007906 |
| JAZF1 | 221895 | chr7 | 28035600 | 28040799 | high | 13.00 | 0.007915 |
| JDP2 | 122953 | chr14 | 75447400 | 75450999 | high | 9.00 | 0.001630 |
| JDP2 | 122953 | chr14 | 75425800 | 75430199 | high | 11.00 | 0.001630 |
| JDP2 | 122953 | chr14 | 75433600 | 75439199 | high | 14.00 | 0.001630 |

| | | | | | | | |
|---|---|---|---|---|---|---|---|
| JMJD1C | 221037 | chr10 | 63455200 | 63458599 | high | 8.50 | 0.003483 |
| JMJD1C | 221037 | chr10 | 63459400 | 63462999 | high | 9.00 | 0.003483 |
| JMJD1C | 221037 | chr10 | 63478400 | 63482399 | high | 10.00 | 0.003482 |
| JMJD1C | 221037 | chr10 | 63503800 | 63509199 | high | 13.50 | 0.003481 |
| JMJD1C | 221037 | chr10 | 63445400 | 63450799 | high | 13.50 | 0.003484 |
| JRKL | 8690 | chr11 | 96387800 | 96392399 | low | -19.63 | 0.000090 |
| JUN | 3725 | chr1 | 58782000 | 58783999 | low | -8.69 | 0.000063 |
| KANK1 | 23189 | chr9 | 737200 | 742599 | high | 13.50 | 0.003495 |
| KANK2 | 25959 | chr19 | 11166400 | 11168999 | high | 6.50 | 0.002325 |
| KANK2 | 25959 | chr19 | 11185200 | 11190399 | high | 13.00 | 0.002321 |
| KANSL1L | 151050 | chr2 | 210169400 | 210172399 | high | 7.50 | 0.000719 |
| KAT2A | 2648 | chr17 | 42111600 | 42118799 | high | 18.00 | 0.000063 |
| KAT5 | 10524 | chr11 | 65717800 | 65721999 | high | 10.50 | 0.000160 |
| KAT6A | 7994 | chr8 | 41996200 | 41999999 | high | 9.50 | 0.000190 |
| KAT6A | 7994 | chr8 | 41983200 | 41987399 | high | 10.50 | 0.000190 |
| KAT6A | 7994 | chr8 | 41974200 | 41979399 | high | 13.00 | 0.000190 |
| KAT6B | 23522 | chr10 | 74947600 | 74950399 | high | 7.00 | 0.000314 |
| KAT6B | 23522 | chr10 | 74880400 | 74883199 | high | 7.00 | 0.000314 |
| KAT6B | 23522 | chr10 | 74908000 | 74911799 | high | 9.50 | 0.000314 |
| KAT6B | 23522 | chr10 | 74893600 | 74897999 | high | 11.00 | 0.000314 |
| KAT6B | 23522 | chr10 | 74920000 | 74925199 | high | 13.00 | 0.000314 |
| KAT6B | 23522 | chr10 | 74953200 | 74958599 | high | 13.50 | 0.000314 |
| KAT7 | 11143 | chr17 | 49787400 | 49790399 | high | 7.50 | 0.000224 |
| KAT8 | 84148 | chr16 | 31117200 | 31119599 | high | 6.00 | 0.002704 |
| KBTBD6 | 89890 | chr13 | 41127600 | 41131399 | high | 9.50 | 0.002186 |
| KCNAB1 | 7881 | chr3 | 156513200 | 156519199 | high | 15.00 | 0.000050 |
| KCNAB1 | 7881 | chr3 | 156483400 | 156490199 | high | 17.00 | 0.000050 |
| KCNC2 | 3747 | chr12 | 75175600 | 75178999 | high | 8.50 | 0.000050 |
| KCNG1 | 3755 | chr20 | 51019600 | 51023999 | high | 11.00 | 0.000074 |
| KCNH3 | 23416 | chr12 | 49543200 | 49550199 | high | 17.50 | 0.000473 |
| KCNH4 | 23415 | chr17 | 42177600 | 42181799 | high | 10.50 | 0.000555 |
| KCNIP2 | 30819 | chr10 | 101837800 | 101846199 | high | 21.00 | 0.000303 |
| KCNIP3 | 30818 | chr2 | 95326400 | 95334799 | high | 21.00 | 0.000323 |
| KCNIP4 | 80333 | chr4 | 21889400 | 21892999 | low | -15.65 | 0.003670 |
| KCNJ15 | 3772 | chr21 | 38286200 | 38289399 | high | 8.00 | 0.000099 |
| KCNJ15 | 3772 | chr21 | 38258400 | 38262199 | high | 9.50 | 0.000099 |
| KCNJ15 | 3772 | chr21 | 38241000 | 38246799 | high | 14.50 | 0.000099 |
| KCNJ16 | 3773 | chr17 | 70078000 | 70082799 | high | 12.00 | 0.000054 |
| KCNMA1 | 3778 | chr10 | 77313200 | 77314999 | high | 4.50 | 0.000049 |
| KDELR2 | 11014 | chr7 | 6482200 | 6483999 | low | -7.82 | 0.001699 |
| KDM1A | 23028 | chr1 | 23018600 | 23021999 | low | -14.78 | 0.001000 |
| KDM1A | 23028 | chr1 | 23051400 | 23053799 | low | -10.43 | 0.000999 |
| KDM2A | 22992 | chr11 | 67233800 | 67237599 | high | 9.50 | 0.000342 |
| KDM2A | 22992 | chr11 | 67214800 | 67219599 | high | 12.00 | 0.000342 |
| KDM2A | 22992 | chr11 | 67143400 | 67149599 | high | 15.50 | 0.000342 |
| KDM2A | 22992 | chr11 | 67244000 | 67250999 | high | 17.50 | 0.000342 |
| KDM3B | 51780 | chr5 | 138352800 | 138355199 | low | -10.43 | 0.000374 |
| KDM4A | 9682 | chr1 | 43698400 | 43703599 | high | 13.00 | 0.000222 |
| KDM4B | 23030 | chr19 | 4974400 | 4979799 | high | 13.50 | 0.004630 |
| KDM4D | 55693 | chr11 | 94972200 | 94974599 | low | -10.43 | 0.000586 |
| KDM5A | 5927 | chr12 | 386000 | 390199 | high | 10.50 | 0.015355 |
| KDM5C | 8242 | chrX | 53214400 | 53217399 | low | -13.04 | 0.000155 |
| KDM6A | 7403 | chrX | 45094600 | 45100399 | low | -25.13 | 0.000164 |
| KDM6B | 23135 | chr17 | 7854800 | 7860799 | high | 15.00 | 0.002945 |
| KDM8 | 79831 | chr16 | 27202400 | 27206599 | high | 10.50 | 0.002935 |
| KEAP1 | 9817 | chr19 | 10501800 | 10503599 | high | 4.50 | 0.000935 |
| KIF11 | 3832 | chr10 | 92592600 | 92596599 | low | -17.38 | 0.000041 |
| KIF11 | 3832 | chr10 | 92616400 | 92618799 | low | -10.43 | 0.000041 |
| KIF13A | 63971 | chr6 | 17865800 | 17869599 | high | 9.50 | 0.003581 |
| KIF13A | 63971 | chr6 | 17858200 | 17862199 | high | 10.00 | 0.003582 |
| KIF13A | 63971 | chr6 | 17835400 | 17839999 | high | 11.50 | 0.003587 |
| KIF13A | 63971 | chr6 | 17961800 | 17966599 | high | 12.00 | 0.003562 |
| KIF13A | 63971 | chr6 | 17943600 | 17952399 | high | 22.00 | 0.003565 |
| KIF13B | 23303 | chr8 | 29143800 | 29148399 | high | 11.50 | 0.000800 |
| KIF15 | 56992 | chr3 | 44823400 | 44828599 | high | 13.00 | 0.001271 |
| KIF15 | 56992 | chr3 | 44842800 | 44848999 | high | 15.50 | 0.001271 |
| KIF16B | 55614 | chr20 | 16526400 | 16529399 | high | 7.50 | 0.003365 |
| KIF17 | 57576 | chr1 | 20664800 | 20668799 | high | 10.00 | 0.002786 |
| KIF17 | 57576 | chr1 | 20691200 | 20696999 | high | 14.50 | 0.002783 |
| KIF18B | 146909 | chr17 | 44946800 | 44948599 | low | -7.82 | 0.003269 |
| KIF1BP | 26128 | chr10 | 68987600 | 68990599 | high | 7.50 | 0.000379 |
| KIF1BP | 26128 | chr10 | 69008000 | 69012599 | high | 11.50 | 0.000379 |
| KIF20B | 9585 | chr10 | 89766800 | 89770599 | low | -16.53 | 0.000107 |
| KIF20B | 9585 | chr10 | 89732600 | 89735399 | low | -12.17 | 0.000107 |
| KIF20B | 9585 | chr10 | 89718000 | 89720399 | low | -10.43 | 0.000107 |
| KIF24 | 347240 | chr9 | 34314400 | 34317599 | high | 8.00 | 0.010119 |
| KIF24 | 347240 | chr9 | 34286400 | 34290399 | high | 10.00 | 0.010128 |
| KIF3A | 11127 | chr5 | 132725600 | 132730399 | high | 12.00 | 0.000084 |

| | | | | | | | |
|---|---|---|---|---|---|---|---|
| KIF3A | 11127 | chr5 | 132710600 | 132716199 | high | 14.00 | 0.000084 |
| KIF3B | 9371 | chr20 | 32331000 | 32332999 | low | -8.69 | 0.000290 |
| KIF3C | 3797 | chr2 | 25927400 | 25930599 | high | 8.00 | 0.000146 |
| KIF3C | 3797 | chr2 | 25976800 | 25981399 | high | 11.50 | 0.000146 |
| KIF5B | 3799 | chr10 | 32035200 | 32038799 | high | 9.00 | 0.000119 |
| KIF5B | 3799 | chr10 | 32016600 | 32020799 | high | 10.50 | 0.000119 |
| KIF7 | 374654 | chr15 | 89654800 | 89655599 | low | -3.47 | 0.004179 |
| KIFAP3 | 22920 | chr1 | 170034800 | 170039599 | high | 12.00 | 0.000135 |
| KIFAP3 | 22920 | chr1 | 170028600 | 170033399 | high | 12.00 | 0.000135 |
| KIFAP3 | 22920 | chr1 | 169995200 | 170001999 | high | 17.00 | 0.000135 |
| KIFC2 | 90990 | chr8 | 144464400 | 144466599 | high | 5.50 | 0.000630 |
| KIFC3 | 3801 | chr16 | 57796600 | 57799799 | high | 8.00 | 0.000066 |
| KIFC3 | 3801 | chr16 | 57761800 | 57765599 | high | 9.50 | 0.000066 |
| KIRREL | 55243 | chr1 | 157992000 | 157995199 | high | 8.00 | 0.000350 |
| KIRREL | 55243 | chr1 | 158038000 | 158042199 | high | 10.50 | 0.000350 |
| KIRREL | 55243 | chr1 | 158003600 | 158007799 | high | 10.50 | 0.000350 |
| KIRREL | 55243 | chr1 | 158016400 | 158021399 | high | 12.50 | 0.000350 |
| KLB | 152831 | chr4 | 39440600 | 39444599 | high | 10.00 | 0.003875 |
| KLC4 | 89953 | chr6 | 43075000 | 43078399 | high | 8.50 | 0.002088 |
| KLC4 | 89953 | chr6 | 43057000 | 43060399 | high | 8.50 | 0.002089 |
| KLF10 | 7071 | chr8 | 102654400 | 102656399 | low | -8.69 | 0.000069 |
| KLF12 | 11278 | chr13 | 73831800 | 73835399 | high | 9.00 | 0.000153 |
| KLF12 | 11278 | chr13 | 73842600 | 73846799 | high | 10.50 | 0.000153 |
| KLF12 | 11278 | chr13 | 73765200 | 73770199 | high | 12.50 | 0.000153 |
| KLF12 | 11278 | chr13 | 73723600 | 73728999 | high | 13.50 | 0.000153 |
| KLF12 | 11278 | chr13 | 73771000 | 73778399 | high | 18.50 | 0.000153 |
| KLF3 | 51274 | chr4 | 38691200 | 38694399 | low | -13.91 | 0.001325 |
| KLF3 | 51274 | chr4 | 38673000 | 38676199 | low | -13.91 | 0.001326 |
| KLF9 | 687 | chr9 | 70408600 | 70411399 | high | 7.00 | 0.000010 |
| KLF9 | 687 | chr9 | 70390600 | 70395799 | high | 13.00 | 0.000010 |
| KLHDC1 | 122773 | chr14 | 49692400 | 49694199 | low | -7.82 | 0.002471 |
| KLHDC2 | 23588 | chr14 | 49767200 | 49770599 | high | 8.50 | 0.000474 |
| KLHDC3 | 116138 | chr6 | 43020800 | 43023399 | high | 6.50 | 0.002700 |
| KLHDC3 | 116138 | chr6 | 43016200 | 43019999 | high | 9.50 | 0.002700 |
| KLHL10 | 317719 | chr17 | 41846200 | 41850399 | high | 10.50 | 0.007593 |
| KLHL11 | 55175 | chr17 | 41846200 | 41850399 | high | 10.50 | 0.001319 |
| KLHL12 | 59349 | chr1 | 202924400 | 202927399 | high | 7.50 | 0.000292 |
| KLHL18 | 23276 | chr3 | 47321400 | 47324999 | high | 9.00 | 0.000492 |
| KLHL20 | 27252 | chr1 | 173714800 | 173717199 | high | 6.00 | 0.000157 |
| KLHL25 | 64410 | chr15 | 85793400 | 85795399 | high | 5.00 | 0.000751 |
| KLHL26 | 55295 | chr19 | 18656000 | 18663399 | high | 18.50 | 0.002964 |
| KLHL28 | 54813 | chr14 | 44959000 | 44962199 | high | 8.00 | 0.001219 |
| KLHL28 | 54813 | chr14 | 44931000 | 44935799 | high | 12.00 | 0.001220 |
| KLHL30 | 377007 | chr2 | 238142000 | 238143999 | high | 5.00 | 0.001583 |
| KLHL38 | 340359 | chr8 | 123645600 | 123649799 | high | 10.50 | 0.002753 |
| KLHL41 | 10324 | chr2 | 169509600 | 169511599 | low | -8.69 | 0.000061 |
| KLHL8 | 57563 | chr4 | 87219000 | 87222199 | low | -13.91 | 0.000660 |
| KLHL8 | 57563 | chr4 | 87194000 | 87196599 | low | -11.30 | 0.000660 |
| KLRA1P | 10748 | chr12 | 10597200 | 10600399 | low | -13.91 | 0.001014 |
| KLRA1P | 10748 | chr12 | 10594200 | 10595799 | low | -6.95 | 0.001015 |
| KLRC4 | 8302 | chr12 | 10404200 | 10409799 | high | 14.00 | 0.000798 |
| KLRF1 | 51348 | chr12 | 9836200 | 9841599 | high | 13.50 | 0.005220 |
| KMT2A | 4297 | chr11 | 118435400 | 118438399 | high | 7.50 | 0.000036 |
| KMT2A | 4297 | chr11 | 118476400 | 118482799 | high | 16.00 | 0.000036 |
| KMT2B | 9757 | chr19 | 35713600 | 35719799 | high | 15.50 | 0.000273 |
| KMT2D | 8085 | chr12 | 49012800 | 49022199 | high | 23.50 | 0.000165 |
| KMT5A | 387893 | chr12 | 123388000 | 123389599 | high | 4.00 | 0.003144 |
| KMT5B | 51111 | chr11 | 68205800 | 68209999 | high | 10.50 | 0.000749 |
| KMT5B | 51111 | chr11 | 68192200 | 68196999 | high | 12.00 | 0.000750 |
| KMT5C | 84787 | chr19 | 55340800 | 55345199 | high | 11.00 | 0.001532 |
| KNL1 | 57082 | chr15 | 40619800 | 40626999 | high | 18.00 | 0.001405 |
| KPNA1 | 3836 | chr3 | 122488200 | 122491399 | high | 8.00 | 0.000031 |
| KPNA1 | 3836 | chr3 | 122433800 | 122437999 | high | 10.50 | 0.000031 |
| KPNA1 | 3836 | chr3 | 122510600 | 122515999 | high | 13.50 | 0.000031 |
| KPNA1 | 3836 | chr3 | 122496600 | 122505399 | high | 22.00 | 0.000031 |
| KPNA2 | 3838 | chr17 | 68034000 | 68038599 | low | -19.53 | 0.000056 |
| KPNA5 | 3841 | chr6 | 116713000 | 116716399 | low | -14.78 | 0.000033 |
| KPNB1 | 3837 | chr17 | 47648800 | 47650999 | low | -9.56 | 0.000081 |
| KRBOX4 | 55634 | chrX | 46458200 | 46459599 | low | -6.08 | 0.001198 |
| KRCC1 | 51315 | chr2 | 88053600 | 88055999 | low | -10.43 | 0.000583 |
| KREMEN2 | 79412 | chr16 | 2967800 | 2970799 | high | 7.50 | 0.002973 |
| KRIT1 | 889 | chr7 | 92223200 | 92224199 | low | -4.34 | 0.000010 |
| KRR1 | 11103 | chr12 | 75510600 | 75511799 | low | -5.21 | 0.000147 |
| KRT10 | 3858 | chr17 | 40815600 | 40822199 | high | 16.50 | 0.000095 |
| KRT7 | 3855 | chr12 | 52230600 | 52236199 | high | 14.00 | 0.000074 |
| KRT80 | 144501 | chr12 | 52179600 | 52190599 | high | 27.50 | 0.002769 |
| KXD1 | 79036 | chr19 | 18557400 | 18564199 | high | 17.00 | 0.004259 |
| KYAT1 | 883 | chr9 | 128851000 | 128855399 | high | 11.00 | 0.000007 |

| | | | | | | | |
|---|---|---|---|---|---|---|---|
| KYNU | 8942 | chr2 | 142981400 | 142985199 | low | -16.47 | 0.000063 |
| KYNU | 8942 | chr2 | 142962200 | 142964799 | low | -11.30 | 0.000063 |
| KYNU | 8942 | chr2 | 142919400 | 142921999 | low | -11.30 | 0.000063 |
| LACC1 | 144811 | chr13 | 43887800 | 43891799 | low | -17.69 | 0.003300 |
| LACTB | 114294 | chr15 | 63136200 | 63138999 | high | 7.00 | 0.001810 |
| LACTB2 | 51110 | chr8 | 70632800 | 70637599 | high | 12.00 | 0.000724 |
| LAG3 | 3902 | chr12 | 6770800 | 6775799 | high | 12.50 | 0.000576 |
| LARP1 | 23367 | chr5 | 154751400 | 154754999 | high | 9.00 | 0.000151 |
| LARP1 | 23367 | chr5 | 154755800 | 154759599 | high | 9.50 | 0.000151 |
| LARP4B | 23185 | chr10 | 844000 | 848199 | high | 10.50 | 0.003052 |
| LARP4B | 23185 | chr10 | 831400 | 835999 | high | 11.50 | 0.003099 |
| LARP4B | 23185 | chr10 | 817600 | 822999 | high | 13.50 | 0.003151 |
| LARS | 51520 | chr5 | 146180200 | 146184599 | high | 11.00 | 0.000352 |
| LASP1 | 3927 | chr17 | 38909000 | 38913399 | high | 11.00 | 0.000101 |
| LASP1 | 3927 | chr17 | 38877400 | 38883999 | high | 16.50 | 0.000101 |
| LATS1 | 9113 | chr6 | 149716000 | 149718599 | low | -11.30 | 0.000061 |
| LATS2 | 26524 | chr13 | 21015000 | 21020599 | high | 14.00 | 0.001262 |
| LATS2 | 26524 | chr13 | 21021600 | 21027599 | high | 15.00 | 0.001262 |
| LCMT2 | 9836 | chr15 | 43329000 | 43332599 | low | -15.65 | 0.000227 |
| LCOR | 84458 | chr10 | 96950200 | 96953999 | high | 9.50 | 0.000871 |
| LCOR | 84458 | chr10 | 96905000 | 96910799 | high | 14.50 | 0.000872 |
| LCORL | 254251 | chr4 | 18019400 | 18022399 | high | 7.50 | 0.014110 |
| LDB1 | 8861 | chr10 | 102118000 | 102122599 | high | 11.50 | 0.000087 |
| LDB1 | 8861 | chr10 | 102107600 | 102117199 | high | 24.00 | 0.000087 |
| LDB3 | 11155 | chr10 | 86709200 | 86713399 | high | 10.50 | 0.000129 |
| LDHB | 3945 | chr12 | 21653600 | 21655799 | high | 5.50 | 0.000182 |
| LEKR1 | 389170 | chr3 | 156825600 | 156830799 | high | 13.00 | 0.002482 |
| LEMD3 | 23592 | chr12 | 65178400 | 65179999 | low | -6.95 | 0.000362 |
| LEMD3 | 23592 | chr12 | 65234000 | 65235399 | low | -6.08 | 0.000362 |
| LENEP | 55891 | chr1 | 154989400 | 154993599 | high | 10.50 | 0.000361 |
| LEPR | 3953 | chr1 | 65576400 | 65579199 | high | 7.00 | 0.000060 |
| LEPR | 3953 | chr1 | 65508200 | 65511399 | high | 8.00 | 0.000060 |
| LETM1 | 3954 | chr4 | 1849000 | 1852599 | high | 9.00 | 0.002138 |
| LFNG | 3955 | chr7 | 2516000 | 2520399 | high | 11.00 | 0.001572 |
| LGI3 | 203190 | chr8 | 22145000 | 22150199 | high | 13.00 | 0.009175 |
| LHFP | 10186 | chr13 | 39576400 | 39579199 | low | -12.17 | 0.000257 |
| LHX9 | 56956 | chr1 | 197922200 | 197923999 | low | -7.82 | 0.000288 |
| LIF | 3976 | chr22 | 30245600 | 30247799 | low | -9.56 | 0.000131 |
| LIG1 | 3978 | chr19 | 48167200 | 48174399 | high | 18.00 | 0.000083 |
| LIG3 | 3980 | chr17 | 35003600 | 35005999 | high | 6.00 | 0.000114 |
| LIG3 | 3980 | chr17 | 34978400 | 34984999 | high | 16.50 | 0.000114 |
| LIG4 | 3981 | chr13 | 108218000 | 108219399 | high | 3.50 | 0.000037 |
| LIMD1 | 8994 | chr3 | 45644400 | 45649199 | high | 12.00 | 0.000197 |
| LIMD2 | 80774 | chr17 | 63699800 | 63701199 | high | 3.50 | 0.001268 |
| LIMK1 | 3984 | chr7 | 74081600 | 74087199 | high | 14.00 | 0.000054 |
| LIMK1 | 3984 | chr7 | 74092000 | 74100599 | high | 21.50 | 0.000054 |
| LIMK2 | 3985 | chr22 | 31211800 | 31213199 | low | -6.08 | 0.000128 |
| LIMS1 | 3987 | chr2 | 108567000 | 108571199 | high | 10.50 | 0.000037 |
| LIMS1 | 3987 | chr2 | 108547000 | 108552399 | high | 13.50 | 0.000037 |
| LIN52 | 91750 | chr14 | 74083000 | 74087199 | high | 10.50 | 0.001238 |
| LIN54 | 132660 | chr4 | 83006200 | 83011399 | high | 13.00 | 0.001598 |
| LIN7A | 8825 | chr12 | 80848000 | 80850999 | high | 7.50 | 0.000109 |
| LIN7A | 8825 | chr12 | 80835200 | 80838399 | high | 8.00 | 0.000109 |
| LIN7A | 8825 | chr12 | 80829200 | 80833999 | high | 12.00 | 0.000109 |
| LIN7C | 55327 | chr11 | 27505400 | 27507399 | low | -8.69 | 0.002011 |
| LIN9 | 286826 | chr1 | 226303000 | 226305399 | low | -10.43 | 0.001267 |
| LMF2 | 91289 | chr22 | 50507000 | 50512399 | high | 13.50 | 0.001807 |
| LMTK3 | 114783 | chr19 | 48487200 | 48490199 | high | 7.50 | 0.002367 |
| LMTK3 | 114783 | chr19 | 48491200 | 48494399 | high | 8.00 | 0.002367 |
| LNX2 | 222484 | chr13 | 27619000 | 27622599 | low | -15.65 | 0.008055 |
| LONP1 | 9361 | chr19 | 5689800 | 5691999 | high | 5.50 | 0.001645 |
| LONP2 | 83752 | chr16 | 48242800 | 48247799 | high | 12.50 | 0.001736 |
| LONRF1 | 91694 | chr8 | 12753000 | 12756599 | high | 9.00 | 0.007190 |
| LPGAT1 | 9926 | chr1 | 211829200 | 211830799 | low | -6.95 | 0.000047 |
| LPIN1 | 23175 | chr2 | 11780400 | 11782799 | high | 6.00 | 0.001967 |
| LPIN1 | 23175 | chr2 | 11692600 | 11699399 | high | 17.00 | 0.001982 |
| LPIN2 | 9663 | chr18 | 2927400 | 2930999 | high | 9.00 | 0.003301 |
| LPIN2 | 9663 | chr18 | 2954600 | 2960199 | high | 14.00 | 0.003270 |
| LPIN2 | 9663 | chr18 | 2975800 | 2981599 | high | 14.50 | 0.003247 |
| LPIN2 | 9663 | chr18 | 2991000 | 2998399 | high | 18.50 | 0.003231 |
| LPP | 4026 | chr3 | 188416400 | 188417399 | high | 2.50 | 0.000021 |
| LPP | 4026 | chr3 | 188433800 | 188436199 | high | 6.00 | 0.000021 |
| LPP | 4026 | chr3 | 188572200 | 188574999 | high | 7.00 | 0.000021 |
| LPP | 4026 | chr3 | 188558800 | 188562199 | high | 8.50 | 0.000021 |
| LPP | 4026 | chr3 | 188437600 | 188440999 | high | 8.50 | 0.000021 |
| LPP | 4026 | chr3 | 188459600 | 188463399 | high | 9.50 | 0.000021 |
| LPP | 4026 | chr3 | 188465800 | 188469799 | high | 10.00 | 0.000021 |
| LPP | 4026 | chr3 | 188545200 | 188549799 | high | 11.50 | 0.000021 |

| | | | | | | | |
|---|---|---|---|---|---|---|---|
| LPP | 4026 | chr3 | 188479400 | 188484999 | high | 14.00 | 0.000021 |
| LPP | 4026 | chr3 | 188448600 | 188455599 | high | 17.50 | 0.000021 |
| LPXN | 9404 | chr11 | 58574600 | 58580599 | high | 15.00 | 0.000161 |
| LRCH1 | 23143 | chr13 | 46552000 | 46555199 | high | 8.00 | 0.000497 |
| LRCH1 | 23143 | chr13 | 46558400 | 46563199 | high | 12.00 | 0.000497 |
| LRCH1 | 23143 | chr13 | 46737000 | 46741999 | high | 12.50 | 0.000495 |
| LRCH3 | 84859 | chr3 | 197867400 | 197869199 | low | -7.82 | 0.000429 |
| LRFN4 | 78999 | chr11 | 66856200 | 66857999 | high | 4.50 | 0.001182 |
| LRP1 | 4035 | chr12 | 57151000 | 57153199 | high | 5.50 | 0.000071 |
| LRP1 | 4035 | chr12 | 57135000 | 57140999 | high | 15.00 | 0.000071 |
| LRP10 | 26020 | chr14 | 22871400 | 22872599 | low | -5.21 | 0.001138 |
| LRP12 | 29967 | chr8 | 104515600 | 104518399 | low | -12.17 | 0.000287 |
| LRP12 | 29967 | chr8 | 104529000 | 104531599 | low | -11.30 | 0.000287 |
| LRP12 | 29967 | chr8 | 104588000 | 104589999 | low | -8.69 | 0.000287 |
| LRP2 | 4036 | chr2 | 169360000 | 169361599 | low | -6.95 | 0.000024 |
| LRP5 | 4041 | chr11 | 68357400 | 68362399 | high | 12.50 | 0.000059 |
| LRP6 | 4040 | chr12 | 12264400 | 12267399 | low | -13.04 | 0.000329 |
| LRP8 | 7804 | chr1 | 53326400 | 53330199 | high | 9.50 | 0.000146 |
| LRWD1 | 222229 | chr7 | 102470200 | 102473799 | high | 9.00 | 0.002169 |
| LSM1 | 27257 | chr8 | 38164600 | 38171199 | high | 16.50 | 0.000714 |
| LSM14B | 149986 | chr20 | 62121600 | 62124399 | high | 7.00 | 0.002414 |
| LSM3 | 27258 | chr3 | 14178200 | 14180199 | low | -8.69 | 0.001923 |
| LSM4 | 25804 | chr19 | 18321400 | 18324199 | high | 7.00 | 0.001408 |
| LSM4 | 25804 | chr19 | 18305600 | 18310199 | high | 11.50 | 0.001410 |
| LSR | 51599 | chr19 | 35265200 | 35270399 | high | 13.00 | 0.001463 |
| LSS | 4047 | chr21 | 46227000 | 46233599 | high | 16.50 | 0.000088 |
| LTBP1 | 4052 | chr2 | 33389200 | 33392199 | high | 7.50 | 0.000121 |
| LTBP1 | 4052 | chr2 | 33256000 | 33259799 | high | 9.50 | 0.000122 |
| LTBP1 | 4052 | chr2 | 33354800 | 33359799 | high | 12.50 | 0.000121 |
| LTBP1 | 4052 | chr2 | 33375800 | 33381199 | high | 13.50 | 0.000121 |
| LTBP2 | 4053 | chr14 | 74594400 | 74597599 | high | 8.00 | 0.000054 |
| LTBP2 | 4053 | chr14 | 74598400 | 74602999 | high | 11.50 | 0.000054 |
| LTBP2 | 4053 | chr14 | 74537600 | 74543399 | high | 14.50 | 0.000054 |
| LTBP3 | 4054 | chr11 | 65536400 | 65542199 | high | 14.50 | 0.000062 |
| LTBP4 | 8425 | chr19 | 40612600 | 40614999 | high | 6.00 | 0.000207 |
| LTBP4 | 8425 | chr19 | 40617800 | 40623799 | high | 15.00 | 0.000207 |
| LTBP4 | 8425 | chr19 | 40601800 | 40608599 | high | 17.00 | 0.000208 |
| LTBP4 | 8425 | chr19 | 40593400 | 40600399 | high | 17.50 | 0.000208 |
| LTBR | 4055 | chr12 | 6374200 | 6376399 | high | 5.50 | 0.000636 |
| LTBR | 4055 | chr12 | 6381600 | 6386399 | high | 12.00 | 0.000635 |
| LTC4S | 4056 | chr5 | 179794400 | 179797999 | high | 9.00 | 0.000023 |
| LTN1 | 26046 | chr21 | 28965800 | 28969599 | high | 9.50 | 0.000899 |
| LTN1 | 26046 | chr21 | 28970800 | 28976199 | high | 13.50 | 0.000899 |
| LUC7L3 | 51747 | chr17 | 50719400 | 50723599 | high | 10.50 | 0.001020 |
| LUM | 4060 | chr12 | 91104800 | 91108799 | low | -17.52 | 0.000045 |
| LY75 | 4065 | chr2 | 159895200 | 159900399 | low | -22.47 | 0.000025 |
| LYAR | 55646 | chr4 | 4288800 | 4292599 | high | 9.50 | 0.012975 |
| LYAR | 55646 | chr4 | 4265800 | 4271599 | high | 14.50 | 0.013045 |
| LYPLA1 | 10434 | chr8 | 54100200 | 54102999 | high | 7.00 | 0.000193 |
| LYRM1 | 57149 | chr16 | 20910200 | 20913599 | high | 8.50 | 0.002733 |
| LYRM1 | 57149 | chr16 | 20898000 | 20902799 | high | 12.00 | 0.002735 |
| LYRM4 | 57128 | chr6 | 5188600 | 5191399 | low | -12.17 | 0.011010 |
| LYSMD2 | 256586 | chr15 | 51723800 | 51729199 | high | 13.50 | 0.004961 |
| LYSMD2 | 256586 | chr15 | 51733800 | 51739999 | high | 15.50 | 0.004960 |
| LYSMD3 | 116068 | chr5 | 90526600 | 90530199 | high | 9.00 | 0.001282 |
| LYST | 1130 | chr1 | 235842000 | 235846599 | high | 11.50 | 0.000005 |
| LYST | 1130 | chr1 | 235873400 | 235878399 | high | 12.50 | 0.000005 |
| LYVE1 | 10894 | chr11 | 10568000 | 10570399 | high | 6.00 | 0.001031 |
| LZIC | 84328 | chr1 | 9942000 | 9945999 | high | 10.00 | 0.008482 |
| LZTFL1 | 54585 | chr3 | 45836800 | 45838599 | low | -7.82 | 0.001191 |
| LZTR1 | 8216 | chr22 | 20980000 | 20982599 | low | -11.30 | 0.000392 |
| M1AP | 130951 | chr2 | 74575400 | 74581799 | high | 16.00 | 0.001756 |
| MACF1 | 23499 | chr1 | 39309400 | 39311799 | high | 6.00 | 0.000598 |
| MACF1 | 23499 | chr1 | 39111400 | 39114399 | high | 7.50 | 0.000601 |
| MACF1 | 23499 | chr1 | 39103200 | 39106999 | high | 9.50 | 0.000601 |
| MACF1 | 23499 | chr1 | 39260400 | 39264399 | high | 10.00 | 0.000599 |
| MACF1 | 23499 | chr1 | 39457200 | 39461399 | high | 10.50 | 0.000596 |
| MACF1 | 23499 | chr1 | 39274400 | 39278599 | high | 10.50 | 0.000598 |
| MACF1 | 23499 | chr1 | 39266800 | 39271599 | high | 12.00 | 0.000598 |
| MACF1 | 23499 | chr1 | 39203000 | 39207799 | high | 12.00 | 0.000599 |
| MACF1 | 23499 | chr1 | 39176800 | 39182399 | high | 14.00 | 0.000600 |
| MACF1 | 23499 | chr1 | 39085200 | 39091999 | high | 17.00 | 0.000601 |
| MAD2L1 | 4085 | chr4 | 120064800 | 120068199 | high | 8.50 | 0.000034 |
| MADD | 8567 | chr11 | 47263800 | 47273799 | high | 25.00 | 0.000181 |
| MAEA | 10296 | chr4 | 1289000 | 1291399 | high | 6.00 | 0.007988 |
| MAEA | 10296 | chr4 | 1300000 | 1304199 | high | 10.50 | 0.007920 |
| MAF1 | 84232 | chr8 | 144102000 | 144104799 | high | 7.00 | 0.000585 |
| MAFK | 7975 | chr7 | 1527800 | 1535799 | high | 20.00 | 0.005220 |

| | | | | | | | |
|---|---|---|---|---|---|---|---|
| MAGI1 | 9223 | chr3 | 65898000 | 65902399 | high | 11.00 | 0.000140 |
| MAGI2 | 9863 | chr7 | 79177800 | 79181399 | low | -15.65 | 0.000125 |
| MAGI2 | 9863 | chr7 | 78366600 | 78369999 | low | -14.78 | 0.000126 |
| MAGI2 | 9863 | chr7 | 78708600 | 78711599 | low | -13.04 | 0.000125 |
| MAGI2 | 9863 | chr7 | 79169800 | 79172199 | low | -10.43 | 0.000125 |
| MAGI2 | 9863 | chr7 | 79152400 | 79154399 | low | -8.69 | 0.000125 |
| MAGI2 | 9863 | chr7 | 79186200 | 79187999 | low | -7.82 | 0.000125 |
| MAGI2 | 9863 | chr7 | 78399000 | 78400599 | low | -6.95 | 0.000126 |
| MAGI3 | 260425 | chr1 | 113416600 | 113419599 | high | 7.50 | 0.002296 |
| MAK16 | 84549 | chr8 | 33485200 | 33487799 | low | -11.30 | 0.002525 |
| MALAT1 | 378938 | chr11 | 65498800 | 65505399 | low | -28.47 | 0.005785 |
| MALSU1 | 115416 | chr7 | 23297600 | 23314799 | high | 43.00 | 0.004954 |
| MALT1 | 10892 | chr18 | 58684800 | 58688799 | high | 10.00 | 0.000186 |
| MAML1 | 9794 | chr5 | 179732000 | 179734599 | low | -11.30 | 0.000054 |
| MAP2K1 | 5604 | chr15 | 66386000 | 66387999 | low | -17.99 | 0.000264 |
| MAP2K2 | 5605 | chr19 | 4122800 | 4124599 | low | -7.82 | 0.001360 |
| MAP2K3 | 5606 | chr17 | 21290400 | 21293999 | low | -9.00 | 0.000263 |
| MAP3K2 | 10746 | chr2 | 127337200 | 127339599 | low | -6.00 | 0.000084 |
| MAP3K3 | 4215 | chr17 | 63622600 | 63626799 | low | -10.50 | 0.000066 |
| MAP3K4 | 4216 | chr6 | 161115000 | 161118399 | low | -14.78 | 0.000026 |
| MAP3K4 | 4216 | chr6 | 160991200 | 160993399 | low | -9.56 | 0.000026 |
| MAP3K5 | 4217 | chr6 | 136752000 | 136753999 | high | 5.00 | 0.000031 |
| MAP3K5 | 4217 | chr6 | 136743800 | 136748199 | high | 11.00 | 0.000031 |
| MAP3K5 | 4217 | chr6 | 136723800 | 136729599 | high | 14.50 | 0.000031 |
| MAP3K5 | 4217 | chr6 | 136710000 | 136718199 | high | 20.50 | 0.000031 |
| MAP3K6 | 9064 | chr1 | 27364800 | 27367399 | high | 6.50 | 0.000331 |
| MAP3K6 | 9064 | chr1 | 27363800 | 27367799 | high | 10.00 | 0.000331 |
| MAP4 | 4134 | chr3 | 47866200 | 47869199 | high | 7.50 | 0.000086 |
| MAP4 | 4134 | chr3 | 47917800 | 47921599 | high | 9.50 | 0.000086 |
| MAP4 | 4134 | chr3 | 47987000 | 47990999 | high | 10.00 | 0.000086 |
| MAP4 | 4134 | chr3 | 48046600 | 48051199 | high | 11.50 | 0.000086 |
| MAP4 | 4134 | chr3 | 48037000 | 48042399 | high | 13.50 | 0.000086 |
| MAP4 | 4134 | chr3 | 48066400 | 48071999 | high | 14.00 | 0.000086 |
| MAP6 | 4135 | chr11 | 75601200 | 75605399 | high | 10.50 | 0.000055 |
| MAP7 | 9053 | chr6 | 136368000 | 136371799 | low | -16.78 | 0.000066 |
| MAPK1 | 5594 | chr22 | 21788000 | 21791599 | high | 9.00 | 0.000257 |
| MAPK1 | 5594 | chr22 | 21798800 | 21802799 | high | 10.00 | 0.000257 |
| MAPK14 | 1432 | chr6 | 36051800 | 36057399 | high | 14.00 | 0.000040 |
| MAPK14 | 1432 | chr6 | 36060000 | 36066399 | high | 16.00 | 0.000040 |
| MAPK3 | 5595 | chr16 | 30117800 | 30119799 | low | -8.69 | 0.000186 |
| MAPK7 | 5598 | chr17 | 19376200 | 19380199 | high | 10.00 | 0.000289 |
| MAPK8 | 5599 | chr10 | 48436400 | 48440599 | high | 10.50 | 0.000116 |
| MAPK8 | 5599 | chr10 | 48429200 | 48433399 | high | 10.50 | 0.000116 |
| MAST4 | 375449 | chr5 | 67058200 | 67061799 | high | 9.00 | 0.005599 |
| MAST4 | 375449 | chr5 | 67037000 | 67040799 | high | 9.50 | 0.005601 |
| MAST4 | 375449 | chr5 | 67044200 | 67048799 | high | 11.50 | 0.005600 |
| MAST4 | 375449 | chr5 | 67154400 | 67159399 | high | 12.50 | 0.005591 |
| MAST4 | 375449 | chr5 | 67135600 | 67141199 | high | 14.00 | 0.005592 |
| MAST4 | 375449 | chr5 | 67008600 | 67014999 | high | 16.00 | 0.005603 |
| MAST4 | 375449 | chr5 | 66987800 | 66994199 | high | 16.00 | 0.005605 |
| MAT2B | 27430 | chr5 | 163503000 | 163506999 | high | 10.00 | 0.000168 |
| MATN2 | 4147 | chr8 | 98008000 | 98009999 | high | 5.00 | 0.000042 |
| MATR3 | 9782 | chr5 | 139285000 | 139288399 | high | 8.50 | 0.000070 |
| MAU2 | 23383 | chr19 | 19319800 | 19323599 | high | 9.50 | 0.001210 |
| MAVS | 57506 | chr20 | 3862200 | 3867199 | high | 12.50 | 0.014889 |
| MB21D2 | 151963 | chr3 | 192916600 | 192918599 | high | 5.00 | 0.000788 |
| MBD2 | 8932 | chr18 | 54221800 | 54224999 | low | -13.91 | 0.000165 |
| MBLAC1 | 255374 | chr7 | 100126200 | 100127599 | low | -6.08 | 0.002551 |
| MBNL1 | 4154 | chr3 | 152321400 | 152324799 | high | 8.50 | 0.000027 |
| MBNL1 | 4154 | chr3 | 152316200 | 152320199 | high | 10.00 | 0.000027 |
| MBNL2 | 10150 | chr13 | 97314000 | 97317399 | low | -14.78 | 0.000104 |
| MBOAT2 | 129642 | chr2 | 8977400 | 8979199 | low | -7.82 | 0.014441 |
| MBOAT2 | 129642 | chr2 | 9003000 | 9004399 | low | -6.08 | 0.014400 |
| MBP | 4155 | chr18 | 77064800 | 77068199 | high | 8.50 | 0.000054 |
| MCC | 4163 | chr5 | 113025600 | 113029599 | high | 10.00 | 0.000037 |
| MCC | 4163 | chr5 | 113035200 | 113039799 | high | 11.50 | 0.000037 |
| MCC | 4163 | chr5 | 113485200 | 113491799 | high | 16.50 | 0.000037 |
| MCC | 4163 | chr5 | 113049800 | 113058199 | high | 21.00 | 0.000037 |
| MCEE | 84693 | chr2 | 71128200 | 71130999 | high | 7.00 | 0.001191 |
| MCF2L2 | 23101 | chr3 | 183368000 | 183371799 | high | 9.50 | 0.000126 |
| MCF2L2 | 23101 | chr3 | 183263200 | 183267799 | high | 11.50 | 0.000126 |
| MCF2L2 | 23101 | chr3 | 183181400 | 183185999 | high | 11.50 | 0.000126 |
| MCM3 | 4172 | chr6 | 52270600 | 52273199 | low | -11.30 | 0.000080 |
| MCM4 | 4173 | chr8 | 47957400 | 47961799 | high | 11.00 | 0.000087 |
| MCM7 | 4176 | chr7 | 100099200 | 100104199 | high | 12.50 | 0.000042 |
| MCM8 | 84515 | chr20 | 5969800 | 5973199 | high | 8.50 | 0.014157 |
| MCM9 | 254394 | chr6 | 118907600 | 118909799 | low | -9.56 | 0.002139 |
| MCMBP | 79892 | chr10 | 119867600 | 119869199 | high | 4.00 | 0.000667 |

| | | | | | | | |
|---|---|---|---|---|---|---|---|
| MCOLN1 | 57192 | chr19 | 7533000 | 7536799 | high | 9.50 | 0.007592 |
| MCPH1 | 79648 | chr8 | 6643600 | 6645399 | low | -7.82 | 0.011989 |
| MCPH1 | 79648 | chr8 | 6604800 | 6605799 | low | -4.34 | 0.012059 |
| MCTP1 | 79772 | chr5 | 94899200 | 94901799 | low | -11.30 | 0.000841 |
| MCUB | 55013 | chr4 | 109559000 | 109561399 | high | 6.00 | 0.000502 |
| MDGA1 | 266727 | chr6 | 37694800 | 37699199 | high | 11.00 | 0.007076 |
| MDM1 | 56890 | chr12 | 68330400 | 68332999 | high | 6.50 | 0.000833 |
| MDM2 | 4193 | chr12 | 68808400 | 68809399 | low | -4.34 | 0.000061 |
| MDM4 | 4194 | chr1 | 204516400 | 204519599 | high | 8.00 | 0.000021 |
| MDN1 | 23195 | chr6 | 89679200 | 89682399 | high | 8.00 | 0.000259 |
| MDP1 | 145553 | chr14 | 24211200 | 24216199 | high | 12.50 | 0.006012 |
| ME3 | 10873 | chr11 | 86628800 | 86631799 | high | 7.50 | 0.000126 |
| ME3 | 10873 | chr11 | 86558000 | 86561399 | high | 8.50 | 0.000126 |
| ME3 | 10873 | chr11 | 86567600 | 86571599 | high | 10.00 | 0.000126 |
| ME3 | 10873 | chr11 | 86562600 | 86566799 | high | 10.50 | 0.000126 |
| ME3 | 10873 | chr11 | 86467800 | 86473199 | high | 13.50 | 0.000126 |
| ME3 | 10873 | chr11 | 86618400 | 86624799 | high | 16.00 | 0.000126 |
| MEAF6 | 64769 | chr1 | 37512800 | 37515199 | low | -10.43 | 0.001727 |
| MED1 | 5469 | chr17 | 39403600 | 39406799 | high | 8.00 | 0.000139 |
| MED13 | 9969 | chr17 | 61951200 | 61954399 | high | 8.00 | 0.000161 |
| MED13 | 9969 | chr17 | 61940800 | 61946199 | high | 13.50 | 0.000161 |
| MED13L | 23389 | chr12 | 116030200 | 116034599 | high | 11.00 | 0.000202 |
| MED13L | 23389 | chr12 | 116064800 | 116071199 | high | 16.00 | 0.000202 |
| MED13L | 23389 | chr12 | 115984000 | 115992999 | high | 22.50 | 0.000202 |
| MED14 | 9282 | chrX | 40732600 | 40736199 | high | 9.00 | 0.000228 |
| MED15 | 51586 | chr22 | 20552200 | 20555199 | high | 7.50 | 0.002510 |
| MED15 | 51586 | chr22 | 20509000 | 20512999 | high | 10.00 | 0.002515 |
| MED15 | 51586 | chr22 | 20529800 | 20533999 | high | 10.50 | 0.002513 |
| MED15 | 51586 | chr22 | 20523200 | 20527599 | high | 11.00 | 0.002514 |
| MED16 | 10025 | chr19 | 888400 | 894199 | high | 14.50 | 0.011284 |
| MED18 | 54797 | chr1 | 28330400 | 28335799 | high | 13.50 | 0.001934 |
| MED24 | 9862 | chr17 | 40021600 | 40025999 | high | 11.00 | 0.000246 |
| MED25 | 81857 | chr19 | 49816400 | 49820999 | high | 11.50 | 0.001643 |
| MED26 | 9441 | chr19 | 16580600 | 16584399 | high | 9.50 | 0.000569 |
| MED27 | 9442 | chr9 | 132030200 | 132034199 | high | 10.00 | 0.000072 |
| MED27 | 9442 | chr9 | 132045200 | 132050399 | high | 13.00 | 0.000072 |
| MED28 | 80306 | chr4 | 17614400 | 17615399 | high | 2.50 | 0.004559 |
| MED31 | 51003 | chr17 | 6650000 | 6653199 | high | 8.00 | 0.007670 |
| MED8 | 112950 | chr1 | 43389400 | 43393599 | high | 10.50 | 0.002603 |
| MED8 | 112950 | chr1 | 43382000 | 43387199 | high | 13.00 | 0.002604 |
| MED9 | 55090 | chr17 | 17477000 | 17479599 | high | 6.50 | 0.003152 |
| MED9 | 55090 | chr17 | 17481000 | 17484399 | high | 8.50 | 0.003151 |
| MEF2C | 4208 | chr5 | 88723000 | 88725799 | high | 7.00 | 0.000047 |
| MEF2D | 4209 | chr1 | 156493600 | 156496599 | high | 7.50 | 0.000027 |
| MEF2D | 4209 | chr1 | 156481800 | 156489999 | high | 20.50 | 0.000027 |
| MEGF10 | 84466 | chr5 | 127366800 | 127370999 | low | -18.35 | 0.000663 |
| MEGF9 | 1955 | chr9 | 120644200 | 120649399 | low | -22.43 | 0.000016 |
| MEGF9 | 1955 | chr9 | 120711800 | 120715599 | low | -16.58 | 0.000016 |
| MEGF9 | 1955 | chr9 | 120696600 | 120698799 | low | -9.56 | 0.000016 |
| MEI1 | 150365 | chr22 | 41694800 | 41699999 | high | 13.00 | 0.003606 |
| MEIS3 | 56917 | chr19 | 47416200 | 47420199 | high | 10.00 | 0.001200 |
| MEOX1 | 4222 | chr17 | 43647400 | 43650199 | high | 7.00 | 0.000097 |
| MEOX1 | 4222 | chr17 | 43656600 | 43660199 | high | 9.00 | 0.000097 |
| MEOX2 | 4223 | chr7 | 15669400 | 15672199 | low | -12.17 | 0.000270 |
| MESDC2 | 23184 | chr15 | 80988400 | 80990999 | low | -11.30 | 0.000286 |
| METAP1D | 254042 | chr2 | 172037600 | 172039599 | low | -8.69 | 0.001477 |
| METAP2 | 10988 | chr12 | 95472600 | 95476999 | high | 11.00 | 0.000115 |
| METRN | 79006 | chr16 | 714600 | 718199 | high | 9.00 | 0.000837 |
| METTL13 | 51603 | chr1 | 171786000 | 171788999 | low | -13.04 | 0.000300 |
| METTL15 | 196074 | chr11 | 28107400 | 28108799 | high | 3.50 | 0.006976 |
| METTL15 | 196074 | chr11 | 28129800 | 28133799 | high | 10.00 | 0.006970 |
| METTL15 | 196074 | chr11 | 28172600 | 28177599 | high | 12.50 | 0.006960 |
| METTL15 | 196074 | chr11 | 28110000 | 28116599 | high | 16.50 | 0.006975 |
| METTL15 | 196074 | chr11 | 28144200 | 28151399 | high | 18.00 | 0.006967 |
| METTL18 | 92342 | chr1 | 169793800 | 169797399 | high | 9.00 | 0.000544 |
| METTL25 | 84190 | chr12 | 82357600 | 82359999 | high | 6.00 | 0.001022 |
| METTL26 | 84326 | chr16 | 634600 | 636199 | low | -6.95 | 0.014765 |
| METTL8 | 79828 | chr2 | 171432200 | 171434599 | high | 6.00 | 0.000466 |
| METTL8 | 79828 | chr2 | 171401800 | 171410799 | high | 22.50 | 0.000466 |
| METTL9 | 51108 | chr16 | 21602800 | 21606199 | high | 8.50 | 0.002366 |
| METTL9 | 51108 | chr16 | 21651600 | 21656199 | high | 11.50 | 0.002360 |
| MEX3C | 51320 | chr18 | 51196000 | 51197599 | low | -6.95 | 0.001002 |
| MEX3D | 399664 | chr19 | 1562600 | 1567999 | high | 13.50 | 0.001019 |
| MFAP1 | 4236 | chr15 | 43824200 | 43824999 | low | -3.47 | 0.000097 |
| MFAP2 | 4237 | chr1 | 16977800 | 16980599 | high | 7.00 | 0.000250 |
| MFAP3 | 4238 | chr5 | 154038000 | 154042199 | low | -18.44 | 0.000028 |
| MFGE8 | 4240 | chr15 | 88911000 | 88913799 | high | 7.00 | 0.000048 |
| MFN2 | 9927 | chr1 | 11979800 | 11984199 | high | 11.00 | 0.000829 |

| | | | | | | | |
|---|---|---|---|---|---|---|---|
| MFSD1 | 64747 | chr3 | 158820600 | 158822999 | low | -10.43 | 0.000408 |
| MFSD1 | 64747 | chr3 | 158827000 | 158828999 | low | -8.69 | 0.000408 |
| MFSD10 | 10227 | chr4 | 2932800 | 2934999 | low | -9.56 | 0.003487 |
| MFSD12 | 126321 | chr19 | 3555800 | 3557799 | low | -8.69 | 0.003947 |
| MFSD3 | 113655 | chr8 | 144508600 | 144511199 | high | 6.50 | 0.000786 |
| MFSD4B | 91749 | chr6 | 111259000 | 111262199 | high | 8.00 | 0.000825 |
| MFSD5 | 84975 | chr12 | 53251600 | 53253399 | low | -7.82 | 0.001596 |
| MFSD6 | 54842 | chr2 | 190450400 | 190454399 | high | 10.00 | 0.000288 |
| MFSD6 | 54842 | chr2 | 190415800 | 190419999 | high | 10.50 | 0.000288 |
| MGA | 23269 | chr15 | 41731200 | 41734599 | low | -14.78 | 0.000558 |
| MGAT1 | 4245 | chr5 | 180800400 | 180803399 | high | 7.50 | 0.000023 |
| MGAT2 | 4247 | chr14 | 49619600 | 49623399 | low | -16.33 | 0.000086 |
| MGAT4B | 11282 | chr5 | 179804600 | 179808199 | high | 9.00 | 0.000063 |
| MGAT4B | 11282 | chr5 | 179794400 | 179797999 | high | 9.00 | 0.000063 |
| MGAT4C | 25834 | chr12 | 86728200 | 86734799 | high | 16.50 | 0.000298 |
| MGAT4C | 25834 | chr12 | 86652800 | 86659799 | high | 17.50 | 0.000298 |
| MGAT5 | 4249 | chr2 | 134254800 | 134257799 | high | 7.50 | 0.000032 |
| MGAT5 | 4249 | chr2 | 134450800 | 134454599 | high | 9.50 | 0.000032 |
| MGAT5 | 4249 | chr2 | 134401200 | 134405999 | high | 12.00 | 0.000032 |
| MGLL | 11343 | chr3 | 127766800 | 127769799 | high | 7.50 | 0.000089 |
| MGLL | 11343 | chr3 | 127697400 | 127700599 | high | 8.00 | 0.000089 |
| MGLL | 11343 | chr3 | 127734800 | 127738199 | high | 8.50 | 0.000089 |
| MGLL | 11343 | chr3 | 127720000 | 127723399 | high | 8.50 | 0.000089 |
| MGLL | 11343 | chr3 | 127724400 | 127728199 | high | 9.50 | 0.000089 |
| MGLL | 11343 | chr3 | 127785000 | 127789399 | high | 11.00 | 0.000089 |
| MGLL | 11343 | chr3 | 127753000 | 127758399 | high | 13.50 | 0.000089 |
| MGLL | 11343 | chr3 | 127778200 | 127783999 | high | 14.50 | 0.000089 |
| MGRN1 | 23295 | chr16 | 4624400 | 4626599 | high | 5.50 | 0.005037 |
| MGST1 | 4257 | chr12 | 16347200 | 16349399 | low | -9.56 | 0.000260 |
| MGST3 | 4259 | chr1 | 165630200 | 165633799 | high | 9.00 | 0.000026 |
| MIA | 8190 | chr19 | 40776400 | 40780999 | high | 11.50 | 0.000201 |
| MIA3 | 375056 | chr1 | 222639200 | 222646199 | high | 17.50 | 0.001685 |
| MIA3 | 375056 | chr1 | 222611200 | 222625199 | high | 35.00 | 0.001685 |
| MICAL2 | 9645 | chr11 | 12233800 | 12235999 | high | 5.50 | 0.000788 |
| MICAL2 | 9645 | chr11 | 12260400 | 12262999 | high | 6.50 | 0.000787 |
| MICAL2 | 9645 | chr11 | 12165200 | 12168199 | high | 7.50 | 0.000793 |
| MICAL2 | 9645 | chr11 | 12147600 | 12150999 | high | 8.50 | 0.000794 |
| MICAL2 | 9645 | chr11 | 12110000 | 12113999 | high | 10.00 | 0.000796 |
| MICAL2 | 9645 | chr11 | 12134200 | 12138999 | high | 12.00 | 0.000795 |
| MICAL2 | 9645 | chr11 | 12119600 | 12128599 | high | 22.50 | 0.000796 |
| MICAL2 | 9645 | chr11 | 12178000 | 12187799 | high | 24.50 | 0.000792 |
| MICAL2 | 9645 | chr11 | 12237600 | 12249599 | high | 30.00 | 0.000788 |
| MICALCL | 84953 | chr11 | 12321600 | 12323799 | high | 5.50 | 0.006895 |
| MICALCL | 84953 | chr11 | 12291200 | 12295999 | high | 12.00 | 0.006912 |
| MICALCL | 84953 | chr11 | 12284400 | 12289999 | high | 14.00 | 0.006916 |
| MICALL1 | 85377 | chr22 | 37928200 | 37932599 | high | 11.00 | 0.002251 |
| MICALL1 | 85377 | chr22 | 37903400 | 37908599 | high | 13.00 | 0.002252 |
| MICALL2 | 79778 | chr7 | 1453600 | 1459399 | high | 14.50 | 0.008982 |
| MICU3 | 286097 | chr8 | 17079400 | 17083799 | high | 11.00 | 0.016751 |
| MICU3 | 286097 | chr8 | 17105000 | 17110399 | high | 13.50 | 0.016726 |
| MID1 | 4281 | chrX | 10618600 | 10621399 | low | -12.17 | 0.000403 |
| MIDN | 90007 | chr19 | 1248400 | 1253399 | high | 12.50 | 0.008011 |
| MIEF1 | 54471 | chr22 | 39513000 | 39517199 | high | 10.50 | 0.001379 |
| MIEN1 | 84299 | chr17 | 39729200 | 39730799 | low | -6.95 | 0.002122 |
| MIER1 | 57708 | chr1 | 66969200 | 66971199 | low | -8.69 | 0.000862 |
| MIER1 | 57708 | chr1 | 66931200 | 66932999 | low | -7.82 | 0.000862 |
| MIER2 | 54531 | chr19 | 341400 | 345199 | high | 9.50 | 0.006714 |
| MIF4GD | 57409 | chr17 | 75269400 | 75273199 | high | 9.50 | 0.000763 |
| MIF4GD | 57409 | chr17 | 75259800 | 75267799 | high | 20.00 | 0.000763 |
| MIGA1 | 374986 | chr1 | 77778200 | 77781199 | high | 7.50 | 0.004821 |
| MIGA1 | 374986 | chr1 | 77785800 | 77790199 | high | 11.00 | 0.004821 |
| MIGA2 | 84895 | chr9 | 129057400 | 129061199 | high | 9.50 | 0.000658 |
| MIGA2 | 84895 | chr9 | 129068400 | 129072399 | high | 10.00 | 0.000658 |
| MILR1 | 284021 | chr17 | 64453600 | 64458999 | high | 13.50 | 0.004407 |
| MINPP1 | 9562 | chr10 | 87551200 | 87554399 | high | 8.00 | 0.000109 |
| MINPP1 | 9562 | chr10 | 87542400 | 87545799 | high | 8.50 | 0.000109 |
| MINPP1 | 9562 | chr10 | 87531000 | 87535399 | high | 11.00 | 0.000109 |
| MIOS | 54468 | chr7 | 7566600 | 7570399 | high | 9.50 | 0.007198 |
| MIP | 4284 | chr12 | 56447200 | 56454399 | high | 18.00 | 0.000076 |
| MIPEP | 4285 | chr13 | 23823200 | 23826599 | high | 8.50 | 0.000180 |
| MIPEP | 4285 | chr13 | 23886800 | 23890399 | high | 9.00 | 0.000179 |
| MIPEP | 4285 | chr13 | 23818800 | 23822399 | high | 9.00 | 0.000180 |
| MIPOL1 | 145282 | chr14 | 37378400 | 37380399 | high | 5.00 | 0.003887 |
| MLEC | 9761 | chr12 | 120686200 | 120688799 | low | -11.30 | 0.000081 |
| MLH1 | 4292 | chr3 | 37037400 | 37041999 | high | 11.50 | 0.000116 |
| MLH1 | 4292 | chr3 | 37031000 | 37036199 | high | 13.00 | 0.000116 |
| MLH3 | 27030 | chr14 | 75015600 | 75022999 | low | -32.16 | 0.000360 |
| MLLT1 | 4298 | chr19 | 6277600 | 6281799 | high | 10.50 | 0.000685 |

| | | | | | | | |
|---|---|---|---|---|---|---|---|
| MLLT1 | 4298 | chr19 | 6216200 | 6221199 | high | 12.50 | 0.000691 |
| MLLT10 | 8028 | chr10 | 21717600 | 21721199 | low | -15.65 | 0.000370 |
| MLLT10 | 8028 | chr10 | 21664400 | 21667799 | low | -14.78 | 0.000371 |
| MLLT10 | 8028 | chr10 | 21672000 | 21674799 | low | -12.17 | 0.000370 |
| MLLT3 | 4300 | chr9 | 20367800 | 20370799 | high | 7.50 | 0.000211 |
| MLLT3 | 4300 | chr9 | 20386400 | 20389599 | high | 8.00 | 0.000211 |
| MLLT3 | 4300 | chr9 | 20473000 | 20476799 | high | 9.50 | 0.000210 |
| MLLT3 | 4300 | chr9 | 20486200 | 20490599 | high | 11.00 | 0.000210 |
| MLLT3 | 4300 | chr9 | 20605200 | 20610199 | high | 12.50 | 0.000209 |
| MLLT3 | 4300 | chr9 | 20614400 | 20619999 | high | 14.00 | 0.000209 |
| MLST8 | 64223 | chr16 | 2204200 | 2206199 | high | 5.00 | 0.003237 |
| MLX | 6945 | chr17 | 42565000 | 42573599 | high | 21.50 | 0.000163 |
| MLXIP | 22877 | chr12 | 122104200 | 122110199 | high | 15.00 | 0.000187 |
| MLXIP | 22877 | chr12 | 122093400 | 122103399 | high | 25.00 | 0.000187 |
| MME | 4311 | chr3 | 155089200 | 155092599 | high | 8.50 | 0.000028 |
| MME | 4311 | chr3 | 155104200 | 155110199 | high | 15.00 | 0.000028 |
| MMP1 | 4312 | chr11 | 102790800 | 102795199 | high | 11.00 | 0.000042 |
| MMP13 | 4322 | chr11 | 102949200 | 102956999 | high | 19.50 | 0.000042 |
| MMP16 | 4325 | chr8 | 88304400 | 88307599 | high | 8.00 | 0.000049 |
| MMP16 | 4325 | chr8 | 88227400 | 88231399 | high | 10.00 | 0.000049 |
| MMP16 | 4325 | chr8 | 88265600 | 88269999 | high | 11.00 | 0.000049 |
| MMP16 | 4325 | chr8 | 88277200 | 88283999 | high | 17.00 | 0.000049 |
| MMP2 | 4313 | chr16 | 55478600 | 55480399 | low | -7.82 | 0.000078 |
| MMP20 | 9313 | chr11 | 102588800 | 102593599 | high | 12.00 | 0.000091 |
| MMP24 | 10893 | chr20 | 35269200 | 35272999 | high | 9.50 | 0.000309 |
| MMP24 | 10893 | chr20 | 35274200 | 35278399 | high | 10.50 | 0.000309 |
| MMP24 | 10893 | chr20 | 35241600 | 35247399 | high | 14.50 | 0.000309 |
| MMP27 | 64066 | chr11 | 102700000 | 102703999 | high | 10.00 | 0.000624 |
| MMP3 | 4314 | chr11 | 102839400 | 102842399 | low | -13.04 | 0.000042 |
| MMP7 | 4316 | chr11 | 102523000 | 102526199 | high | 8.00 | 0.000042 |
| MMP7 | 4316 | chr11 | 102528400 | 102532399 | high | 10.00 | 0.000042 |
| MMP8 | 4317 | chr11 | 102712200 | 102715799 | high | 9.00 | 0.000042 |
| MMS19 | 64210 | chr10 | 97477600 | 97481199 | high | 9.00 | 0.000659 |
| MNAT1 | 4331 | chr14 | 60773800 | 60779399 | high | 14.00 | 0.000071 |
| MNAT1 | 4331 | chr14 | 60745200 | 60750799 | high | 14.00 | 0.000071 |
| MNAT1 | 4331 | chr14 | 60761800 | 60768999 | high | 18.00 | 0.000071 |
| MOB1A | 55233 | chr2 | 74176600 | 74179799 | high | 8.00 | 0.000745 |
| MOB1B | 92597 | chr4 | 70978800 | 70980999 | low | -9.56 | 0.001305 |
| MOB3A | 126308 | chr19 | 2086400 | 2089799 | high | 8.50 | 0.003873 |
| MOB4 | 25843 | chr2 | 197514200 | 197517799 | high | 9.00 | 0.000131 |
| MOK | 5891 | chr14 | 102224800 | 102229799 | high | 12.50 | 0.000058 |
| MOK | 5891 | chr14 | 102276800 | 102283799 | high | 17.50 | 0.000058 |
| MON2 | 23041 | chr12 | 62466400 | 62469999 | low | -15.65 | 0.000369 |
| MON2 | 23041 | chr12 | 62539400 | 62541799 | low | -10.43 | 0.000368 |
| MORC2 | 22880 | chr22 | 30947400 | 30952399 | high | 12.50 | 0.000739 |
| MORC3 | 23515 | chr21 | 36362200 | 36364599 | high | 6.00 | 0.000647 |
| MORN1 | 79906 | chr1 | 2389600 | 2392799 | high | 8.00 | 0.003715 |
| MORN3 | 283385 | chr12 | 121651000 | 121656199 | high | 13.00 | 0.002329 |
| MORN4 | 118812 | chr10 | 97631800 | 97634399 | low | -11.30 | 0.001217 |
| MOV10 | 4343 | chr1 | 112672800 | 112677199 | high | 11.00 | 0.000039 |
| MOXD1 | 26002 | chr6 | 132381400 | 132384399 | high | 7.50 | 0.000196 |
| MOXD1 | 26002 | chr6 | 132397200 | 132402599 | high | 13.50 | 0.000196 |
| MPDZ | 8777 | chr9 | 13118400 | 13121799 | high | 8.50 | 0.000669 |
| MPG | 4350 | chr16 | 76600 | 80399 | high | 9.50 | 0.006310 |
| MPG | 4350 | chr16 | 81400 | 86599 | high | 13.00 | 0.005938 |
| MPL | 4352 | chr1 | 43348600 | 43350599 | low | -8.69 | 0.000100 |
| MPP4 | 58538 | chr2 | 201690600 | 201694199 | high | 9.00 | 0.000290 |
| MPP4 | 58538 | chr2 | 201642200 | 201648199 | high | 15.00 | 0.000290 |
| MPP5 | 64398 | chr14 | 67240400 | 67242799 | high | 6.00 | 0.000958 |
| MPP6 | 51678 | chr7 | 24604400 | 24607199 | high | 7.00 | 0.002100 |
| MPP7 | 143098 | chr10 | 28125000 | 28128999 | low | -17.39 | 0.005088 |
| MPRIP | 23164 | chr17 | 17150000 | 17152999 | high | 7.50 | 0.001351 |
| MPRIP | 23164 | chr17 | 17041800 | 17046599 | high | 12.00 | 0.001359 |
| MPRIP | 23164 | chr17 | 17121400 | 17126799 | high | 13.50 | 0.001353 |
| MPST | 4357 | chr22 | 37018600 | 37021399 | low | -12.17 | 0.000118 |
| MPZL1 | 9019 | chr1 | 167744600 | 167748599 | high | 10.00 | 0.000054 |
| MPZL1 | 9019 | chr1 | 167723400 | 167727599 | high | 10.50 | 0.000054 |
| MRC2 | 9902 | chr17 | 62652200 | 62655199 | high | 7.50 | 0.000158 |
| MRC2 | 9902 | chr17 | 62685600 | 62689799 | high | 10.50 | 0.000158 |
| MRI1 | 84245 | chr19 | 13764200 | 13766599 | low | -10.43 | 0.006121 |
| MRM2 | 29960 | chr7 | 2240800 | 2243799 | high | 7.50 | 0.013370 |
| MRM3 | 55178 | chr17 | 780200 | 783199 | low | -13.04 | 0.007858 |
| MRPL1 | 65008 | chr4 | 77924400 | 77930599 | high | 15.50 | 0.000834 |
| MRPL10 | 124995 | chr17 | 47830800 | 47833399 | high | 6.50 | 0.002613 |
| MRPL15 | 29088 | chr8 | 54134800 | 54137999 | high | 8.00 | 0.000537 |
| MRPL17 | 63875 | chr11 | 6681800 | 6683999 | high | 5.50 | 0.009560 |
| MRPL18 | 29074 | chr6 | 159787400 | 159791199 | high | 9.50 | 0.000182 |
| MRPL2 | 51069 | chr6 | 43057000 | 43060399 | high | 8.50 | 0.001186 |

| | | | | | | | |
|---|---|---|---|---|---|---|---|
| MRPL2 | 51069 | chr6 | 43049400 | 43054199 | high | 12.00 | 0.001186 |
| MRPL20 | 55052 | chr1 | 1401400 | 1404799 | high | 8.50 | 0.004365 |
| MRPL24 | 79590 | chr1 | 156738000 | 156743599 | high | 14.00 | 0.000508 |
| MRPL27 | 51264 | chr17 | 50370800 | 50373999 | low | -13.91 | 0.001018 |
| MRPL3 | 11222 | chr3 | 131501800 | 131503399 | high | 4.00 | 0.000085 |
| MRPL30 | 51263 | chr2 | 99180600 | 99181999 | low | -6.08 | 0.000517 |
| MRPL32 | 64983 | chr7 | 42930600 | 42933799 | low | -13.91 | 0.001514 |
| MRPL33 | 9553 | chr2 | 27776600 | 27780799 | high | 10.50 | 0.000344 |
| MRPL38 | 64978 | chr17 | 75903000 | 75905799 | low | -12.17 | 0.000856 |
| MRPL39 | 54148 | chr21 | 25605200 | 25607799 | high | 6.50 | 0.002115 |
| MRPL4 | 51073 | chr19 | 10251800 | 10252599 | high | 2.00 | 0.004982 |
| MRPL44 | 65080 | chr2 | 223956200 | 223959599 | low | -14.78 | 0.000291 |
| MRPL46 | 26589 | chr15 | 88465800 | 88468599 | high | 7.00 | 0.000301 |
| MRPL48 | 51642 | chr11 | 73824600 | 73828799 | high | 10.50 | 0.000700 |
| MRPL48 | 51642 | chr11 | 73844400 | 73849399 | high | 12.50 | 0.000699 |
| MRPL49 | 740 | chr11 | 65120400 | 65122799 | low | -10.43 | 0.000011 |
| MRPL53 | 116540 | chr2 | 74471800 | 74472599 | low | -3.47 | 0.001565 |
| MRPL54 | 116541 | chr19 | 3759200 | 3763199 | high | 10.00 | 0.003445 |
| MRPL55 | 128308 | chr1 | 228107800 | 228109199 | low | -6.08 | 0.000562 |
| MRPL9 | 65005 | chr1 | 151763400 | 151763999 | low | -2.60 | 0.000428 |
| MRPS11 | 64963 | chr15 | 88465800 | 88468599 | high | 7.00 | 0.000734 |
| MRPS12 | 6183 | chr19 | 38928200 | 38932999 | low | -20.83 | 0.000159 |
| MRPS16 | 51021 | chr10 | 73244200 | 73249599 | high | 13.50 | 0.000697 |
| MRPS25 | 64432 | chr3 | 15056200 | 15060999 | high | 12.00 | 0.004279 |
| MRPS26 | 64949 | chr20 | 3043000 | 3047199 | high | 10.50 | 0.002372 |
| MRPS28 | 28957 | chr8 | 79991400 | 79994599 | low | -13.91 | 0.000362 |
| MRPS35 | 60488 | chr12 | 27739400 | 27742999 | high | 9.00 | 0.002181 |
| MRPS5 | 64969 | chr2 | 95120600 | 95122999 | low | -10.43 | 0.000683 |
| MRRF | 92399 | chr9 | 122306800 | 122310599 | high | 9.50 | 0.000755 |
| MRRF | 92399 | chr9 | 122275800 | 122280999 | high | 13.00 | 0.000756 |
| MRVI1 | 10335 | chr11 | 10596800 | 10599599 | high | 7.00 | 0.000975 |
| MRVI1 | 10335 | chr11 | 10591000 | 10595999 | high | 12.50 | 0.000976 |
| MRVI1 | 10335 | chr11 | 10684400 | 10689599 | high | 13.00 | 0.000967 |
| MRVI1 | 10335 | chr11 | 10632000 | 10637999 | high | 15.00 | 0.000972 |
| MRVI1 | 10335 | chr11 | 10651800 | 10660999 | high | 23.00 | 0.000970 |
| MSH2 | 4436 | chr2 | 47467200 | 47471799 | high | 11.50 | 0.000093 |
| MSH2 | 4436 | chr2 | 47451600 | 47456599 | high | 12.50 | 0.000093 |
| MSH3 | 4437 | chr5 | 80685400 | 80688399 | high | 7.50 | 0.000055 |
| MSH3 | 4437 | chr5 | 80749200 | 80754799 | high | 14.00 | 0.000055 |
| MSH3 | 4437 | chr5 | 80706200 | 80714399 | high | 20.50 | 0.000055 |
| MSL1 | 339287 | chr17 | 40121200 | 40124199 | low | -13.04 | 0.008457 |
| MSL2 | 55167 | chr3 | 136159000 | 136163799 | high | 12.00 | 0.000405 |
| MSL2 | 55167 | chr3 | 136193200 | 136198199 | high | 12.50 | 0.000405 |
| MSL2 | 55167 | chr3 | 136149600 | 136154599 | high | 12.50 | 0.000405 |
| MSN | 4478 | chrX | 65696200 | 65698399 | low | -9.56 | 0.000068 |
| MSR1 | 4481 | chr8 | 16108800 | 16112799 | high | 10.00 | 0.000278 |
| MSRA | 4482 | chr8 | 10052600 | 10055999 | high | 8.50 | 0.000446 |
| MSRB3 | 253827 | chr12 | 65342400 | 65345199 | high | 7.00 | 0.003885 |
| MSRB3 | 253827 | chr12 | 65381400 | 65384999 | high | 9.00 | 0.003882 |
| MSRB3 | 253827 | chr12 | 65332800 | 65336799 | high | 10.00 | 0.003885 |
| MSRB3 | 253827 | chr12 | 65320600 | 65329399 | high | 22.00 | 0.003886 |
| MTA1 | 9112 | chr14 | 105418000 | 105420999 | high | 7.50 | 0.000086 |
| MTA3 | 57504 | chr2 | 42658800 | 42659799 | low | -4.34 | 0.001348 |
| MTCL1 | 23255 | chr18 | 8818400 | 8822599 | high | 10.50 | 0.002637 |
| MTCL1 | 23255 | chr18 | 8746000 | 8753599 | high | 19.00 | 0.002659 |
| MTDH | 92140 | chr8 | 97644400 | 97646999 | high | 6.50 | 0.000944 |
| MTERF1 | 7978 | chr7 | 91879400 | 91881599 | low | -9.56 | 0.000087 |
| MTERF2 | 80298 | chr12 | 106984800 | 106989399 | high | 11.50 | 0.000751 |
| MTF1 | 4520 | chr1 | 37848800 | 37853399 | high | 11.50 | 0.000119 |
| MTF2 | 22823 | chr1 | 93113400 | 93116799 | high | 8.50 | 0.000245 |
| MTF2 | 22823 | chr1 | 93080400 | 93083999 | high | 9.00 | 0.000245 |
| MTF2 | 22823 | chr1 | 93133600 | 93137999 | high | 11.00 | 0.000245 |
| MTFP1 | 51537 | chr22 | 30423400 | 30429399 | high | 15.00 | 0.001694 |
| MTFR1L | 56181 | chr1 | 25819400 | 25820799 | low | -6.08 | 0.002176 |
| MTHFD1L | 25902 | chr6 | 150864600 | 150867399 | high | 7.00 | 0.000172 |
| MTHFD2L | 441024 | chr4 | 74171600 | 74175399 | high | 9.50 | 0.005946 |
| MTHFD2L | 441024 | chr4 | 74156600 | 74162799 | high | 15.50 | 0.005947 |
| MTHFR | 4524 | chr1 | 11805000 | 11806999 | high | 5.00 | 0.000383 |
| MTHFSD | 64779 | chr16 | 86554200 | 86556799 | high | 6.50 | 0.000748 |
| MTMR12 | 54545 | chr5 | 32302000 | 32305199 | low | -13.91 | 0.001689 |
| MTMR2 | 8898 | chr11 | 95846800 | 95849799 | high | 7.50 | 0.000093 |
| MTMR2 | 8898 | chr11 | 95832400 | 95837399 | high | 12.50 | 0.000093 |
| MTMR6 | 9107 | chr13 | 25286200 | 25288799 | low | -11.30 | 0.000360 |
| MTPAP | 55149 | chr10 | 30347400 | 30349999 | low | -11.30 | 0.001817 |
| MTRF1 | 9617 | chr13 | 41262600 | 41263799 | high | 3.00 | 0.000233 |
| MTRF1 | 9617 | chr13 | 41241400 | 41245999 | high | 11.50 | 0.000233 |
| MTRF1 | 9617 | chr13 | 41251400 | 41258399 | high | 17.50 | 0.000233 |
| MTRF1L | 54516 | chr6 | 153002000 | 153003199 | low | -5.21 | 0.000356 |

| | | | | | | | |
|---|---|---|---|---|---|---|---|
| MTSS1L | 92154 | chr16 | 70683400 | 70686399 | high | 7.50 | 0.001304 |
| MTUS1 | 57509 | chr8 | 17660000 | 17665199 | high | 13.00 | 0.003256 |
| MTX1 | 4580 | chr1 | 155208400 | 155209599 | high | 3.00 | 0.000030 |
| MTX2 | 10651 | chr2 | 176326400 | 176328199 | low | -7.82 | 0.000060 |
| MTX3 | 345778 | chr5 | 79981400 | 79986199 | high | 12.00 | 0.004323 |
| MUS81 | 80198 | chr11 | 65860400 | 65861199 | low | -3.47 | 0.001218 |
| MUSK | 4593 | chr9 | 110754000 | 110755999 | high | 5.00 | 0.000041 |
| MUSK | 4593 | chr9 | 110728200 | 110730399 | high | 5.50 | 0.000041 |
| MUSK | 4593 | chr9 | 110715200 | 110718199 | high | 7.50 | 0.000041 |
| MUSK | 4593 | chr9 | 110781400 | 110784599 | high | 8.00 | 0.000041 |
| MUSK | 4593 | chr9 | 110786800 | 110790599 | high | 9.50 | 0.000041 |
| MUSK | 4593 | chr9 | 110668800 | 110673199 | high | 11.00 | 0.000042 |
| MUSK | 4593 | chr9 | 110740400 | 110745199 | high | 12.00 | 0.000041 |
| MUSK | 4593 | chr9 | 110691200 | 110699999 | high | 22.00 | 0.000041 |
| MVD | 4597 | chr16 | 88655600 | 88658799 | high | 8.00 | 0.000052 |
| MX2 | 4600 | chr21 | 41367000 | 41373599 | high | 16.50 | 0.000111 |
| MXD3 | 83463 | chr5 | 177300600 | 177312599 | high | 30.00 | 0.000471 |
| MXD4 | 10608 | chr4 | 2258600 | 2263599 | high | 12.50 | 0.004697 |
| MXI1 | 4601 | chr10 | 110209000 | 110212599 | high | 9.00 | 0.000042 |
| MXRA7 | 439921 | chr17 | 76707800 | 76712999 | high | 13.00 | 0.005735 |
| MXRA8 | 54587 | chr1 | 1357000 | 1362199 | high | 13.00 | 0.004470 |
| MYADM | 91663 | chr19 | 53867600 | 53869799 | high | 5.50 | 0.001702 |
| MYB | 4602 | chr6 | 135178800 | 135186399 | high | 19.00 | 0.000034 |
| MYBL1 | 4603 | chr8 | 66564000 | 66568399 | high | 11.00 | 0.000069 |
| MYBPHL | 343263 | chr1 | 109293200 | 109300999 | high | 19.50 | 0.003141 |
| MYCBP | 26292 | chr1 | 38872000 | 38876599 | high | 11.50 | 0.000676 |
| MYCBP2 | 23077 | chr13 | 77073400 | 77077999 | high | 11.50 | 0.000299 |
| MYCBP2 | 23077 | chr13 | 77122800 | 77127599 | high | 12.00 | 0.000299 |
| MYCBP2 | 23077 | chr13 | 77051600 | 77058399 | high | 17.00 | 0.000300 |
| MYDGF | 56005 | chr19 | 4667600 | 4670599 | high | 7.50 | 0.011999 |
| MYH7B | 57644 | chr20 | 34955200 | 34958599 | high | 8.50 | 0.001649 |
| MYH9 | 4627 | chr22 | 36378200 | 36380799 | high | 6.50 | 0.000127 |
| MYH9 | 4627 | chr22 | 36349400 | 36352199 | high | 7.00 | 0.000127 |
| MYL4 | 4635 | chr17 | 47217800 | 47226599 | high | 22.00 | 0.000098 |
| MYL5 | 4636 | chr4 | 676600 | 679599 | high | 7.50 | 0.006852 |
| MYL6 | 4637 | chr12 | 56157800 | 56161399 | low | -15.65 | 0.000083 |
| MYL6B | 140465 | chr12 | 56157800 | 56161399 | low | -15.65 | 0.002501 |
| MYLIP | 29116 | chr6 | 16128000 | 16132199 | high | 10.50 | 0.001805 |
| MYLK | 4638 | chr3 | 123812000 | 123813999 | high | 5.00 | 0.000037 |
| MYLK | 4638 | chr3 | 123763600 | 123767399 | high | 9.50 | 0.000037 |
| MYLK | 4638 | chr3 | 123880400 | 123884599 | high | 10.50 | 0.000037 |
| MYLK | 4638 | chr3 | 123789000 | 123793399 | high | 11.00 | 0.000037 |
| MYLK | 4638 | chr3 | 123818200 | 123823199 | high | 12.50 | 0.000037 |
| MYLK | 4638 | chr3 | 123865800 | 123872599 | high | 17.00 | 0.000037 |
| MYLK | 4638 | chr3 | 123780000 | 123786999 | high | 17.50 | 0.000037 |
| MYLK4 | 340156 | chr6 | 2732200 | 2734399 | low | -9.56 | 0.013833 |
| MYLK4 | 340156 | chr6 | 2709400 | 2711199 | low | -7.82 | 0.013950 |
| MYLPF | 29895 | chr16 | 30377600 | 30381999 | high | 11.00 | 0.000984 |
| MYO10 | 4651 | chr5 | 16929400 | 16931999 | high | 6.50 | 0.000275 |
| MYO10 | 4651 | chr5 | 16933600 | 16937199 | high | 9.00 | 0.000275 |
| MYO10 | 4651 | chr5 | 16817000 | 16820799 | high | 9.50 | 0.000277 |
| MYO10 | 4651 | chr5 | 16862200 | 16866199 | high | 10.00 | 0.000276 |
| MYO10 | 4651 | chr5 | 16710800 | 16714999 | high | 10.50 | 0.000278 |
| MYO10 | 4651 | chr5 | 16912000 | 16918199 | high | 15.50 | 0.000275 |
| MYO10 | 4651 | chr5 | 16875200 | 16882199 | high | 17.50 | 0.000276 |
| MYO15A | 51168 | chr17 | 18127400 | 18132999 | high | 14.00 | 0.002823 |
| MYO15B | 80022 | chr17 | 75620800 | 75625599 | high | 12.00 | 0.001058 |
| MYO18A | 399687 | chr17 | 29178000 | 29181799 | high | 9.50 | 0.013698 |
| MYO18A | 399687 | chr17 | 29138600 | 29144199 | high | 14.00 | 0.013717 |
| MYO18A | 399687 | chr17 | 29126000 | 29132599 | high | 16.50 | 0.013723 |
| MYO1B | 4430 | chr2 | 191417400 | 191422399 | high | 12.50 | 0.000023 |
| MYO1B | 4430 | chr2 | 191410600 | 191415999 | high | 13.50 | 0.000023 |
| MYO1B | 4430 | chr2 | 191384200 | 191392599 | high | 21.00 | 0.000023 |
| MYO1E | 4643 | chr15 | 59171000 | 59175599 | high | 11.50 | 0.000078 |
| MYO1E | 4643 | chr15 | 59183200 | 59192199 | high | 22.50 | 0.000078 |
| MYO1F | 4542 | chr19 | 8551600 | 8555399 | high | 9.50 | 0.000531 |
| MYO5A | 4644 | chr15 | 52390600 | 52393399 | high | 7.00 | 0.000089 |
| MYO5A | 4644 | chr15 | 52382400 | 52385399 | high | 7.50 | 0.000089 |
| MYO6 | 4646 | chr6 | 75757800 | 75762599 | high | 12.00 | 0.000061 |
| MYO6 | 4646 | chr6 | 75747200 | 75753799 | high | 16.50 | 0.000061 |
| MYO7A | 4647 | chr11 | 77195200 | 77197199 | low | -8.69 | 0.000060 |
| MYO9A | 4649 | chr15 | 72054600 | 72057199 | low | -11.30 | 0.000065 |
| MYO9A | 4649 | chr15 | 72066000 | 72070399 | high | 11.00 | 0.000065 |
| MYO9A | 4649 | chr15 | 72037400 | 72042999 | high | 14.00 | 0.000065 |
| MYO9A | 4649 | chr15 | 71984000 | 71989999 | high | 15.00 | 0.000065 |
| MYO9A | 4649 | chr15 | 72007000 | 72014599 | high | 19.00 | 0.000065 |
| MYO9B | 4650 | chr19 | 17100600 | 17105199 | high | 11.50 | 0.000272 |
| MYO9B | 4650 | chr19 | 17108000 | 17112799 | high | 12.00 | 0.000272 |

| | | | | | | | |
|---|---|---|---|---|---|---|---|
| MYO9B | 4650 | chr19 | 17089400 | 17095799 | high | 16.00 | 0.000272 |
| MYO9B | 4650 | chr19 | 17208600 | 17216799 | high | 20.50 | 0.000270 |
| MYOF | 26509 | chr10 | 93445800 | 93449599 | high | 9.50 | 0.000284 |
| MYOF | 26509 | chr10 | 93468400 | 93472799 | high | 11.00 | 0.000284 |
| MYOF | 26509 | chr10 | 93430800 | 93438999 | high | 20.50 | 0.000284 |
| MYOM1 | 8736 | chr18 | 3147200 | 3149799 | low | -11.30 | 0.002776 |
| MYOM1 | 8736 | chr18 | 3093200 | 3096799 | high | 9.00 | 0.002824 |
| MYOM1 | 8736 | chr18 | 3080400 | 3087599 | high | 18.00 | 0.002836 |
| MYOZ2 | 51778 | chr4 | 119149600 | 119153799 | high | 10.50 | 0.000435 |
| MYOZ2 | 51778 | chr4 | 119177400 | 119182399 | high | 12.50 | 0.000434 |
| MYOZ2 | 51778 | chr4 | 119154800 | 119160599 | high | 14.50 | 0.000435 |
| MYOZ3 | 91977 | chr5 | 150671200 | 150673599 | low | -10.43 | 0.000610 |
| MYPN | 84665 | chr10 | 68119600 | 68122599 | high | 7.50 | 0.001243 |
| MYPN | 84665 | chr10 | 68147400 | 68151799 | high | 11.00 | 0.001242 |
| MYPN | 84665 | chr10 | 68108400 | 68116599 | high | 20.50 | 0.001243 |
| MYSM1 | 114803 | chr1 | 58698800 | 58699999 | low | -5.21 | 0.001956 |
| MYT1L | 23040 | chr2 | 2295400 | 2295999 | low | -2.60 | 0.010037 |
| MZF1 | 7593 | chr19 | 58572400 | 58575799 | low | -14.78 | 0.000130 |
| MZF1 | 7593 | chr19 | 58563200 | 58564599 | low | -6.08 | 0.000130 |
| N4BP1 | 9683 | chr16 | 48609400 | 48610799 | low | -6.08 | 0.000199 |
| N4BP2 | 55728 | chr4 | 40100400 | 40103399 | high | 7.50 | 0.001390 |
| N4BP2 | 55728 | chr4 | 40095400 | 40098599 | high | 8.00 | 0.001390 |
| N4BP2 | 55728 | chr4 | 40152200 | 40156799 | high | 11.50 | 0.001388 |
| N4BP2 | 55728 | chr4 | 40053600 | 40059799 | high | 15.50 | 0.001391 |
| NAA16 | 79612 | chr13 | 41359400 | 41361799 | high | 6.00 | 0.001925 |
| NAA16 | 79612 | chr13 | 41355800 | 41358599 | high | 7.00 | 0.001925 |
| NAA16 | 79612 | chr13 | 41368800 | 41371799 | high | 7.50 | 0.001924 |
| NAA20 | 51126 | chr20 | 20016800 | 20017799 | high | 2.50 | 0.002554 |
| NAA25 | 80018 | chr12 | 112106200 | 112110599 | high | 11.00 | 0.000714 |
| NAA35 | 60560 | chr9 | 85941400 | 85943199 | high | 4.50 | 0.000705 |
| NAA60 | 79903 | chr16 | 3453800 | 3459799 | high | 15.00 | 0.002571 |
| NAAA | 27163 | chr4 | 75916400 | 75919999 | high | 9.00 | 0.000358 |
| NABP2 | 79035 | chr12 | 56224000 | 56225999 | high | 5.00 | 0.001406 |
| NACAD | 23148 | chr7 | 45084600 | 45089999 | high | 13.50 | 0.000513 |
| NACC1 | 112939 | chr19 | 13131400 | 13136799 | high | 13.50 | 0.008601 |
| NADK2 | 133686 | chr5 | 36236800 | 36239399 | high | 6.50 | 0.003689 |
| NADK2 | 133686 | chr5 | 36226000 | 36229199 | high | 8.00 | 0.003690 |
| NAE1 | 8883 | chr16 | 66804800 | 66809199 | high | 11.00 | 0.000133 |
| NAF1 | 92345 | chr4 | 163165000 | 163168799 | low | -16.52 | 0.000566 |
| NAGA | 4668 | chr22 | 42068600 | 42072199 | high | 9.00 | 0.000111 |
| NAGK | 55577 | chr2 | 71067400 | 71072599 | high | 13.00 | 0.000782 |
| NAGS | 162417 | chr17 | 44006400 | 44009599 | low | -13.91 | 0.003691 |
| NAIF1 | 203245 | chr9 | 128065600 | 128073799 | high | 20.50 | 0.001587 |
| NANP | 140838 | chr20 | 25622200 | 25625799 | high | 9.00 | 0.005497 |
| NAP1L1 | 4673 | chr12 | 76062400 | 76064199 | low | -7.82 | 0.000061 |
| NAP1L1 | 4673 | chr12 | 76074800 | 76076199 | low | -6.08 | 0.000061 |
| NAPB | 63908 | chr20 | 23386800 | 23392399 | high | 14.00 | 0.002733 |
| NAPG | 8774 | chr18 | 10531200 | 10533999 | high | 7.00 | 0.000833 |
| NAPG | 8774 | chr18 | 10524200 | 10527599 | high | 8.50 | 0.000834 |
| NAPG | 8774 | chr18 | 10540400 | 10544199 | high | 9.50 | 0.000832 |
| NARFL | 64428 | chr16 | 727200 | 732399 | high | 13.00 | 0.009844 |
| NARS | 4677 | chr18 | 57619600 | 57621999 | low | -10.43 | 0.000081 |
| NARS2 | 79731 | chr11 | 78572800 | 78575399 | low | -11.30 | 0.001015 |
| NARS2 | 79731 | chr11 | 78566800 | 78568599 | low | -7.82 | 0.001015 |
| NAT10 | 55226 | chr11 | 34105000 | 34107199 | high | 5.50 | 0.001619 |
| NAT10 | 55226 | chr11 | 34139800 | 34145599 | high | 14.50 | 0.001618 |
| NAT14 | 57106 | chr19 | 55482200 | 55486399 | high | 10.50 | 0.001029 |
| NAT9 | 26151 | chr17 | 74775200 | 74776999 | high | 4.50 | 0.000350 |
| NAV1 | 89796 | chr1 | 201653400 | 201655599 | high | 5.50 | 0.000445 |
| NAV1 | 89796 | chr1 | 201685600 | 201688799 | high | 8.00 | 0.000445 |
| NAV1 | 89796 | chr1 | 201790800 | 201795199 | high | 11.00 | 0.000445 |
| NAV1 | 89796 | chr1 | 201672200 | 201676999 | high | 12.00 | 0.000445 |
| NAV1 | 89796 | chr1 | 201660600 | 201666199 | high | 14.00 | 0.000445 |
| NAV1 | 89796 | chr1 | 201646200 | 201652399 | high | 15.50 | 0.000445 |
| NAV1 | 89796 | chr1 | 201824800 | 201831399 | high | 16.50 | 0.000445 |
| NAV2 | 89797 | chr11 | 19837400 | 19839999 | high | 6.50 | 0.004527 |
| NAV2 | 89797 | chr11 | 19921800 | 19924799 | high | 7.50 | 0.004507 |
| NAV2 | 89797 | chr11 | 20009000 | 20012199 | high | 8.00 | 0.004488 |
| NAV2 | 89797 | chr11 | 19927000 | 19930599 | high | 9.00 | 0.004506 |
| NAV2 | 89797 | chr11 | 19425400 | 19428999 | high | 9.00 | 0.004623 |
| NAV2 | 89797 | chr11 | 19711600 | 19715599 | high | 10.00 | 0.004556 |
| NAV2 | 89797 | chr11 | 19599800 | 19603799 | high | 10.00 | 0.004582 |
| NAV2 | 89797 | chr11 | 19386000 | 19389999 | high | 10.00 | 0.004632 |
| NAV2 | 89797 | chr11 | 19364200 | 19368399 | high | 10.50 | 0.004637 |
| NAV2 | 89797 | chr11 | 19993600 | 19997999 | high | 11.00 | 0.004491 |
| NAV2 | 89797 | chr11 | 19378400 | 19382799 | high | 11.00 | 0.004634 |
| NAV2 | 89797 | chr11 | 19940000 | 19944599 | high | 11.50 | 0.004503 |
| NAV2 | 89797 | chr11 | 19976600 | 19981599 | high | 12.50 | 0.004495 |

| | | | | | | | |
|---|---|---|---|---|---|---|---|
| NAV2 | 89797 | chr11 | 19605000 | 19610199 | high | 13.00 | 0.004580 |
| NAV2 | 89797 | chr11 | 19805800 | 19812399 | high | 16.50 | 0.004534 |
| NAV2 | 89797 | chr11 | 19870800 | 19877999 | high | 18.00 | 0.004519 |
| NBAS | 51594 | chr2 | 15211400 | 15215199 | low | -16.52 | 0.003392 |
| NBAS | 51594 | chr2 | 15458400 | 15460399 | low | -8.69 | 0.003338 |
| NBEA | 26960 | chr13 | 35510600 | 35512799 | high | 5.50 | 0.000759 |
| NBEAL2 | 23218 | chr3 | 46978600 | 46981399 | low | -12.17 | 0.000494 |
| NBR2 | 10230 | chr17 | 43137400 | 43142399 | high | 12.50 | 0.000237 |
| NCAPD3 | 23310 | chr11 | 134220400 | 134225399 | high | 12.50 | 0.000174 |
| NCAPG | 64151 | chr4 | 17809600 | 17811799 | high | 5.50 | 0.003602 |
| NCBP2 | 22916 | chr3 | 196942200 | 196943599 | low | -6.08 | 0.000116 |
| NCBP3 | 55421 | chr17 | 3844200 | 3846399 | low | -9.56 | 0.014417 |
| NCDN | 23154 | chr1 | 35554000 | 35558999 | high | 12.50 | 0.000651 |
| NCDN | 23154 | chr1 | 35562400 | 35568199 | high | 14.50 | 0.000651 |
| NCEH1 | 57552 | chr3 | 172684800 | 172688799 | high | 10.00 | 0.000333 |
| NCEH1 | 57552 | chr3 | 172692600 | 172698999 | high | 16.00 | 0.000333 |
| NCF2 | 4688 | chr1 | 183582200 | 183585399 | high | 8.00 | 0.000026 |
| NCF2 | 4688 | chr1 | 183559800 | 183563199 | high | 8.50 | 0.000026 |
| NCK1 | 4690 | chr3 | 136888600 | 136893599 | high | 12.50 | 0.000034 |
| NCL | 4691 | chr2 | 231462400 | 231466399 | high | 10.00 | 0.000020 |
| NCLN | 56926 | chr19 | 3184400 | 3187599 | high | 8.00 | 0.017877 |
| NCLN | 56926 | chr19 | 3188800 | 3193399 | high | 11.50 | 0.017852 |
| NCOA1 | 8648 | chr2 | 24690600 | 24697999 | high | 18.50 | 0.000350 |
| NCOA5 | 57727 | chr20 | 46078800 | 46081999 | low | -13.91 | 0.001253 |
| NCOA6 | 23054 | chr20 | 34733200 | 34738599 | high | 13.50 | 0.000664 |
| NCOA6 | 23054 | chr20 | 34714200 | 34720599 | high | 16.00 | 0.000664 |
| NCOA6 | 23054 | chr20 | 34821200 | 34828999 | high | 19.50 | 0.000662 |
| NCOA7 | 135112 | chr6 | 125888200 | 125891799 | low | -15.65 | 0.001073 |
| NCOA7 | 135112 | chr6 | 125904800 | 125907599 | low | -12.17 | 0.001073 |
| NCOA7 | 135112 | chr6 | 125881600 | 125882999 | low | -6.08 | 0.001073 |
| NCOR1 | 9611 | chr17 | 16155800 | 16157999 | high | 5.50 | 0.000595 |
| NCOR1 | 9611 | chr17 | 16191800 | 16194599 | high | 7.00 | 0.000594 |
| NCOR1 | 9611 | chr17 | 16037400 | 16040999 | high | 9.00 | 0.000599 |
| NCOR1 | 9611 | chr17 | 16083400 | 16087599 | high | 10.50 | 0.000598 |
| NCOR1 | 9611 | chr17 | 16109000 | 16113399 | high | 11.00 | 0.000597 |
| NCOR1 | 9611 | chr17 | 16169600 | 16175399 | high | 14.50 | 0.000594 |
| NCOR1 | 9611 | chr17 | 16045800 | 16056399 | high | 26.50 | 0.000599 |
| NCOR2 | 9612 | chr12 | 124413400 | 124417399 | high | 10.00 | 0.000077 |
| NCOR2 | 9612 | chr12 | 124407800 | 124412599 | high | 12.00 | 0.000077 |
| NCOR2 | 9612 | chr12 | 124391600 | 124398399 | high | 17.00 | 0.000077 |
| NCOR2 | 9612 | chr12 | 124381800 | 124389799 | high | 20.00 | 0.000077 |
| NCS1 | 23413 | chr9 | 130170000 | 130178399 | high | 21.00 | 0.000180 |
| NDC1 | 55706 | chr1 | 53766800 | 53768799 | high | 5.00 | 0.001036 |
| NDC1 | 55706 | chr1 | 53820600 | 53823199 | high | 6.50 | 0.001035 |
| NDC1 | 55706 | chr1 | 53806800 | 53810199 | high | 8.50 | 0.001035 |
| NDC1 | 55706 | chr1 | 53771400 | 53775399 | high | 10.00 | 0.001036 |
| NDC1 | 55706 | chr1 | 53780400 | 53786799 | high | 16.00 | 0.001036 |
| NDC1 | 55706 | chr1 | 53832400 | 53839599 | high | 18.00 | 0.001035 |
| NDEL1 | 81565 | chr17 | 8435200 | 8437799 | high | 6.50 | 0.009670 |
| NDFIP1 | 80762 | chr5 | 142107600 | 142111399 | high | 9.50 | 0.000568 |
| NDFIP2 | 54602 | chr13 | 79513600 | 79516399 | low | -12.17 | 0.000687 |
| NDOR1 | 27158 | chr9 | 137204600 | 137206799 | high | 5.50 | 0.000198 |
| NDRG3 | 57446 | chr20 | 36729000 | 36732799 | high | 9.50 | 0.001564 |
| NDST1 | 3340 | chr5 | 150542600 | 150546999 | high | 11.00 | 0.000022 |
| NDST1 | 3340 | chr5 | 150551800 | 150557599 | high | 14.50 | 0.000022 |
| NDST3 | 9348 | chr4 | 118155400 | 118157999 | high | 6.50 | 0.000079 |
| NDST3 | 9348 | chr4 | 118237400 | 118242799 | high | 13.50 | 0.000079 |
| NEDD1 | 121441 | chr12 | 96906400 | 96908599 | low | -9.56 | 0.001253 |
| NEDD4 | 4734 | chr15 | 55970800 | 55974199 | high | 8.50 | 0.000085 |
| NEDD4 | 4734 | chr15 | 55988800 | 55994999 | high | 15.50 | 0.000085 |
| NEDD4L | 23327 | chr18 | 58358000 | 58362599 | high | 11.50 | 0.000400 |
| NEDD4L | 23327 | chr18 | 58236600 | 58241599 | high | 12.50 | 0.000401 |
| NEDD4L | 23327 | chr18 | 58211600 | 58217399 | high | 14.50 | 0.000401 |
| NEDD4L | 23327 | chr18 | 58337000 | 58344599 | high | 19.00 | 0.000400 |
| NEDD4L | 23327 | chr18 | 58220400 | 58229599 | high | 23.00 | 0.000401 |
| NEDD8 | 4738 | chr14 | 24230800 | 24235399 | high | 11.50 | 0.000196 |
| NEDD9 | 4739 | chr6 | 11249600 | 11254999 | high | 13.50 | 0.000421 |
| NEFH | 4744 | chr22 | 29479400 | 29482599 | low | -4.98 | 0.001164 |
| NEGR1 | 257194 | chr1 | 71895400 | 71898399 | low | -13.04 | 0.003577 |
| NEIL1 | 79661 | chr15 | 75346000 | 75348799 | high | 7.00 | 0.001057 |
| NEIL2 | 252969 | chr8 | 11775800 | 11779399 | high | 9.00 | 0.002387 |
| NEIL3 | 55247 | chr4 | 177332400 | 177335399 | low | -13.04 | 0.000312 |
| NEK4 | 6787 | chr3 | 52768000 | 52771399 | high | 8.50 | 0.000129 |
| NEK6 | 10783 | chr9 | 124258000 | 124259599 | high | 4.00 | 0.000087 |
| NEK6 | 10783 | chr9 | 124281800 | 124286199 | high | 11.00 | 0.000087 |
| NEK6 | 10783 | chr9 | 124287000 | 124291999 | high | 12.50 | 0.000087 |
| NEK6 | 10783 | chr9 | 124331800 | 124337199 | high | 13.50 | 0.000087 |
| NEK6 | 10783 | chr9 | 124265400 | 124277999 | high | 31.50 | 0.000087 |

| | | | | | | | |
|---|---|---|---|---|---|---|---|
| NEK7 | 140609 | chr1 | 198259400 | 198262399 | high | 7.50 | 0.000709 |
| NEK7 | 140609 | chr1 | 198267000 | 198271599 | high | 11.50 | 0.000709 |
| NEK8 | 284086 | chr17 | 28727800 | 28731199 | high | 8.50 | 0.009889 |
| NEK8 | 284086 | chr17 | 28737400 | 28740999 | high | 9.00 | 0.009886 |
| NEK8 | 284086 | chr17 | 28742400 | 28747999 | high | 14.00 | 0.009884 |
| NEK9 | 91754 | chr14 | 75116200 | 75119999 | high | 9.50 | 0.001221 |
| NELFA | 7469 | chr4 | 1979200 | 1982999 | high | 9.50 | 0.003774 |
| NELFB | 25920 | chr9 | 137254800 | 137256999 | high | 5.50 | 0.000189 |
| NEMF | 9147 | chr14 | 49803800 | 49808199 | high | 11.00 | 0.000184 |
| NEMP1 | 23306 | chr12 | 57077800 | 57079599 | low | -7.82 | 0.000408 |
| NEMP2 | 100131211 | chr2 | 190527600 | 190535799 | low | -35.64 | 0.000053 |
| NEMP2 | 100131211 | chr2 | 190514200 | 190517599 | low | -14.78 | 0.000053 |
| NES | 10763 | chr1 | 156676000 | 156677999 | high | 5.00 | 0.000069 |
| NETO1 | 81832 | chr18 | 72865600 | 72869399 | high | 9.50 | 0.001123 |
| NEU3 | 10825 | chr11 | 75004800 | 75007199 | low | -10.43 | 0.000144 |
| NEURL2 | 140825 | chr20 | 45887000 | 45892599 | high | 14.00 | 0.003069 |
| NEURL4 | 84461 | chr17 | 7312600 | 7316599 | high | 10.00 | 0.011550 |
| NEXN | 91624 | chr1 | 77890800 | 77892999 | high | 5.50 | 0.001176 |
| NEXN | 91624 | chr1 | 77939200 | 77943599 | high | 11.00 | 0.001176 |
| NEXN | 91624 | chr1 | 77923000 | 77927599 | high | 11.50 | 0.001176 |
| NF2 | 4771 | chr22 | 29606400 | 29610399 | high | 10.00 | 0.000161 |
| NF2 | 4771 | chr22 | 29680200 | 29684399 | high | 10.50 | 0.000161 |
| NF2 | 4771 | chr22 | 29614400 | 29618799 | high | 11.00 | 0.000161 |
| NFATC3 | 4775 | chr16 | 68085400 | 68087799 | high | 6.00 | 0.000070 |
| NFATC3 | 4775 | chr16 | 68129400 | 68133799 | high | 11.00 | 0.000070 |
| NFE2L1 | 4779 | chr17 | 48047600 | 48049999 | high | 6.00 | 0.000099 |
| NFE4 | 58160 | chr7 | 102972200 | 102975999 | high | 9.50 | 0.000565 |
| NFIC | 4782 | chr19 | 3400600 | 3406199 | high | 14.00 | 0.001406 |
| NFIC | 4782 | chr19 | 3429800 | 3435999 | high | 15.50 | 0.001394 |
| NFIC | 4782 | chr19 | 3365600 | 3372399 | high | 17.00 | 0.001421 |
| NFIX | 4784 | chr19 | 13015200 | 13018799 | high | 9.00 | 0.000368 |
| NFIX | 4784 | chr19 | 13030400 | 13035199 | high | 12.00 | 0.000367 |
| NFIX | 4784 | chr19 | 13070400 | 13076399 | high | 15.00 | 0.000366 |
| NFIX | 4784 | chr19 | 13022400 | 13029399 | high | 17.50 | 0.000367 |
| NFKB2 | 4791 | chr10 | 102392400 | 102397599 | high | 13.00 | 0.000047 |
| NFKBIE | 4794 | chr6 | 44263200 | 44266799 | low | -15.65 | 0.000108 |
| NFKBIZ | 64332 | chr3 | 101827200 | 101828599 | high | 3.50 | 0.000632 |
| NFRKB | 4798 | chr11 | 129893800 | 129896599 | high | 7.00 | 0.000037 |
| NFU1 | 27247 | chr2 | 69435600 | 69438199 | low | -11.30 | 0.000392 |
| NFX1 | 4799 | chr9 | 33290200 | 33291399 | high | 3.00 | 0.000144 |
| NFX1 | 4799 | chr9 | 33328800 | 33333599 | high | 12.00 | 0.000144 |
| NFX1 | 4799 | chr9 | 33295800 | 33300799 | high | 12.50 | 0.000144 |
| NFXL1 | 152518 | chr4 | 47908200 | 47911399 | low | -13.91 | 0.003184 |
| NFXL1 | 152518 | chr4 | 47913400 | 47915199 | low | -7.82 | 0.003183 |
| NFYA | 4800 | chr6 | 41073400 | 41077999 | low | -19.93 | 0.000117 |
| NFYC | 4802 | chr1 | 40709200 | 40712199 | high | 7.50 | 0.000118 |
| NGRN | 51335 | chr15 | 90261600 | 90266599 | high | 12.50 | 0.000569 |
| NHLRC3 | 387921 | chr13 | 39035800 | 39039199 | high | 8.50 | 0.009938 |
| NHLRC4 | 283948 | chr16 | 567000 | 571799 | high | 12.00 | 0.000123 |
| NHSL1 | 57224 | chr6 | 138475000 | 138479199 | high | 10.50 | 0.000413 |
| NHSL1 | 57224 | chr6 | 138511800 | 138517199 | high | 13.50 | 0.000413 |
| NHSL1 | 57224 | chr6 | 138527800 | 138533399 | high | 14.00 | 0.000413 |
| NID1 | 4811 | chr1 | 236009000 | 236011999 | high | 7.50 | 0.000020 |
| NID1 | 4811 | chr1 | 235981200 | 235984399 | high | 8.00 | 0.000020 |
| NID1 | 4811 | chr1 | 236038800 | 236042799 | high | 10.00 | 0.000020 |
| NID2 | 22795 | chr14 | 52058000 | 52060799 | high | 7.00 | 0.000438 |
| NID2 | 22795 | chr14 | 52052000 | 52055599 | high | 9.00 | 0.000438 |
| NID2 | 22795 | chr14 | 52027200 | 52034799 | high | 19.00 | 0.000438 |
| NIF3L1 | 60491 | chr2 | 200896200 | 200899199 | high | 7.50 | 0.000301 |
| NINJ2 | 4815 | chr12 | 609200 | 612999 | high | 9.50 | 0.007904 |
| NINL | 22981 | chr20 | 25543800 | 25545799 | low | -8.69 | 0.000900 |
| NIP7 | 51388 | chr16 | 69342400 | 69344599 | high | 5.50 | 0.000741 |
| NIPAL3 | 57185 | chr1 | 24434400 | 24436399 | high | 5.00 | 0.002340 |
| NIPBL | 25836 | chr5 | 36996000 | 36998999 | high | 7.50 | 0.000698 |
| NISCH | 11188 | chr3 | 52487400 | 52493599 | high | 15.50 | 0.000213 |
| NIT2 | 56954 | chr3 | 100334000 | 100337199 | low | -13.91 | 0.000568 |
| NKPD1 | 284353 | chr19 | 45151800 | 45154199 | high | 6.00 | 0.006298 |
| NKTR | 4820 | chr3 | 42648200 | 42657199 | high | 22.50 | 0.000113 |
| NLRC5 | 84166 | chr16 | 57036600 | 57044599 | high | 20.00 | 0.001476 |
| NLRP1 | 22861 | chr17 | 5513400 | 5517199 | high | 9.50 | 0.004146 |
| NLRP1 | 22861 | chr17 | 5537000 | 5542199 | high | 13.00 | 0.004129 |
| NMB | 4828 | chr15 | 84654200 | 84656799 | low | -11.30 | 0.000057 |
| NMBR | 4829 | chr6 | 142084600 | 142086399 | low | -7.82 | 0.000034 |
| NME7 | 29922 | chr1 | 169139000 | 169140599 | low | -6.95 | 0.000177 |
| NME9 | 347736 | chr3 | 138274600 | 138279799 | high | 13.00 | 0.002515 |
| NMRK1 | 54981 | chr9 | 75087000 | 75089199 | high | 5.50 | 0.000732 |
| NMT1 | 4836 | chr17 | 45095000 | 45098399 | high | 8.50 | 0.000107 |
| NMT1 | 4836 | chr17 | 45101600 | 45108799 | high | 18.00 | 0.000107 |

| | | | | | | | |
|---|---|---|---|---|---|---|---|
| NMT2 | 9397 | chr10 | 15167400 | 15169399 | low | -8.69 | 0.000620 |
| NMT2 | 9397 | chr10 | 15159000 | 15160999 | low | -8.69 | 0.000620 |
| NOB1 | 28987 | chr16 | 69745600 | 69752599 | high | 17.50 | 0.000416 |
| NOCT | 25819 | chr4 | 139035200 | 139039599 | high | 11.00 | 0.000186 |
| NOL11 | 25926 | chr17 | 67717800 | 67719599 | high | 4.50 | 0.000383 |
| NOL12 | 79159 | chr22 | 37683400 | 37688199 | high | 12.00 | 0.002101 |
| NOL3 | 8996 | chr16 | 67170000 | 67175799 | high | 14.50 | 0.000134 |
| NOL4L | 140688 | chr20 | 32481600 | 32485799 | high | 10.50 | 0.004331 |
| NOL4L | 140688 | chr20 | 32441600 | 32446599 | high | 12.50 | 0.004337 |
| NOL4L | 140688 | chr20 | 32471200 | 32478399 | high | 18.00 | 0.004333 |
| NOL6 | 65083 | chr9 | 33471400 | 33475599 | high | 10.50 | 0.001944 |
| NOL7 | 51406 | chr6 | 13612200 | 13617999 | high | 14.50 | 0.003776 |
| NOL8 | 55035 | chr9 | 92323000 | 92326799 | high | 9.50 | 0.000596 |
| NOP56 | 10528 | chr20 | 2652200 | 2654999 | low | -12.17 | 0.003970 |
| NOP58 | 51602 | chr2 | 202265800 | 202267999 | low | -9.56 | 0.000255 |
| NOP58 | 51602 | chr2 | 202295000 | 202295999 | low | -4.34 | 0.000255 |
| NOP9 | 161424 | chr14 | 24299000 | 24302199 | high | 8.00 | 0.006643 |
| NOTCH2 | 4853 | chr1 | 119917400 | 119921599 | high | 10.50 | 0.000040 |
| NOTCH2 | 4853 | chr1 | 119957000 | 119962599 | high | 14.00 | 0.000040 |
| NOX4 | 50507 | chr11 | 89508600 | 89510999 | low | -10.43 | 0.000564 |
| NOX4 | 50507 | chr11 | 89481800 | 89483599 | low | -7.82 | 0.000564 |
| NPC1 | 4864 | chr18 | 23561600 | 23567599 | high | 15.00 | 0.000206 |
| NPEPPS | 9520 | chr17 | 47621600 | 47623999 | high | 6.00 | 0.000200 |
| NPEPPS | 9520 | chr17 | 47611200 | 47616199 | high | 12.50 | 0.000200 |
| NPHP3 | 27031 | chr3 | 132720400 | 132723399 | low | -13.04 | 0.000204 |
| NPLOC4 | 55666 | chr17 | 81635000 | 81637599 | high | 6.50 | 0.000682 |
| NPLOC4 | 55666 | chr17 | 81606200 | 81610599 | high | 11.00 | 0.000682 |
| NPM1 | 4869 | chr5 | 171387800 | 171389799 | high | 5.00 | 0.000028 |
| NPM2 | 10361 | chr8 | 22023600 | 22027199 | high | 9.00 | 0.000470 |
| NPR3 | 4883 | chr5 | 32774000 | 32777599 | high | 9.00 | 0.000149 |
| NPR3 | 4883 | chr5 | 32785400 | 32789599 | high | 10.50 | 0.000149 |
| NPR3 | 4883 | chr5 | 32747200 | 32752199 | high | 12.50 | 0.000149 |
| NPR3 | 4883 | chr5 | 32778600 | 32783999 | high | 13.50 | 0.000149 |
| NPRL2 | 10641 | chr3 | 50348400 | 50352399 | high | 10.00 | 0.000211 |
| NPRL3 | 8131 | chr16 | 81400 | 86599 | high | 13.00 | 0.011099 |
| NPRL3 | 8131 | chr16 | 113400 | 119599 | high | 15.50 | 0.007967 |
| NPTN | 27020 | chr15 | 73631000 | 73633599 | low | -11.30 | 0.000367 |
| NPTN | 27020 | chr15 | 73583400 | 73585599 | low | -9.56 | 0.000367 |
| NPTX1 | 4884 | chr17 | 80474400 | 80477399 | high | 7.50 | 0.000061 |
| NQO1 | 1728 | chr16 | 69720400 | 69722799 | high | 6.00 | 0.000025 |
| NR1H2 | 7376 | chr19 | 50382800 | 50388399 | high | 14.00 | 0.000146 |
| NR1H3 | 10062 | chr11 | 47256400 | 47260599 | high | 10.50 | 0.000213 |
| NR1H3 | 10062 | chr11 | 47263800 | 47273799 | high | 25.00 | 0.000213 |
| NR2C1 | 7181 | chr12 | 95040600 | 95044599 | high | 10.00 | 0.000076 |
| NR2C2 | 7182 | chr3 | 14947000 | 14950199 | high | 8.00 | 0.000480 |
| NR3C1 | 2908 | chr5 | 143430600 | 143433199 | high | 6.50 | 0.000020 |
| NR3C1 | 2908 | chr5 | 143320200 | 143323199 | high | 7.50 | 0.000020 |
| NR3C1 | 2908 | chr5 | 143410200 | 143413799 | high | 9.00 | 0.000020 |
| NR3C1 | 2908 | chr5 | 143291400 | 143295199 | high | 9.50 | 0.000020 |
| NR3C1 | 2908 | chr5 | 143312800 | 143316999 | high | 10.50 | 0.000020 |
| NR3C1 | 2908 | chr5 | 143334800 | 143339599 | high | 12.00 | 0.000020 |
| NR5A2 | 2494 | chr1 | 200027800 | 200028799 | high | 2.50 | 0.000012 |
| NR5A2 | 2494 | chr1 | 200121800 | 200125199 | high | 8.50 | 0.000012 |
| NR6A1 | 2649 | chr9 | 124660200 | 124664799 | high | 11.50 | 0.000021 |
| NR6A1 | 2649 | chr9 | 124625000 | 124629599 | high | 11.50 | 0.000021 |
| NRAV | 100506668 | chr12 | 120494600 | 120499199 | high | 11.50 | 0.001191 |
| NRBF2 | 29982 | chr10 | 63133400 | 63134999 | high | 4.00 | 0.000475 |
| NRCAM | 4897 | chr7 | 108417400 | 108422599 | high | 13.00 | 0.000045 |
| NRCAM | 4897 | chr7 | 108292400 | 108297599 | high | 13.00 | 0.000045 |
| NRDC | 4898 | chr1 | 51816800 | 51821399 | high | 11.50 | 0.000095 |
| NRDC | 4898 | chr1 | 51833800 | 51843199 | high | 23.50 | 0.000094 |
| NREP | 9315 | chr5 | 111959600 | 111964399 | high | 12.00 | 0.000083 |
| NREP | 9315 | chr5 | 111750800 | 111756199 | high | 13.50 | 0.000083 |
| NRF1 | 4899 | chr7 | 129614200 | 129619999 | high | 14.50 | 0.000038 |
| NRG1 | 3084 | chr8 | 32490600 | 32496999 | low | -16.00 | 0.000095 |
| NRG1 | 3084 | chr8 | 32319000 | 32324399 | low | -13.50 | 0.000095 |
| NRG1 | 3084 | chr8 | 32267800 | 32272999 | low | -13.00 | 0.000096 |
| NRG1 | 3084 | chr8 | 32256600 | 32261199 | low | -11.50 | 0.000096 |
| NRG1 | 3084 | chr8 | 32293600 | 32297999 | low | -11.00 | 0.000095 |
| NRG1 | 3084 | chr8 | 32567000 | 32571199 | low | -10.50 | 0.000095 |
| NRG1 | 3084 | chr8 | 32463200 | 32467199 | low | -10.00 | 0.000095 |
| NRG1 | 3084 | chr8 | 32347400 | 32351199 | low | -9.50 | 0.000095 |
| NRG1 | 3084 | chr8 | 32410800 | 32413599 | low | -7.00 | 0.000095 |
| NRG1 | 3084 | chr8 | 32264200 | 32266799 | low | -6.50 | 0.000096 |
| NRIP1 | 8204 | chr21 | 15061800 | 15065799 | high | 10.00 | 0.000545 |
| NRP1 | 8829 | chr10 | 33326200 | 33328999 | high | 7.00 | 0.000265 |
| NRP1 | 8829 | chr10 | 33252200 | 33255999 | high | 9.50 | 0.000266 |
| NRP1 | 8829 | chr10 | 33264600 | 33269999 | high | 13.50 | 0.000265 |

| | | | | | | | |
|---|---|---|---|---|---|---|---|
| NRP1 | 8829 | chr10 | 33300600 | 33306599 | high | 15.00 | 0.000265 |
| NRP1 | 8829 | chr10 | 33290800 | 33298599 | high | 19.50 | 0.000265 |
| NRP2 | 8828 | chr2 | 205703000 | 205708199 | high | 13.00 | 0.000043 |
| NRSN2 | 80023 | chr20 | 347000 | 348799 | low | -7.82 | 0.000023 |
| NRTN | 4902 | chr19 | 5824400 | 5831199 | high | 17.00 | 0.000842 |
| NSA2 | 10412 | chr5 | 74766000 | 74769799 | low | -16.52 | 0.000139 |
| NSD1 | 64324 | chr5 | 177280400 | 177282199 | high | 4.50 | 0.000363 |
| NSRP1 | 84081 | chr17 | 30123600 | 30126199 | low | -11.30 | 0.002791 |
| NSRP1 | 84081 | chr17 | 30164600 | 30165399 | low | -3.47 | 0.002787 |
| NSUN4 | 387338 | chr1 | 46346600 | 46351599 | high | 12.50 | 0.008357 |
| NT5DC1 | 221294 | chr6 | 116102400 | 116105799 | high | 8.50 | 0.001906 |
| NT5DC1 | 221294 | chr6 | 116233600 | 116237999 | high | 11.00 | 0.001904 |
| NT5DC1 | 221294 | chr6 | 116152000 | 116157799 | high | 14.50 | 0.001905 |
| NT5DC3 | 51559 | chr12 | 103839600 | 103842599 | high | 7.50 | 0.000497 |
| NT5E | 4907 | chr6 | 85474800 | 85483999 | high | 23.00 | 0.000057 |
| NTHL1 | 4913 | chr16 | 2037400 | 2042599 | high | 13.00 | 0.002411 |
| NTM | 50863 | chr11 | 132100000 | 132103599 | high | 9.00 | 0.000385 |
| NTM | 50863 | chr11 | 132090400 | 132094199 | high | 9.50 | 0.000385 |
| NTM | 50863 | chr11 | 131982600 | 131986599 | high | 10.00 | 0.000385 |
| NTM | 50863 | chr11 | 132111200 | 132115799 | high | 11.50 | 0.000385 |
| NTM | 50863 | chr11 | 131942800 | 131947999 | high | 13.00 | 0.000385 |
| NTMT1 | 28989 | chr9 | 129609800 | 129615399 | high | 14.00 | 0.000224 |
| NTMT1 | 28989 | chr9 | 129626200 | 129633199 | high | 17.50 | 0.000224 |
| NTNG1 | 22854 | chr1 | 107140800 | 107143199 | high | 6.00 | 0.000213 |
| NTNG1 | 22854 | chr1 | 107337200 | 107341599 | high | 11.00 | 0.000213 |
| NTPCR | 84284 | chr1 | 232950000 | 232953599 | high | 9.00 | 0.000362 |
| NTRK1 | 4914 | chr1 | 156865400 | 156870599 | high | 13.00 | 0.000031 |
| NTSR2 | 23620 | chr2 | 11667000 | 11670399 | high | 8.50 | 0.002025 |
| NUAK2 | 81788 | chr1 | 205318200 | 205322199 | high | 10.00 | 0.000398 |
| NUAK2 | 81788 | chr1 | 205302400 | 205307199 | high | 12.00 | 0.000398 |
| NUAK2 | 81788 | chr1 | 205308200 | 205316999 | high | 22.00 | 0.000398 |
| NUB1 | 51667 | chr7 | 151341200 | 151343399 | low | -9.56 | 0.000341 |
| NUCB2 | 4925 | chr11 | 17276400 | 17281999 | high | 14.00 | 0.000285 |
| NUDCD3 | 23386 | chr7 | 44458200 | 44461799 | high | 9.00 | 0.000526 |
| NUDCD3 | 23386 | chr7 | 44414800 | 44419199 | high | 11.00 | 0.000527 |
| NUDT1 | 4521 | chr7 | 2240800 | 2243799 | high | 7.50 | 0.002018 |
| NUDT17 | 200035 | chr1 | 145848400 | 145851199 | low | -12.17 | 0.001372 |
| NUDT21 | 11051 | chr16 | 56448200 | 56453599 | high | 13.50 | 0.000196 |
| NUDT3 | 11165 | chr6 | 34317400 | 34321199 | high | 9.50 | 0.000325 |
| NUDT3 | 11165 | chr6 | 34337400 | 34341399 | high | 10.00 | 0.000325 |
| NUDT6 | 11162 | chr4 | 122909800 | 122914799 | high | 12.50 | 0.000091 |
| NUDT9 | 53343 | chr4 | 87421600 | 87424399 | low | -12.17 | 0.000610 |
| NUF2 | 83540 | chr1 | 163320600 | 163324199 | low | -15.65 | 0.000512 |
| NUF2 | 83540 | chr1 | 163327400 | 163329399 | low | -8.69 | 0.000511 |
| NUF2 | 83540 | chr1 | 163336600 | 163337999 | low | -6.08 | 0.000511 |
| NUFIP1 | 26747 | chr13 | 44968000 | 44971599 | high | 9.00 | 0.000595 |
| NUFIP1 | 26747 | chr13 | 44977000 | 44981599 | high | 11.50 | 0.000595 |
| NUFIP1 | 26747 | chr13 | 44950000 | 44955599 | high | 14.00 | 0.000595 |
| NUFIP2 | 57532 | chr17 | 29262000 | 29266999 | high | 12.50 | 0.001966 |
| NUMA1 | 4926 | chr11 | 72075600 | 72085199 | high | 24.00 | 0.000068 |
| NUMB | 8650 | chr14 | 73299200 | 73301599 | high | 6.00 | 0.000118 |
| NUMB | 8650 | chr14 | 73311200 | 73315199 | high | 10.00 | 0.000118 |
| NUMB | 8650 | chr14 | 73321000 | 73327399 | high | 16.00 | 0.000118 |
| NUMBL | 9253 | chr19 | 40687000 | 40691399 | high | 11.00 | 0.000227 |
| NUP107 | 57122 | chr12 | 68726400 | 68728399 | low | -8.69 | 0.000831 |
| NUP153 | 9972 | chr6 | 17655200 | 17657799 | high | 6.50 | 0.000565 |
| NUP153 | 9972 | chr6 | 17670600 | 17676999 | high | 16.00 | 0.000564 |
| NUP155 | 9631 | chr5 | 37327200 | 37329599 | low | -10.43 | 0.000258 |
| NUP188 | 23511 | chr9 | 128989800 | 128992799 | high | 7.50 | 0.000182 |
| NVL | 4931 | chr1 | 224234600 | 224236999 | low | -10.43 | 0.000022 |
| NXF1 | 10482 | chr11 | 62790400 | 62792999 | high | 6.50 | 0.000167 |
| NXN | 64359 | chr17 | 873000 | 876199 | high | 8.00 | 0.008191 |
| NXN | 64359 | chr17 | 889800 | 893599 | high | 9.50 | 0.009737 |
| NXPE3 | 91775 | chr3 | 101778200 | 101780999 | high | 7.00 | 0.000902 |
| NXPE3 | 91775 | chr3 | 101783400 | 101787599 | high | 10.50 | 0.000902 |
| OAS2 | 4939 | chr12 | 112978600 | 112980399 | low | -7.82 | 0.000044 |
| OAZ1 | 4946 | chr19 | 2269200 | 2273999 | low | -20.89 | 0.002180 |
| OAZ3 | 51686 | chr1 | 151763400 | 151763999 | low | -2.60 | 0.000341 |
| OBSL1 | 23363 | chr2 | 219551200 | 219554399 | high | 8.00 | 0.000106 |
| ODC1 | 4953 | chr2 | 10444000 | 10449199 | high | 13.00 | 0.000474 |
| ODF1 | 4956 | chr8 | 102557400 | 102559399 | low | -8.69 | 0.000048 |
| ODF2 | 4957 | chr9 | 128455800 | 128459799 | high | 10.00 | 0.000039 |
| ODF2L | 57489 | chr1 | 86363600 | 86365399 | low | -7.82 | 0.000666 |
| ODF3L1 | 161753 | chr15 | 75723000 | 75725399 | low | -10.43 | 0.002136 |
| OGDH | 4967 | chr7 | 44604600 | 44609199 | high | 11.50 | 0.000111 |
| OGDH | 4967 | chr7 | 44629000 | 44634199 | high | 13.00 | 0.000111 |
| OGDH | 4967 | chr7 | 44644000 | 44649599 | high | 14.00 | 0.000111 |
| OGFR | 11054 | chr20 | 62803000 | 62805599 | high | 6.50 | 0.000176 |

| | | | | | | | |
|---|---|---|---|---|---|---|---|
| OGFRP1 | 388906 | chr22 | 42269000 | 42272799 | high | 9.50 | 0.009201 |
| OIT3 | 170392 | chr10 | 72900200 | 72907399 | high | 18.00 | 0.002337 |
| OLA1 | 29789 | chr2 | 174092800 | 174096799 | high | 10.00 | 0.000171 |
| OLA1 | 29789 | chr2 | 174156800 | 174161799 | high | 12.50 | 0.000171 |
| OLA1 | 29789 | chr2 | 174191000 | 174197399 | high | 16.00 | 0.000171 |
| OLAH | 55301 | chr10 | 15043400 | 15045599 | low | -9.56 | 0.003676 |
| OMD | 4958 | chr9 | 92422600 | 92427799 | high | 13.00 | 0.000054 |
| OPTN | 10133 | chr10 | 13099400 | 13103599 | high | 10.50 | 0.000774 |
| ORC4 | 5000 | chr2 | 147978600 | 147982599 | high | 10.00 | 0.000034 |
| OS9 | 10956 | chr12 | 57698800 | 57701799 | low | -13.04 | 0.000190 |
| OSER1 | 51526 | chr20 | 44209800 | 44211999 | high | 5.50 | 0.001165 |
| OSER1 | 51526 | chr20 | 44201000 | 44203599 | high | 6.50 | 0.001166 |
| OSGIN1 | 29948 | chr16 | 83952600 | 83956999 | high | 11.00 | 0.000357 |
| OSMR | 9180 | chr5 | 38901600 | 38904799 | high | 8.00 | 0.000236 |
| OSMR | 9180 | chr5 | 38919000 | 38922799 | high | 9.50 | 0.000236 |
| OSMR | 9180 | chr5 | 38930200 | 38936599 | high | 16.00 | 0.000236 |
| OSR1 | 130497 | chr2 | 19355800 | 19359799 | high | 10.00 | 0.006742 |
| OSTC | 58505 | chr4 | 108648600 | 108652599 | high | 10.00 | 0.000538 |
| OSTF1 | 26578 | chr9 | 75087000 | 75089199 | high | 5.50 | 0.000354 |
| OSTF1 | 26578 | chr9 | 75124800 | 75128199 | high | 8.50 | 0.000354 |
| OSTF1 | 26578 | chr9 | 75129000 | 75134399 | high | 13.50 | 0.000354 |
| OSTM1 | 28962 | chr6 | 108073600 | 108075199 | high | 4.00 | 0.000268 |
| OTOGL | 283310 | chr12 | 80357400 | 80362199 | high | 12.00 | 0.003526 |
| OTOGL | 283310 | chr12 | 80337400 | 80342599 | high | 13.00 | 0.003527 |
| OTUB1 | 55611 | chr11 | 63985400 | 63987799 | high | 6.00 | 0.000869 |
| OTUD3 | 23252 | chr1 | 19882400 | 19885399 | high | 7.50 | 0.001169 |
| OTUD4 | 54726 | chr4 | 145178200 | 145180199 | low | -8.69 | 0.000377 |
| OTUD7B | 56957 | chr1 | 149941400 | 149945199 | high | 9.50 | 0.000380 |
| OTULIN | 90268 | chr5 | 14674600 | 14679399 | high | 12.00 | 0.006151 |
| OTULIN | 90268 | chr5 | 14661800 | 14667799 | high | 15.00 | 0.006157 |
| OVOL3 | 728361 | chr19 | 36113400 | 36115999 | low | -11.30 | 0.020169 |
| OXA1L | 5018 | chr14 | 22766400 | 22771799 | high | 13.50 | 0.000220 |
| OXCT1 | 5019 | chr5 | 41786400 | 41790199 | high | 9.50 | 0.000120 |
| OXLD1 | 339229 | chr17 | 81666400 | 81668199 | low | -7.82 | 0.004154 |
| P2RX4 | 5025 | chr12 | 121223200 | 121227799 | high | 11.50 | 0.000041 |
| P2RY2 | 5029 | chr11 | 73237600 | 73242799 | high | 13.00 | 0.000069 |
| P2RY6 | 5031 | chr11 | 73268800 | 73273199 | high | 11.00 | 0.000069 |
| P2RY6 | 5031 | chr11 | 73293400 | 73298399 | high | 12.50 | 0.000069 |
| P3H1 | 64175 | chr1 | 42766200 | 42768999 | high | 7.00 | 0.001501 |
| P3H2 | 55214 | chr3 | 190062000 | 190065399 | high | 8.50 | 0.000291 |
| P3H2 | 55214 | chr3 | 190047000 | 190052399 | high | 13.50 | 0.000291 |
| P3H2 | 55214 | chr3 | 190121200 | 190126799 | high | 14.00 | 0.000290 |
| P3H2 | 55214 | chr3 | 189969000 | 189979599 | high | 26.50 | 0.000291 |
| P3H3 | 10536 | chr12 | 6835800 | 6840599 | high | 12.00 | 0.001541 |
| P3H3 | 10536 | chr12 | 6821000 | 6830799 | high | 24.50 | 0.001545 |
| P3H4 | 10609 | chr17 | 41811200 | 41814399 | low | -13.91 | 0.000254 |
| P4HA1 | 5033 | chr10 | 73010000 | 73013599 | high | 9.00 | 0.000069 |
| P4HA1 | 5033 | chr10 | 73092600 | 73098199 | high | 14.00 | 0.000069 |
| P4HA2 | 8974 | chr5 | 132207200 | 132212399 | high | 13.00 | 0.000068 |
| P4HA2 | 8974 | chr5 | 132191000 | 132198399 | high | 18.50 | 0.000068 |
| P4HA3 | 283208 | chr11 | 74298600 | 74304799 | high | 15.50 | 0.003812 |
| P4HA3 | 283208 | chr11 | 74273400 | 74280799 | high | 18.50 | 0.003813 |
| P4HB | 5034 | chr17 | 81843000 | 81848999 | high | 15.00 | 0.000062 |
| P4HTM | 54681 | chr3 | 49006400 | 49008799 | high | 6.00 | 0.001116 |
| P4HTM | 54681 | chr3 | 48989800 | 48992799 | high | 7.50 | 0.001116 |
| PA2G4 | 5036 | chr12 | 56103800 | 56105799 | low | -8.69 | 0.000090 |
| PABPC1 | 26986 | chr8 | 100719000 | 100723399 | high | 11.00 | 0.000268 |
| PACS2 | 23241 | chr14 | 105299400 | 105302999 | high | 9.00 | 0.000221 |
| PACS2 | 23241 | chr14 | 105311600 | 105315799 | high | 10.50 | 0.000221 |
| PAIP1 | 10605 | chr5 | 43527600 | 43533999 | high | 16.00 | 0.000244 |
| PAK1 | 5058 | chr11 | 77377600 | 77380599 | high | 7.50 | 0.000065 |
| PAK1 | 5058 | chr11 | 77461600 | 77465399 | high | 9.50 | 0.000065 |
| PAK1 | 5058 | chr11 | 77369800 | 77376799 | high | 17.50 | 0.000065 |
| PAK1 | 5058 | chr11 | 77348600 | 77356399 | high | 19.50 | 0.000065 |
| PAK1IP1 | 55003 | chr6 | 10693600 | 10695599 | low | -8.69 | 0.005144 |
| PAK4 | 10298 | chr19 | 39154200 | 39159799 | high | 14.00 | 0.000263 |
| PAK5 | 57144 | chr20 | 9605400 | 9612199 | high | 17.00 | 0.005949 |
| PAK6 | 56924 | chr15 | 40269200 | 40272799 | high | 9.00 | 0.001414 |
| PAK6 | 56924 | chr15 | 40273600 | 40278199 | high | 11.50 | 0.001413 |
| PAK6 | 56924 | chr15 | 40236800 | 40241799 | high | 12.50 | 0.001415 |
| PALLD | 23022 | chr4 | 168718000 | 168721399 | high | 8.50 | 0.000136 |
| PALLD | 23022 | chr4 | 168555200 | 168558599 | high | 8.50 | 0.000137 |
| PALLD | 23022 | chr4 | 168846000 | 168849799 | high | 9.50 | 0.000136 |
| PALLD | 23022 | chr4 | 168875200 | 168879199 | high | 10.00 | 0.000136 |
| PALLD | 23022 | chr4 | 168868400 | 168872799 | high | 11.00 | 0.000136 |
| PALLD | 23022 | chr4 | 168742200 | 168747199 | high | 12.50 | 0.000136 |
| PALLD | 23022 | chr4 | 168562600 | 168567599 | high | 12.50 | 0.000137 |
| PALLD | 23022 | chr4 | 168907600 | 168912799 | high | 13.00 | 0.000136 |

| | | | | | | | |
|---|---|---|---|---|---|---|---|
| PALLD | 23022 | chr4 | 168769200 | 168775199 | high | 15.00 | 0.000136 |
| PALM2 | 114299 | chr9 | 109913400 | 109917799 | high | 11.00 | 0.001040 |
| PAMR1 | 25891 | chr11 | 35523600 | 35527199 | high | 9.00 | 0.000729 |
| PAMR1 | 25891 | chr11 | 35483000 | 35487999 | high | 12.50 | 0.000730 |
| PANK2 | 80025 | chr20 | 3894600 | 3897799 | high | 8.00 | 0.002283 |
| PANK2 | 80025 | chr20 | 3917400 | 3920799 | high | 8.50 | 0.000206 |
| PANK2 | 80025 | chr20 | 3905800 | 3909199 | high | 8.50 | 0.002277 |
| PANK3 | 79646 | chr5 | 168576200 | 168579999 | high | 9.50 | 0.000472 |
| PANX1 | 24145 | chr11 | 94162600 | 94165399 | high | 7.00 | 0.000256 |
| PANX1 | 24145 | chr11 | 94127000 | 94131199 | high | 10.50 | 0.000257 |
| PANX1 | 24145 | chr11 | 94171400 | 94176999 | high | 14.00 | 0.000256 |
| PAPD4 | 167153 | chr5 | 79610200 | 79615399 | high | 13.00 | 0.002100 |
| PAPD5 | 64282 | chr16 | 50151200 | 50155599 | high | 11.00 | 0.001282 |
| PAPD5 | 64282 | chr16 | 50160200 | 50166399 | high | 15.50 | 0.001282 |
| PAPD7 | 11044 | chr5 | 6729400 | 6736999 | high | 19.00 | 0.001641 |
| PAPLN | 89932 | chr14 | 73243200 | 73246999 | high | 9.50 | 0.001228 |
| PAPLN | 89932 | chr14 | 73235800 | 73239599 | high | 9.50 | 0.001228 |
| PAPLN | 89932 | chr14 | 73267800 | 73272199 | high | 11.00 | 0.001227 |
| PAPPA | 5069 | chr9 | 116153800 | 116156799 | high | 7.50 | 0.000044 |
| PAPPA | 5069 | chr9 | 116159400 | 116162799 | high | 8.50 | 0.000044 |
| PAPPA | 5069 | chr9 | 116193000 | 116199999 | high | 17.50 | 0.000044 |
| PAPSS1 | 9061 | chr4 | 107630000 | 107631999 | low | -8.69 | 0.000084 |
| PAPSS1 | 9061 | chr4 | 107719400 | 107720599 | low | -5.21 | 0.000084 |
| PAQR4 | 124222 | chr16 | 2967800 | 2970799 | high | 7.50 | 0.004651 |
| PAQR5 | 54852 | chr15 | 69378600 | 69382999 | high | 11.00 | 0.000791 |
| PAQR5 | 54852 | chr15 | 69305200 | 69309999 | high | 12.00 | 0.000791 |
| PAQR5 | 54852 | chr15 | 69342000 | 69347199 | high | 13.00 | 0.000791 |
| PAQR8 | 85315 | chr6 | 52361600 | 52364199 | high | 6.50 | 0.001629 |
| PARD3 | 56288 | chr10 | 34181800 | 34183599 | high | 4.50 | 0.001647 |
| PARD3 | 56288 | chr10 | 34121000 | 34123799 | high | 7.00 | 0.001650 |
| PARD3 | 56288 | chr10 | 34241600 | 34245999 | high | 11.00 | 0.001644 |
| PARGP1 | 728407 | chr10 | 45971600 | 45972999 | low | -6.08 | 0.015845 |
| PARK7 | 11315 | chr1 | 7965200 | 7969199 | high | 10.00 | 0.001421 |
| PARL | 55486 | chr3 | 183844200 | 183850599 | high | 16.00 | 0.000302 |
| PARP15 | 165631 | chr3 | 122630200 | 122635599 | high | 13.50 | 0.001351 |
| PARP3 | 10039 | chr3 | 51941200 | 51945199 | high | 10.00 | 0.000193 |
| PARP4 | 143 | chr13 | 24511200 | 24514199 | high | 7.50 | 0.000006 |
| PARP8 | 79668 | chr5 | 50664200 | 50668399 | high | 10.50 | 0.001572 |
| PARP9 | 83666 | chr3 | 122563000 | 122568799 | high | 14.50 | 0.000683 |
| PARPBP | 55010 | chr12 | 102119000 | 102121999 | low | -13.04 | 0.000539 |
| PARVA | 55742 | chr11 | 12433000 | 12435599 | high | 6.50 | 0.004483 |
| PARVA | 55742 | chr11 | 12420600 | 12424799 | high | 10.50 | 0.004488 |
| PARVA | 55742 | chr11 | 12393800 | 12398399 | high | 11.50 | 0.004498 |
| PARVA | 55742 | chr11 | 12492200 | 12497599 | high | 13.50 | 0.004462 |
| PARVA | 55742 | chr11 | 12523800 | 12529599 | high | 14.50 | 0.004451 |
| PARVA | 55742 | chr11 | 12377000 | 12382799 | high | 14.50 | 0.004504 |
| PARVA | 55742 | chr11 | 12399600 | 12406399 | high | 17.00 | 0.004495 |
| PASK | 23178 | chr2 | 241148800 | 241151599 | high | 7.00 | 0.000096 |
| PAWR | 5074 | chr12 | 79615600 | 79619999 | high | 11.00 | 0.000064 |
| PAWR | 5074 | chr12 | 79598000 | 79606599 | high | 21.50 | 0.000064 |
| PBRM1 | 55193 | chr3 | 52609400 | 52612799 | high | 8.50 | 0.001049 |
| PBRM1 | 55193 | chr3 | 52595400 | 52601799 | high | 16.00 | 0.001049 |
| PBX3 | 5090 | chr9 | 125844600 | 125849599 | low | -3.98 | 0.001698 |
| PBX4 | 80714 | chr19 | 19574600 | 19579399 | high | 12.00 | 0.004123 |
| PCAT1 | 100750225 | chr8 | 127012200 | 127015999 | high | 9.50 | 0.002327 |
| PCBD2 | 84105 | chr5 | 134925200 | 134927199 | high | 5.00 | 0.000623 |
| PCBP2 | 5094 | chr12 | 53465800 | 53471399 | high | 14.00 | 0.000095 |
| PCBP3 | 54039 | chr21 | 45863800 | 45867399 | high | 9.00 | 0.001178 |
| PCBP3 | 54039 | chr21 | 45883000 | 45888199 | high | 13.00 | 0.001178 |
| PCBP4 | 57060 | chr3 | 51965200 | 51967799 | high | 6.50 | 0.001098 |
| PCBP4 | 57060 | chr3 | 51954800 | 51963199 | high | 21.00 | 0.001098 |
| PCCB | 5096 | chr3 | 136276200 | 136277999 | low | -7.82 | 0.000037 |
| PCDH9 | 5101 | chr13 | 67188000 | 67190999 | high | 7.50 | 0.000076 |
| PCDH9 | 5101 | chr13 | 66897400 | 66900599 | high | 8.00 | 0.000076 |
| PCDH9 | 5101 | chr13 | 67209400 | 67212999 | high | 9.00 | 0.000076 |
| PCDH9 | 5101 | chr13 | 66613800 | 66618399 | high | 11.50 | 0.000077 |
| PCDH9 | 5101 | chr13 | 67222600 | 67227399 | high | 12.00 | 0.000076 |
| PCDH9 | 5101 | chr13 | 66659200 | 66664599 | high | 13.50 | 0.000077 |
| PCED1A | 64773 | chr20 | 2840600 | 2843399 | high | 7.00 | 0.002534 |
| PCED1B | 91523 | chr12 | 47163800 | 47168199 | high | 11.00 | 0.001941 |
| PCED1B | 91523 | chr12 | 47079000 | 47083599 | high | 11.50 | 0.001944 |
| PCED1B | 91523 | chr12 | 47188400 | 47193999 | high | 14.00 | 0.001940 |
| PCED1B | 91523 | chr12 | 47091400 | 47097199 | high | 14.50 | 0.001944 |
| PCGF5 | 84333 | chr10 | 91234000 | 91238999 | high | 12.50 | 0.000924 |
| PCGF5 | 84333 | chr10 | 91275800 | 91280999 | high | 13.00 | 0.000924 |
| PCID2 | 55795 | chr13 | 113192800 | 113197199 | high | 11.00 | 0.000493 |
| PCID2 | 55795 | chr13 | 113183000 | 113187599 | high | 11.50 | 0.000493 |
| PCIF1 | 63935 | chr20 | 45933400 | 45938199 | high | 12.00 | 0.001392 |

| | | | | | | | |
|---|---|---|---|---|---|---|---|
| PCLO | 27445 | chr7 | 82777800 | 82780399 | high | 6.50 | 0.000332 |
| PCMT1 | 5110 | chr6 | 149749400 | 149752999 | high | 9.00 | 0.000034 |
| PCMTD1 | 115294 | chr8 | 51896800 | 51900199 | low | -14.78 | 0.002222 |
| PCNT | 5116 | chr21 | 46322400 | 46324399 | low | -8.69 | 0.000110 |
| PCNX1 | 22990 | chr14 | 71092200 | 71095999 | low | -16.52 | 0.000323 |
| PCNX1 | 22990 | chr14 | 71100200 | 71102999 | low | -12.17 | 0.000323 |
| PCNX1 | 22990 | chr14 | 71012400 | 71014399 | low | -8.69 | 0.000324 |
| PCNX2 | 80003 | chr1 | 233165400 | 233168599 | high | 8.00 | 0.000343 |
| PCNX3 | 399909 | chr11 | 65611200 | 65616799 | high | 14.00 | 0.006095 |
| PCNX3 | 399909 | chr11 | 65629400 | 65637199 | high | 19.50 | 0.006093 |
| PCSK1 | 5122 | chr5 | 96430400 | 96435999 | high | 14.00 | 0.000053 |
| PCSK4 | 54760 | chr19 | 1489400 | 1493199 | high | 9.50 | 0.004085 |
| PCTP | 58488 | chr17 | 55749200 | 55753599 | high | 11.00 | 0.001049 |
| PCYT1A | 5130 | chr3 | 196266600 | 196269799 | high | 8.00 | 0.000026 |
| PDAP1 | 11333 | chr7 | 99404400 | 99410199 | high | 14.50 | 0.000114 |
| PDCD11 | 22984 | chr10 | 103394800 | 103396799 | high | 5.00 | 0.000222 |
| PDCD6 | 10016 | chr5 | 281400 | 287399 | high | 15.00 | 0.003955 |
| PDCD6 | 10016 | chr5 | 313600 | 319999 | high | 16.00 | 0.003549 |
| PDCL | 5082 | chr9 | 122827800 | 122828999 | low | -5.21 | 0.000041 |
| PDDC1 | 347862 | chr11 | 775200 | 778599 | high | 8.50 | 0.009288 |
| PDE1A | 5136 | chr2 | 182235600 | 182236999 | low | -6.08 | 0.000028 |
| PDE2A | 5138 | chr11 | 72640800 | 72644599 | high | 9.50 | 0.000071 |
| PDE3A | 5139 | chr12 | 20494200 | 20497599 | high | 8.50 | 0.000251 |
| PDE3A | 5139 | chr12 | 20501800 | 20505999 | high | 10.50 | 0.000251 |
| PDE3A | 5139 | chr12 | 20512400 | 20516799 | high | 11.00 | 0.000251 |
| PDE4A | 5141 | chr19 | 10429800 | 10433399 | high | 9.00 | 0.000493 |
| PDE4B | 5142 | chr1 | 66072400 | 66076199 | low | -16.52 | 0.000078 |
| PDE4B | 5142 | chr1 | 66205600 | 66208199 | low | -11.30 | 0.000078 |
| PDE4B | 5142 | chr1 | 66191000 | 66192799 | low | -7.82 | 0.000078 |
| PDE5A | 8654 | chr4 | 119494200 | 119497599 | high | 8.50 | 0.000072 |
| PDE5A | 8654 | chr4 | 119554600 | 119558599 | high | 10.00 | 0.000072 |
| PDE5A | 8654 | chr4 | 119515200 | 119520799 | high | 14.00 | 0.000072 |
| PDE6D | 5147 | chr2 | 231778400 | 231787999 | low | -41.73 | 0.000022 |
| PDE7B | 27115 | chr6 | 136023400 | 136026999 | high | 9.00 | 0.000199 |
| PDE7B | 27115 | chr6 | 136070400 | 136074399 | high | 10.00 | 0.000199 |
| PDE7B | 27115 | chr6 | 136031800 | 136035799 | high | 10.00 | 0.000199 |
| PDE7B | 27115 | chr6 | 135935800 | 135939799 | high | 10.00 | 0.000199 |
| PDE7B | 27115 | chr6 | 135973600 | 135978199 | high | 11.50 | 0.000199 |
| PDE7B | 27115 | chr6 | 135943400 | 135948199 | high | 12.00 | 0.000199 |
| PDE7B | 27115 | chr6 | 135982000 | 135988599 | high | 16.50 | 0.000199 |
| PDE8A | 5151 | chr15 | 85028400 | 85031999 | high | 9.00 | 0.000061 |
| PDE8A | 5151 | chr15 | 85089800 | 85094199 | high | 11.00 | 0.000061 |
| PDE8A | 5151 | chr15 | 85000800 | 85005399 | high | 11.50 | 0.000061 |
| PDE8A | 5151 | chr15 | 85040200 | 85045799 | high | 14.00 | 0.000061 |
| PDE8A | 5151 | chr15 | 85076400 | 85082599 | high | 15.50 | 0.000061 |
| PDF | 64146 | chr16 | 69326400 | 69334399 | high | 20.00 | 0.000925 |
| PDGFC | 56034 | chr4 | 156831800 | 156834799 | high | 7.50 | 0.000357 |
| PDGFC | 56034 | chr4 | 156804400 | 156808199 | high | 9.50 | 0.000357 |
| PDGFC | 56034 | chr4 | 156767200 | 156770999 | high | 9.50 | 0.000357 |
| PDGFC | 56034 | chr4 | 156863200 | 156867599 | high | 11.00 | 0.000357 |
| PDGFC | 56034 | chr4 | 156813200 | 156818199 | high | 12.50 | 0.000357 |
| PDGFC | 56034 | chr4 | 156960400 | 156965799 | high | 13.50 | 0.000357 |
| PDGFC | 56034 | chr4 | 156846200 | 156851799 | high | 14.00 | 0.000357 |
| PDGFC | 56034 | chr4 | 156934800 | 156941799 | high | 17.50 | 0.000357 |
| PDHX | 8050 | chr11 | 34937200 | 34939199 | high | 5.00 | 0.000230 |
| PDHX | 8050 | chr11 | 34940800 | 34944199 | high | 8.50 | 0.000230 |
| PDHX | 8050 | chr11 | 34953600 | 34958199 | high | 11.50 | 0.000230 |
| PDHX | 8050 | chr11 | 34966600 | 34973599 | high | 17.50 | 0.000230 |
| PDIA5 | 10954 | chr3 | 123085800 | 123090399 | high | 11.50 | 0.000089 |
| PDIA5 | 10954 | chr3 | 123065600 | 123071799 | high | 15.50 | 0.000089 |
| PDIA6 | 10130 | chr2 | 10808800 | 10814599 | high | 14.50 | 0.000937 |
| PDK1 | 5163 | chr2 | 172580800 | 172585199 | high | 11.00 | 0.000030 |
| PDK1 | 5163 | chr2 | 172610000 | 172615799 | high | 14.50 | 0.000030 |
| PDK2 | 5164 | chr17 | 50094200 | 50096799 | high | 6.50 | 0.000103 |
| PDLIM1 | 9124 | chr10 | 95286400 | 95292199 | high | 14.50 | 0.000096 |
| PDLIM1 | 9124 | chr10 | 95279000 | 95285399 | high | 16.00 | 0.000096 |
| PDLIM2 | 64236 | chr8 | 22595000 | 22600199 | high | 13.00 | 0.002843 |
| PDLIM2 | 64236 | chr8 | 22576800 | 22582399 | high | 14.00 | 0.002845 |
| PDLIM4 | 8572 | chr5 | 132257200 | 132260799 | low | -15.65 | 0.000065 |
| PDLIM7 | 9260 | chr5 | 177496400 | 177497599 | low | -5.21 | 0.000052 |
| PDP2 | 57546 | chr16 | 66880000 | 66882999 | high | 7.50 | 0.000860 |
| PDP2 | 57546 | chr16 | 66885000 | 66889799 | high | 12.00 | 0.000860 |
| PDS5B | 23047 | chr13 | 32730800 | 32734799 | high | 10.00 | 0.000704 |
| PDXK | 8566 | chr21 | 43732000 | 43736799 | high | 12.00 | 0.000196 |
| PDXK | 8566 | chr21 | 43725600 | 43730799 | high | 13.00 | 0.000196 |
| PDXP | 57026 | chr22 | 37657400 | 37660399 | high | 7.50 | 0.001514 |
| PDXP | 57026 | chr22 | 37661200 | 37668799 | high | 19.00 | 0.001514 |
| PDZD7 | 79955 | chr10 | 101029000 | 101030799 | low | -7.82 | 0.000791 |

| | | | | | | | |
|---|---|---|---|---|---|---|---|
| PDZD8 | 118987 | chr10 | 117370600 | 117376599 | high | 15.00 | 0.001014 |
| PDZK1 | 5174 | chr1 | 145687000 | 145692399 | high | 13.50 | 0.000036 |
| PDZRN3 | 23024 | chr3 | 73613000 | 73615799 | high | 7.00 | 0.000313 |
| PDZRN3 | 23024 | chr3 | 73506400 | 73511399 | high | 12.50 | 0.000313 |
| PEA15 | 8682 | chr1 | 160205400 | 160207999 | high | 6.50 | 0.000054 |
| PEAK1 | 79834 | chr15 | 77416800 | 77420399 | high | 9.00 | 0.001031 |
| PEAK1 | 79834 | chr15 | 77251000 | 77254599 | high | 9.00 | 0.001033 |
| PEAK1 | 79834 | chr15 | 77217400 | 77221399 | high | 10.00 | 0.001034 |
| PEAK1 | 79834 | chr15 | 77304000 | 77308799 | high | 12.00 | 0.001033 |
| PEAK1 | 79834 | chr15 | 77118600 | 77124399 | high | 14.50 | 0.001035 |
| PEBP1 | 5037 | chr12 | 118134800 | 118137999 | high | 8.00 | 0.000043 |
| PEF1 | 553115 | chr1 | 31644000 | 31645799 | high | 4.50 | 0.017479 |
| PEG10 | 23089 | chr7 | 94650600 | 94658799 | high | 20.50 | 0.000244 |
| PELI1 | 57162 | chr2 | 64092600 | 64097199 | high | 11.50 | 0.000892 |
| PELI2 | 57161 | chr14 | 56117000 | 56120599 | high | 9.00 | 0.001019 |
| PELI2 | 57161 | chr14 | 56192200 | 56196599 | high | 11.00 | 0.001017 |
| PELI2 | 57161 | chr14 | 56127000 | 56131399 | high | 11.00 | 0.001018 |
| PELI2 | 57161 | chr14 | 56292400 | 56297599 | high | 13.00 | 0.001015 |
| PELI2 | 57161 | chr14 | 56154600 | 56160599 | high | 15.00 | 0.001018 |
| PELI3 | 246330 | chr11 | 66466200 | 66468999 | high | 7.00 | 0.003706 |
| PELO | 53918 | chr5 | 52798400 | 52805399 | high | 17.50 | 0.001021 |
| PEMT | 10400 | chr17 | 17558800 | 17561799 | high | 7.50 | 0.000592 |
| PER1 | 5187 | chr17 | 8151400 | 8152799 | low | -6.08 | 0.000636 |
| PERP | 64065 | chr6 | 138093400 | 138099999 | high | 16.50 | 0.000464 |
| PES1 | 23481 | chr22 | 30589000 | 30591599 | low | -11.30 | 0.000768 |
| PEX1 | 5189 | chr7 | 92487800 | 92489599 | high | 4.50 | 0.000056 |
| PEX13 | 5194 | chr2 | 61016200 | 61018799 | low | -11.30 | 0.000085 |
| PEX14 | 5195 | chr1 | 10489600 | 10496799 | high | 18.00 | 0.000495 |
| PEX16 | 9409 | chr11 | 45916800 | 45918999 | high | 5.50 | 0.000205 |
| PEX26 | 55670 | chr22 | 18077000 | 18081599 | high | 11.50 | 0.003080 |
| PEX3 | 8504 | chr6 | 143455800 | 143459599 | high | 9.50 | 0.000059 |
| PEX6 | 5190 | chr6 | 42977000 | 42979799 | high | 7.00 | 0.000121 |
| PEX7 | 5191 | chr6 | 136832400 | 136834799 | low | -10.43 | 0.000038 |
| PFAS | 5198 | chr17 | 8247600 | 8251599 | high | 10.00 | 0.000630 |
| PFDN1 | 5201 | chr5 | 140247400 | 140250799 | high | 8.50 | 0.000037 |
| PFKFB3 | 5209 | chr10 | 6201800 | 6203799 | high | 5.00 | 0.000840 |
| PFKFB3 | 5209 | chr10 | 6143400 | 6149199 | high | 14.50 | 0.000848 |
| PFKFB4 | 5210 | chr3 | 48555400 | 48557399 | low | -8.69 | 0.000107 |
| PFKL | 5211 | chr21 | 44298800 | 44302999 | high | 10.50 | 0.000118 |
| PFKM | 5213 | chr12 | 48105000 | 48107799 | high | 7.00 | 0.000108 |
| PFKM | 5213 | chr12 | 48115600 | 48120199 | high | 11.50 | 0.000108 |
| PFKP | 5214 | chr10 | 3067600 | 3070799 | high | 8.00 | 0.001700 |
| PFN2 | 5217 | chr3 | 149969400 | 149971999 | low | -11.30 | 0.000035 |
| PFN4 | 375189 | chr2 | 24122200 | 24123799 | low | -6.95 | 0.015554 |
| PGAM5 | 192111 | chr12 | 132710200 | 132711599 | high | 3.50 | 0.001448 |
| PGD | 5226 | chr1 | 10411600 | 10416599 | high | 12.50 | 0.000502 |
| PGGT1B | 5229 | chr5 | 115260600 | 115263199 | high | 6.50 | 0.000045 |
| PGM1 | 5236 | chr1 | 63592200 | 63594999 | high | 7.00 | 0.000082 |
| PGM2L1 | 283209 | chr11 | 74341600 | 74350799 | high | 23.00 | 0.003810 |
| PGM3 | 5238 | chr6 | 83190200 | 83195199 | high | 12.50 | 0.000063 |
| PGPEP1 | 54858 | chr19 | 18338000 | 18348999 | high | 27.50 | 0.002991 |
| PGS1 | 9489 | chr17 | 78377000 | 78380799 | high | 9.50 | 0.000121 |
| PGS1 | 9489 | chr17 | 78392800 | 78401599 | high | 22.00 | 0.000121 |
| PHAX | 51808 | chr5 | 126600200 | 126602399 | low | -9.56 | 0.000409 |
| PHC2 | 1912 | chr1 | 33348000 | 33352199 | high | 10.50 | 0.000057 |
| PHC2 | 1912 | chr1 | 33340000 | 33346999 | high | 17.50 | 0.000057 |
| PHF10 | 55274 | chr6 | 169721200 | 169724799 | high | 9.00 | 0.000326 |
| PHF13 | 148479 | chr1 | 6612800 | 6615599 | low | -12.17 | 0.002495 |
| PHF14 | 9678 | chr7 | 11139200 | 11144399 | high | 13.00 | 0.000869 |
| PHF20 | 51230 | chr20 | 35915800 | 35918399 | high | 6.50 | 0.001426 |
| PHF20 | 51230 | chr20 | 35906400 | 35910399 | high | 10.00 | 0.001427 |
| PHF20 | 51230 | chr20 | 35944000 | 35948999 | high | 12.50 | 0.001425 |
| PHF20 | 51230 | chr20 | 35924800 | 35930599 | high | 14.50 | 0.001426 |
| PHF23 | 79142 | chr17 | 7236400 | 7239199 | low | -12.17 | 0.010937 |
| PHF3 | 23469 | chr6 | 63636000 | 63637799 | low | -7.82 | 0.000369 |
| PHF5A | 84844 | chr22 | 41466800 | 41469799 | high | 7.50 | 0.002046 |
| PHIP | 55023 | chr6 | 79077000 | 79079399 | low | -10.43 | 0.000696 |
| PHIP | 55023 | chr6 | 79018000 | 79019799 | low | -7.82 | 0.000696 |
| PHIP | 55023 | chr6 | 79060400 | 79061599 | low | -5.21 | 0.000696 |
| PHKB | 5257 | chr16 | 47567800 | 47571599 | low | -16.52 | 0.000111 |
| PHKB | 5257 | chr16 | 47460400 | 47461599 | low | -5.21 | 0.000111 |
| PHKG2 | 5261 | chr16 | 30759400 | 30764399 | high | 12.50 | 0.000171 |
| PHLDA1 | 22822 | chr12 | 76024600 | 76026999 | high | 6.00 | 0.000300 |
| PHLDB1 | 23187 | chr11 | 118606800 | 118609399 | high | 6.50 | 0.000195 |
| PHLDB1 | 23187 | chr11 | 118636600 | 118640799 | high | 10.50 | 0.000195 |
| PHLDB1 | 23187 | chr11 | 118627600 | 118632399 | high | 12.00 | 0.000195 |
| PHLDB2 | 90102 | chr3 | 111765400 | 111768999 | high | 9.00 | 0.000806 |
| PHLDB2 | 90102 | chr3 | 111943200 | 111947199 | high | 10.00 | 0.000805 |

| | | | | | | | |
|---|---|---|---|---|---|---|---|
| PHLDB2 | 90102 | chr3 | 111931800 | 111935999 | high | 10.50 | 0.000805 |
| PHLDB2 | 90102 | chr3 | 111937800 | 111942399 | high | 11.50 | 0.000805 |
| PHLDB2 | 90102 | chr3 | 111732600 | 111739999 | high | 18.50 | 0.000806 |
| PHLDB3 | 653583 | chr19 | 43474000 | 43480199 | high | 15.50 | 0.015034 |
| PHLPP1 | 23239 | chr18 | 62727800 | 62731599 | high | 9.50 | 0.000370 |
| PHLPP1 | 23239 | chr18 | 62714000 | 62717799 | high | 9.50 | 0.000371 |
| PHLPP2 | 23035 | chr16 | 71681600 | 71687199 | high | 14.00 | 0.000321 |
| PHPT1 | 29085 | chr9 | 136847800 | 136850599 | low | -12.17 | 0.000213 |
| PHTF1 | 10745 | chr1 | 113742800 | 113746999 | high | 10.50 | 0.000094 |
| PHTF2 | 57157 | chr7 | 77831000 | 77835399 | high | 11.00 | 0.000734 |
| PHTF2 | 57157 | chr7 | 77794000 | 77799199 | high | 13.00 | 0.000735 |
| PHYHIP | 9796 | chr8 | 22226200 | 22230599 | high | 11.00 | 0.000441 |
| PI16 | 221476 | chr6 | 36951600 | 36956599 | low | -21.08 | 0.005994 |
| PIANP | 196500 | chr12 | 6699400 | 6701599 | low | -9.56 | 0.003259 |
| PIAS1 | 8554 | chr15 | 68086600 | 68091399 | high | 12.00 | 0.000126 |
| PIAS1 | 8554 | chr15 | 68189600 | 68194799 | high | 13.00 | 0.000125 |
| PIAS1 | 8554 | chr15 | 68053400 | 68058799 | high | 13.50 | 0.000126 |
| PICALM | 8301 | chr11 | 85980200 | 85982999 | low | -12.17 | 0.000097 |
| PICALM | 8301 | chr11 | 85988000 | 85989799 | low | -7.82 | 0.000097 |
| PIDD1 | 55367 | chr11 | 802000 | 806599 | high | 11.50 | 0.000998 |
| PIEZO1 | 9780 | chr16 | 88753400 | 88756799 | high | 8.50 | 0.000110 |
| PIGB | 9488 | chr15 | 55328800 | 55331599 | low | -12.17 | 0.000171 |
| PIGB | 9488 | chr15 | 55349800 | 55351199 | low | -6.08 | 0.000171 |
| PIGF | 5281 | chr2 | 46615600 | 46617799 | high | 5.50 | 0.000113 |
| PIGF | 5281 | chr2 | 46589200 | 46592999 | high | 9.50 | 0.000113 |
| PIGL | 9487 | chr17 | 16230200 | 16232799 | high | 6.50 | 0.000585 |
| PIGL | 9487 | chr17 | 16292200 | 16297999 | high | 14.50 | 0.000582 |
| PIGN | 23556 | chr18 | 62088600 | 62092399 | low | -16.52 | 0.000379 |
| PIGN | 23556 | chr18 | 62104400 | 62106799 | low | -10.43 | 0.000379 |
| PIGN | 23556 | chr18 | 62186600 | 62188799 | low | -9.56 | 0.000379 |
| PIGO | 84720 | chr9 | 35095400 | 35096999 | low | -6.95 | 0.002414 |
| PIGQ | 9091 | chr16 | 567000 | 571799 | high | 12.00 | 0.016034 |
| PIGT | 51604 | chr20 | 45415200 | 45419799 | high | 11.50 | 0.001136 |
| PIGU | 128869 | chr20 | 34636600 | 34640599 | high | 10.00 | 0.003721 |
| PIGU | 128869 | chr20 | 34578800 | 34583999 | high | 13.00 | 0.003727 |
| PIGU | 128869 | chr20 | 34672200 | 34681999 | high | 24.50 | 0.003717 |
| PIGX | 54965 | chr3 | 196710600 | 196712799 | high | 5.50 | 0.000279 |
| PIGY | 84992 | chr4 | 88522600 | 88524599 | high | 5.00 | 0.000960 |
| PIGZ | 80235 | chr3 | 196965400 | 196969999 | high | 11.50 | 0.000407 |
| PIH1D2 | 120379 | chr11 | 112068600 | 112070799 | low | -9.56 | 0.001074 |
| PIK3C3 | 5289 | chr18 | 42027400 | 42030599 | high | 8.00 | 0.000126 |
| PIK3CB | 5291 | chr3 | 138747600 | 138751799 | high | 10.50 | 0.000038 |
| PIK3CD | 5293 | chr1 | 9715600 | 9719199 | high | 9.00 | 0.000545 |
| PIK3CD | 5293 | chr1 | 9650600 | 9656599 | high | 15.00 | 0.000548 |
| PIK3IP1 | 113791 | chr22 | 31288400 | 31293999 | high | 14.00 | 0.003637 |
| PIK3R1 | 5295 | chr5 | 68215000 | 68217199 | high | 5.50 | 0.000078 |
| PIK3R1 | 5295 | chr5 | 68268800 | 68271799 | high | 7.50 | 0.000078 |
| PIK3R1 | 5295 | chr5 | 68232000 | 68235199 | high | 8.00 | 0.000078 |
| PIK3R2 | 5296 | chr19 | 18152400 | 18155999 | high | 9.00 | 0.000292 |
| PIK3R3 | 8503 | chr1 | 46039200 | 46042399 | high | 8.00 | 0.000185 |
| PIN1 | 5300 | chr19 | 9833000 | 9839399 | high | 16.00 | 0.000539 |
| PIP4K2C | 79837 | chr12 | 57592400 | 57596999 | high | 11.50 | 0.001386 |
| PIP5K1A | 8394 | chr1 | 151237000 | 151240399 | high | 8.50 | 0.000056 |
| PIP5K1A | 8394 | chr1 | 151241600 | 151246799 | high | 13.00 | 0.000056 |
| PIP5K1C | 23396 | chr19 | 3697600 | 3700599 | high | 7.50 | 0.006327 |
| PIP5K1C | 23396 | chr19 | 3635200 | 3640599 | high | 13.50 | 0.006436 |
| PIP5KL1 | 138429 | chr9 | 127927200 | 127930999 | high | 9.50 | 0.001082 |
| PISD | 23761 | chr22 | 31629200 | 31631199 | high | 5.00 | 0.000751 |
| PITHD1 | 57095 | chr1 | 23777200 | 23779199 | low | -8.69 | 0.002401 |
| PITPNA | 5306 | chr17 | 1515400 | 1518199 | high | 7.00 | 0.003501 |
| PITPNC1 | 26207 | chr17 | 67525200 | 67527399 | high | 5.50 | 0.000388 |
| PITPNC1 | 26207 | chr17 | 67499000 | 67501199 | high | 5.50 | 0.000388 |
| PITPNC1 | 26207 | chr17 | 67482400 | 67484799 | high | 6.00 | 0.000388 |
| PITPNC1 | 26207 | chr17 | 67413200 | 67416199 | high | 7.50 | 0.000389 |
| PITPNC1 | 26207 | chr17 | 67646800 | 67649999 | high | 8.00 | 0.000387 |
| PITPNC1 | 26207 | chr17 | 67490400 | 67493799 | high | 8.50 | 0.000388 |
| PITPNC1 | 26207 | chr17 | 67477600 | 67481599 | high | 10.00 | 0.000388 |
| PITPNC1 | 26207 | chr17 | 67408000 | 67412399 | high | 11.00 | 0.000389 |
| PITPNC1 | 26207 | chr17 | 67637400 | 67642399 | high | 12.50 | 0.000387 |
| PITPNC1 | 26207 | chr17 | 67421800 | 67427799 | high | 15.00 | 0.000389 |
| PITPNM2 | 57605 | chr12 | 123093600 | 123096999 | high | 8.50 | 0.000468 |
| PITPNM2 | 57605 | chr12 | 123089000 | 123092599 | high | 9.00 | 0.000468 |
| PITPNM2 | 57605 | chr12 | 122980000 | 122983599 | high | 9.00 | 0.000468 |
| PITPNM2 | 57605 | chr12 | 122986800 | 122991199 | high | 11.00 | 0.000468 |
| PITRM1 | 10531 | chr10 | 3169600 | 3173599 | high | 10.00 | 0.003323 |
| PITX3 | 5309 | chr10 | 102229800 | 102234999 | high | 13.00 | 0.000052 |
| PITX3 | 5309 | chr10 | 102237400 | 102243799 | high | 16.00 | 0.000052 |
| PIWIL2 | 55124 | chr8 | 22298400 | 22303199 | high | 12.00 | 0.002472 |

| | | | | | | | |
|---|---|---|---|---|---|---|---|
| PIWIL2 | 55124 | chr8 | 22281000 | 22286199 | high | 13.00 | 0.002474 |
| PIWIL2 | 55124 | chr8 | 22270600 | 22275999 | high | 13.50 | 0.002475 |
| PJA2 | 9867 | chr5 | 109383800 | 109388599 | high | 12.00 | 0.000090 |
| PKD1L1 | 168507 | chr7 | 47778400 | 47781799 | low | -14.78 | 0.003527 |
| PKD1L1 | 168507 | chr7 | 47800400 | 47803599 | low | -13.91 | 0.003525 |
| PKD2 | 5311 | chr4 | 88007600 | 88011599 | high | 10.00 | 0.000060 |
| PKDCC | 91461 | chr2 | 42048400 | 42050599 | high | 5.50 | 0.002175 |
| PKIB | 5570 | chr6 | 122470800 | 122473999 | low | -13.91 | 0.000045 |
| PKIG | 11142 | chr20 | 44573000 | 44577599 | high | 11.50 | 0.000250 |
| PKM | 5315 | chr15 | 72220200 | 72224599 | high | 11.00 | 0.000074 |
| PKMYT1 | 9088 | chr16 | 2977800 | 2979799 | low | -8.69 | 0.003052 |
| PKN2 | 5586 | chr1 | 88749800 | 88752599 | high | 7.00 | 0.000063 |
| PKNOX1 | 5316 | chr21 | 42974600 | 42975799 | low | -5.21 | 0.000124 |
| PKP3 | 11187 | chr11 | 394000 | 397799 | high | 9.50 | 0.003155 |
| PKP4 | 8502 | chr2 | 158490000 | 158491799 | low | -7.82 | 0.000054 |
| PKP4 | 8502 | chr2 | 158493000 | 158494599 | low | -6.95 | 0.000054 |
| PLAT | 5327 | chr8 | 42195800 | 42198199 | high | 6.00 | 0.000126 |
| PLAU | 5328 | chr10 | 73909000 | 73913599 | high | 11.50 | 0.000072 |
| PLB1 | 151056 | chr2 | 28522200 | 28525399 | high | 8.00 | 0.005296 |
| PLB1 | 151056 | chr2 | 28596000 | 28599999 | high | 10.00 | 0.005282 |
| PLB1 | 151056 | chr2 | 28568400 | 28572799 | high | 11.00 | 0.005288 |
| PLB1 | 151056 | chr2 | 28638200 | 28644399 | high | 15.50 | 0.005275 |
| PLCB1 | 23236 | chr20 | 8856800 | 8859999 | high | 8.00 | 0.002624 |
| PLCB1 | 23236 | chr20 | 8852200 | 8855999 | high | 9.50 | 0.002625 |
| PLCB1 | 23236 | chr20 | 8774800 | 8779399 | high | 11.50 | 0.002648 |
| PLCB1 | 23236 | chr20 | 8789200 | 8793999 | high | 12.00 | 0.002644 |
| PLCB1 | 23236 | chr20 | 8794800 | 8799799 | high | 12.50 | 0.002642 |
| PLCB1 | 23236 | chr20 | 8845800 | 8850999 | high | 13.00 | 0.002627 |
| PLCB1 | 23236 | chr20 | 8760400 | 8765799 | high | 13.50 | 0.002652 |
| PLCB3 | 5331 | chr11 | 64250200 | 64253399 | high | 8.00 | 0.000083 |
| PLCB3 | 5331 | chr11 | 64266200 | 64270799 | high | 11.50 | 0.000083 |
| PLCB4 | 5332 | chr20 | 9073200 | 9076399 | high | 8.00 | 0.000588 |
| PLCB4 | 5332 | chr20 | 9085600 | 9089199 | high | 9.00 | 0.000587 |
| PLCB4 | 5332 | chr20 | 9260800 | 9264599 | high | 9.50 | 0.000576 |
| PLCB4 | 5332 | chr20 | 9206400 | 9210199 | high | 9.50 | 0.000579 |
| PLCB4 | 5332 | chr20 | 9246800 | 9251199 | high | 11.00 | 0.000577 |
| PLCB4 | 5332 | chr20 | 9094800 | 9099599 | high | 12.00 | 0.000586 |
| PLCB4 | 5332 | chr20 | 9067200 | 9071999 | high | 12.00 | 0.000588 |
| PLCB4 | 5332 | chr20 | 9316200 | 9321599 | high | 13.50 | 0.000572 |
| PLCB4 | 5332 | chr20 | 9274600 | 9280599 | high | 15.00 | 0.000575 |
| PLCB4 | 5332 | chr20 | 9306200 | 9314799 | high | 21.50 | 0.000573 |
| PLCD3 | 113026 | chr17 | 45109800 | 45114199 | high | 11.00 | 0.002506 |
| PLCD3 | 113026 | chr17 | 45118200 | 45123199 | high | 12.50 | 0.002505 |
| PLCE1 | 51196 | chr10 | 94316000 | 94319199 | high | 8.00 | 0.000543 |
| PLCE1 | 51196 | chr10 | 94099600 | 94104199 | high | 11.50 | 0.000544 |
| PLCG1 | 5335 | chr20 | 41167600 | 41171399 | high | 9.50 | 0.000130 |
| PLCXD2 | 257068 | chr3 | 111765400 | 111768999 | high | 9.00 | 0.002300 |
| PLCXD2 | 257068 | chr3 | 111678600 | 111683999 | high | 13.50 | 0.002302 |
| PLCXD2 | 257068 | chr3 | 111732600 | 111739999 | high | 18.50 | 0.002301 |
| PLD2 | 5338 | chr17 | 4805600 | 4808999 | high | 8.50 | 0.001111 |
| PLD3 | 23646 | chr19 | 40347600 | 40349399 | high | 4.50 | 0.000586 |
| PLEC | 5339 | chr8 | 143950400 | 143952999 | high | 6.50 | 0.000037 |
| PLEC | 5339 | chr8 | 143940600 | 143943799 | high | 8.00 | 0.000037 |
| PLEK2 | 26499 | chr14 | 67409400 | 67414999 | high | 14.00 | 0.000393 |
| PLIN2 | 123 | chr9 | 19126400 | 19128399 | low | -8.69 | 0.000006 |
| PLIN3 | 10226 | chr19 | 4866400 | 4868799 | low | -10.43 | 0.002101 |
| PLOD2 | 5352 | chr3 | 146109400 | 146112199 | high | 7.00 | 0.000037 |
| PLOD2 | 5352 | chr3 | 146159600 | 146162999 | high | 8.50 | 0.000037 |
| PLOD2 | 5352 | chr3 | 146136200 | 146141399 | high | 13.00 | 0.000037 |
| PLOD2 | 5352 | chr3 | 146150200 | 146157599 | high | 18.50 | 0.000037 |
| PLOD3 | 8985 | chr7 | 101215600 | 101222599 | high | 17.50 | 0.000089 |
| PLPP1 | 8611 | chr5 | 55533600 | 55535399 | high | 4.50 | 0.000155 |
| PLPP1 | 8611 | chr5 | 55463200 | 55470599 | high | 18.50 | 0.000155 |
| PLPP3 | 8613 | chr1 | 56513800 | 56520399 | high | 16.50 | 0.000152 |
| PLPP6 | 403313 | chr9 | 4663200 | 4666599 | low | -14.78 | 0.009610 |
| PMF1 | 11243 | chr1 | 156211400 | 156216399 | high | 12.50 | 0.000072 |
| PML | 5371 | chr15 | 74000400 | 74005599 | high | 13.00 | 0.000073 |
| PML | 5371 | chr15 | 74014800 | 74020199 | high | 13.50 | 0.000073 |
| PMP22 | 5376 | chr17 | 15259800 | 15262199 | high | 6.00 | 0.000352 |
| PMPCA | 23203 | chr9 | 136410200 | 136412399 | low | -9.56 | 0.000170 |
| PMS1 | 5378 | chr2 | 189810000 | 189814599 | high | 11.50 | 0.000028 |
| PMS2 | 5395 | chr7 | 6007800 | 6013399 | high | 14.00 | 0.000898 |
| PMS2P3 | 5387 | chr7 | 75526800 | 75528999 | high | 5.50 | 0.000071 |
| PMS2P4 | 5382 | chr7 | 67301800 | 67305399 | high | 9.00 | 0.000080 |
| PNKD | 25953 | chr2 | 218286600 | 218290599 | high | 10.00 | 0.000119 |
| PNKD | 25953 | chr2 | 218291400 | 218297999 | high | 16.50 | 0.000119 |
| PNKP | 11284 | chr19 | 49857000 | 49862399 | high | 13.50 | 0.000226 |
| PNO1 | 56902 | chr2 | 68160200 | 68164599 | high | 11.00 | 0.000835 |

| | | | | | | | |
|---|---|---|---|---|---|---|---|
| PNPLA2 | 57104 | chr11 | 823200 | 826799 | high | 9.00 | 0.007561 |
| PNPLA6 | 10908 | chr19 | 7533000 | 7536799 | high | 9.50 | 0.001448 |
| PNPLA7 | 375775 | chr9 | 137541200 | 137545399 | high | 10.50 | 0.002732 |
| PNPO | 55163 | chr17 | 47944400 | 47950199 | high | 14.50 | 0.001151 |
| PNPT1 | 87178 | chr2 | 55690400 | 55694399 | high | 10.00 | 0.001565 |
| PNRC1 | 10957 | chr6 | 89079000 | 89082799 | high | 9.50 | 0.000123 |
| PNRC2 | 55629 | chr1 | 23960000 | 23960799 | low | -3.47 | 0.002322 |
| POC1B | 282809 | chr12 | 89465000 | 89466799 | high | 4.50 | 0.003161 |
| POC1B | 282809 | chr12 | 89469400 | 89472399 | high | 7.50 | 0.003161 |
| POC1B | 282809 | chr12 | 89447000 | 89449999 | high | 7.50 | 0.003162 |
| POC1B | 282809 | chr12 | 89426000 | 89429799 | high | 9.50 | 0.003162 |
| POC1B | 282809 | chr12 | 89507000 | 89512399 | high | 13.50 | 0.003160 |
| POC1B | 282809 | chr12 | 89417600 | 89425199 | high | 19.00 | 0.003163 |
| POC5 | 134359 | chr5 | 75706000 | 75708399 | low | -10.43 | 0.001775 |
| PODNL1 | 79883 | chr19 | 13949600 | 13954799 | high | 13.00 | 0.005727 |
| PODXL2 | 50512 | chr3 | 127670400 | 127675199 | high | 12.00 | 0.000396 |
| POFUT1 | 23509 | chr20 | 32210600 | 32215999 | high | 13.50 | 0.000730 |
| POLA2 | 23649 | chr11 | 65260200 | 65266599 | high | 16.00 | 0.000362 |
| POLD1 | 5424 | chr19 | 50382800 | 50388399 | high | 14.00 | 0.000108 |
| POLD2 | 5425 | chr7 | 44113600 | 44117199 | high | 9.00 | 0.000123 |
| POLD4 | 57804 | chr11 | 67351000 | 67356599 | high | 14.00 | 0.000858 |
| POLDIP3 | 84271 | chr22 | 42585800 | 42588799 | high | 7.50 | 0.001979 |
| POLE2 | 5427 | chr14 | 49643200 | 49646799 | high | 9.00 | 0.000109 |
| POLE2 | 5427 | chr14 | 49656600 | 49660399 | high | 9.50 | 0.000109 |
| POLE2 | 5427 | chr14 | 49683800 | 49691399 | high | 19.00 | 0.000109 |
| POLE3 | 54107 | chr9 | 113410000 | 113410999 | low | -4.34 | 0.000477 |
| POLG | 5428 | chr15 | 89324400 | 89326799 | high | 6.00 | 0.000061 |
| POLG | 5428 | chr15 | 89315200 | 89322799 | high | 19.00 | 0.000061 |
| POLG2 | 11232 | chr17 | 64480400 | 64483999 | low | -15.65 | 0.000174 |
| POLG2 | 11232 | chr17 | 64495600 | 64497199 | low | -6.95 | 0.000174 |
| POLH | 5429 | chr6 | 43603400 | 43605199 | high | 4.50 | 0.000125 |
| POLM | 27434 | chr7 | 44076800 | 44083199 | high | 16.00 | 0.000622 |
| POLM | 27434 | chr7 | 44069000 | 44075999 | high | 17.50 | 0.000623 |
| POMP | 51371 | chr13 | 28657000 | 28661599 | high | 11.50 | 0.001793 |
| POP1 | 10940 | chr8 | 98156600 | 98158399 | low | -7.82 | 0.000111 |
| POT1 | 25913 | chr7 | 124907600 | 124910599 | low | -13.04 | 0.000207 |
| POU2F2 | 5452 | chr19 | 42116200 | 42119199 | high | 7.50 | 0.000129 |
| POU2F2 | 5452 | chr19 | 42106200 | 42109399 | high | 8.00 | 0.000129 |
| POU2F2 | 5452 | chr19 | 42129200 | 42135199 | high | 15.00 | 0.000129 |
| POU6F1 | 5463 | chr12 | 51210600 | 51214399 | high | 9.50 | 0.000107 |
| PPA1 | 5464 | chr10 | 70232000 | 70233599 | low | -6.95 | 0.000078 |
| PPA2 | 27068 | chr4 | 105472800 | 105473999 | high | 3.00 | 0.000257 |
| PPA2 | 27068 | chr4 | 105395000 | 105398399 | high | 8.50 | 0.000257 |
| PPAN | 56342 | chr19 | 10104400 | 10108199 | high | 9.50 | 0.005576 |
| PPARD | 5467 | chr6 | 35359400 | 35360799 | high | 3.50 | 0.000155 |
| PPARD | 5467 | chr6 | 35420000 | 35422199 | high | 5.50 | 0.000154 |
| PPARD | 5467 | chr6 | 35388200 | 35391999 | high | 9.50 | 0.000154 |
| PPARD | 5467 | chr6 | 35424400 | 35428599 | high | 10.50 | 0.000154 |
| PPARG | 5468 | chr3 | 12359600 | 12365199 | high | 14.00 | 0.000442 |
| PPCDC | 60490 | chr15 | 75034800 | 75039599 | high | 12.00 | 0.000806 |
| PPFIA1 | 8500 | chr11 | 70307600 | 70315999 | high | 21.00 | 0.000121 |
| PPHLN1 | 51535 | chr12 | 42358400 | 42362799 | high | 11.00 | 0.001217 |
| PPHLN1 | 51535 | chr12 | 42332800 | 42337199 | high | 11.00 | 0.001217 |
| PPIA | 5478 | chr7 | 44795200 | 44798399 | high | 8.00 | 0.000122 |
| PPIB | 5479 | chr15 | 64161200 | 64164599 | low | -14.78 | 0.000085 |
| PPIG | 9360 | chr2 | 169623800 | 169625199 | low | -6.08 | 0.000055 |
| PPIL2 | 23759 | chr22 | 21664600 | 21667599 | high | 7.50 | 0.001097 |
| PPIP5K2 | 23262 | chr5 | 103198400 | 103201199 | high | 7.00 | 0.000225 |
| PPM1D | 8493 | chr17 | 60661600 | 60665199 | high | 9.00 | 0.000140 |
| PPM1D | 8493 | chr17 | 60628200 | 60631999 | high | 9.50 | 0.000140 |
| PPM1H | 57460 | chr12 | 62644600 | 62646999 | high | 6.00 | 0.000917 |
| PPM1H | 57460 | chr12 | 62699400 | 62703799 | high | 11.00 | 0.000916 |
| PPM1H | 57460 | chr12 | 62670600 | 62675399 | high | 12.00 | 0.000917 |
| PPM1K | 152926 | chr4 | 88282200 | 88286399 | low | -18.69 | 0.001732 |
| PPM1M | 132160 | chr3 | 52245800 | 52248399 | high | 6.50 | 0.002530 |
| PPME1 | 51400 | chr11 | 74233600 | 74235999 | high | 6.00 | 0.000692 |
| PPME1 | 51400 | chr11 | 74243400 | 74247199 | high | 9.50 | 0.000692 |
| PPP1CC | 5501 | chr12 | 110741200 | 110742799 | low | -6.95 | 0.000050 |
| PPP1R7 | 5510 | chr2 | 241148800 | 241151599 | high | 7.00 | 0.000023 |
| PPP1R8 | 5511 | chr1 | 27830800 | 27834599 | high | 9.50 | 0.000198 |
| PPP3CA | 5530 | chr4 | 101306000 | 101309399 | high | 8.50 | 0.000055 |
| PPP3CA | 5530 | chr4 | 101268600 | 101272199 | high | 9.00 | 0.000055 |
| PPP3CA | 5530 | chr4 | 101279400 | 101283999 | high | 11.50 | 0.000055 |
| PPP3CA | 5530 | chr4 | 101273800 | 101278599 | high | 12.00 | 0.000055 |
| PPP3CB | 5532 | chr10 | 73435400 | 73437999 | low | -11.30 | 0.000075 |
| PPP3CB | 5532 | chr10 | 73438800 | 73441199 | low | -10.43 | 0.000075 |
| PPP3CC | 5533 | chr8 | 22443800 | 22446999 | high | 8.00 | 0.000247 |
| PPP3CC | 5533 | chr8 | 22482600 | 22486399 | high | 9.50 | 0.000246 |

| | | | | | | | |
|---|---|---|---|---|---|---|---|
| PPP3CC | 5533 | chr8 | 22459200 | 22465799 | high | 16.50 | 0.000246 |
| PPP3R1 | 5534 | chr2 | 68190800 | 68194399 | high | 9.00 | 0.000081 |
| PPP4C | 5531 | chr16 | 30075800 | 30078399 | high | 6.50 | 0.000184 |
| PPP4R1 | 9989 | chr18 | 9594600 | 9598199 | high | 9.00 | 0.001041 |
| PPP4R1 | 9989 | chr18 | 9546400 | 9551399 | high | 12.50 | 0.001046 |
| PPP4R1 | 9989 | chr18 | 9604200 | 9609999 | high | 14.50 | 0.001040 |
| PPP4R3B | 57223 | chr2 | 55604200 | 55607199 | low | -13.04 | 0.001029 |
| PPP6C | 5537 | chr9 | 125188800 | 125190599 | high | 4.50 | 0.000044 |
| PPP6R1 | 22870 | chr19 | 55226600 | 55233199 | high | 16.50 | 0.000414 |
| PPP6R2 | 9701 | chr22 | 50443400 | 50448399 | high | 12.50 | 0.000192 |
| PPP6R2 | 9701 | chr22 | 50412800 | 50417799 | high | 12.50 | 0.000192 |
| PPP6R2 | 9701 | chr22 | 50373800 | 50378799 | high | 12.50 | 0.000193 |
| PPP6R3 | 55291 | chr11 | 68459600 | 68462399 | high | 7.00 | 0.000808 |
| PPWD1 | 23398 | chr5 | 65563200 | 65565599 | high | 6.00 | 0.000357 |
| PPWD1 | 23398 | chr5 | 65575000 | 65578599 | high | 9.00 | 0.000357 |
| PQLC1 | 80148 | chr18 | 79950200 | 79952599 | high | 6.00 | 0.001002 |
| PRADC1 | 84279 | chr2 | 73233000 | 73236399 | high | 8.50 | 0.001151 |
| PRC1 | 9055 | chr15 | 90992800 | 90995799 | high | 7.50 | 0.000100 |
| PRC1 | 9055 | chr15 | 90963600 | 90969199 | high | 14.00 | 0.000100 |
| PRCC | 5546 | chr1 | 156788000 | 156792199 | low | -18.06 | 0.000035 |
| PRCD | 768206 | chr17 | 76541000 | 76544199 | high | 8.00 | 0.010037 |
| PRCD | 768206 | chr17 | 76529800 | 76539599 | high | 24.50 | 0.010038 |
| PRDM1 | 639 | chr6 | 106089800 | 106091399 | low | -6.95 | 0.000006 |
| PRDM2 | 7799 | chr1 | 13747200 | 13749999 | low | -12.17 | 0.000567 |
| PRDM4 | 11108 | chr12 | 107756800 | 107761799 | high | 12.50 | 0.000103 |
| PRDX1 | 5052 | chr1 | 45520800 | 45521799 | low | -4.34 | 0.000111 |
| PRDX2 | 7001 | chr19 | 12799800 | 12802999 | high | 8.00 | 0.000547 |
| PRDX3 | 10935 | chr10 | 119165200 | 119170599 | high | 13.50 | 0.000092 |
| PRDX5 | 25824 | chr11 | 64316600 | 64320799 | high | 10.50 | 0.000402 |
| PREP | 5550 | chr6 | 105400400 | 105404199 | low | -16.52 | 0.000053 |
| PREX1 | 57580 | chr20 | 48755000 | 48758199 | high | 8.00 | 0.001181 |
| PREX1 | 57580 | chr20 | 48653200 | 48656599 | high | 8.50 | 0.001183 |
| PREX1 | 57580 | chr20 | 48824400 | 48828599 | high | 10.50 | 0.001179 |
| PREX1 | 57580 | chr20 | 48780800 | 48785999 | high | 13.00 | 0.001180 |
| PREX1 | 57580 | chr20 | 48765400 | 48770999 | high | 14.00 | 0.001181 |
| PRIM1 | 5557 | chr12 | 56749000 | 56750599 | low | -6.95 | 0.000098 |
| PRKCA | 5578 | chr17 | 66397600 | 66399999 | high | 6.00 | 0.000084 |
| PRKCA | 5578 | chr17 | 66464400 | 66466999 | high | 6.50 | 0.000084 |
| PRKCA | 5578 | chr17 | 66442800 | 66445799 | high | 7.50 | 0.000084 |
| PRKCA | 5578 | chr17 | 66473200 | 66476799 | high | 9.00 | 0.000084 |
| PRKCA | 5578 | chr17 | 66423600 | 66428599 | high | 12.50 | 0.000084 |
| PRKCA | 5578 | chr17 | 66436800 | 66441999 | high | 13.00 | 0.000084 |
| PRKCA | 5578 | chr17 | 66449400 | 66455199 | high | 14.50 | 0.000084 |
| PRKCA | 5578 | chr17 | 66503400 | 66509399 | high | 15.00 | 0.000084 |
| PRKCA | 5578 | chr17 | 66429400 | 66435999 | high | 16.50 | 0.000084 |
| PRKCA | 5578 | chr17 | 66490800 | 66500799 | high | 25.00 | 0.000084 |
| PRKCD | 5580 | chr3 | 53160800 | 53162399 | low | -6.95 | 0.000105 |
| PRKCE | 5581 | chr2 | 46130600 | 46134399 | high | 9.50 | 0.000121 |
| PRKCE | 5581 | chr2 | 46143400 | 46147599 | high | 10.50 | 0.000121 |
| PRKCE | 5581 | chr2 | 46072400 | 46076799 | high | 11.00 | 0.000121 |
| PRKCE | 5581 | chr2 | 46116600 | 46121199 | high | 11.50 | 0.000121 |
| PRKCE | 5581 | chr2 | 46090400 | 46095199 | high | 12.00 | 0.000121 |
| PRKCE | 5581 | chr2 | 46013600 | 46018599 | high | 12.50 | 0.000121 |
| PRKCE | 5581 | chr2 | 46151400 | 46156599 | high | 13.00 | 0.000121 |
| PRKCE | 5581 | chr2 | 46182800 | 46188799 | high | 15.00 | 0.000121 |
| PRKCE | 5581 | chr2 | 46064800 | 46071399 | high | 16.50 | 0.000121 |
| PRKCE | 5581 | chr2 | 46167000 | 46174599 | high | 19.00 | 0.000121 |
| PRKCE | 5581 | chr2 | 46106200 | 46113799 | high | 19.00 | 0.000121 |
| PRKCH | 5583 | chr14 | 61507000 | 61508199 | high | 3.00 | 0.000091 |
| PRKCH | 5583 | chr14 | 61511200 | 61515399 | high | 10.50 | 0.000091 |
| PRKCI | 5584 | chr3 | 170257200 | 170260199 | low | -13.04 | 0.000033 |
| PRKCI | 5584 | chr3 | 170243400 | 170245799 | low | -10.43 | 0.000033 |
| PRKD1 | 5587 | chr14 | 29828600 | 29831199 | high | 6.50 | 0.000187 |
| PRKD1 | 5587 | chr14 | 29582400 | 29584999 | high | 6.50 | 0.000189 |
| PRKD1 | 5587 | chr14 | 29703600 | 29707799 | high | 10.50 | 0.000188 |
| PRKD1 | 5587 | chr14 | 29842200 | 29846599 | high | 11.00 | 0.000187 |
| PRKD1 | 5587 | chr14 | 29625600 | 29630399 | high | 12.00 | 0.000189 |
| PRKD1 | 5587 | chr14 | 29819800 | 29825999 | high | 15.50 | 0.000187 |
| PRKD1 | 5587 | chr14 | 29753000 | 29759599 | high | 16.50 | 0.000188 |
| PRKD2 | 25865 | chr19 | 46716000 | 46717999 | high | 5.00 | 0.000554 |
| PRKD3 | 23683 | chr2 | 37254200 | 37256999 | high | 7.00 | 0.000636 |
| PRKD3 | 23683 | chr2 | 37306200 | 37311199 | high | 12.50 | 0.000635 |
| PRKDC | 5591 | chr8 | 47957400 | 47961799 | high | 11.00 | 0.000117 |
| PRKDC | 5591 | chr8 | 47853800 | 47859799 | high | 15.00 | 0.000117 |
| PRKG1 | 5592 | chr10 | 51201400 | 51203199 | low | -7.82 | 0.000109 |
| PRKRA | 8575 | chr2 | 178436600 | 178439199 | low | -11.30 | 0.000048 |
| PRKRIP1 | 79706 | chr7 | 102424400 | 102426799 | high | 6.00 | 0.000778 |
| PRKRIP1 | 79706 | chr7 | 102395000 | 102397799 | high | 7.00 | 0.000778 |

| | | | | | | | |
|---|---|---|---|---|---|---|---|
| PRMT2 | 3275 | chr21 | 46644600 | 46647999 | high | 8.50 | 0.000070 |
| PRMT2 | 3275 | chr21 | 46633600 | 46638999 | high | 13.50 | 0.000070 |
| PRMT3 | 10196 | chr11 | 20386400 | 20388999 | low | -11.30 | 0.000500 |
| PRMT5 | 10419 | chr14 | 22918000 | 22921199 | high | 8.00 | 0.000455 |
| PRMT5 | 10419 | chr14 | 22924600 | 22930599 | high | 15.00 | 0.000454 |
| PRMT6 | 55170 | chr1 | 107056200 | 107059399 | low | -13.91 | 0.000515 |
| PRMT7 | 54496 | chr16 | 68328400 | 68338799 | high | 26.00 | 0.000798 |
| PRNCR1 | 101867536 | chr8 | 127085600 | 127094799 | high | 23.00 | 0.003144 |
| PROB1 | 389333 | chr5 | 139390000 | 139395399 | high | 13.50 | 0.002793 |
| PROM2 | 150696 | chr2 | 95286200 | 95288399 | low | -9.56 | 0.001582 |
| PROSC | 11212 | chr8 | 37773800 | 37780999 | high | 18.00 | 0.000297 |
| PROSER1 | 80209 | chr13 | 39035800 | 39039199 | high | 8.50 | 0.002055 |
| PRPF4B | 8899 | chr6 | 4021000 | 4022999 | low | -8.69 | 0.002213 |
| PRPH2 | 5961 | chr6 | 42701800 | 42705999 | high | 10.50 | 0.000140 |
| PRPSAP1 | 5635 | chr17 | 76336400 | 76340999 | high | 11.50 | 0.000074 |
| PRPSAP2 | 5636 | chr17 | 18855400 | 18859599 | high | 10.50 | 0.000299 |
| PRR16 | 51334 | chr5 | 120540000 | 120542799 | high | 7.00 | 0.000426 |
| PRR16 | 51334 | chr5 | 120637800 | 120641799 | high | 10.00 | 0.000426 |
| PRR16 | 51334 | chr5 | 120613600 | 120619599 | high | 15.00 | 0.000426 |
| PRR16 | 51334 | chr5 | 120508200 | 120514399 | high | 15.50 | 0.000426 |
| PRR19 | 284338 | chr19 | 42301800 | 42306799 | low | -21.57 | 0.006722 |
| PRR34 | 55267 | chr22 | 46053200 | 46056199 | low | -13.04 | 0.001200 |
| PRR5L | 79899 | chr11 | 36394200 | 36398399 | high | 10.50 | 0.002195 |
| PRR5L | 79899 | chr11 | 36459600 | 36465999 | high | 16.00 | 0.002191 |
| PRR7 | 80758 | chr5 | 177444400 | 177449599 | high | 13.00 | 0.000455 |
| PRRC2B | 84726 | chr9 | 131493600 | 131499399 | high | 14.50 | 0.000644 |
| PRRC2C | 23215 | chr1 | 171484000 | 171487799 | high | 9.50 | 0.000135 |
| PRRC2C | 23215 | chr1 | 171580800 | 171586399 | high | 14.00 | 0.000135 |
| PRRT2 | 112476 | chr16 | 29813800 | 29822999 | high | 23.00 | 0.003773 |
| PRRT3 | 285368 | chr3 | 9946200 | 9947999 | low | -7.82 | 0.003188 |
| PRRX2 | 51450 | chr9 | 129669200 | 129671999 | high | 7.00 | 0.000397 |
| PRSS12 | 8492 | chr4 | 118300000 | 118305799 | high | 14.50 | 0.000072 |
| PRSS12 | 8492 | chr4 | 118283400 | 118289999 | high | 16.50 | 0.000072 |
| PRSS23 | 11098 | chr11 | 86917000 | 86920199 | high | 8.00 | 0.000128 |
| PRSS23 | 11098 | chr11 | 86816600 | 86820599 | high | 10.00 | 0.000128 |
| PRSS23 | 11098 | chr11 | 86912000 | 86916199 | high | 10.50 | 0.000128 |
| PRSS23 | 11098 | chr11 | 86807000 | 86811799 | high | 12.00 | 0.000128 |
| PRSS23 | 11098 | chr11 | 86921400 | 86927799 | high | 16.00 | 0.000128 |
| PRSS23 | 11098 | chr11 | 86946400 | 86953399 | high | 17.50 | 0.000128 |
| PRTFDC1 | 56952 | chr10 | 24920600 | 24922399 | low | -7.82 | 0.002285 |
| PRTFDC1 | 56952 | chr10 | 24901600 | 24905799 | high | 10.50 | 0.002287 |
| PRTG | 283659 | chr15 | 55615400 | 55617799 | low | -10.43 | 0.005100 |
| PRUNE2 | 158471 | chr9 | 76648200 | 76651399 | low | -13.91 | 0.002068 |
| PRUNE2 | 158471 | chr9 | 76640800 | 76643399 | low | -11.30 | 0.002068 |
| PRUNE2 | 158471 | chr9 | 76625000 | 76626599 | low | -6.95 | 0.002068 |
| PSCA | 8000 | chr8 | 142679800 | 142684599 | high | 12.00 | 0.000056 |
| PSD | 5662 | chr10 | 102407200 | 102410999 | high | 9.50 | 0.000055 |
| PSD3 | 23362 | chr8 | 18856600 | 18859999 | high | 8.50 | 0.001239 |
| PSD3 | 23362 | chr8 | 18795400 | 18800799 | high | 13.50 | 0.001243 |
| PSEN1 | 5663 | chr14 | 73176200 | 73178399 | high | 5.50 | 0.000077 |
| PSEN1 | 5663 | chr14 | 73195400 | 73200199 | high | 12.00 | 0.000077 |
| PSENEN | 55851 | chr19 | 35743800 | 35746599 | low | -12.17 | 0.001563 |
| PSIP1 | 11168 | chr9 | 15509400 | 15512399 | low | -13.04 | 0.000720 |
| PSMA1 | 5682 | chr11 | 14577600 | 14580199 | low | -11.30 | 0.000390 |
| PSMA2 | 5683 | chr7 | 42930600 | 42933799 | low | -13.91 | 0.000132 |
| PSMA2 | 5683 | chr7 | 42920600 | 42922599 | low | -8.69 | 0.000132 |
| PSMA3 | 5684 | chr14 | 58265000 | 58267599 | high | 6.50 | 0.000098 |
| PSMA4 | 5685 | chr15 | 78539800 | 78541199 | low | -6.08 | 0.000072 |
| PSMA6 | 5687 | chr14 | 35313200 | 35315799 | high | 6.50 | 0.000161 |
| PSMA6 | 5687 | chr14 | 35278600 | 35286199 | high | 19.00 | 0.000161 |
| PSMB2 | 5690 | chr1 | 35639600 | 35642599 | high | 7.50 | 0.000160 |
| PSMB4 | 5692 | chr1 | 151399600 | 151401199 | low | -6.95 | 0.000038 |
| PSMB5 | 5693 | chr14 | 23032400 | 23035799 | high | 8.50 | 0.000247 |
| PSMB6 | 5694 | chr17 | 4795600 | 4797599 | low | -8.69 | 0.001187 |
| PSMC3IP | 29893 | chr17 | 42577400 | 42582199 | high | 12.00 | 0.000702 |
| PSMD11 | 5717 | chr17 | 32443800 | 32446399 | low | -11.30 | 0.000176 |
| PSMD13 | 5719 | chr11 | 237000 | 237999 | low | -4.34 | 0.002681 |
| PSMD14 | 10213 | chr2 | 161307400 | 161310199 | high | 7.00 | 0.000063 |
| PSMD2 | 5708 | chr3 | 184298600 | 184300399 | low | -7.82 | 0.000031 |
| PSMD3 | 5709 | chr17 | 39980200 | 39981599 | low | -6.08 | 0.000143 |
| PSMD4 | 5710 | chr1 | 151254000 | 151255799 | low | -7.82 | 0.000038 |
| PSMD5 | 5711 | chr9 | 120841800 | 120843199 | low | -6.08 | 0.000047 |
| PSMD6 | 9861 | chr3 | 64018600 | 64021999 | high | 8.50 | 0.000154 |
| PSMD8 | 5714 | chr19 | 38374000 | 38376999 | high | 7.50 | 0.000149 |
| PSMD8 | 5714 | chr19 | 38382800 | 38386199 | high | 8.50 | 0.000149 |
| PSMD9 | 5715 | chr12 | 121898200 | 121900799 | high | 6.50 | 0.000047 |
| PSMD9 | 5715 | chr12 | 121915600 | 121919599 | high | 10.00 | 0.000047 |
| PSME1 | 5720 | chr14 | 24134000 | 24138399 | high | 11.00 | 0.000237 |

| | | | | | | | |
|---|---|---|---|---|---|---|---|
| PSME3 | 10197 | chr17 | 42832000 | 42836799 | high | 12.00 | 0.000238 |
| PSME3 | 10197 | chr17 | 42838200 | 42844799 | high | 16.50 | 0.000238 |
| PSME4 | 23198 | chr2 | 53969600 | 53971799 | low | -9.56 | 0.000430 |
| PSMG1 | 8624 | chr21 | 39182200 | 39184799 | low | -11.30 | 0.000220 |
| PSMG3 | 84262 | chr7 | 1569000 | 1571599 | high | 6.50 | 0.005967 |
| PSPC1 | 55269 | chr13 | 19780800 | 19783199 | low | -10.43 | 0.002794 |
| PSPH | 5723 | chr7 | 56047000 | 56051599 | high | 11.50 | 0.000102 |
| PSTPIP2 | 9050 | chr18 | 46052600 | 46056599 | low | -17.91 | 0.000197 |
| PTAFR | 5724 | chr1 | 28173600 | 28176199 | low | -11.30 | 0.000203 |
| PTBP2 | 58155 | chr1 | 96777800 | 96782199 | high | 11.00 | 0.000601 |
| PTBP2 | 58155 | chr1 | 96802200 | 96807399 | high | 13.00 | 0.000601 |
| PTCD1 | 26024 | chr7 | 99437200 | 99441199 | high | 10.00 | 0.000262 |
| PTCD2 | 79810 | chr5 | 72355400 | 72357999 | high | 6.50 | 0.001103 |
| PTCD2 | 79810 | chr5 | 72318200 | 72324599 | high | 16.00 | 0.001104 |
| PTCH2 | 8643 | chr1 | 44840200 | 44844799 | high | 11.50 | 0.000193 |
| PTCH2 | 8643 | chr1 | 44828800 | 44834199 | high | 13.50 | 0.000193 |
| PTDSS1 | 9791 | chr8 | 96265400 | 96273599 | high | 20.50 | 0.000102 |
| PTGER2 | 5732 | chr14 | 52323600 | 52332399 | high | 22.00 | 0.000110 |
| PTGER2 | 5732 | chr14 | 52313000 | 52322199 | high | 23.00 | 0.000110 |
| PTGES | 9536 | chr9 | 129748600 | 129751799 | high | 8.00 | 0.000073 |
| PTGES3 | 10728 | chr12 | 56687200 | 56689599 | low | -10.43 | 0.000189 |
| PTGFR | 5737 | chr1 | 78533000 | 78537199 | high | 10.50 | 0.000073 |
| PTGFR | 5737 | chr1 | 78526800 | 78531599 | high | 12.00 | 0.000073 |
| PTGIS | 5740 | chr20 | 49543200 | 49546799 | high | 9.00 | 0.000116 |
| PTGR1 | 22949 | chr9 | 111598000 | 111599599 | low | -6.95 | 0.000206 |
| PTGR2 | 145482 | chr14 | 73875400 | 73879399 | high | 10.00 | 0.001969 |
| PTGS1 | 5742 | chr9 | 122368200 | 122375999 | high | 19.50 | 0.000047 |
| PTK2 | 5747 | chr8 | 140968000 | 140970799 | high | 7.00 | 0.000041 |
| PTK2 | 5747 | chr8 | 140951000 | 140954399 | high | 8.50 | 0.000041 |
| PTK2 | 5747 | chr8 | 140909000 | 140912799 | high | 9.50 | 0.000041 |
| PTK2 | 5747 | chr8 | 140662200 | 140666799 | high | 11.50 | 0.000041 |
| PTK7 | 5754 | chr6 | 43075000 | 43078399 | high | 8.50 | 0.000134 |
| PTK7 | 5754 | chr6 | 43113000 | 43117799 | high | 12.00 | 0.000133 |
| PTMA | 5757 | chr2 | 231708800 | 231711199 | high | 6.00 | 0.000025 |
| PTMS | 5763 | chr12 | 6770800 | 6775799 | high | 12.50 | 0.000851 |
| PTN | 5764 | chr7 | 137271600 | 137275399 | low | -16.52 | 0.000042 |
| PTN | 5764 | chr7 | 137310000 | 137313199 | low | -13.91 | 0.000042 |
| PTOV1 | 53635 | chr19 | 49857000 | 49862399 | high | 13.50 | 0.001076 |
| PTP4A1 | 7803 | chr6 | 63572200 | 63574799 | low | -11.30 | 0.000123 |
| PTP4A2 | 8073 | chr1 | 31936600 | 31937999 | low | -6.08 | 0.000253 |
| PTPA | 5524 | chr9 | 129144200 | 129147199 | high | 7.50 | 0.000043 |
| PTPA | 5524 | chr9 | 129108800 | 129112399 | high | 9.00 | 0.000043 |
| PTPMT1 | 114971 | chr11 | 47564600 | 47567199 | high | 6.50 | 0.002417 |
| PTPN1 | 5770 | chr20 | 50573600 | 50582799 | high | 23.00 | 0.000114 |
| PTPN11 | 5781 | chr12 | 112429800 | 112434999 | high | 13.00 | 0.000051 |
| PTPN12 | 5782 | chr7 | 77635200 | 77637799 | high | 6.50 | 0.000074 |
| PTPN12 | 5782 | chr7 | 77551200 | 77553799 | high | 6.50 | 0.000075 |
| PTPN12 | 5782 | chr7 | 77544600 | 77548399 | high | 9.50 | 0.000075 |
| PTPN13 | 5783 | chr4 | 86760800 | 86765799 | high | 12.50 | 0.000067 |
| PTPN13 | 5783 | chr4 | 86738600 | 86743599 | high | 12.50 | 0.000067 |
| PTPN13 | 5783 | chr4 | 86719400 | 86724799 | high | 13.50 | 0.000067 |
| PTPN13 | 5783 | chr4 | 86776800 | 86783199 | high | 16.00 | 0.000067 |
| PTPN14 | 5784 | chr1 | 214373200 | 214376799 | high | 9.00 | 0.000027 |
| PTPN14 | 5784 | chr1 | 214363400 | 214367399 | high | 10.00 | 0.000027 |
| PTPN14 | 5784 | chr1 | 214382600 | 214388199 | high | 14.00 | 0.000027 |
| PTPN14 | 5784 | chr1 | 214417200 | 214423599 | high | 16.00 | 0.000027 |
| PTPN22 | 26191 | chr1 | 113860600 | 113866199 | high | 14.00 | 0.000230 |
| PTPN22 | 26191 | chr1 | 113867600 | 113875199 | high | 19.00 | 0.000230 |
| PTPN4 | 5775 | chr2 | 119755600 | 119764599 | high | 22.50 | 0.000048 |
| PTPN6 | 5777 | chr12 | 6959000 | 6961999 | high | 7.50 | 0.000830 |
| PTPN6 | 5777 | chr12 | 6949400 | 6954999 | high | 14.00 | 0.000831 |
| PTPN9 | 5780 | chr15 | 75517200 | 75524599 | high | 18.50 | 0.000077 |
| PTPRA | 5786 | chr20 | 2992200 | 2994799 | low | -11.30 | 0.001934 |
| PTPRF | 5792 | chr1 | 43529000 | 43532799 | high | 9.50 | 0.000133 |
| PTPRF | 5792 | chr1 | 43551200 | 43555999 | high | 12.00 | 0.000133 |
| PTPRG | 5793 | chr3 | 61847400 | 61850799 | high | 8.50 | 0.000094 |
| PTPRG | 5793 | chr3 | 61851800 | 61855399 | high | 9.00 | 0.000094 |
| PTPRG | 5793 | chr3 | 61822200 | 61829399 | high | 18.00 | 0.000094 |
| PTPRJ | 5795 | chr11 | 48095800 | 48099599 | high | 9.50 | 0.000120 |
| PTPRJ | 5795 | chr11 | 48108200 | 48112999 | high | 12.00 | 0.000120 |
| PTPRM | 5797 | chr18 | 8141000 | 8144599 | high | 9.00 | 0.000712 |
| PTPRM | 5797 | chr18 | 8158400 | 8163599 | high | 13.00 | 0.000711 |
| PTPRO | 5800 | chr12 | 15536000 | 15539399 | high | 8.50 | 0.000373 |
| PTPRO | 5800 | chr12 | 15561400 | 15565199 | high | 9.50 | 0.000373 |
| PTPRO | 5800 | chr12 | 15528600 | 15533399 | high | 12.00 | 0.000374 |
| PTPRO | 5800 | chr12 | 15569400 | 15577799 | high | 21.00 | 0.000373 |
| PTPRQ | 374462 | chr12 | 80638800 | 80641799 | high | 7.50 | 0.004644 |
| PTPRQ | 374462 | chr12 | 80661000 | 80664399 | high | 8.50 | 0.004642 |

| | | | | | | | |
|---|---|---|---|---|---|---|---|
| PTPRQ | 374462 | chr12 | 80631000 | 80634399 | high | 8.50 | 0.004644 |
| PTPRQ | 374462 | chr12 | 80672600 | 80677199 | high | 11.50 | 0.004642 |
| PTPRQ | 374462 | chr12 | 80652800 | 80659599 | high | 17.00 | 0.004643 |
| PTRH2 | 51651 | chr17 | 59697400 | 59703199 | high | 14.50 | 0.000865 |
| PUM1 | 9698 | chr1 | 30990600 | 30994399 | high | 9.50 | 0.000313 |
| PUM1 | 9698 | chr1 | 31062200 | 31066199 | high | 10.00 | 0.000312 |
| PUM1 | 9698 | chr1 | 31006000 | 31011199 | high | 13.00 | 0.000313 |
| PUM1 | 9698 | chr1 | 30956000 | 30961199 | high | 13.00 | 0.000313 |
| PUM2 | 23369 | chr2 | 20325200 | 20329199 | high | 10.00 | 0.001150 |
| PUM2 | 23369 | chr2 | 20278000 | 20281999 | high | 10.00 | 0.001152 |
| PUM2 | 23369 | chr2 | 20338600 | 20344999 | high | 16.00 | 0.001149 |
| PURB | 5814 | chr7 | 44878000 | 44886399 | high | 21.00 | 0.000130 |
| PUS1 | 80324 | chr12 | 131928000 | 131930999 | high | 7.50 | 0.000609 |
| PUS10 | 150962 | chr2 | 61016200 | 61018799 | low | -11.30 | 0.002474 |
| PUS7 | 54517 | chr7 | 105519400 | 105523399 | high | 10.00 | 0.000517 |
| PUS7L | 83448 | chr12 | 43730400 | 43735399 | high | 12.50 | 0.001908 |
| PUSL1 | 126789 | chr1 | 1307600 | 1310199 | low | -11.30 | 0.010774 |
| PVT1 | 5820 | chr8 | 127800000 | 127802799 | high | 7.00 | 0.000046 |
| PVT1 | 5820 | chr8 | 127908800 | 127912199 | high | 8.50 | 0.000046 |
| PVT1 | 5820 | chr8 | 127885000 | 127888399 | high | 8.50 | 0.000046 |
| PVT1 | 5820 | chr8 | 127876600 | 127880199 | high | 9.00 | 0.000046 |
| PVT1 | 5820 | chr8 | 127868000 | 127871799 | high | 9.50 | 0.000046 |
| PVT1 | 5820 | chr8 | 127974400 | 127978799 | high | 11.00 | 0.000045 |
| PVT1 | 5820 | chr8 | 127965400 | 127969799 | high | 11.00 | 0.000045 |
| PVT1 | 5820 | chr8 | 127943400 | 127947799 | high | 11.00 | 0.000045 |
| PVT1 | 5820 | chr8 | 127934800 | 127939599 | high | 12.00 | 0.000045 |
| PVT1 | 5820 | chr8 | 127848200 | 127853199 | high | 12.50 | 0.000046 |
| PVT1 | 5820 | chr8 | 128007800 | 128012999 | high | 13.00 | 0.000045 |
| PVT1 | 5820 | chr8 | 127792400 | 127797599 | high | 13.00 | 0.000046 |
| PXK | 54899 | chr3 | 58407400 | 58412399 | high | 12.50 | 0.000940 |
| PXN | 5829 | chr12 | 120245200 | 120250199 | high | 12.50 | 0.000048 |
| PXYLP1 | 92370 | chr3 | 141282800 | 141288799 | high | 15.00 | 0.000654 |
| PYGB | 5834 | chr20 | 25247200 | 25249199 | high | 5.00 | 0.000231 |
| PYGL | 5836 | chr14 | 50927800 | 50930399 | low | -11.30 | 0.000115 |
| PYGL | 5836 | chr14 | 50909800 | 50911799 | low | -8.69 | 0.000115 |
| PYM1 | 84305 | chr12 | 55924200 | 55927999 | low | -16.52 | 0.001507 |
| PYM1 | 84305 | chr12 | 55914800 | 55917599 | high | 7.00 | 0.001508 |
| QPCTL | 54814 | chr19 | 45692200 | 45693399 | low | -5.21 | 0.001200 |
| QRICH1 | 54870 | chr3 | 49025600 | 49030999 | high | 13.50 | 0.001119 |
| QRICH2 | 84074 | chr17 | 76274600 | 76280599 | high | 15.00 | 0.001102 |
| QSER1 | 79832 | chr11 | 32954600 | 32956799 | high | 5.50 | 0.002422 |
| QSER1 | 79832 | chr11 | 32977400 | 32980399 | high | 7.50 | 0.002421 |
| R3HCC1 | 203069 | chr8 | 23287400 | 23291999 | high | 11.50 | 0.008720 |
| R3HCC1 | 203069 | chr8 | 23293400 | 23300599 | high | 18.00 | 0.008718 |
| R3HDM1 | 23518 | chr2 | 135635200 | 135638199 | high | 7.50 | 0.000173 |
| R3HDM2 | 22864 | chr12 | 57279400 | 57282999 | high | 9.00 | 0.000399 |
| R3HDM2 | 22864 | chr12 | 57306000 | 57310399 | high | 11.00 | 0.000399 |
| R3HDM4 | 91300 | chr19 | 898600 | 902199 | high | 9.00 | 0.004928 |
| R3HDM4 | 91300 | chr19 | 907400 | 913799 | high | 16.00 | 0.006171 |
| RAB10 | 10890 | chr2 | 26063200 | 26066999 | high | 9.50 | 0.000418 |
| RAB10 | 10890 | chr2 | 26122000 | 26127799 | high | 14.50 | 0.000417 |
| RAB11A | 8766 | chr15 | 65888600 | 65890999 | low | -10.43 | 0.000133 |
| RAB11A | 8766 | chr15 | 65869000 | 65870999 | low | -8.69 | 0.000133 |
| RAB13 | 5872 | chr1 | 153984000 | 153987399 | high | 8.50 | 0.000038 |
| RAB18 | 22931 | chr10 | 27503600 | 27508199 | high | 11.50 | 0.000834 |
| RAB1A | 5861 | chr2 | 65127600 | 65130599 | high | 7.50 | 0.000090 |
| RAB1A | 5861 | chr2 | 65113000 | 65117399 | high | 11.00 | 0.000090 |
| RAB1B | 81876 | chr11 | 66267800 | 66269599 | low | -7.82 | 0.001236 |
| RAB25 | 57111 | chr1 | 156062800 | 156066199 | low | -14.78 | 0.000366 |
| RAB26 | 25837 | chr16 | 2147000 | 2151799 | high | 12.00 | 0.012034 |
| RAB26 | 25837 | chr16 | 2152800 | 2161599 | high | 22.00 | 0.012002 |
| RAB27A | 5873 | chr15 | 55209800 | 55212999 | high | 8.00 | 0.000106 |
| RAB27B | 5874 | chr18 | 54829800 | 54833199 | high | 8.50 | 0.000107 |
| RAB27B | 5874 | chr18 | 54837000 | 54840799 | high | 9.50 | 0.000107 |
| RAB27B | 5874 | chr18 | 54852200 | 54857999 | high | 14.50 | 0.000107 |
| RAB29 | 8934 | chr1 | 205772400 | 205776799 | high | 11.00 | 0.000043 |
| RAB2A | 5862 | chr8 | 60515600 | 60520999 | high | 13.50 | 0.000097 |
| RAB30 | 27314 | chr11 | 83046800 | 83050599 | high | 9.50 | 0.000329 |
| RAB30 | 27314 | chr11 | 83024200 | 83028599 | high | 11.00 | 0.000329 |
| RAB30 | 27314 | chr11 | 82990600 | 82996599 | high | 15.00 | 0.000329 |
| RAB31 | 11031 | chr18 | 9778000 | 9782199 | high | 10.50 | 0.001128 |
| RAB31 | 11031 | chr18 | 9755400 | 9762999 | high | 19.00 | 0.001131 |
| RAB32 | 10981 | chr6 | 146544200 | 146546399 | high | 5.50 | 0.000075 |
| RAB3A | 5864 | chr19 | 18192400 | 18197599 | high | 13.00 | 0.000322 |
| RAB3B | 5865 | chr1 | 51966600 | 51970799 | high | 10.50 | 0.000113 |
| RAB3B | 5865 | chr1 | 51960000 | 51964399 | high | 11.00 | 0.000113 |
| RABAC1 | 10567 | chr19 | 41957000 | 41959399 | high | 6.00 | 0.000252 |
| RABEP1 | 9135 | chr17 | 5368000 | 5371799 | high | 9.50 | 0.001702 |

| | | | | | | | |
|---|---|---|---|---|---|---|---|
| RABIF | 5877 | chr1 | 202887800 | 202889799 | high | 5.00 | 0.000029 |
| RABL3 | 285282 | chr3 | 120692800 | 120699399 | high | 16.50 | 0.002364 |
| RAC3 | 5881 | chr17 | 82030600 | 82032799 | high | 5.50 | 0.000072 |
| RAD18 | 56852 | chr3 | 8881000 | 8887399 | high | 16.00 | 0.006402 |
| RAD21 | 5885 | chr8 | 116850600 | 116853799 | low | -13.91 | 0.000050 |
| RAD21L1 | 642636 | chr20 | 1231400 | 1235599 | high | 10.50 | 0.009427 |
| RAD21L1 | 642636 | chr20 | 1240600 | 1245799 | high | 13.00 | 0.005202 |
| RAD23B | 5887 | chr9 | 107306800 | 107310999 | high | 10.50 | 0.000055 |
| RAD23B | 5887 | chr9 | 107315200 | 107319999 | high | 12.00 | 0.000055 |
| RAD50 | 10111 | chr5 | 132558600 | 132562399 | high | 9.50 | 0.000076 |
| RAD51 | 5888 | chr15 | 40694600 | 40698599 | high | 10.00 | 0.000145 |
| RAD52 | 5893 | chr12 | 924800 | 930399 | high | 14.00 | 0.006372 |
| RAD52 | 5893 | chr12 | 989800 | 995799 | high | 15.00 | 0.005954 |
| RAI1 | 10743 | chr17 | 17780600 | 17783599 | high | 7.50 | 0.000604 |
| RAI1 | 10743 | chr17 | 17712200 | 17715199 | high | 7.50 | 0.000607 |
| RAI1 | 10743 | chr17 | 17791600 | 17796599 | high | 12.50 | 0.000604 |
| RAI1 | 10743 | chr17 | 17728400 | 17733399 | high | 12.50 | 0.000606 |
| RAI1 | 10743 | chr17 | 17739800 | 17748599 | high | 22.00 | 0.000606 |
| RALA | 5898 | chr7 | 39689200 | 39692799 | low | -15.65 | 0.000149 |
| RALA | 5898 | chr7 | 39666000 | 39667999 | low | -8.69 | 0.000149 |
| RALBP1 | 10928 | chr18 | 9510600 | 9514199 | high | 9.00 | 0.001149 |
| RALBP1 | 10928 | chr18 | 9520000 | 9523999 | high | 10.00 | 0.001148 |
| RALBP1 | 10928 | chr18 | 9533400 | 9538599 | high | 13.00 | 0.001146 |
| RALBP1 | 10928 | chr18 | 9527000 | 9532399 | high | 13.50 | 0.001147 |
| RALBP1 | 10928 | chr18 | 9502000 | 9507799 | high | 14.50 | 0.001150 |
| RALGPS1 | 9649 | chr9 | 127151400 | 127153199 | low | -7.82 | 0.000076 |
| RALGPS2 | 55103 | chr1 | 178725000 | 178726799 | high | 4.50 | 0.000308 |
| RALGPS2 | 55103 | chr1 | 178863400 | 178867599 | high | 10.50 | 0.000308 |
| RALGPS2 | 55103 | chr1 | 178761800 | 178766999 | high | 13.00 | 0.000308 |
| RAMP1 | 10267 | chr2 | 237869000 | 237874599 | high | 14.00 | 0.000043 |
| RANBP2 | 5903 | chr2 | 108774200 | 108779799 | high | 14.00 | 0.000054 |
| RANBP3 | 8498 | chr19 | 5967000 | 5969599 | high | 6.50 | 0.001424 |
| RANBP3 | 8498 | chr19 | 5956000 | 5959199 | high | 8.00 | 0.001427 |
| RANBP3 | 8498 | chr19 | 5962200 | 5966199 | high | 10.00 | 0.001425 |
| RANBP3 | 8498 | chr19 | 5932600 | 5936799 | high | 10.50 | 0.001432 |
| RANBP3 | 8498 | chr19 | 5974400 | 5980399 | high | 15.00 | 0.001422 |
| RANBP9 | 10048 | chr6 | 13708200 | 13712399 | high | 10.50 | 0.000733 |
| RANGAP1 | 5905 | chr22 | 41284600 | 41288399 | high | 9.50 | 0.000143 |
| RAP1A | 5906 | chr1 | 111644000 | 111645799 | high | 4.50 | 0.000053 |
| RAP2A | 5911 | chr13 | 97432800 | 97435799 | high | 7.50 | 0.000061 |
| RAPH1 | 65059 | chr2 | 203533800 | 203536399 | high | 6.50 | 0.000320 |
| RAPH1 | 65059 | chr2 | 203516000 | 203519399 | high | 8.50 | 0.000320 |
| RAPH1 | 65059 | chr2 | 203461400 | 203464999 | high | 9.00 | 0.000320 |
| RAPH1 | 65059 | chr2 | 203527400 | 203531199 | high | 9.50 | 0.000320 |
| RARA | 5914 | chr17 | 40313600 | 40324999 | high | 28.50 | 0.000147 |
| RASA1 | 5921 | chr5 | 87324800 | 87330199 | high | 13.50 | 0.000068 |
| RASA3 | 22821 | chr13 | 114024600 | 114027999 | high | 8.50 | 0.000200 |
| RASAL2 | 9462 | chr1 | 178122800 | 178126399 | low | -15.65 | 0.000053 |
| RASAL2 | 9462 | chr1 | 178275600 | 178278599 | low | -13.04 | 0.000053 |
| RASAL2 | 9462 | chr1 | 178147200 | 178149999 | low | -12.17 | 0.000053 |
| RASAL2 | 9462 | chr1 | 178097800 | 178100199 | low | -10.43 | 0.000053 |
| RASAL2 | 9462 | chr1 | 178263800 | 178265599 | low | -7.82 | 0.000053 |
| RASAL3 | 64926 | chr19 | 15447200 | 15451799 | high | 11.50 | 0.004203 |
| RASD1 | 51655 | chr17 | 17494600 | 17497599 | high | 7.50 | 0.002953 |
| RASGRF2 | 5924 | chr5 | 81018200 | 81021999 | high | 9.50 | 0.000073 |
| RASGRF2 | 5924 | chr5 | 81071000 | 81075599 | high | 11.50 | 0.000073 |
| RASGRF2 | 5924 | chr5 | 81001200 | 81007799 | high | 16.50 | 0.000073 |
| RASGRF2 | 5924 | chr5 | 81175800 | 81182599 | high | 17.00 | 0.000073 |
| RASGRP3 | 25780 | chr2 | 33451200 | 33455799 | high | 11.50 | 0.000771 |
| RASGRP3 | 25780 | chr2 | 33517400 | 33523199 | high | 14.50 | 0.000769 |
| RASIP1 | 54922 | chr19 | 48724600 | 48731399 | high | 17.00 | 0.001127 |
| RASL11A | 387496 | chr13 | 27270000 | 27272999 | low | -13.04 | 0.014210 |
| RASSF1 | 11186 | chr3 | 50334400 | 50341799 | high | 18.50 | 0.000222 |
| RASSF3 | 283349 | chr12 | 64625600 | 64627999 | high | 6.00 | 0.004384 |
| RASSF6 | 166824 | chr4 | 73612000 | 73615799 | high | 9.50 | 0.002266 |
| RASSF8 | 11228 | chr12 | 26011800 | 26015199 | high | 8.50 | 0.000432 |
| RASSF8 | 11228 | chr12 | 26029800 | 26033799 | high | 10.00 | 0.000431 |
| RASSF8 | 11228 | chr12 | 25992200 | 25997399 | high | 13.00 | 0.000432 |
| RAVER2 | 55225 | chr1 | 64819000 | 64820399 | low | -6.08 | 0.000852 |
| RB1CC1 | 9821 | chr8 | 52712200 | 52716399 | high | 10.50 | 0.000186 |
| RBBP4 | 5928 | chr1 | 32650600 | 32652199 | high | 4.00 | 0.000182 |
| RBBP4 | 5928 | chr1 | 32676400 | 32682799 | high | 16.00 | 0.000181 |
| RBCK1 | 10616 | chr20 | 407200 | 410799 | high | 9.00 | 0.002897 |
| RBFA | 79863 | chr18 | 80030800 | 80035199 | high | 11.00 | 0.000998 |
| RBKS | 64080 | chr2 | 27890200 | 27891599 | high | 3.50 | 0.002298 |
| RBKS | 64080 | chr2 | 27789000 | 27791599 | high | 6.50 | 0.002306 |
| RBKS | 64080 | chr2 | 27846800 | 27849799 | high | 7.50 | 0.002301 |
| RBKS | 64080 | chr2 | 27798200 | 27801999 | high | 9.50 | 0.002305 |

| | | | | | | | |
|---|---|---|---|---|---|---|---|
| RBKS | 64080 | chr2 | 27808000 | 27812199 | high | 10.50 | 0.002304 |
| RBKS | 64080 | chr2 | 27814600 | 27820799 | high | 15.50 | 0.002304 |
| RBL1 | 5933 | chr20 | 37095000 | 37096599 | high | 4.00 | 0.000160 |
| RBL1 | 5933 | chr20 | 37051000 | 37054399 | high | 8.50 | 0.000160 |
| RBM10 | 8241 | chrX | 47145200 | 47146399 | high | 3.00 | 0.000175 |
| RBM14 | 10432 | chr11 | 66615800 | 66619999 | high | 10.50 | 0.000157 |
| RBM15 | 64783 | chr1 | 110339400 | 110342799 | high | 8.50 | 0.000587 |
| RBM18 | 92400 | chr9 | 122253000 | 122256599 | high | 9.00 | 0.000756 |
| RBM22 | 55696 | chr5 | 150699800 | 150701199 | high | 3.50 | 0.000370 |
| RBM23 | 55147 | chr14 | 22918000 | 22921199 | high | 8.00 | 0.002406 |
| RBM25 | 58517 | chr14 | 73107200 | 73110799 | high | 9.00 | 0.000800 |
| RBM25 | 58517 | chr14 | 73096600 | 73100799 | high | 10.50 | 0.000801 |
| RBM26 | 64062 | chr13 | 79397800 | 79402799 | high | 12.50 | 0.000807 |
| RBM27 | 54439 | chr5 | 146202200 | 146206399 | high | 10.50 | 0.000372 |
| RBM33 | 155435 | chr7 | 155644000 | 155647399 | low | -14.78 | 0.000999 |
| RBM34 | 23029 | chr1 | 235130800 | 235133599 | low | -12.17 | 0.000098 |
| RBM47 | 54502 | chr4 | 40575000 | 40577399 | high | 6.00 | 0.001343 |
| RBM8A | 9939 | chr1 | 145926000 | 145927799 | low | -7.82 | 0.000068 |
| RBMS1 | 5937 | chr2 | 160405000 | 160407999 | high | 7.50 | 0.000037 |
| RBMS1 | 5937 | chr2 | 160357000 | 160361599 | high | 11.50 | 0.000037 |
| RBMS1 | 5937 | chr2 | 160305400 | 160309999 | high | 11.50 | 0.000037 |
| RBMS1 | 5937 | chr2 | 160276000 | 160281399 | high | 13.50 | 0.000037 |
| RBMS1 | 5937 | chr2 | 160326800 | 160333599 | high | 17.00 | 0.000037 |
| RBMS1 | 5937 | chr2 | 160267000 | 160274999 | high | 20.00 | 0.000037 |
| RBMS3 | 27303 | chr3 | 29522000 | 29524999 | high | 7.50 | 0.000925 |
| RBMS3 | 27303 | chr3 | 29281800 | 29284799 | high | 7.50 | 0.000932 |
| RBMS3 | 27303 | chr3 | 29880000 | 29884399 | high | 11.00 | 0.000914 |
| RBMS3 | 27303 | chr3 | 29335600 | 29339999 | high | 11.00 | 0.000931 |
| RBMS3 | 27303 | chr3 | 29350600 | 29355599 | high | 12.50 | 0.000930 |
| RBMS3 | 27303 | chr3 | 29322200 | 29327399 | high | 13.00 | 0.000931 |
| RBPJ | 3516 | chr4 | 26422800 | 26425999 | high | 8.00 | 0.000133 |
| RBPJ | 3516 | chr4 | 26418800 | 26421999 | high | 8.00 | 0.000133 |
| RBPJ | 3516 | chr4 | 26400800 | 26405199 | high | 11.00 | 0.000133 |
| RBPJ | 3516 | chr4 | 26410200 | 26416399 | high | 15.50 | 0.000133 |
| RBPMS | 11030 | chr8 | 30524600 | 30527799 | high | 8.00 | 0.000361 |
| RBPMS | 11030 | chr8 | 30532200 | 30536799 | high | 11.50 | 0.000361 |
| RBPMS | 11030 | chr8 | 30506400 | 30511199 | high | 12.00 | 0.000362 |
| RBPMS | 11030 | chr8 | 30547000 | 30553399 | high | 16.00 | 0.000361 |
| RBPMS | 11030 | chr8 | 30537800 | 30545599 | high | 19.50 | 0.000361 |
| RBPMS | 11030 | chr8 | 30497200 | 30505399 | high | 20.50 | 0.000362 |
| RBSN | 64145 | chr3 | 15095400 | 15099599 | low | -18.18 | 0.004249 |
| RBX1 | 9978 | chr22 | 40953600 | 40956799 | high | 8.00 | 0.000244 |
| RCAN1 | 1827 | chr21 | 34550800 | 34553999 | high | 8.00 | 0.000053 |
| RCAN2 | 10231 | chr6 | 46461000 | 46462799 | low | -7.82 | 0.000220 |
| RCAN3 | 11123 | chr1 | 24501400 | 24506399 | high | 12.50 | 0.000454 |
| RCC1L | 81554 | chr7 | 75072400 | 75074399 | high | 5.00 | 0.001086 |
| RCC2 | 55920 | chr1 | 17435000 | 17441799 | high | 17.00 | 0.003207 |
| RCL1 | 10171 | chr9 | 4836600 | 4840399 | high | 9.50 | 0.002103 |
| RCL1 | 10171 | chr9 | 4852000 | 4859999 | high | 20.00 | 0.002096 |
| RCN1 | 5954 | chr11 | 32099800 | 32103599 | high | 9.50 | 0.000185 |
| RCN2 | 5955 | chr15 | 76930600 | 76932999 | high | 6.00 | 0.000077 |
| RDH5 | 5959 | chr12 | 55718400 | 55721999 | high | 9.00 | 0.000107 |
| RECK | 8434 | chr9 | 36114800 | 36119599 | high | 12.00 | 0.000234 |
| RECK | 8434 | chr9 | 36042200 | 36047199 | high | 12.50 | 0.000234 |
| RECQL | 5965 | chr12 | 21500000 | 21504399 | high | 11.00 | 0.000277 |
| RECQL5 | 9400 | chr17 | 75663600 | 75668599 | high | 12.50 | 0.000124 |
| REEP4 | 80346 | chr8 | 22136600 | 22144199 | high | 19.00 | 0.003630 |
| REEP6 | 92840 | chr19 | 1489400 | 1493199 | high | 9.50 | 0.005702 |
| REL | 5966 | chr2 | 60879200 | 60882999 | high | 9.50 | 0.000098 |
| RELA | 5970 | chr11 | 65662000 | 65663199 | low | -5.21 | 0.000091 |
| RELL1 | 768211 | chr4 | 37663800 | 37666799 | high | 7.50 | 0.020397 |
| REPIN1 | 29803 | chr7 | 150368800 | 150369599 | low | -3.47 | 0.000198 |
| REPS1 | 85021 | chr6 | 138962600 | 138965999 | high | 8.50 | 0.000612 |
| RER1 | 11079 | chr1 | 2389600 | 2392799 | high | 8.00 | 0.004636 |
| RERE | 473 | chr1 | 8386800 | 8389399 | high | 6.50 | 0.000056 |
| RERE | 473 | chr1 | 8532000 | 8535599 | high | 9.00 | 0.000055 |
| RERE | 473 | chr1 | 8592600 | 8596799 | high | 10.50 | 0.000055 |
| RERE | 473 | chr1 | 8640200 | 8644599 | high | 11.00 | 0.000055 |
| RERE | 473 | chr1 | 8406000 | 8410599 | high | 11.50 | 0.000056 |
| RERE | 473 | chr1 | 8626600 | 8631399 | high | 12.00 | 0.000055 |
| REV3L | 5980 | chr6 | 111324600 | 111328999 | high | 11.00 | 0.000054 |
| REXO4 | 57109 | chr9 | 133414600 | 133418599 | high | 10.00 | 0.000428 |
| RFC1 | 5981 | chr4 | 39344400 | 39348999 | high | 11.50 | 0.000152 |
| RFESD | 317671 | chr5 | 95649400 | 95653399 | high | 10.00 | 0.003321 |
| RFFL | 117584 | chr17 | 35087200 | 35089599 | high | 6.00 | 0.003351 |
| RFTN1 | 23180 | chr3 | 16454000 | 16458199 | high | 10.50 | 0.001409 |
| RFTN1 | 23180 | chr3 | 16414800 | 16418999 | high | 10.50 | 0.001412 |
| RFWD2 | 64326 | chr1 | 176204600 | 176207799 | high | 8.00 | 0.000365 |

| | | | | | | | |
|---|---|---|---|---|---|---|---|
| RFWD3 | 55159 | chr16 | 74627000 | 74629799 | low | -12.17 | 0.000739 |
| RFX1 | 5989 | chr19 | 13977400 | 13981799 | high | 11.00 | 0.000428 |
| RFX2 | 5990 | chr19 | 6106600 | 6111799 | high | 13.00 | 0.000981 |
| RFX3 | 5991 | chr9 | 3288800 | 3292599 | low | -16.52 | 0.001822 |
| RFX3 | 5991 | chr9 | 3247200 | 3249999 | low | -12.17 | 0.001845 |
| RFX3 | 5991 | chr9 | 3524800 | 3527199 | low | -10.43 | 0.001700 |
| RFX3 | 5991 | chr9 | 3450200 | 3452199 | low | -8.69 | 0.001736 |
| RFX3 | 5991 | chr9 | 3471600 | 3473399 | low | -7.82 | 0.001726 |
| RFX5 | 5993 | chr1 | 151345600 | 151347399 | high | 4.50 | 0.000040 |
| RFX7 | 64864 | chr15 | 56168600 | 56172199 | high | 9.00 | 0.001155 |
| RFX7 | 64864 | chr15 | 56240800 | 56244999 | high | 10.50 | 0.001153 |
| RFX7 | 64864 | chr15 | 56163000 | 56167599 | high | 11.50 | 0.001155 |
| RFX7 | 64864 | chr15 | 56189000 | 56194399 | high | 13.50 | 0.001154 |
| RFX7 | 64864 | chr15 | 56215200 | 56221599 | high | 16.00 | 0.001154 |
| RGCC | 28984 | chr13 | 41459000 | 41460799 | high | 4.50 | 0.000699 |
| RGL1 | 23179 | chr1 | 183632800 | 183637599 | high | 12.00 | 0.000126 |
| RGL1 | 23179 | chr1 | 183841200 | 183846399 | high | 13.00 | 0.000126 |
| RGL1 | 23179 | chr1 | 183831600 | 183836999 | high | 13.50 | 0.000126 |
| RGL1 | 23179 | chr1 | 183819000 | 183824599 | high | 14.00 | 0.000126 |
| RGL1 | 23179 | chr1 | 183803800 | 183809599 | high | 14.50 | 0.000126 |
| RGL1 | 23179 | chr1 | 183884400 | 183890399 | high | 15.00 | 0.000126 |
| RGL3 | 57139 | chr19 | 11419000 | 11423599 | high | 11.50 | 0.005004 |
| RGMB | 285704 | chr5 | 98784400 | 98786799 | high | 6.00 | 0.002892 |
| RGS10 | 6001 | chr10 | 119511000 | 119513999 | low | -13.04 | 0.000050 |
| RGS13 | 6003 | chr1 | 192637600 | 192640999 | high | 8.50 | 0.000031 |
| RGS18 | 64407 | chr1 | 192172200 | 192177199 | high | 12.50 | 0.000335 |
| RGS2 | 5997 | chr1 | 192807200 | 192811599 | high | 11.00 | 0.000031 |
| RGS20 | 8601 | chr8 | 53955000 | 53959199 | high | 10.50 | 0.000159 |
| RGS20 | 8601 | chr8 | 53878400 | 53882999 | high | 11.50 | 0.000160 |
| RGS21 | 431704 | chr1 | 192363400 | 192367199 | high | 9.50 | 0.002244 |
| RGS3 | 5998 | chr9 | 113474400 | 113477999 | high | 9.00 | 0.000053 |
| RGS3 | 5998 | chr9 | 113535800 | 113539799 | high | 10.00 | 0.000053 |
| RGS3 | 5998 | chr9 | 113461000 | 113465599 | high | 11.50 | 0.000053 |
| RGS3 | 5998 | chr9 | 113552600 | 113557799 | high | 13.00 | 0.000053 |
| RGS3 | 5998 | chr9 | 113573600 | 113579799 | high | 15.50 | 0.000053 |
| RGS4 | 5999 | chr1 | 163069400 | 163073799 | high | 11.00 | 0.000037 |
| RGS5 | 8490 | chr1 | 163230400 | 163232599 | high | 5.50 | 0.000052 |
| RGS5 | 8490 | chr1 | 163177000 | 163180599 | high | 9.00 | 0.000052 |
| RGS5 | 8490 | chr1 | 163146600 | 163151799 | high | 13.00 | 0.000052 |
| RGS5 | 8490 | chr1 | 163263400 | 163269799 | high | 16.00 | 0.000052 |
| RGS6 | 9628 | chr14 | 72525200 | 72529799 | high | 11.50 | 0.000133 |
| RGS9 | 8787 | chr17 | 65183600 | 65188399 | high | 12.00 | 0.000135 |
| RGS9 | 8787 | chr17 | 65189800 | 65194999 | high | 13.00 | 0.000135 |
| RHBDD1 | 84236 | chr2 | 226908200 | 226912799 | high | 11.50 | 0.000371 |
| RHBDF2 | 79651 | chr17 | 76498200 | 76502599 | high | 11.00 | 0.001041 |
| RHEB | 6009 | chr7 | 151517400 | 151521199 | high | 9.50 | 0.000040 |
| RHEB | 6009 | chr7 | 151503400 | 151507599 | high | 10.50 | 0.000040 |
| RHEB | 6009 | chr7 | 151485400 | 151489799 | high | 11.00 | 0.000040 |
| RHEB | 6009 | chr7 | 151508400 | 151516399 | high | 20.00 | 0.000040 |
| RHNO1 | 83695 | chr12 | 2889400 | 2893799 | high | 11.00 | 0.003218 |
| RHO | 6010 | chr3 | 129529200 | 129534399 | high | 13.00 | 0.000046 |
| RHOA | 387 | chr3 | 49401800 | 49405399 | high | 9.00 | 0.000008 |
| RHOA | 387 | chr3 | 49409400 | 49413399 | high | 10.00 | 0.000008 |
| RHOB | 388 | chr2 | 20446800 | 20448599 | low | -7.82 | 0.000019 |
| RHOC | 389 | chr1 | 112703800 | 112708199 | high | 11.00 | 0.000003 |
| RHOF | 54509 | chr12 | 121791800 | 121794999 | high | 8.00 | 0.000448 |
| RHOQ | 23433 | chr2 | 46541200 | 46544399 | high | 8.00 | 0.000503 |
| RHOQ | 23433 | chr2 | 46566400 | 46570799 | high | 11.00 | 0.000503 |
| RHOT1 | 55288 | chr17 | 32157800 | 32162999 | high | 13.00 | 0.001719 |
| RHOT1 | 55288 | chr17 | 32188400 | 32194799 | high | 16.00 | 0.001718 |
| RHOT2 | 89941 | chr16 | 665000 | 671399 | high | 16.00 | 0.002496 |
| RIBC1 | 158787 | chrX | 53422600 | 53425199 | low | -11.30 | 0.002972 |
| RIC1 | 57589 | chr9 | 5752000 | 5754399 | high | 6.00 | 0.010012 |
| RIC1 | 57589 | chr9 | 5628000 | 5630599 | high | 6.50 | 0.010233 |
| RIC1 | 57589 | chr9 | 5698000 | 5702599 | high | 11.50 | 0.010107 |
| RILPL1 | 353116 | chr12 | 123478000 | 123481599 | high | 9.00 | 0.002860 |
| RILPL1 | 353116 | chr12 | 123466000 | 123471999 | high | 15.00 | 0.002860 |
| RILPL2 | 196383 | chr12 | 123434800 | 123437199 | high | 6.00 | 0.001591 |
| RIMS2 | 9699 | chr8 | 103515600 | 103517599 | low | -8.69 | 0.000094 |
| RIN1 | 9610 | chr11 | 66334000 | 66336599 | high | 6.50 | 0.000145 |
| RIN2 | 54453 | chr20 | 19895600 | 19899399 | high | 9.50 | 0.002737 |
| RIN2 | 54453 | chr20 | 19904800 | 19909399 | high | 11.50 | 0.002736 |
| RINT1 | 60561 | chr7 | 105530600 | 105537199 | low | -28.13 | 0.000574 |
| RIOK1 | 83732 | chr6 | 7395600 | 7398599 | low | -13.04 | 0.011322 |
| RIOK2 | 55781 | chr5 | 97181800 | 97183999 | low | -9.56 | 0.000574 |
| RIPK1 | 8737 | chr6 | 3110200 | 3114599 | high | 11.00 | 0.002809 |
| RIPK1 | 8737 | chr6 | 3079600 | 3083999 | high | 11.00 | 0.002837 |
| RLBP1 | 6017 | chr15 | 89213600 | 89216399 | low | -12.17 | 0.000067 |

| | | | | | | | |
|---|---|---|---|---|---|---|---|
| RLF | 6018 | chr1 | 40161200 | 40164999 | high | 9.50 | 0.000150 |
| RLF | 6018 | chr1 | 40206400 | 40212199 | high | 14.50 | 0.000150 |
| RMDN1 | 51115 | chr8 | 86506400 | 86515399 | high | 22.50 | 0.000591 |
| RMDN2 | 151393 | chr2 | 38053600 | 38057799 | high | 10.50 | 0.003978 |
| RMI1 | 80010 | chr9 | 83977200 | 83983799 | high | 16.50 | 0.000953 |
| RMRP | 6023 | chr9 | 35658000 | 35659799 | low | -7.82 | 0.000169 |
| RN7SK | 125050 | chr6 | 52993000 | 52996799 | low | -16.52 | 0.002360 |
| RND3 | 390 | chr2 | 150471800 | 150474799 | high | 7.50 | 0.000003 |
| RNF10 | 9921 | chr12 | 120534000 | 120542399 | high | 21.00 | 0.000082 |
| RNF11 | 26994 | chr1 | 51236200 | 51238599 | low | -10.43 | 0.000527 |
| RNF114 | 55905 | chr20 | 49936000 | 49938599 | low | -11.30 | 0.001120 |
| RNF115 | 27246 | chr1 | 145768400 | 145772399 | high | 10.00 | 0.000187 |
| RNF121 | 55298 | chr11 | 71987800 | 71991199 | high | 8.50 | 0.000768 |
| RNF121 | 55298 | chr11 | 71952400 | 71956799 | high | 11.00 | 0.000769 |
| RNF123 | 63891 | chr3 | 49720800 | 49724599 | high | 9.50 | 0.001285 |
| RNF13 | 11342 | chr3 | 149847200 | 149851199 | high | 10.00 | 0.000076 |
| RNF13 | 11342 | chr3 | 149923800 | 149928199 | high | 11.00 | 0.000076 |
| RNF13 | 11342 | chr3 | 149824800 | 149829199 | high | 11.00 | 0.000076 |
| RNF13 | 11342 | chr3 | 149929000 | 149933999 | high | 12.50 | 0.000076 |
| RNF13 | 11342 | chr3 | 149894000 | 149899199 | high | 13.00 | 0.000076 |
| RNF13 | 11342 | chr3 | 149879000 | 149884999 | high | 15.00 | 0.000076 |
| RNF14 | 9604 | chr5 | 141982600 | 141988999 | high | 16.00 | 0.000068 |
| RNF141 | 50862 | chr11 | 10522200 | 10528999 | high | 17.00 | 0.004834 |
| RNF145 | 153830 | chr5 | 159204400 | 159211199 | high | 17.00 | 0.000966 |
| RNF149 | 284996 | chr2 | 101292400 | 101296999 | high | 11.50 | 0.002814 |
| RNF149 | 284996 | chr2 | 101305200 | 101310399 | high | 13.00 | 0.002813 |
| RNF150 | 57484 | chr4 | 141129400 | 141133999 | high | 11.50 | 0.000407 |
| RNF157 | 114804 | chr17 | 76138800 | 76142999 | high | 10.50 | 0.001508 |
| RNF157 | 114804 | chr17 | 76231400 | 76237799 | high | 16.00 | 0.001506 |
| RNF167 | 26001 | chr17 | 4939800 | 4942999 | low | -13.91 | 0.005264 |
| RNF169 | 254225 | chr11 | 74835200 | 74837199 | low | -8.69 | 0.003397 |
| RNF170 | 81790 | chr8 | 42886200 | 42889999 | high | 9.50 | 0.001907 |
| RNF181 | 51255 | chr2 | 85595200 | 85597999 | high | 7.00 | 0.000599 |
| RNF185 | 91445 | chr22 | 31192000 | 31196199 | high | 10.50 | 0.002932 |
| RNF19A | 25897 | chr8 | 100280400 | 100285799 | high | 13.50 | 0.000258 |
| RNF19B | 127544 | chr1 | 32935400 | 32940799 | high | 13.50 | 0.003873 |
| RNF2 | 6045 | chr1 | 185091200 | 185098799 | high | 19.00 | 0.000033 |
| RNF213 | 57674 | chr17 | 80376000 | 80382599 | high | 16.50 | 0.000718 |
| RNF214 | 257160 | chr11 | 117236200 | 117239999 | high | 9.50 | 0.002194 |
| RNF215 | 200312 | chr22 | 30379200 | 30380199 | low | -4.34 | 0.006594 |
| RNF216 | 54476 | chr7 | 5686800 | 5690199 | high | 8.50 | 0.009579 |
| RNF216 | 54476 | chr7 | 5741800 | 5745799 | high | 10.00 | 0.009488 |
| RNF220 | 55182 | chr1 | 44609400 | 44613799 | high | 11.00 | 0.001237 |
| RNF24 | 11237 | chr20 | 3970200 | 3973599 | high | 8.50 | 0.002830 |
| RNF24 | 11237 | chr20 | 3943600 | 3947599 | high | 10.00 | 0.002849 |
| RNF24 | 11237 | chr20 | 4009200 | 4015599 | high | 16.00 | 0.002803 |
| RNF25 | 64320 | chr2 | 218659200 | 218663999 | low | -20.23 | 0.000294 |
| RNF32 | 140545 | chr7 | 156638800 | 156642599 | high | 9.50 | 0.000897 |
| RNF34 | 80196 | chr12 | 121415200 | 121419999 | high | 12.00 | 0.000661 |
| RNF38 | 152006 | chr9 | 36387600 | 36389999 | low | -10.43 | 0.004177 |
| RNF38 | 152006 | chr9 | 36400000 | 36401199 | low | -5.21 | 0.004176 |
| RNF4 | 6047 | chr4 | 2468600 | 2469799 | low | -5.21 | 0.002450 |
| RNF40 | 9810 | chr16 | 30759400 | 30764399 | high | 12.50 | 0.000319 |
| RNF40 | 9810 | chr16 | 30773200 | 30779199 | high | 15.00 | 0.000319 |
| RNF41 | 10193 | chr12 | 56220000 | 56221799 | low | -7.82 | 0.000181 |
| RNFT1 | 51136 | chr17 | 59963000 | 59964799 | low | -7.82 | 0.000853 |
| RNGTT | 8732 | chr6 | 88856800 | 88858999 | low | -9.56 | 0.000098 |
| RNGTT | 8732 | chr6 | 88849600 | 88851399 | low | -7.82 | 0.000098 |
| RNLS | 55328 | chr10 | 88400000 | 88404199 | high | 10.50 | 0.000626 |
| RNLS | 55328 | chr10 | 88433800 | 88438999 | high | 13.00 | 0.000626 |
| RNLS | 55328 | chr10 | 88475400 | 88480799 | high | 13.50 | 0.000625 |
| RNLS | 55328 | chr10 | 88491200 | 88498999 | high | 19.50 | 0.000625 |
| RNPEP | 6051 | chr1 | 201982400 | 201985999 | high | 9.00 | 0.000030 |
| RNPS1 | 10921 | chr16 | 2267400 | 2270399 | high | 7.50 | 0.004817 |
| RP9 | 6100 | chr7 | 33107200 | 33109799 | high | 6.50 | 0.000184 |
| RP9P | 441212 | chr7 | 32941200 | 32943399 | low | -9.56 | 0.013394 |
| RPAIN | 84268 | chr17 | 5418600 | 5423199 | high | 11.50 | 0.015552 |
| RPAP2 | 79871 | chr1 | 92319000 | 92321799 | low | -12.17 | 0.000865 |
| RPAP2 | 79871 | chr1 | 92297400 | 92300199 | low | -12.17 | 0.000865 |
| RPAP2 | 79871 | chr1 | 92328400 | 92330199 | low | -7.82 | 0.000865 |
| RPAP3 | 79657 | chr12 | 47699200 | 47700999 | high | 4.50 | 0.001670 |
| RPF1 | 80135 | chr1 | 84485400 | 84488599 | high | 8.00 | 0.000949 |
| RPF1 | 80135 | chr1 | 84491800 | 84495599 | high | 9.50 | 0.000948 |
| RPF2 | 84154 | chr6 | 110981200 | 110985199 | high | 10.00 | 0.000758 |
| RPL13 | 6137 | chr16 | 89559400 | 89566999 | high | 19.00 | 0.000069 |
| RPL17 | 6139 | chr18 | 49489600 | 49492599 | high | 7.50 | 0.000124 |
| RPL21 | 6144 | chr13 | 27251000 | 27252799 | high | 4.50 | 0.000225 |
| RPL22 | 6146 | chr1 | 6198600 | 6200599 | low | -8.69 | 0.000992 |

| | | | | | | | |
|---|---|---|---|---|---|---|---|
| RPL23A | 6147 | chr17 | 28719400 | 28720599 | low | -5.21 | 0.000214 |
| RPL27 | 6155 | chr17 | 42998200 | 42999799 | low | -6.95 | 0.000143 |
| RPL28 | 6158 | chr19 | 55383400 | 55389199 | high | 14.50 | 0.000111 |
| RPL3 | 6122 | chr22 | 39318200 | 39319999 | low | -7.82 | 0.000156 |
| RPL36 | 25873 | chr19 | 5689800 | 5691999 | low | -9.56 | 0.004547 |
| RPL37 | 6167 | chr5 | 40832800 | 40836599 | high | 9.50 | 0.000151 |
| RPL5 | 6125 | chr1 | 92839600 | 92843199 | high | 9.00 | 0.000066 |
| RPL7 | 6129 | chr8 | 73292800 | 73294399 | low | -6.95 | 0.000084 |
| RPLP0 | 6175 | chr12 | 120199400 | 120202399 | high | 7.50 | 0.000051 |
| RPN1 | 6184 | chr3 | 128648000 | 128651399 | high | 8.50 | 0.000048 |
| RPP14 | 11102 | chr3 | 58305800 | 58309599 | high | 9.50 | 0.000190 |
| RPP38 | 10557 | chr10 | 15097200 | 15098799 | low | -6.95 | 0.000699 |
| RPRD1A | 55197 | chr18 | 36023800 | 36029599 | high | 14.50 | 0.001532 |
| RPRD1B | 58490 | chr20 | 38050000 | 38056399 | high | 16.00 | 0.001537 |
| RPRD1B | 58490 | chr20 | 38081400 | 38089399 | high | 20.00 | 0.001536 |
| RPRD2 | 23248 | chr1 | 150418400 | 150422199 | high | 9.50 | 0.000155 |
| RPS10 | 6204 | chr6 | 34414800 | 34419199 | high | 11.00 | 0.000180 |
| RPS11 | 6205 | chr19 | 49495800 | 49498199 | high | 6.00 | 0.000125 |
| RPS12 | 6206 | chr6 | 132814000 | 132815799 | low | -7.82 | 0.000047 |
| RPS13 | 6207 | chr11 | 17075600 | 17078599 | low | -13.04 | 0.000364 |
| RPS14 | 6208 | chr5 | 150448200 | 150449799 | low | -6.95 | 0.000041 |
| RPS15 | 6209 | chr19 | 1437800 | 1439999 | low | -9.56 | 0.004318 |
| RPS15A | 6210 | chr16 | 18789000 | 18790599 | low | -6.95 | 0.000331 |
| RPS2 | 6187 | chr16 | 1963000 | 1965799 | low | -12.17 | 0.003152 |
| RPS21 | 6227 | chr20 | 62386200 | 62388599 | high | 6.00 | 0.000100 |
| RPS23 | 6228 | chr5 | 82274000 | 82278199 | high | 10.50 | 0.000076 |
| RPS26 | 6231 | chr12 | 56042200 | 56043399 | low | -5.21 | 0.000111 |
| RPS27 | 6232 | chr1 | 153990400 | 153992999 | low | -11.30 | 0.000040 |
| RPS27L | 51065 | chr15 | 63153200 | 63157999 | high | 12.00 | 0.000809 |
| RPS28 | 6234 | chr19 | 8319000 | 8321799 | high | 7.00 | 0.000749 |
| RPS3 | 6188 | chr11 | 75399200 | 75400799 | low | -6.95 | 0.000082 |
| RPS3A | 6189 | chr4 | 151099200 | 151100599 | low | -6.08 | 0.000041 |
| RPS6 | 6194 | chr9 | 19376800 | 19380599 | low | -16.52 | 0.000320 |
| RPS7 | 6201 | chr2 | 3574400 | 3576199 | high | 4.50 | 0.001735 |
| RPS8 | 6202 | chr1 | 44776200 | 44777799 | low | -6.95 | 0.000139 |
| RPTOR | 57521 | chr17 | 80575200 | 80578399 | high | 8.00 | 0.000714 |
| RPTOR | 57521 | chr17 | 80864400 | 80867999 | high | 9.00 | 0.000711 |
| RPTOR | 57521 | chr17 | 80715800 | 80719399 | high | 9.00 | 0.000713 |
| RPTOR | 57521 | chr17 | 80543800 | 80547399 | high | 9.00 | 0.000714 |
| RPTOR | 57521 | chr17 | 80889200 | 80893399 | high | 10.50 | 0.000711 |
| RPTOR | 57521 | chr17 | 80811200 | 80815399 | high | 10.50 | 0.000712 |
| RPTOR | 57521 | chr17 | 80637600 | 80641799 | high | 10.50 | 0.000713 |
| RPTOR | 57521 | chr17 | 80555000 | 80560399 | high | 13.50 | 0.000714 |
| RPTOR | 57521 | chr17 | 80849000 | 80854999 | high | 15.00 | 0.000711 |
| RPTOR | 57521 | chr17 | 80786600 | 80792599 | high | 15.00 | 0.000712 |
| RPTOR | 57521 | chr17 | 80653400 | 80659399 | high | 15.00 | 0.000713 |
| RPTOR | 57521 | chr17 | 80770800 | 80776999 | high | 15.50 | 0.000712 |
| RPTOR | 57521 | chr17 | 80924400 | 80931199 | high | 17.00 | 0.000711 |
| RPTOR | 57521 | chr17 | 80817600 | 80830399 | high | 32.00 | 0.000712 |
| RPUSD2 | 27079 | chr15 | 40569000 | 40571399 | low | -10.43 | 0.000667 |
| RPUSD3 | 285367 | chr3 | 9843000 | 9844999 | high | 5.00 | 0.003221 |
| RRAS2 | 22800 | chr11 | 14294600 | 14298599 | high | 10.00 | 0.001595 |
| RRAS2 | 22800 | chr11 | 14304600 | 14310399 | high | 14.50 | 0.001594 |
| RRBP1 | 6238 | chr20 | 17659400 | 17664399 | high | 12.50 | 0.000353 |
| RRBP1 | 6238 | chr20 | 17644600 | 17658599 | high | 35.00 | 0.000354 |
| RRM1 | 6240 | chr11 | 4094000 | 4095599 | low | -6.95 | 0.001524 |
| RRP1 | 8568 | chr21 | 43788000 | 43791199 | high | 8.00 | 0.000196 |
| RRP1B | 23076 | chr21 | 43668600 | 43673199 | high | 11.50 | 0.000528 |
| RRP36 | 88745 | chr6 | 43020800 | 43023399 | high | 6.50 | 0.002063 |
| RRP9 | 9136 | chr3 | 51941200 | 51945199 | high | 10.00 | 0.000176 |
| RSAD1 | 55316 | chr17 | 50478000 | 50479599 | high | 4.00 | 0.001096 |
| RSBN1 | 54665 | chr1 | 113801800 | 113805999 | high | 10.50 | 0.000480 |
| RSBN1 | 54665 | chr1 | 113795000 | 113800799 | high | 14.50 | 0.000480 |
| RSF1 | 51773 | chr11 | 77712400 | 77716999 | high | 11.50 | 0.000666 |
| RSG1 | 79363 | chr1 | 16234400 | 16237999 | high | 9.00 | 0.004889 |
| RSRC1 | 51319 | chr3 | 158213000 | 158215999 | low | -13.04 | 0.000324 |
| RSRC1 | 51319 | chr3 | 158534200 | 158536799 | low | -11.30 | 0.000324 |
| RSRC1 | 51319 | chr3 | 158263600 | 158265999 | low | -10.43 | 0.000324 |
| RSRC2 | 65117 | chr12 | 122518000 | 122522999 | high | 12.50 | 0.000531 |
| RTCA | 8634 | chr1 | 100264800 | 100266799 | high | 5.00 | 0.000086 |
| RTEL1 | 51750 | chr20 | 63695800 | 63699399 | high | 9.00 | 0.000812 |
| RTEL1 | 51750 | chr20 | 63681200 | 63687199 | high | 15.00 | 0.000813 |
| RTF1 | 23168 | chr15 | 41423800 | 41426599 | high | 7.00 | 0.000559 |
| RTN3 | 10313 | chr11 | 63706400 | 63711599 | high | 13.00 | 0.000162 |
| RTN4 | 57142 | chr2 | 55005000 | 55009399 | low | -19.27 | 0.001039 |
| RTN4 | 57142 | chr2 | 55048200 | 55051399 | low | -13.91 | 0.001038 |
| RTN4 | 57142 | chr2 | 55040400 | 55042999 | low | -11.30 | 0.001038 |
| RTN4 | 57142 | chr2 | 55010600 | 55013199 | low | -11.30 | 0.001039 |

| | | | | | | | |
|---|---|---|---|---|---|---|---|
| RTN4 | 57142 | chr2 | 54975400 | 54977799 | low | -10.43 | 0.001039 |
| RTN4 | 57142 | chr2 | 54994000 | 54995999 | low | -8.69 | 0.001039 |
| RTTN | 25914 | chr18 | 70127800 | 70132599 | low | -20.43 | 0.000370 |
| RTTN | 25914 | chr18 | 70036800 | 70038999 | low | -9.56 | 0.000370 |
| RUBCN | 9711 | chr3 | 197691000 | 197694799 | high | 9.50 | 0.000049 |
| RUFY1 | 80230 | chr5 | 179557800 | 179560199 | low | -10.43 | 0.000447 |
| RUNX1 | 861 | chr21 | 34986800 | 34989799 | high | 7.50 | 0.000025 |
| RUNX1 | 861 | chr21 | 35045800 | 35050799 | high | 12.50 | 0.000025 |
| RUNX1 | 861 | chr21 | 35033600 | 35038599 | high | 12.50 | 0.000025 |
| RUNX1 | 861 | chr21 | 34850400 | 34855399 | high | 12.50 | 0.000025 |
| RUNX1 | 861 | chr21 | 34792600 | 34797599 | high | 12.50 | 0.000025 |
| RUNX1 | 861 | chr21 | 35000600 | 35005799 | high | 13.00 | 0.000025 |
| RUNX1 | 861 | chr21 | 34820400 | 34825599 | high | 13.00 | 0.000025 |
| RUNX2 | 860 | chr6 | 45451200 | 45453199 | high | 8.69 | 0.000019 |
| RUNX2 | 860 | chr6 | 45404200 | 45406199 | high | 8.69 | 0.000019 |
| RUNX2 | 860 | chr6 | 45327400 | 45330799 | high | 14.78 | 0.000019 |
| RUNX2 | 860 | chr6 | 45471800 | 45475399 | high | 15.65 | 0.000019 |
| RUNX3 | 864 | chr1 | 24928800 | 24933599 | high | 12.00 | 0.000035 |
| RUNX3 | 864 | chr1 | 24945600 | 24950599 | high | 12.50 | 0.000035 |
| RUSC2 | 9853 | chr9 | 35489400 | 35491599 | high | 5.50 | 0.000278 |
| RUSC2 | 9853 | chr9 | 35537600 | 35540599 | high | 7.50 | 0.000277 |
| RUSC2 | 9853 | chr9 | 35541600 | 35545199 | high | 9.00 | 0.000277 |
| RUSC2 | 9853 | chr9 | 35513000 | 35519199 | high | 15.50 | 0.000277 |
| RUVBL1 | 8607 | chr3 | 128093400 | 128096599 | high | 8.00 | 0.000067 |
| RUVBL1 | 8607 | chr3 | 128061600 | 128066199 | high | 11.50 | 0.000067 |
| RUVBL1 | 8607 | chr3 | 128108400 | 128113399 | high | 12.50 | 0.000067 |
| RWDD1 | 51389 | chr6 | 116570800 | 116573199 | low | -10.43 | 0.000441 |
| RWDD2A | 112611 | chr6 | 83190200 | 83195199 | high | 12.50 | 0.001354 |
| RWDD3 | 25950 | chr1 | 95232800 | 95240399 | high | 19.00 | 0.000272 |
| RXFP2 | 122042 | chr13 | 31747600 | 31750999 | low | -14.78 | 0.003844 |
| RYK | 6259 | chr3 | 134237000 | 134240199 | high | 8.00 | 0.000047 |
| S100A11 | 6282 | chr1 | 152034200 | 152036999 | low | -12.17 | 0.000041 |
| S100A5 | 6276 | chr1 | 153539200 | 153542199 | high | 7.50 | 0.000041 |
| S100PBP | 64766 | chr1 | 32826200 | 32829799 | high | 9.00 | 0.001973 |
| S1PR1 | 1901 | chr1 | 101235600 | 101237399 | low | -7.82 | 0.000019 |
| S1PR3 | 1903 | chr9 | 88991200 | 88993399 | low | -9.56 | 0.000021 |
| SAC3D1 | 29901 | chr11 | 65039800 | 65043199 | high | 8.50 | 0.000460 |
| SACM1L | 22908 | chr3 | 45689200 | 45691399 | high | 5.50 | 0.000501 |
| SACM1L | 22908 | chr3 | 45742000 | 45747799 | high | 14.50 | 0.000501 |
| SACS | 26278 | chr13 | 23415600 | 23419199 | high | 9.00 | 0.001122 |
| SACS | 26278 | chr13 | 23372600 | 23377199 | high | 11.50 | 0.001124 |
| SAE1 | 10055 | chr19 | 47165600 | 47167399 | low | -7.82 | 0.000213 |
| SAE1 | 10055 | chr19 | 47142400 | 47144199 | low | -7.82 | 0.000213 |
| SAFB | 6294 | chr19 | 5644800 | 5648799 | high | 10.00 | 0.001115 |
| SAFB2 | 9667 | chr19 | 5585400 | 5588999 | high | 9.00 | 0.001731 |
| SAP18 | 10284 | chr13 | 21140000 | 21141199 | low | -5.21 | 0.000486 |
| SAP30 | 8819 | chr4 | 173369400 | 173372599 | high | 8.00 | 0.000051 |
| SAR1A | 56681 | chr10 | 70168400 | 70170799 | high | 6.00 | 0.000808 |
| SARNP | 84324 | chr12 | 55758400 | 55760799 | high | 6.00 | 0.001512 |
| SARNP | 84324 | chr12 | 55804600 | 55808199 | high | 9.00 | 0.001511 |
| SARNP | 84324 | chr12 | 55817200 | 55822199 | high | 12.50 | 0.001511 |
| SARNP | 84324 | chr12 | 55742800 | 55753199 | high | 26.00 | 0.001513 |
| SARS2 | 54938 | chr19 | 38928200 | 38932999 | low | -20.34 | 0.001411 |
| SART3 | 9733 | chr12 | 108553000 | 108568199 | high | 38.00 | 0.000090 |
| SASS6 | 163786 | chr1 | 100105600 | 100107999 | high | 6.00 | 0.001636 |
| SAT1 | 6303 | chrX | 23782000 | 23785199 | high | 8.00 | 0.000265 |
| SAV1 | 60485 | chr14 | 50660800 | 50664199 | high | 8.50 | 0.001194 |
| SBF1 | 6305 | chr22 | 50466600 | 50470399 | high | 9.50 | 0.000125 |
| SBF1 | 6305 | chr22 | 50443400 | 50448399 | high | 12.50 | 0.000125 |
| SBF2 | 81846 | chr11 | 10118600 | 10121599 | high | 7.50 | 0.008089 |
| SBF2 | 81846 | chr11 | 9983200 | 9986399 | high | 8.00 | 0.008198 |
| SBF2 | 81846 | chr11 | 10049000 | 10052599 | high | 9.00 | 0.008145 |
| SBF2 | 81846 | chr11 | 10205000 | 10209399 | high | 11.00 | 0.008020 |
| SBF2 | 81846 | chr11 | 9943200 | 9948399 | high | 13.00 | 0.008231 |
| SBF2 | 81846 | chr11 | 9773400 | 9779399 | high | 15.00 | 0.008374 |
| SBF2 | 81846 | chr11 | 9918400 | 9929599 | high | 28.00 | 0.008252 |
| SBF2 | 81846 | chr11 | 10236000 | 10247599 | high | 29.00 | 0.007996 |
| SBNO1 | 55206 | chr12 | 123312200 | 123314999 | low | -12.17 | 0.000448 |
| SCAF1 | 58506 | chr19 | 49658400 | 49663199 | high | 12.00 | 0.001178 |
| SCAF11 | 9169 | chr12 | 45948600 | 45950799 | high | 5.50 | 0.000200 |
| SCAF8 | 22828 | chr6 | 154732800 | 154734999 | low | -9.56 | 0.000148 |
| SCAF8 | 22828 | chr6 | 154833200 | 154834999 | low | -7.82 | 0.000147 |
| SCAI | 286205 | chr9 | 124940600 | 124944799 | high | 10.50 | 0.002291 |
| SCAI | 286205 | chr9 | 125095200 | 125101799 | high | 16.50 | 0.002288 |
| SCFD1 | 23256 | chr14 | 30644200 | 30646599 | high | 6.00 | 0.000759 |
| SCFD2 | 152579 | chr4 | 53315000 | 53317799 | high | 7.00 | 0.002862 |
| SCFD2 | 152579 | chr4 | 53018200 | 53021199 | high | 7.50 | 0.002878 |
| SCFD2 | 152579 | chr4 | 53186400 | 53189599 | high | 8.00 | 0.002869 |

| | | | | | | | |
|---|---|---|---|---|---|---|---|
| SCFD2 | 152579 | chr4 | 53152600 | 53155799 | high | 8.00 | 0.002871 |
| SCFD2 | 152579 | chr4 | 53191200 | 53194599 | high | 8.50 | 0.002869 |
| SCFD2 | 152579 | chr4 | 53125800 | 53129199 | high | 8.50 | 0.002872 |
| SCFD2 | 152579 | chr4 | 52912000 | 52915799 | high | 9.50 | 0.002884 |
| SCFD2 | 152579 | chr4 | 53267800 | 53271799 | high | 10.00 | 0.002864 |
| SCFD2 | 152579 | chr4 | 52933400 | 52937799 | high | 11.00 | 0.002882 |
| SCFD2 | 152579 | chr4 | 52998000 | 53002599 | high | 11.50 | 0.002879 |
| SCFD2 | 152579 | chr4 | 53160200 | 53165599 | high | 13.50 | 0.002870 |
| SCFD2 | 152579 | chr4 | 53060000 | 53065599 | high | 14.00 | 0.002876 |
| SCFD2 | 152579 | chr4 | 53274200 | 53280399 | high | 15.50 | 0.002864 |
| SCFD2 | 152579 | chr4 | 53037600 | 53043799 | high | 15.50 | 0.002877 |
| SCFD2 | 152579 | chr4 | 53141800 | 53148199 | high | 16.00 | 0.002871 |
| SCFD2 | 152579 | chr4 | 53045600 | 53057799 | high | 30.50 | 0.002876 |
| SCLT1 | 132320 | chr4 | 129088200 | 129094399 | high | 15.50 | 0.001025 |
| SCLY | 51540 | chr2 | 238060600 | 238063999 | high | 8.50 | 0.000216 |
| SCLY | 51540 | chr2 | 238080600 | 238085199 | high | 11.50 | 0.000216 |
| SCN7A | 6332 | chr2 | 166427800 | 166430399 | low | -11.30 | 0.000038 |
| SCN7A | 6332 | chr2 | 166403600 | 166405799 | low | -9.56 | 0.000038 |
| SCN9A | 6335 | chr2 | 166266200 | 166268999 | low | -12.17 | 0.000038 |
| SCN9A | 6335 | chr2 | 166216600 | 166218599 | low | -8.69 | 0.000038 |
| SCN9A | 6335 | chr2 | 166273800 | 166275799 | high | 5.00 | 0.000038 |
| SCNN1A | 6337 | chr12 | 6374200 | 6376399 | high | 5.50 | 0.000994 |
| SCNN1A | 6337 | chr12 | 6344800 | 6348999 | high | 10.50 | 0.000999 |
| SCO2 | 9997 | chr22 | 50523000 | 50526399 | low | -14.78 | 0.000198 |
| SCOC | 60592 | chr4 | 140277400 | 140281199 | high | 9.50 | 0.000432 |
| SCOC | 60592 | chr4 | 140322400 | 140327799 | high | 13.50 | 0.000432 |
| SCOC | 60592 | chr4 | 140267600 | 140273399 | high | 14.50 | 0.000432 |
| SCP2 | 6342 | chr1 | 53046600 | 53049799 | high | 8.00 | 0.000120 |
| SCRG1 | 11341 | chr4 | 173388600 | 173391799 | low | -13.91 | 0.000065 |
| SCRG1 | 11341 | chr4 | 173394400 | 173397399 | low | -13.04 | 0.000065 |
| SCRN1 | 9805 | chr7 | 29985600 | 29990199 | high | 11.50 | 0.000327 |
| SCRN2 | 90507 | chr17 | 47838600 | 47842599 | low | -17.10 | 0.001892 |
| SCRT1 | 83482 | chr8 | 144330400 | 144334599 | high | 10.50 | 0.000578 |
| SCUBE3 | 222663 | chr6 | 35228800 | 35233999 | high | 13.00 | 0.006320 |
| SCYL2 | 55681 | chr12 | 100266800 | 100268399 | low | -6.95 | 0.000555 |
| SDAD1 | 55153 | chr4 | 75990400 | 75991999 | high | 4.00 | 0.000726 |
| SDC1 | 6382 | chr2 | 20214200 | 20219999 | high | 14.50 | 0.000316 |
| SDC2 | 6383 | chr8 | 96494400 | 96498599 | high | 10.50 | 0.000066 |
| SDC2 | 6383 | chr8 | 96607200 | 96613799 | high | 16.50 | 0.000066 |
| SDCBP | 6386 | chr8 | 58570000 | 58573599 | high | 9.00 | 0.000109 |
| SDCBP | 6386 | chr8 | 58564000 | 58567599 | high | 9.00 | 0.000109 |
| SDCBP2 | 27111 | chr20 | 1308800 | 1311599 | high | 7.00 | 0.002302 |
| SDCBP2 | 27111 | chr20 | 1321200 | 1328799 | high | 19.00 | 0.002280 |
| SDE2 | 163859 | chr1 | 225993600 | 225996799 | high | 8.00 | 0.000725 |
| SDF2 | 6388 | chr17 | 28640400 | 28648599 | high | 20.50 | 0.000223 |
| SDF4 | 51150 | chr1 | 1231200 | 1232599 | low | -6.08 | 0.004616 |
| SDHAF3 | 57001 | chr7 | 97117000 | 97119199 | low | -9.56 | 0.000587 |
| SDHB | 6390 | chr1 | 17050800 | 17053999 | high | 8.00 | 0.000375 |
| SDK1 | 221935 | chr7 | 3610600 | 3613799 | low | -13.91 | 0.006830 |
| SDK1 | 221935 | chr7 | 3452000 | 3454599 | low | -11.30 | 0.007144 |
| SDPR | 8436 | chr2 | 191837000 | 191840799 | low | -16.52 | 0.000044 |
| SEC11A | 23478 | chr15 | 84714800 | 84717199 | high | 6.00 | 0.000277 |
| SEC11C | 90701 | chr18 | 59150800 | 59154799 | high | 10.00 | 0.001533 |
| SEC14L1 | 6397 | chr17 | 77181800 | 77185399 | high | 9.00 | 0.000083 |
| SEC14L1 | 6397 | chr17 | 77125000 | 77130199 | high | 13.00 | 0.000083 |
| SEC14L1 | 6397 | chr17 | 77138000 | 77150199 | high | 30.50 | 0.000083 |
| SEC14L2 | 23541 | chr22 | 30412600 | 30417199 | high | 11.50 | 0.000774 |
| SEC14L2 | 23541 | chr22 | 30423400 | 30429399 | high | 15.00 | 0.000774 |
| SEC22A | 26984 | chr3 | 123214200 | 123217399 | low | -13.91 | 0.000219 |
| SEC22A | 26984 | chr3 | 123248800 | 123251399 | low | -11.30 | 0.000219 |
| SEC22B | 9554 | chr1 | 120175800 | 120176799 | low | -4.34 | 0.000080 |
| SEC22C | 9117 | chr3 | 42592800 | 42595199 | high | 6.00 | 0.000214 |
| SEC22C | 9117 | chr3 | 42573800 | 42578799 | high | 12.50 | 0.000214 |
| SEC23B | 10483 | chr20 | 18507000 | 18509799 | high | 7.00 | 0.000566 |
| SEC23IP | 11196 | chr10 | 119899200 | 119900999 | low | -7.82 | 0.000093 |
| SEC24A | 10802 | chr5 | 134648000 | 134650599 | low | -11.30 | 0.000080 |
| SEC24C | 9632 | chr10 | 73760000 | 73762399 | high | 6.00 | 0.000131 |
| SEC24C | 9632 | chr10 | 73769000 | 73773199 | high | 10.50 | 0.000131 |
| SEC24D | 9871 | chr4 | 118758200 | 118761999 | high | 9.50 | 0.000083 |
| SEC24D | 9871 | chr4 | 118746600 | 118750599 | high | 10.00 | 0.000083 |
| SEC61A1 | 29927 | chr3 | 128061600 | 128066199 | high | 11.50 | 0.000234 |
| SEC61A2 | 55176 | chr10 | 12129800 | 12132199 | low | -10.43 | 0.004549 |
| SEC61A2 | 55176 | chr10 | 12163600 | 12165799 | low | -9.56 | 0.004536 |
| SEC61B | 10952 | chr9 | 99219600 | 99222799 | high | 8.00 | 0.000110 |
| SECISBP2 | 79048 | chr9 | 89317200 | 89320999 | high | 9.50 | 0.000885 |
| SECTM1 | 6398 | chr17 | 82330600 | 82334999 | high | 11.00 | 0.000078 |
| SEH1L | 81929 | chr18 | 12947200 | 12950399 | high | 8.00 | 0.006328 |
| SELO | 83642 | chr22 | 50199200 | 50203799 | high | 11.50 | 0.001666 |

| | | | | | | | |
|---|---|---|---|---|---|---|---|
| SELPLG | 6404 | chr12 | 108621400 | 108625999 | high | 11.50 | 0.000059 |
| SELT | 51714 | chr3 | 150602200 | 150604999 | high | 7.00 | 0.000343 |
| SEMA3A | 10371 | chr7 | 84086800 | 84089999 | high | 8.00 | 0.000123 |
| SEMA3A | 10371 | chr7 | 84159800 | 84163799 | high | 10.00 | 0.000123 |
| SEMA3A | 10371 | chr7 | 84109400 | 84113799 | high | 11.00 | 0.000123 |
| SEMA3A | 10371 | chr7 | 84074200 | 84079199 | high | 12.50 | 0.000123 |
| SEMA3A | 10371 | chr7 | 84096200 | 84102599 | high | 16.00 | 0.000123 |
| SEMA3A | 10371 | chr7 | 84037400 | 84044599 | high | 18.00 | 0.000123 |
| SEMA3A | 10371 | chr7 | 84116800 | 84126199 | high | 23.50 | 0.000123 |
| SEMA3B | 7869 | chr3 | 50267200 | 50269999 | low | -12.17 | 0.000157 |
| SEMA3C | 10512 | chr7 | 80772600 | 80776599 | low | -17.30 | 0.000130 |
| SEMA3C | 10512 | chr7 | 80759000 | 80762399 | low | -14.78 | 0.000130 |
| SEMA3C | 10512 | chr7 | 80746200 | 80747399 | low | -5.21 | 0.000130 |
| SEMA3D | 223117 | chr7 | 85105200 | 85109199 | low | -17.80 | 0.002622 |
| SEMA3D | 223117 | chr7 | 85094000 | 85096999 | low | -13.04 | 0.002622 |
| SEMA3D | 223117 | chr7 | 84995400 | 84998199 | low | -12.17 | 0.002625 |
| SEMA3E | 9723 | chr7 | 83402000 | 83405399 | low | -14.78 | 0.000117 |
| SEMA3F | 6405 | chr3 | 50159400 | 50162799 | high | 8.50 | 0.000128 |
| SEMA3F | 6405 | chr3 | 50181600 | 50186199 | high | 11.50 | 0.000128 |
| SEMA3F | 6405 | chr3 | 50163600 | 50168999 | high | 13.50 | 0.000128 |
| SEMA4A | 64218 | chr1 | 156148000 | 156153599 | high | 14.00 | 0.000411 |
| SEMA4B | 10509 | chr15 | 90199200 | 90202199 | high | 7.50 | 0.000117 |
| SEMA4C | 54910 | chr2 | 96868200 | 96870399 | high | 5.50 | 0.000567 |
| SEMA4G | 57715 | chr10 | 100985600 | 100989799 | low | -18.25 | 0.000572 |
| SEMA5A | 9037 | chr5 | 9199800 | 9201399 | high | 4.00 | 0.000982 |
| SEMA5A | 9037 | chr5 | 9505600 | 9507599 | high | 5.00 | 0.000951 |
| SEMA5A | 9037 | chr5 | 9278600 | 9281199 | high | 6.50 | 0.000974 |
| SEMA5A | 9037 | chr5 | 9052000 | 9055799 | high | 9.50 | 0.000998 |
| SEMA5A | 9037 | chr5 | 9427200 | 9431399 | high | 10.50 | 0.000959 |
| SEMA5A | 9037 | chr5 | 9186600 | 9190999 | high | 11.00 | 0.000984 |
| SEMA5A | 9037 | chr5 | 9409600 | 9414199 | high | 11.50 | 0.000960 |
| SEMA5A | 9037 | chr5 | 9284200 | 9288799 | high | 11.50 | 0.000973 |
| SEMA5A | 9037 | chr5 | 9341200 | 9345999 | high | 12.00 | 0.000967 |
| SEMA5A | 9037 | chr5 | 9179200 | 9184199 | high | 12.50 | 0.000985 |
| SEMA5A | 9037 | chr5 | 9064800 | 9069799 | high | 12.50 | 0.000997 |
| SEMA5A | 9037 | chr5 | 9499600 | 9504799 | high | 13.00 | 0.000951 |
| SEMA5A | 9037 | chr5 | 9395800 | 9400999 | high | 13.00 | 0.000962 |
| SEMA5A | 9037 | chr5 | 9332800 | 9337999 | high | 13.00 | 0.000968 |
| SEMA5A | 9037 | chr5 | 9449200 | 9454599 | high | 13.50 | 0.000956 |
| SEMA5A | 9037 | chr5 | 9249000 | 9254399 | high | 13.50 | 0.000977 |
| SEMA5A | 9037 | chr5 | 9303400 | 9309599 | high | 15.50 | 0.000971 |
| SEMA5A | 9037 | chr5 | 9241800 | 9247999 | high | 15.50 | 0.000978 |
| SEMA5A | 9037 | chr5 | 9360400 | 9367999 | high | 19.00 | 0.000965 |
| SEMA5A | 9037 | chr5 | 9164400 | 9173599 | high | 23.00 | 0.000986 |
| SEMA5B | 54437 | chr3 | 122932000 | 122936599 | high | 11.50 | 0.000443 |
| SEMA6C | 10500 | chr1 | 151144200 | 151146999 | high | 7.00 | 0.000069 |
| SEMA6D | 80031 | chr15 | 47739000 | 47742599 | high | 9.00 | 0.001676 |
| SEMA6D | 80031 | chr15 | 47556600 | 47560999 | high | 11.00 | 0.001683 |
| SEMA6D | 80031 | chr15 | 47713600 | 47718599 | high | 12.50 | 0.001677 |
| SEMA6D | 80031 | chr15 | 47616600 | 47624999 | high | 21.00 | 0.001681 |
| SENP1 | 29843 | chr12 | 48105000 | 48107799 | high | 7.00 | 0.000620 |
| SENP3 | 26168 | chr17 | 7565600 | 7569799 | high | 10.50 | 0.003459 |
| SENP3 | 26168 | chr17 | 7560000 | 7564199 | high | 10.50 | 0.003461 |
| SENP7 | 57337 | chr3 | 101472800 | 101474799 | high | 5.00 | 0.000565 |
| SENP8 | 123228 | chr15 | 72126600 | 72128199 | high | 4.00 | 0.001708 |
| SEPHS1 | 22929 | chr10 | 13346400 | 13349999 | low | -15.65 | 0.001718 |
| SEPHS2 | 22928 | chr16 | 30443600 | 30446999 | low | -14.78 | 0.000753 |
| SERAC1 | 84947 | chr6 | 158117000 | 158120599 | high | 9.00 | 0.000537 |
| SERBP1 | 26135 | chr1 | 67426400 | 67428399 | low | -8.69 | 0.000388 |
| SERINC1 | 57515 | chr6 | 122459400 | 122462999 | low | -15.65 | 0.000470 |
| SERINC1 | 57515 | chr6 | 122470800 | 122473999 | low | -13.91 | 0.000470 |
| SERINC1 | 57515 | chr6 | 122452800 | 122455199 | low | -10.43 | 0.000470 |
| SERINC4 | 619189 | chr15 | 43799000 | 43802199 | high | 8.00 | 0.014137 |
| SERINC5 | 256987 | chr5 | 80246000 | 80248799 | low | -12.17 | 0.003202 |
| SERPINB1 | 1992 | chr6 | 2839000 | 2844599 | high | 14.00 | 0.000702 |
| SERPINB7 | 8710 | chr18 | 63773600 | 63779399 | high | 14.50 | 0.000137 |
| SERPINB8 | 5271 | chr18 | 63969000 | 63971999 | high | 7.50 | 0.000082 |
| SERPINE1 | 5054 | chr7 | 101136000 | 101140199 | high | 10.50 | 0.000050 |
| SERPINE3 | 647174 | chr13 | 51352000 | 51355399 | high | 8.50 | 0.012603 |
| SERTAD2 | 9792 | chr2 | 64639000 | 64640599 | high | 4.00 | 0.000151 |
| SESN1 | 27244 | chr6 | 109008000 | 109011399 | high | 8.50 | 0.000250 |
| SESN1 | 27244 | chr6 | 109094200 | 109098799 | high | 11.50 | 0.000250 |
| SESN1 | 27244 | chr6 | 109032400 | 109038399 | high | 15.00 | 0.000250 |
| SESN2 | 83667 | chr1 | 28259000 | 28260399 | low | -6.08 | 0.002961 |
| SETD1B | 23067 | chr12 | 121808600 | 121813199 | high | 11.50 | 0.000189 |
| SETD2 | 29072 | chr3 | 47124400 | 47127599 | high | 8.00 | 0.000617 |
| SETD2 | 29072 | chr3 | 47118800 | 47122399 | high | 9.00 | 0.000617 |
| SETD2 | 29072 | chr3 | 47075600 | 47081999 | high | 16.00 | 0.000618 |

| | | | | | | | |
|---|---|---|---|---|---|---|---|
| SETD2 | 29072 | chr3 | 47050200 | 47058799 | high | 21.50 | 0.000618 |
| SETD3 | 84193 | chr14 | 99480200 | 99484399 | high | 10.50 | 0.000846 |
| SETD4 | 54093 | chr21 | 36058400 | 36063199 | high | 12.00 | 0.001500 |
| SETD6 | 79918 | chr16 | 58520400 | 58524399 | high | 10.00 | 0.001366 |
| SETD7 | 80854 | chr4 | 139555400 | 139557199 | high | 4.50 | 0.000579 |
| SETD7 | 80854 | chr4 | 139528600 | 139531799 | high | 8.00 | 0.000579 |
| SETDB2 | 83852 | chr13 | 49488200 | 49493199 | high | 12.50 | 0.001694 |
| SETDB2 | 83852 | chr13 | 49470200 | 49475199 | high | 12.50 | 0.001695 |
| SF3A1 | 10291 | chr22 | 30348800 | 30351599 | high | 7.00 | 0.000339 |
| SF3B1 | 23451 | chr2 | 197432200 | 197437599 | low | -23.43 | 0.000119 |
| SF3B3 | 23450 | chr16 | 70522200 | 70524599 | high | 6.00 | 0.000333 |
| SF3B3 | 23450 | chr16 | 70546200 | 70549799 | high | 9.00 | 0.000332 |
| SF3B3 | 23450 | chr16 | 70530800 | 70534599 | high | 9.50 | 0.000332 |
| SF3B5 | 83443 | chr6 | 144094600 | 144095999 | low | -6.08 | 0.000579 |
| SFMBT1 | 51460 | chr3 | 53044800 | 53046599 | high | 4.50 | 0.000970 |
| SFR1 | 119392 | chr10 | 104123400 | 104126799 | high | 8.50 | 0.001147 |
| SFRP1 | 6422 | chr8 | 41305600 | 41311399 | high | 14.50 | 0.000155 |
| SFT2D1 | 113402 | chr6 | 166340800 | 166343399 | low | -11.30 | 0.000682 |
| SFTPB | 6439 | chr2 | 85664200 | 85666199 | low | -8.69 | 0.000075 |
| SFTPC | 6440 | chr8 | 22162600 | 22165599 | high | 7.50 | 0.000291 |
| SFXN2 | 118980 | chr10 | 102725400 | 102728599 | high | 8.00 | 0.001158 |
| SFXN3 | 81855 | chr10 | 101031200 | 101033799 | high | 6.50 | 0.000810 |
| SFXN3 | 81855 | chr10 | 101037000 | 101040199 | high | 8.00 | 0.000810 |
| SFXN4 | 119559 | chr10 | 119165200 | 119170599 | high | 13.50 | 0.001003 |
| SFXN5 | 94097 | chr2 | 73070600 | 73072599 | high | 5.00 | 0.001288 |
| SFXN5 | 94097 | chr2 | 73015600 | 73021199 | high | 14.00 | 0.001289 |
| SGCE | 8910 | chr7 | 94636000 | 94640399 | high | 11.00 | 0.000094 |
| SGCE | 8910 | chr7 | 94650600 | 94658799 | high | 20.50 | 0.000094 |
| SGIP1 | 84251 | chr1 | 66732200 | 66735199 | high | 7.50 | 0.001263 |
| SGIP1 | 84251 | chr1 | 66555600 | 66560599 | high | 12.50 | 0.001266 |
| SGIP1 | 84251 | chr1 | 66704200 | 66710399 | high | 15.50 | 0.001263 |
| SGIP1 | 84251 | chr1 | 66615000 | 66621399 | high | 16.00 | 0.001265 |
| SGIP1 | 84251 | chr1 | 66568200 | 66574999 | high | 17.00 | 0.001266 |
| SGIP1 | 84251 | chr1 | 66544800 | 66552799 | high | 20.00 | 0.001266 |
| SGK1 | 6446 | chr6 | 134236200 | 134240999 | low | -20.39 | 0.000048 |
| SGK1 | 6446 | chr6 | 134173000 | 134176599 | low | -15.65 | 0.000048 |
| SGK223 | 157285 | chr8 | 8350600 | 8352199 | low | -6.95 | 0.018835 |
| SGK3 | 23678 | chr8 | 66773200 | 66775799 | high | 6.50 | 0.000355 |
| SGK3 | 23678 | chr8 | 66783200 | 66786399 | high | 8.00 | 0.000355 |
| SGK494 | 124923 | chr17 | 28610200 | 28613799 | high | 9.00 | 0.004366 |
| SGO1 | 151648 | chr3 | 20184800 | 20186999 | high | 5.50 | 0.007513 |
| SGSM3 | 27352 | chr22 | 40369200 | 40372599 | high | 8.50 | 0.000678 |
| SGSM3 | 27352 | chr22 | 40375800 | 40379599 | high | 9.50 | 0.000677 |
| SGTB | 54557 | chr5 | 65712800 | 65715399 | high | 6.50 | 0.000830 |
| SH2B1 | 25970 | chr16 | 28862000 | 28865599 | high | 9.00 | 0.000900 |
| SH2B3 | 10019 | chr12 | 111443800 | 111449399 | high | 14.00 | 0.000090 |
| SH2B3 | 10019 | chr12 | 111408200 | 111415199 | high | 17.50 | 0.000090 |
| SH2D3C | 10044 | chr9 | 127738400 | 127740999 | high | 6.50 | 0.000079 |
| SH2D3C | 10044 | chr9 | 127761200 | 127765599 | high | 11.00 | 0.000079 |
| SH2D4A | 63898 | chr8 | 19375000 | 19379799 | high | 12.00 | 0.003298 |
| SH2D4A | 63898 | chr8 | 19353200 | 19364199 | high | 27.50 | 0.003302 |
| SH3BP1 | 23616 | chr22 | 37653000 | 37656799 | low | -16.52 | 0.000627 |
| SH3BP2 | 6452 | chr4 | 2812200 | 2815199 | high | 7.50 | 0.002294 |
| SH3BP4 | 23677 | chr2 | 235004400 | 235007999 | high | 9.00 | 0.000101 |
| SH3BP4 | 23677 | chr2 | 235047400 | 235052199 | high | 12.00 | 0.000101 |
| SH3BP4 | 23677 | chr2 | 234990200 | 234995799 | high | 14.00 | 0.000101 |
| SH3BP5 | 9467 | chr3 | 15271400 | 15274599 | high | 8.00 | 0.000620 |
| SH3BP5 | 9467 | chr3 | 15253200 | 15258999 | high | 14.50 | 0.000621 |
| SH3BP5 | 9467 | chr3 | 15327400 | 15336799 | high | 23.50 | 0.000618 |
| SH3BP5L | 80851 | chr1 | 248817400 | 248822399 | high | 12.50 | 0.000325 |
| SH3GL1 | 6455 | chr19 | 4383800 | 4386199 | high | 6.00 | 0.001472 |
| SH3GL1 | 6455 | chr19 | 4397600 | 4400599 | high | 7.50 | 0.001468 |
| SH3GL1 | 6455 | chr19 | 4375600 | 4381199 | high | 14.00 | 0.001475 |
| SH3GLB2 | 56904 | chr9 | 129026400 | 129028999 | high | 6.50 | 0.000441 |
| SH3RF1 | 57630 | chr4 | 169164600 | 169165799 | high | 3.00 | 0.000341 |
| SH3RF1 | 57630 | chr4 | 169218600 | 169220799 | high | 5.50 | 0.000341 |
| SH3RF1 | 57630 | chr4 | 169258200 | 169261399 | high | 8.00 | 0.000340 |
| SH3RF1 | 57630 | chr4 | 169203400 | 169207599 | high | 10.50 | 0.000341 |
| SH3RF1 | 57630 | chr4 | 169222000 | 169228999 | high | 17.50 | 0.000341 |
| SHANK3 | 85358 | chr22 | 50695200 | 50699999 | high | 12.00 | 0.001684 |
| SHB | 6461 | chr9 | 38025800 | 38028399 | high | 6.50 | 0.000170 |
| SHB | 6461 | chr9 | 38001000 | 38003799 | high | 7.00 | 0.000170 |
| SHB | 6461 | chr9 | 38059200 | 38063999 | high | 12.00 | 0.000170 |
| SHB | 6461 | chr9 | 37983400 | 37988199 | high | 12.00 | 0.000170 |
| SHB | 6461 | chr9 | 38008000 | 38013199 | high | 13.00 | 0.000170 |
| SHB | 6461 | chr9 | 37929800 | 37935799 | high | 15.00 | 0.000170 |
| SHB | 6461 | chr9 | 37966400 | 37972999 | high | 16.50 | 0.000170 |
| SHB | 6461 | chr9 | 38046400 | 38053399 | high | 17.50 | 0.000170 |

| | | | | | | | |
|---|---|---|---|---|---|---|---|
| SHC1 | 6464 | chr1 | 154966800 | 154978599 | high | 29.50 | 0.000042 |
| SHC3 | 53358 | chr9 | 89092600 | 89098399 | high | 14.50 | 0.000599 |
| SHC3 | 53358 | chr9 | 89150000 | 89155999 | high | 15.00 | 0.000599 |
| SHC4 | 399694 | chr15 | 48876800 | 48879199 | high | 6.00 | 0.008178 |
| SHC4 | 399694 | chr15 | 48859200 | 48863599 | high | 11.00 | 0.008181 |
| SHC4 | 399694 | chr15 | 48830800 | 48841599 | high | 27.00 | 0.008185 |
| SHF | 90525 | chr15 | 45198800 | 45201799 | high | 7.50 | 0.002003 |
| SHISA4 | 149345 | chr1 | 201888400 | 201894399 | high | 15.00 | 0.000740 |
| SHISA5 | 51246 | chr3 | 48472600 | 48478199 | high | 14.00 | 0.001057 |
| SHKBP1 | 92799 | chr19 | 40584400 | 40590999 | high | 16.50 | 0.002287 |
| SHOC2 | 8036 | chr10 | 110962600 | 110964799 | high | 5.50 | 0.000072 |
| SHOC2 | 8036 | chr10 | 110992600 | 110995799 | high | 8.00 | 0.000072 |
| SHPK | 23729 | chr17 | 3635600 | 3636799 | high | 3.00 | 0.006527 |
| SHQ1 | 55164 | chr3 | 72806000 | 72811399 | high | 13.50 | 0.000758 |
| SIAH1 | 6477 | chr16 | 48382800 | 48385999 | high | 8.00 | 0.000134 |
| SIAH1 | 6477 | chr16 | 48367800 | 48371999 | high | 10.50 | 0.000134 |
| SIAH2 | 6478 | chr3 | 150759600 | 150763799 | high | 10.50 | 0.000043 |
| SIK2 | 23235 | chr11 | 111605000 | 111609799 | high | 12.00 | 0.000208 |
| SIK3 | 23387 | chr11 | 116855600 | 116858999 | high | 8.50 | 0.000200 |
| SIK3 | 23387 | chr11 | 116845800 | 116849199 | high | 8.50 | 0.000200 |
| SIK3 | 23387 | chr11 | 116877400 | 116881399 | high | 10.00 | 0.000200 |
| SIK3 | 23387 | chr11 | 116866200 | 116874599 | high | 21.00 | 0.000200 |
| SIK3 | 23387 | chr11 | 117024400 | 117036999 | high | 31.50 | 0.000200 |
| SIN3A | 25942 | chr15 | 75416000 | 75418799 | high | 7.00 | 0.000344 |
| SIN3A | 25942 | chr15 | 75440000 | 75443999 | high | 10.00 | 0.000344 |
| SIN3A | 25942 | chr15 | 75428400 | 75436199 | high | 19.50 | 0.000344 |
| SIPA1L1 | 26037 | chr14 | 71691600 | 71693999 | high | 6.00 | 0.000363 |
| SIPA1L1 | 26037 | chr14 | 71593800 | 71596799 | high | 7.50 | 0.000364 |
| SIPA1L1 | 26037 | chr14 | 71608800 | 71612599 | high | 9.50 | 0.000364 |
| SIPA1L1 | 26037 | chr14 | 71682600 | 71687199 | high | 11.50 | 0.000363 |
| SIPA1L1 | 26037 | chr14 | 71621200 | 71625999 | high | 12.00 | 0.000364 |
| SIPA1L1 | 26037 | chr14 | 71718000 | 71723999 | high | 15.00 | 0.000363 |
| SIPA1L3 | 23094 | chr19 | 37979600 | 37982399 | high | 7.00 | 0.000608 |
| SIPA1L3 | 23094 | chr19 | 37904800 | 37908599 | high | 9.50 | 0.000609 |
| SIPA1L3 | 23094 | chr19 | 38062400 | 38066399 | high | 10.00 | 0.000607 |
| SIPA1L3 | 23094 | chr19 | 37973600 | 37978799 | high | 13.00 | 0.000608 |
| SIPA1L3 | 23094 | chr19 | 38195400 | 38200799 | high | 13.50 | 0.000605 |
| SIPA1L3 | 23094 | chr19 | 37989400 | 37996199 | high | 17.00 | 0.000608 |
| SIPA1L3 | 23094 | chr19 | 38001400 | 38008599 | high | 18.00 | 0.000608 |
| SIRT1 | 23411 | chr10 | 67891600 | 67896399 | high | 12.00 | 0.000345 |
| SIRT5 | 23408 | chr6 | 13612200 | 13617999 | high | 14.50 | 0.001720 |
| SIVA1 | 10572 | chr14 | 104752800 | 104755399 | high | 6.50 | 0.000101 |
| SKA2 | 348235 | chr17 | 59149400 | 59151599 | high | 5.50 | 0.005887 |
| SKI | 6497 | chr1 | 2290400 | 2294199 | high | 9.50 | 0.002837 |
| SKI | 6497 | chr1 | 2299200 | 2303399 | high | 10.50 | 0.002826 |
| SKIDA1 | 387640 | chr10 | 21521400 | 21524599 | high | 8.00 | 0.018012 |
| SKIDA1 | 387640 | chr10 | 21514400 | 21520599 | high | 15.50 | 0.018018 |
| SKIV2L2 | 23517 | chr5 | 55406400 | 55411199 | high | 12.00 | 0.000424 |
| SKIV2L2 | 23517 | chr5 | 55400000 | 55405399 | high | 13.50 | 0.000424 |
| SKIV2L2 | 23517 | chr5 | 55388600 | 55394599 | high | 15.00 | 0.000425 |
| SKP2 | 6502 | chr5 | 36178200 | 36180999 | high | 7.00 | 0.000180 |
| SLA | 6503 | chr8 | 133057200 | 133060399 | low | -13.91 | 0.000049 |
| SLA | 6503 | chr8 | 133052400 | 133054999 | low | -11.30 | 0.000049 |
| SLAIN2 | 57606 | chr4 | 48345800 | 48348599 | high | 7.00 | 0.001192 |
| SLAIN2 | 57606 | chr4 | 48393400 | 48396799 | high | 8.50 | 0.001190 |
| SLAIN2 | 57606 | chr4 | 48340200 | 48344599 | high | 11.00 | 0.001192 |
| SLAIN2 | 57606 | chr4 | 48350200 | 48355199 | high | 12.50 | 0.001191 |
| SLAIN2 | 57606 | chr4 | 48409200 | 48414399 | high | 13.00 | 0.001190 |
| SLBP | 7884 | chr4 | 1697800 | 1704399 | high | 16.50 | 0.004644 |
| SLC10A7 | 84068 | chr4 | 146520800 | 146522999 | low | -9.56 | 0.000574 |
| SLC11A2 | 4891 | chr12 | 51004600 | 51006999 | high | 6.00 | 0.000096 |
| SLC12A2 | 6558 | chr5 | 128102800 | 128107799 | high | 12.50 | 0.000051 |
| SLC12A3 | 6559 | chr16 | 56902000 | 56906799 | high | 12.00 | 0.000115 |
| SLC12A4 | 6560 | chr16 | 67967800 | 67968999 | high | 3.00 | 0.000097 |
| SLC12A6 | 9990 | chr15 | 34274200 | 34277399 | high | 8.00 | 0.000291 |
| SLC12A8 | 84561 | chr3 | 125120000 | 125121199 | high | 3.00 | 0.000676 |
| SLC12A8 | 84561 | chr3 | 125108000 | 125111799 | high | 9.50 | 0.000676 |
| SLC12A8 | 84561 | chr3 | 125092000 | 125095799 | high | 9.50 | 0.000676 |
| SLC12A8 | 84561 | chr3 | 125201400 | 125205399 | high | 10.00 | 0.000675 |
| SLC12A9 | 56996 | chr7 | 100855400 | 100858599 | high | 8.00 | 0.000565 |
| SLC14A1 | 6563 | chr18 | 45736000 | 45738999 | high | 7.50 | 0.000143 |
| SLC16A12 | 387700 | chr10 | 89464000 | 89465799 | low | -7.82 | 0.004334 |
| SLC16A12 | 387700 | chr10 | 89461400 | 89462999 | low | -6.95 | 0.004334 |
| SLC16A12 | 387700 | chr10 | 89437200 | 89438399 | low | -5.21 | 0.004335 |
| SLC16A7 | 9194 | chr12 | 59654600 | 59656399 | low | -7.82 | 0.000154 |
| SLC17A5 | 26503 | chr6 | 73617800 | 73622399 | high | 11.50 | 0.000360 |
| SLC17A5 | 26503 | chr6 | 73593200 | 73598199 | high | 12.50 | 0.000360 |
| SLC17A7 | 57030 | chr19 | 49439200 | 49443799 | high | 11.50 | 0.001154 |

| | | | | | | | |
|---|---|---|---|---|---|---|---|
| SLC19A1 | 6573 | chr21 | 45540800 | 45543399 | low | -11.30 | 0.000144 |
| SLC19A2 | 10560 | chr1 | 169485000 | 169486799 | high | 4.50 | 0.000062 |
| SLC1A1 | 6505 | chr9 | 4558000 | 4561599 | high | 9.00 | 0.001427 |
| SLC1A1 | 6505 | chr9 | 4543200 | 4551399 | high | 20.50 | 0.001432 |
| SLC1A2 | 6506 | chr11 | 35386000 | 35388599 | high | 6.50 | 0.000184 |
| SLC1A2 | 6506 | chr11 | 35416800 | 35419999 | high | 8.00 | 0.000184 |
| SLC1A2 | 6506 | chr11 | 35337600 | 35340799 | high | 8.00 | 0.000184 |
| SLC1A2 | 6506 | chr11 | 35303000 | 35306399 | high | 8.50 | 0.000184 |
| SLC1A2 | 6506 | chr11 | 35330800 | 35334399 | high | 9.00 | 0.000184 |
| SLC1A2 | 6506 | chr11 | 35358200 | 35362199 | high | 10.00 | 0.000184 |
| SLC1A2 | 6506 | chr11 | 35293000 | 35297599 | high | 11.50 | 0.000184 |
| SLC1A2 | 6506 | chr11 | 35272600 | 35277399 | high | 12.00 | 0.000184 |
| SLC1A2 | 6506 | chr11 | 35410800 | 35415799 | high | 12.50 | 0.000184 |
| SLC1A2 | 6506 | chr11 | 35402400 | 35407599 | high | 13.00 | 0.000184 |
| SLC1A2 | 6506 | chr11 | 35319400 | 35324999 | high | 14.00 | 0.000184 |
| SLC1A2 | 6506 | chr11 | 35376000 | 35382399 | high | 16.00 | 0.000184 |
| SLC1A5 | 6510 | chr19 | 46783800 | 46789399 | high | 14.00 | 0.000139 |
| SLC20A1 | 6574 | chr2 | 112656400 | 112661199 | high | 12.00 | 0.000058 |
| SLC20A1 | 6574 | chr2 | 112643600 | 112649799 | high | 15.50 | 0.000058 |
| SLC20A2 | 6575 | chr8 | 42540400 | 42542399 | high | 5.00 | 0.000155 |
| SLC20A2 | 6575 | chr8 | 42499000 | 42503399 | high | 11.00 | 0.000155 |
| SLC22A5 | 6584 | chr5 | 132387600 | 132393599 | high | 15.00 | 0.000050 |
| SLC22A5 | 6584 | chr5 | 132366600 | 132373799 | high | 18.00 | 0.000050 |
| SMG6 | 23293 | chr17 | 2063200 | 2072999 | high | 24.50 | 0.011290 |
| SMG7 | 9887 | chr1 | 183490800 | 183494199 | high | 8.50 | 0.000054 |
| SMG7 | 9887 | chr1 | 183528800 | 183535799 | high | 17.50 | 0.000054 |
| SMG8 | 55181 | chr17 | 59210200 | 59213399 | low | -13.91 | 0.000932 |
| SMIM13 | 221710 | chr6 | 11093400 | 11095599 | low | -9.56 | 0.019986 |
| SMIM14 | 201895 | chr4 | 39553800 | 39557999 | high | 10.50 | 0.005104 |
| SMIM14 | 201895 | chr4 | 39539200 | 39547399 | high | 20.50 | 0.005106 |
| SMIM15 | 643155 | chr5 | 61159600 | 61163599 | high | 10.00 | 0.010516 |
| SMIM19 | 114926 | chr8 | 42540400 | 42542399 | high | 5.00 | 0.002702 |
| SMIM2 | 79024 | chr13 | 44141600 | 44144199 | low | -11.30 | 0.001790 |
| SMIM3 | 85027 | chr5 | 150775800 | 150780799 | high | 12.50 | 0.000564 |
| SMIM3 | 85027 | chr5 | 150787000 | 150793799 | high | 17.00 | 0.000564 |
| SMIM4 | 440957 | chr3 | 52536400 | 52537399 | high | 2.50 | 0.008393 |
| SMOX | 54498 | chr20 | 4148200 | 4150199 | high | 5.00 | 0.013138 |
| SMOX | 54498 | chr20 | 4169000 | 4173199 | high | 10.50 | 0.013072 |
| SMOX | 54498 | chr20 | 4163800 | 4168199 | high | 11.00 | 0.013089 |
| SMTN | 6525 | chr22 | 31097400 | 31100399 | low | -13.04 | 0.000210 |
| SMTN | 6525 | chr22 | 31081200 | 31082599 | low | -6.08 | 0.000210 |
| SMU1 | 55234 | chr9 | 33040800 | 33046799 | high | 15.00 | 0.001672 |
| SMURF2 | 64750 | chr17 | 64591600 | 64594199 | high | 6.50 | 0.001002 |
| SMURF2 | 64750 | chr17 | 64600600 | 64605399 | high | 12.00 | 0.001002 |
| SMURF2 | 64750 | chr17 | 64595000 | 64599799 | high | 12.00 | 0.001002 |
| SMURF2 | 64750 | chr17 | 64627800 | 64634199 | high | 16.00 | 0.001002 |
| SMURF2 | 64750 | chr17 | 64562800 | 64571199 | high | 21.00 | 0.001003 |
| SMURF2 | 64750 | chr17 | 64638600 | 64647999 | high | 23.50 | 0.001002 |
| SMYD3 | 64754 | chr1 | 246506000 | 246507799 | low | -7.82 | 0.000263 |
| SMYD3 | 64754 | chr1 | 246432400 | 246434199 | low | -7.82 | 0.000263 |
| SMYD4 | 114826 | chr17 | 1810800 | 1814799 | high | 10.00 | 0.005050 |
| SMYD5 | 10322 | chr2 | 73212600 | 73216799 | high | 10.50 | 0.000141 |
| SNAI1 | 6615 | chr20 | 49985400 | 49988799 | high | 8.50 | 0.000132 |
| SNAI2 | 6591 | chr8 | 48918600 | 48922399 | low | -16.52 | 0.000135 |
| SNAI3 | 333929 | chr16 | 88683400 | 88689199 | high | 14.50 | 0.003765 |
| SNAP23 | 8773 | chr15 | 42527200 | 42532399 | high | 13.00 | 0.000206 |
| SNAP23 | 8773 | chr15 | 42503800 | 42510199 | high | 16.00 | 0.000206 |
| SNAP25 | 6616 | chr20 | 10263600 | 10267799 | high | 10.50 | 0.000645 |
| SNAP29 | 9342 | chr22 | 20858000 | 20859599 | high | 4.00 | 0.000448 |
| SNAPC3 | 6619 | chr9 | 15431800 | 15434599 | high | 7.00 | 0.000429 |
| SNAPC4 | 6621 | chr9 | 136370400 | 136375999 | high | 14.00 | 0.000049 |
| SNF8 | 11267 | chr17 | 48942000 | 48945199 | high | 8.00 | 0.000230 |
| SNHG12 | 85028 | chr1 | 28580400 | 28582799 | low | -10.43 | 0.002975 |
| SNHG15 | 285958 | chr7 | 44985200 | 44986999 | low | -7.82 | 0.006357 |
| SNHG16 | 100507246 | chr17 | 76556600 | 76561999 | high | 13.50 | 0.005988 |
| SNHG17 | 388796 | chr20 | 38431000 | 38437599 | high | 16.50 | 0.010117 |
| SNHG19 | 100507303 | chr16 | 2152800 | 2161599 | high | 22.00 | 0.001401 |
| SNHG6 | 641638 | chr8 | 66924200 | 66925799 | low | -6.95 | 0.009588 |
| SNHG7 | 84973 | chr9 | 136724200 | 136728799 | high | 11.50 | 0.000621 |
| SNHG9 | 735301 | chr16 | 1963000 | 1965799 | low | -12.17 | 0.000038 |
| SNN | 8303 | chr16 | 11668000 | 11671399 | high | 8.50 | 0.000712 |
| SNPH | 9751 | chr20 | 1308800 | 1311599 | high | 7.00 | 0.007450 |
| SNPH | 9751 | chr20 | 1264600 | 1269399 | high | 12.00 | 0.007711 |
| SNRK | 54861 | chr3 | 43286600 | 43290199 | low | -15.65 | 0.001267 |
| SNRK | 54861 | chr3 | 43329600 | 43331399 | low | -7.82 | 0.001266 |
| SNRK | 54861 | chr3 | 43337800 | 43339199 | low | -6.08 | 0.001266 |
| SNRPC | 6631 | chr6 | 34756800 | 34759999 | high | 8.00 | 0.000191 |
| SNRPC | 6631 | chr6 | 34770800 | 34775599 | high | 12.00 | 0.000191 |

| | | | | | | | |
|---|---|---|---|---|---|---|---|
| SNRPD3 | 6634 | chr22 | 24563000 | 24566399 | high | 8.50 | 0.000270 |
| SNRPF | 6636 | chr12 | 95858000 | 95863799 | high | 14.50 | 0.000069 |
| SNTB1 | 6641 | chr8 | 120582800 | 120586199 | high | 8.50 | 0.000055 |
| SNTB1 | 6641 | chr8 | 120675400 | 120679199 | high | 9.50 | 0.000055 |
| SNTB1 | 6641 | chr8 | 120656400 | 120660799 | high | 11.00 | 0.000055 |
| SNTB1 | 6641 | chr8 | 120595200 | 120600399 | high | 13.00 | 0.000055 |
| SNTB2 | 6645 | chr16 | 69306200 | 69309199 | low | -13.04 | 0.000096 |
| SNTB2 | 6645 | chr16 | 69299600 | 69302199 | low | -11.30 | 0.000096 |
| SNTB2 | 6645 | chr16 | 69303200 | 69304599 | low | -6.08 | 0.000096 |
| SNU13 | 4809 | chr22 | 41687600 | 41689399 | low | -7.82 | 0.000115 |
| SNX15 | 29907 | chr11 | 65039800 | 65043199 | high | 8.50 | 0.000460 |
| SNX15 | 29907 | chr11 | 65027000 | 65031399 | high | 11.00 | 0.000460 |
| SNX2 | 6643 | chr5 | 122774400 | 122777799 | high | 8.50 | 0.000054 |
| SNX21 | 90203 | chr20 | 45832400 | 45835799 | high | 8.50 | 0.001968 |
| SNX22 | 79856 | chr15 | 64151000 | 64155799 | high | 12.00 | 0.001245 |
| SNX24 | 28966 | chr5 | 122987800 | 122990999 | high | 8.00 | 0.000236 |
| SNX24 | 28966 | chr5 | 122894800 | 122899799 | high | 12.50 | 0.000236 |
| SNX25 | 83891 | chr4 | 185304600 | 185308599 | high | 10.00 | 0.000453 |
| SNX27 | 81609 | chr1 | 151611200 | 151613999 | high | 7.00 | 0.000538 |
| SNX27 | 81609 | chr1 | 151624800 | 151628799 | high | 10.00 | 0.000538 |
| SNX29 | 92017 | chr16 | 11978400 | 11983799 | high | 13.50 | 0.007682 |
| SNX3 | 8724 | chr6 | 108260000 | 108261799 | high | 4.50 | 0.000081 |
| SNX31 | 169166 | chr8 | 100611000 | 100614799 | high | 9.50 | 0.001681 |
| SNX33 | 257364 | chr15 | 75655200 | 75659599 | low | -19.35 | 0.003402 |
| SNX4 | 8723 | chr3 | 125517800 | 125522199 | high | 11.00 | 0.000069 |
| SNX5 | 27131 | chr20 | 17959200 | 17965199 | high | 15.00 | 0.001511 |
| SNX7 | 51375 | chr1 | 98661400 | 98663799 | low | -10.43 | 0.000521 |
| SNX8 | 29886 | chr7 | 2251600 | 2255399 | high | 9.50 | 0.013273 |
| SNX9 | 51429 | chr6 | 157836600 | 157842999 | high | 16.00 | 0.000326 |
| SON | 6651 | chr21 | 33557600 | 33565199 | high | 19.00 | 0.000198 |
| SON | 6651 | chr21 | 33567200 | 33580199 | high | 32.50 | 0.000198 |
| SOS1 | 6654 | chr2 | 38987200 | 38991799 | low | -19.44 | 0.000171 |
| SOS1 | 6654 | chr2 | 39118800 | 39121999 | low | -13.91 | 0.000170 |
| SOS1 | 6654 | chr2 | 38994000 | 38996399 | low | -10.43 | 0.000171 |
| SOS2 | 6655 | chr14 | 50115400 | 50119199 | high | 9.50 | 0.000133 |
| SOS2 | 6655 | chr14 | 50164200 | 50168799 | high | 11.50 | 0.000133 |
| SOX5 | 6660 | chr12 | 24033800 | 24036799 | low | -13.04 | 0.000277 |
| SOX9 | 6662 | chr17 | 72119000 | 72122399 | high | 8.50 | 0.000092 |
| SP1 | 6667 | chr12 | 53402800 | 53407199 | high | 11.00 | 0.000125 |
| SP1 | 6667 | chr12 | 53409600 | 53414199 | high | 11.50 | 0.000125 |
| SP100 | 6672 | chr2 | 230419400 | 230425199 | high | 14.50 | 0.000029 |
| SP110 | 3431 | chr2 | 230204000 | 230207399 | high | 8.50 | 0.000015 |
| SP2 | 6668 | chr17 | 47905400 | 47907999 | high | 6.50 | 0.000139 |
| SP6 | 80320 | chr17 | 47850000 | 47855799 | high | 14.50 | 0.001679 |
| SP7 | 121340 | chr12 | 53330600 | 53334799 | high | 10.50 | 0.002275 |
| SPACA6 | 147650 | chr19 | 51700400 | 51705399 | high | 12.50 | 0.002856 |
| SPAG16 | 79582 | chr2 | 213983600 | 213985399 | low | -7.82 | 0.000372 |
| SPAG16 | 79582 | chr2 | 213977000 | 213978599 | low | -6.95 | 0.000372 |
| SPAG4 | 6676 | chr20 | 35615400 | 35619799 | high | 11.00 | 0.000187 |
| SPAG5 | 10615 | chr17 | 28597400 | 28600199 | high | 7.00 | 0.000371 |
| SPAG5 | 10615 | chr17 | 28581200 | 28584999 | high | 9.50 | 0.000371 |
| SPAG7 | 9552 | chr17 | 4966800 | 4969999 | high | 8.00 | 0.001923 |
| SPAG9 | 9043 | chr17 | 51087400 | 51091399 | high | 10.00 | 0.000177 |
| SPAG9 | 9043 | chr17 | 51118600 | 51122799 | high | 10.50 | 0.000177 |
| SPAG9 | 9043 | chr17 | 51075600 | 51079799 | high | 10.50 | 0.000177 |
| SPAG9 | 9043 | chr17 | 50962600 | 50967599 | high | 12.50 | 0.000177 |
| SPAST | 6683 | chr2 | 32062400 | 32065399 | high | 7.50 | 0.000208 |
| TBK1 | 29110 | chr12 | 64500200 | 64503399 | high | 8.00 | 0.000451 |
| TBK1 | 29110 | chr12 | 64456400 | 64461999 | high | 14.00 | 0.000452 |
| TBP | 6908 | chr6 | 170552400 | 170556999 | high | 11.50 | 0.000041 |
| TBRG1 | 84897 | chr11 | 124622600 | 124623799 | high | 3.00 | 0.000681 |
| TBRG4 | 9238 | chr7 | 45111000 | 45112399 | low | -6.08 | 0.000205 |
| TBX18 | 9096 | chr6 | 84756600 | 84759199 | low | -11.30 | 0.000107 |
| TBX2 | 6909 | chr17 | 61396000 | 61401599 | high | 14.00 | 0.000113 |
| TBX3 | 6926 | chr12 | 114682400 | 114685199 | low | -12.17 | 0.000060 |
| TCAF1 | 9747 | chr7 | 143882800 | 143885399 | low | -11.30 | 0.000068 |
| TCEA1 | 6917 | chr8 | 53985400 | 53989599 | high | 10.50 | 0.000128 |
| TCEA1 | 6917 | chr8 | 54017400 | 54024199 | high | 17.00 | 0.000128 |
| TCEA2 | 6919 | chr20 | 64062600 | 64065799 | high | 8.00 | 0.000108 |
| TCEA3 | 6920 | chr1 | 23401400 | 23405199 | low | -16.52 | 0.000296 |
| TCEANC | 170082 | chrX | 13657400 | 13661199 | high | 9.50 | 0.012453 |
| TCEB3 | 6924 | chr1 | 23753800 | 23757599 | high | 9.50 | 0.000291 |
| TCERG1 | 10915 | chr5 | 146480200 | 146482999 | high | 7.00 | 0.000075 |
| TCF12 | 6938 | chr15 | 56944000 | 56947399 | high | 8.50 | 0.000122 |
| TCF12 | 6938 | chr15 | 57064400 | 57067999 | high | 9.00 | 0.000122 |
| TCF12 | 6938 | chr15 | 57130800 | 57134999 | high | 10.50 | 0.000121 |
| TCF12 | 6938 | chr15 | 57069200 | 57073399 | high | 10.50 | 0.000122 |
| TCF12 | 6938 | chr15 | 56929800 | 56934399 | high | 11.50 | 0.000122 |

| | | | | | | | |
|---|---|---|---|---|---|---|---|
| TCF12 | 6938 | chr15 | 57152400 | 57157399 | high | 12.50 | 0.000121 |
| TCF12 | 6938 | chr15 | 57139400 | 57144399 | high | 12.50 | 0.000121 |
| TCF12 | 6938 | chr15 | 57008600 | 57013599 | high | 12.50 | 0.000122 |
| TCF12 | 6938 | chr15 | 56992200 | 56997199 | high | 12.50 | 0.000122 |
| TCF12 | 6938 | chr15 | 56969200 | 56974199 | high | 12.50 | 0.000122 |
| TCF12 | 6938 | chr15 | 56981600 | 56987999 | high | 16.00 | 0.000122 |
| TCF12 | 6938 | chr15 | 57174800 | 57181999 | high | 18.00 | 0.000121 |
| TCF20 | 6942 | chr22 | 42269000 | 42272799 | high | 9.50 | 0.000164 |
| TCF21 | 6943 | chr6 | 133889000 | 133893599 | high | 11.50 | 0.000052 |
| TCF25 | 22980 | chr16 | 89871800 | 89875599 | high | 9.50 | 0.000256 |
| TCF4 | 6925 | chr18 | 55273000 | 55278599 | high | 14.00 | 0.000125 |
| TCF4 | 6925 | chr18 | 55251400 | 55258999 | high | 19.00 | 0.000125 |
| TCF7 | 6932 | chr5 | 134137800 | 134141199 | high | 8.50 | 0.000052 |
| TCF7 | 6932 | chr5 | 134122200 | 134126399 | high | 10.50 | 0.000052 |
| TCHP | 84260 | chr12 | 109899400 | 109903999 | high | 11.50 | 0.000767 |
| TCIRG1 | 10312 | chr11 | 68039800 | 68044199 | high | 11.00 | 0.000152 |
| TCOF1 | 6949 | chr5 | 150357800 | 150358799 | low | -4.34 | 0.000046 |
| TCP1 | 6950 | chr6 | 159787400 | 159791199 | high | 9.50 | 0.000043 |
| TCP1 | 6950 | chr6 | 159781200 | 159785999 | high | 12.00 | 0.000043 |
| TCTA | 6988 | chr3 | 49409400 | 49413399 | high | 10.00 | 0.000141 |
| TCTN2 | 79867 | chr12 | 123681400 | 123685399 | high | 10.00 | 0.000646 |
| TDG | 6996 | chr12 | 103975200 | 103981199 | high | 15.00 | 0.000067 |
| TDG | 6996 | chr12 | 103964200 | 103970599 | high | 16.00 | 0.000067 |
| TDP1 | 55775 | chr14 | 89970200 | 89973799 | high | 9.00 | 0.000620 |
| TDRD3 | 81550 | chr13 | 60396000 | 60399799 | high | 9.50 | 0.001350 |
| TDRD7 | 23424 | chr9 | 97412000 | 97413999 | high | 5.00 | 0.000240 |
| TEAD1 | 7003 | chr11 | 12717200 | 12721199 | high | 10.00 | 0.000551 |
| TEAD1 | 7003 | chr11 | 12672800 | 12677599 | high | 12.00 | 0.000553 |
| TEAD1 | 7003 | chr11 | 12683400 | 12695999 | high | 31.50 | 0.000552 |
| TEAD2 | 8463 | chr19 | 49359800 | 49363199 | high | 8.50 | 0.000171 |
| TEAD3 | 7005 | chr6 | 35492600 | 35499599 | high | 17.50 | 0.000197 |
| TEAD4 | 7004 | chr12 | 2959000 | 2962999 | low | -17.50 | 0.002367 |
| TEC | 7006 | chr4 | 48162600 | 48164799 | high | 5.50 | 0.000145 |
| TEC | 7006 | chr4 | 48180000 | 48183399 | high | 8.50 | 0.000145 |
| TEC | 7006 | chr4 | 48174800 | 48179199 | high | 11.00 | 0.000145 |
| TEF | 7008 | chr22 | 41379400 | 41383599 | high | 10.50 | 0.000169 |
| TEFM | 79736 | chr17 | 30905400 | 30906999 | low | -6.95 | 0.002580 |
| TELO2 | 9894 | chr16 | 1492800 | 1494399 | high | 4.00 | 0.006628 |
| TERF2 | 7014 | chr16 | 69364400 | 69369199 | high | 12.00 | 0.000101 |
| TES | 26136 | chr7 | 116209600 | 116212399 | high | 7.00 | 0.000225 |
| TES | 26136 | chr7 | 116253200 | 116262999 | high | 24.50 | 0.000225 |
| TESK1 | 7016 | chr9 | 35603200 | 35607799 | high | 11.50 | 0.000197 |
| TESK1 | 7016 | chr9 | 35609600 | 35614599 | high | 12.50 | 0.000197 |
| TESK2 | 10420 | chr1 | 45338200 | 45344599 | high | 16.00 | 0.000230 |
| TET1 | 80312 | chr10 | 68594800 | 68601999 | high | 18.00 | 0.001171 |
| TET2 | 54790 | chr4 | 105208600 | 105211799 | high | 8.00 | 0.000521 |
| TET2 | 54790 | chr4 | 105146000 | 105151199 | high | 13.00 | 0.000521 |
| TEX10 | 54881 | chr9 | 100330800 | 100334999 | high | 10.50 | 0.000547 |
| TEX10 | 54881 | chr9 | 100324400 | 100329999 | high | 14.00 | 0.000547 |
| TEX26 | 122046 | chr13 | 30968200 | 30973399 | high | 13.00 | 0.003941 |
| TEX40 | 25858 | chr11 | 64304000 | 64308599 | high | 11.50 | 0.000402 |
| TEX41 | 401014 | chr2 | 145017400 | 145020199 | high | 7.00 | 0.002765 |
| TEX41 | 401014 | chr2 | 144970400 | 144973599 | high | 8.00 | 0.002766 |
| TEX41 | 401014 | chr2 | 144954600 | 144957799 | high | 8.00 | 0.002766 |
| TEX41 | 401014 | chr2 | 144794800 | 144798199 | high | 8.50 | 0.002770 |
| TEX41 | 401014 | chr2 | 145030000 | 145033799 | high | 9.50 | 0.002765 |
| TEX41 | 401014 | chr2 | 144711800 | 144715799 | high | 10.00 | 0.002771 |
| TEX41 | 401014 | chr2 | 145011200 | 145016599 | high | 13.50 | 0.002765 |
| TEX41 | 401014 | chr2 | 144974600 | 144979999 | high | 13.50 | 0.002766 |
| TFAP2C | 7022 | chr20 | 56629000 | 56630799 | low | -7.82 | 0.000124 |
| TFAP4 | 7023 | chr16 | 4261800 | 4265199 | high | 8.50 | 0.001648 |
| TFAP4 | 7023 | chr16 | 4269800 | 4273999 | high | 10.50 | 0.001645 |
| TFDP1 | 7027 | chr13 | 113584400 | 113588399 | high | 10.00 | 0.000062 |
| TFEB | 7942 | chr6 | 41716400 | 41718799 | low | -10.43 | 0.000190 |
| TFPI2 | 7980 | chr7 | 93890400 | 93895199 | high | 12.00 | 0.000085 |
| TFRC | 7037 | chr3 | 196080400 | 196082399 | high | 5.00 | 0.000036 |
| TGFB2 | 7042 | chr1 | 218415400 | 218417799 | high | 6.00 | 0.000032 |
| TGFB2 | 7042 | chr1 | 218350600 | 218355799 | high | 13.00 | 0.000032 |
| TGFBI | 7045 | chr5 | 136049400 | 136054599 | high | 13.00 | 0.000052 |
| TGFBR1 | 7046 | chr9 | 99141400 | 99143599 | high | 5.50 | 0.000071 |
| TGFBR3 | 7049 | chr1 | 91731000 | 91732399 | high | 3.50 | 0.000077 |
| TGFBR3 | 7049 | chr1 | 91745400 | 91748799 | high | 8.50 | 0.000077 |
| TGFBR3 | 7049 | chr1 | 91740600 | 91744399 | high | 9.50 | 0.000077 |
| TGFBR3 | 7049 | chr1 | 91753600 | 91759399 | high | 14.50 | 0.000077 |
| TGIF2 | 60436 | chr20 | 36573000 | 36574999 | high | 5.00 | 0.001652 |
| TGM2 | 7052 | chr20 | 38137800 | 38146799 | high | 22.50 | 0.000185 |
| TGS1 | 96764 | chr8 | 55780600 | 55783599 | high | 7.50 | 0.001735 |
| THAP11 | 57215 | chr16 | 67841600 | 67847799 | high | 15.50 | 0.000843 |

| | | | | | | | |
|---|---|---|---|---|---|---|---|
| THAP3 | 90326 | chr1 | 6624600 | 6625999 | low | -6.08 | 0.013635 |
| THAP4 | 51078 | chr2 | 241610000 | 241611199 | high | 3.00 | 0.000211 |
| THBS1 | 7057 | chr15 | 39592600 | 39594599 | low | -8.69 | 0.000178 |
| THNSL1 | 79896 | chr10 | 25014800 | 25016599 | high | 4.50 | 0.003194 |
| THOC1 | 9984 | chr18 | 266200 | 268399 | high | 5.50 | 0.004167 |
| THOC1 | 9984 | chr18 | 215600 | 219199 | high | 9.00 | 0.000365 |
| THOC1 | 9984 | chr18 | 243800 | 250599 | high | 17.00 | 0.004550 |
| THOC5 | 8563 | chr22 | 29548800 | 29550799 | low | -8.69 | 0.000290 |
| THOC5 | 8563 | chr22 | 29523800 | 29525599 | low | -7.82 | 0.000290 |
| THOC5 | 8563 | chr22 | 29553000 | 29554399 | low | -6.08 | 0.000290 |
| THOC7 | 80145 | chr3 | 63848400 | 63851599 | high | 8.00 | 0.001255 |
| THOC7 | 80145 | chr3 | 63854200 | 63859599 | high | 13.50 | 0.001255 |
| THOP1 | 7064 | chr19 | 2784400 | 2786599 | low | -9.56 | 0.002537 |
| THRAP3 | 9967 | chr1 | 36280000 | 36285799 | high | 14.50 | 0.000275 |
| THRAP3 | 9967 | chr1 | 36247000 | 36256199 | high | 23.00 | 0.000275 |
| THRB | 7068 | chr3 | 24255000 | 24259399 | high | 11.00 | 0.000291 |
| THSD7A | 221981 | chr7 | 11824000 | 11826999 | low | -13.04 | 0.018774 |
| THSD7A | 221981 | chr7 | 11738000 | 11740399 | low | -10.43 | 0.018911 |
| TIFA | 92610 | chr4 | 112285000 | 112286199 | low | -5.21 | 0.000825 |
| TIGD4 | 201798 | chr4 | 152779000 | 152782999 | high | 10.00 | 0.001321 |
| TIMM9 | 26520 | chr14 | 58411600 | 58414199 | high | 6.50 | 0.000454 |
| TIMM9 | 26520 | chr14 | 58423800 | 58429599 | high | 14.50 | 0.000454 |
| TINAG | 27283 | chr6 | 54318000 | 54321199 | low | -13.91 | 0.000502 |
| TINAG | 27283 | chr6 | 54348400 | 54351399 | low | -13.04 | 0.000502 |
| TIPARP | 25976 | chr3 | 156681600 | 156685799 | high | 10.50 | 0.000166 |
| TIPARP | 25976 | chr3 | 156686600 | 156692399 | high | 14.50 | 0.000166 |
| TIPARP | 25976 | chr3 | 156672800 | 156679199 | high | 16.00 | 0.000166 |
| TIPIN | 54962 | chr15 | 66353000 | 66354599 | low | -6.95 | 0.000828 |
| TIPIN | 54962 | chr15 | 66355600 | 66356599 | low | -4.34 | 0.000828 |
| TIPRL | 261726 | chr1 | 168195000 | 168198199 | high | 8.00 | 0.001556 |
| TJAP1 | 93643 | chr6 | 43492200 | 43497399 | high | 13.00 | 0.002153 |
| TLE1 | 7088 | chr9 | 81679800 | 81686399 | high | 16.50 | 0.000087 |
| TLE4 | 7091 | chr9 | 79691400 | 79694599 | high | 8.00 | 0.000089 |
| TLK1 | 9874 | chr2 | 171130400 | 171134999 | high | 11.50 | 0.000058 |
| TLK1 | 9874 | chr2 | 171122400 | 171127799 | high | 13.50 | 0.000058 |
| TLN1 | 7094 | chr9 | 35732000 | 35734599 | high | 6.50 | 0.000199 |
| TLN2 | 83660 | chr15 | 62667400 | 62672399 | high | 12.50 | 0.001335 |
| TLR2 | 7097 | chr4 | 153694800 | 153700399 | high | 14.00 | 0.000046 |
| TM2D3 | 80213 | chr15 | 101650200 | 101654199 | high | 10.00 | 0.000789 |
| TMA7 | 51372 | chr3 | 48439800 | 48442399 | high | 6.50 | 0.001061 |
| TMC1 | 117531 | chr9 | 72579600 | 72586199 | high | 16.50 | 0.001619 |
| TMCC1 | 23023 | chr3 | 129657000 | 129661199 | high | 10.50 | 0.000178 |
| TMCC1 | 23023 | chr3 | 129728600 | 129733199 | high | 11.50 | 0.000177 |
| TMCC3 | 57458 | chr12 | 94612600 | 94618199 | high | 14.00 | 0.000607 |
| TMCO3 | 55002 | chr13 | 113507600 | 113509999 | high | 6.00 | 0.000485 |
| TMCO4 | 255104 | chr1 | 19765800 | 19769799 | high | 10.00 | 0.012906 |
| TMED1 | 11018 | chr19 | 10833800 | 10836399 | high | 6.50 | 0.001017 |
| TMED10 | 10972 | chr14 | 75139000 | 75143199 | high | 10.50 | 0.000146 |
| TMED3 | 23423 | chr15 | 79310800 | 79313799 | low | -13.04 | 0.000295 |
| TMED4 | 222068 | chr7 | 44580400 | 44582799 | high | 6.00 | 0.004981 |
| TMED5 | 50999 | chr1 | 93180000 | 93182599 | high | 6.50 | 0.000547 |
| TMED6 | 146456 | chr16 | 69342400 | 69344599 | low | -9.56 | 0.002112 |
| TMED8 | 283578 | chr14 | 77376200 | 77378799 | high | 6.50 | 0.003665 |
| USP37 | 57695 | chr2 | 218566600 | 218574599 | high | 20.00 | 0.000264 |
| USP4 | 7375 | chr3 | 49274600 | 49277999 | high | 8.50 | 0.000150 |
| USP42 | 84132 | chr7 | 6104000 | 6106999 | high | 7.50 | 0.013783 |
| USP42 | 84132 | chr7 | 6135800 | 6142999 | high | 18.00 | 0.013712 |
| USP45 | 85015 | chr6 | 99512000 | 99515599 | high | 9.00 | 0.000854 |
| USP45 | 85015 | chr6 | 99484000 | 99487799 | high | 9.50 | 0.000855 |
| USP46 | 64854 | chr4 | 52658600 | 52661599 | low | -13.04 | 0.001232 |
| USP46 | 64854 | chr4 | 52627000 | 52629799 | low | -12.17 | 0.001232 |
| USP47 | 55031 | chr11 | 11950400 | 11954399 | high | 10.00 | 0.004605 |
| USP47 | 55031 | chr11 | 11894000 | 11901199 | high | 18.00 | 0.004627 |
| USP48 | 84196 | chr1 | 21763000 | 21766599 | high | 9.00 | 0.003869 |
| USP48 | 84196 | chr1 | 21736600 | 21742799 | high | 15.50 | 0.003873 |
| USP49 | 25862 | chr6 | 41891200 | 41896399 | high | 13.00 | 0.000617 |
| USP5 | 8078 | chr12 | 6863000 | 6865399 | low | -10.43 | 0.001177 |
| USP5 | 8078 | chr12 | 6854000 | 6855399 | low | -6.08 | 0.001179 |
| USP54 | 159195 | chr10 | 73494600 | 73497799 | low | -13.91 | 0.002166 |
| USP54 | 159195 | chr10 | 73515400 | 73516999 | low | -6.95 | 0.002165 |
| USP7 | 7874 | chr16 | 8961400 | 8964599 | low | -13.91 | 0.000879 |
| UST | 10090 | chr6 | 148937800 | 148942199 | high | 11.00 | 0.000068 |
| UST | 10090 | chr6 | 149036800 | 149042799 | high | 15.00 | 0.000068 |
| UST | 10090 | chr6 | 148865800 | 148872599 | high | 17.00 | 0.000068 |
| UTP23 | 84294 | chr8 | 116765800 | 116767599 | high | 4.50 | 0.000722 |
| UTP3 | 57050 | chr4 | 70687800 | 70689199 | low | -6.08 | 0.000807 |
| UTP6 | 55813 | chr17 | 31862200 | 31864199 | low | -8.69 | 0.001752 |
| UTP6 | 55813 | chr17 | 31900800 | 31901999 | low | -5.21 | 0.001750 |

| | | | | | | | |
|---|---|---|---|---|---|---|---|
| UTRN | 7402 | chr6 | 144570000 | 144572199 | high | 5.50 | 0.000051 |
| UTRN | 7402 | chr6 | 144430200 | 144432599 | high | 6.00 | 0.000051 |
| UTRN | 7402 | chr6 | 144499400 | 144501999 | high | 6.50 | 0.000051 |
| UTRN | 7402 | chr6 | 144410600 | 144413399 | high | 7.00 | 0.000051 |
| UTRN | 7402 | chr6 | 144406400 | 144409599 | high | 8.00 | 0.000051 |
| UTRN | 7402 | chr6 | 144316200 | 144319399 | high | 8.00 | 0.000051 |
| UTRN | 7402 | chr6 | 144320800 | 144324399 | high | 9.00 | 0.000051 |
| UTRN | 7402 | chr6 | 144299600 | 144303199 | high | 9.00 | 0.000051 |
| UTRN | 7402 | chr6 | 144689400 | 144693199 | high | 9.50 | 0.000051 |
| UTRN | 7402 | chr6 | 144377000 | 144380799 | high | 9.50 | 0.000051 |
| UTRN | 7402 | chr6 | 144337000 | 144340999 | high | 10.00 | 0.000051 |
| UTRN | 7402 | chr6 | 144310200 | 144314199 | high | 10.00 | 0.000051 |
| UTRN | 7402 | chr6 | 144399000 | 144403399 | high | 11.00 | 0.000051 |
| UTRN | 7402 | chr6 | 144386200 | 144391199 | high | 12.50 | 0.000051 |
| UTRN | 7402 | chr6 | 144581600 | 144586799 | high | 13.00 | 0.000051 |
| UTRN | 7402 | chr6 | 144562000 | 144567799 | high | 14.50 | 0.000051 |
| UTS2B | 257313 | chr3 | 191295400 | 191298199 | low | -12.17 | 0.001345 |
| UTS2B | 257313 | chr3 | 191329200 | 191330999 | low | -7.82 | 0.001345 |
| VAC14 | 55697 | chr16 | 70692000 | 70694399 | high | 6.00 | 0.000788 |
| VAC14 | 55697 | chr16 | 70772000 | 70776399 | high | 11.00 | 0.000787 |
| VAC14 | 55697 | chr16 | 70705800 | 70710399 | high | 11.50 | 0.000788 |
| VAC14 | 55697 | chr16 | 70778400 | 70783599 | high | 13.00 | 0.000787 |
| VAC14 | 55697 | chr16 | 70711400 | 70716599 | high | 13.00 | 0.000788 |
| VAC14 | 55697 | chr16 | 70718000 | 70727399 | high | 23.50 | 0.000788 |
| VAMP2 | 6844 | chr17 | 8160600 | 8164999 | high | 11.00 | 0.000839 |
| VAMP4 | 8674 | chr1 | 171726800 | 171729599 | low | -12.17 | 0.000051 |
| VAMP5 | 10791 | chr2 | 85583600 | 85588799 | high | 13.00 | 0.000126 |
| VAMP8 | 8673 | chr2 | 85578400 | 85579999 | low | -6.95 | 0.000101 |
| VAPA | 9218 | chr18 | 9947400 | 9950399 | low | -13.04 | 0.000927 |
| VAPA | 9218 | chr18 | 9932400 | 9933599 | low | -5.21 | 0.000928 |
| VAPB | 9217 | chr20 | 58387200 | 58391399 | low | -18.34 | 0.000158 |
| VAT1 | 10493 | chr17 | 43021200 | 43022399 | low | -5.21 | 0.000244 |
| VCAM1 | 7412 | chr1 | 100722000 | 100725199 | high | 8.00 | 0.000074 |
| VCAM1 | 7412 | chr1 | 100733600 | 100738199 | high | 11.50 | 0.000074 |
| VCAN | 1462 | chr5 | 83547600 | 83550199 | high | 6.50 | 0.000017 |
| VCAN | 1462 | chr5 | 83502800 | 83506799 | high | 10.00 | 0.000018 |
| VCAN | 1462 | chr5 | 83484800 | 83488799 | high | 10.00 | 0.000018 |
| VCAN | 1462 | chr5 | 83539600 | 83543999 | high | 11.00 | 0.000018 |
| VCAN | 1462 | chr5 | 83490000 | 83494599 | high | 11.50 | 0.000018 |
| VCAN | 1462 | chr5 | 83515200 | 83521399 | high | 15.50 | 0.000018 |
| VCAN | 1462 | chr5 | 83577000 | 83583799 | high | 17.00 | 0.000017 |
| VCAN | 1462 | chr5 | 83470400 | 83477599 | high | 18.00 | 0.000018 |
| VCL | 7414 | chr10 | 74006000 | 74008399 | high | 6.00 | 0.000100 |
| VCL | 7414 | chr10 | 74045400 | 74047999 | high | 6.50 | 0.000100 |
| VCL | 7414 | chr10 | 74062400 | 74065599 | high | 8.00 | 0.000100 |
| VCL | 7414 | chr10 | 74041200 | 74044399 | high | 8.00 | 0.000100 |
| VDR | 7421 | chr12 | 47867600 | 47871799 | high | 10.50 | 0.000155 |
| VEGFC | 7424 | chr4 | 176774600 | 176780199 | high | 14.00 | 0.000042 |
| VEPH1 | 79674 | chr3 | 157392600 | 157394799 | high | 5.50 | 0.000506 |
| VEPH1 | 79674 | chr3 | 157369400 | 157372599 | high | 8.00 | 0.000506 |
| VEPH1 | 79674 | chr3 | 157407200 | 157412199 | high | 12.50 | 0.000506 |
| VEPH1 | 79674 | chr3 | 157299200 | 157305799 | high | 16.50 | 0.000507 |
| VEPH1 | 79674 | chr3 | 157414200 | 157421599 | high | 18.50 | 0.000506 |
| VEPH1 | 79674 | chr3 | 157360200 | 157367799 | high | 19.00 | 0.000506 |
| VEPH1 | 79674 | chr3 | 157276000 | 157283799 | high | 19.50 | 0.000507 |
| VEZF1 | 7716 | chr17 | 57986000 | 57989999 | high | 10.00 | 0.000133 |
| VEZF1 | 7716 | chr17 | 57980000 | 57985199 | high | 13.00 | 0.000133 |
| VEZT | 55591 | chr12 | 95217000 | 95218799 | high | 4.50 | 0.000584 |
| VEZT | 55591 | chr12 | 95265600 | 95270399 | high | 12.00 | 0.000584 |
| VEZT | 55591 | chr12 | 95251200 | 95256199 | high | 12.50 | 0.000584 |
| VGLL3 | 389136 | chr3 | 86937000 | 86940999 | high | 10.00 | 0.004476 |
| VGLL4 | 9686 | chr3 | 11569400 | 11572199 | high | 7.00 | 0.000837 |
| VGLL4 | 9686 | chr3 | 11719000 | 11722399 | high | 8.50 | 0.000827 |
| VGLL4 | 9686 | chr3 | 11598400 | 11602599 | high | 10.50 | 0.000835 |
| VGLL4 | 9686 | chr3 | 11556600 | 11562799 | high | 15.50 | 0.000838 |
| VHL | 7428 | chr3 | 10140400 | 10143199 | high | 7.00 | 0.000733 |
| VHL | 7428 | chr3 | 10144000 | 10149799 | high | 14.50 | 0.000732 |
| VIMP | 55829 | chr15 | 101271000 | 101272999 | high | 5.00 | 0.000551 |
| VMAC | 400673 | chr19 | 5900400 | 5905999 | high | 14.00 | 0.007545 |
| VMP1 | 81671 | chr17 | 59810000 | 59813599 | high | 9.00 | 0.001366 |
| VMP1 | 81671 | chr17 | 59751800 | 59757799 | high | 15.00 | 0.001367 |
| VMP1 | 81671 | chr17 | 59826200 | 59832399 | high | 15.50 | 0.001365 |
| VMP1 | 81671 | chr17 | 59792800 | 59799199 | high | 16.00 | 0.001366 |
| VMP1 | 81671 | chr17 | 59816400 | 59822999 | high | 16.50 | 0.001365 |
| VMP1 | 81671 | chr17 | 59760800 | 59767799 | high | 17.50 | 0.001367 |
| VMP1 | 81671 | chr17 | 59781600 | 59788999 | high | 18.50 | 0.001366 |
| VNN2 | 8875 | chr6 | 132748800 | 132753399 | high | 11.50 | 0.000067 |
| VNN3 | 55350 | chr6 | 132728600 | 132736599 | high | 20.00 | 0.000417 |

| | | | | | | | |
|---|---|---|---|---|---|---|---|
| VOPP1 | 81552 | chr7 | 55552000 | 55558599 | high | 16.50 | 0.001468 |
| VPS11 | 55823 | chr11 | 119077000 | 119079399 | low | -10.43 | 0.000469 |
| VPS13D | 55187 | chr1 | 12452400 | 12457199 | high | 12.00 | 0.004432 |
| VPS16 | 64601 | chr20 | 2840600 | 2843399 | high | 7.00 | 0.002527 |
| VPS16 | 64601 | chr20 | 2858600 | 2863599 | high | 12.50 | 0.002511 |
| VPS18 | 57617 | chr15 | 40893000 | 40895999 | high | 7.50 | 0.001409 |
| VPS18 | 57617 | chr15 | 40892800 | 40900199 | high | 18.50 | 0.001409 |
| VPS25 | 84313 | chr17 | 42771800 | 42775599 | high | 9.50 | 0.001971 |
| VPS26B | 112936 | chr11 | 134220400 | 134225399 | high | 12.50 | 0.000841 |
| VPS28 | 51160 | chr8 | 144427600 | 144428399 | low | -3.47 | 0.000354 |
| VPS29 | 51699 | chr12 | 110500200 | 110504199 | high | 10.00 | 0.000468 |
| VPS33A | 65082 | chr12 | 122257600 | 122260399 | high | 7.00 | 0.000532 |
| VPS37C | 55048 | chr11 | 61149800 | 61154799 | high | 12.50 | 0.000900 |
| VPS37D | 155382 | chr7 | 73668000 | 73670399 | high | 6.00 | 0.002109 |
| VPS45 | 11311 | chr1 | 150102600 | 150103999 | high | 3.50 | 0.000075 |
| VPS4A | 27183 | chr16 | 69310400 | 69312399 | low | -8.69 | 0.000392 |
| VPS4B | 9525 | chr18 | 63420400 | 63423599 | high | 8.00 | 0.000150 |
| VPS53 | 55275 | chr17 | 553400 | 557399 | high | 8.00 | 0.011098 |
| VPS53 | 55275 | chr17 | 681800 | 689399 | high | 15.20 | 0.009008 |
| VPS54 | 51542 | chr2 | 63890000 | 63892799 | high | 5.60 | 0.000807 |
| VPS8 | 23355 | chr3 | 184818000 | 184821399 | high | 6.80 | 0.000126 |
| VPS8 | 23355 | chr3 | 184811200 | 184816599 | high | 10.80 | 0.000126 |
| VPS8 | 23355 | chr3 | 184990600 | 184996199 | high | 11.20 | 0.000126 |
| VRK1 | 7443 | chr14 | 96817600 | 96818999 | low | -6.08 | 0.000077 |
| VRK2 | 7444 | chr2 | 58025000 | 58028399 | high | 6.80 | 0.000128 |
| VRK2 | 7444 | chr2 | 58098200 | 58102999 | high | 9.60 | 0.000128 |
| VRK2 | 7444 | chr2 | 58106000 | 58111599 | high | 11.20 | 0.000128 |
| VRTN | 55237 | chr14 | 74354200 | 74357999 | high | 7.60 | 0.000743 |
| VSIG8 | 391123 | chr1 | 159852200 | 159856799 | high | 9.20 | 0.002447 |
| VSNL1 | 7447 | chr2 | 17614600 | 17618599 | low | -17.37 | 0.000423 |
| VTA1 | 51534 | chr6 | 142178800 | 142180399 | high | 3.20 | 0.000362 |
| VTI1B | 10490 | chr14 | 67674000 | 67675399 | low | -6.08 | 0.000155 |
| VWCE | 220001 | chr11 | 61272000 | 61275599 | high | 7.20 | 0.003591 |
| WAC | 51322 | chr10 | 28586600 | 28591999 | high | 10.80 | 0.001795 |
| WASF2 | 10163 | chr1 | 27429400 | 27434599 | high | 10.40 | 0.000371 |
| WASF2 | 10163 | chr1 | 27413400 | 27420199 | high | 13.60 | 0.000371 |
| WBP1L | 54838 | chr10 | 102776600 | 102779199 | high | 5.20 | 0.000534 |
| WBP1L | 54838 | chr10 | 102772600 | 102775399 | high | 5.60 | 0.000534 |
| WBP1L | 54838 | chr10 | 102745200 | 102747999 | high | 5.60 | 0.000534 |
| WBP4 | 11193 | chr13 | 41059400 | 41065999 | high | 13.20 | 0.000273 |
| WDCP | 80304 | chr2 | 24043200 | 24047999 | high | 9.60 | 0.003340 |
| WDFY2 | 115825 | chr13 | 51582400 | 51587599 | high | 10.40 | 0.002245 |
| WDFY2 | 115825 | chr13 | 51601800 | 51608599 | high | 13.60 | 0.002245 |
| WDFY3 | 23001 | chr4 | 84844000 | 84847599 | high | 7.20 | 0.000271 |
| WDFY3 | 23001 | chr4 | 84952800 | 84956599 | high | 7.60 | 0.000271 |
| WDFY3 | 23001 | chr4 | 84725000 | 84729199 | high | 8.40 | 0.000271 |
| WDFY3 | 23001 | chr4 | 84689200 | 84694599 | high | 10.80 | 0.000272 |
| WDFY3 | 23001 | chr4 | 84875600 | 84881199 | high | 11.20 | 0.000271 |
| WDHD1 | 11169 | chr14 | 55002800 | 55006199 | high | 6.80 | 0.000203 |
| WDHD1 | 11169 | chr14 | 54976200 | 54979999 | high | 7.60 | 0.000203 |
| WDHD1 | 11169 | chr14 | 55026800 | 55033999 | high | 14.40 | 0.000203 |
| WDR1 | 9948 | chr4 | 10100400 | 10104799 | high | 8.80 | 0.000985 |
| WDR12 | 55759 | chr2 | 202909200 | 202912199 | low | -13.04 | 0.000275 |
| WDR18 | 57418 | chr19 | 983200 | 985599 | low | -10.43 | 0.006489 |
| WDR20 | 91833 | chr14 | 102217400 | 102220399 | high | 6.00 | 0.000898 |
| WDR20 | 91833 | chr14 | 102190600 | 102196999 | high | 12.80 | 0.000899 |
| WDR26 | 80232 | chr1 | 224400000 | 224401999 | high | 4.00 | 0.000358 |
| WDR27 | 253769 | chr6 | 169701200 | 169703799 | high | 5.20 | 0.001495 |
| WDR34 | 89891 | chr9 | 128634400 | 128638199 | high | 7.60 | 0.000699 |
| WDR4 | 10785 | chr21 | 42877600 | 42880399 | low | -12.17 | 0.000252 |
| WDR43 | 23160 | chr2 | 28900000 | 28905399 | high | 10.80 | 0.000801 |
| WDR43 | 23160 | chr2 | 28926200 | 28932199 | high | 12.00 | 0.000801 |
| WDR44 | 54521 | chrX | 118346000 | 118348599 | high | 5.20 | 0.000461 |
| WDR45B | 56270 | chr17 | 82630600 | 82633799 | high | 6.40 | 0.000681 |
| WDR47 | 22911 | chr1 | 108980200 | 108986399 | high | 12.40 | 0.000210 |
| WDR5 | 11091 | chr9 | 134135800 | 134139399 | high | 7.20 | 0.000083 |
| WDR53 | 348793 | chr3 | 196568000 | 196570399 | low | -10.43 | 0.001774 |
| WDR66 | 144406 | chr12 | 121939200 | 121942599 | high | 6.80 | 0.001184 |
| WDR66 | 144406 | chr12 | 121960600 | 121964599 | high | 8.00 | 0.001184 |
| WDR66 | 144406 | chr12 | 121915600 | 121919599 | high | 8.00 | 0.001184 |
| WDR66 | 144406 | chr12 | 121951200 | 121957999 | high | 13.60 | 0.001184 |
| WDR7 | 23335 | chr18 | 56650600 | 56652799 | high | 4.40 | 0.000412 |
| WDR70 | 55100 | chr5 | 37494200 | 37497599 | high | 6.80 | 0.001470 |
| WDR70 | 55100 | chr5 | 37546000 | 37549599 | high | 7.20 | 0.001468 |
| WDR70 | 55100 | chr5 | 37694000 | 37697799 | high | 7.60 | 0.001462 |
| WDR70 | 55100 | chr5 | 37702600 | 37706799 | high | 8.40 | 0.001461 |
| WDR73 | 84942 | chr15 | 84654200 | 84656799 | low | -11.30 | 0.001003 |
| WDR76 | 79968 | chr15 | 43825800 | 43828199 | low | -10.43 | 0.001825 |

| | | | | | | | |
|---|---|---|---|---|---|---|---|
| WDR77 | 79084 | chr1 | 111448800 | 111449799 | low | -4.34 | 0.000710 |
| WDR78 | 79819 | chr1 | 66919200 | 66921199 | high | 4.00 | 0.001193 |
| WDR78 | 79819 | chr1 | 66848800 | 66852599 | high | 7.60 | 0.001194 |
| WDR78 | 79819 | chr1 | 66893200 | 66897199 | high | 8.00 | 0.001193 |
| WDR82 | 80335 | chr3 | 52276600 | 52280199 | high | 7.20 | 0.001537 |
| WDR83 | 84292 | chr19 | 12666000 | 12667999 | high | 4.00 | 0.006655 |
| WDR83 | 84292 | chr19 | 12675200 | 12680199 | high | 10.00 | 0.006650 |
| WDR87 | 83889 | chr19 | 37904800 | 37908599 | high | 7.60 | 0.002213 |
| WDR90 | 197335 | chr16 | 665000 | 671399 | high | 12.80 | 0.009023 |
| WDSUB1 | 151525 | chr2 | 159278800 | 159287399 | high | 17.20 | 0.000951 |
| WDSUB1 | 151525 | chr2 | 159232400 | 159241599 | high | 18.40 | 0.000952 |
| WEE1 | 7465 | chr11 | 9574000 | 9576999 | low | -13.04 | 0.000780 |
| WHAMM | 123720 | chr15 | 82809400 | 82811199 | high | 3.60 | 0.001494 |
| WHSC1 | 7468 | chr4 | 1922000 | 1926199 | high | 8.40 | 0.003886 |
| WHSC1 | 7468 | chr4 | 1869800 | 1875199 | high | 10.80 | 0.003994 |
| WHSC1 | 7468 | chr4 | 1972000 | 1977599 | high | 11.20 | 0.003787 |
| WHSC1 | 7468 | chr4 | 1898000 | 1904399 | high | 12.80 | 0.003935 |
| WIPF1 | 7456 | chr2 | 174566600 | 174569399 | high | 5.60 | 0.000043 |
| WIPF2 | 147179 | chr17 | 40272800 | 40276999 | high | 8.40 | 0.003655 |
| WIPI2 | 26100 | chr7 | 5196200 | 5201599 | high | 10.80 | 0.005023 |
| WISP1 | 8840 | chr8 | 133202000 | 133207799 | high | 11.60 | 0.000066 |
| WIZ | 58525 | chr19 | 15447200 | 15451799 | high | 9.20 | 0.003789 |
| WNK1 | 65125 | chr12 | 841400 | 843799 | high | 4.80 | 0.008600 |
| WNK1 | 65125 | chr12 | 868400 | 871599 | high | 6.40 | 0.008333 |
| WNK1 | 65125 | chr12 | 860200 | 863999 | high | 7.60 | 0.008412 |
| WNK1 | 65125 | chr12 | 905200 | 909199 | high | 8.00 | 0.007994 |
| WNK1 | 65125 | chr12 | 878000 | 883199 | high | 10.40 | 0.009681 |
| WNK4 | 65266 | chr17 | 42792400 | 42798999 | high | 13.20 | 0.001525 |
| WNT10B | 7480 | chr12 | 48968000 | 48971399 | high | 6.80 | 0.000153 |
| WNT16 | 51384 | chr7 | 121333400 | 121335999 | high | 5.20 | 0.000423 |
| WNT5A | 7474 | chr3 | 55486000 | 55489199 | high | 6.40 | 0.000135 |
| WNT5B | 81029 | chr12 | 1634600 | 1636399 | high | 3.60 | 0.005508 |
| WNT5B | 81029 | chr12 | 1640200 | 1646599 | high | 12.80 | 0.005489 |
| WNT7B | 7477 | chr22 | 45965000 | 45970399 | high | 10.80 | 0.000163 |
| WRNIP1 | 56897 | chr6 | 2765000 | 2768199 | high | 6.40 | 0.002286 |
| WRNIP1 | 56897 | chr6 | 2769000 | 2773399 | high | 8.80 | 0.002283 |
| WSB1 | 26118 | chr17 | 27300400 | 27303399 | low | -13.04 | 0.000957 |
| WSB1 | 26118 | chr17 | 27293600 | 27296599 | low | -13.04 | 0.000957 |
| WTAP | 9589 | chr6 | 159726400 | 159728999 | low | -11.30 | 0.000060 |
| WWC1 | 23286 | chr5 | 168379000 | 168383799 | high | 9.60 | 0.000138 |
| WWC2 | 80014 | chr4 | 183138000 | 183142799 | high | 9.60 | 0.000437 |
| WWP1 | 11059 | chr8 | 86442600 | 86445599 | low | -13.04 | 0.000128 |
| WWP2 | 11060 | chr16 | 69851600 | 69857799 | high | 12.40 | 0.000158 |
| WWP2 | 11060 | chr16 | 69840000 | 69849599 | high | 19.20 | 0.000158 |
| WWTR1 | 25937 | chr3 | 149596600 | 149600799 | high | 8.40 | 0.000173 |
| WWTR1 | 25937 | chr3 | 149630600 | 149635199 | high | 9.20 | 0.000173 |
| XDH | 7498 | chr2 | 31368200 | 31373199 | high | 10.00 | 0.000239 |
| XIRP2 | 129446 | chr2 | 167156400 | 167161199 | high | 9.60 | 0.000774 |
| XIST | 7503 | chrX | 73850800 | 73853599 | high | 5.60 | 0.000102 |
| XKR8 | 55113 | chr1 | 27957400 | 27963599 | high | 12.40 | 0.001971 |
| XPA | 7507 | chr9 | 97695200 | 97697799 | high | 5.20 | 0.000077 |
| XPC | 7508 | chr3 | 14178200 | 14180199 | low | -8.69 | 0.000530 |
| XPO4 | 64328 | chr13 | 20876800 | 20878999 | low | -9.56 | 0.003081 |
| XPO4 | 64328 | chr13 | 20901800 | 20903199 | low | -6.08 | 0.003078 |
| XPO5 | 57510 | chr6 | 43544400 | 43549799 | high | 10.80 | 0.001321 |
| XPO7 | 23039 | chr8 | 21947400 | 21952199 | high | 9.60 | 0.001050 |
| XPO7 | 23039 | chr8 | 21934000 | 21938799 | high | 9.60 | 0.001050 |
| XPO7 | 23039 | chr8 | 21927800 | 21932599 | high | 9.60 | 0.001051 |
| XPO7 | 23039 | chr8 | 21917600 | 21924799 | high | 14.40 | 0.001051 |
| XPOT | 11260 | chr12 | 64404000 | 64409999 | high | 12.00 | 0.000175 |
| XPR1 | 9213 | chr1 | 180631400 | 180632799 | low | -6.08 | 0.000051 |
| XRN1 | 54464 | chr3 | 142417600 | 142420799 | high | 6.40 | 0.000382 |
| XRN1 | 54464 | chr3 | 142446200 | 142455999 | high | 19.60 | 0.000382 |
| XRN2 | 22803 | chr20 | 21302200 | 21305199 | high | 6.00 | 0.001070 |
| XYLT1 | 64131 | chr16 | 17361000 | 17365799 | high | 9.60 | 0.003694 |
| YAE1D1 | 57002 | chr7 | 39592400 | 39594799 | low | -10.43 | 0.001440 |
| YAE1D1 | 57002 | chr7 | 39608400 | 39610399 | low | -8.69 | 0.001439 |
| YAF2 | 10138 | chr12 | 42237000 | 42238399 | high | 2.80 | 0.000240 |
| YAP1 | 10413 | chr11 | 102182200 | 102184799 | high | 5.20 | 0.000102 |
| YAP1 | 10413 | chr11 | 102199600 | 102203399 | high | 7.60 | 0.000102 |
| YAP1 | 10413 | chr11 | 102211200 | 102216599 | high | 10.80 | 0.000102 |
| YARS | 8565 | chr1 | 32772000 | 32775599 | high | 7.20 | 0.000261 |
| YARS | 8565 | chr1 | 32797400 | 32801799 | high | 8.80 | 0.000261 |
| YARS2 | 51067 | chr12 | 32754600 | 32756599 | low | -8.69 | 0.001559 |
| YBEY | 54059 | chr21 | 46284200 | 46289199 | high | 10.00 | 0.001168 |
| YBX1 | 4904 | chr1 | 42681200 | 42683799 | low | -11.30 | 0.000115 |
| YBX3 | 8531 | chr12 | 10707400 | 10711999 | high | 9.20 | 0.000797 |
| YES1 | 7525 | chr18 | 772800 | 777199 | high | 8.80 | 0.009737 |

| | | | | | | | |
|---|---|---|---|---|---|---|---|
| YIPF3 | 25844 | chr6 | 43515600 | 43519999 | low | -19.39 | 0.000594 |
| YIPF4 | 84272 | chr2 | 32281000 | 32284399 | high | 6.80 | 0.002611 |
| YIPF5 | 81555 | chr5 | 144155600 | 144163999 | high | 16.80 | 0.000566 |
| YKT6 | 10652 | chr7 | 44200800 | 44201999 | low | -5.21 | 0.000241 |
| YME1L1 | 10730 | chr10 | 27145000 | 27150599 | high | 11.20 | 0.000395 |
| YME1L1 | 10730 | chr10 | 27118600 | 27125999 | high | 14.80 | 0.000396 |
| YPEL3 | 83719 | chr16 | 30092800 | 30097999 | high | 10.40 | 0.002782 |
| YTHDF2 | 51441 | chr1 | 28737000 | 28740599 | high | 7.20 | 0.001790 |
| YTHDF3 | 253943 | chr8 | 63167400 | 63169999 | low | -11.30 | 0.004020 |
| YTHDF3 | 253943 | chr8 | 63170800 | 63172999 | low | -9.56 | 0.004020 |
| YWHAB | 7529 | chr20 | 44898800 | 44901399 | high | 5.20 | 0.000168 |
| YWHAB | 7529 | chr20 | 44885000 | 44888199 | high | 6.40 | 0.000168 |
| YWHAQ | 10971 | chr2 | 9586800 | 9591999 | high | 10.40 | 0.001144 |
| YWHAZ | 7534 | chr8 | 100939000 | 100944399 | high | 10.80 | 0.000075 |
| YY1 | 7528 | chr14 | 100274600 | 100277599 | high | 6.00 | 0.000075 |
| YY1 | 7528 | chr14 | 100250400 | 100254599 | high | 8.40 | 0.000075 |
| ZADH2 | 284273 | chr18 | 75207800 | 75209199 | low | -6.08 | 0.003780 |
| ZAK | 51776 | chr2 | 173202800 | 173205399 | low | -11.30 | 0.000299 |
| ZAK | 51776 | chr2 | 173187400 | 173189799 | low | -10.43 | 0.000299 |
| ZAK | 51776 | chr2 | 173176600 | 173178799 | low | -9.56 | 0.000299 |
| ZBED4 | 9889 | chr22 | 49856000 | 49860599 | high | 9.20 | 0.000198 |
| ZBED6 | 100381270 | chr1 | 203793400 | 203799799 | high | 12.80 | 0.009069 |
| ZBTB10 | 65986 | chr8 | 80484800 | 80489399 | high | 9.20 | 0.000820 |
| ZBTB16 | 7704 | chr11 | 114158800 | 114163599 | high | 9.60 | 0.000067 |
| ZBTB16 | 7704 | chr11 | 114058600 | 114063599 | high | 10.00 | 0.000068 |
| ZBTB17 | 7709 | chr1 | 15951200 | 15955599 | high | 8.80 | 0.000483 |
| ZBTB17 | 7709 | chr1 | 15958600 | 15967399 | high | 17.60 | 0.000483 |
| ZBTB20 | 26137 | chr3 | 114595600 | 114598799 | high | 6.40 | 0.000228 |
| ZBTB20 | 26137 | chr3 | 114589000 | 114594599 | high | 11.20 | 0.000228 |
| ZBTB21 | 49854 | chr21 | 42007000 | 42012399 | high | 10.80 | 0.001187 |
| ZBTB24 | 9841 | chr6 | 109461600 | 109468199 | high | 13.20 | 0.000090 |
| ZBTB25 | 7597 | chr14 | 64467400 | 64470199 | high | 5.60 | 0.000118 |
| ZBTB32 | 27033 | chr19 | 35713600 | 35719799 | high | 12.40 | 0.000757 |
| ZBTB38 | 253461 | chr3 | 141418400 | 141421999 | high | 7.20 | 0.001792 |
| ZBTB38 | 253461 | chr3 | 141384000 | 141387999 | high | 8.00 | 0.001793 |
| ZBTB38 | 253461 | chr3 | 141339400 | 141344799 | high | 10.80 | 0.001793 |
| ZBTB41 | 360023 | chr1 | 197169200 | 197174599 | high | 10.80 | 0.001826 |
| ZBTB41 | 360023 | chr1 | 197197400 | 197203199 | high | 11.60 | 0.001826 |
| ZBTB43 | 23099 | chr9 | 126833200 | 126835199 | low | -8.69 | 0.000182 |
| ZBTB43 | 23099 | chr9 | 126804600 | 126806399 | low | -7.82 | 0.000182 |
| ZBTB47 | 92999 | chr3 | 42648200 | 42657199 | high | 18.00 | 0.002181 |
| ZBTB49 | 166793 | chr4 | 4288800 | 4292599 | high | 7.60 | 0.004321 |
| ZBTB6 | 10773 | chr9 | 122911800 | 122914599 | high | 5.60 | 0.000088 |
| ZZEF1 | 23140 | chr17 | 4110600 | 4115199 | high | 9.20 | 0.005629 |
| ZZEF1 | 23140 | chr17 | 4142200 | 4147199 | high | 10.00 | 0.005586 |
