# Supplementary material for: KDM2B and its peptides promote the stem cells from apical papilla mediated nerve injury repair in rats by intervening EZH2 function
Source: Cell Prolif. 2024 Oct 2;58(2):e13756. doi: 10.1111/cpr.13756 (PMC11839186; doi:10.1111/cpr.13756)
Supplement: Supplementary file 7 — Table S4. [file CPR-58-e13756-s006.pdf]

Table S4. The significant binding sites number and formation of KDM2B-peptides.

Peptide number	Microarray sequence number	Peptide point gray value (%)
KDM2B-PP7	5	96.96
KDM2B-PP1	46	100
	47	100
ConPP	83	0
	84	0
KDM2B-PP2	122	100
	123	97.26
KDM2B-PP3	131	100
	132	100
KDM2B-PP4	139	85.09
	142	100
KDM2B-PP5	151	100
	152	97.79
KDM2B-PP6	231	100
	232	95.43
