# Supplementary material for: KDM2B and its peptides promote the stem cells from apical papilla mediated nerve injury repair in rats by intervening EZH2 function
Source: Cell Prolif. 2024 Oct 2;58(2):e13756. doi: 10.1111/cpr.13756 (PMC11839186; doi:10.1111/cpr.13756)
Supplement: Supplementary file 8 — File S1. [file CPR-58-e13756-s007.pdf]

Supplementary file for

KDM2B and its Peptides Promote the Stem Cells from Apical Papilla Mediated Spinal Cord Injury Repair in Rat Models by Intervening EZH2 Function

Yangyang Cao *et al.*

Corresponding author: Zhipeng Fan, zpfan@ccmu.edu.cn.

This PDF file includes:

Supplemental file 1. The triplicates and original scans of western blot gels presented in the paper are shown with molecular weight marker. Blank delineation was explicated this blot cropped from different parts of the different gels.

Figure 1C

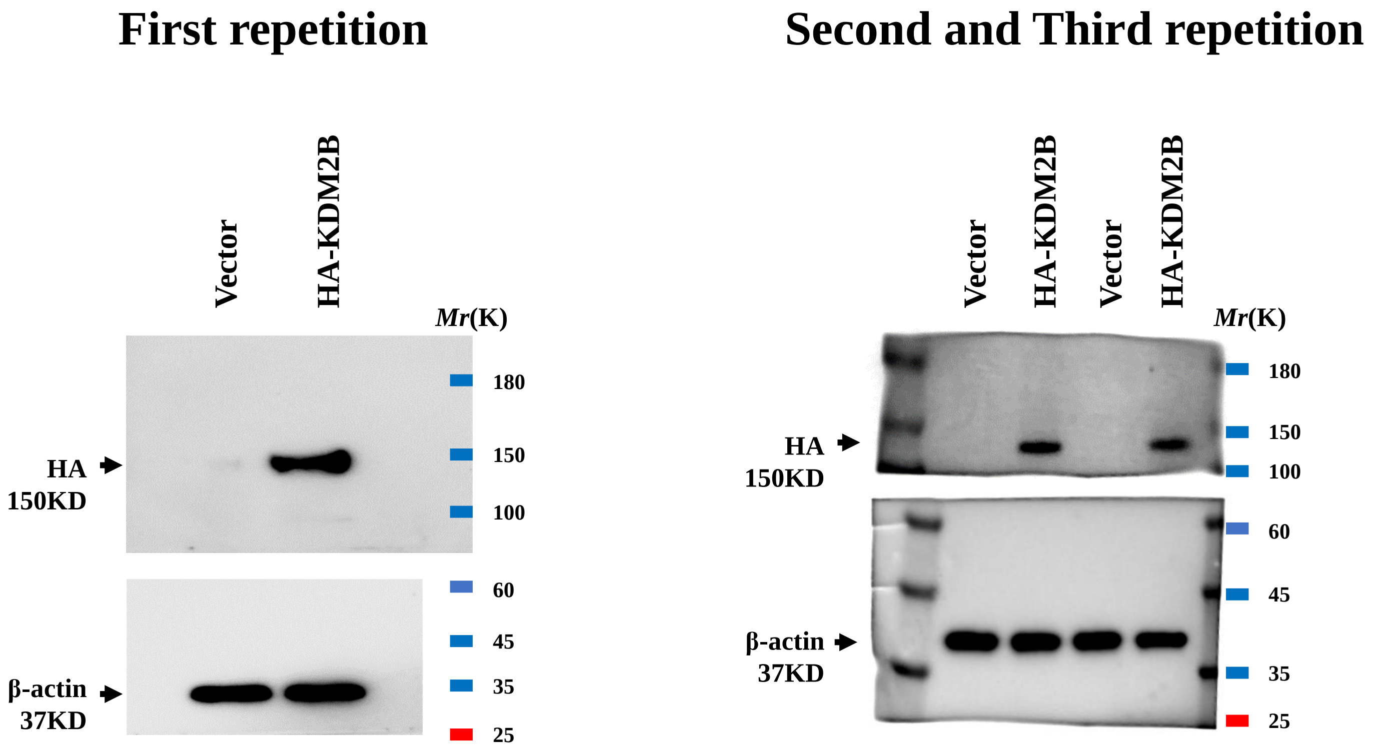
First repetition
Second and Third repetition
Vector
HA-KDM2B
Vector
HA-KDM2B
Vector
HA-KDM2B
Mr(K)
Mr(K)
HA
150KD
β-actin
37KD
HA
150KD
β-actin
37KD
180
150
100
60
45
35
25
180
150
100
60
45
35
25

Figure 4C

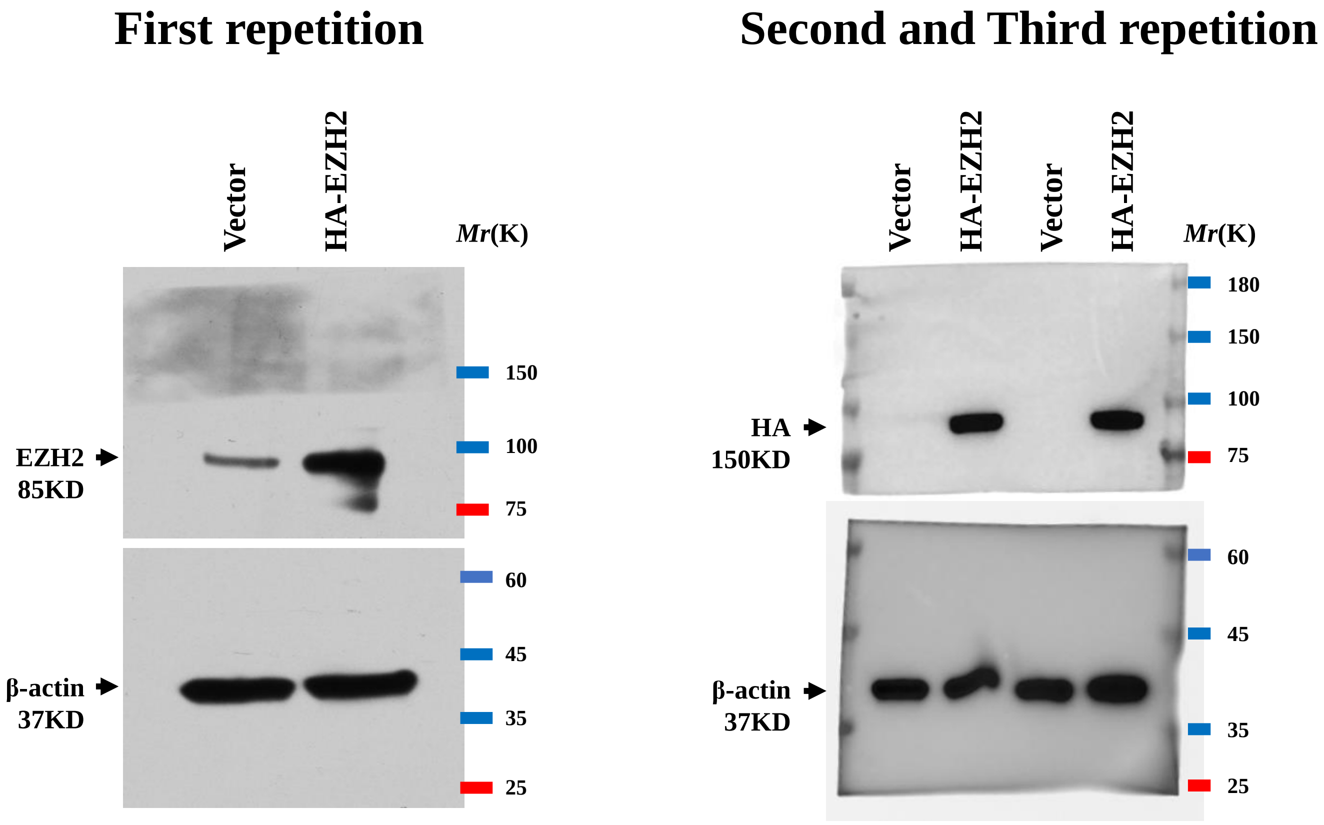
First repetition
Vector
HA-EZH2
Mr(K)
150
EZH2
85KD
100
75
60
β-actin
37KD
45
35
25
Second and Third repetition
Vector
HA-EZH2
Vector
HA-EZH2
Mr(K)
180
150
100
HA
150KD
75
60
45
β-actin
37KD
35
25

Figure 4P

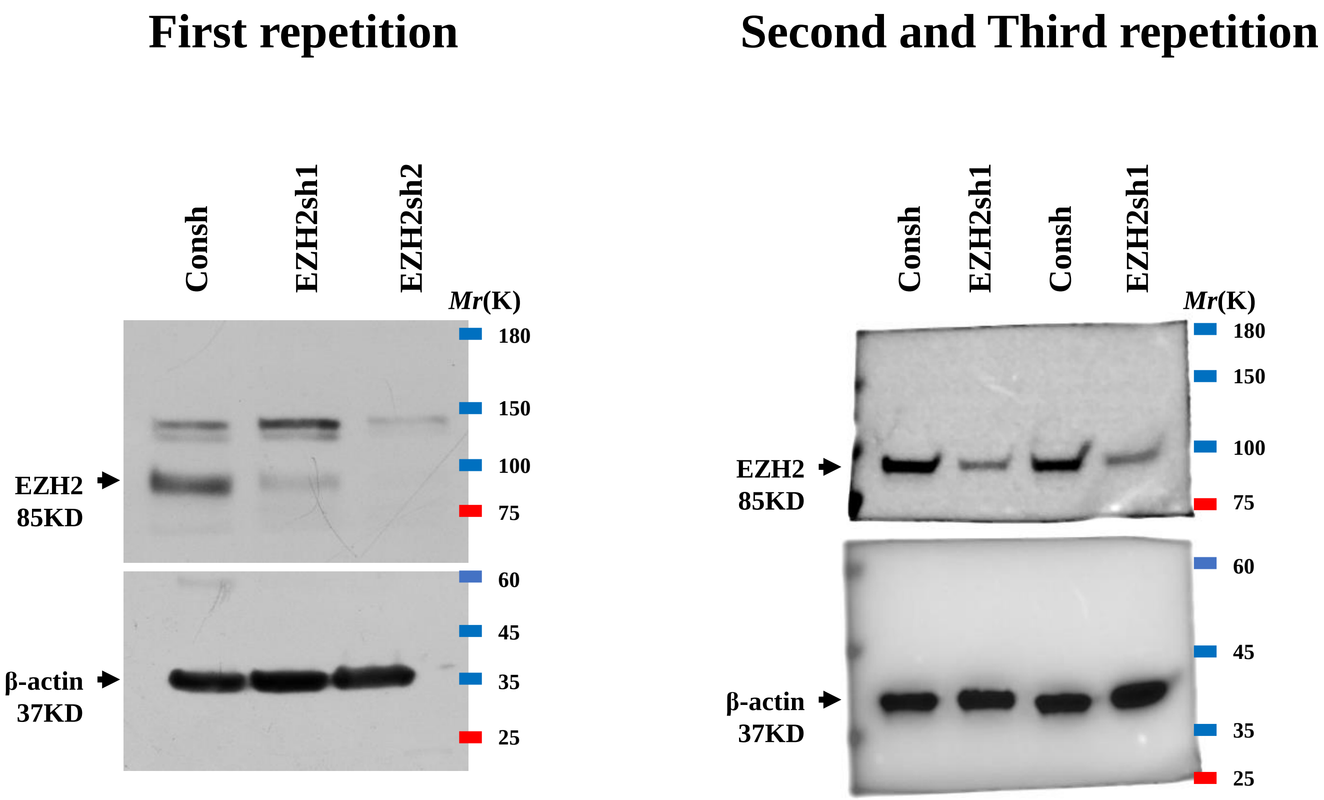
First repetition
Second and Third repetition
Consh
EZH2sh1
EZH2sh2
Consh
EZH2sh1
Consh
EZH2sh1
Mr(K)
180
150
100
75
60
45
35
25
EZH2
85KD
β-actin
37KD
EZH2
85KD
β-actin
37KD
Mr(K)
180
150
100
75
60
45
35
25

Figure 4E

First repetition

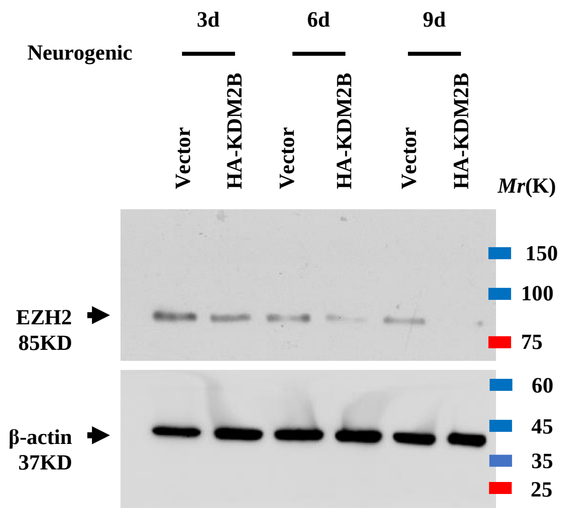

Second repetition

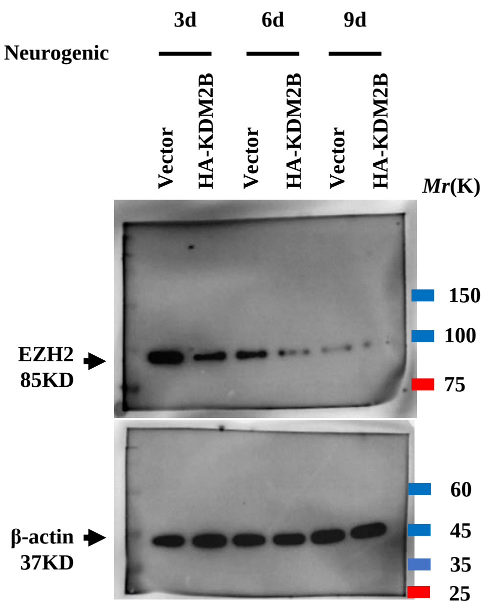

Third repetition

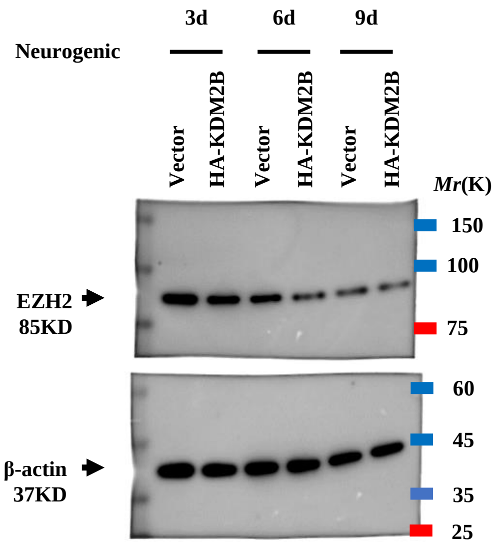

Figure 5A

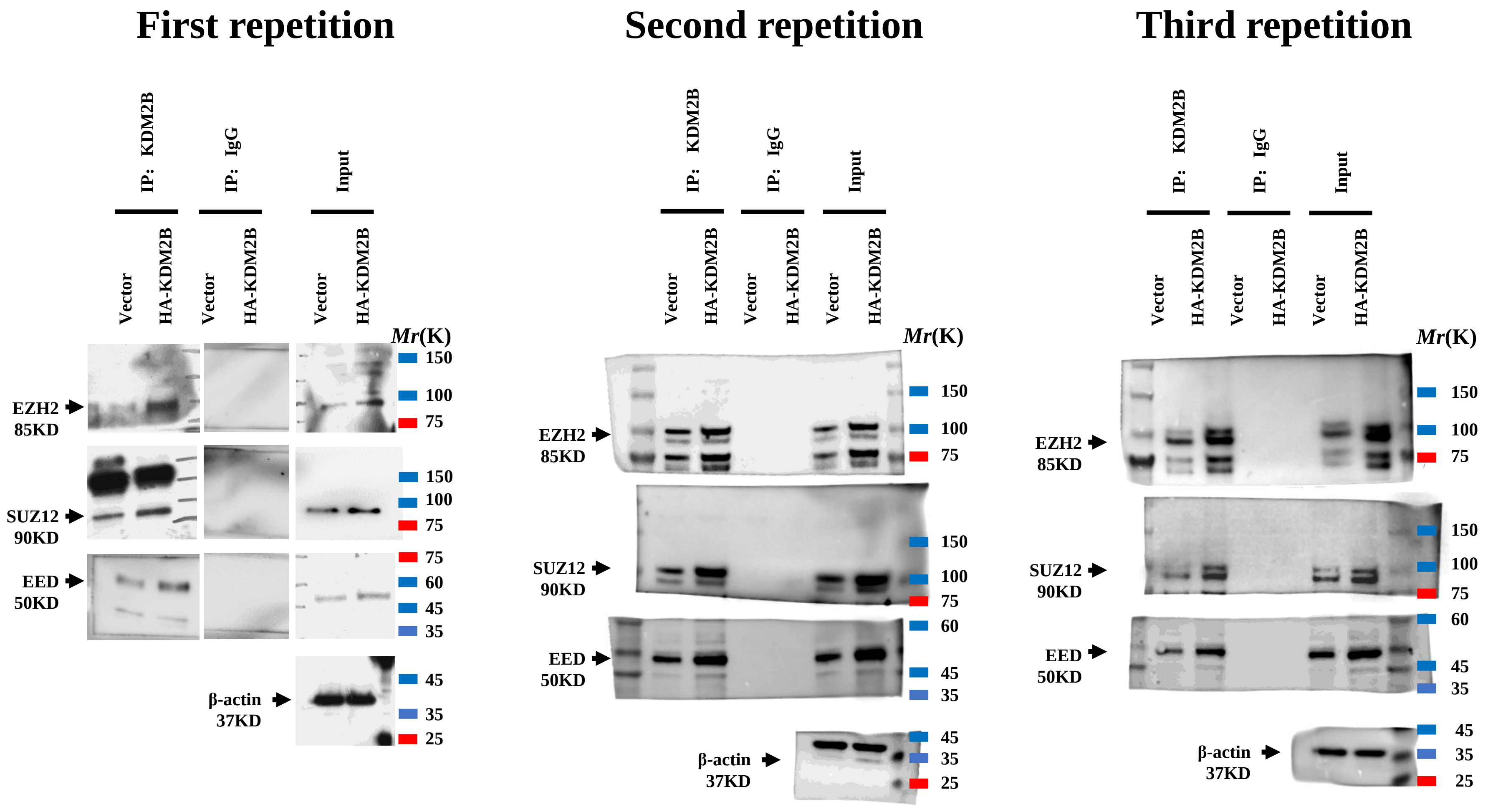

First repetition
Second repetition
Third repetition
IP: KDM2B
IP: IgG
Input
Vector
HA-KDM2B
Mr(K)
EZH2
85KD
SUZ12
90KD
EED
50KD
β-actin
37KD
150
100
75
60
45
35
25

Figure 5B

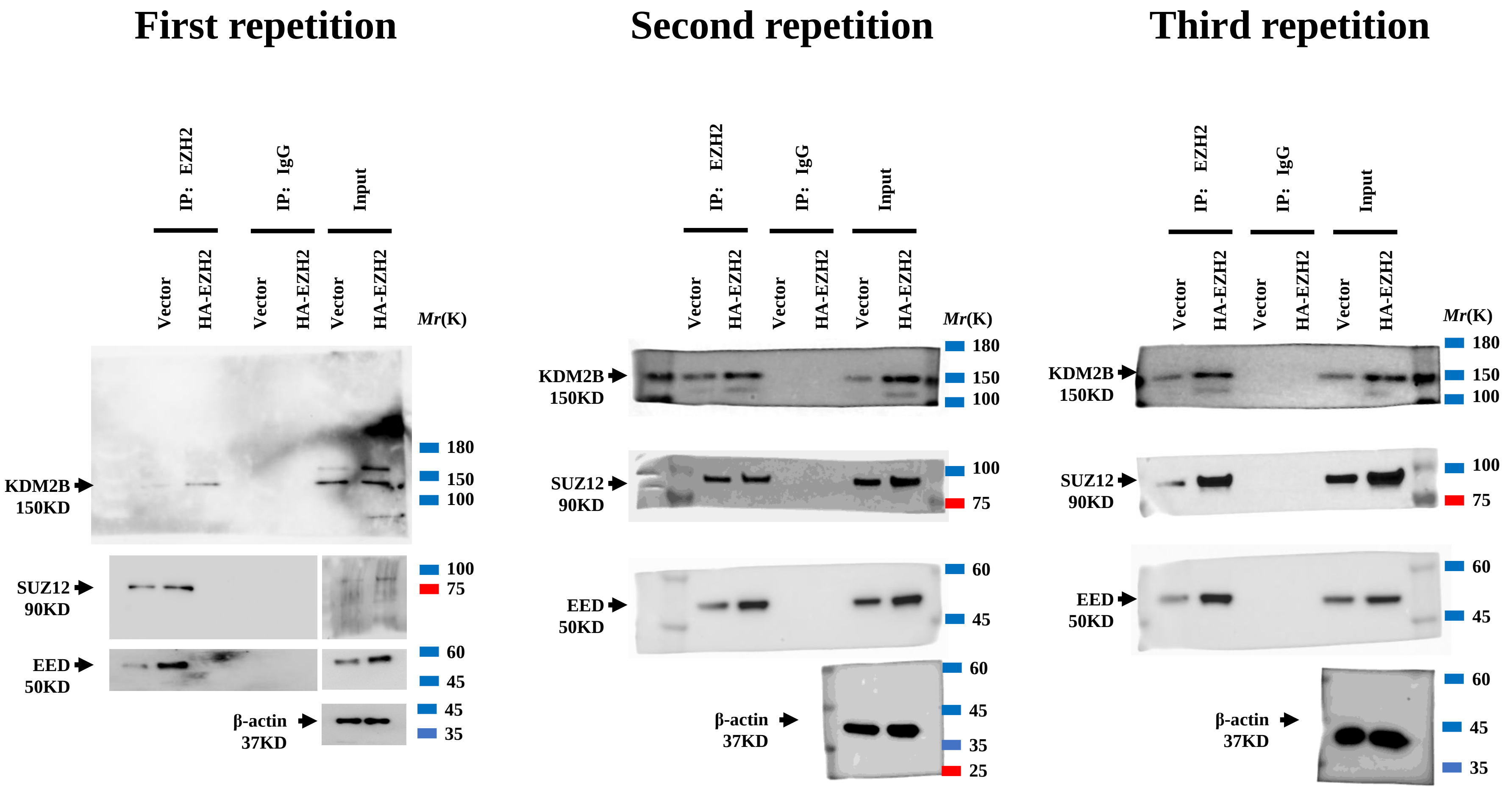
First repetition
Second repetition
Third repetition
IP: EZH2
IP: IgG
Input
Vector
HA-EZH2
Mr(K)
KDM2B
150KD
SUZ12
90KD
EED
50KD
β-actin
37KD
180
150
100
75
60
45
35
25

Figure 5C

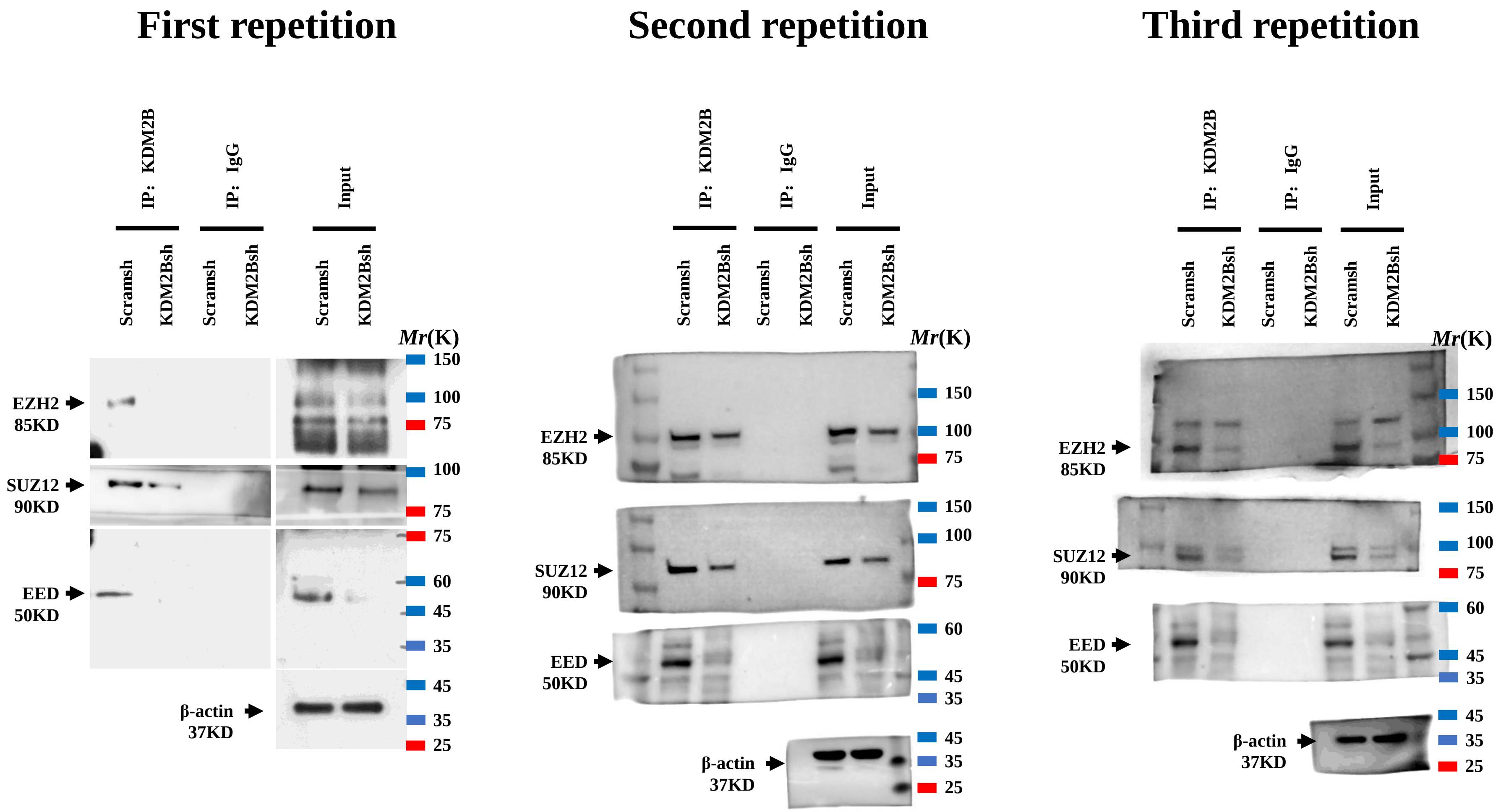
First repetition
Second repetition
Third repetition
IP: KDM2B
IP: IgG
Input
Scramsh
KDM2Bsh
Mr(K)
EZH2
85KD
SUZ12
90KD
EED
50KD
β-actin
37KD
150
100
75
60
45
35
25

Figure 5D

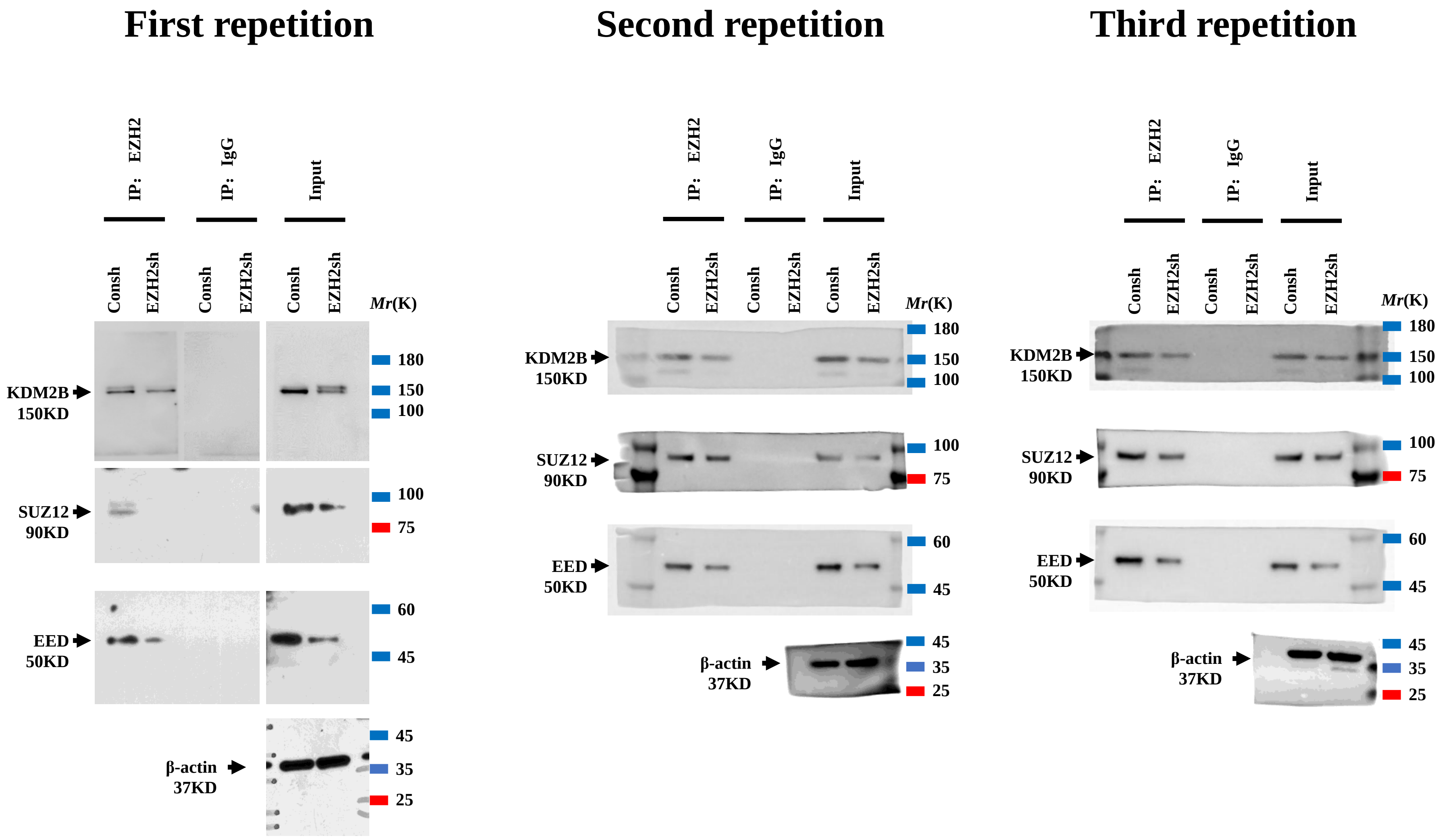
First repetition
Second repetition
Third repetition
IP: EZH2
IP: IgG
Input
Consh
EZH2sh
Mr(K)
KDM2B
150KD
SUZ12
90KD
EED
50KD
β-actin
37KD
180
150
100
75
60
45
35
25

Figure 5E

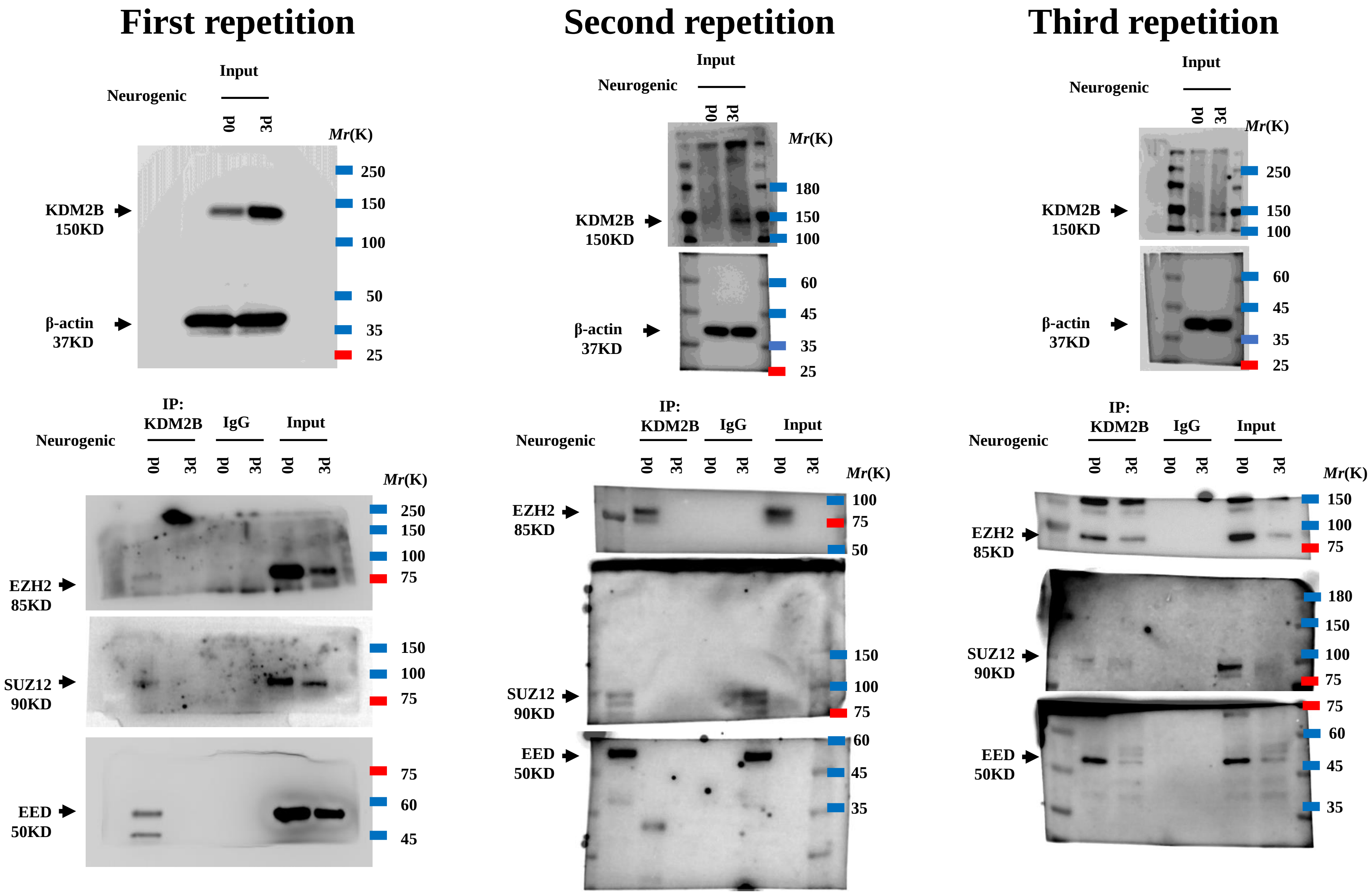
First repetition
Second repetition
Third repetition
Input
Neurogenic
0d
3d
Mr(K)
KDM2B 150KD
β-actin 37KD
250
180
150
100
60
50
45
35
25
IP: KDM2B
IgG
EZH2 85KD
SUZ12 90KD
EED 50KD
75

Figure 6D

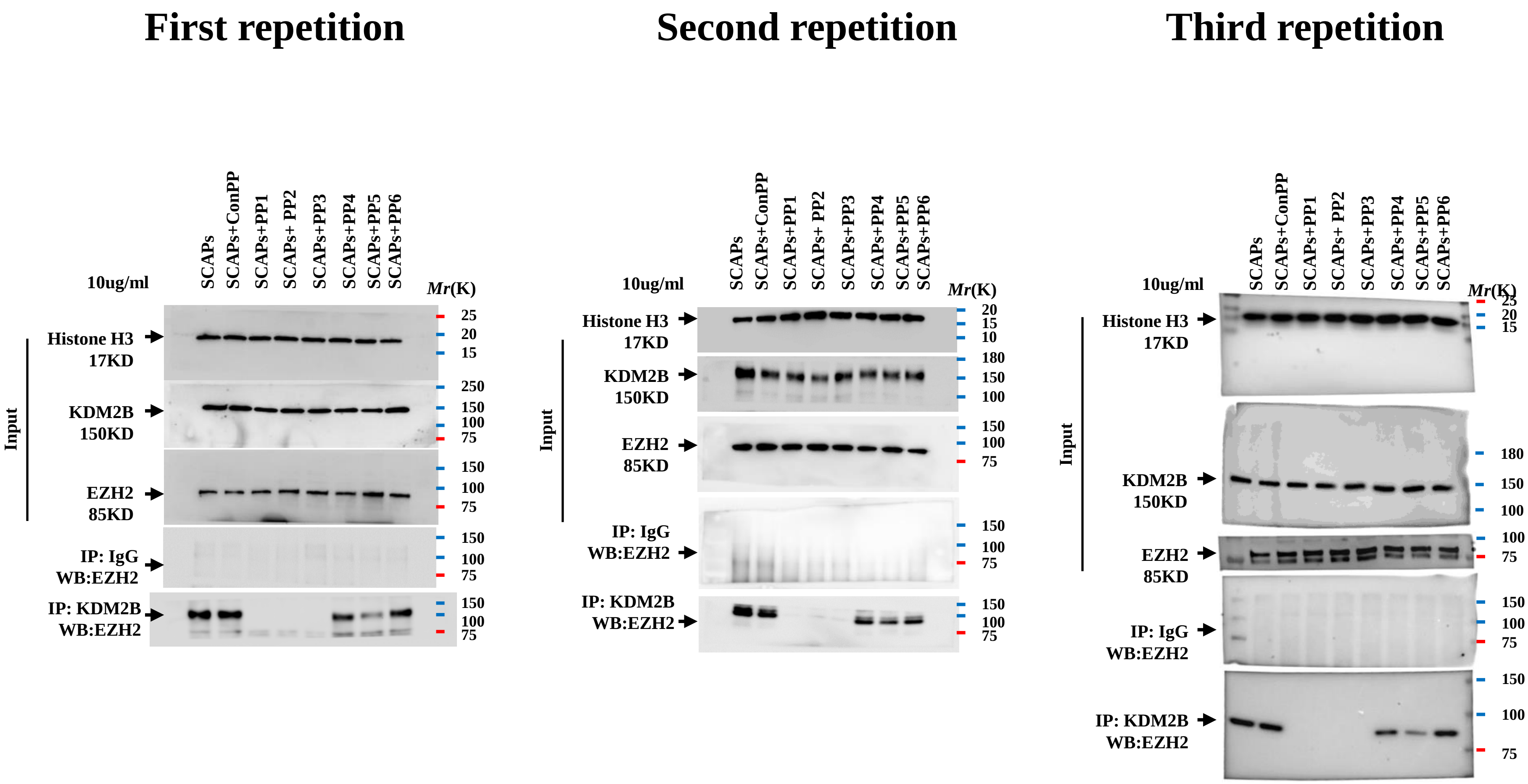
First repetition
Second repetition
Third repetition
10ug/ml
SCAPs
SCAPs+ConPP
SCAPs+PP1
SCAPs+ PP2
SCAPs+PP3
SCAPs+PP4
SCAPs+PP5
SCAPs+PP6
Mr(K)
Input
Histone H3
17KD
KDM2B
150KD
EZH2
85KD
IP: IgG
WB:EZH2
IP: KDM2B
WB:EZH2
25
20
15
250
180
150
100
75
10

Figure S2

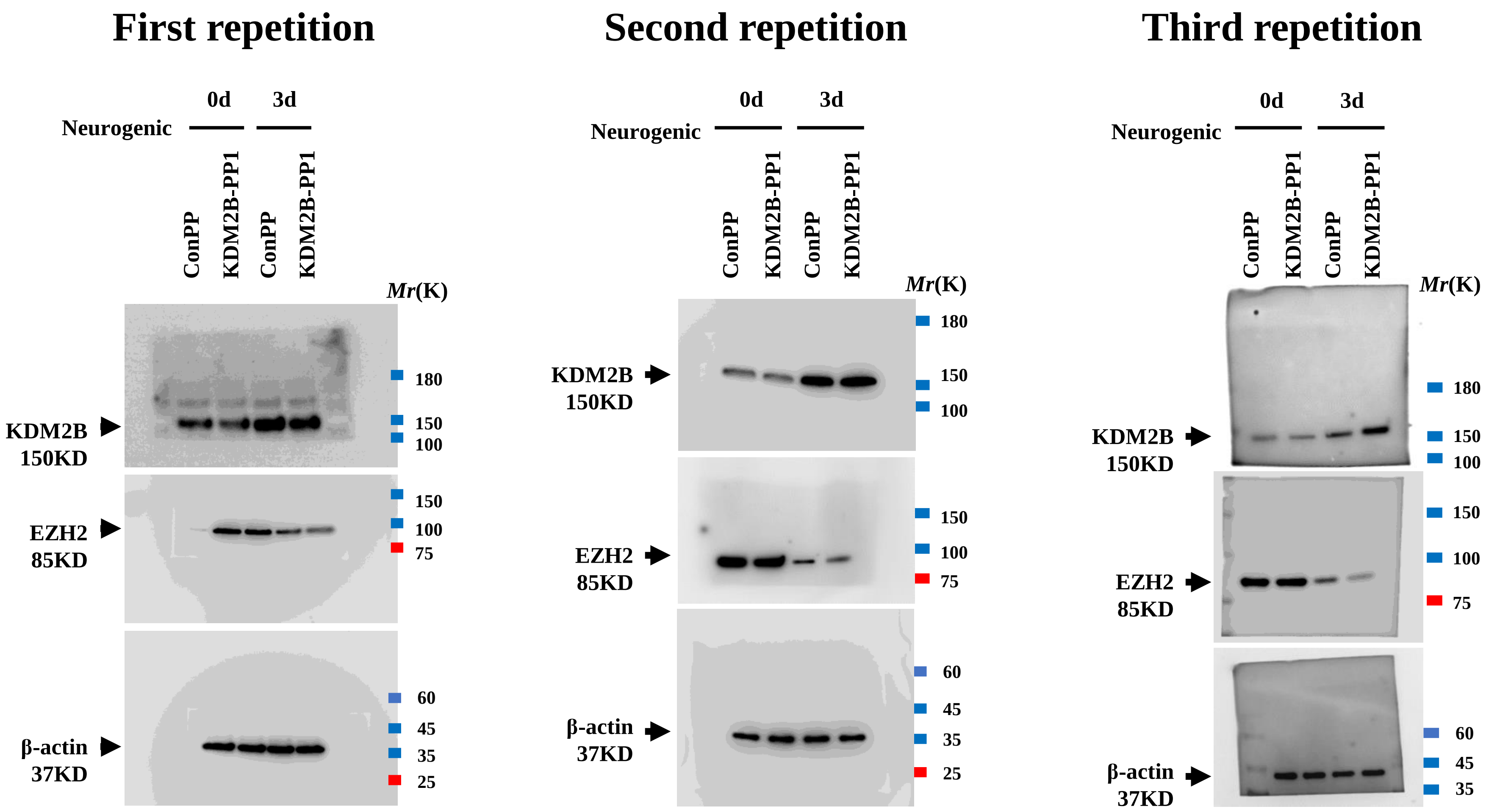
First repetition
Second repetition
Third repetition
Neurogenic
0d
3d
ConPP
KDM2B-PP1
ConPP
KDM2B-PP1
Mr(K)
KDM2B
150KD
EZH2
85KD
β-actin
37KD
180
150
100
75
60
45
35
25
